# SEAweb: the small RNA Expression Atlas web application

**DOI:** 10.1093/nar/gkz869

**Published:** 2019-10-10

**Authors:** Raza-Ur Rahman, Anna-Maria Liebhoff, Vikas Bansal, Maksims Fiosins, Ashish Rajput, Abdul Sattar, Daniel S Magruder, Sumit Madan, Ting Sun, Abhivyakti Gautam, Sven Heins, Timur Liwinski, Jörn Bethune, Claudia Trenkwalder, Juliane Fluck, Brit Mollenhauer, Stefan Bonn

**Affiliations:** 1 Institute of Medical Systems Biology, Center for Molecular Neurobiology, University Medical Center Hamburg-Eppendorf, 20251 Hamburg, Germany; 2 German Center for Neurodegenerative Diseases, 72076 Tübingen, Germany; 3 Genevention GmbH, 37079 Göttingen, Germany; 4 Fraunhofer Institute for Algorithms and Scientific Computing, Schloss Birlinghoven, 53757 Sankt Augustin, Germany; 5 Rheinische Friedrich-Wilhelms-Universität Bonn, 53113 Bonn, Germany; 6 Department of Neurogenetics, Max Planck Institute of Experimental Medicine, 37075 Göttingen, Germany; 7 Department of Medicine, University Medical Center Hamburg-Eppendorf, 20251 Hamburg, Germany; 8 Paracelsus-Elena-Klinik, 34128 Kassel, Germany; 9 Department of Neurosurgery, University Medical Center Göttingen, 37075 Göttingen, Germany; 10 Institute of Geodesy and Geoinformation, University of Bonn, 53115 Bonn, Germany; 11 German National Library of Medicine (ZB MED) - Information Centre for Life Sciences, 53115 Bonn, Germany; 12 Institute of Neurology, University Medical Center Göttingen, 37075 Göttingen, Germany

## Abstract

We present the Small RNA Expression Atlas (SEAweb), a web application that allows for the interactive querying, visualization and analysis of known and novel small RNAs across 10 organisms. It contains sRNA and pathogen expression information for over 4200 published samples with standardized search terms and ontologies. In addition, SEAweb allows for the interactive visualization and re-analysis of 879 differential expression and 514 classification comparisons. SEAweb's user model enables sRNA researchers to compare and re-analyze user-specific and published datasets, highlighting common and distinct sRNA expression patterns. We provide evidence for SEAweb's fidelity by (i) generating a set of 591 tissue specific miRNAs across 29 tissues, (ii) finding known and novel bacterial and viral infections across diseases and (iii) determining a Parkinson's disease-specific blood biomarker signature using novel data. We believe that SEAweb's simple semantic search interface, the flexible interactive reports and the user model with rich analysis capabilities will enable researchers to better understand the potential function and diagnostic value of sRNAs or pathogens across tissues, diseases and organisms.

## INTRODUCTION

Small RNAs (sRNAs) are a class of short, non-coding RNAs with important biological functions in nearly all aspects of organismal development in health and disease. Especially in diagnostic and therapeutic research, sRNAs such as miRNAs and piRNAs received recent attention (1). The increasing number of deep sequencing sRNA studies (sRNA-seq) is reflecting the importance of sRNAs in biological processes as well as disease diagnosis and therapy. In addition, recent evidence highlights the pivotal roles of viral and bacterial-derived sRNAs in the pathogenesis of infectious diseases, across the animal and plant kingdoms (2–4). Viral sRNAs play vital roles in the viral replication, persistence, the immune escape and host cell transformation (2,3). Many DNA and RNA viruses encode various classes of small RNAs, which associate with host RNAs and proteins and affect their stability and function. The introduction of sRNA deep sequencing (sRNA-seq) allowed for the quantitative analysis of sRNAs of a specific organism, but its generic nature also enables the simultaneous detection of microbial and viral reads. sRNA-seq data therefore naturally lends itself for the analysis of host-pathogen interactions, which has been recently exemplified for RNA-seq data (5). Using the vast amount of publicly available sRNA-seq data in repositories such as Sequence Read Archive (SRA) (6) and Gene Expression Omnibus (7) enables the unbiased charting of viral and bacterial infections across tissues, diseases, species, age and sex. This would not only highlight novel causal or comorbid relationships between diseases and infections, it could also shed mechanistic insights onto how the infectious agent interacts with and modifies the host cell. To harvest the true potential of existing data, it is important to allow for querying, visualization and analysis of sRNA-seq data across organisms, tissues, cell types and disease states. This would allow researchers, for example, to search for disease-specific sRNA or pathogenic biomarker signatures across all disease entities investigated. Data integration and interoperability require (i) a streamlined analysis workflow to reduce analysis bias between experiments (ii) also necessitates standardized annotation using ontologies to search and retrieve relevant samples and (iii) flexible and interactive visualization of the data.

To date, several web-based sRNA-seq expression profile databases are available that differ in their level of information, portfolio, performance and user-friendliness. Recent additions to sRNA web based databases include miRmine (8), provides expression of a single or multiple miRNAs for a specific tissue, cell-line or disease. Results are displayed in multiple interactive, graphical and downloadable formats. miratlas (9) allows for searching miRNA expression profiles as well as sRNA-seq experiments and provides information on the miRNA modification analysis. YM500v3 (10) provides interactive web reports on sRNA expression profiles, novel miRNA expression profiles, miRNA modification analysis, sRNA differential expression and miRNA gene targets. SPAR (11) is a user-friendly web server for the analysis, annotation and visualization of sRNA-seq data. It provides expression profiles of 10 different types of sRNAs across different tissues and cell types of human (hg19, hg38) and mouse (mm10). Currently, SPAR is the only tool that allows users to compare their input experimental data against the reference datasets from ENCODE (12) and DASHR (13). Moreover, it supports different genome versions of an organism. DASHR2 (14) supports sRNA expression profiles across different genome versions of the same species across tissues and cell types and supports 10 types of sRNAs. Results are provided in an interactive manner, such as sncRNA locus sorting and filtering by biological features. All annotation and expression information are downloadable and accessible as UCSC genome browser tracks.

Although many good web platforms for the sRNA-seq data exist, some important aspects for storing and searching have yet to be integrated. For example, no current web application allows for the ontology based search of sRNA-seq experiments. Current tools lack an important association of miRNAs with disease. miRNA disease associations are provided by HMDD (15), but it does not provide miRNA expression information. Except for YM500v3, current tools do not provide miRNAs and gene targets. Of note YM500v3 is only limited to cancer miRNome studies. Also, there is currently no web application that allows for the identification of biomarkers of disease via machine-learning. The above mentioned web platforms do not provide expression of novel miRNAs in known disease state or tissues, including the structure and probability of the novel miRNA prediction. To our knowledge no other data repository provides pathogenic signatures from sRNA-seq data including their differential expression in healthy and diseased condition. Except for SPAR, current sRNA-seq web services do not allow for the user data upload. At last, in current tools users can only search for the results that are stored in the database, there is no option for the users to reanalyze data with the samples of their choice. This feature would greatly facilitate researchers to perform differential expression between male and female of an experiment or to compare old aged patients (samples) with young ones in the same group. In the end, these functionalities should be paired with a flexible and interactive visualization of the sRNA-seq data supporting more species and cross study comparisons.

In order to address the above mentioned limitations, we hereby present the **s**mall-RNA **E**xpression **A**tlas (SEAweb), a web application that allows for querying, visualization and analysis of over 4200 published sRNA-seq expression samples. SEAweb automatically downloads and re-analyzes published data using Oasis 2 (16), semantically annotates relevant meta-information using standardized terms (the annotations are later checked and corrected manually), synchronizes sRNA information with other databases, allows for the querying of terms across ontological graphs and presents quality curated sRNA expression information as interactive web reports. In addition, SEAweb stores sRNA differential expression, sRNA based classification, pathogenic sRNA signatures from bacteria and viruses and pathogen differential expression. Gene targets and disease associations for miRNAs are also incorporated into SEAweb.

One of the most useful features of SEAweb is to enable users to upload their analysis results of differential expression and classification from Oasis 2. This allow users to compare their data to over 4200 experimental samples across different conditions. Using SEAweb's interactive visualizations, users can upload their data into their own workspace, select the published datasets to compare to, and define if differential expression or classification results should be compared. SEAweb also provides users with an option to perform on the fly analysis such as overlapping differentially expressed (DE) sRNAs or pathogens across different studies or the most important features (sRNAs) identified with classification. At last, SEAweb enables end users to re-submit samples from interactive plots for differential expression or classification, this helps users to choose samples of their choice from an experiment. It currently supports 10 organisms (Table 1) and is continuously updated with novel published sRNA-seq datasets and relevant sRNA information from various online resources. A detailed comparison of SEAweb to other existing sRNA expression databases (Table 2) highlights that SEAweb is superior in terms of supported organism, ontological annotations, diseases, tissues, sRNA based classification, pathogen k-mer DE, known miRNA disease associations, user specific experimental data upload, cross study comparisons and re-analysis with selected samples. SEAweb contains over 4200 samples in its database, which is considerably less than YM500v3, which hosts over 8000 cancer samples. It is to be noted, however, that the YM500v3 database only supports cancer datasets and no other disease types (Table 2). Additionally, SEAweb also stores in-house data (for a month) from the end users to enable comparison with the data in SEA.

**Table 1.** Supported SEAweb organisms and their corresponding genome versions

Organism	genome-version	genome-date
*Bos taurus*	UMD3.1	2009-11
*Caenorhabditis elegans*	WBcel235	2012-12
*Danio rerio*	GRCz10	2014-09
*Drosophila melanogaster*	BDGP6	2014-07
*Mus musculus*	GRCm38	2012-01
*Gallus gallus*	Galgal4	2011-11
*Rattus norvegicus*	Rnor_6.0	2014-07
*Homo sapiens*	GRCh38	2013-12
*Sus scrofa*	Sscrofa10.2	2011-08
*Anopheles gambiae*	AgamP4	2006-02

**Table 2.** Comparison of sRNA expression databases

Feature	SEAweb	miRmine^1^	DASHR2^2^	miratlas^3^	YM500v3^4^	SPAR^5^
Organisms	10	1	1	2	1	2
sRNA types	5	1	10	1	5	10
Samples	>4200	304	802	461	>8000*	365^$^
Novel miRNAs	+				+	
Ontology search^#^	+					
sRNA DE	+				+	
sRNA classification	+					
Pathogen k-mer expression	+					
Pathogen k-mer DE	+					
miRNA targets	+				+	
miRNA disease associations	+					
User data upload	+					+
Cross study comparisons	+					+
Re-analysis with selected samples	+					
Dataset search	+			+		+
Genome versions			+			+
Modification analysis				+	+	
Tissue specificity			+			+

This table includes recent sRNA expression databases and a list of features we deem relevant.

*Supports mainly cancer-related datasets.

^#^Use of ontological graphs for the annotation and querying of samples.

^$^Number of datasets based on ^2^(14) (information about number of samples cannot be obtained).

^1^(8), ^2^(14), ^3^(9), ^4^(10), ^5^(11). For number of samples per organism, see Supplementary Material Table S5.

## MATERIALS AND METHODS

### User data

In case users want to upload their in-house data for comparing it to all the available data in SEAweb, they need to create an account. User-DB, stores their account information as well as sRNA-seq data uploaded by the users. Moreover, user uploaded data is shown only from their respective account and is not available to other users. Users have the option to include their data in the SEAweb for a limited time (30 days). We do not provide users to include their data in the SEAweb permanently or publicly for several reasons: (i) these data are unpublished and we can run into data protection issues. (ii) The ontological annotations of these data by the end users might not be consistent with ours and hence not comparable. (iii) Users might not want to provide information about their experiments such as tissue or disease etc. (iv) End users might not be able trust the system, if anyone could add any quality of data. Data that are added by us follows a manual curation for quality checks. With these measures, we encourage users to upload their data (temporarily), without any data protection issues.

### sRNA tissue specificity

To compute tissue specificity indices (TSI) for human sRNAs we calculated median of reads per million (RPM) expression per dataset and tissue. sRNAs with a median RPM expression of at least three were considered in all the tissue specificity analysis. Moreover, sRNAs which had no expression in any tissue and tissues with no sRNAs expression were excluded from the TSI analysis. Healthy and diseased samples were mixed for tissues within the same dataset (Figure 2; Supplementary Figure S3 and Table S1). To remove potential biases introduced by diseased samples we also calculated TSI for non-diseased samples only (Supplementary Figures S2-3, Tables S1 and 4). These analyses were performed for two sets of sRNAs, miRNAs (Figure 2; Supplementary Figure S2 and Table S1) and all non-miRNA sRNAs including piRNA, snoRNA, snRNA and rRNA in SEAweb (Supplementary Figures S3-4 and Table S4). Shannon entropy from BioQC R package was used to calculate TSI for each miRNA across tissues. In the end, 1522 miRNAs across 64 datasets were considered for miRNA tissue specificity in healthy and diseased mixed samples, 1365 miRNAs across 43 datasets were considered for miRNA tissue specificity in non-diseased samples, 4300 sRNAs (piRNA, snoRNA, snRNA and rRNA) across 64 datasets were considered for sRNA tissue specificity in healthy and diseased mixed samples, and 1672 sRNAs (piRNA, snoRNA, snRNA and rRNA) across 43 datasets were considered for sRNA tissue specificity in non-diseased samples (Supplementary Tables S1 and 4).

### Novel miRNA gene targets

miRDB (17) was used to obtain targets of the novel miRNAs. We restricted the analysis to highly probable gene targets having a score of 70 or more.

### Text mining pipeline

To extract miRNA–gene targets, a dedicated text mining pipeline that reads unstructured text data and outputs structured data that includes the detected and normalized genes and miRNAs as well as the relations between them. Named entity recognition software ProMiner (18) and MiRNADetector (19) are used to detect and normalize genes and miRNAs, respectively. Both detectors are incorporated in the BELIEF text mining pipeline (20) that contains machine learning models to detect specific relations from the complete Medline abstracts.

### Gene enrichment analysis

Gene enrichment analysis was performed using webgestalt R package version 0.3.0.

### In-house Parkinson’s disease data

#### Isolation of total RNA from peripheral blood sample

Peripheral blood samples were collected into PAXgene Blood RNA tube (PreAnalytiX) from consenting patients and healthy controls, the tubes were gently inverted for multiple times, incubated for 20–24 h under room temperature and stored under −80°C until processing. Total RNA was isolated using the PAXgene Blood RNA kit (PreAnalytiX) according to the manufacturer's protocol. The purity and concentration of isolated RNA were measured with NanoDrop™ 2000 spectrophotometer (Thermo Fisher Scientific). The RNA integrity was determined by Agilent RNA 6000 Nanochip (Agilent Technologies) using the 2100 Bioanalyzer (Agilent Technologies).

#### Small RNA library preparation

Small RNA libraries were prepared using 1 µg high-quality RNA following the protocol of Illumina TrueSeq small RNA library kit (Illumina). In brief, 3′adapter was denatured for 2 min under 70°C, and ligated to the RNA with T4 RNA Ligase 2 deletion mutant for 1 h at 28°C. Then the reaction was stopped with stop solution for 15 min under 28°C. Subsequently, 5′ adapter was denatured for 2 min at 70°C, then added to the RNA with adenosine triphosphate and T4 DNA ligase for 1 h under 28°C. After adaptors ligation, the RNA was reverse transcribed to complement DNA (cDNA) by using SuperScript II Reverse Transcriptase (Thermo Fisher Scientific) and dNTPs for 1 h at 50°C. Then, the cDNA was indexed and amplified with polymerase chain reaction (PCR) mix and primers supplied in the kit for 12 cycles (denaturing at 98°C for 30 s, annealing at 60°C for 30 s, extension at 72°C for 15 s, with a final extension at 72°C for 10 min). Amplified and indexed cDNAs were then pooled together, mixed with DNA loading dye and loaded on a 5% Tris-borate-EDTA (TBE) acrylamide gels (Bio-Rad). After 57 min electrophoresis under 145 V, the gel was stained with Midori Green for 5 min and viewed under the UV transilluminator, fragments between Illumina's custom ladder 145 and 160 bp were cut out for library preparation. The gel was centrifuged at 20 000 × *g* for 2 min through a Gel Breaker tube (Bio-Cat). Then cDNA was eluted from the homogenized gel by adding 300 µl UltraPure water and shaking under 800 × rpm for 2 h. Then the gel was transferred on a 5 um filter tube (Bio-Cat) and centrifuged for 10 s under 600 × *g* and the gel debris was separated. Afterward, 2 µl Glycoblue, 30 µl of 3M sodium acetate and 975 µl 100% ethanol (pre-chilled under −20°C) were added and well mixed to the sample, following an immediate centrifuge at 20 000 × *g* for 20 min under 4°C. After remove and discard the supernatant, the pellet was washed with 500 µl 70% pre-chilled ethanol. The supernatant was discarded after sample being centrifuged at 20 000 × *g* for 2 min under room temperature, and the pellet was dried in a 37°C heat block for 10 min with open lid. At last, the pellet was resuspended in 10 µl 10 mM Tris–HCL (pH 8.5) and the sample quality was checked using Agilent High Sensitivity DNA chip (Agilent Technologies) using the 2100 Bioanalyzer (Agilent Technologies). All high quality libraries were then sequenced on Illumina HiSeq 2000 Sequencer.

### Classification feature pruning

We used Oasis 2 to identify Parkinson's disease (PD) biomarker using 47 PD and 53 frequency-matched healthy controls. For classification analysis, we used all small RNAs (*n* = 49 965) in Oasis 2. The random forest (RF) classifier in Oasis 2 selected these 18 sRNAs by filtering for informative features while removing the non-informative ones. In brief, The RF selects part of the features for the construction of each tree (mtry parameter, which is by default equal to √n where n is total number of features). If there is a big number of non-informative features (‘noise’), many trees can be build based on noise only and therefore affect the classification quality. The way to avoid trees built of noise is feature pruning. The idea is to arrange the variables based on their importance in the full model and then remove less important variables one-by-one, calculating model performance at each step. At the end, the subset of variables with the best performance are considered as important features. We used cross-validation-based backward selection, implemented in the R caret package with 10-fold cross-validation, repeated 10 times at each step for the performance calculation.

## SYSTEM DESIGN

**Figure 1.**
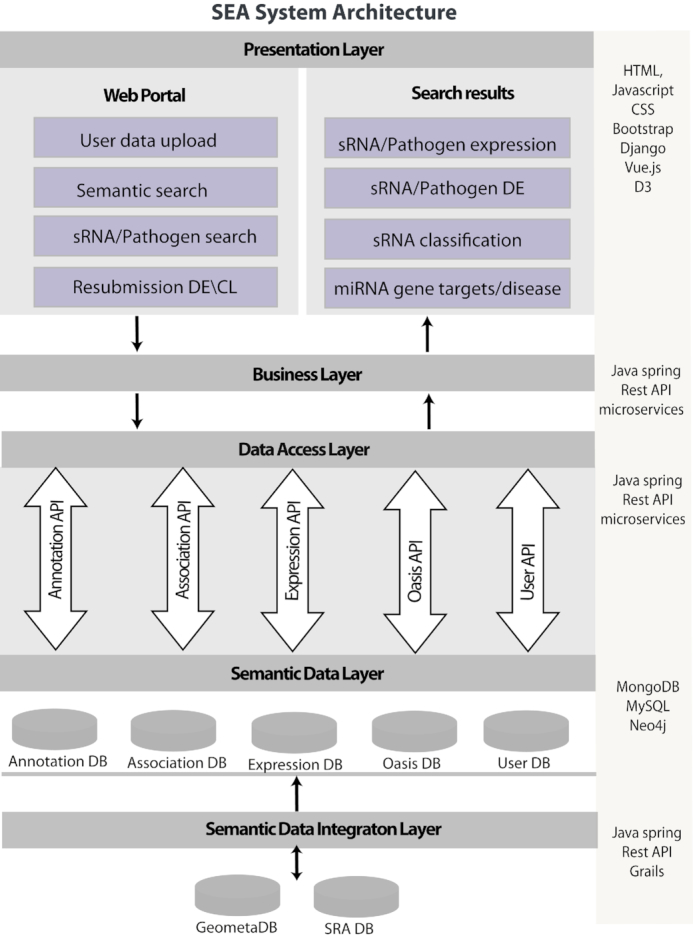
SEAweb system architecture. SEAweb system was developed using the modular system design approach (model-view-controller). The system has a presentation layer for user interface and visualization of search results. Presentation layer is followed by a business layer which transform complex user queries and distribute particular requests to the data access layer REST API services. There is a semantic data layer, to store and access primary and derived data together with annotations and links to secondary data. Annotation-DB stores metadata for experiments, samples, corresponding ontological terms as well as relations between dataset/sample, sample/term and term/term. Association-DB contains information about sRNAs and genes chromosomal locations, miRNA target genes and miRNA disease associations. Expression-DB stores sRNA expression profiles, sRNA differential expression; sRNA based classification as well as pathogen detection and pathogen differential expression. It also store details about dataset processing pipeline and parameters. Oasis-DB was used to store novel predicted miRNA information. User DB contains in-house data uploaded by the end users from Oasis 2 pipeline. Semantic data integration layer integrates primary and secondary data into the mentioned databases. Microservices were implemented in order to achieve strong encapsulation and well-defined interfaces via REST APIs.

SEAweb stores sRNA expression information, sRNA differential expression, sRNA-based classification, pathogenic sRNA signatures from bacteria and viruses, pathogen differential expression, miRNA gene targets and disease association as well as deep and standardized metadata on the samples, analysis workflows and databases used. Meta-data information is normalized using ontologies to allow for standardized search and retrieval across ontological hierarchies (section ‘Semantic data layer’ and Supplementary Material). The following sections will detail the system design of SEAweb (Figure 1).

### Acquisition and analysis of sRNA datasets

SEAweb acquires raw published sRNA-seq datasets and their primary annotation from Gene Expression Omnibus (GEO) and NCBI’s Sequence Reads Archive (SRA) repository (Supplementary Material). Novel datasets are downloaded and stored in SEAweb's raw data repository while corresponding annotations are stored in SEAweb's annotation database and are manually curated. In order to retrieve relevant samples for downloading, we optimized our search queries to look for the datasets that have, (i) Experiment type as non-coding RNA profiling by high throughput sequencing, (ii) Sequencing platform as Illumina, (iii) Tissue, cell type, disease or cell line information and (iv) is one of the 10 organisms that SEAweb supports at the moment (Table 1). Raw data are downloaded and subsequently processed automatically by SEAweb's sRNA analysis workflow using Oasis 2.0 (http://oasis.ims.bio/) (Supplementary Material). Subsequently, sRNA counts of high-quality samples are stored in the sRNA expression database. For all the experiments with samples from different conditions such as disease, tissue, cell line or cell type; sRNA differential expression and classification was performed within the experiment using Oasis 2. All possible comparisons for an experiment were taken into account such as healthy versus disease stage 1, healthy versus disease stage 2, disease stage 1 versus disease stage 2 as explained in Supplementary Section 3.4. Additionally, differential expression analysis of detected pathogens was performed using DESeq2 package (21). In order to reduce bias that could be introduced into the data by using different analysis routines, every sample in SEAweb has been analyzed by identical analysis workflows using identical databases and genome versions. Moreover, SEAweb stores analysis workflow parameters used to analyze the samples such as adapter sequence, genome, number of mismatches, minimum and maximum read lengths along with the versioning information about the software and databases used for the analysis. In case of changes in databases or analysis routines, we completely re-analyze all SEAweb’ data for consistency.

Additionally, sample annotations are processed automatically with SEAweb's annotation workflow. Processed files and annotations are subsequently semi-automatically curated (Supplementary Sections 2.3 and 3).

### Data storage

Once the raw sequencing data is analyzed, the next step is to store the analysis results to the database for downstream analysis and querying. Most metadata is quite different between experiments. Some experiments may have information such as disease, tissue, cell line, gender, age of patient while others may completely lack this. Due to this sparse nature of the biological experimental data, we opted to use NoSQL database management systems such as MongoDB and Neo4J for hierarchical (connected) normalized data. A multi-database management system architecture was used to store different types of data:

In brief, **Expression-DB** is created to save sRNA expression profiles, sRNA differential expression, sRNA based classification as well as pathogen detection and pathogen differential expression. This database stores the identification and description of the experiment (dataset), information about dataset processing (pipeline information and parameters), information about samples. **Association-DB** is used to store genomic coordinates for sRNAs, miRNA gene targets and miRNA diseases association. It contains information about sRNA’s and gene's chromosomal locations, miRNA target genes and miRNA disease associations. Chromosomal coordinates were obtained from miRBase version 21 (22), ensemble version 84 (23) and piRNA bank (24), miRNA gene targets were obtained from mirTarBase version 7.0 (25) as well as from BELIEF text mining pipeline (20) (‘Materials and Methods’ section), miRNA disease associations were obtained from HMDD database version 2.0 (15). In order to support the aggregation and comparison of these different types of data we normalized the identifiers across databases. To enable search by ontological terms, **Annotation-DB** is created using the Neo4J database management system. Neo4J is a graph database, representing elements as graph nodes or vertices. **Annotation-DB** (supplementary Figure S1) stores the following three node types: (i) Experiments (datasets), this type of node stores information about the experiment such as description of the experiment, reference to database, experimental design and any global level information, which is common among all the samples. (ii) Sample node type is used to store information about individual sample, such as description of a sample, reference to database, sample-specific processing parameters. (iii) Annotation term node type stores annotation term information of samples such as organism, disease, tissue, cell type, cell line, age, gender, condition (treated\untreated) and extracted molecule for sequencing etc. We normalize organism with the NCBI taxonomy ontology (26), tissue with the BRENDA tissue ontology (27), disease with the human disease ontology (28), cell type with the cell ontology (29) and cell line with the cell line ontology (30) or experimental factor ontology (31) (Table 3). If the annotation term is normalized, it stores ontology reference (term identifier and preferred level). The nodes are connected if they have a relation (dataset/sample, sample/term and term/term) (supplementary Figure S1). To allow for fast ontological search, all parents of a term in the ontology are also stored in the database and connected with their corresponding annotation terms (section ‘Semantic data layer’ and Supplementary Material). **User-DB** stores in-house sRNA-seq data (differential expression and classification from Oasis) uploaded by the users. This database allows users to compare their own data to the huge and diverse sRNA-seq published data. User uploaded data are deleted after 30 days.

**Table 3.** SEAweb keys and used ontologies (as of June 2019)

Key	Ontology(s)	# Annotations	# Terms
Organism	NCBI Taxonomy	4235	126
Tissue	BRENDA tissue/enzyme source	3021	190
Disease	Human Disease Ontology	1951	287
Cell type	Cell Ontology	732	304
Cell line	Cell Line Ontology	663	132
	Experimental Factor Ontology	134	76

In addition, SEAweb contains information about the GEO series accession (GSE) and sample accession (GSM) identifiers along with the sample identifier from the SRA database (SRR) in the Annotation-DB together with the annotations and in the Expression-DB together with expression profiles, differential expression and classification analysis. We optimize search and retrieval times by indexing for the most common queries and most relevant terms.

### Semantic data layer

Given the diversity of the biological data, users of the SEAweb system are given a possibility to interpret data independently using common terminologies. In order to enable users to browse data autonomously using common well-structured terminology, a standardized semantic layer for data retrieval is developed (Figure 1). It includes semantic annotations of data and semantic search, linking data with semantic lookup platform (OLS), as well as storing primary and derived data together with provenance information and references to secondary data.

One of the most important aspects of semantic layer are ontology-based data annotations. They enable interoperability of the data, as well as using of standard terminologies for data retrieval. It is important to standardize annotations using ontologies and semantic mappings (32). Ontologies define not only standard classes, but also the relations between terms, which enables semantic search by term hierarchies, for example, by parent terms. In SEAweb, we connect (normalize) annotations with ontologies in a semi-automatic way, i.e. first automatically extract possible annotation terms from GEO descriptions and normalize them, and later curate annotations manually (Supplementary Section 3). The Ontologies and the number of normalized terms in SEAweb are listed in Table 3. To enable the search across ontological hierarchies we integrated data with the relevant ontologies into the graph database Neo4J (supplementary Figure S1).

Ontology Lookup Service (OLS) is a service which allows to extract relevant terms from ontologies together with term information. SEAweb uses OLS for annotation normalization and accesses ontologies via the OLS REST interface, which supports complex and compound queries and query auto-completion (33). Details about annotation criteria, processing and group annotation are described in Supplementary Section 3.2.

Another aspect of the semantic layer is storing of the primary and the derived data together with provenance information. For SEAweb, primary data are FASTQ files, retrieved from the NCBI SRR database. This data are not stored after Oasis analysis, only provenance data about source and analysis details is saved. So for SEAweb, primary data are sRNA counts. Based on those counts, DE and classification results are obtained and are also saved to allow data interpretation. From derived data, the provenance information allows to retrieve raw counts and check how those results are obtained.

### Querying and visualization

Application programming interfaces (APIs) are developed to access data in SEAweb databases (Supplementary Section 3.5). The APIs help to use the multi-database system components independently as well as in combination. In brief, we extend the SEAweb backend application with RESTful web services, such as Annotation-API, Association-API, Expression-API, User-Expression-API, Predicted miRNA-API to access Annotation-DB, Association-DB, Expression-DB, User-Expression-DB and Oasis-DB, respectively. Additionally the SEAweb business logic API is created in order to combine all those APIs and make necessary data transformations between frontend and other APIs. As a result, the user can make queries to answer biological questions like; what is the expression of hsa-miR-488-5p across all human tissues? Is hsa-miR-488-5p expressed higher in adenocarcinomas as compared to other cancer types? Is a particular sRNA/pathogen DE in Alzheimer's disease? What are common DE sRNAs/pathogens or potential sRNA based biomarkers in a particular disease or tissue? What is the expression of a novel miRNA for known disease states? All API calls are described in Supplementary Section 3.5.

In addition, users can browse and query all datasets using the browse link. A three-panel browse function (Supplementary Figure S8) facilitates searching for specific small RNAs (miRNA, piRNA, snoRNA, snRNA and rRNA), annotation terms (organism, tissue, cell type, cell line and disease), and pathogens (bacteria or viruses). By selecting single or several terms from the three panel browse function the user can make arbitrarily specific searches in SEAweb. For example, the user can click on a small RNA and cancer to see its expression profiles in the cancer datasets.

In brief, the SEAweb system is developed using the modular system design approach (Figure 1). We build micro services to achieve strong encapsulation and well-defined interfaces via REST APIs. An object oriented programming approach is used to build the SEAweb application using the spring framework and Java 8. The SEAweb user interface (UI) is developed in Django framework version 2.0, HTML version 5, D3 and CSS 3. SEAweb visualizes the results depending on the user query, such as a violin plot for the expression of sRNAs or pathogens. Upset plots are shown for the overlap of sRNAs or pathogens (based on DE or classification) across experiments. SEAweb enables the download of search results in the form of CSV files. The functionality is tested on all major browsers (Table 4).

**Table 4.** SEAweb browser compatibility

Browser	Version
Chrome	61.0.3163.100, 62.0.3202.62
Mozilla Firefox	55.0.3, 56.0 (64-bit), 57.0 (64-bit)
Chromium	62.0.3202.75
Safari	11.0.1
Internet explorer	11

Browsers that are used to test SEAweb functionalities.

### SEAweb usage

SEAweb is a publicly available data repository and a web server and users can use it without an account or login. In case users want to upload and compare their own data to the data in SEAweb they need to create an account. Users have an option to sign in with their google account or they can register in the SEAweb system directly with a valid email address, choosing a username and password for their account. We have created User-DB to store their account information as well as sRNA-seq data uploaded by the users. Moreover, user-uploaded data are only accessible from the user's account. Users have the option to include their data in SEAweb for 30 days. For the data protection, security, and storage space reasons, we currently do not allow users to add data permanently to SEAweb (‘Materials and Methods’ section).

## APPLICATION OF SEA

In this section, we describe a few examples that illustrate how SEAweb can be employed to answer biological questions and to uncover unappreciated properties of sRNA data integration with interactive result visualization. First, we took advantage of the diverse and massive sRNA-seq data in SEAweb to present the most comprehensive set of tissue specific miRNAs till date. Second, we utilized the pathogenic reads in sRNA-seq to find their association to diseases. At last, we show a use case of SEAweb by comparing an in-house PD sRNA-ome to other neurodegenerative diseases sRNA expression profiles available in SEAweb.

### sRNA tissue specificity

**Figure 2.**
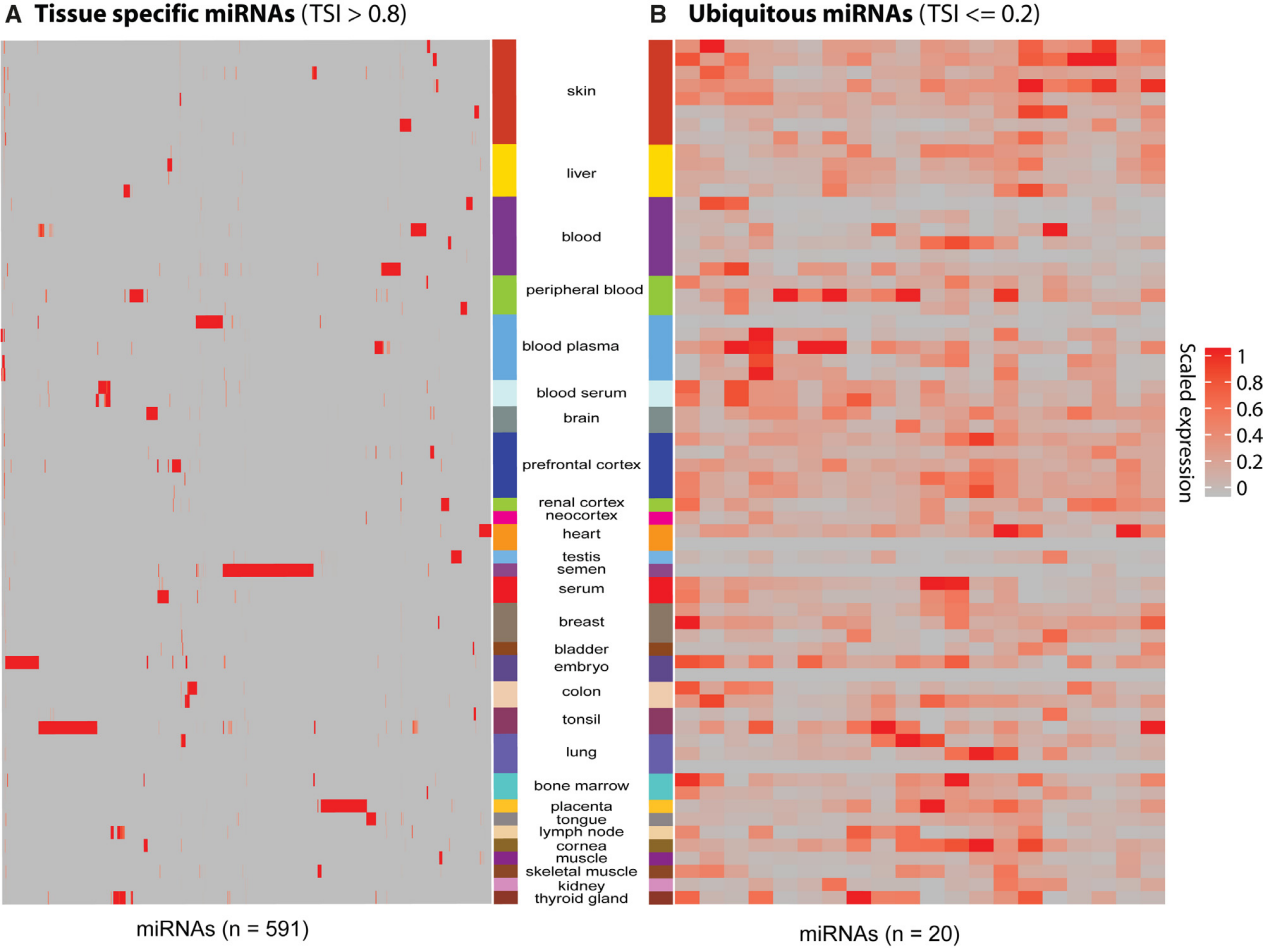
Tissue specific miRNAs. The heatmaps show the scaled expression (0-1) of (**A**) tissue specific or (**B**) ubiquitous miRNAs across all the tissues. (A) Tissue specific miRNAs. miRNA expression across all the tissues with TSI > 0.8 (*n* = 591). (B) Ubiquitous miRNAs. miRNA expression across all the tissues with TSI ≤ 0.2 (*n* = 20). miRNA names are omitted for simplicity. A complete list of tissue specific and ubiquitous miRNAs with their Shannon entropy score can be found in Supplementary Table S1. These calculations are based on healthy and disease samples within an experiment (‘Materials and Methods’ section).

Several studies have shown tissue specificity for miRNAs. Recently, Ludwig *et al.*, (34) analyzed several human tissue biopsies of different organs from two individuals to define the distribution of miRNAs using tissue specificity index (TSI) and found several groups of miRNAs with tissue-specific expression. Similarly, Lee *et al.*, (35) provides the expression of 201 miRNAs across nine human tissues to find tissue specificity of miRNAs. miRNAs whose expression is 20-fold or higher in a certain tissue compared with the mean of all the other tissues were characterized as tissue specific. According to Lee *et al.*, skeletal muscle, brain, heart and pancreas are the tissues expressing the most specific miRNAs. Moreover, Guo *et al.*, (36) manually extracted 116 tissue-specific miRNAs across 12 human tissues. We used Shannon entropy to calculate TSI for each human miRNA across all the human tissues available in SEAweb (‘Materials and Methods’ section). In order to calculate tissue specificity, we mixed healthy and diseased human samples (where available) within an experiment (Figure 2 and Supplementary Table S1). We used very stringent criteria: miRNAs with Shannon entropy score more than 0.8 were considered as tissue specific and ≤0.2 were considered as ubiquitous miRNAs (Figure 2 and Supplementary Table S1). We were able to provide by far the most comprehensive set of 591 distinct tissue-specific miRNAs across 29 tissues; blood plasma, skin, blood serum, liver, bone marrow, serum, testis, blood, semen, prefrontal cortex, peripheral blood, colon, brain, cornea, breast, renal cortex, bladder, embryo, placenta, lung, tongue, tonsil, skeletal muscle, kidney, lymph node, heart, muscle, thyroid gland and neocortex (Figure 2 and Supplementary Table S1). In order to compare the TSI for miRNAs in SEAweb with the existing findings, we merged the list of miRNAs from the above studies and retained all the 12 tissues. Out of 12 tissues, we did not have sequencing data for four of them: thymus, pancreas, spleen and bone.

We were able to detect two out of the three **heart** specific miRNAs (miR-1 and miR-302d) from Lee *et al.*, study, and 6 out of 10 heart specific miRNAs (hsa-miR-1-5p, hsa-miR-208a-3p, hsa-miR-208b-5p, hsa-miR-208b-3p, hsa-miR-302d-3p, hsa-miR-133b, hsa-miR-302a-3p, hsa-miR-302a-5p, hsa-miR-302b-3p) from the manually curated list of Guo *et al.* miR-208 is obtained from an old annotation, because the latest release of miRBase has more specific annotation like miR-208a-3p, miR-208b-3/5p. **Interestingly** we were able to find the whole family of miR-208 as heart specific. We were not able to detect miR-126, miR-302c, miR-367, hsa-miR-133a-5p in heart. Of note, none of these three is heart specific in the Lee *et al.*, study.

**Muscle** and **brain** were the only two tissues covered by all the three above mentioned studies. In muscle, we were able to detect two out of the three muscle specific miRNAs (miR-133b, miR-1-3p) from Ludwig *et al.*, three out of four (miR-95 was not found to be muscle specific) from Lee *et al.*, and 4 out of 10 for Guo *et al.*, compilation. We were not able to detect miR-206, miR-133a, miR-134, miR-193a, miR-95 and miR-128a. Note that from the same study miR-134 is mentioned as muscle as well as testis specific and miR-128a as muscle as well as brain specific. Moreover miR-95 is the only miRNA that is muscle specific in all of the three studies.

Another tissue covered by all of the three studies is the brain. In total 30 miRNAs were known to be brain specific, only 1 out of 30 (miR-7) is common among all the three studies and only three in two studies (miR-124, miR-9, miR-218) one of which is in the curated list. In our study, we found 26 miRNAs to be brain specific but none from the known ones.

Tissue with the most number (*n* = 43) of known specific miRNA was **placenta** provided by Guo *et al.* Interestingly, miRNAs associated with placenta were mostly evolutionary related. We were able to detect these evolutionary related miRNAs to be placenta specific as well. In short, we detected **517**a/b/c, **518**a/b/c/d/e/f, **519**a/b/c/d/e, **520**a/d/e/f/g (not detecting **520**b/c/h). Moreover we were also able to detect miR-371, miR-372, miR-512, miR-522, miR-523, miR-524, miR-525, miR-526b and miR-527. Out of 43, we detected 35 and did not detected miR-377, miR-526a, miR-184, miR-154, miR-381, miR-503, miR-450 and miR-136. We detected only 2 (miR-513c-5p, miR-202-3p) out of 15 for **testes**. There were two tissues, **lung** and **liver**; mentioned only in one study Guo *et al.*, we could not detect the only miRNA miR-126 for lung. Interestingly this miRNA is also mentioned as heart specific in the same study. We also did not find the four liver specific miRNAs miR-122, miR-483, miR-92a, miR-192; two (miR-483, miR-92a) of which are shown as bone specific in the same study. In **kidney** we could not detect any miRNA out of eight kidney specific in Guo *et al.* Of note Lee *et al.*, also found only one miRNA miR-204 to be kidney specific and does not have any evidence for the rest of the seven miRNAs. In brief, there is no significant consensus on the tissue specific miRNAs in the previous studies. However, our work still aligns reasonably well to their findings.

To understand if disease samples might affect the tissue specificity calculations we also performed a tissue specificity analysis using only non-disease samples (Supplementary Figure S2, 4, Table S1 and 4). Using only non-disease samples we found three additional tissue specific miRNAs, hsa-miR-503-3p in placenta, hsa-miR-1-3p and hsa-miR-133a-5p in muscle and heart. Overall, our miRNA tissue predictions, mixed as well as non-disease only, were consistent with published information on tissue-specific miRNA expression (34–36). As Ludwig *et al.*, used only two individual's tissues, Lee *et al.*, also performed own experiments in a control (same laboratory, same protocols) environment and used different statistical methods compared to ours, we were still able to get a reasonable overlap with tissue-specific miRNAs considering diverse (different laboratories, different protocols) and massive data. Therefore, we think that this work provides the most comprehensive set of tissue-specific miRNAs till date (*n* = 591 miRNAs) (Supplementary Table S1). In order to explore the tissue specificity of other types of sRNAs in SEAweb such as piRNA, snoRNA, snRNA and rRNA, we repeated the above analysis with exactly the same set of samples once for the healthy and diseased mixed and once for the non-diseased samples (Supplementary Figures S3, 4 and Table S4). We found 3445 out of 4300 (filtered for minimum reads, see ‘Materials and Methods’ section) sRNAs to be tissue specific and only 73 to be ubiquitous in the healthy and disease mixed samples (Supplementary Figure 3 and Table S4). In the non-diseased samples, we found 1005 sRNAs to be specific and 45 to be ubiquitously expressed across tissues (Supplementary Figure S3 and Table S4).

### Known and novel bacterial or viral infections

**Figure 3.**
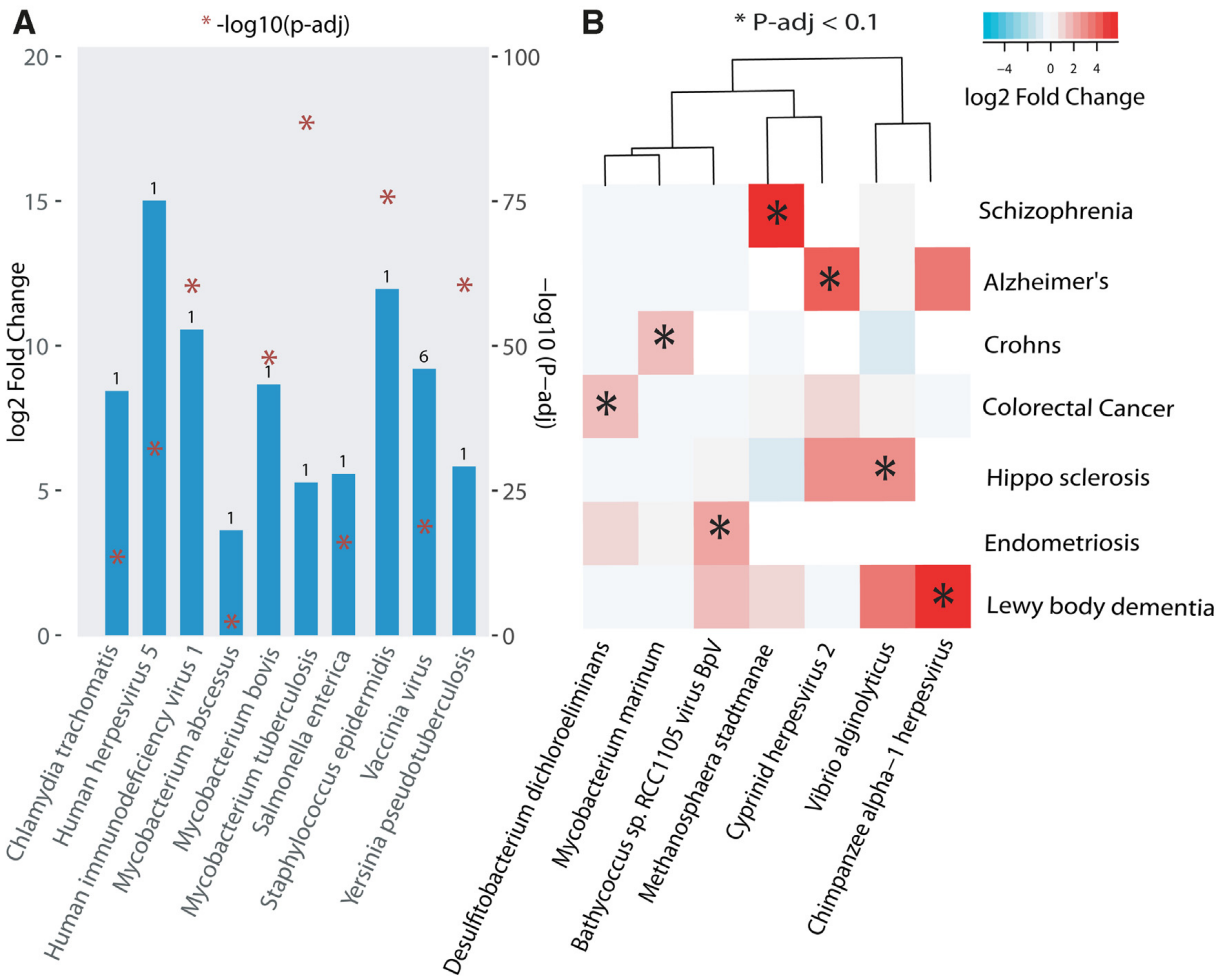
Known and Novel bacterial or viral infections. (**A**) Known associations. Pathogen detection using seven datasets known to be infected with seven bacterial and three viral pathogens. Bar represents pathogen log_2_-fold difference between the uninfected and infected state (Supplementary Table S2). Number on top of the bar denotes rank of the pathogen compared to all the other DE pathogens within the comparison (i.e. smallest adjusted *P*-value). (**B**) Novel associations. Heatmap shows log_2_-fold difference of pathogens significantly upregulated in disease as compared to healthy (fold change > 1 and padj < 0.1) (Supplementary Table S2). Comparisons that have less than six pathogens significantly DE are selected for specificity. Details about dataset, comparison groups, log_2_fold and padj for both (A and B) are provided in (Supplementary Table S2).

We have validated our approach of pathogen detection in Oasis 2 (16) using sRNA datasets with defined viral or bacterial infections. Overall, the prediction of bacterial (*Mycobacteriumabscessus*) and viral (HIV, HHV4, HHV5, Gallid_herpesvirus_2) infections resulted in high F-scores, recall and precision, especially when the top five predicted pathogen species are reported. However, the current work additionally involves differential expression analysis of pathogens and therefore we validated our approach of pathogen differential regulation using seven datasets with known infection status. The samples in these datasets are known to be infected with seven bacterial pathogens and three viral pathogens. Of note, we focused on within-dataset comparison in order to avoid technical confounders (Supplementary Table S2). For each sample, k-mer counts were calculated for all infectious species present in Kraken database (4336 viral and 2784 bacterial/archaeal genomes) and differential abundance analysis was carried out for those species that have at least three counts (baseMean) in a particular comparison. As expected, in all comparisons the known pathogen represented the best hit (i.e. smallest adjusted *P*-value) except Vaccinia virus (Figure 3A). However, Vaccinia virus has the highest log_2_ fold change as expected within the dataset (GSE54235) comparison. It is worthy to note that Chlamydia trachomatis detection is based on sRNA-seq performed on conjunctival tissue from children with follicular trachoma and children with healthy conjunctivae, indicating a good performance of our pathogen detection pipeline from tissues.

Next, we aimed to find novel associations of pathogens with disease. We took all the comparisons, which has ‘healthy’ and at least a disease state annotation (Supplementary Table S2). In order to achieve more specificity we took only comparisons that have less than six pathogens significantly upregulated in disease as compared to healthy (FC > 1 and padj < 0.1). There were a total of eight comparisons but we removed ‘GSE69837’ as this was a known case (Chlamydia trachomatis already shown in Figure 3A). It was interesting to find viruses and bacteria significantly upregulated in sRNA-seq data in certain disease compared to healthy patients (Figure 3B). Some of the most interesting cases are highlighted in this section below.

#### Mycobacterium marinum in patients with ileal Crohn's disease

In the original study, expression of microRNAs in mucosae of patients with a normal pouch after colectomy for intractable ulcerative colitis was compared to several control cohorts, among them was a cohort of patients with Crohn's disease (CD) of the terminal ileum (37). CD patients were previously not exposed to immunosuppression. Compared to patients with non-inflamed ileal pouch, patients with ileal CD showed an increased mucosal expression of *Mycobacterium marinum*. The bacterial genus *Mycobacterium* causes diverse diseases in humans, of which Tuberculosis is the most serious with around one-quarter of the world population latently infected and ∼1.6 million deaths in 2017 on a global scale. *M. marinum* is a non-tuberculous (also termed ‘atypical’) *Mycobacterium* species, which is ubiquitously abundant in aquatic environments (38). Infection of humans is well known, but it is considered a rare event. It typically occurs after exposure to contaminated water or infected marine animals, and it is more common in immunosuppressed individuals. The most commonly affected organ is the skin, in more severe cases involvement of muscles, bones or joints is reported (38). Opportunistic infection with *M. marinum* in CD is recognized in those patients receiving anti-tumor necrosis factor therapy (e.g. infliximab) (39). However, to the best of our knowledge, enteric super-infection with *M. marinum* has not been reported in the literature so far. Interestingly, due to the resemblance of the granulomatous intestinal inflammation in CD with enteric infection caused by other Mycobacteria, it has been hypothesized that Mycobacterial infection is involved in the pathogenesis of CD, with much focus on *Mycobacterium avium paratuberculosis* (40). However, the aetiological significance of this pathogen in CD remains uncertain. Hence, the gut mucosal prevalence of *M. marinum* and its potential pathophysiologic significance in patients with CD should be further explored.

#### Methanosphaera stadtmanae in patients with schizophrenia

We detected an overabundance of *Methanosphaera stadtmanae* in neurons derived from induced pluripotent stem cells (iPSC) of patients with schizophrenia, compared to healthy controls. *M. stadtmanae* is an Archaeal microorganism which is frequently detected in the healthy human gut microbiota (41). It is involved in intestinal methanogenesis and associated fermentative dynamics. *M. stadtmanae* is recognized by the innate immune system, therefore it can induce inflammatory cytokine responses and could have diverse immunomodulatory functions (42). Interestingly, *M. stadtmanae* was found with an increased prevalence in faecal samples of patients with inflammatory bowel diseases (IBD) Crohn's disease (CD) and ulcerative colitis with antigen-specific IgG-responses (43). Immune system processes have been proposed to be involved in the pathogenesis of schizophrenia (44). Regarding the immune-genetic basis of schizophrenia, genome-wide pleiotropy has been reported between schizophrenia and CD as well as an increased prevalence of schizophrenia in patients with IBD (45). Therefore, the potential immunogenic importance of *M. stadtmanae* in schizophrenia should be investigated.

#### Chimpanzee herpesvirus in Lewy body dementia

We detected an increased abundance of a viral pathogen identified as *chimpanzee herpesvirus* (ChHV) in the cerebral cortex of patients with lewy body dementia (LBD) compared to non-demented controls (46). ChHV is an alphaherpesvirus closely related to human herpes simplex virus type 2 (HSV-2) (47). LBD is a neurodegenerative disorder, which underlies 4.2% of all dementia cases, second only to Alzheimer's dementia (AD) (48). The aetiology of LBD is obscure, but growing evidence points toward neuro inflammation as a key pathophysiologic factor, analogous to the pathogenesis of AD (49). In AD it is assumed that multiple pathogens infecting the brain are key triggers of neural dysfunctional protein accumulation and neuro inflammation in genetically vulnerable individuals (50). Among the pathogens detected in brains of AD patients, multiple lines of evidence point at herpes simplex virus type 1 (HSV-1) and HSV-2 as two of the main drivers of AD neurodegeneration (50,51). Given the close phylogenetic relationship between ChHV and HSV-2, ChHV might play a role in inflammatory neurodegenerative processes in LBD similar to the other herpesviruses in AD. Therefore, the association detected in the present study should be further elaborated.

### Analyzing in-house data and comparing with SEAweb data

One of the key features available in SEAweb is uploading the in-house data and comparing it with the already integrated data. Mostly, researchers use different analysis pipelines to carry out differential expression or classification, which makes it very hard to compare the results with the publicly available data. Therefore, we require a database with interactive visualizations that has all the publicly available data analyzed using the same pipeline with same parameters. For SEAweb, we have analyzed and integrated all the data using Oasis 2 pipeline. We expect that comparing the in-house data with the data in SEAweb will yield disease-specific signatures, in this case a sRNA or group of sRNAs. Note that uploading to SEAweb requires the output of Oasis 2 (Supplementary Material).

In order to test this feature, we uploaded in-house sRNA-seq data from well characterized 47 PD and 53 frequency-matched healthy controls, which is a baseline data from the longitudinal *denovo* Parkinson disease (DeNoPa) cohort (Supplementary Table S3) and available as ‘demo user data’ in SEAweb. SEAweb gives us a unique opportunity to identify PD-specific biomarkers associated with early-stage PD that can eventually help us in early diagnosis, therefore, better treatment of the disease. Below we describe the differential expression and classification results from PD data and an approach in order to identify PD-specific biomarkers that do not overlap with other neurodegenerative diseases.

**Figure 4.**
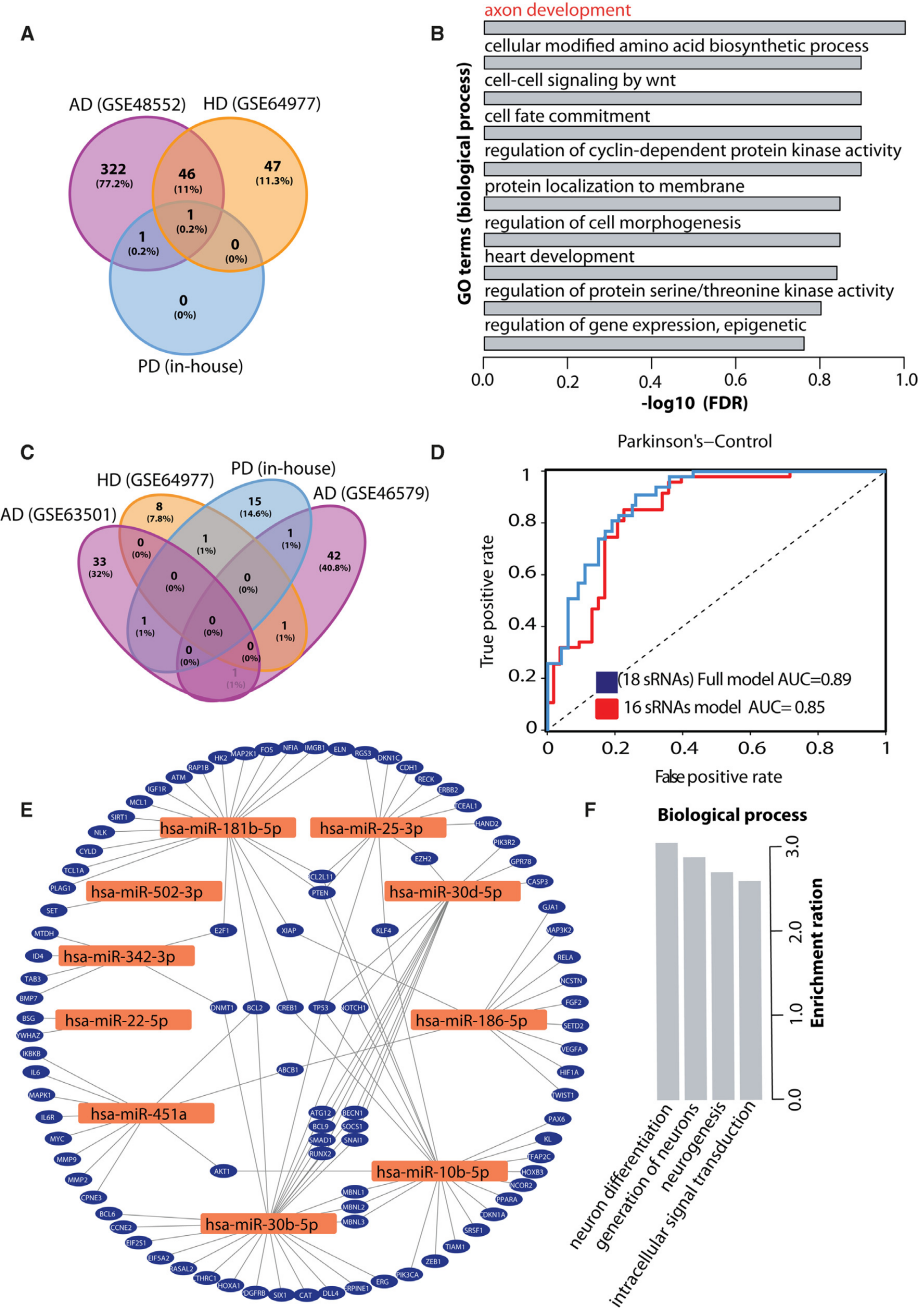
In-house *denovo* Parkinson disease (DeNoPa). (**A**) sRNA DE Overlap. Overlap of upregulated sRNAs between in-house denopa (blue), AD (purple) and HD (orange). Overall nine neurodegenerative disease comparisons were considered and overlap was found with these two datasets. (**B**) GO terms. Top 10 GO terms associated with the target genes of the two downregulated sRNAs. (**C**) sRNA classification Overlap. Overlap of classification features (sRNAs) between in-house denopa (blue), AD (two datasets) (purple) and HD (orange). (**D**) DeNoPa classification. Receiver‐operating characteristic (ROC) curve showing true‐ and false‐positive rates for DeNoPa disease prediction based on sRNA expression profile using 18 sRNAs in full model (blue) and 16 unique (not found in other neurodegenerative diseases) sRNAs (red). (**E**) PD associated genes. Network of PD associated genes and 13 known miRNAs from the classification. (**F**) GO terms for novel miRNAs. GO terms associated with the target genes of the three novel miRNAs from the classification.

We found four significantly DE miRNAs with adjusted *P*-value < 0.1. Out of these, two are upregulated in PD (hsa-miR-502-3p and hsa-miR-532-5p) and two are downregulated in PD (hsa-miR-30d-5p and hsa-miR-22-5p) (Supplementary Table S3). Next, we overlapped these four DE miRNAs with all the neurodegenerative disease-related datasets integrated in SEAweb. We focused on nine comparisons (from five datasets) in which one of the conditions is a healthy state and the other is a diseased condition (Alzheimer's disease (AD), LBD, tangle-predominant dementia, Huntington's disease (HD), Frontotemporal dementia or Hippocampal sclerosis of aging). Out of the two upregulated miRNAs in PD, one (hsa-miR-502-3p) is upregulated in Alzheimer's disease and one (hsa-miR-532-5p) is upregulated in both Alzheimer's and Huntington's disease (Figure 4A). In contrast, none of the downregulated miRNAs in PD were found to be significantly down in any of these nine comparisons. Interestingly, it has been shown that the expression of miR-22 is downregulated in a 6-hydroxydopamine-induced cell model of PD using RT-PCR (52). Moreover, Margis *et al.*, found that hsa-miR-22 has reduced expression in the blood of *denovo* PD patients (53). Furthermore, family members of hsa-miR-30d-5p are known to be deregulated in PD (54) and putatively target the PD-related gene, **LRRK2 (PARK8)** (55). These results confirms, the potential role of hsa-miR-30d-5p and hsa-miR-22-5p in PD. To explore the mechanism by which these two miRNA are involved in PD, we performed gene ontology (GO) analysis of the validated and predicted targets using webgestalt (56). The top ten terms ranked according to FDR adjusted *P*-value are shown in the (Figure 4B). The top significant hit (FDR < 0.1) is axon development. Recent publications (57–59) have suggested the role of massive and unmyelinated axonal arbor in PD. In substantia nigra pars compacta (SNc), the axonal arbor of dopamine neurons is very large as compared to other neuronal types. This leads to the hypothesis that these dopamine neurons have selective and exceptional vulnerability in PD, and have a higher energy demand that may play a crucial role in cell death (57).

To obtain a unique PD biomarker we explored the classification results integrated in SEAweb. PD and healthy were classified with an AUC of 0.89 (Figure 4D). Interestingly, the classifier used only 18 sRNAs (‘Materials and Methods’ section) to separate the two states (Supplementary Table S3 and Figure S5). Moreover, only two sRNAs hsa-miR-30d-5p (downregulated in PD) and hsa-miR-502-3p (upregulated in PD) are DE between healthy and PD out of the 18 sRNAs identified by the classifier (Supplementary Figure S6). We overlapped these 18 sRNAs with the classification results from other neurodegenerative diseases integrated in SEAweb (Figure 4C). There are only three sRNA that are also found in AD or HD but they have opposite change of expression. This suggests the specificity of these sRNAs to PD as compared to other neurodegenerative diseases. Furthermore, to filter out sRNAs known to be associated with other neurodegenerative diseases, we used the association database of sRNA-disease association available in SEAweb. The results showed that hsa-miR-342-3p has been associated with other neurodegenerative diseases (60,61). Next, we also filtered out sRNAs if the base mean read count is less than five and also, hsa-miR-502-3p that was found to be upregulated in AD (Figure 4A). Then we run a random forest classifier using the normalized counts for the remaining 15 sRNAs and hsa-miR-22-5p that is downregulated in our data. (Figure 4D) shows that using 16 sRNAs to classify PD and controls, yielded 85% area under the curve (AUC) with 83% recall and 77% of precision. Furthermore, to find the relevance of the 13 known miRNAs (out of 16 sRNAs) in PD, we obtained their target genes from SEAweb (only 10 miRNAs out of 13 have targets supported by strong evidence) and overlapped with the targets genes of PD associated miRNAs in SEAweb. Interestingly, these 10 known miRNAs targets 96 genes, which are known to be associated with PD (Figure 4E and Supplementary Table S3). The list includes **TP53** (62) that contributes to the apoptotic deterioration taking place in PD, **PTEN** (63) that has been linked to PD via DNA damage and DNA repair machinery, **SMAD1** (64) is an important regulator required for neurite growth, **EZH2** (65) is a lysine methyltransferase component of polycomb repressive complex 2 that has been associated with PD and **BCL2** (66) is required for proper development of the dopaminergic system and has been implicated in the pathogenesis of PD. To gain further insights into the three novel predicted miRNAs (out of 16 sRNAs) used to classify PD and controls, we performed gene enrichment analysis on their target genes using webgestalt (67). The novel miRNAs were p-hsa-miR-113, p-hsa-miR-247 and p-hsa-miR-235-**1/2/3** (Supplementary Material). We used miRDB (17) to get target genes for the mature sequences of these predicted miRNAs (‘Materials and Methods’ section). Interestingly the GO terms for these miRNAs were **neuron differentiation, generation of neurons**, **neurogenesis** and **regulation of intracellular signal transduction** (Figure 4F). All these processes are highly related to PD, and hence we think these novel miRNAs should further be explored and validated in the laboratory. Predicted structure of these miRNAs can be found in Supplementary Material.

All together, these results make a strong case in favor of using SEAweb in order to retrieve disease-specific biomarkers.

## CONCLUSION

SEAweb is designed for the biological or medical end-user that is interested to define where and when a sRNA of interest is expressed. Prototypical questions that can be addressed with SEAweb are: What is the expression of hsa-miR-488-5p across all human tissues? Is hsa-miR-488-5p expressed higher in adenocarcinomas as compared to other cancer types? Is the tissue-specific expression of hsa-miR-488-5p conserved in mice? Its unique selling points are the deep and standardized annotation of meta-information, the re-analysis of published data with Oasis 2 to reduce analysis bias, a user-friendly search interface that supports complex queries and the fast and interactive visualization of analysis results across 10 organisms (Table 1) and various sRNA-species. SEAweb also contains information on the expression of currently 769 high-quality predicted miRNAs, across organisms and tissues.

In addition, SEAweb also stores sRNA differential expression, sRNA based classification, pathogenic sRNA signatures from bacteria and viruses and pathogen differential expression. Furthermore, SEAweb can be used to search gene targets or diseases associated with a miRNA. Moreover, SEAweb allows end users to upload their analysis results of differential expression and classification from Oasis 2. This will allow users to compare their data to over 4200 experimental samples across different conditions. SEAweb also provides users with an option to perform on the fly analysis such as overlapping DE sRNAs or pathogens across different studies or the most important features (sRNAs) identified with classification. SEAweb enables end users to re-submit samples from interactive plots for differential expression or classification, this will help users to choose samples of their choice from an experiment (Supplementary Figure S7).

Moreover, SEAweb is continuously growing and aims to eventually encompass all sRNA-seq datasets across all organisms deposited in GEO and other repositories. In order to keep SEAweb up to-date with the current small RNA sequencing data or the data that will be published to GEO in the future, we have written programs that automatically search GEO and SRA databases every two weeks (consistent with the GEO update cycle). These programs download raw fastq files, submit these to Oasis 2, and assign responsibility to another program for the semi-automated annotation for tissue, cell line, cell type and other meta-data available. In case the system cannot fully annotate all fields, automatic annotation is followed by manual curation using a front-end curation system. Currently manual annotation QA is the rate-limiting step, which is why we actively develop deep learning-based annotation prediction routines for future versions of SEAweb (68). Genome versions will be updated with every major release of SEAweb. SEAweb will be backward compatible in the future by allowing users to choose previous genome versions and annotations.

A detailed comparison of SEAweb to other existing sRNA expression databases highlights that SEAweb is superior in terms of supported organism, annotations, diseases, tissues, sRNA based classification, pathogen k-mer DE, known miRNA disease associations, user specific experimental data upload, cross study comparisons and re-analysis with selected samples (Table 2).

As far as we are aware, SEAweb is the only sRNA-seq database that supports ontology-based queries, supporting single or combined searches for five predefined keys (organism, tissue, disease, cell type and cell line) across all datasets. However, the SEAweb database system contains additional (meta)-information including age, gender, developmental stage, genotype as well as technical experimental details such as the sequencing instrument and protocol details (e.g. library kit, RNA extraction procedure). We plan to normalize most of this additional information in future versions of SEAweb. This will allow users, for example, to query and analyze sRNA expression effects that are introduced by library kit or sequencing platform differences (both of these features can introduce large biases in the detection and expression of sRNAs). Other future developments will include information on sRNA editing, modifications and mutation events.

In summary, SEAweb supports interactive result visualization on all levels, from querying and displaying of sRNA expression information to the mapping and quality information for each of the over 4200 samples. SEAweb is a fast, flexible, and fully interactive web application for the investigation of sRNA and pathogen expression across cell lines, tissues, diseases, organisms and sRNA-species. As such, SEAweb should be a valuable addition to the landscape of sRNA expression databases.

Additionally, we presented the most comprehensive set of tissue specific miRNAs till date. We were able to provide by far the most complete set of 591 distinct tissue specific miRNAs across 30 tissues. To our knowledge this is by far the most comprehensive analysis (set) of tissue-specific miRNAs.

In the current work, we also found pathogen signatures from sRNA-seq data. We found signatures of pathogens in severe diseases like dementia. In brief, we found differential regulation of *M*. *marinum* in patients with ileal crohn's disease, methanosphaera stadtmanae in patients with schizophrenia and chimpanzee herpesvirus in LBD.

From our in-house PD data, we were able to find potential biomarkers based on differential expression and classification for the early detection of PD. The top term for the GO analysis of the two downregulated miRNAs is axon development, suggesting their role in PD. Moreover, gene targets of the sRNAs for the top important features (potential biomarkers) for PD using classification were overlapping with the targets of the known PD miRNAs. Additionally, GO analysis for the targets of the three novel miRNAs are neuron differentiation, generation of neurons, neurogenesis and regulation of intracellular signal transduction (Figure 4F). We think these novel miRNAs should be further explored and validated in the laboratory.

At last, researchers have used massive sRNA data from SEAweb for other tasks, for example, it enables to use deep learning for data augmentation problem such as predicting sex and tissue based on sRNA expression profiles (68). As such, SEAweb should be a valuable addition to the landscape of sRNA-seq web applications.

## DATA AVAILABILITY

SEAweb is implemented in Java, J2EE, spring, Django, html5, css3, JavaScript, Bootstrap, Vue.js, D3, mongodb and neo4j. It is freely available at http://sea.ims.bio/.

## Supplementary Material

gkz869_Supplemental_Files
