# Supplementary material for: SEAweb: the small RNA Expression Atlas web application
Source: Nucleic Acids Res. 2019 Oct 10;48(D1):D204–19. doi: 10.1093/nar/gkz869 (PMC6943056; doi:10.1093/nar/gkz869)
Supplement: gkz869_Supplemental_Files [file gkz869_supplemental_files.zip › p-hsa-miR-113.pdf]

Provisional ID	:	chr2_1671
Score total	:	526.3
Score for star read(s)	:	3.9
Score for read counts	:	520.1
Score for mfe	:	1.3
Score for randfold	:	1.6
Score for cons. seed	:	-0.6
Total read count	:	1032
Mature read count	:	1012
Loop read count	:	1
Star read count	:	19

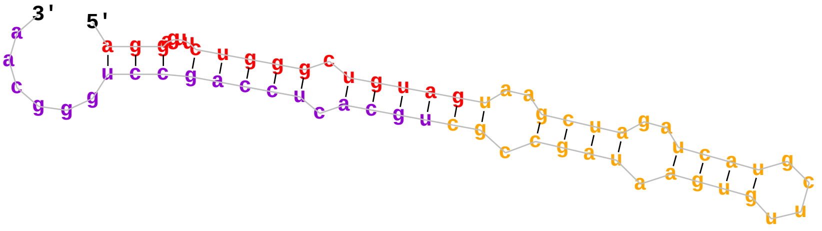

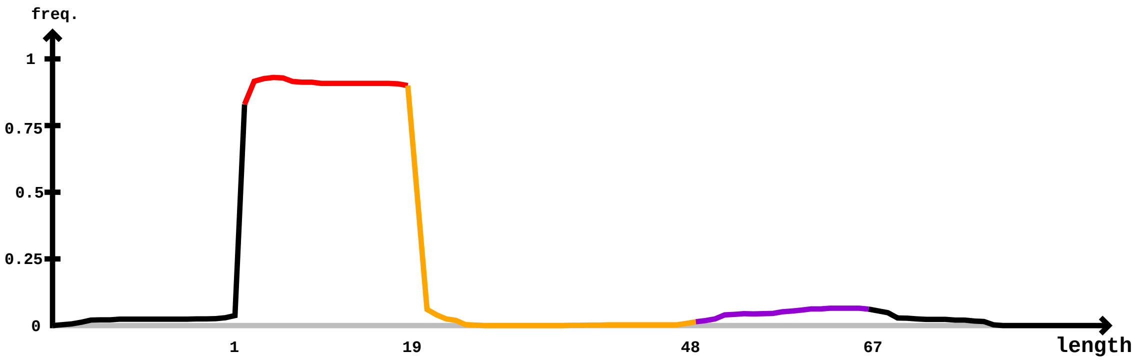

Mature Star

```
5'- ggaggauugcuugaaccuaggaguucugggcuguaguaagcuagaucaugcuugugaauagccgcugcacuccagccugggcaacagaaggagacuaugucucuaaaa   -3'   obs
    ggaggauugcuugaaccuaggaguucugggcuguaguaagcuagaucaugcuugugaauagccgcugcacuccagccugggcaacagaaggagacuaugucucuaaaa         exp
    ......(((.(((..((((((....(((((.((((((..((((..((((....)))).)))).)))))).)))))))))))))))))..((((((...)))))).... reads  mm  sample
    ggagAauugcuugaaccu.......................................................................................... 2      1   aou
    ggagAauugcuugaaccuaggag..................................................................................... 2      1   aou
    ggaggauugcuugaaccuaggagAuc.................................................................................. 3      1   aou
    .gaggauugcuugaUccuagga...................................................................................... 1      1   aou
    .gagAauugcuugaaccuaggag..................................................................................... 1      1   aou
    .gaggauugcuugaaccuaggagu.................................................................................... 3      0   aou
    .gaggauugcuugaaccuaggagAuc.................................................................................. 2      1   aou
    ..aggauugcuugaaccuagga...................................................................................... 1      0   aou
    ..agAauugcuugaaccuaggag..................................................................................... 5      1   aou
    ..aggauugcuugaaccuaggag..................................................................................... 3      0   aou
    ...gAauugcuugaaccuaggag..................................................................................... 1      1   aou
    .....auugcuugaaccuGggag..................................................................................... 3      1   aou
    .............aGccuaggaguucugggcugu.......................................................................... 1      1   aou
    ...............ccCaggaguucugggcuguaguaa..................................................................... 1      1   aou
    ................cNaggaguucugggcugu.......................................................................... 1      1   aou
    ................cNaggaguucugggcugua......................................................................... 1      1   aou
    ................cAaggaguucugggcuguag........................................................................ 1      1   aou
    ................cCaggaguucugggcuguaguaagc................................................................... 1      1   aou
    .................uaggaguucugggcugua......................................................................... 5      0   aou
    .................Gaggaguucugggcugua......................................................................... 1      1   aou
    .................uaggaguucugggcuguag........................................................................ 1      0   aou
    .................uaggaguucugggcugGag........................................................................ 1      1   aou
    .................Caggaguucugggcuguaguaa..................................................................... 1      1   aou
    ..................aggaguucugggcuguag........................................................................ 507    0   aou
    ..................aggaguucugggcuUuag........................................................................ 4      1   aou
    ..................aggNguucugggcuguag........................................................................ 1      1   aou
    ..................aggaguucugggGuguag........................................................................ 1      1   aou
    ..................agNaguucugggcuguag........................................................................ 1      1   aou
    ..................aggaguucugggcuguCg........................................................................ 1      1   aou
    ..................aggaguucugggcuguaC........................................................................ 1      1   aou
    ..................aggaguucugggUuguag........................................................................ 7      1   aou
    ..................aggaguucugggcugGag........................................................................ 1      1   aou
    ..................aggCguucugggcuguag........................................................................ 1      1   aou
```

Mature **Star**

```
ggaggauugcuugaaccuaggaguucugggcuguaguaagcuagaucaugcuugugaauagccgcugcacuccagccugggcaacagaaggagacuaugucucuaaaa
..................aggaguucugCgcuguag........................................................................ 4 1 aou
..................aggaguucugggcuAuag........................................................................ 1 1 aou
..................aggaguucuUggcuguag........................................................................ 6 1 aou
..................aggaguucuggUcuguag........................................................................ 10 1 aou
..................aggaguucugUgcuguag........................................................................ 9 1 aou
..................aggaguucuggCcuguag........................................................................ 1 1 aou
..................aggaguucugggcuguGg........................................................................ 2 1 aou
..................aggGguucugggcuguag........................................................................ 2 1 aou
..................aggaguucugggcuguaA........................................................................ 2 1 aou
..................aggaguucuggAcuguag........................................................................ 1 1 aou
..................aggaguucugggAuguag........................................................................ 1 1 aou
..................Nggaguucugggcuguag........................................................................ 1 1 aou
..................aggaguucugggcugAag........................................................................ 2 1 aou
..................aggaguuAugggcuguag........................................................................ 3 1 aou
..................aggagCucugggcuguag........................................................................ 3 1 aou
..................aNgaguucugggcuguag........................................................................ 1 1 aou
..................aggaguuAugggcuguagu....................................................................... 2 1 aou
..................aggaguucugUgcuguagu....................................................................... 10 1 aou
..................aggaguucugggcuguNgu....................................................................... 1 1 aou
..................aggaguucugggcuguaAu....................................................................... 3 1 aou
..................aggCguucugggcuguagu....................................................................... 2 1 aou
..................Nggaguucugggcuguagu....................................................................... 1 1 aou
..................aggaguucugggUuguagu....................................................................... 5 1 aou
..................aggaguucugggcugGagu....................................................................... 3 1 aou
..................aggaguucugCgcuguagu....................................................................... 3 1 aou
..................aggaguucugggcuguCgu....................................................................... 1 1 aou
..................aggaAuucugggcuguagu....................................................................... 1 1 aou
..................aggagCucugggcuguagu....................................................................... 1 1 aou
..................aggaguucugggcuguagC....................................................................... 3 1 aou
..................aggaguucuggCcuguagu....................................................................... 4 1 aou
..................agCaguucugggcuguagu....................................................................... 1 1 aou
..................aggaguucuggUcuguagu....................................................................... 13 1 aou
..................aggaguucugggcuguagu....................................................................... 218 0 aou
..................aggGguucugggcuguagu....................................................................... 1 1 aou
..................aggaguucugggcuNuagu....................................................................... 1 1 aou
..................Cggaguucugggcuguagu....................................................................... 1 1 aou
..................agUaguucugggcuguagu....................................................................... 1 1 aou
..................agAaguucugggcuguagu....................................................................... 2 1 aou
..................aggaguucugggcugCagu....................................................................... 3 1 aou
..................aggaguucugggcuUuagu....................................................................... 4 1 aou
..................aggaguCcugggcuguagu....................................................................... 1 1 aou
..................aggaguucugAgcuguagu....................................................................... 1 1 aou
..................aggaguucugggcuguagua...................................................................... 3 0 aou
..................aggaguucugggcuguaguC...................................................................... 11 1 aou
..................aggaguucugggcuguaAua...................................................................... 2 1 aou
..................aggaguucugggcuguaguU...................................................................... 1 1 aou
..................aggaguucugggcuguaguGa..................................................................... 9 1 aou
..................aggaguucugggcuguaguaaC.................................................................... 1 1 aou
..................aggaguucugggcuguaguaaAc................................................................... 1 1 aou
..................aggaguucugggcuguaguaaUc................................................................... 1 1 aou
...................gUaguucugggcuguagu....................................................................... 1 1 aou
...................ggaguCcugggcuguagu....................................................................... 1 1 aou
...................ggCguucugggcuguagu....................................................................... 2 1 aou
...................ggaguucugggcuguaCu....................................................................... 1 1 aou
...................ggaguucugggcuguagu....................................................................... 70 0 aou
...................ggaguucugggAuguagu....................................................................... 1 1 aou
...................Ugaguucugggcuguagu....................................................................... 1 1 aou
...................ggaguucugggcuguaguC...................................................................... 1 1 aou
...................ggaguucugggcuguaguU...................................................................... 1 1 aou
...................ggaguucugggcuguagua...................................................................... 2 0 aou
...................ggaguucugggcuguaAua...................................................................... 1 1 aou
...................ggaguucugggcuguaguGa..................................................................... 5 1 aou
...................ggaguucugggcuguaguaaC.................................................................... 4 1 aou
...................ggaguucugggcuguaguCagc................................................................... 1 1 aou
...................ggaguucugggcuguaguUagc................................................................... 2 1 aou
...................ggaguucugggcuguaguGagc................................................................... 1 1 aou
...................ggaguucuAggcuguaguaagc................................................................... 1 1 aou
...................ggaguucugggcuguaguGagcu.................................................................. 1 1 aou
....................gaguucugggcuguaguC...................................................................... 1 1 aou
....................gaguucugggcuguaguCa..................................................................... 1 1 aou
```

```
                  Mature                                               Star
ggaggauugcuugaaccuaggaguucugggcuguaguaagcuagaucaugcuugugaauagccgcugcacuccagccugggcaacagaaggagacuaugucucuaaaa
....................gaguucugggcuguaguGag....................................................................  1    1    aou
....................gaguucugggcuguaguaaUc...................................................................  1    1    aou
....................gaguucugggcuguaguaaAc...................................................................  1    1    aou
....................gaguucugggcuguaguaagc...................................................................  3    0    aou
....................gaguucugggcuguaguUagc...................................................................  1    1    aou
....................gaguucugggcuguaguaCgcua.................................................................  2    1    aou
.....................aguucugggcuguaguaaAc...................................................................  2    1    aou
.....................aguucugggcuguaguGagc...................................................................  2    1    aou
.....................aguucugggcuguaguGagcu..................................................................  1    1    aou
....................................................ugugaauagcGgcugcacu.....................................  1    1    aou
......................................................ugaauagcCcugcacucca...................................  1    1    aou
........................................................aauagccCcugcacuccag.................................  1    1    aou
................................................................cugcacuccagccCgggca.........................  1    1    aou
................................................................cugcacCccagccugggca.........................  1    1    aou
................................................................cugcacuccaUccugggca.........................  1    1    aou
................................................................cugcacucNagccugggcaa........................  1    1    aou
................................................................cugcacuccaNccugggcaa........................  1    1    aou
................................................................cugcacuccaNccugggcaac.......................  1    1    aou
.................................................................ugcacuccagAcugggca.........................  1    1    aou
.................................................................ugcacucNagccugggcaa........................  1    1    aou
.................................................................ugcacuccagAcugggcaa........................  1    1    aou
.................................................................ugcacuccagNcugggcaa........................  1    1    aou
.................................................................ugcaUuccagccugggcaa........................  1    1    aou
.................................................................ugcacuccagNcugggcaaca......................  2    1    aou
..................................................................gcacuccagcUugggcaa........................  1    1    aou
..................................................................gcacuGcagccugggcaa........................  2    1    aou
..................................................................gcacuUcagccugggcaac.......................  1    1    aou
..................................................................gcacuGcagccugggcaac.......................  1    1    aou
...................................................................cacuccaNccugggcaac.......................  1    1    aou
...................................................................cacuccagccuggUcaac.......................  1    1    aou
...................................................................cacuGcagccugggcaac.......................  1    1    aou
...................................................................cacuccagcUugggcaaca......................  2    1    aou
...................................................................cacuccagccugUgcaaca......................  1    1    aou
...................................................................cacuGcagccugggcaaca......................  1    1    aou
....................................................................acuccagcAugggcaaca......................  1    1    aou
....................................................................acuccagccugAgcaaca......................  12   1    aou
....................................................................acuccagAcugggcaaca......................  1    1    aou
....................................................................acuccagccAgggcaaca......................  1    1    aou
....................................................................acuccagcUugggcaaca......................  1    1    aou
....................................................................acuccagcGugggcaacag.....................  1    1    aou
.....................................................................cuccagcAugggcaacaga....................  1    1    aou
.....................................................................cuccagGcugggcaacaga....................  1    1    aou
......................................................................uccagccuggNcaacaga....................  1    1    aou
......................................................................uccagccugggcaCcagaa...................  1    1    aou
......................................................................uccagAcugggcaacagaa...................  1    1    aou
........................................................................cagccuggAcaacagaagga................  1    1    aou
.........................................................................Cgccugggcaacagaaggagacu............  1    1    aou
..........................................................................gccugggcaacagaaUga................  1    1    aou
..........................................................................gccugggcaacagaaCga................  1    1    aou
..........................................................................gccugggcaacagaaAgagac.............  1    1    aou
..........................................................................gccugggcaacagaagUagacu............  1    1    aou
..........................................................................Accugggcaacagaaggagacu............  3    1    aou
..........................................................................gccugggcaacagaaggagacu............  1    0    aou
...........................................................................ccugggcaacagaaCgaga..............  1    1    aou
...........................................................................ccugggcaacagaaUgaga..............  1    1    aou
...........................................................................ccugggcaacagaaUgagac.............  1    1    aou
...........................................................................ccugggcaacagaagUagacu............  1    1    aou
............................................................................cugggcaacagaGggaga..............  2    1    aou
............................................................................cugggcaacagaaAgagacu............  1    1    aou
............................................................................cugggcaacagaaggagacu............  1    0    aou
.............................................................................ugggcaacagaGggagacu............  3    1    aou
.............................................................................ugggcaacagaaggagacu............  2    0    aou
...............................................................................ggcaacagaaCgagacua...........  3    1    aou
```
