# Supplementary material for: SEAweb: the small RNA Expression Atlas web application
Source: Nucleic Acids Res. 2019 Oct 10;48(D1):D204–19. doi: 10.1093/nar/gkz869 (PMC6943056; doi:10.1093/nar/gkz869)
Supplement: gkz869_Supplemental_Files [file gkz869_supplemental_files.zip › p-hsa-miR-235-1.pdf]

Provisional ID	:	chrUn_GL000220v1_21620
Score total	:	144078.7
Score for star read(s)	:	3.9
Score for read counts	:	144071.6
Score for mfe	:	2.2
Score for randfold	:	1.6
Score for cons. seed	:	-0.6
Total read count	:	282602
Mature read count	:	36506
Loop read count	:	245911
Star read count	:	185

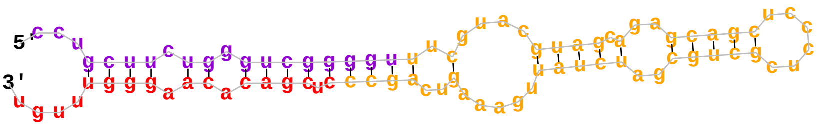

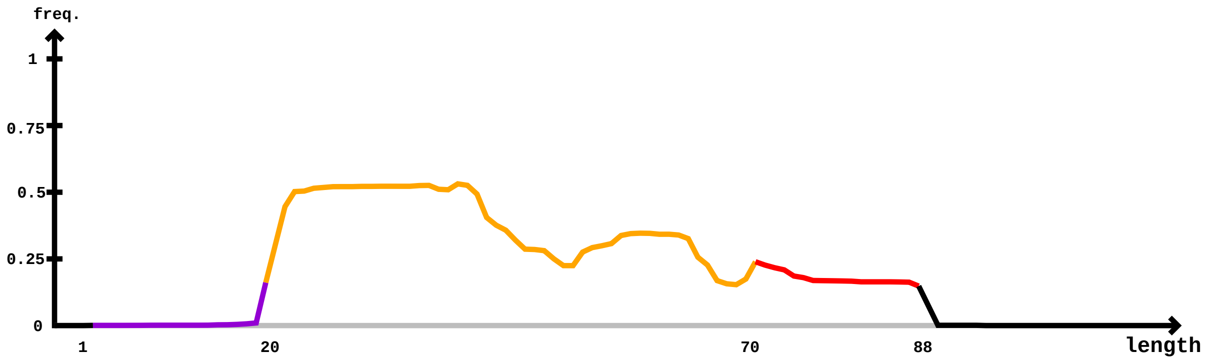

Star Mature

```
5'- gaccugcuucugggucgggguuucguacguagcagagcagcucccucgcugcgaucuauugaaagucagcccucgacacaaggguuuguccgcgcgcgcgcgcgcgcgcgugc  -3'   obs
    gaccugcuucugggucgggguuucguacguagcagagcagcucccucgcugcgaucuauugaaagucagcccucgacacaaggguuuguccgcgcgcgcgcgcgcgcgcgugc        exp
    (((..((((.((.(((((((((.(....((((.(..(((((......)))))..))))).....)..))))).)))).)).)))).))).((((((((((....))))))))))  reads      mm         sample
    ..ccugcuucugggucgggg...............................................................................................  7          0          7y1
    ..ccugcuucugggucggggG..............................................................................................  6          1          7y1
    ..ccugcuucugggucggggA..............................................................................................  1          1          7y1
    ..cAugcuucugggucggggu..............................................................................................  1          1          7y1
    ..ccugcuucugggucggggu..............................................................................................  164        0          7y1
    ...cugcuucuggguAgggguuucgu.........................................................................................  2          1          7y1
    ...cugcuucugUgucgggguuucgu.........................................................................................  1          1          7y1
    ...cugcuucugggucgggguAucgu.........................................................................................  1          1          7y1
    ...cugcuucugggucgggUuuucgu.........................................................................................  2          1          7y1
    ....ugcuucAgggucgggguu.............................................................................................  1          1          7y1
    .....gcuucugggAcgggguuucgu.........................................................................................  1          1          7y1
    .....gcuucuggguAgggguuucgu.........................................................................................  1          1          7y1
    .....gcuucuggCucgggguuucgu.........................................................................................  1          1          7y1
    .....gcuucuggUucgggguuucgu.........................................................................................  1          1          7y1
    ......cuucugggucgggguGuc...........................................................................................  1          1          7y1
    ......cuucuAggucgggguuuc...........................................................................................  1          1          7y1
    ......cuucugggucgggguuuG...........................................................................................  1          1          7y1
    ......cuucugggAcgggguuuc...........................................................................................  1          1          7y1
    ......cuucugggucggCguuuc...........................................................................................  1          1          7y1
    ......cuucuUggucgggguuucgu.........................................................................................  1          1          7y1
    ......cuucuggguGgggguuucgu.........................................................................................  1          1          7y1
    ......cuucugggucgggguCucgu.........................................................................................  1          1          7y1
    ......cuucugggucUggguuucgu.........................................................................................  1          1          7y1
    ......cuucugggAcgggguuucgu.........................................................................................  2          1          7y1
    ......cuucuCggucgggguuucgu.........................................................................................  1          1          7y1
    ......cuucuggguAgggguuucgu.........................................................................................  4          1          7y1
    ......cuucugggucCggguuucgu.........................................................................................  1          1          7y1
    ......cuucugggucgggguAucgu.........................................................................................  1          1          7y1
    ......cuucugggucggggGuucgu.........................................................................................  2          1          7y1
    ......cuucugggucgggAuuucgu.........................................................................................  1          1          7y1
    ......cuucugggucggUguuucgu.........................................................................................  1          1          7y1
    ......cuucugggucggggAuucgu.........................................................................................  1          1          7y1
    ......cuucugggucgggUuuucgu.........................................................................................  1          1          7y1
```

Star Mature

```
gaccugcuucugggucgggguuucguacguagcagagcagcucccucgcugcgaucuauugaaagucagcccucgacacaaggguuuguccgcgcgcgcgcgcgcgcgcgcgugc
......cuucugggucggCguuucgu.........................................................................................   1   1   7y1
......cuucugggucgggguuucUu.........................................................................................   3   1   7y1
.......uucugggucgggguCucgu.........................................................................................   2   1   7y1
.......uucuUggucgggguuucgu.........................................................................................   2   1   7y1
.......uucugggAcgggguuucgu.........................................................................................   3   1   7y1
.......uucugggucggUguuucgu.........................................................................................   1   1   7y1
.......uucugggucgggCuuucgu.........................................................................................   4   1   7y1
.......uucugggucgUgguuucgu.........................................................................................   2   1   7y1
.......uucugggucggggCuucgu.........................................................................................   1   1   7y1
.......uucuggguAgggguuucgu.........................................................................................   6   1   7y1
.......uucugggucgggguAucgu.........................................................................................   3   1   7y1
.......uucugCgucgggguuucgu.........................................................................................   4   1   7y1
.......uucugggucggCguuucgu.........................................................................................   1   1   7y1
.......uucugggucUggguuucgu.........................................................................................   2   1   7y1
.......uucugggucggggGuucgu.........................................................................................   1   1   7y1
.......uucuggUucgggguuucgu.........................................................................................   2   1   7y1
.......uucugUgucgggguuucgu.........................................................................................   1   1   7y1
........ucuggCucgggguuucgu.........................................................................................   4   1   7y1
........ucugggucgggCuuucgu.........................................................................................   1   1   7y1
........ucugggucgggguAucgu.........................................................................................   4   1   7y1
........ucugggucgCgguuucgu.........................................................................................   8   1   7y1
........ucuAggucgggguuucgu.........................................................................................   2   1   7y1
........ucugCgucgggguuucgu.........................................................................................   1   1   7y1
........ucuggUucgggguuucgu.........................................................................................   7   1   7y1
........ucugggucCggguuucgu.........................................................................................   2   1   7y1
........ucuggAucgggguuucgu.........................................................................................   1   1   7y1
........ucugggucggUguuucgu.........................................................................................   3   1   7y1
........ucugggucUggguuucgu.........................................................................................   2   1   7y1
........ucugggucgggguCucgu.........................................................................................   1   1   7y1
........ucugggucgUgguuucgu.........................................................................................   1   1   7y1
........ucuggguAgggguuucgu.........................................................................................   4   1   7y1
........ucugggucggggAuucgu.........................................................................................   1   1   7y1
........ucugUgucgggguuucgu.........................................................................................   2   1   7y1
........ucugggAcgggguuucgu.........................................................................................   4   1   7y1
........ucugggucgggUuuucgu.........................................................................................   1   1   7y1
........ucuggguGgggguuucgu.........................................................................................   1   1   7y1
........ucugggucgggguuAcgu.........................................................................................   1   1   7y1
........ucugggucggCguuucgu.........................................................................................   6   1   7y1
........ucugggucggCguuucgua........................................................................................   2   1   7y1
........ucugUgucgggguuucgua........................................................................................   2   1   7y1
........ucugggucgCgguuucgua........................................................................................   6   1   7y1
........ucugggucgggCuuucgua........................................................................................   1   1   7y1
........ucuggAucgggguuucgua........................................................................................   1   1   7y1
........ucugggucggAguuucgua........................................................................................   1   1   7y1
........ucugggucgggUuuucgua........................................................................................   2   1   7y1
........ucugggucggggAuucgua........................................................................................   1   1   7y1
........ucugggucgUgguuucgua........................................................................................   3   1   7y1
........ucuggguGgggguuucgua........................................................................................   1   1   7y1
........ucugggucgggguuGcgua........................................................................................   1   1   7y1
........ucugggucgggguuAcgua........................................................................................   1   1   7y1
........ucugggucUggguuucgua........................................................................................   3   1   7y1
........ucugggucCggguuucgua........................................................................................   2   1   7y1
........ucugCgucgggguuucgua........................................................................................   2   1   7y1
........ucuggUucgggguuucgua........................................................................................   5   1   7y1
........ucugggAcgggguuucgua........................................................................................   12  1   7y1
........ucugggucgggguAucgua........................................................................................   2   1   7y1
........ucugggucggUguuucgua........................................................................................   4   1   7y1
........ucuggguAgggguuucgua........................................................................................   4   1   7y1
.........cugCgucgggguuucgua........................................................................................   1   1   7y1
.........cuggguAgggguuucgua........................................................................................   5   1   7y1
.........cugggucggUguuucgua........................................................................................   1   1   7y1
.........cugggucgggguuuAgua........................................................................................   5   1   7y1
.........cugggucgggUuuucgua........................................................................................   1   1   7y1
.........cugggucgAgguuucgua........................................................................................   1   1   7y1
.........cugggucgggguuuGgua........................................................................................   1   1   7y1
.........cugggucUggguuucgua........................................................................................   1   1   7y1
.........cugggucgCgguuucgua........................................................................................   1   1   7y1
.........cugggucggggGuucgua........................................................................................   1   1   7y1
.........cugggAcgggguuucguac.......................................................................................   1   1   7y1
.........cuggguAgggguuucguac.......................................................................................   1   1   7y1
```

Star **Mature**

```
gaccugcuucugggucgggguuucguacguagcagagcagcucccucgcugcgaucuauugaaagucagcccucgacacaaggguuuguccgcgcgcgcgcgcgcgcgcgcgugc
.........cugggucgggguuAcguac.......................................................................................   1   1   7y1
.........cugggucgggUuuucguac.......................................................................................   1   1   7y1
.........cugggucCggguuucguac.......................................................................................   1   1   7y1
.........cugggucgggguuuGguac.......................................................................................   1   1   7y1
.........cugggucggCguuucguac.......................................................................................   1   1   7y1
.........cugggucgggguuuAguac.......................................................................................   1   1   7y1
.............ucgggguAucguacguag....................................................................................   1   1   7y1
.............ucggggAuucguacguag....................................................................................   1   1   7y1
.............ucggggAuucguacguagc...................................................................................   1   1   7y1
.............ucggggCuucguacguagc...................................................................................   1   1   7y1
.............ucgggguAucguacguagc...................................................................................   1   1   7y1
.............ucgggUuuucguacguagc...................................................................................   1   1   7y1
.............ucggggGuucguacguagc...................................................................................   1   1   7y1
.............ucgggguuucguaUguagc...................................................................................   1   1   7y1
.............ucgggguuucAuacguagcagagc..............................................................................   2   1   7y1
.............ucgggUuuucguacguagcagagcagcuc.........................................................................   4   1   7y1
.............ucgggguCucguacguagcagagcagcuc.........................................................................   1   1   7y1
.............ucgggCuuucguacguagcagagcagcuc.........................................................................   6   1   7y1
.............ucggggAuucguacguagcagagcagcuc.........................................................................   23  1   7y1
.............ucggggCuucguacguagcagagcagcuc.........................................................................   2   1   7y1
.............ucgggguGucguacguagcagagcagcuc.........................................................................   1   1   7y1
...............cgggguuucgCacguagc..................................................................................   1   1   7y1
...............cggAguuucguacguagc..................................................................................   1   1   7y1
...............cgggguuAcguacguagc..................................................................................   1   1   7y1
...............cgggguuucUuacguagc..................................................................................   1   1   7y1
...............cggggAuucguacguagc..................................................................................   2   1   7y1
...............cggCguuucguacguagc..................................................................................   1   1   7y1
...............cgggguAucguacguagc..................................................................................   3   1   7y1
...............cgggguuucguaAguagc..................................................................................   1   1   7y1
...............cgggguuucguacAuagca.................................................................................   1   1   7y1
...............cgggguuucUuacguagca.................................................................................   1   1   7y1
...............cggggAuucguacguagca.................................................................................   2   1   7y1
...............cgggguuucguaAguagca.................................................................................   1   1   7y1
...............cgggguuuAguacguagcag................................................................................   1   1   7y1
...............cgggguuucCuacguagcag................................................................................   1   1   7y1
...............cggggAuucguacguagcag................................................................................   1   1   7y1
...............cgggUuuucguacguagcag................................................................................   1   1   7y1
...............cgggguuucguacgAagcag................................................................................   1   1   7y1
...............cgggguuucguacCuagcag................................................................................   1   1   7y1
...............Agggguuucguacguagcag................................................................................   1   1   7y1
...............cgggguuucUuacguagcaga...............................................................................   1   1   7y1
...............cgggguuuUguacguagcaga...............................................................................   1   1   7y1
...............cgggCuuucguacguagcaga...............................................................................   3   1   7y1
...............cgggguGucguacguagcaga...............................................................................   1   1   7y1
...............cgggguuucguaAguagcaga...............................................................................   3   1   7y1
...............cgggguuuGguacguagcaga...............................................................................   1   1   7y1
...............cgggguuucguacgCagcaga...............................................................................   1   1   7y1
...............cgggguAucguacguagcaga...............................................................................   4   1   7y1
...............cgggguuAcguacguagcaga...............................................................................   2   1   7y1
...............cgggguuucCuacguagcaga...............................................................................   1   1   7y1
...............cgggguuucguaGguagcaga...............................................................................   1   1   7y1
...............cggggGuucguacguagcaga...............................................................................   1   1   7y1
...............cgggguuucguacUuagcaga...............................................................................   4   1   7y1
...............cgggguuuAguacguagcaga...............................................................................   1   1   7y1
...............cggggAuucguacguagcaga...............................................................................   5   1   7y1
...............cgggguuucgAacguagcagag..............................................................................   1   1   7y1
...............cgggguuucguaAguagcagag..............................................................................   3   1   7y1
...............cgggguuuAguacguagcagag..............................................................................   1   1   7y1
...............cgggguuucguacUuagcagag..............................................................................   1   1   7y1
...............cgggguuucAuacguagcagag..............................................................................   1   1   7y1
...............cgggUuuucguacguagcagag..............................................................................   1   1   7y1
...............cgggguuucUuacguagcagag..............................................................................   1   1   7y1
...............cgggguAucguacguagcagag..............................................................................   2   1   7y1
...............cgggUuuucguacguagcagagc.............................................................................   2   1   7y1
...............cgggguuucguacUuagcagagc.............................................................................   2   1   7y1
...............cgggguuucguGcguagcagagc.............................................................................   1   1   7y1
...............cgggguuucAuacguagcagagc.............................................................................   1   1   7y1
...............cgggCuuucguacguagcagagc.............................................................................   3   1   7y1
...............cgggguuuAguacguagcagagc.............................................................................   7   1   7y1
...............cgggguuucgGacguagcagagc.............................................................................   1   1   7y1
```

Star **Mature**

gaccugcuucugggucgggguuucguacguagcagagcagcucccucgcugcgaucuauugaaagucagcccucgacacaaggguuuguccgcgcgcgcgcgcgcgcgcgugc

...............cgggguuucguacCuagcagagc...	1	1	7y1
...............cgggguuucCuacguagcagagc...	2	1	7y1
...............cgggguuucguacAuagcagagc...	1	1	7y1
...............cggggGuucguacguagcagagc...	3	1	7y1
...............cgggguuucguacgAagcagagc...	3	1	7y1
...............cgggguuuGguacguagcagagc...	1	1	7y1
...............cgggguuucguaAguagcagagc...	11	1	7y1
...............cgggguuucgCacguagcagagc...	3	1	7y1
...............cggggAuucguacguagcagagc...	27	1	7y1
...............cgggguuucUuacguagcagagc...	1	1	7y1
...............cgggguuucgAacguagcagagc...	3	1	7y1
...............cgggguuCcguacguagcagagc...	2	1	7y1
...............cgggguCucguacguagcagagc...	1	1	7y1
...............cgggguAucguacguagcagagc...	14	1	7y1
...............cgggguuAcguacguagcagagc...	3	1	7y1
...............cgggguuucguaUguagcagagc...	1	1	7y1
...............cgggguuucguacgAagcagagca..	3	1	7y1
...............cggggGuucguacguagcagagca..	1	1	7y1
...............cgggguuucCuacguagcagagca..	2	1	7y1
...............cgggguuucguacUuagcagagca..	2	1	7y1
...............cgggguuucgGacguagcagagca..	1	1	7y1
...............cgggguuucguacgGagcagagca..	1	1	7y1
...............cgggguuuUguacguagcagagca..	1	1	7y1
...............cgggguuucUuacguagcagagca..	4	1	7y1
...............cgggguuucguGcguagcagagca..	2	1	7y1
...............cgggguuucgAacguagcagagca..	3	1	7y1
...............cgggguuAcguacguagcagagca..	6	1	7y1
...............cgggguuucguaAguagcagagca..	4	1	7y1
...............cgggCuuucguacguagcagagca..	3	1	7y1
...............cgggUuuucguacguagcagagca..	1	1	7y1
...............cgggguAucguacguagcagagca..	10	1	7y1
...............cgggAuuucguacguagcagagca..	1	1	7y1
...............cgggguuucgCacguagcagagca..	1	1	7y1
...............cgggguuuAguacguagcagagca..	5	1	7y1
...............cggggAuucguacguagcagagca..	10	1	7y1
...............cgggguCucguacguagcagagca..	1	1	7y1
...............cgggguuucAuacguagcagagca..	1	1	7y1
...............cgggguAucguacguagcagagcag..	1	1	7y1
...............cgggCuuucguacguagcagagcag..	1	1	7y1
...............cgggguuAcguacguagcagagcag..	1	1	7y1
...............cgggUuuucguacguagcagagcag..	2	1	7y1
...............cgggguuucguacUuagcagagcag..	1	1	7y1
...............cgggguuucgAacguagcagagcag..	1	1	7y1
...............cgggguuuGguacguagcagagcag..	1	1	7y1
...............cgggguuucCuacguagcagagcag..	1	1	7y1
...............cgggguuucguaAguagcagagcag..	1	1	7y1
...............cgggguuuAguacguagcagagcag..	4	1	7y1
...............cgggguAucguacguagcagagcagc...	9	1	7y1
...............cgggguuucguacgAagcagagcagc...	2	1	7y1
...............cgggguuucgAacguagcagagcagc...	4	1	7y1
...............cggggGuucguacguagcagagcagc...	1	1	7y1
...............cgggguGucguacguagcagagcagc...	1	1	7y1
...............cggggAuucguacguagcagagcagc...	10	1	7y1
...............cgggguuucgGacguagcagagcagc...	1	1	7y1
...............cgggguuucguacUuagcagagcagc...	2	1	7y1
...............cggggCuucguacguagcagagcagc...	1	1	7y1
...............cgggguuAcguacguagcagagcagc...	2	1	7y1
...............cgggguuucguaGguagcagagcagc...	1	1	7y1
...............cgggguuuAguacguagcagagcagc...	2	1	7y1
...............cgggguuuUguacguagcagagcagc...	1	1	7y1
...............cgggguuucguaAguagcagagcagc...	5	1	7y1
...............cgggguuCcguacguagcagagcagc...	1	1	7y1
...............cgggguuucAuacguagcagagcagc...	2	1	7y1
...............cgggguuucguacCuagcagagcagc...	1	1	7y1
...............cgggguAucguacguagcagagcagcu..	12	1	7y1
...............cggggGuucguacguagcagagcagcu..	3	1	7y1
...............cgggguuucguacUuagcagagcagcu..	2	1	7y1
...............cgggguuuAguacguagcagagcagcu..	1	1	7y1
...............cggggCuucguacguagcagagcagcu..	1	1	7y1
...............cggggAuucguacguagcagagcagcu..	10	1	7y1

Star Mature

gaccugcuucugggucgggguuucguacguagcagagcagcucccucgcugcgaucuauugaaagucagcccucgacacaaggguuuguccgcgcgcgcgcgcgcgcgugc			
...............cgggguuucguacgAagcagagcagcu...	1	1	7y1
...............cgggguuucgAacguagcagagcagcu...	4	1	7y1
...............cggggugCucguacguagcagagcagcu...	1	1	7y1
...............cgggguuucguaAguagcagagcagcu...	2	1	7y1
...............cgggguuucCuacguagcagagcagcu...	1	1	7y1
...............cgggguuucguGcguagcagagcagcu...	1	1	7y1
...............cgggguuucAuacguagcagagcagcu...	2	1	7y1
...............cgggguuAcguacguagcagagcagcu...	4	1	7y1
...............cgggUuucguacguagcagagcagcu...	2	1	7y1
...............cgggguuucgGacguagcagagcagcu...	1	1	7y1
...............cgggguuuUguacguagcagagcagcu...	1	1	7y1
...............cgggguuucUuacguagcagagcagcu...	1	1	7y1
...............cgggguGucguacguagcagagcagcuc..	2	1	7y1
...............cgggguuucCuacguagcagagcagcuc..	1	1	7y1
...............cgggguuucAuacguagcagagcagcuc..	2	1	7y1
...............cgggUuucguacguagcagagcagcuc..	2	1	7y1
...............cggggAuucguacguagcagagcagcuc..	12	1	7y1
...............cgggguuAcguacguagcagagcagcuc..	7	1	7y1
...............cggggGuucguacguagcagagcagcuc..	4	1	7y1
...............cgggguuucgAacguagcagagcagcuc..	2	1	7y1
...............cgggguuucUuacguagcagagcagcuc..	1	1	7y1
...............cgggguuucguacCuagcagagcagcuc..	1	1	7y1
...............cgggguuucgCacguagcagagcagcuc..	4	1	7y1
...............cgggguAucguacguagcagagcagcuc..	16	1	7y1
...............cgggCuuucguacguagcagagcagcuc..	2	1	7y1
...............cgggguuucguaAguagcagagcagcuc..	1	1	7y1
...............cgggguuucguacgAagcagagcagcuc..	1	1	7y1
...............cgggguuucguaUguagcagagcagcuc..	1	1	7y1
...............cgggguuuGguacguagcagagcagcuc..	1	1	7y1
...............cgggguuucguacUuagcagagcagcuc..	3	1	7y1
...............cgggguuuAguacguagcagagcagcuc..	5	1	7y1
...............cggggAuucguacguagcagagcagcucccuc..	1	1	7y1
................gggguuucguaAguagcaga...	1	1	7y1
................gggguuAcguacguagcaga...	2	1	7y1
................gggguuucgAacguagcagag..	1	1	7y1
................gggguuuAguacguagcagag..	1	1	7y1
................gggguuuAguacguagcagagc...	2	1	7y1
................gggguuucUuacguagcagagc...	1	1	7y1
................gggguAucguacguagcagagc...	5	1	7y1
................gggguuAcguacguagcagagc...	3	1	7y1
................gggUuucguacguagcagagc...	3	1	7y1
................gggguuucgAacguagcagagca..	1	1	7y1
................gggguuAcguacguagcagagca..	2	1	7y1
................gggguuucguaAguagcagagca..	1	1	7y1
................gggguuuAguacguagcagagca..	1	1	7y1
................gggguAucguacguagcagagca..	2	1	7y1
................gggUuucguacguagcagagca..	1	1	7y1
................gggguuucguacguGgcagagcagc..	1	1	7y1
................gggguGucguacguagcagagcagc..	1	1	7y1
................gggguuAcguacguagcagagcagc..	3	1	7y1
................gggguuCcguacguagcagagcagc..	1	1	7y1
................gggguuucgAacguagcagagcagc..	1	1	7y1
................gggguuucguacgCagcagagcagc..	1	1	7y1
................gggguuuAguacguagcagagcagc..	3	1	7y1
................gggguuucguUcguagcagagcagc..	2	1	7y1
................ggggGuucguacguagcagagcagc..	1	1	7y1
................gggguAucguacguagcagagcagc..	3	1	7y1
................gggguuucguacgAagcagagcagc..	1	1	7y1
................ggggAuucguacguagcagagcagcu...	2	1	7y1
................gggguuucguacgCagcagagcagcu...	2	1	7y1
................ggggGuucguacguagcagagcagcu...	1	1	7y1
................gggguuucCuacguagcagagcagcu...	1	1	7y1
................gggguuucguGcguagcagagcagcu...	1	1	7y1
................gggguuucguacUuagcagagcagcu...	1	1	7y1
................gggCuuucguacguagcagagcagcu...	1	1	7y1
................gggguuucguaAguagcagagcagcu...	1	1	7y1
................gggguAucguacguagcagagcagcu...	6	1	7y1
................gggguuAcguacguagcagagcagcu...	1	1	7y1
................gggguuuAguacguagcagagcagcu...	1	1	7y1
................gggguuucguGcguagcagagcagcuc..	1	1	7y1

Star **Mature**

gaccugcuucugggucggggguucguacguagcagagcagcucccucgcugcgaucuauugaaagucagcccucgacacaaggguuuguccgcgcgcgcgcgcgcgcgugc			
................gggUuuucguacguagcagagcagcuc..	1	1	7y1
................gggguuGcguacguagcagagcagcuc..	1	1	7y1
................gggguuucguacAuagcagagcagcuc..	2	1	7y1
................ggggAuucguacguagcagagcagcuc..	1	1	7y1
................gggguuAcguacguagcagagcagcuc..	4	1	7y1
................gggguuucguacguGgcagagcagcuc..	1	1	7y1
................gggguuucAuacguagcagagcagcuc..	1	1	7y1
................gggguuuAguacguagcagagcagcuc..	8	1	7y1
................gggguAucguacguagcagagcagcuc..	9	1	7y1
................gggguuucguaAguagcagagcagcuc..	3	1	7y1
................gggguuucCuacguagcagagcagcuc..	1	1	7y1
................gggguuucguacUuagcagagcagcuc..	3	1	7y1
................gggguuucguacguUgcagagcagcuc..	1	1	7y1
................gggguuucgAacguagcagagcagcuc..	2	1	7y1
................gggguCucguacguagcagagcagcuc..	2	1	7y1
.................ggguuucguacgCagcaga...	1	1	7y1
.................ggguuucguaAguagcaga...	1	1	7y1
.................ggguuuAguacguagcaga...	1	1	7y1
.................ggguuAcguacguagcaga...	1	1	7y1
.................ggguuucAuacguagcagagc...	1	1	7y1
.................ggguuAcguacguagcagagc...	1	1	7y1
.................ggguuucguaAguagcagagc...	1	1	7y1
.................ggguAucguacguagcagagc...	2	1	7y1
.................ggguuuAguacguagcagagc...	5	1	7y1
.................gggAuucguacguagcagagc...	1	1	7y1
.................ggguuucgAacguagcagagc...	2	1	7y1
.................ggguuucguaUguagcagagc...	1	1	7y1
.................gggGuucguacguagcagagc...	5	1	7y1
.................ggguuAcguacguagcagagca..	1	1	7y1
.................ggguuucgGacguagcagagca..	1	1	7y1
.................ggguuucguacguGgcagagca..	1	1	7y1
.................ggguuucUuacguagcagagca..	1	1	7y1
.................ggguGucguacguagcagagca..	1	1	7y1
.................ggguuCcguacguagcagagca..	1	1	7y1
.................gggGuucguacguagcagagca..	1	1	7y1
.................ggguuucguacguaCcagagca..	1	1	7y1
.................ggguuuAguacguagcagagca..	3	1	7y1
.................ggCuuucguacguagcagagca..	269	1	7y1
.................ggguuucguacguaUcagagca..	1	1	7y1
.................ggguuucgAacguagcagagca..	1	1	7y1
.................ggguCucguacguagcagagca..	1	1	7y1
.................ggguAucguacguagcagagca..	1	1	7y1
.................ggguuucguaAguagcagagca..	1	1	7y1
.................ggguuuGguacguagcagagca..	1	1	7y1
.................ggguuucguaAguagcagagcagc..	1	1	7y1
.................gggGuucguacguagcagagcagc..	1	1	7y1
.................ggguuucgAacguagcagagcagc..	1	1	7y1
.................ggguuuAguacguagcagagcagc..	2	1	7y1
.................gggGuucguacguagcagagcagcu...	2	1	7y1
.................ggguuucguacCuagcagagcagcu...	1	1	7y1
.................ggguuAcguacguagcagagcagcu...	5	1	7y1
.................ggguAucguacguagcagagcagcu...	2	1	7y1
.................ggguuucguaUguagcagagcagcu...	1	1	7y1
.................ggguuuAguacguagcagagcagcu...	6	1	7y1
.................ggCuuucguacguagcagagcagcu...	1	1	7y1
.................ggguuucguacguaUcagagcagcu...	1	1	7y1
.................ggguuucCuacguagcagagcagcu...	1	1	7y1
.................ggguuucguaAguagcagagcagcu...	1	1	7y1
.................ggguuucgAacguagcagagcagcu...	1	1	7y1
.................gggGuucguacguagcagagcagcuc..	2	1	7y1
.................ggguuucguaAguagcagagcagcuc..	2	1	7y1
.................ggguuCcguacguagcagagcagcuc..	1	1	7y1
.................ggguuucguacUuagcagagcagcuc..	1	1	7y1
.................ggguuAcguacguagcagagcagcuc..	6	1	7y1
.................ggguuuAguacguagcagagcagcuc..	9	1	7y1
.................ggguuucAuacguagcagagcagcuc..	2	1	7y1
.................ggguAucguacguagcagagcagcuc..	5	1	7y1
.................ggguuucguacgAagcagagcagcuc..	2	1	7y1
.................ggguuucguacguCgcagagcagcuc..	1	1	7y1
.................ggguuucguaUguagcagagcagcuc..	1	1	7y1

Star **Mature**

```
gaccugcuucugggucgggguuucguacguagcagagcagcucccucgcugcgaucuauugaaagucagcccucgacacaaggguuuguccgcgcgcgcgcgcgcgcgcgugc
.................ggguuucUuacguagcagagcagcuc......................................................................  1  1  7y1
.................gggCuucguacguagcagagcagcuc......................................................................  1  1  7y1
.................ggguuucguacCuagcagagcagcuc......................................................................  1  1  7y1
.................ggguuucgAacguagcagagcagcuc......................................................................  1  1  7y1
.................ggguuucguacgAagcagagcagcucc.....................................................................  1  1  7y1
.................ggguuucguacguGgcagagcagcucccu...................................................................  1  1  7y1
.................ggguuuAguacguagcagagcagcucccu...................................................................  1  1  7y1
.................ggguuAcguacguagcagagcagcucccu...................................................................  1  1  7y1
.................gggGuucguacguagcagagcagcucccu...................................................................  1  1  7y1
.................ggguuucgAacguagcagagcagcucccu...................................................................  1  1  7y1
..................gUuuucguacguagcagagc...........................................................................  3  1  7y1
..................gguuucguacguagAagagc...........................................................................  5  1  7y1
..................gguuucguacgGagcagagc...........................................................................  1  1  7y1
..................gguuucguUcguagcagagc...........................................................................  1  1  7y1
..................ggAuucguacguagcagagc...........................................................................  4  1  7y1
..................gguuuGguacguagcagagc...........................................................................  2  1  7y1
..................gguuAcguacguagcagagc...........................................................................  1  1  7y1
..................gguGucguacguagcagagc...........................................................................  1  1  7y1
..................gCuuucguacguagcagagc...........................................................................  2  1  7y1
..................gguuucguaGguagcagagc...........................................................................  1  1  7y1
..................gguuucguacguaUcagagc...........................................................................  2  1  7y1
..................gguuucguaAguagcagagc...........................................................................  16  1  7y1
..................gguuucgAacguagcagagc...........................................................................  9  1  7y1
..................gguuucCuacguagcagagc...........................................................................  4  1  7y1
..................gguCucguacguagcagagc...........................................................................  1  1  7y1
..................gguuuAguacguagcagagc...........................................................................  11  1  7y1
..................gguuucguacguagGagagc...........................................................................  1  1  7y1
..................gguuucguacguUgcagagc...........................................................................  1  1  7y1
..................gguuucguacguaCcagagc...........................................................................  3  1  7y1
..................gguuucAuacguagcagagc...........................................................................  3  1  7y1
..................gguuucguacguGgcagagc...........................................................................  1  1  7y1
..................gguuucguacUuagcagagc...........................................................................  1  1  7y1
..................gguAucguacguagcagagc...........................................................................  3  1  7y1
..................gguuucguacCuagcagagc...........................................................................  2  1  7y1
..................ggGuucguacguagcagagc...........................................................................  1  1  7y1
..................gguuucguacgAagcagagc...........................................................................  5  1  7y1
..................gguuucCuacguagcagagca..........................................................................  2  1  7y1
..................gCuuucguacguagcagagca..........................................................................  1  1  7y1
..................gguuAcguacguagcagagca..........................................................................  1  1  7y1
..................gguuucguaUguagcagagca..........................................................................  1  1  7y1
..................gguuuAguacguagcagagca..........................................................................  3  1  7y1
..................gguuucgGacguagcagagca..........................................................................  1  1  7y1
..................gguuucguacguagUagagca..........................................................................  1  1  7y1
..................gguuucguaAguagcagagca..........................................................................  3  1  7y1
..................gguuucguacCuagcagagca..........................................................................  1  1  7y1
..................ggAuucguacguagcagagca..........................................................................  1  1  7y1
..................gguuucguacguaAcagagca..........................................................................  1  1  7y1
..................gguuucgAacguagcagagca..........................................................................  1  1  7y1
..................gguCucguacguagcagagca..........................................................................  1  1  7y1
..................gguuucguacguaCcagagca..........................................................................  2  1  7y1
..................gUuuucguacguagcagagcag.........................................................................  1  1  7y1
..................gguuucgAacguagcagagcag.........................................................................  2  1  7y1
..................gguAucguacguagcagagcag.........................................................................  1  1  7y1
..................gguuuAguacguagcagagcag.........................................................................  1  1  7y1
..................ggAuucguacguagcagagcag.........................................................................  1  1  7y1
..................gguuucCuacguagcagagcag.........................................................................  1  1  7y1
..................gguuucguaGguagcagagcagc........................................................................  2  1  7y1
..................gguuucguacguagGagagcagc........................................................................  3  1  7y1
..................gCuuucguacguagcagagcagc........................................................................  3  1  7y1
..................gguuucguaUguagcagagcagc........................................................................  1  1  7y1
..................gguuucguacguaCcagagcagc........................................................................  2  1  7y1
..................gAuuucguacguagcagagcagc........................................................................  1  1  7y1
..................gUuuucguacguagcagagcagc........................................................................  6  1  7y1
..................gguuucguacguagUagagcagc........................................................................  2  1  7y1
..................gguuucguacUuagcagagcagc........................................................................  3  1  7y1
..................gguuucguacCuagcagagcagc........................................................................  1  1  7y1
..................gguuucguacgCagcagagcagc........................................................................  1  1  7y1
..................gguuucguacguaUcagagcagc........................................................................  4  1  7y1
..................gguuucguacguagAagagcagc........................................................................  4  1  7y1
..................gguAucguacguagcagagcagc........................................................................  2  1  7y1
```

Star **Mature**

```
gaccugcuucugggucgggguuucguacguagcagagcagcucccucgcugcgaucuauugaaagucagcccucgacacaaggguuuguccgcgcgcgcgcgcgcgcgcgcgugc
..................ggGuucguacguagcagagcagc..........................................................................   1   1   7y1
..................ggAuucguacguagcagagcagc..........................................................................   7   1   7y1
..................gguuucgAacguagcagagcagc..........................................................................   8   1   7y1
..................gguuucCuacguagcagagcagc..........................................................................   3   1   7y1
..................gguuuAguacguagcagagcagc..........................................................................   7   1   7y1
..................gguCucguacguagcagagcagc..........................................................................   2   1   7y1
..................gguuucguaAguagcagagcagc..........................................................................   5   1   7y1
..................gguuucguacgAagcagagcagc..........................................................................   6   1   7y1
..................gguuucUuacguagcagagcagc..........................................................................   4   1   7y1
..................gguuucguacguGgcagagcagcu.........................................................................   3   1   7y1
..................gguuucguGcguagcagagcagcu.........................................................................   1   1   7y1
..................gguuucguacguagAagagcagcu.........................................................................   10  1   7y1
..................gguuAcguacguagcagagcagcu.........................................................................   5   1   7y1
..................gguuuAguacguagcagagcagcu.........................................................................   20  1   7y1
..................gguuucAuacguagcagagcagcu.........................................................................   2   1   7y1
..................gguuucguacUuagcagagcagcu.........................................................................   1   1   7y1
..................gguuucCuacguagcagagcagcu.........................................................................   2   1   7y1
..................gguuucgAacguagcagagcagcu.........................................................................   10  1   7y1
..................gUuuucguacguagcagagcagcu.........................................................................   4   1   7y1
..................gguuucguacCuagcagagcagcu.........................................................................   7   1   7y1
..................gguuuGguacguagcagagcagcu.........................................................................   2   1   7y1
..................gguuucguacgCagcagagcagcu.........................................................................   1   1   7y1
..................gguuuUguacguagcagagcagcu.........................................................................   1   1   7y1
..................gguuucguUcguagcagagcagcu.........................................................................   2   1   7y1
..................gguuucguaAguagcagagcagcu.........................................................................   20  1   7y1
..................gguuGcguacguagcagagcagcu.........................................................................   1   1   7y1
..................gguuucUuacguagcagagcagcu.........................................................................   4   1   7y1
..................gguuucguacgAagcagagcagcu.........................................................................   6   1   7y1
..................gguuucguacguagGagagcagcu.........................................................................   2   1   7y1
..................gguAucguacguagcagagcagcu.........................................................................   5   1   7y1
..................gCuuucguacguagcagagcagcu.........................................................................   2   1   7y1
..................ggAuucguacguagcagagcagcu.........................................................................   6   1   7y1
..................gguuucguaGguagcagagcagcu.........................................................................   1   1   7y1
..................gguuucguacAuagcagagcagcu.........................................................................   4   1   7y1
..................gguuucguacguaUcagagcagcu.........................................................................   4   1   7y1
..................gguuucguacguaAcagagcagcu.........................................................................   1   1   7y1
..................ggCuucguacguagcagagcagcu.........................................................................   1   1   7y1
..................gguuCcguacguagcagagcagcu.........................................................................   3   1   7y1
..................gguuucguacguagUagagcagcuc........................................................................   1   1   7y1
..................gCuuucguacguagcagagcagcuc........................................................................   3   1   7y1
..................gguuucguacguaUcagagcagcuc........................................................................   1   1   7y1
..................gguuucguacAuagcagagcagcuc........................................................................   2   1   7y1
..................gguuucgCacguagcagagcagcuc........................................................................   1   1   7y1
..................gguuucCuacguagcagagcagcuc........................................................................   1   1   7y1
..................gguuucguaUguagcagagcagcuc........................................................................   1   1   7y1
..................ggGuucguacguagcagagcagcuc........................................................................   2   1   7y1
..................gguuucguaAguagcagagcagcuc........................................................................   6   1   7y1
..................gguuucguacgAagcagagcagcuc........................................................................   4   1   7y1
..................gguuucUuacguagcagagcagcuc........................................................................   1   1   7y1
..................gguuucAuacguagcagagcagcuc........................................................................   1   1   7y1
..................gguuuAguacguagcagagcagcuc........................................................................   3   1   7y1
..................gguuucguacCuagcagagcagcuc........................................................................   2   1   7y1
..................gguuucguacCuagcagagcagcucc.......................................................................   2   1   7y1
..................gguuucguCcguagcagagcagcucc.......................................................................   1   1   7y1
..................gguuucgCacguagcagagcagcucc.......................................................................   1   1   7y1
..................gguuucUuacguagcagagcagcucc.......................................................................   1   1   7y1
..................gguuucguacguaUcagagcagcucc.......................................................................   1   1   7y1
..................gUuuucguacguagcagagcagcucc.......................................................................   1   1   7y1
..................gguuAcguacguagcagagcagcucc.......................................................................   2   1   7y1
..................gguAucguacguagcagagcagcucc.......................................................................   1   1   7y1
..................gguuucguacguagAagagcagcucc.......................................................................   1   1   7y1
..................ggAuucguacguagcagagcagcucc.......................................................................   4   1   7y1
..................gguuucguNcguagcagagcagcucc.......................................................................   1   1   7y1
..................ggGuucguacguagcagagcagcucc.......................................................................   2   1   7y1
..................gguuuAguacguagcagagcagcucc.......................................................................   5   1   7y1
..................gguuucguacgAagcagagcagcucc.......................................................................   2   1   7y1
..................gCuuucguacguagcagagcagcucc.......................................................................   1   1   7y1
..................gguuucCuacguagcagagcagcucc.......................................................................   1   1   7y1
..................gguuucguacAuagcagagcagcucc.......................................................................   1   1   7y1
..................gguuGcguacguagcagagcagcucc.......................................................................   1   1   7y1
```

```
  Star                                Mature
gaccugcuucugggucgggguuucguacguagcagagcagcucccucgcugcgaucuauugaaagucagcccucgacacaaggguuuguccgcgcgcgcgcgcgcgcgcgugc
..................gguuucguUcguagcagagcagcucc.....................................................................    1    1    7y1
..................gguuucguaAguagcagagcagcucc.....................................................................    2    1    7y1
..................gguuucguacAuagcagagcagcuccc....................................................................    1    1    7y1
..................gguAucguacguagcagagcagcuccc....................................................................    2    1    7y1
..................ggCuucguacguagcagagcagcuccc....................................................................    1    1    7y1
..................gguuucguacguaUcagagcagcuccc....................................................................    1    1    7y1
..................gguuucguacgAagcagagcagcuccc....................................................................    2    1    7y1
..................gguuuUguacguagcagagcagcuccc....................................................................    1    1    7y1
..................gguuucUuacguagcagagcagcuccc....................................................................    4    1    7y1
..................gCuuucguacguagcagagcagcuccc....................................................................    1    1    7y1
..................gguuucCuacguagcagagcagcuccc....................................................................    3    1    7y1
..................gguuucguacUuagcagagcagcuccc....................................................................    1    1    7y1
..................gUuuucguacguagcagagcagcuccc....................................................................    2    1    7y1
..................gguuGcguacguagcagagcagcuccc....................................................................    1    1    7y1
..................gguuucguacgGagcagagcagcuccc....................................................................    1    1    7y1
..................gguuucguacguagGagagcagcuccc....................................................................    1    1    7y1
..................ggGuucguacguagcagagcagcuccc....................................................................    3    1    7y1
..................gguuucgGacguagcagagcagcuccc....................................................................    2    1    7y1
..................gguuuAguacguagcagagcagcuccc....................................................................    15   1    7y1
..................gguuucguacgCagcagagcagcuccc....................................................................    1    1    7y1
..................ggAuucguacguagcagagcagcuccc....................................................................    3    1    7y1
..................gguuucguacguaCcagagcagcuccc....................................................................    3    1    7y1
..................gguuucguacguagAagagcagcuccc....................................................................    5    1    7y1
..................gguuucguaAguagcagagcagcuccc....................................................................    11   1    7y1
..................gguuucguacCuagcagagcagcuccc....................................................................    4    1    7y1
..................gguuucgAacguagcagagcagcuccc....................................................................    2    1    7y1
..................gguuucguaGguagcagagcagcuccc....................................................................    2    1    7y1
..................gguuucguacguCgcagagcagcucccu...................................................................    1    1    7y1
..................gguuucgCacguagcagagcagcucccu...................................................................    1    1    7y1
..................gguuucguacgAagcagagcagcucccu...................................................................    5    1    7y1
..................gguuuUguacguagcagagcagcucccu...................................................................    1    1    7y1
..................gguuucguacCuagcagagcagcucccu...................................................................    2    1    7y1
..................gCuuucguacguagcagagcagcucccu...................................................................    2    1    7y1
..................gguuucguacguGgcagagcagcucccu...................................................................    2    1    7y1
..................ggCuucguacguagcagagcagcucccu...................................................................    1    1    7y1
..................gAuuucguacguagcagagcagcucccu...................................................................    1    1    7y1
..................gguuucguacguaAcagagcagcucccu...................................................................    2    1    7y1
..................gguuucguacguagAagagcagcucccu...................................................................    5    1    7y1
..................gguuucgAacguagcagagcagcucccu...................................................................    7    1    7y1
..................gguuucguCcguagcagagcagcucccu...................................................................    2    1    7y1
..................gguuucguacguagGagagcagcucccu...................................................................    1    1    7y1
..................gguuucguacguaUcagagcagcucccu...................................................................    3    1    7y1
..................ggGuucguacguagcagagcagcucccu...................................................................    2    1    7y1
..................gguuucguGcguagcagagcagcucccu...................................................................    1    1    7y1
..................gguuucUuacguagcagagcagcucccu...................................................................    5    1    7y1
..................gguuucguaAguagcagagcagcucccu...................................................................    12   1    7y1
..................gguuuAguacguagcagagcagcucccu...................................................................    9    1    7y1
..................gguuucguaUguagcagagcagcucccu...................................................................    1    1    7y1
..................gguuucAuacguagcagagcagcucccu...................................................................    1    1    7y1
..................gUuuucguacguagcagagcagcucccu...................................................................    1    1    7y1
..................gguuAcguacguagcagagcagcucccu...................................................................    2    1    7y1
..................gguAucguacguagcagagcagcucccu...................................................................    3    1    7y1
..................ggAuucguacguagcagagcagcucccu...................................................................    4    1    7y1
..................gguuucguacUuagcagagcagcucccu...................................................................    1    1    7y1
..................gguuucguacguagAagagcagcucccuc..................................................................    1    1    7y1
..................gguuucCuacguagcagagcagcucccuc..................................................................    2    1    7y1
..................gguuucguacgAagcagagcagcucccuc..................................................................    1    1    7y1
..................gguuuAguacguagcagagcagcucccuc..................................................................    1    1    7y1
..................gguuucgAacguagcagagcagcucccuc..................................................................    2    1    7y1
..................gguuucUuacguagcagagcagcucccuc..................................................................    1    1    7y1
..................gguuAcguacguagcagagcagcucccuc..................................................................    1    1    7y1
..................ggAuucguacguagcagagcagcucccuc..................................................................    2    1    7y1
..................gguuucguacguaCcagagcagcucccuc..................................................................    1    1    7y1
..................gguuucguacUuagcagagcagcucccuc..................................................................    2    1    7y1
..................gguuucguacguaUcagagcagcucccuc..................................................................    1    1    7y1
..................gguuucguaGguagcagagcagcucccuc..................................................................    1    1    7y1
..................gguuucguGcguagcagagcagcucccuc..................................................................    1    1    7y1
..................gguuucgGacguagcagagcagcucccuc..................................................................    1    1    7y1
..................gguuuGguacguagcagagcagcucccuc..................................................................    1    1    7y1
..................gguuucguacguGgcagagcagcucccuc..................................................................    1    1    7y1
```

Star **Mature**

gaccugcuucugggucgggguuucguacguagcagagcagcucccucgcugcgaucuauugaaagucagcccucgacacaaggguuuguccgcgcgcgcgcgcgcgcgcgugc

..................gguuucguacAuagcagagcagcucccuc..	2	1	7y1
..................gCuuucguacguagcagagcagcucccuc..	1	1	7y1
..................gguuucguaAguagcagagcagcucccuc..	1	1	7y1
..................ggCuucguacguagcagagcagcucccuc..	1	1	7y1
..................gguuucguacguagAagagcagcucccucg...	2	1	7y1
..................gguuucguacguaCcagagcagcucccucg...	1	1	7y1
..................gguuucgGacguagcagagcagcucccucg...	1	1	7y1
..................gguuucguacguagUagagcagcucccucg...	1	1	7y1
..................gguuucCuacguagcagagcagcucccucg...	2	1	7y1
..................gguuucguacgAagcagagcagcucccucg...	3	1	7y1
..................gguuucguacguagGagagcagcucccucg...	1	1	7y1
..................gguuucguaAguagcagagcagcucccucg...	1	1	7y1
..................gguuucguaUguagcagagcagcucccucg...	2	1	7y1
..................gguuuAguacguagcagagcagcucccucg...	3	1	7y1
..................gguuAcguacguagcagagcagcucccucg...	1	1	7y1
..................gguAucguacguagcagagcagcucccucg...	2	1	7y1
..................ggAuucguacguagcagagcagcucccucg...	3	1	7y1
..................gguuucguUcguagcagagcagcucccucg...	1	1	7y1
..................gCuuucguacguagcagagcagcucccucg...	1	1	7y1
..................gguuucguacguaAcagagcagcucccucgc..	1	1	7y1
..................gguCucguacguagcagagcagcucccucgc..	1	1	7y1
..................gCuuucguacguagcagagcagcucccucgc..	1	1	7y1
..................gUuuucguacguagcagagcagcucccucgc..	2	1	7y1
..................gguuucguacUuagcagagcagcucccucgc..	1	1	7y1
..................gguuucgAacguagcagagcagcucccucgc..	4	1	7y1
..................gguuuAguacguagcagagcagcucccucgc..	1	1	7y1
..................gguuucAuacguagcagagcagcucccucgc..	1	1	7y1
..................gguuCcguacguagcagagcagcucccucgc..	1	1	7y1
..................ggGuucguacguagcagagcagcucccucgc..	2	1	7y1
..................gguuucguacguGgcagagcagcucccucgc..	1	1	7y1
..................gguuucguacguagUagagcagcucccucgc..	1	1	7y1
..................gguGucguacguagcagagcagcucccucgc..	1	1	7y1
..................gguuucguacgAagcagagcagcucccucgc..	2	1	7y1
..................gguuucguaAguagcagagcagcucccucgc..	5	1	7y1
..................ggAuucguacguagcagagcagcucccucgc..	2	1	7y1
..................gguAucguacguagcagagcagcucccucgc..	1	1	7y1
..................gguuucCuacguagcagagcagcucccucgc..	4	1	7y1
..................gguuucguacCuagcagagcagcucccucgcu...	1	1	7y1
..................gguuucguGcguagcagagcagcucccucgcu...	1	1	7y1
..................gguuucguacguagAagagcagcucccucgcu...	1	1	7y1
..................gguuucUuacguagcagagcagcucccucgcu...	1	1	7y1
..................gguuucguacguaUcagagcagcucccucgcu...	1	1	7y1
..................gUuuucguacguagcagagcagcucccucgcu...	1	1	7y1
..................gguuucAuacguagcagagcagcucccucgcu...	1	1	7y1
..................ggAuucguacguagcagagcagcucccucgcu...	1	1	7y1
..................gguuucguacguaCcagagcagcucccucgcu...	1	1	7y1
...................guuGcguacguagcagagc...	1	1	7y1
...................Uuuucguacguagcagagc...	1	1	7y1
...................guuucgAacguagcagagc...	3	1	7y1
...................guuucguaAguagcagagc...	5	1	7y1
...................Cuuucguacguagcagagc...	3	1	7y1
...................guuuAguacguagcagagc...	1	1	7y1
...................guuucUuacguagcagagc...	1	1	7y1
...................guuucCuacguagcagagc...	1	1	7y1
...................Nuuucguacguagcagagc...	1	1	7y1
...................guuAcguacguagcagagca..	1	1	7y1
...................guuCcguacguagcagagca..	1	1	7y1
...................guuuAguacguagcagagca..	1	1	7y1
...................guuucguacguagAagagca..	2	1	7y1
...................gAuucguacguagcagagca..	1	1	7y1
...................guuucguacCuagcagagca..	2	1	7y1
...................Cuuucguacguagcagagca..	2	1	7y1
...................Uuuucguacguagcagagca..	1	1	7y1
...................guuucgAacguagcagagca..	5	1	7y1
...................guAucguacguagcagagca..	1	1	7y1
...................guuucCuacguagcagagca..	2	1	7y1
...................guuucguacgAagcagagcagc..	1	1	7y1
...................guuucguaAguagcagagcagc..	2	1	7y1
...................gAuucguacguagcagagcagc..	5	1	7y1
...................guAucguacguagcagagcagc..	3	1	7y1

Star **Mature**

gaccugcuucugggucgggguuucguacguagcagagcagcucccucgcugcgaucuauugaaagucagcccucgacacaaggguuuguccgcgcgcgcgcgcgcgcgcgugc			
...................Uuuucguacguagcagagcagc..	286	1	7y1
...................guuucgAacguagcagagcagc..	2	1	7y1
...................Auuucguacguagcagagcagc..	1	1	7y1
...................guuucguUcguagcagagcagc..	1	1	7y1
...................Uuuucguacguagcagagcagcu...	3	1	7y1
...................guuucguacguGgcagagcagcu...	1	1	7y1
...................gGuucguacguagcagagcagcu...	1	1	7y1
...................guuAcguacguagcagagcagcu...	2	1	7y1
...................guuucguacguagAagagcagcu...	1	1	7y1
...................Cuuucguacguagcagagcagcu...	2	1	7y1
...................guuucgAacguagcagagcagcu...	3	1	7y1
...................guuucguacUuagcagagcagcu...	1	1	7y1
...................Nuuucguacguagcagagcagcu...	3	1	7y1
...................guuucUuacguagcagagcagcu...	1	1	7y1
...................guAucguacguagcagagcagcu...	4	1	7y1
...................gAuucguacguagcagagcagcu...	6	1	7y1
...................guuuAguacguagcagagcagcu...	1	1	7y1
...................guuucguacgAagcagagcagcu...	3	1	7y1
...................gAuucguacguagcagagcagcuc..	2	1	7y1
...................gGuucguacguagcagagcagcuc..	1	1	7y1
...................guAucguacguagcagagcagcuc..	3	1	7y1
...................guCucguacguagcagagcagcuc..	1	1	7y1
...................guuuUguacguagcagagcagcuc..	1	1	7y1
...................guuucguacgAagcagagcagcucc...	1	1	7y1
...................guuucguGcguagcagagcagcucc...	1	1	7y1
...................guAucguacguagcagagcagcucc...	1	1	7y1
...................Uuuucguacguagcagagcagcucc...	1	1	7y1
...................guuucguacguaAcagagcagcucc...	1	1	7y1
...................guuucguacguaUcagagcagcucc...	179	1	7y1
...................gAuucguacguagcagagcagcuccc..	4	1	7y1
...................guuAcguacguagcagagcagcuccc..	1	1	7y1
...................guuucgAacguagcagagcagcuccc..	2	1	7y1
...................guuucCuacguagcagagcagcuccc..	2	1	7y1
...................guuucguacguagAagagcagcuccc..	5	1	7y1
...................gCuucguacguagcagagcagcuccc..	2	1	7y1
...................guuucguacguCgcagagcagcuccc..	1	1	7y1
...................guuucguacguaAcagagcagcuccc..	2	1	7y1
...................guuucguacguaUcagagcagcuccc..	1	1	7y1
...................guuucguacgGagcagagcagcuccc..	1	1	7y1
...................guAucguacguagcagagcagcuccc..	2	1	7y1
...................Cuuucguacguagcagagcagcuccc..	2	1	7y1
...................guuuUguacguagcagagcagcuccc..	1	1	7y1
...................guuucguacgAagcagagcagcuccc..	1	1	7y1
...................guuucguacguaCcagagcagcuccc..	3	1	7y1
...................guuucguacCuagcagagcagcuccc..	3	1	7y1
...................guuucguaAguagcagagcagcuccc..	2	1	7y1
...................guuuAguacguagcagagcagcuccc..	3	1	7y1
...................guuucguacCuagcagagcagcucccu...	1	1	7y1
...................guuucgAacguagcagagcagcucccu...	7	1	7y1
...................gAuucguacguagcagagcagcucccu...	7	1	7y1
...................guuucguacgGagcagagcagcucccu...	1	1	7y1
...................guuucguacguagAagagcagcucccu...	3	1	7y1
...................guuuAguacguagcagagcagcucccu...	1	1	7y1
...................Uuuucguacguagcagagcagcucccu...	2	1	7y1
...................guuucguacAuagcagagcagcucccu...	2	1	7y1
...................guuucguacgCagcagagcagcucccu...	1	1	7y1
...................guuAcguacguagcagagcagcucccu...	2	1	7y1
...................guAucguacguagcagagcagcucccu...	2	1	7y1
...................gGuucguacguagcagagcagcucccu...	295	1	7y1
...................guuucguacguagcGgagcagcucccu...	2	1	7y1
...................guuucCuacguagcagagcagcucccu...	1	1	7y1
...................Cuuucguacguagcagagcagcucccu...	3	1	7y1
...................guuucguacguagcGgagcagcucccuc..	1	1	7y1
...................guuucguacAuagcagagcagcucccuc..	1	1	7y1
...................guuucguaAguagcagagcagcucccuc..	1	1	7y1
...................guuucguacUuagcagagcagcucccuc..	3	1	7y1
...................guuCcguacguagcagagcagcucccuc..	1	1	7y1
...................guuucguGcguagcagagcagcucccuc..	2	1	7y1
...................gGuucguacguagcagagcagcucccuc..	1	1	7y1
...................guuucgAacguagcagagcagcucccuc..	2	1	7y1

Star **Mature**

```
gaccugcuucugggucgggguuucguacguagcagagcagcucccucgcugcgaucuauugaaagucagcccucgacacaagguuuguccgcgcgcgcgcgcgcgcgcgugc
...................guuucguacCuagcagagcagcucccuc.................................................................   1     1   7y1
...................guAucguacguagcagagcagcucccuc.................................................................   1     1   7y1
...................Uuuucguacguagcagagcagcucccuc.................................................................   3     1   7y1
...................Cuuucguacguagcagagcagcucccuc.................................................................   1     1   7y1
...................guuucguacguagAagagcagcucccuc.................................................................   2     1   7y1
...................guuucguacgAagcagagcagcucccuc.................................................................   1     1   7y1
...................guuucguacguaUcagagcagcucccuc.................................................................   1     1   7y1
...................guuucgAacguagcagagcagcucccucg................................................................   4     1   7y1
...................guuucguacguagcGgagcagcucccucg................................................................   1     1   7y1
...................gAuucguacguagcagagcagcucccucg................................................................   1     1   7y1
...................guuucguacguaUcagagcagcucccucg................................................................   1     1   7y1
...................guAucguacguagcagagcagcucccucg................................................................   1     1   7y1
...................guuucguacAuagcagagcagcucccucg................................................................   1     1   7y1
...................guuucguacCuagcagagcagcucccucgc...............................................................   1     1   7y1
...................guuucguacgCagcagagcagcucccucgc...............................................................   1     1   7y1
...................guuucgAacguagcagagcagcucccucgc...............................................................   4     1   7y1
...................guuucgGacguagcagagcagcucccucgc...............................................................   1     1   7y1
...................gGuucguacguagcagagcagcucccucgc...............................................................   1     1   7y1
...................guuAcguacguagcagagcagcucccucgc...............................................................   2     1   7y1
...................guuucguacguaUcagagcagcucccucgc...............................................................   2     1   7y1
...................guuucguacUuagcagagcagcucccucgc...............................................................   2     1   7y1
...................guuucCuacguagcagagcagcucccucgc...............................................................   1     1   7y1
...................Nuuucguacguagcagagcagcucccucgc...............................................................   1     1   7y1
...................Cuuucguacguagcagagcagcucccucgc...............................................................   1     1   7y1
...................guAucguacguagcagagcagcucccucgc...............................................................   2     1   7y1
...................guuucUuacguagcagagcagcucccucgc...............................................................   1     1   7y1
...................Uuuucguacguagcagagcagcucccucgc...............................................................   1     1   7y1
...................guuucguacguagAagagcagcucccucgc...............................................................   1     1   7y1
...................guuucguaAguagcagagcagcucccucgc...............................................................   3     1   7y1
...................gAuucguacguagcagagcagcucccucgc...............................................................   1     1   7y1
...................guuucguacgAagcagagcagcucccucgc...............................................................   1     1   7y1
...................gAuucguacguagcagagcagcucccucgcu..............................................................   2     1   7y1
...................Cuuucguacguagcagagcagcucccucgcu..............................................................   2     1   7y1
...................guuucguacguagAagagcagcucccucgcu..............................................................   1     1   7y1
...................guuucguaGguagcagagcagcucccucgcu..............................................................   1     1   7y1
...................guuucUuacguagcagagcagcucccucgcu..............................................................   1     1   7y1
...................guuucguacguagAagagcagcucccucgcug.............................................................   1     1   7y1
...................guuucguacUuagcagagcagcucccucgcug.............................................................   1     1   7y1
...................gAuucguacguagcagagcagcucccucgcug.............................................................   1     1   7y1
....................uuucUuacguagcagagc..........................................................................   1     1   7y1
....................uuucAuacguagcagagc..........................................................................   1     1   7y1
....................uuucguacguagcUgagc..........................................................................   2     1   7y1
....................uuucguacgCagcagagc..........................................................................   1     1   7y1
....................uuucguacguagcagaUc..........................................................................   5     1   7y1
....................uuucgAacguagcagagc..........................................................................   8     1   7y1
....................Cuucguacguagcagagc..........................................................................   2     1   7y1
....................uuucguacCuagcagagc..........................................................................   4     1   7y1
....................Auucguacguagcagagc..........................................................................   52    1   7y1
....................uuucguacguagcaUagc..........................................................................   1     1   7y1
....................uuAcguacguagcagagc..........................................................................   2     1   7y1
....................uAucguacguagcagagc..........................................................................   9     1   7y1
....................uuuAguacguagcagagc..........................................................................   5     1   7y1
....................uuucguacguagcagaCc..........................................................................   2     1   7y1
....................uuucguacUuagcagagc..........................................................................   1     1   7y1
....................uuucguacguGgcagagc..........................................................................   2     1   7y1
....................uuucCuacguagcagagc..........................................................................   192   1   7y1
....................uuucguaUguagcagagc..........................................................................   1     1   7y1
....................uuucguacguagcagagA..........................................................................   7     1   7y1
....................uuucguacguagAagagc..........................................................................   4     1   7y1
....................uuucguacguaCcagagc..........................................................................   1     1   7y1
....................uuucguacgAagcagagc..........................................................................   11    1   7y1
....................uuucguacguagcGgagc..........................................................................   2     1   7y1
....................uuucgGacguagcagagc..........................................................................   1     1   7y1
....................Guucguacguagcagagc..........................................................................   9     1   7y1
....................uuuGguacguagcagagc..........................................................................   3     1   7y1
....................uuucguaAguagcagagc..........................................................................   8     1   7y1
....................uuucguacguaUcagagc..........................................................................   2     1   7y1
....................Nuucguacguagcagagc..........................................................................   3     1   7y1
....................uuucguacguagcagagc..........................................................................   4344  0   7y1
....................uuucguaGguagcagagca.........................................................................   1     1   7y1
```

Star **Mature**

gaccugcuucugggucgggguuucguacguagcagagcagcucccucgcugcgaucuauugaaagucagcccucgacacaaggguuuguccgcgcgcgcgcgcgcgcgcgugc			
....................uuucguacguaUcagagca..	2	1	7y1
....................uuucguacguagcagagAa..	4	1	7y1
....................uuucguacguagAagagca..	3	1	7y1
....................uuucguacgCagcagagca..	1	1	7y1
....................uuucguacCuagcagagca..	2	1	7y1
....................uCucguacguagcagagca..	1	1	7y1
....................uuucguacguagcagCgca..	3	1	7y1
....................uuucguacgGagcagagca..	1	1	7y1
....................uuucguacguagcGgagca..	1	1	7y1
....................uuucCuacguagcagagca..	1	1	7y1
....................uuuAguacguagcagagca..	2	1	7y1
....................uuucguaAguagcagagca..	2	1	7y1
....................Nuucguacguagcagagca..	3	1	7y1
....................uuucguCcguagcagagca..	1	1	7y1
....................Auucguacguagcagagca..	24	1	7y1
....................uuucguacguagcagaUca..	1	1	7y1
....................uuucguacguaCcagagca..	1	1	7y1
....................uuucguacUuagcagagca..	1	1	7y1
....................uuucguacguagcagagca..	1926	0	7y1
....................uuucguacguagcUgagca..	1	1	7y1
....................Guucguacguagcagagca..	4	1	7y1
....................uuucgCacguagcagagca..	1	1	7y1
....................uuucguGcguagcagagca..	1	1	7y1
....................uuucguacguUgcagagca..	2	1	7y1
....................uuucguacguagcagaCca..	3	1	7y1
....................uuucguacguagcagagGa..	1	1	7y1
....................uuucguacguagUagagca..	1	1	7y1
....................uuucgAacguagcagagca..	2	1	7y1
....................uAucguacguagcagagca..	5	1	7y1
....................uuucguacguagcaUagca..	1	1	7y1
....................uuucguacguagcaUagcag...	1	1	7y1
....................uuCcguacguagcagagcag...	1	1	7y1
....................uuucguacgAagcagagcag...	1	1	7y1
....................uuAcguacguagcagagcag...	1	1	7y1
....................uAucguacguagcagagcag...	4	1	7y1
....................uuucguacguagcagagAag...	1	1	7y1
....................uuucguacguagcGgagcag...	1	1	7y1
....................uuucguacguagcagaUcag...	1	1	7y1
....................uuucguacguagcagagcaU...	2	1	7y1
....................uuucguacguagcagagcaC...	2	1	7y1
....................Auucguacguagcagagcag...	11	1	7y1
....................uuucgCacguagcagagcag...	1	1	7y1
....................uuucguacguagcagagcag...	641	0	7y1
....................uuucguacguagcagagGagc..	2	1	7y1
....................uuucguacguagcGgagcagc..	1	1	7y1
....................uuucguacUuagcagagcagc..	1	1	7y1
....................uAucguacguagcagagcagc..	5	1	7y1
....................uuucguacguagUagagcagc..	1	1	7y1
....................uuucgAacguagcagagcagc..	3	1	7y1
....................uuucgGacguagcagagcagc..	3	1	7y1
....................Nuucguacguagcagagcagc..	1	1	7y1
....................uuucguacguagcagagcaCc..	1	1	7y1
....................uuucguGcguagcagagcagc..	1	1	7y1
....................uuucguaAguagcagagcagc..	5	1	7y1
....................uuucguaGguagcagagcagc..	1	1	7y1
....................uuucguacguagcagagcaAc..	1	1	7y1
....................uuucguaUguagcagagcagc..	1	1	7y1
....................uuucguacguagcagagAagc..	3	1	7y1
....................uuucguacguagcagagcaUc..	1	1	7y1
....................uuucguacguagAagagcagc..	2	1	7y1
....................uuucguacguagGagagcagc..	1	1	7y1
....................uuucguCcguagcagagcagc..	1	1	7y1
....................uuucguacguagcagagcagc..	2127	0	7y1
....................Cuucguacguagcagagcagc..	237	1	7y1
....................uuucguacguagcaUagcagc..	1	1	7y1
....................uuucguacguagcagagcagA..	1	1	7y1
....................uuuGguacguagcagagcagc..	2	1	7y1
....................uuucgCacguagcagagcagc..	1	1	7y1
....................Guucguacguagcagagcagc..	3	1	7y1
....................Auucguacguagcagagcagc..	18	1	7y1

Star **Mature**

gaccugcuucugggucgggguuucguacguagcagagcagcucccucgcugcgaucuauugaaagucagccccucgacacaaggguuuguccgcgcgcgcgcgcgcgcgcgugc

....................uuucguacguagcagCgcagc..	5	1	7y1
....................uuucguacgAagcagagcagc..	2	1	7y1
....................uuucguacguagcUgagcagcu...	1	1	7y1
....................uuucguacguagcagagcCgcu...	3	1	7y1
....................uuucguacAuagcagagcagcu...	1	1	7y1
....................uuucguacUuagcagagcagcu...	1	1	7y1
....................uuucguacguaCcagagcagcu...	1	1	7y1
....................uuuAguacguagcagagcagcu...	7	1	7y1
....................uuucguacguagcagGgcagcu...	1	1	7y1
....................Cuucguacguagcagagcagcu...	3	1	7y1
....................uuucguacguagAagagcagcu...	2	1	7y1
....................uuucguacguagcaUagcagcu...	272	1	7y1
....................uuucguacguagcagagcagcG...	22	1	7y1
....................Auucguacguagcagagcagcu...	37	1	7y1
....................uuucguacguagcagaCcagcu...	1	1	7y1
....................uuucgAacguagcagagcagcu...	4	1	7y1
....................uuucguacguagcagagcagAu...	1	1	7y1
....................Nuucguacguagcagagcagcu...	2	1	7y1
....................uuucguacguagcagagcagcA...	3	1	7y1
....................uuucguacCuagcagagcagcu...	4	1	7y1
....................uuucguacguagcagCgcagcu...	4	1	7y1
....................uuucguacguagcagagcagcu...	4088	0	7y1
....................uuucguacguagcagagcagcC...	1	1	7y1
....................uuucguacguagcagagGagcu...	2	1	7y1
....................uuucguacguagcagagcaUcu...	4	1	7y1
....................uAucguacguagcagagcagcu...	10	1	7y1
....................uuucguacguaAcagagcagcu...	1	1	7y1
....................uuucgCacguagcagagcagcu...	1	1	7y1
....................uuucguacguagcagagcaCcu...	1	1	7y1
....................uuucguGcguagcagagcagcu...	1	1	7y1
....................uuAcguacguagcagagcagcu...	3	1	7y1
....................uuucAuacguagcagagcagcu...	2	1	7y1
....................uuucguaUguagcagagcagcu...	1	1	7y1
....................uuucguacgAagcagagcagcu...	11	1	7y1
....................uuucguacguagcagagAagcu...	9	1	7y1
....................Guucguacguagcagagcagcu...	7	1	7y1
....................uuucguacguagUagagcagcu...	1	1	7y1
....................uuuUguacguagcagagcagcu...	2	1	7y1
....................uuucguacguagcagUgcagcu...	2	1	7y1
....................uuucguaAguagcagagcagcu...	8	1	7y1
....................uuucguacguagcaCagcagcu...	2	1	7y1
....................uuucCuacguagcagagcagcu...	2	1	7y1
....................uuucguacguagcaCagcagcuc..	1	1	7y1
....................uuucguacguagcagagcagGuc..	1	1	7y1
....................uuuAguacguagcagagcagcuc..	12	1	7y1
....................uuucCuacguagcagagcagcuc..	2	1	7y1
....................uuucguacCuagcagagcagcuc..	4	1	7y1
....................uuucguacguagcagagcagcGc..	1	1	7y1
....................uuucguacguagcagagcagcuc..	7935	0	7y1
....................uuucguacguagcagagcagcuG..	1	1	7y1
....................uuucguacguagcCgagcagcuc..	1	1	7y1
....................uuucAuacguagcagagcagcuc..	1	1	7y1
....................uuucguacguagcagagAagcuc..	10	1	7y1
....................uuucguacguagcagagcUgcuc..	1	1	7y1
....................uuucguacAuagcagagcagcuc..	1	1	7y1
....................uuucUuacguagcagagcagcuc..	6	1	7y1
....................Guucguacguagcagagcagcuc..	13	1	7y1
....................uuucguacguagcUgagcagcuc..	1	1	7y1
....................uuucguacguagcagUgcagcuc..	1	1	7y1
....................uuucguaAguagcagagcagcuc..	9	1	7y1
....................uuucguacguagcagagcagcAc..	2	1	7y1
....................uuCcguacguagcagagcagcuc..	1	1	7y1
....................uuucgAacguagcagagcagcuc..	16	1	7y1
....................uuucguacguagcagagcaUcuc..	7	1	7y1
....................uuucguacguagcagagcCgcuc..	3	1	7y1
....................uuucguacguGgcagagcagcuc..	2	1	7y1
....................uuucguacguagcGgagcagcuc..	1	1	7y1
....................uuucguacguagcagagcagcuA..	8	1	7y1
....................uuucguacguagcagagcagcuU..	1	1	7y1
....................uuucgCacguagcagagcagcuc..	1	1	7y1

Star **Mature**

```
gaccugcuucugggucggggguucguacguagcagagcagcucccucgcugcgaucuauugaaagucagcccucgacacaaggguuuguccgcgcgcgcgcgcgcgcgcgugc
....................uuucguacguagcagaUcagcuc......................................................................  3     1  7y1
....................uuucguacgAagcagagcagcuc......................................................................  11    1  7y1
....................uuucguacUuagcagagcagcuc......................................................................  3     1  7y1
....................uuucguacguagcagaCcagcuc......................................................................  4     1  7y1
....................uuucguaUguagcagagcagcuc......................................................................  1     1  7y1
....................uuucguacguagcagCgcagcuc......................................................................  7     1  7y1
....................uuuGguacguagcagagcagcuc......................................................................  3     1  7y1
....................uuucguCcguagcagagcagcuc......................................................................  2     1  7y1
....................Auucguacguagcagagcagcuc......................................................................  89    1  7y1
....................uuucguacguagcagagcGgcuc......................................................................  2     1  7y1
....................uuucguacguagcagagcagAuc......................................................................  5     1  7y1
....................uuucguacguagcagagcaCcuc......................................................................  4     1  7y1
....................uuuUguacguagcagagcagcuc......................................................................  1     1  7y1
....................Nuucguacguagcagagcagcuc......................................................................  4     1  7y1
....................uuucguacguaCcagagcagcuc......................................................................  2     1  7y1
....................uAucguacguagcagagcagcuc......................................................................  11    1  7y1
....................uuucguacguaUcagagcagcuc......................................................................  1     1  7y1
....................uuucguacguagcagagGagcuc......................................................................  1     1  7y1
....................Cuucguacguagcagagcagcuc......................................................................  175   1  7y1
....................uuucguGcguagcagagcagcuc......................................................................  1     1  7y1
....................uuucguacgCagcagagcagcuc......................................................................  1     1  7y1
....................uuucguacguagUagagcagcuc......................................................................  2     1  7y1
....................uuucguacguagcaUagcagcuc......................................................................  2     1  7y1
....................uuAcguacguagcagagcagcuc......................................................................  3     1  7y1
....................uuucguacguagcagGgcagcuc......................................................................  1     1  7y1
....................uCucguacguagcagagcagcuc......................................................................  1     1  7y1
....................uuucguacguagAagagcagcuc......................................................................  9     1  7y1
....................uuGcguacguagcagagcagcuc......................................................................  1     1  7y1
....................uuucguacguagcagagcagcucG.....................................................................  1     1  7y1
....................uuucguacgAagcagagcagcucc.....................................................................  7     1  7y1
....................uuucguacguagcagGgcagcucc.....................................................................  4     1  7y1
....................uuucguacgGagcagagcagcucc.....................................................................  1     1  7y1
....................uuucguacguagcagagcagcuUc.....................................................................  1     1  7y1
....................uuucguaAguagcagagcagcucc.....................................................................  7     1  7y1
....................uuucguacguagcagagcagcAcc.....................................................................  1     1  7y1
....................uuucguacguagcaUagcagcucc.....................................................................  1     1  7y1
....................uuucguacCuagcagagcagcucc.....................................................................  4     1  7y1
....................uuucguacguagcaCagcagcucc.....................................................................  1     1  7y1
....................uuucUuacguagcagagcagcucc.....................................................................  2     1  7y1
....................uuucgCacguagcagagcagcucc.....................................................................  2     1  7y1
....................uuAcguacguagcagagcagcucc.....................................................................  1     1  7y1
....................uuCcguacguagcagagcagcucc.....................................................................  2     1  7y1
....................uuucguacguaUcagagcagcucc.....................................................................  3     1  7y1
....................uuucguacguagcagagcCgcucc.....................................................................  1     1  7y1
....................uuucguacguagcagagcaUcucc.....................................................................  3     1  7y1
....................uuucCuacguagcagagcagcucc.....................................................................  1     1  7y1
....................uuGcguacguagcagagcagcucc.....................................................................  1     1  7y1
....................Guucguacguagcagagcagcucc.....................................................................  14    1  7y1
....................uuucgAacguagcagagcagcucc.....................................................................  11    1  7y1
....................uuucguacguagcagagcagAucc.....................................................................  4     1  7y1
....................uuucguacguagcagagcaCcucc.....................................................................  3     1  7y1
....................uuucguacguUgcagagcagcucc.....................................................................  1     1  7y1
....................uuucguacguagcagagcagcucc.....................................................................  5981  0  7y1
....................uAucguacguagcagagcagcucc.....................................................................  9     1  7y1
....................uuucguacguagcagaCcagcucc.....................................................................  3     1  7y1
....................uuucguacguagcagagcagcucU.....................................................................  2     1  7y1
....................uuucguacgCagcagagcagcucc.....................................................................  1     1  7y1
....................Auucguacguagcagagcagcucc.....................................................................  67    1  7y1
....................uuucguacguagcagagcGgcucc.....................................................................  1     1  7y1
....................uuucguacguagcagagcagcCcc.....................................................................  2     1  7y1
....................Nuucguacguagcagagcagcucc.....................................................................  3     1  7y1
....................uuucguacguagcGgagcagcucc.....................................................................  1     1  7y1
....................uuucguacguagcagagAagcucc.....................................................................  7     1  7y1
....................uuucguaGguagcagagcagcucc.....................................................................  2     1  7y1
....................uuuGguacguagcagagcagcucc.....................................................................  2     1  7y1
....................uuucguaUguagcagagcagcucc.....................................................................  1     1  7y1
....................uuucguacUuagcagagcagcucc.....................................................................  2     1  7y1
....................uuucguacguagcagagcagcuAc.....................................................................  15    1  7y1
....................Cuucguacguagcagagcagcucc.....................................................................  1     1  7y1
....................uuucguUcguagcagagcagcucc.....................................................................  2     1  7y1
```

```
  Star                                                                Mature
gaccugcuucugggucggggguuucguacguagcagagcagcucccucgcugcgaucuauugaaagucagcccucgacacaaggguuuguccgcgcgcgcgcgcgcgcgcgugc
....................uuuAguacguagcagagcagcucc.....................................................................	6	1	7y1
....................uNucguacguagcagagcagcucc.....................................................................	1	1	7y1
....................uuucguacguagAagagcagcucc.....................................................................	9	1	7y1
....................uuucguacguagcagaUcagcucc.....................................................................	1	1	7y1
....................uuucguacguagcagCgcagcucc.....................................................................	5	1	7y1
....................uuuUguacguagcagagcagcucc.....................................................................	1	1	7y1
....................uuucguacguagcagagcaAcucc.....................................................................	1	1	7y1
....................uuucguacguagcagUgcagcucc.....................................................................	3	1	7y1
....................uuucguacguagcagagcagcucA.....................................................................	2	1	7y1
....................uuucguacguagcagagcagAuccc....................................................................	1	1	7y1
....................Auucguacguagcagagcagcuccc....................................................................	17	1	7y1
....................uuucUuacguagcagagcagcuccc....................................................................	1	1	7y1
....................uuucguacguagcagagcagGuccc....................................................................	1	1	7y1
....................uuucguacguagcagCgcagcuccc....................................................................	4	1	7y1
....................uuucguacCuagcagagcagcuccc....................................................................	1	1	7y1
....................uuucguacguagcagUgcagcuccc....................................................................	2	1	7y1
....................uuucguacguagcagagcaUcuccc....................................................................	3	1	7y1
....................uuucCuacguagcagagcagcuccc....................................................................	1	1	7y1
....................uAucguacguagcagagcagcuccc....................................................................	3	1	7y1
....................uuucguacguagcagagcagcuccA....................................................................	4	1	7y1
....................uuucguacguagUagagcagcuccc....................................................................	277	1	7y1
....................uuuGguacguagcagagcagcuccc....................................................................	3	1	7y1
....................uuucguacguagcagagcagcuccG....................................................................	1	1	7y1
....................uuucguacUuagcagagcagcuccc....................................................................	1	1	7y1
....................uuucgCacguagcagagcagcuccc....................................................................	1	1	7y1
....................uuucguacguagcagagcagcuAcc....................................................................	11	1	7y1
....................uuucguacguagcagagAagcuccc....................................................................	2	1	7y1
....................uuucguacguagcagagcaCcuccc....................................................................	2	1	7y1
....................Guucguacguagcagagcagcuccc....................................................................	2	1	7y1
....................uuucguaGguagcagagcagcuccc....................................................................	1	1	7y1
....................uuucguacguagAagagcagcuccc....................................................................	2	1	7y1
....................uuucguacguagcagagcagcuccc....................................................................	2072	0	7y1
....................uuucguacguagcagaCcagcuccc....................................................................	1	1	7y1
....................uuucguacgAagcagagcagcuccc....................................................................	3	1	7y1
....................uuucguacguagcagagcagcAccc....................................................................	1	1	7y1
....................uuucguacguagcGgagcagcuccc....................................................................	1	1	7y1
....................uuucguacguaUcagagcagcuccc....................................................................	1	1	7y1
....................uuucguaAguagcagagcagcuccc....................................................................	3	1	7y1
....................uuuAguacguagcagagcagcuccc....................................................................	2	1	7y1
....................uuucAuacguagcagagcagcuccc....................................................................	1	1	7y1
....................uuAcguacguagcagagcagcuccc....................................................................	2	1	7y1
....................uuucguacguagGagagcagcuccc....................................................................	1	1	7y1
....................uuucgAacguagcagagcagcuccc....................................................................	4	1	7y1
....................uuucguacguaCcagagcagcucccu...................................................................	1	1	7y1
....................uuucguacguagcagGgcagcucccu...................................................................	2	1	7y1
....................uuucguacguagcaUagcagcucccu...................................................................	1	1	7y1
....................uuucguacguagcagagcagAucccu...................................................................	2	1	7y1
....................uuucguacguagcagagcagcucccu...................................................................	4551	0	7y1
....................uuucguacgAagcagagcagcucccu...................................................................	7	1	7y1
....................uuAcguacguagcagagcagcucccu...................................................................	4	1	7y1
....................uuucguacguagcagagcagcAcccu...................................................................	1	1	7y1
....................uuucguGcguagcagagcagcucccu...................................................................	1	1	7y1
....................uuucguacguagcagagcagcCcccu...................................................................	1	1	7y1
....................uuucguacguagcagagcCgcucccu...................................................................	1	1	7y1
....................uuucguacAuagcagagcagcucccu...................................................................	3	1	7y1
....................uuucUuacguagcagagcagcucccu...................................................................	3	1	7y1
....................Guucguacguagcagagcagcucccu...................................................................	14	1	7y1
....................uuucguacguagcagaUcagcucccu...................................................................	1	1	7y1
....................uuucguacguagcaAagcagcucccu...................................................................	1	1	7y1
....................uuucguaUguagcagagcagcucccu...................................................................	2	1	7y1
....................uuucguacguagcagagcagcuccAu...................................................................	4	1	7y1
....................uAucguacguagcagagcagcucccu...................................................................	9	1	7y1
....................uuucguacguagcagagcagcucccG...................................................................	17	1	7y1
....................uuucgAacguagcagagcagcucccu...................................................................	9	1	7y1
....................uuucguacguagcagagcagcucccC...................................................................	152	1	7y1
....................uuucguacguagcagagcGgcucccu...................................................................	1	1	7y1
....................uuGcguacguagcagagcagcucccu...................................................................	1	1	7y1
....................uuucguacguagcagagcagcucccA...................................................................	8	1	7y1
....................uCucguacguagcagagcagcucccu...................................................................	1	1	7y1
....................uuucguacguagcUgagcagcucccu...................................................................	1	1	7y1
```

Star **Mature**

gaccugcuucugggucggggguuucguacguagcagagcagcucccucgcugcgaucuauugaaagucagcccucgacacaaggguuuguccgcgcgcgcgcgcgcgcgcgugc

....................uuucguacguagcGgagcagcucccu...	1	1	7y1
....................uuucguacguagcagagAagcucccu...	6	1	7y1
....................uuucguaAguagcagagcagcucccu...	4	1	7y1
....................uuucguacguagUagagcagcucccu...	225	1	7y1
....................uuucguacguagcagagcagcucAcu...	3	1	7y1
....................uuucguacguagAagagcagcucccu...	2	1	7y1
....................Nuucguacguagcagagcagcucccu...	1	1	7y1
....................uuucguacguagcagCgcagcucccu...	10	1	7y1
....................Cuucguacguagcagagcagcucccu...	2	1	7y1
....................uGucguacguagcagagcagcucccu...	1	1	7y1
....................uuucguacUuagcagagcagcucccu...	7	1	7y1
....................uuucguacguagcagaCcagcucccu...	2	1	7y1
....................uuucguacguaAcagagcagcucccu...	2	1	7y1
....................uuucCuacguagcagagcagcucccu...	1	1	7y1
....................uuucguacCuagcagagcagcucccu...	1	1	7y1
....................uuucguacguGgcagagcagcucccu...	2	1	7y1
....................Auucguacguagcagagcagcucccu...	42	1	7y1
....................uuucguacguaUcagagcagcucccu...	1	1	7y1
....................uuucguacguagcagagcagcuAccu...	10	1	7y1
....................uuuAguacguagcagagcagcucccu...	6	1	7y1
....................Auucguacguagcagagcagcucccuc..	39	1	7y1
....................uuAcguacguagcagagcagcucccuc..	3	1	7y1
....................uuucCuacguagcagagcagcucccuc..	2	1	7y1
....................uuucgAacguagcagagcagcucccuc..	9	1	7y1
....................uuucguacgAagcagagcagcucccuc..	7	1	7y1
....................uuuGguacguagcagagcagcucccuc..	1	1	7y1
....................uuucguaAguagcagagcagcucccuc..	3	1	7y1
....................uuucguacguagcagagcagcucccuA..	3	1	7y1
....................uuucguacguagcagagAagcucccuc..	6	1	7y1
....................uuucguacguagcagagcaCcucccuc..	1	1	7y1
....................Cuucguacguagcagagcagcucccuc..	1	1	7y1
....................uuucguacguagcagagGagcucccuc..	1	1	7y1
....................uuucguacguagcagCgcagcucccuc..	10	1	7y1
....................uuucguacguagcagagcagcuAccuc..	7	1	7y1
....................uuucguacguagcagagcCgcucccuc..	1	1	7y1
....................Nuucguacguagcagagcagcucccuc..	6	1	7y1
....................uuucguacguagcagagcagcCcccuc..	1	1	7y1
....................uuucguacguaAcagagcagcucccuc..	1	1	7y1
....................uuucguacguagcagUgcagcucccuc..	2	1	7y1
....................uuucguacguagcagagcaUcucccuc..	2	1	7y1
....................uuucguacguagcagagcagcuccAuc..	5	1	7y1
....................uuucguacguagcagagcagcucUcuc..	1	1	7y1
....................uuucguacguagcaUagcagcucccuc..	2	1	7y1
....................uuucgCacguagcagagcagcucccuc..	1	1	7y1
....................uuucguacguagcaCagcagcucccuc..	2	1	7y1
....................uuucguacguagcagagcGgcucccuc..	2	1	7y1
....................uuucguaUguagcagagcagcucccuc..	1	1	7y1
....................uuucguacguagcagagcagcuGccuc..	1	1	7y1
....................uuucguacguagcagagcagcucccAc..	4	1	7y1
....................uuucguacguagcagagcagcucAcuc..	6	1	7y1
....................uuucguacguagcagagcagcAcccuc..	2	1	7y1
....................uuuAguacguagcagagcagcucccuc..	11	1	7y1
....................uuucguacguagcagagcagcuUccuc..	1	1	7y1
....................uuucguacguaUcagagcagcucccuc..	1	1	7y1
....................uuucguacguagcagagcagcucGcuc..	2	1	7y1
....................uuucguacCuagcagagcagcucccuc..	1	1	7y1
....................uuucguacguagcagagcagAucccuc..	4	1	7y1
....................uuucguacguagcagagcagcucccuc..	4379	0	7y1
....................uuucUuacguagcagagcagcucccuc..	2	1	7y1
....................uuucguacguagcagagcUgcucccuc..	1	1	7y1
....................uuucguacguagAagagcagcucccuc..	5	1	7y1
....................uAucguacguagcagagcagcucccuc..	9	1	7y1
....................uuucguacguagUagagcagcucccuc..	1	1	7y1
....................Guucguacguagcagagcagcucccuc..	12	1	7y1
....................uuucguacUuagcagagcagcucccuc..	2	1	7y1
....................uuucguacguagcagagAagcucccucgc..	1	1	7y1
....................uuuGguacguagcagagcagcucccucgc..	1	1	7y1
....................uuucguacguagcagagcagcucccAcgc..	1	1	7y1
....................uuucguacguagcagagcUgcucccucgc..	1	1	7y1
....................Guucguacguagcagagcagcucccucgc..	2	1	7y1

Star **Mature**

gaccugcuucugggucgggguuucguacguagcagagcagcucccucgcugcgaucuauugaaagucagcccucgacacaaggguuuguccgcgcgcgcgcgcgcgcgcgugc

....................uuucguacguagcagagcaCcucccucgc..	1	1	7y1
....................uuucguacguagcagagcagcucccucgc..	614	0	7y1
....................uuucguacgAagcagagcagcucccucgc..	1	1	7y1
....................uuAcguacguagcagagcagcucccucgc..	1	1	7y1
....................uAucguacguagcagagcagcucccucgc..	1	1	7y1
....................uuucguacguagcagCgcagcucccucgc..	1	1	7y1
....................uuucgAacguagcagagcagcucccucgc..	1	1	7y1
....................uuuAguacguagcagagcagcucccucgc..	1	1	7y1
....................uuucguacguagUagagcagcucccucgc..	1	1	7y1
....................uuucguacguagcagagcagcucAcucgc..	1	1	7y1
....................uuucguacCuagcagagcagcucccucgc..	1	1	7y1
....................uuucguacguagcagaUcagcucccucgc..	1	1	7y1
....................uuucguacguagcagagcagcuAccucgc..	1	1	7y1
....................Auucguacguagcagagcagcucccucgc..	4	1	7y1
....................uuucguacguagcagGgcagcucccucgcu...	2	1	7y1
....................uuucguacguGgcagagcagcucccucgcu...	1	1	7y1
....................uuucgAacguagcagagcagcucccucgcu...	4	1	7y1
....................uuucguacguagcagagcagcucccucgcu...	2277	0	7y1
....................uuuAguacguagcagagcagcucccucgcu...	4	1	7y1
....................uuucguacguagGagagcagcucccucgcu...	1	1	7y1
....................uuucguacguagcagagcagcucccuGgcu...	1	1	7y1
....................uuucguaAguagcagagcagcucccucgcu...	2	1	7y1
....................uuucguacguagcagaCcagcucccucgcu...	1	1	7y1
....................uuucguacguaCcagagcagcucccucgcu...	1	1	7y1
....................Guucguacguagcagagcagcucccucgcu...	178	1	7y1
....................uuucguacguagcagagcagcCcccucgcu...	1	1	7y1
....................uuucguacguagAagagcagcucccucgcu...	3	1	7y1
....................uuucguacCuagcagagcagcucccucgcu...	1	1	7y1
....................uuucguacguagcCgagcagcucccucgcu...	1	1	7y1
....................uAucguacguagcagagcagcucccucgcu...	1	1	7y1
....................uuucguacguagcagagcagcucGcucgcu...	1	1	7y1
....................uuucguacguagcagagcCgcucccucgcu...	2	1	7y1
....................Auucguacguagcagagcagcucccucgcu...	20	1	7y1
....................uuGcguacguagcagagcagcucccucgcu...	1	1	7y1
....................uuAcguacguagcagagcagcucccucgcu...	1	1	7y1
....................uuucguacguagcagCgcagcucccucgcu...	2	1	7y1
....................uuucguacgCagcagagcagcucccucgcu...	1	1	7y1
....................uuucguacgAagcagagcagcucccucgcu...	6	1	7y1
....................uuucguacguagcagagcUgcucccucgcu...	1	1	7y1
....................uuucguacguagcagagcagcucUcucgcu...	2	1	7y1
....................uuucguUcguagcagagcagcucccucgcu...	1	1	7y1
....................uuucguacguagcagagAagcucccucgcu...	2	1	7y1
....................uuucguacguagcaCagcagcucccucgcu...	2	1	7y1
....................uuucguacUuagcagagcagcucccucgcu...	1	1	7y1
....................uuucguacguagcagagcagUucccucgcu...	1	1	7y1
....................uuucguacguagcagagcGgcucccucgcu...	1	1	7y1
....................uuucguacguagcagagcagcucccucAcu...	2	1	7y1
....................uuucAuacguagcagagcagcucccucgcu...	2	1	7y1
....................uuucguacguagcagagcaUcucccucgcu...	1	1	7y1
....................uuucguacguagcagUgcagcucccucgcu...	1	1	7y1
....................uuucguacguagcagagcagcucccucgAu...	3	1	7y1
....................uuucguacguagcagagcagcucccucgcG...	20	1	7y1
....................uuucguacguagcagaUcagcucccucgcu...	1	1	7y1
....................Nuucguacguagcagagcagcucccucgcu...	2	1	7y1
....................uuucguacguagcagagcagcucccAcgcu...	2	1	7y1
....................uuucguacguagcagagcagcuAccucgcu...	7	1	7y1
....................uuucguacguagcagagcagcucccuAgcu...	3	1	7y1
....................uuucguacguagcagagcagcucAcucgcu...	1	1	7y1
....................uuucguacCuagcagagcagcucccucgcug..	1	1	7y1
....................uuucAuacguagcagagcagcucccucgcug..	1	1	7y1
....................uuucguacguagcUgagcagcucccucgcug..	1	1	7y1
....................uuucguacguagcagagcGgcucccucgcug..	1	1	7y1
....................uuucguacguagcagagcagcuAccucgcug..	2	1	7y1
....................uuucguacguagcagagAagcucccucgcug..	2	1	7y1
....................uuucguacguagcagagcagcucccucCcug..	1	1	7y1
....................uAucguacguagcagagcagcucccucgcug..	2	1	7y1
....................uuucguacguagcagagcagcucccucgcGg..	9	1	7y1
....................Auucguacguagcagagcagcucccucgcug..	6	1	7y1
....................uuucguCcguagcagagcagcucccucgcug..	1	1	7y1
....................uuucguacguagcagagcagcucccucgGug..	1	1	7y1

Star **Mature**

```
gaccugcuucugggucggggguuucguacguagcagagcagcucccucgcugcgaucuauugaaagucagcccucgacacaaggguuuguccgcgcgcgcgcgcgcgcgcgcgugc
....................uuucguacguagcagagcagcucccAcgcug..............................................................  1     1  7y1
....................uuucguUcguagcagagcagcucccucgcug..............................................................  1     1  7y1
....................uuucgAacguagcagagcagcucccucgcug..............................................................  2     1  7y1
....................uuucguacguagcagagcagcucccucgcug..............................................................  855   0  7y1
....................uuucguacguagAagagcagcucccucgcug..............................................................  1     1  7y1
....................uuucguacguagcagagcagcucccucUcug..............................................................  1     1  7y1
....................uuucguacgAagcagagcagcucccucgcug..............................................................  1     1  7y1
....................uuucguacguagcagagcaAcucccucgcug..............................................................  1     1  7y1
....................uuucguaAguagcagagcagcucccucgcug..............................................................  1     1  7y1
....................uuucUuacguagcagagcagcucccucgcug..............................................................  1     1  7y1
....................Auucguacguagcagagcagcucccucgcugc.............................................................  5     1  7y1
....................uuucguacgAagcagagcagcucccucgcugc.............................................................  1     1  7y1
....................uAucguacguagcagagcagcucccucgcugc.............................................................  1     1  7y1
....................uuuAguacguagcagagcagcucccucgcugc.............................................................  1     1  7y1
....................uuucguacguagcagagcagcuAccucgcugc.............................................................  1     1  7y1
....................uuucguacguagcagagcagcAcccucgcugc.............................................................  1     1  7y1
....................uuucguacCuagcagagcagcucccucgcugc.............................................................  1     1  7y1
....................Guucguacguagcagagcagcucccucgcugc.............................................................  1     1  7y1
....................Nuucguacguagcagagcagcucccucgcugc.............................................................  2     1  7y1
....................uuucguacguagcagagcagcucccucgcugc.............................................................  499   0  7y1
....................uuucguacguagcagagcagAucccucgcugc.............................................................  1     1  7y1
....................uuucguacguagcagagcagcucccucgAugc.............................................................  1     1  7y1
....................uuucguacguagcagagcagcucccucgcuCc.............................................................  1     1  7y1
.....................uucguacguagcaUagca..........................................................................  1     1  7y1
.....................uucguacguagcaCagca..........................................................................  1     1  7y1
.....................uucguacguagcagagAa..........................................................................  3     1  7y1
.....................uucguacguaCcagagca..........................................................................  1     1  7y1
.....................uucguacguaAcagagca..........................................................................  1     1  7y1
.....................uucguacguagcagGgca..........................................................................  1     1  7y1
.....................uucguacguagcagagGa..........................................................................  1     1  7y1
.....................uucguacguagAagagca..........................................................................  1     1  7y1
.....................uucguacgAagcagagca..........................................................................  3     1  7y1
.....................uucAuacguagcagagca..........................................................................  1     1  7y1
.....................uAcguacguagcagagca..........................................................................  4     1  7y1
.....................Nucguacguagcagagca..........................................................................  1     1  7y1
.....................uucguaAguagcagagca..........................................................................  8     1  7y1
.....................Gucguacguagcagagca..........................................................................  2     1  7y1
.....................uuAguacguagcagagca..........................................................................  4     1  7y1
.....................uucguacguagcagaAca..........................................................................  1     1  7y1
.....................uucguacguagcagagcG..........................................................................  1     1  7y1
.....................uNcguacguagcagagca..........................................................................  1     1  7y1
.....................Aucguacguagcagagca..........................................................................  12    1  7y1
.....................uucguacCuagcagagca..........................................................................  2     1  7y1
.....................uucguacguagcagagca..........................................................................  1212  0  7y1
.....................Cucguacguagcagagca..........................................................................  1     1  7y1
.....................uucguacguagcagagcC..........................................................................  1     1  7y1
.....................uucguacguagcagaUca..........................................................................  2     1  7y1
.....................uucguacAuagcagagcag.........................................................................  1     1  7y1
.....................uucguacguagcaCagcag.........................................................................  1     1  7y1
.....................Aucguacguagcagagcag.........................................................................  16    1  7y1
.....................uAcguacguagcagagcag.........................................................................  3     1  7y1
.....................Gucguacguagcagagcag.........................................................................  2     1  7y1
.....................uucguacguagcagagcag.........................................................................  1378  0  7y1
.....................uucguacguagAagagcag.........................................................................  1     1  7y1
.....................uucguaAguagcagagcag.........................................................................  4     1  7y1
.....................uucgCacguagcagagcag.........................................................................  1     1  7y1
.....................uucguacgAagcagagcag.........................................................................  2     1  7y1
.....................uucguacguagcagagAag.........................................................................  1     1  7y1
.....................uucguacguGgcagagcag.........................................................................  1     1  7y1
.....................uucguacguagcagagcaU.........................................................................  2     1  7y1
.....................uucguacCuagcagagcag.........................................................................  1     1  7y1
.....................uucguacguagcagagcUg.........................................................................  1     1  7y1
.....................uucguacguaUcagagcag.........................................................................  1     1  7y1
.....................uuAguacguagcagagcag.........................................................................  4     1  7y1
.....................uucguUcguagcagagcagc........................................................................  2     1  7y1
.....................uucguacgAagcagagcagc........................................................................  1     1  7y1
.....................uucguacAuagcagagcagc........................................................................  1     1  7y1
.....................Nucguacguagcagagcagc........................................................................  1     1  7y1
.....................uucguaGguagcagagcagc........................................................................  2     1  7y1
.....................uAcguacguagcagagcagc........................................................................  2     1  7y1
```

Star **Mature**

```
gaccugcuucugggucggggu uucguacguagcagagcagcucccucgcugcgaucuauugaaagucagcc cucgacacaaggguuugu ccgcgcgcgcgcgcgcgcgcgugc
.....................uucguacguagcaCagcagc........................................................................  1     1   7y1
.....................uucCuacguagcagagcagc........................................................................  1     1   7y1
.....................uucguacCuagcagagcagc........................................................................  1     1   7y1
.....................uucguacguagcagagcaUc........................................................................  3     1   7y1
.....................uucguacguagcagaUcagc........................................................................  1     1   7y1
.....................uucguacguagcagaCcagc........................................................................  1     1   7y1
.....................Aucguacguagcagagcagc........................................................................  33    1   7y1
.....................uucguacguGgcagagcagc........................................................................  2     1   7y1
.....................uucguacguagcagagcagA........................................................................  2     1   7y1
.....................uuUguacguagcagagcagc........................................................................  1     1   7y1
.....................uucguacguagcaUagcagc........................................................................  1     1   7y1
.....................uucgAacguagcagagcagc........................................................................  1     1   7y1
.....................uucguacguagcUgagcagc........................................................................  1     1   7y1
.....................uucguaAguagcagagcagc........................................................................  2     1   7y1
.....................uucguacUuagcagagcagc........................................................................  2     1   7y1
.....................uucguacguaAcagagcagc........................................................................  1     1   7y1
.....................uCcguacguagcagagcagc........................................................................  1     1   7y1
.....................uucguacgCagcagagcagc........................................................................  1     1   7y1
.....................uucguacguagcaAagcagc........................................................................  1     1   7y1
.....................uucguacguagcagagcagc........................................................................  1790  0   7y1
.....................uucguacguagcagGgcagc........................................................................  1     1   7y1
.....................uuAguacguagcagagcagc........................................................................  5     1   7y1
.....................uucguacguagcagagcGgcu.......................................................................  5     1   7y1
.....................uucguacguagcagagAagcu.......................................................................  9     1   7y1
.....................uucguacguagcaUagcagcu.......................................................................  5     1   7y1
.....................uucguacguagUagagcagcu.......................................................................  185   1   7y1
.....................uucguacguagcagagcagcA.......................................................................  4     1   7y1
.....................uucguacguagcagagcagAu.......................................................................  4     1   7y1
.....................uucguacguagcGgagcagcu.......................................................................  1     1   7y1
.....................Nucguacguagcagagcagcu.......................................................................  2     1   7y1
.....................uucguGcguagcagagcagcu.......................................................................  1     1   7y1
.....................uucguacguaUcagagcagcu.......................................................................  3     1   7y1
.....................uucguacguagcUgagcagcu.......................................................................  2     1   7y1
.....................uucguacguagcagagcUgcu.......................................................................  1     1   7y1
.....................uucCuacguagcagagcagcu.......................................................................  1     1   7y1
.....................uucguacgAagcagagcagcu.......................................................................  8     1   7y1
.....................uucUuacguagcagagcagcu.......................................................................  5     1   7y1
.....................uucguacguagcagagcaUcu.......................................................................  3     1   7y1
.....................uucguacguagcagagcaAcu.......................................................................  1     1   7y1
.....................uucguacguagcagaCcagcu.......................................................................  5     1   7y1
.....................uuGguacguagcagagcagcu.......................................................................  2     1   7y1
.....................uucguacguagcagagcagGu.......................................................................  2     1   7y1
.....................uucguacguagcagGgcagcu.......................................................................  1     1   7y1
.....................uucguacguagcagagcaCcu.......................................................................  3     1   7y1
.....................uucgGacguagcagagcagcu.......................................................................  1     1   7y1
.....................uucguacguagcagagcagcu.......................................................................  7198  0   7y1
.....................Aucguacguagcagagcagcu.......................................................................  80    1   7y1
.....................uucguacguagcagagcagcG.......................................................................  40    1   7y1
.....................uucgCacguagcagagcagcu.......................................................................  3     1   7y1
.....................uucguacguagAagagcagcu.......................................................................  3     1   7y1
.....................uucguacguagcagCgcagcu.......................................................................  7     1   7y1
.....................uucguacguGgcagagcagcu.......................................................................  2     1   7y1
.....................uucgAacguagcagagcagcu.......................................................................  9     1   7y1
.....................uucguacguagcagagcagcC.......................................................................  1     1   7y1
.....................uucguacguUgcagagcagcu.......................................................................  1     1   7y1
.....................uucguaUguagcagagcagcu.......................................................................  3     1   7y1
.....................Gucguacguagcagagcagcu.......................................................................  8     1   7y1
.....................uucguacCuagcagagcagcu.......................................................................  4     1   7y1
.....................uucguacguagcaAagcagcu.......................................................................  1     1   7y1
.....................uuAguacguagcagagcagcu.......................................................................  24    1   7y1
.....................uucguUcguagcagagcagcu.......................................................................  1     1   7y1
.....................uucguacguaCcagagcagcu.......................................................................  3     1   7y1
.....................uAcguacguagcagagcagcu.......................................................................  18    1   7y1
.....................uucguacguagcagagUagcu.......................................................................  1     1   7y1
.....................uucguacguagcagagGagcu.......................................................................  4     1   7y1
.....................uucguaGguagcagagcagcu.......................................................................  1     1   7y1
.....................uucguacguagcaCagcagcu.......................................................................  1     1   7y1
.....................uucguacAuagcagagcagcu.......................................................................  1     1   7y1
.....................uucguaAguagcagagcagcu.......................................................................  22    1   7y1
.....................uucguacguagcagagcCgcu.......................................................................  1     1   7y1
```

**Star**	**Mature**		
gaccugcuucugggucggggguuucguacguagcagagcagcucccucgcugcgaucuauugaaagucagcccucgacacaaggguuuguccgcgcgcgcgcgcgcgcgcgcgugc			
.....................uucguacguagGagagcagcu...	1	1	7y1
.....................uucguacUuagcagagcagcu...	2	1	7y1
.....................Cucguacguagcagagcagcu...	2	1	7y1
.....................uucguacguagcagagcagcuU..	1	1	7y1
.....................uucguaUguagcagagcagcuc..	3	1	7y1
.....................uucguacguagcagagcagAuc..	9	1	7y1
.....................uucguacguagcagagcagcAc..	4	1	7y1
.....................uucguacguagcagaUcagcuc..	6	1	7y1
.....................uucAuacguagcagagcagcuc..	6	1	7y1
.....................uucguacguaUcagagcagcuc..	11	1	7y1
.....................uucguacgCagcagagcagcuc..	1	1	7y1
.....................uucguaGguagcagagcagcuc..	1	1	7y1
.....................uucguacguagcagagcUgcuc..	1	1	7y1
.....................uucguacguagcagagcGgcuc..	3	1	7y1
.....................uAcguacguagcagagcagcuc..	27	1	7y1
.....................uucgCacguagcagagcagcuc..	2	1	7y1
.....................uucguacguagcagagcCgcuc..	1	1	7y1
.....................uucCuacguagcagagcagcuc..	2	1	7y1
.....................uucguacguagUagagcagcuc..	1	1	7y1
.....................uucguacguagcagagcaCcuc..	5	1	7y1
.....................uucguacguagcagagAagcuc..	19	1	7y1
.....................uucguacUuagcagagcagcuc..	5	1	7y1
.....................uucgAacguagcagagcagcuc..	8	1	7y1
.....................uucguacguagcaUagcagcuc..	5	1	7y1
.....................uuUguacguagcagagcagcuc..	2	1	7y1
.....................Aucguacguagcagagcagcuc..	123	1	7y1
.....................uucguacguagGagagcagcuc..	3	1	7y1
.....................uucguacguagcagagcagcuc..	12187	0	7y1
.....................uucguGcguagcagagcagcuc..	1	1	7y1
.....................uucguacguagAagagcagcuc..	13	1	7y1
.....................uucguacguaCcagagcagcuc..	9	1	7y1
.....................uucguacguagcaCagcagcuc..	5	1	7y1
.....................uucguacCuagcagagcagcuc..	7	1	7y1
.....................uCcguacguagcagagcagcuc..	200	1	7y1
.....................uucguacguagcagUgcagcuc..	2	1	7y1
.....................uucguacguagcagagUagcuc..	1	1	7y1
.....................uucguacAuagcagagcagcuc..	3	1	7y1
.....................uucguacguagcGgagcagcuc..	1	1	7y1
.....................uucguacguagcagagcaUcuc..	8	1	7y1
.....................uucguacguagcagagcagcuA..	8	1	7y1
.....................uucgGacguagcagagcagcuc..	1	1	7y1
.....................Nucguacguagcagagcagcuc..	5	1	7y1
.....................uucguacguagcagagcagcGc..	1	1	7y1
.....................uucguacguagcagagcagcCc..	1	1	7y1
.....................uucguUcguagcagagcagcuc..	2	1	7y1
.....................uuAguacguagcagagcagcuc..	33	1	7y1
.....................uucguacgAagcagagcagcuc..	15	1	7y1
.....................uucguacguagcagagGagcuc..	2	1	7y1
.....................uucguacguaAcagagcagcuc..	2	1	7y1
.....................uucguacguagcagagcagcuG..	1	1	7y1
.....................Gucguacguagcagagcagcuc..	29	1	7y1
.....................uucguacguGgcagagcagcuc..	2	1	7y1
.....................uucguacguagcagagcagUuc..	1	1	7y1
.....................uucguacguagcagagcagGuc..	1	1	7y1
.....................uucguacguagcagaAcagcuc..	1	1	7y1
.....................uucguCcguagcagagcagcuc..	1	1	7y1
.....................uucguacguagcagaCcagcuc..	8	1	7y1
.....................uucguacguagcagCgcagcuc..	16	1	7y1
.....................uucguaAguagcagagcagcuc..	55	1	7y1
.....................uucguacguagcagCgcagcucc...	4	1	7y1
.....................uucguacguagcagagcagAucc...	5	1	7y1
.....................uucguacguagcaUagcagcucc...	4	1	7y1
.....................uucguacAuagcagagcagcucc...	1	1	7y1
.....................uucguacguagcagaCcagcucc...	2	1	7y1
.....................uucguacguagcagagcagcCcc...	1	1	7y1
.....................uucguacguagcGgagcagcucc...	5	1	7y1
.....................uucUuacguagcagagcagcucc...	3	1	7y1
.....................uucguacguagcagagcagGucc...	1	1	7y1
.....................Gucguacguagcagagcagcucc...	17	1	7y1
.....................uucAuacguagcagagcagcucc...	5	1	7y1

Star **Mature**

gaccugcuucugggucgggguuucguacguagcagagcagcucccucgcugcgaucuauugaaagucagcccucgacacaaggguuuguccgcgcgcgcgcgcgcgcgcgugc

.....................uucguacguagcagagcagcucA...	13	1	7y1
.....................uucguacguagcUgagcagcucc...	2	1	7y1
.....................uucguacgGagcagagcagcucc...	1	1	7y1
.....................uucguacguagcagagcagcuAc...	9	1	7y1
.....................uucguacguagcagagcagcGcc...	1	1	7y1
.....................uucguacguaCcagagcagcucc...	3	1	7y1
.....................uuUguacguagcagagcagcucc...	1	1	7y1
.....................uucguacguagcagagcCgcucc...	1	1	7y1
.....................uucguacguagcagagGagcucc...	2	1	7y1
.....................uucguacguagcagagUagcucc...	1	1	7y1
.....................Nucguacguagcagagcagcucc...	4	1	7y1
.....................uucguacCuagcagagcagcucc...	6	1	7y1
.....................uucguaGguagcagagcagcucc...	1	1	7y1
.....................uNcguacguagcagagcagcucc...	1	1	7y1
.....................uucguaAguagcagagcagcucc...	26	1	7y1
.....................uucguacguagcagagAagcucc...	8	1	7y1
.....................uucgAacguagcagagcagcucc...	1	1	7y1
.....................uAcguacguagcagagcagcucc...	17	1	7y1
.....................uuAguacguagcagagcagcucc...	16	1	7y1
.....................uucguUcguagcagagcagcucc...	1	1	7y1
.....................uuGguacguagcagagcagcucc...	2	1	7y1
.....................uucguacgAagcagagcagcucc...	6	1	7y1
.....................Aucguacguagcagagcagcucc...	71	1	7y1
.....................uucguacUuagcagagcagcucc...	2	1	7y1
.....................uucguacguagcagagcagcucc...	7274	0	7y1
.....................uucguacguagAagagcagcucc...	11	1	7y1
.....................uucguacguaUcagagcagcucc...	8	1	7y1
.....................uucguacguagcagagcagcAcc...	1	1	7y1
.....................uucguacguGgcagagcagcucc...	2	1	7y1
.....................uucguCcguagcagagcagcucc...	1	1	7y1
.....................uucguacguagcagagcaAcucc...	1	1	7y1
.....................uucguacguagcagagcagcucG...	1	1	7y1
.....................uucgCacguagcagagcagcucc...	3	1	7y1
.....................uucguacguagcagagcaUcucc...	3	1	7y1
.....................uucguacguagcaCagcagcucc...	1	1	7y1
.....................uucguacguagGagagcagcucc...	1	1	7y1
.....................uucguacguagcagaUcagcucc...	5	1	7y1
.....................uucguacguagUagagcagcucc...	1	1	7y1
.....................uucguacguagcagagcaCcucc...	1	1	7y1
.....................uucguacguGgcagagcagcuccc..	1	1	7y1
.....................uucguacguaCcagagcagcuccc..	2	1	7y1
.....................uucguacguagcagagcCgcuccc..	2	1	7y1
.....................uucguacUuagcagagcagcuccc..	1	1	7y1
.....................uucguacguaUcagagcagcuccc..	1	1	7y1
.....................uucguacguagcagagcagcuAcc..	7	1	7y1
.....................uucguacguagcagagcagcuccA..	2	1	7y1
.....................uAcguacguagcagagcagcuccc..	6	1	7y1
.....................Nucguacguagcagagcagcuccc..	1	1	7y1
.....................uucguacguagcagagcagcuccc..	3826	0	7y1
.....................uucguacCuagcagagcagcuccc..	1	1	7y1
.....................Aucguacguagcagagcagcuccc..	31	1	7y1
.....................uucguacguagcagagcagcuUcc..	1	1	7y1
.....................uucguacguagcagagcNgcuccc..	1	1	7y1
.....................uucguCcguagcagagcagcuccc..	1	1	7y1
.....................uucguacguagcagagcagcCccc..	1	1	7y1
.....................uuAguacguagcagagcagcuccc..	10	1	7y1
.....................uucguacguagcagagcagcucAc..	16	1	7y1
.....................uucguacguagcGgagcagcuccc..	1	1	7y1
.....................uucguacguagcagagcagcuGcc..	1	1	7y1
.....................uucAuacguagcagagcagcuccc..	1	1	7y1
.....................uucguacgAagcagagcagcuccc..	2	1	7y1
.....................uucUuacguagcagagcagcuccc..	1	1	7y1
.....................uucguacguagcagagAagcuccc..	4	1	7y1
.....................uucgAacguagcagagcagcuccc..	4	1	7y1
.....................Gucguacguagcagagcagcuccc..	13	1	7y1
.....................uucguaAguagcagagcagcuccc..	11	1	7y1
.....................uucguacguagAagagcagcuccc..	1	1	7y1
.....................uucguacguagcaCagcagcuccc..	1	1	7y1
.....................uucguacguagcagaCcagcuccc..	1	1	7y1
.....................uucguacguagcagagcaCcuccc..	4	1	7y1

```
  Star                                                       Mature
gaccugcuucugggucgggguuucguacguagcagagcagcucccucgcugcgaucuauugaaagucagcccucgacacaaggguuuguccgcgcgcgcgcgcgcgcgcgugc
.....................uucguacguagcaUagcagcuccc....................................................................   6   1   7y1
.....................uucguacguagcagagUagcuccc....................................................................   1   1   7y1
.....................uucguacguagcagagcaUcuccc....................................................................   5   1   7y1
.....................uucguacguagcagagcaAcuccc....................................................................   1   1   7y1
.....................uucguacguagcagaUcagcuccc....................................................................   3   1   7y1
.....................uucguacguagcagagcagcAccc....................................................................   2   1   7y1
.....................uucguacguagcagagcagcucGc....................................................................   1   1   7y1
.....................uucCuacguagcagagcagcuccc....................................................................   1   1   7y1
.....................uucguacguagcagagcagcuUccu...................................................................   1   1   7y1
.....................uucUuacguagcagagcagcucccu...................................................................   4   1   7y1
.....................uucguacguagcagCgcagcucccu...................................................................   4   1   7y1
.....................uucguacguagcagagcagcGcccu...................................................................   2   1   7y1
.....................uucguacgCagcagagcagcucccu...................................................................   2   1   7y1
.....................uucguacguagcUgagcagcucccu...................................................................   1   1   7y1
.....................uucguacguagcagagcUgcucccu...................................................................   6   1   7y1
.....................uucguacguagcagagcagcucUcu...................................................................   5   1   7y1
.....................Gucguacguagcagagcagcucccu...................................................................  24   1   7y1
.....................uucguacguaAcagagcagcucccu...................................................................   1   1   7y1
.....................uucguacguagcagGgcagcucccu...................................................................   1   1   7y1
.....................Cucguacguagcagagcagcucccu...................................................................   1   1   7y1
.....................uucguacguagcagagcagcAcccu...................................................................   6   1   7y1
.....................uucguacguagcagagcagcuAccu...................................................................  23   1   7y1
.....................uucguacguagcagagAagcucccu...................................................................  11   1   7y1
.....................uucguacguagcagagcagcucAcu...................................................................  24   1   7y1
.....................uucguacguagcGgagcagcucccu...................................................................   2   1   7y1
.....................uucguacguagcagagcagcucccA...................................................................  13   1   7y1
.....................uNcguacguagcagagcagcucccu...................................................................   1   1   7y1
.....................uucguacguagcagagcCgcucccu...................................................................   2   1   7y1
.....................uucguacguagcagaCcagcucccu...................................................................   3   1   7y1
.....................uucguUcguagcagagcagcucccu...................................................................   1   1   7y1
.....................Nucguacguagcagagcagcucccu...................................................................   8   1   7y1
.....................uucguacgGagcagagcagcucccu...................................................................   1   1   7y1
.....................uucguacguagAagagcagcucccu...................................................................   7   1   7y1
.....................uucguacguagcagagcagcuccGu...................................................................   2   1   7y1
.....................uucguacgAagcagagcagcucccu...................................................................   9   1   7y1
.....................uuGguacguagcagagcagcucccu...................................................................   2   1   7y1
.....................uucCuacguagcagagcagcucccu...................................................................   1   1   7y1
.....................uucguaUguagcagagcagcucccu...................................................................   1   1   7y1
.....................uucguacguagcagUgcagcucccu...................................................................   1   1   7y1
.....................uucgCacguagcagagcagcucccu...................................................................   2   1   7y1
.....................uucguacguagcCgagcagcucccu...................................................................   2   1   7y1
.....................uucguacguagcagagcagAucccu...................................................................   5   1   7y1
.....................uucguacguagcagagcaAcucccu...................................................................   1   1   7y1
.....................uucguacguagUagagcagcucccu...................................................................   1   1   7y1
.....................uucguacguagcaUagcagcucccu...................................................................   5   1   7y1
.....................Aucguacguagcagagcagcucccu...................................................................  78   1   7y1
.....................uucguacguagcaCagcagcucccu...................................................................   3   1   7y1
.....................uucguacguagcagagcaCcucccu...................................................................   1   1   7y1
.....................uucguacguagcagagGagcucccu...................................................................   2   1   7y1
.....................uucguacguagcagagUagcucccu...................................................................   1   1   7y1
.....................uucguacguGgcagagcagcucccu...................................................................   1   1   7y1
.....................uucgAacguagcagagcagcucccu...................................................................   5   1   7y1
.....................uucguaGguagcagagcagcucccu...................................................................   1   1   7y1
.....................uucguacguagcagagcaUcucccu...................................................................   5   1   7y1
.....................uucguacguagcagagcagcCcccu...................................................................   2   1   7y1
.....................uucguacguagcagagcagcuGccu...................................................................   1   1   7y1
.....................uucguacguagcagagcagcucccG...................................................................  35   1   7y1
.....................uucguacguaUcagagcagcucccu...................................................................   5   1   7y1
.....................uucguacguaCcagagcagcucccu...................................................................   3   1   7y1
.....................uucguacguagcagagcGgcucccu...................................................................   8   1   7y1
.....................uucguacguagcagagcagcucccC...................................................................   1   1   7y1
.....................uucguacUuagcagagcagcucccu...................................................................   4   1   7y1
.....................uucguacguagcagagcagcuccAu...................................................................   6   1   7y1
.....................uucguacCuagcagagcagcucccu...................................................................   5   1   7y1
.....................uucguacguagGagagcagcucccu...................................................................   1   1   7y1
.....................uucguGcguagcagagcagcucccu...................................................................   1   1   7y1
.....................uucguaAguagcagagcagcucccu...................................................................  40   1   7y1
.....................uucguacAuagcagagcagcucccu...................................................................   3   1   7y1
.....................uuAguacguagcagagcagcucccu...................................................................  23   1   7y1
.....................uAcguacguagcagagcagcucccu...................................................................  21   1   7y1
```

Star **Mature**

```
gaccugcuucugggucgggguuucguacguagcagagcagcucccucgcugcgaucuauugaaagucagcccucgacacaaggguuuguccgcgcgcgcgcgcgcgcgugc
.....................uucguacguagcagaUcagcucccu.................................................................    2     1    7y1
.....................uucguacguagcaAagcagcucccu.................................................................    2     1    7y1
.....................uCcguacguagcagagcagcucccu.................................................................    2     1    7y1
.....................uucguacguagcagagcagcucccu.................................................................    9826  0    7y1
.....................uucAuacguagcagagcagcucccu.................................................................    1     1    7y1
.....................uucguacguagcaUagcagcucccuc................................................................    4     1    7y1
.....................uucguacguagcagagcagcuAccuc................................................................    12    1    7y1
.....................uucguacguagcGgagcagcucccuc................................................................    1     1    7y1
.....................uucguacguagcagagcaUcucccuc................................................................    1     1    7y1
.....................uucCuacguagcagagcagcucccuc................................................................    2     1    7y1
.....................uucguacguagcagagcagcuccAuc................................................................    6     1    7y1
.....................uucguacguaUcagagcagcucccuc................................................................    3     1    7y1
.....................uucguacguagcagGgcagcucccuc................................................................    1     1    7y1
.....................uucguacguaCcagagcagcucccuc................................................................    1     1    7y1
.....................uucguacAuagcagagcagcucccuc................................................................    1     1    7y1
.....................uucguaAguagcagagcagcucccuc................................................................    33    1    7y1
.....................uucguacguaAcagagcagcucccuc................................................................    1     1    7y1
.....................uucguacgCagcagagcagcucccuc................................................................    1     1    7y1
.....................Nucguacguagcagagcagcucccuc................................................................    5     1    7y1
.....................uucguacguagcagagcagcGcccuc................................................................    1     1    7y1
.....................uucguacguagcagagcaCcucccuc................................................................    1     1    7y1
.....................uucguacguagcagagcagcucccuA................................................................    1     1    7y1
.....................uucguacguagcagagcagcucccAc................................................................    1     1    7y1
.....................uucguacguagcagagUagcucccuc................................................................    1     1    7y1
.....................uucguacguagcagagcagcucccuc................................................................    7376  0    7y1
.....................uucguacguagcagaUcagcucccuc................................................................    4     1    7y1
.....................uucguacUuagcagagcagcucccuc................................................................    2     1    7y1
.....................uucguacgAagcagagcagcucccuc................................................................    6     1    7y1
.....................Gucguacguagcagagcagcucccuc................................................................    10    1    7y1
.....................uucguacguagcagagcagcuUccuc................................................................    1     1    7y1
.....................uucguacguagcagagcagcucccCc................................................................    2     1    7y1
.....................uucguacguagcagUgcagcucccuc................................................................    2     1    7y1
.....................uucguacguagcagagcUgcucccuc................................................................    1     1    7y1
.....................uucguacguagcagagcCgcucccuc................................................................    3     1    7y1
.....................uucguacguagcagagcagcAcccuc................................................................    5     1    7y1
.....................uucguacguagcagagcaAcucccuc................................................................    2     1    7y1
.....................uucguGcguagcagagcagcucccuc................................................................    1     1    7y1
.....................uucguUcguagcagagcagcucccuc................................................................    1     1    7y1
.....................uucguacCuagcagagcagcucccuc................................................................    4     1    7y1
.....................uucguacguagcagagcagcucccuG................................................................    1     1    7y1
.....................uucgAacguagcagagcagcucccuc................................................................    4     1    7y1
.....................uucguacguagcUgagcagcucccuc................................................................    2     1    7y1
.....................Cucguacguagcagagcagcucccuc................................................................    1     1    7y1
.....................uucguacguagAagagcagcucccuc................................................................    5     1    7y1
.....................uucguacguagcagCgcagcucccuc................................................................    8     1    7y1
.....................Aucguacguagcagagcagcucccuc................................................................    59    1    7y1
.....................uuGguacguagcagagcagcucccuc................................................................    1     1    7y1
.....................uucguacguagcagagAagcucccuc................................................................    11    1    7y1
.....................uAcguacguagcagagcagcucccuc................................................................    11    1    7y1
.....................uuUguacguagcagagcagcucccuc................................................................    1     1    7y1
.....................uucguacguagcagagcagAucccuc................................................................    5     1    7y1
.....................uucUuacguagcagagcagcucccuc................................................................    2     1    7y1
.....................uuAguacguagcagagcagcucccuc................................................................    12    1    7y1
.....................uCcguacguagcagagcagcucccuc................................................................    1     1    7y1
.....................uucgCacguagcagagcagcucccuc................................................................    253   1    7y1
.....................uucguacguagcaAagcagcucccuc................................................................    1     1    7y1
.....................uucguacguagcagaCcagcucccuc................................................................    2     1    7y1
.....................uucguacguagcagagcagcucccGc................................................................    1     1    7y1
.....................uucgGacguagcagagcagcucccuc................................................................    1     1    7y1
.....................uucguacguagcagagcGgcucccuc................................................................    2     1    7y1
.....................uucguacguagcagagcagcuGccuc................................................................    4     1    7y1
.....................uucguacguagcagagcagcucAcuc................................................................    19    1    7y1
.....................uucCuacguagcagagcagcucccucg...............................................................    1     1    7y1
.....................uucguCcguagcagagcagcucccucg...............................................................    1     1    7y1
.....................uAcguacguagcagagcagcucccucg...............................................................    2     1    7y1
.....................uucguacguagcagagcagcucccucC...............................................................    1     1    7y1
.....................uucguacguagcagGgcagcucccucg...............................................................    2     1    7y1
.....................uucguacguagcagagcagcucccucA...............................................................    1     1    7y1
.....................uucguacguagcagaUcagcucccucg...............................................................    3     1    7y1
.....................Nucguacguagcagagcagcucccucg...............................................................    1     1    7y1
```

Star Mature

```
gaccugcuucugggucggggguucguacguagcagagcagcucccucgcugcgaucuauugaaagucagcccucgacacaaggguuuguccgcgcgcgcgcgcgcgcgcgcgugc
.....................uucguacguagcagagcagcucAcucg...................................................................  4     1  7y1
.....................uucguacguagcagagcaCcucccucg...................................................................  1     1  7y1
.....................uucguacCuagcagagcagcucccucg...................................................................  3     1  7y1
.....................uucgAacguagcagagcagcucccucg...................................................................  2     1  7y1
.....................uucguacguaCcagagcagcucccucg...................................................................  1     1  7y1
.....................uucguacguagcagagcagcucccGcg...................................................................  1     1  7y1
.....................uuAguacguagcagagcagcucccucg...................................................................  3     1  7y1
.....................Gucguacguagcagagcagcucccucg...................................................................  1     1  7y1
.....................uucguaAguagcagagcagcucccucg...................................................................  5     1  7y1
.....................uucguacguagcagCgcagcucccucg...................................................................  2     1  7y1
.....................uucguGcguagcagagcagcucccucg...................................................................  1     1  7y1
.....................uucguacgCagcagagcagcucccucg...................................................................  1     1  7y1
.....................uucguacguagcagagcagcuAccucg...................................................................  1     1  7y1
.....................uucguacguagcagagcagcuccAucg...................................................................  1     1  7y1
.....................uucguacguagcagagcagcucccucg...................................................................  1431  0  7y1
.....................Aucguacguagcagagcagcucccucg...................................................................  13    1  7y1
.....................uucguacguagcagagcagcucccAcg...................................................................  1     1  7y1
.....................uucgCacguagcagagcagcucccucg...................................................................  1     1  7y1
.....................uucguacguagcagagAagcucccucg...................................................................  1     1  7y1
.....................uucguacguagcagagUagcucccucg...................................................................  1     1  7y1
.....................uucguacguagcagagcagAucccucgc..................................................................  1     1  7y1
.....................uuAguacguagcagagcagcucccucgc..................................................................  2     1  7y1
.....................uucguacguagcagagcaAcucccucgc..................................................................  1     1  7y1
.....................uucguacguagcagagcaCcucccucgc..................................................................  1     1  7y1
.....................uucguaAguagcagagcagcucccucgc..................................................................  3     1  7y1
.....................uucguacguagcagagcagcucccucgA..................................................................  1     1  7y1
.....................Cucguacguagcagagcagcucccucgc..................................................................  1     1  7y1
.....................uucguacguagcagagcagcucccucgc..................................................................  923   0  7y1
.....................uucgCacguagcagagcagcucccucgc..................................................................  1     1  7y1
.....................uucguacguagcagagcagGucccucgc..................................................................  1     1  7y1
.....................uAcguacguagcagagcagcucccucgc..................................................................  1     1  7y1
.....................uucguacguagcagagcagcuGccucgc..................................................................  1     1  7y1
.....................uucguacguaCcagagcagcucccucgc..................................................................  1     1  7y1
.....................uucguacguagcagagcagcuAccucgc..................................................................  2     1  7y1
.....................Aucguacguagcagagcagcucccucgc..................................................................  15    1  7y1
.....................uucguacgAagcagagcagcucccucgc..................................................................  1     1  7y1
.....................Gucguacguagcagagcagcucccucgc..................................................................  1     1  7y1
.....................uucguacguagcagagcGgcucccucgc..................................................................  1     1  7y1
.....................uucguacUuagcagagcagcucccucgc..................................................................  1     1  7y1
.....................uucguacguagcagagcagcucAcucgc..................................................................  1     1  7y1
.....................Nucguacguagcagagcagcucccucgc..................................................................  1     1  7y1
.....................uucguacguagcagagcagcucccuAgc..................................................................  1     1  7y1
.....................uucguacguagcagagcagcucccucgcA.................................................................  2     1  7y1
.....................uucguacUuagcagagcagcucccucgcu.................................................................  3     1  7y1
.....................uucguacguagcagagcagcCcccucgcu.................................................................  1     1  7y1
.....................uCcguacguagcagagcagcucccucgcu.................................................................  2     1  7y1
.....................uucguacguagcagagAagcucccucgcu.................................................................  3     1  7y1
.....................uucguacguagcagagcUgcucccucgcu.................................................................  2     1  7y1
.....................uucguacguagcagagcagcucAcucgcu.................................................................  9     1  7y1
.....................uucguacguagcagagcaUcucccucgcu.................................................................  2     1  7y1
.....................uucguaAguagcagagcagcucccucgcu.................................................................  13    1  7y1
.....................uucguacguagcagagcGgcucccucgcu.................................................................  2     1  7y1
.....................uucguacguagcagagcagcucGcucgcu.................................................................  1     1  7y1
.....................uucguacguagAagagcagcucccucgcu.................................................................  6     1  7y1
.....................uucguacCuagcagagcagcucccucgcu.................................................................  1     1  7y1
.....................uucguacguagcagagcagcucccucgGu.................................................................  1     1  7y1
.....................uucAuacguagcagagcagcucccucgcu.................................................................  1     1  7y1
.....................uucguGcguagcagagcagcucccucgcu.................................................................  1     1  7y1
.....................uucCuacguagcagagcagcucccucgcu.................................................................  4     1  7y1
.....................uucguacguagcagagcagcucccucgcu.................................................................  4044  0  7y1
.....................Gucguacguagcagagcagcucccucgcu.................................................................  6     1  7y1
.....................uucguacguagcagagcagcuUccucgcu.................................................................  1     1  7y1
.....................uucguacguagcagagcagAucccucgcu.................................................................  1     1  7y1
.....................uucguacguagcagagcagcucccAcgcu.................................................................  2     1  7y1
.....................uGcguacguagcagagcagcucccucgcu.................................................................  1     1  7y1
.....................uucguacguagcGgagcagcucccucgcu.................................................................  2     1  7y1
.....................uucguacguagcagagcagcucccucUcu.................................................................  1     1  7y1
.....................uucguacguagcagagcagcucccucgAu.................................................................  3     1  7y1
.....................uucguacguagcaUagcagcucccucgcu.................................................................  4     1  7y1
.....................uucguacAuagcagagcagcucccucgcu.................................................................  2     1  7y1
```

Star **Mature**

gaccugcuucugggucgggguuucguacguagcagagcagcucccucgcugcgaucuauugaaagucagcccucgacacaaggguuuguccgcgcgcgcgcgcgcgcgcgcgugc			
.....................uucguacgAagcagagcagcucccucgcu...	4	1	7y1
.....................uucguacguagcagagcagcuAccucgcu...	5	1	7y1
.....................uAcguacguagcagagcagcucccucgcu...	6	1	7y1
.....................uuGguacguagcagagcagcucccucgcu...	2	1	7y1
.....................uucguacguagcUgagcagcucccucgcu...	1	1	7y1
.....................uucguacguaCcagagcagcucccucgcu...	1	1	7y1
.....................uucguacguagcagagcagcucccucCcu...	4	1	7y1
.....................uucguacguagcagagcCgcucccucgcu...	1	1	7y1
.....................Aucguacguagcagagcagcucccucgcu...	36	1	7y1
.....................uNcguacguagcagagcagcucccucgcu...	1	1	7y1
.....................uuAguacguagcagagcagcucccucgcu...	8	1	7y1
.....................uucguacguagcagagcagcucccucgcG...	23	1	7y1
.....................uucguaUguagcagagcagcucccucgcu...	1	1	7y1
.....................uucguacguagcagagcagcuccAucgcu...	2	1	7y1
.....................uucguacguagcagagcagcucccuAgcu...	1	1	7y1
.....................uucguacguagcagagcagcAcccucgcu...	1	1	7y1
.....................uucgAacguagcagagcagcucccucgcu...	1	1	7y1
.....................uucgGacguagcagagcagcucccucgcu...	1	1	7y1
.....................Nucguacguagcagagcagcucccucgcu...	2	1	7y1
.....................uucCuacguagcagagcagcucccucgcug..	1	1	7y1
.....................uucguacguagcagagcagcucccuAgcug..	2	1	7y1
.....................uucguacgAagcagagcagcucccucgcug..	1	1	7y1
.....................uucguaAguagcagagcagcucccucgcug..	4	1	7y1
.....................uucguacUuagcagagcagcucccucgcug..	1	1	7y1
.....................Nucguacguagcagagcagcucccucgcug..	1	1	7y1
.....................uucguacguagcagaCcagcucccucgcug..	1	1	7y1
.....................Aucguacguagcagagcagcucccucgcug..	5	1	7y1
.....................uucguacguagcagagcagcucccucgcGg..	1	1	7y1
.....................uucguacguagcagagcagAucccucgcug..	1	1	7y1
.....................uucguacguagcagagcagcucccucgcug..	459	0	7y1
.....................uAcguacguagcagagcagcucccucgcug..	2	1	7y1
.....................uucguacCuagcagagcagcucccucgcug..	2	1	7y1
.....................Gucguacguagcagagcagcucccucgcugc...	1	1	7y1
.....................uucguGcguagcagagcagcucccucgcugc...	1	1	7y1
.....................uucguacguagcagagcagcucccucgcuCc...	1	1	7y1
.....................uucguacguagcaUagcagcucccucgcugc...	1	1	7y1
.....................uucguacUuagcagagcagcucccucgcugc...	1	1	7y1
.....................Aucguacguagcagagcagcucccucgcugc...	9	1	7y1
.....................uucguacguaCcagagcagcucccucgcugc...	1	1	7y1
.....................uucguacguagcagagcagcucAcucgcugc...	2	1	7y1
.....................uucguaAguagcagagcagcucccucgcugc...	1	1	7y1
.....................uucguacguagcagagcCgcucccucgcugc...	1	1	7y1
.....................uucguacguagAagagcagcucccucgcugc...	2	1	7y1
.....................uuAguacguagcagagcagcucccucgcugc...	1	1	7y1
.....................uucguacguagcagaCcagcucccucgcugc...	1	1	7y1
.....................uucguacguagcagagcagcucccucgcugA...	1	1	7y1
.....................uucguacguagcagagcagcuccAucgcugc...	1	1	7y1
.....................uAcguacguagcagagcagcucccucgcugc...	3	1	7y1
.....................uucguacguagcagGgcagcucccucgcugc...	1	1	7y1
.....................uucguacguagcagagcagcucccucgcugc...	924	0	7y1
......................ucguacCuagcagagcagc..	1	1	7y1
......................ucguacguagcagagcagc..	1251	0	7y1
......................ucguacguagAagagcagc..	3	1	7y1
......................ucguacguagcagagcagA..	1	1	7y1
......................ucguacgAagcagagcagc..	3	1	7y1
......................Acguacguagcagagcagc..	13	1	7y1
......................uAguacguagcagagcagc..	2	1	7y1
......................ucguaAguagcagagcagc..	2	1	7y1
......................ucguacguagcagCgcagc..	4	1	7y1
......................Gcguacguagcagagcagc..	1	1	7y1
......................ucguacUuagcagagcagc..	1	1	7y1
......................ucguacguagcagagcagG..	1	1	7y1
......................ucguacguagcagagcaUc..	2	1	7y1
......................ucguacguagcaCagcagc..	1	1	7y1
......................ucguacguagcGgagcagc..	1	1	7y1
......................ucguacguagcaCagcagcu...	2	1	7y1
......................uNguacguagcagagcagcu...	1	1	7y1
......................uAguacguagcagagcagcu...	6	1	7y1
......................ucUuacguagcagagcagcu...	2	1	7y1
......................ucguacguagcagagcagcG...	20	1	7y1

Star **Mature**

```
gaccugcuucugggucggggguuucguacguagcagagcagcucccucgcugcgaucuauugaaagucagcccucgacacaaggguuuguccgcgcgcgcgcgcgcgcgcgugc
```

```
......................ucguacguagcagagAagcu.......................................................................    6     1    7y1
......................ucguacguagAagagcagcu.......................................................................    5     1    7y1
......................ucguacguagcagagcaUcu.......................................................................    4     1    7y1
......................ucguacguagcagCgcagcu.......................................................................    4     1    7y1
......................Gcguacguagcagagcagcu.......................................................................    6     1    7y1
......................ucguacguagcagagcagcu.......................................................................    2186  0    7y1
......................ucguacguagcagagcagcA.......................................................................    1     1    7y1
......................uUguacguagcagagcagcu.......................................................................    1     1    7y1
......................ucguacguagcagaUcagcu.......................................................................    2     1    7y1
......................ucguacguaCcagagcagcu.......................................................................    1     1    7y1
......................ucguaAguagcagagcagcu.......................................................................    6     1    7y1
......................ucguacguagcagagcGgcu.......................................................................    1     1    7y1
......................ucguacguagcagUgcagcu.......................................................................    1     1    7y1
......................Acguacguagcagagcagcu.......................................................................    36    1    7y1
......................ucguacguaUcagagcagcu.......................................................................    2     1    7y1
......................ucguGcguagcagagcagcu.......................................................................    1     1    7y1
......................ucguacguagcagagcUgcu.......................................................................    1     1    7y1
......................ucguacgAagcagagcagcu.......................................................................    2     1    7y1
......................uAguacguagcagagcagcuc......................................................................    19    1    7y1
......................ucguacguagcagaUcagcuc......................................................................    6     1    7y1
......................ucgAacguagcagagcagcuc......................................................................    1     1    7y1
......................ucguacguaCcagagcagcuc......................................................................    6     1    7y1
......................ucguacCuagcagagcagcuc......................................................................    4     1    7y1
......................ucguacguagcagagcaCcuc......................................................................    6     1    7y1
......................ucUuacguagcagagcagcuc......................................................................    5     1    7y1
......................ucguacguagcagagcagcuc......................................................................    6627  0    7y1
......................Acguacguagcagagcagcuc......................................................................    79    1    7y1
......................ucguacguagcagagGagcuc......................................................................    1     1    7y1
......................ucguacguagAagagcagcuc......................................................................    9     1    7y1
......................ucguacguagcagGgcagcuc......................................................................    2     1    7y1
......................ucguacguagcagagcagcuA......................................................................    7     1    7y1
......................ucguacguagcagagUagcuc......................................................................    1     1    7y1
......................ucguacguagcaCagcagcuc......................................................................    238   1    7y1
......................ucguacguagcagUgcagcuc......................................................................    1     1    7y1
......................ucguacguagUagagcagcuc......................................................................    1     1    7y1
......................ucguacgAagcagagcagcuc......................................................................    9     1    7y1
......................Gcguacguagcagagcagcuc......................................................................    7     1    7y1
......................ucguacUuagcagagcagcuc......................................................................    6     1    7y1
......................Ncguacguagcagagcagcuc......................................................................    4     1    7y1
......................ucguacgCagcagagcagcuc......................................................................    2     1    7y1
......................ucguacguagcagagcGgcuc......................................................................    1     1    7y1
......................uGguacguagcagagcagcuc......................................................................    1     1    7y1
......................uUguacguagcagagcagcuc......................................................................    1     1    7y1
......................ucguacguagcagagcagGuc......................................................................    1     1    7y1
......................ucguacguagcagCgcagcuc......................................................................    5     1    7y1
......................ucguacguaAcagagcagcuc......................................................................    1     1    7y1
......................ucguacguagcagaCcagcuc......................................................................    1     1    7y1
......................ucguacguagcagagcagcGc......................................................................    3     1    7y1
......................ucguacAuagcagagcagcuc......................................................................    1     1    7y1
......................ucguacguagcagagcagcAc......................................................................    4     1    7y1
......................ucCuacguagcagagcagcuc......................................................................    5     1    7y1
......................ucguacguagcagagcCgcuc......................................................................    1     1    7y1
......................ucguacguaUcagagcagcuc......................................................................    3     1    7y1
......................ucguacguagcagagcUgcuc......................................................................    2     1    7y1
......................ucguaAguagcagagcagcuc......................................................................    15    1    7y1
......................ucguacguagcagagcaUcuc......................................................................    3     1    7y1
......................ucguUcguagcagagcagcuc......................................................................    1     1    7y1
......................ucguacguagcGgagcagcuc......................................................................    2     1    7y1
......................ucguacguagcagagAagcuc......................................................................    15    1    7y1
......................ucguaUguagcagagcagcuc......................................................................    2     1    7y1
......................ucguaGguagcagagcagcuc......................................................................    1     1    7y1
......................ucguacguagcagagcagcuG......................................................................    1     1    7y1
......................ucAuacguagcagagcagcuc......................................................................    1     1    7y1
......................ucguacguagcagagcagAuc......................................................................    3     1    7y1
......................ucguacguGgcagagcagcucc.....................................................................    1     1    7y1
......................ucguacgAagcagagcagcucc.....................................................................    1     1    7y1
......................ucguacguagcagaUcagcucc.....................................................................    1     1    7y1
......................ucguacguagAagagcagcucc.....................................................................    1     1    7y1
......................ucguacguagcGgagcagcucc.....................................................................    1     1    7y1
......................uGguacguagcagagcagcucc.....................................................................    1     1    7y1
```

Star **Mature**

gaccugcuucugggucgggguuucguacguagcagagcagcucccucgcugcgaucuauugaaagucagcccucgacacaaggguuuguccgcgcgcgcgcgcgcgcgcgugc			
......................ucguacguagcagagUagcucc...	1	1	7y1
......................Ncguacguagcagagcagcucc...	1	1	7y1
......................ucguacguagcagagcagcucA...	6	1	7y1
......................ucguacguagcagagcagcucU...	1	1	7y1
......................ucUuacguagcagagcagcucc...	2	1	7y1
......................ucguacguagcagagAagcucc...	2	1	7y1
......................ucguacguagcagagcagAucc...	1	1	7y1
......................Acguacguagcagagcagcucc...	15	1	7y1
......................Gcguacguagcagagcagcucc...	5	1	7y1
......................ucguacguagcagaCcagcucc...	1	1	7y1
......................ucguacguagcagagcagcucc...	1758	0	7y1
......................ucguacguagcagagcagcCcc...	1	1	7y1
......................ucguaAguagcagagcagcucc...	4	1	7y1
......................ucguacguaUcagagcagcucc...	2	1	7y1
......................ucguaUguagcagagcagcucc...	1	1	7y1
......................ucguacguagcagagcagcuAc...	4	1	7y1
......................ucguacguagcagagcagcuUc...	1	1	7y1
......................uAguacguagcagagcagcucc...	1	1	7y1
......................ucguacguagcagGgcagcucc...	1	1	7y1
......................ucguGcguagcagagcagcucc...	1	1	7y1
......................ucguacguaCcagagcagcucc...	4	1	7y1
......................ucguacguagcagaAcagcucc...	1	1	7y1
......................ucCuacguagcagagcagcucc...	2	1	7y1
......................ucguacguagcagUgcagcuccc..	1	1	7y1
......................ucguacguagcagagcagcuAcc..	3	1	7y1
......................ucguacguagcagagcagcuccc..	2876	0	7y1
......................Gcguacguagcagagcagcuccc..	4	1	7y1
......................ucguacguagcagagcagcAccc..	1	1	7y1
......................ucguacguagcagagcagcucAc..	5	1	7y1
......................ucguacguagAagagcagcuccc..	3	1	7y1
......................ucguaAguagcagagcagcuccc..	8	1	7y1
......................uAguacguagcagagcagcuccc..	2	1	7y1
......................ucguacguagcagagcaUcuccc..	1	1	7y1
......................ucguacguaUcagagcagcuccc..	1	1	7y1
......................uUguacguagcagagcagcuccc..	2	1	7y1
......................ucguacguagcagagcaCcuccc..	2	1	7y1
......................ucguacguagcagagAagcuccc..	4	1	7y1
......................ucguacguaCcagagcagcuccc..	2	1	7y1
......................ucguacguagcagCgcagcuccc..	7	1	7y1
......................ucguacguagcagagcagcuccA..	2	1	7y1
......................ucAuacguagcagagcagcuccc..	2	1	7y1
......................ucUuacguagcagagcagcuccc..	1	1	7y1
......................ucgAacguagcagagcagcuccc..	1	1	7y1
......................ucguacguagcagagcagcuGcc..	1	1	7y1
......................ucguUcguagcagagcagcuccc..	1	1	7y1
......................ucguacguagcagagcagAuccc..	4	1	7y1
......................Acguacguagcagagcagcuccc..	26	1	7y1
......................uGguacguagcagagcagcuccc..	1	1	7y1
......................ucguacCuagcagagcagcuccc..	1	1	7y1
......................ucguacgAagcagagcagcuccc..	1	1	7y1
......................ucguacguagcagagGagcuccc..	1	1	7y1
......................ucguacguagcagaAcagcuccc..	1	1	7y1
......................ucguacguagcaCagcagcuccc..	2	1	7y1
......................ucguacgAagcagagcagcucccu...	3	1	7y1
......................Ccguacguagcagagcagcucccu...	1	1	7y1
......................ucguacguagcagCgcagcucccu...	6	1	7y1
......................uUguacguagcagagcagcucccu...	1	1	7y1
......................ucguacguaUcagagcagcucccu...	1	1	7y1
......................ucguacguagcagagcagcucGcu...	1	1	7y1
......................ucguacguagcUgagcagcucccu...	1	1	7y1
......................ucguacguagcagagcagcuGccu...	1	1	7y1
......................ucguacguagcagagcagUucccu...	1	1	7y1
......................ucguacguagcagagcagcuAccu...	2	1	7y1
......................Gcguacguagcagagcagcucccu...	5	1	7y1
......................ucguacguagcagaCcagcucccu...	2	1	7y1
......................ucguacguagcaUagcagcucccu...	2	1	7y1
......................uAguacguagcagagcagcucccu...	6	1	7y1
......................ucguacguagcagagAagcucccu...	1	1	7y1
......................ucguacguagcagagcagcCcccu...	1	1	7y1
......................ucguacguagcagagcCgcucccu...	2	1	7y1

Star Mature

```
gaccugcuucugggucgggguuucguacguagcagagcagcucccucgcugcgaucuauugaaagucagcccucgacacaaggguuuguccgcgcgcgcgcgcgcgcgcgugc
......................ucguacguGgcagagcagcucccu................................................................... 2 1 7y1
......................ucguGcguagcagagcagcucccu................................................................... 1 1 7y1
......................ucguacguaCcagagcagcucccu................................................................... 1 1 7y1
......................ucguaAguagcagagcagcucccu................................................................... 3 1 7y1
......................ucguacguagcagagcagcucccG................................................................... 9 1 7y1
......................ucguacguagcGgagcagcucccu................................................................... 2 1 7y1
......................ucgAacguagcagagcagcucccu................................................................... 1 1 7y1
......................ucguacguagcagagcagAucccu................................................................... 1 1 7y1
......................ucguacguagcagagcagcucccu................................................................... 2486 0 7y1
......................ucguacguagcagagcagcucAcu................................................................... 4 1 7y1
......................ucCuacguagcagagcagcucccu................................................................... 2 1 7y1
......................ucguacguagcagagUagcucccu................................................................... 1 1 7y1
......................ucguacguagcagagcagcuUccu................................................................... 1 1 7y1
......................ucguacguagcagagcagcucccA................................................................... 5 1 7y1
......................ucguacguagcagagcagGucccu................................................................... 1 1 7y1
......................ucguacguagAagagcagcucccu................................................................... 1 1 7y1
......................Acguacguagcagagcagcucccu................................................................... 21 1 7y1
......................ucguacguagcagagcagcuccAu................................................................... 6 1 7y1
......................ucguacUuagcagagcagcucccu................................................................... 3 1 7y1
......................ucguacCuagcagagcagcucccu................................................................... 1 1 7y1
......................uUguacguagcagagcagcucccuc.................................................................. 1 1 7y1
......................ucguacCuagcagagcagcucccuc.................................................................. 1 1 7y1
......................ucgAacguagcagagcagcucccuc.................................................................. 1 1 7y1
......................ucguacguagcagagcagcuAccuc.................................................................. 1 1 7y1
......................ucguacguagcagagcagcuUccuc.................................................................. 1 1 7y1
......................ucguacguagAagagcagcucccuc.................................................................. 1 1 7y1
......................ucCuacguagcagagcagcucccuc.................................................................. 1 1 7y1
......................ucguacguagcagagcagcucccuG.................................................................. 1 1 7y1
......................ucguacguagcagagcagcucccuc.................................................................. 555 0 7y1
......................Acguacguagcagagcagcucccuc.................................................................. 7 1 7y1
......................ucguacguaCcagagcagcucccuc.................................................................. 1 1 7y1
......................ucguaAguagcagagcagcucccuc.................................................................. 1 1 7y1
......................Ncguacguagcagagcagcucccuc.................................................................. 1 1 7y1
......................ucguacguagcagagcagcAcccuc.................................................................. 1 1 7y1
......................ucguacguagcagagcGgcucccuc.................................................................. 1 1 7y1
......................ucguacguagcagagcagcucAcuc.................................................................. 1 1 7y1
......................ucguacguagcagCgcagcucccucg................................................................. 1 1 7y1
......................Ncguacguagcagagcagcucccucg................................................................. 1 1 7y1
......................ucguacguagcagagcagcuccAucg................................................................. 1 1 7y1
......................ucguacguaCcagagcagcucccucg................................................................. 1 1 7y1
......................ucguacguagcCgagcagcucccucg................................................................. 1 1 7y1
......................ucguacguagcagaUcagcucccucg................................................................. 1 1 7y1
......................ucguacgAagcagagcagcucccucg................................................................. 1 1 7y1
......................ucCuacguagcagagcagcucccucg................................................................. 1 1 7y1
......................ucguacguagcagagcaCcucccucg................................................................. 1 1 7y1
......................ucguacguagcagagcagcucccucg................................................................. 219 0 7y1
......................ucguacAuagcagagcagcucccucg................................................................. 1 1 7y1
......................uAguacguagcagagcagcucccucg................................................................. 1 1 7y1
......................ucguaAguagcagagcagcucccucgc................................................................ 2 1 7y1
......................ucguacguagcagagcagcucccuAgc................................................................ 1 1 7y1
......................ucguacguagcagagcagcuAccucgc................................................................ 1 1 7y1
......................ucguacguagcagagcagcucccucgc................................................................ 270 0 7y1
......................Acguacguagcagagcagcucccucgc................................................................ 4 1 7y1
......................uAguacguagcagagcagcucccucgc................................................................ 2 1 7y1
......................ucguacguagcagagAagcucccucgc................................................................ 1 1 7y1
......................ucguacguagcagagcagcucccucAcu............................................................... 1 1 7y1
......................ucguacguagcagaAcagcucccucgcu............................................................... 1 1 7y1
......................ucguacguaAcagagcagcucccucgcu............................................................... 1 1 7y1
......................ucguacguagcagagcagcCcccucgcu............................................................... 1 1 7y1
......................ucguacguaCcagagcagcucccucgcu............................................................... 1 1 7y1
......................ucguacguagcagagcagcucccucgcA............................................................... 1 1 7y1
......................ucguacguagcagagcagcuAccucgcu............................................................... 1 1 7y1
......................ucguacUuagcagagcagcucccucgcu............................................................... 1 1 7y1
......................ucguacguagcagUgcagcucccucgcu............................................................... 1 1 7y1
......................ucguacguagcagagcagcucccAcgcu............................................................... 1 1 7y1
......................ucguacguagAagagcagcucccucgcu............................................................... 1 1 7y1
......................ucguacguagcagagcagcucccucgcu............................................................... 1342 0 7y1
......................uUguacguagcagagcagcucccucgcu............................................................... 1 1 7y1
......................ucguacguagcagagAagcucccucgcu............................................................... 3 1 7y1
......................ucguacAuagcagagcagcucccucgcu............................................................... 2 1 7y1
```

Star **Mature**

```
gaccugcuucugggucgggguuucguacguagcagagcagcucccucgcugcgaucuauugaaagucagcccucgacacaaggguuuguccgcgcgcgcgcgcgcgcgcgugc
......................ucguacgAagcagagcagcucccucgcu...............................................................   4     1   7y1
......................ucguacguagcagagcagcucccucUcu...............................................................   1     1   7y1
......................ucguacguagcagagcagcuUccucgcu...............................................................   1     1   7y1
......................ucguaUguagcagagcagcucccucgcu...............................................................   1     1   7y1
......................ucguacguagcagagcagcucccucgcG...............................................................   16    1   7y1
......................ucguacguaUcagagcagcucccucgcu...............................................................   1     1   7y1
......................ucguacguagcagagcagcucGcucgcu...............................................................   1     1   7y1
......................ucguacCuagcagagcagcucccucgcu...............................................................   1     1   7y1
......................ucguacguagcagagcagcucAcucgcu...............................................................   1     1   7y1
......................ucguacguagcagagcagAucccucgcu...............................................................   2     1   7y1
......................ucguacguagcagagcagcucccuAgcu...............................................................   3     1   7y1
......................ucguacguagcagagcagcucccucCcu...............................................................   2     1   7y1
......................ucguaAguagcagagcagcucccucgcu...............................................................   1     1   7y1
......................ucguacguagcagagcagcuccAucgcu...............................................................   1     1   7y1
......................Acguacguagcagagcagcucccucgcu...............................................................   11    1   7y1
......................Ncguacguagcagagcagcucccucgcu...............................................................   3     1   7y1
......................uAguacguagcagagcagcucccucgcu...............................................................   2     1   7y1
......................ucguacguagcGgagcagcucccucgcu...............................................................   1     1   7y1
......................ucguacguagcagagcagcuAccucgcug..............................................................   3     1   7y1
......................ucguacguagcagagcagUucccucgcug..............................................................   1     1   7y1
......................uAguacguagcagagcagcucccucgcug..............................................................   3     1   7y1
......................Acguacguagcagagcagcucccucgcug..............................................................   17    1   7y1
......................ucguacguagcagagcagcuccAucgcug..............................................................   4     1   7y1
......................ucguacguagcagagcagcucccucUcug..............................................................   1     1   7y1
......................ucguacguagGagagcagcucccucgcug..............................................................   1     1   7y1
......................ucguacguaUcagagcagcucccucgcug..............................................................   1     1   7y1
......................ucguacguagAagagcagcucccucgcug..............................................................   3     1   7y1
......................ucgAacguagcagagcagcucccucgcug..............................................................   1     1   7y1
......................ucguacguagcagagcagcucccucgcuC..............................................................   1     1   7y1
......................ucguGcguagcagagcagcucccucgcug..............................................................   1     1   7y1
......................ucguacguGgcagagcagcucccucgcug..............................................................   1     1   7y1
......................Gcguacguagcagagcagcucccucgcug..............................................................   3     1   7y1
......................ucguacCuagcagagcagcucccucgcug..............................................................   3     1   7y1
......................uGguacguagcagagcagcucccucgcug..............................................................   2     1   7y1
......................ucguacguaCcagagcagcucccucgcug..............................................................   1     1   7y1
......................ucguacguagcagagcagcuUccucgcug..............................................................   1     1   7y1
......................ucUuacguagcagagcagcucccucgcug..............................................................   1     1   7y1
......................ucguacguagcagagcagcucccAcgcug..............................................................   4     1   7y1
......................ucguaAguagcagagcagcucccucgcug..............................................................   2     1   7y1
......................ucguacguagcagagcagcucAcucgcug..............................................................   3     1   7y1
......................ucguacguagcaCagcagcucccucgcug..............................................................   1     1   7y1
......................ucguacguagcagagcagcucccuUgcug..............................................................   1     1   7y1
......................ucCuacguagcagagcagcucccucgcug..............................................................   3     1   7y1
......................ucguacguagcagagcagcucccucCcug..............................................................   1     1   7y1
......................ucguacguagcagagcagcucccucgcug..............................................................   2333  0   7y1
......................ucguacgAagcagagcagcucccucgcug..............................................................   4     1   7y1
......................Ncguacguagcagagcagcucccucgcug..............................................................   1     1   7y1
......................ucguacguagcagagcagcucccucgcuU..............................................................   6     1   7y1
......................Ccguacguagcagagcagcucccucgcug..............................................................   1     1   7y1
......................ucguacguagcagagcagcCcccucgcug..............................................................   2     1   7y1
......................ucguacguagcagagcagcucccucgcGg..............................................................   8     1   7y1
......................ucguacguagUagagcagcucccucgcug..............................................................   1     1   7y1
......................ucguacguagcagagcagcucccucgAug..............................................................   1     1   7y1
......................ucguacguagcagCgcagcucccucgcug..............................................................   2     1   7y1
.......................cguacguagUagagcagc........................................................................   1     1   7y1
.......................cguacguagcaUagcagc........................................................................   1     1   7y1
.......................cguacguagcagaUcagc........................................................................   2     1   7y1
.......................cguacguaAcagagcagc........................................................................   1     1   7y1
.......................cguacguagcagagcCgc........................................................................   1     1   7y1
.......................cguacguagcagagcagc........................................................................   1761  0   7y1
.......................cguacguaUcagagcagc........................................................................   3     1   7y1
.......................cguacguagcUgagcagc........................................................................   1     1   7y1
.......................cguacguagcGgagcagc........................................................................   2     1   7y1
.......................cguacAuagcagagcagc........................................................................   1     1   7y1
.......................cguacguagcagagAagc........................................................................   2     1   7y1
.......................cgAacguagcagagcagc........................................................................   2     1   7y1
.......................cUuacguagcagagcagc........................................................................   3     1   7y1
.......................cguacguagcagagcaUc........................................................................   1     1   7y1
.......................Gguacguagcagagcagc........................................................................   2     1   7y1
.......................cguacgCagcagagcagc........................................................................   2     1   7y1
```

Star **Mature**

```
gaccugcuucugggucggggguuucguacguagcagagcagcucccucgcugcgaucuauugaaagucagcccucgacacaaggguuuguccgcgcgcgcgcgcgcgcgcgcgugc
```

```
.......................cguacguagcagCgcagc..........................................................................  2     1  7y1
.......................cguacguagAagagcagc..........................................................................  2     1  7y1
.......................cguacgAagcagagcagc..........................................................................  1     1  7y1
.......................cguCcguagcagagcagc..........................................................................  1     1  7y1
.......................cguacguagcagagcGgc..........................................................................  1     1  7y1
.......................cguacNuagcagagcagc..........................................................................  1     1  7y1
.......................cguacguagcaAagcagc..........................................................................  1     1  7y1
.......................cCuacguagcagagcagc..........................................................................  1     1  7y1
.......................cguacguagcagagcagA..........................................................................  2     1  7y1
.......................Aguacguagcagagcagc..........................................................................  6     1  7y1
.......................Nguacguagcagagcagc..........................................................................  2     1  7y1
.......................cAuacguagcagagcagc..........................................................................  1     1  7y1
.......................cguaAguagcagagcagc..........................................................................  1     1  7y1
.......................cCuacguagcagagcagcu.........................................................................  1     1  7y1
.......................Aguacguagcagagcagcu.........................................................................  5     1  7y1
.......................cguacguagUagagcagcu.........................................................................  1     1  7y1
.......................cguacguagcagagGagcu.........................................................................  2     1  7y1
.......................cUuacguagcagagcagcu.........................................................................  5     1  7y1
.......................cguacguagcagagcagcA.........................................................................  1     1  7y1
.......................cgAacguagcagagcagcu.........................................................................  1     1  7y1
.......................cAuacguagcagagcagcu.........................................................................  2     1  7y1
.......................cguacguagcagaAcagcu.........................................................................  1     1  7y1
.......................cguacguagcagaCcagcu.........................................................................  1     1  7y1
.......................cguacUuagcagagcagcu.........................................................................  1     1  7y1
.......................cguacguagcagagcagcu.........................................................................  1200  0  7y1
.......................cguacguagcagagcagAu.........................................................................  3     1  7y1
.......................cgCacguagcagagcagcu.........................................................................  1     1  7y1
.......................cguacguagAagagcagcu.........................................................................  3     1  7y1
.......................cguaAguagcagagcagcu.........................................................................  2     1  7y1
.......................cguacguagcagagcagcG.........................................................................  4     1  7y1
.......................cguacguagcaUagcagcu.........................................................................  1     1  7y1
.......................Gguacguagcagagcagcu.........................................................................  1     1  7y1
.......................cguacguagcagagAagcu.........................................................................  3     1  7y1
.......................cguacguagcagagcaUcu.........................................................................  1     1  7y1
.......................cguacguagcagCgcagcu.........................................................................  1     1  7y1
.......................Nguacguagcagagcagcu.........................................................................  1     1  7y1
.......................cguacguagcagagcaCcu.........................................................................  2     1  7y1
.......................cguacgAagcagagcagcu.........................................................................  3     1  7y1
.......................cguacguagcagagcaUcuc........................................................................  5     1  7y1
.......................cguacguagcagagcaCcuc........................................................................  4     1  7y1
.......................cguacguaAcagagcagcuc........................................................................  1     1  7y1
.......................cguacguagUagagcagcuc........................................................................  1     1  7y1
.......................cguacguagcagagcagUuc........................................................................  1     1  7y1
.......................cguacguagcagagcagcAc........................................................................  5     1  7y1
.......................cguacguagAagagcagcuc........................................................................  22    1  7y1
.......................cguGcguagcagagcagcuc........................................................................  2     1  7y1
.......................cguacguagcagUgcagcuc........................................................................  1     1  7y1
.......................cUuacguagcagagcagcuc........................................................................  5     1  7y1
.......................cguacguagcagagcagcGc........................................................................  1     1  7y1
.......................cguacguaUcagagcagcuc........................................................................  4     1  7y1
.......................cguacguagcagagUagcuc........................................................................  1     1  7y1
.......................cguacguaCcagagcagcuc........................................................................  2     1  7y1
.......................cguacguagcaUagcagcuc........................................................................  3     1  7y1
.......................cguacguagcagagcagcuA........................................................................  8     1  7y1
.......................cguacguagcagagAagcuc........................................................................  29    1  7y1
.......................cguaGguagcagagcagcuc........................................................................  1     1  7y1
.......................cguacguagcagagGagcuc........................................................................  2     1  7y1
.......................cguacguagcagGgcagcuc........................................................................  3     1  7y1
.......................cguacguagcagagcUgcuc........................................................................  1     1  7y1
.......................cgAacguagcagagcagcuc........................................................................  12    1  7y1
.......................Aguacguagcagagcagcuc........................................................................  25    1  7y1
.......................cguacguagGagagcagcuc........................................................................  1     1  7y1
.......................cguacAuagcagagcagcuc........................................................................  4     1  7y1
.......................cguacguagcagagcagcuG........................................................................  1     1  7y1
.......................cguacgAagcagagcagcuc........................................................................  23    1  7y1
.......................cguacguagcaCagcagcuc........................................................................  2     1  7y1
.......................cguacCuagcagagcagcuc........................................................................  2     1  7y1
.......................Nguacguagcagagcagcuc........................................................................  2     1  7y1
.......................cguacguagcagaCcagcuc........................................................................  4     1  7y1
.......................cCuacguagcagagcagcuc........................................................................  11    1  7y1
```

Star **Mature**

```
gaccugcuucugggucgggguuucguacguagcagagcagcucccucgcugcgaucuauugaaagucagcccucgacacaaggguuuguccgcgcgcgcgcgcgcgcgcgcgugc
.......................cguacguagcagagcagcuc........................................................................ 7571 0 7y1
.......................cguacguagcagagcGgcuc........................................................................ 1 1 7y1
.......................cguacgCagcagagcagcuc........................................................................ 2 1 7y1
.......................Gguacguagcagagcagcuc........................................................................ 3 1 7y1
.......................cguaAguagcagagcagcuc........................................................................ 10 1 7y1
.......................cguacUuagcagagcagcuc........................................................................ 5 1 7y1
.......................cguacguagcagagcagAuc........................................................................ 9 1 7y1
.......................Uguacguagcagagcagcuc........................................................................ 1 1 7y1
.......................cguacguagcagCgcagcuc........................................................................ 4 1 7y1
.......................cAuacguagcagagcagcuc........................................................................ 1 1 7y1
.......................cguacguagcagaUcagcuc........................................................................ 4 1 7y1
.......................cguacguagcGgagcagcuc........................................................................ 2 1 7y1
.......................cguacguagcagagcagcAcc....................................................................... 1 1 7y1
.......................cguacguagcagagAagcucc....................................................................... 1 1 7y1
.......................cguacguagcagagcagcuAc....................................................................... 1 1 7y1
.......................cguacguagcagCgcagcucc....................................................................... 1 1 7y1
.......................Aguacguagcagagcagcucc....................................................................... 1 1 7y1
.......................cguacguagcagagcagcucc....................................................................... 434 0 7y1
.......................cUuacguagcagagcagcucc....................................................................... 1 1 7y1
.......................cguacguagcagagNagcucc....................................................................... 1 1 7y1
.......................cguacguagcagaUcagcucc....................................................................... 1 1 7y1
.......................cguacguagcagagGagcucc....................................................................... 1 1 7y1
.......................cguacguaCcagagcagcucc....................................................................... 1 1 7y1
.......................cAuacguagcagagcagcucc....................................................................... 1 1 7y1
.......................cguaAguagcagagcagcucc....................................................................... 1 1 7y1
.......................cguacguagcagagAagcuccc...................................................................... 2 1 7y1
.......................cUuacguagcagagcagcuccc...................................................................... 3 1 7y1
.......................cguacguagcagagcagcAccc...................................................................... 1 1 7y1
.......................cguacguagcagagcagcuccA...................................................................... 2 1 7y1
.......................cguacgAagcagagcagcuccc...................................................................... 1 1 7y1
.......................cguacguagcagagcagcuccc...................................................................... 660 0 7y1
.......................Aguacguagcagagcagcuccc...................................................................... 3 1 7y1
.......................cguacguagcagagcaUcuccc...................................................................... 2 1 7y1
.......................cguacguagAagagcagcuccc...................................................................... 1 1 7y1
.......................cguacguagcagUgcagcuccc...................................................................... 1 1 7y1
.......................cguaAguagcagagcagcuccc...................................................................... 1 1 7y1
.......................cguacguagcagagcagcuGcc...................................................................... 1 1 7y1
.......................cguacguagcagagcagcuAcc...................................................................... 1 1 7y1
.......................cguacguagcagagcaUcucccu..................................................................... 2 1 7y1
.......................cguacguagcagagcagcucccA..................................................................... 1 1 7y1
.......................cguaUguagcagagcagcucccu..................................................................... 1 1 7y1
.......................cguacguagcagagcagcucccG..................................................................... 6 1 7y1
.......................cguacguagcagagcagcuccAu..................................................................... 2 1 7y1
.......................cguacguagcagagcGgcucccu..................................................................... 2 1 7y1
.......................cguacguagcagagcagcuAccu..................................................................... 2 1 7y1
.......................cguacguagcagagAagcucccu..................................................................... 1 1 7y1
.......................cAuacguagcagagcagcucccu..................................................................... 1 1 7y1
.......................cgAacguagcagagcagcucccu..................................................................... 1 1 7y1
.......................cguacguagcagagcagcAcccu..................................................................... 1 1 7y1
.......................cUuacguagcagagcagcucccu..................................................................... 2 1 7y1
.......................cCuacguagcagagcagcucccu..................................................................... 1 1 7y1
.......................cguacguagcagagcagcucccu..................................................................... 943 0 7y1
.......................cguacguagcagaUcagcucccu..................................................................... 1 1 7y1
.......................cguacguagcagagcUgcucccu..................................................................... 1 1 7y1
.......................cguacguagcaUagcagcucccu..................................................................... 1 1 7y1
.......................cguacguagcagagcagcucGcu..................................................................... 1 1 7y1
.......................cguacguagAagagcagcucccu..................................................................... 1 1 7y1
.......................cguacguagcagagcCgcucccu..................................................................... 1 1 7y1
.......................cguaAguagcagagcagcucccu..................................................................... 1 1 7y1
.......................cguacguagcagagcagGucccuc.................................................................... 1 1 7y1
.......................cguacCuagcagagcagcucccuc.................................................................... 2 1 7y1
.......................cguacguagcagagcagcucccAc.................................................................... 2 1 7y1
.......................cguacAuagcagagcagcucccuc.................................................................... 1 1 7y1
.......................cguacgAagcagagcagcucccuc.................................................................... 4 1 7y1
.......................cguacguagcagaUcagcucccuc.................................................................... 2 1 7y1
.......................cguacguagcagagcagcucAcuc.................................................................... 2 1 7y1
.......................Nguacguagcagagcagcucccuc.................................................................... 1 1 7y1
.......................cguacguagcagGgcagcucccuc.................................................................... 1 1 7y1
.......................cUuacguagcagagcagcucccuc.................................................................... 3 1 7y1
.......................cguacguagcagCgcagcucccuc.................................................................... 2 1 7y1
```

Star **Mature**

gaccugcuucugggucgggguuucguacguagcagagcagcucccucgcugcgaucuauugaaagucagcccucgacacaaggguuuguccgcgcgcgcgcgcgcgcgcgugc			
.......................cCuacguagcagagcagcucccuc..	1	1	7y1
.......................cguacguaCcagagcagcucccuc..	1	1	7y1
.......................cguacguagcagagcagcucccuc..	744	0	7y1
.......................cguacguagcagagcagcAcccuc..	1	1	7y1
.......................cguacguagcaCagcagcucccuc..	1	1	7y1
.......................cguacguagcagagAagcucccuc..	4	1	7y1
.......................cguacguagcagaAcagcucccuc..	2	1	7y1
.......................cguacgGagcagagcagcucccuc..	1	1	7y1
.......................cguacguagcagagcagcuAccuc..	1	1	7y1
.......................cguacgAagcagagcagcucccucg...	1	1	7y1
.......................cguacguaUcagagcagcucccucg...	1	1	7y1
.......................cguacguagcagaUcagcucccucg...	1	1	7y1
.......................cguacguagcagGgcagcucccucg...	1	1	7y1
.......................cguacguagcagagcagcucccucg...	297	0	7y1
.......................cguacguagcagagAagcucccucg...	1	1	7y1
.......................cguacUuagcagagcagcucccucg...	1	1	7y1
.......................cUuacguagcagagcagcucccucg...	1	1	7y1
.......................Aguacguagcagagcagcucccucg...	2	1	7y1
.......................cguaAguagcagagcagcucccucgc..	1	1	7y1
.......................cguacguagcagUgcagcucccucgc..	1	1	7y1
.......................cguacCuagcagagcagcucccucgc..	1	1	7y1
.......................cguacguagcagagcagcucccucCc..	1	1	7y1
.......................cguacguagcagagcagAucccucgc..	1	1	7y1
.......................cguacguagcagagcagcucccucgc..	264	0	7y1
.......................cguacguagcagagAagcucccucgc..	1	1	7y1
.......................cguacgAagcagagcagcucccucgc..	1	1	7y1
.......................cguacguagcagagcagAucccucgcu...	1	1	7y1
.......................cguacguagcagagcagcucccGcgcu...	1	1	7y1
.......................cguacguagcaUagcagcucccucgcu...	1	1	7y1
.......................cguacguagAagagcagcucccucgcu...	1	1	7y1
.......................cUuacguagcagagcagcucccucgcu...	3	1	7y1
.......................cguacguagcagagcagcucccucgcu...	407	0	7y1
.......................Aguacguagcagagcagcucccucgcu...	2	1	7y1
.......................cguacgAagcagagcagcucccucgcu...	2	1	7y1
.......................cguaUguagcagagcagcucccucgcu...	264	1	7y1
.......................cguaAguagcagagcagcucccucgcu...	2	1	7y1
.......................cCuacguagcagagcagcucccucgcu...	1	1	7y1
.......................cguacguagcagagcagcucccucgcG...	3	1	7y1
.......................cguacguagcagagcagcuGccucgcu...	1	1	7y1
.......................cAuacguagcagagcagcucccucgcug..	2	1	7y1
.......................cguacguagcagagcagcucAcucgcug..	1	1	7y1
.......................cguacguagcagagcagcucccucgcuC..	1	1	7y1
.......................cguacguaCcagagcagcucccucgcug..	2	1	7y1
.......................Aguacguagcagagcagcucccucgcug..	7	1	7y1
.......................cguacguagcagagcagcucccuUcug..	2	1	7y1
.......................cguacguagcagagcagcucccucgcGg..	8	1	7y1
.......................cCuacguagcagagcagcucccucgcug..	1	1	7y1
.......................cUuacguagcagagcagcucccucgcug..	2	1	7y1
.......................Nguacguagcagagcagcucccucgcug..	2	1	7y1
.......................cguacguagcagagcagcuAccucgcug..	2	1	7y1
.......................cguacguagcGgagcagcucccucgcug..	1	1	7y1
.......................Gguacguagcagagcagcucccucgcug..	1	1	7y1
.......................cgCacguagcagagcagcucccucgcug..	1	1	7y1
.......................cguacguagcagagcagcucccucgcuA..	1	1	7y1
.......................cguacguagcagagcagcucccucgcug..	1992	0	7y1
.......................cguaAguagcagagcagcucccucgcug..	1	1	7y1
.......................cguacguagcagagcagcuccAucgcug..	1	1	7y1
.......................cguacguagcagCgcagcucccucgcug..	1	1	7y1
.......................cguacgAagcagagcagcucccucgcug..	2	1	7y1
.......................cguacUuagcagagcagcucccucgcug..	1	1	7y1
.......................cguacguagcagagAagcucccucgcug..	2	1	7y1
.......................cguacguagcagagcagcucccucgcuU..	1	1	7y1
.......................cguacCuagcagagcagcucccucgcug..	2	1	7y1
.......................cguacguagcagagcagcuGccucgcug..	1	1	7y1
.......................cgGacguagcagagcagcucccucgcug..	1	1	7y1
.......................cguacguagcagagcagcucccucgcAg..	4	1	7y1
.......................cgAacguagcagagcagcucccucgcug..	2	1	7y1
.......................cguacguagcagagcagcucGcucgcug..	1	1	7y1
.......................cguacguagcagagcagcucccuAgcug..	1	1	7y1
.......................cguacguagcaUagcagcucccucgcug..	1	1	7y1

Star **Mature**

```
gaccugcuucugggucgggguuucguacguagcagagcagcucccucgcugcgaucuauugaaagucagcccucgacacaaggguuuguccgcgcgcgcgcgcgcgcgcgugc
.......................cguacguagAagagcagcucccucgcug..............................................................  3    1  7y1
.......................cguacguagcagagcagcucccucgAug..............................................................  2    1  7y1
........................gAacguagcagagcagcu.......................................................................  2    1  7y1
........................guaAguagcagagcagcu.......................................................................  1    1  7y1
........................guacguagcagagcagcu.......................................................................  154  0  7y1
........................Cuacguagcagagcagcuc......................................................................  2    1  7y1
........................guacguagcagagcagAuc......................................................................  1    1  7y1
........................guacguagcagagcagcuc......................................................................  173  0  7y1
........................guacgAagcagagcagcuc......................................................................  1    1  7y1
........................Cuacguagcagagcagcucccucgc................................................................  1    1  7y1
........................guacguagcagagcagcucccucgcu...............................................................  164  0  7y1
........................guacguagcagagcagAucccucgcu...............................................................  2    1  7y1
........................guacguagcagagAagcucccucgcu...............................................................  1    1  7y1
........................Uuacguagcagagcagcucccucgcu...............................................................  1    1  7y1
........................guacguagcagagcagcucccucgcG...............................................................  3    1  7y1
........................guacgAagcagagcagcucccucgcu...............................................................  1    1  7y1
........................guacguaUcagagcagcucccucgcu...............................................................  1    1  7y1
........................guGcguagcagagcagcucccucgcu...............................................................  1    1  7y1
........................guacguagcagagcagGucccucgcu...............................................................  1    1  7y1
........................guacguagcagagcGgcucccucgcug..............................................................  1    1  7y1
........................guaAguagcagagcagcucccucgcug..............................................................  1    1  7y1
........................guacguagcagagcagcucccucgcug..............................................................  61   0  7y1
.........................uacguagcagagcagcuA......................................................................  2    1  7y1
.........................uacguagcagagcaAcuc......................................................................  1    1  7y1
.........................uacguagcagagAagcuc......................................................................  1    1  7y1
.........................uacguagcagagcagcuc......................................................................  616  0  7y1
.........................uacguaUcagagcagcuc......................................................................  2    1  7y1
.........................Aacguagcagagcagcuc......................................................................  15   1  7y1
.........................uaAguagcagagcagcuc......................................................................  1    1  7y1
.........................uacguagcagagGagcuc......................................................................  1    1  7y1
.........................uacguagcagagcagcAc......................................................................  2    1  7y1
.........................uacguagcagagcCgcuc......................................................................  1    1  7y1
.........................uacguagcagagcagcuG......................................................................  1    1  7y1
.........................Gacguagcagagcagcuccc....................................................................  1    1  7y1
.........................uacguagcGgagcagcuccc....................................................................  1    1  7y1
.........................uacguagcagCgcagcuccc....................................................................  1    1  7y1
.........................uacguagcagagcagcAccc....................................................................  1    1  7y1
.........................Aacguagcagagcagcuccc....................................................................  3    1  7y1
.........................uacguagcagagcagcuccc....................................................................  193  0  7y1
.........................uacguagcagagcaCcucccucgcu...............................................................  1    1  7y1
.........................uaAguagcagagcagcucccucgcu...............................................................  1    1  7y1
.........................uacguagcagagcUgcucccucgcu...............................................................  1    1  7y1
.........................uacguagcagagcagcucccucgcu...............................................................  493  0  7y1
.........................Nacguagcagagcagcucccucgcu...............................................................  1    1  7y1
.........................uacguagcagagcagcuAccucgcu...............................................................  1    1  7y1
.........................uacguagcagagAagcucccucgcu...............................................................  2    1  7y1
.........................Aacguagcagagcagcucccucgcu...............................................................  5    1  7y1
.........................uacguagcagagcagcucccucUcu...............................................................  1    1  7y1
.........................uacguagcagagcagcucccucgcG...............................................................  4    1  7y1
.........................uacguagcaCagcagcucccucgcu...............................................................  2    1  7y1
.........................uacguagcagagcagcucccuUgcu...............................................................  1    1  7y1
.........................uacguaCcagagcagcucccucgcu...............................................................  2    1  7y1
.........................uacguagcagagcagcucccucgGu...............................................................  1    1  7y1
.........................uacguagcagagcaAcucccucgcug..............................................................  1    1  7y1
.........................uGcguagcagagcagcucccucgcug..............................................................  1    1  7y1
.........................uacguagcagagcagcucccAcgcug..............................................................  1    1  7y1
.........................uacguagcagagcagcucAcucgcug..............................................................  4    1  7y1
.........................uacguagcagagcagcucccucgcuA..............................................................  1    1  7y1
.........................uacguagcagagcagcucccucgcuC..............................................................  2    1  7y1
.........................uacguGgcagagcagcucccucgcug..............................................................  1    1  7y1
.........................uacgAagcagagcagcucccucgcug..............................................................  2    1  7y1
.........................uacguagcagagcagcAcccucgcug..............................................................  1    1  7y1
.........................uacguagcagagcagcucccuAgcug..............................................................  1    1  7y1
.........................uacguagcagagcagcuccGucgcug..............................................................  1    1  7y1
.........................uacguagcagagcaUcucccucgcug..............................................................  2    1  7y1
.........................uacguagcagagcagcucGcucgcug..............................................................  1    1  7y1
.........................uacguagcagagcagcuAccucgcug..............................................................  2    1  7y1
.........................uacguagcUgagcagcucccucgcug..............................................................  1    1  7y1
.........................uacguagcagagcagcucccucgcGg..............................................................  19   1  7y1
.........................uacguagcagagcaNcucccucgcug..............................................................  1    1  7y1
```

Star **Mature**

gaccugcuucugggucgggguuucguacguagcagagcagcucccucgcugcgaucuauugaaagucagcccucgacacaaggguuuguccgcgcgcgcgcgcgcgcgcgcgugc			
.........................uacguagcagagcagcucccucgcAg..	1	1	7y1
.........................uacguagcagagcagcucccucgAug..	2	1	7y1
.........................Aacguagcagagcagcucccucgcug..	17	1	7y1
.........................uacguUgcagagcagcucccucgcug..	1	1	7y1
.........................uacguagcagagcagcucccucgcCg..	1	1	7y1
.........................uUcguagcagagcagcucccucgcug..	1	1	7y1
.........................uacguagcagagcagcucccucgcug..	1768	0	7y1
.........................uacguaCcagagcagcucccucgcug..	1	1	7y1
.........................uacguagAagagcagcucccucgcug..	3	1	7y1
.........................uaAguagcagagcagcucccucgcug..	5	1	7y1
.........................uacguagcagagcCgcucccucgcug..	1	1	7y1
.........................uaGguagcagagcagcucccucgcug..	3	1	7y1
.........................uacguagcagagcagcucccucgcuU..	1	1	7y1
.........................Gacguagcagagcagcucccucgcug..	2	1	7y1
.........................uacguaUcagagcagcucccucgcug..	1	1	7y1
..........................acguagcagaUcagcucccucgcu...	1	1	7y1
..........................acguagcagagcagcucccucgcu...	416	0	7y1
..........................acguagcagagcagcucccucgcG...	9	1	7y1
..........................Gcguagcagagcagcucccucgcu...	5	1	7y1
..........................acguagcagagcagAucccucgcu...	1	1	7y1
..........................acguagcGgagcagcucccucgcu...	2	1	7y1
..........................acguagcagagcagcucccAcgcu...	1	1	7y1
..........................acguagcagagcaUcucccucgcu...	3	1	7y1
..........................acguaCcagagcagcucccucgcu...	1	1	7y1
..........................acgAagcagagcagcucccucgcu...	1	1	7y1
..........................acguaUcagagcagcucccucgcu...	1	1	7y1
..........................acguagcagagAagcucccucgcu...	1	1	7y1
..........................aAguagcagagcagcucccucgcu...	2	1	7y1
..........................acguagcagagcagcuAccucgcu...	1	1	7y1
..........................acguagAagagcagcucccucgcu...	2	1	7y1
..........................acguagcagagcCgcucccucgcu...	1	1	7y1
..........................acguagcaUagcagcucccucgcug..	1	1	7y1
..........................acguagAagagcagcucccucgcug..	2	1	7y1
..........................acguagcagaUcagcucccucgcug..	2	1	7y1
..........................acguagcagagAagcucccucgcug..	1	1	7y1
..........................acguagcagagcagcucccucgcGg..	5	1	7y1
..........................acguagcagagcGgcucccucgcug..	1	1	7y1
..........................acguagcagagcagcucccucgcug..	537	0	7y1
..........................acgAagcagagcagcucccucgcug..	1	1	7y1
..........................acCuagcagagcagcucccucgcug..	1	1	7y1
..........................acguagcagagcagcucccucgcuC..	1	1	7y1
..........................acguagcagagcagcuccAucgcug..	2	1	7y1
..........................Gcguagcagagcagcucccucgcug..	1	1	7y1
...........................cguagcagagcagcuccc..	199	0	7y1
...........................cguagcagagcagcucGc..	1	1	7y1
...........................cguagcagagcagcucAcu...	1	1	7y1
...........................cguagAagagcagcucccu...	3	1	7y1
...........................cguagcagagcagcuAccu...	2	1	7y1
...........................cguagcagaUcagcucccu...	1	1	7y1
...........................cgGagcagagcagcucccu...	1	1	7y1
...........................cguagcagagcagcucccu...	204	0	7y1
...........................cguagcagaCcagcucccu...	1	1	7y1
...........................cguaCcagagcagcucccucgcug..	1	1	7y1
...........................cCuagcagagcagcucccucgcug..	1	1	7y1
...........................cguagcagagcagcuUccucgcug..	1	1	7y1
...........................cguagcagagcagcucccucAcug..	1	1	7y1
...........................cguagcagCgcagcucccucgcug..	1	1	7y1
...........................cguagcagagcagcucccucgAug..	1	1	7y1
...........................cguagcagagcagcucccucgcuU..	2	1	7y1
...........................cguagcagagcagcucccucgcug..	541	0	7y1
...........................cguaUcagagcagcucccucgcug..	1	1	7y1
...........................cguagcagagcagAucccucgcug..	1	1	7y1
...........................Nguagcagagcagcucccucgcug..	2	1	7y1
...........................cguagcagagcagcucccAcgcug..	1	1	7y1
...........................cgAagcagagcagcucccucgcug..	1	1	7y1
...........................Gguagcagagcagcucccucgcug..	1	1	7y1
...........................cguagcagagcagcuGccucgcug..	1	1	7y1
...........................cguagcagagcagcAcccucgcug..	1	1	7y1
............................guagcagagcagcucccucgc..	2	0	7y1
............................guagcagagcagcucccucgcug..	73	0	7y1

Star **Mature**

```
gaccugcuucugggucgggguuucguacguagcagagcagcucccucgcugcgaucuauugaaagucagcccucgacacaaggguuuguccgcgcgcgcgcgcgcgcgcgugc
..............................agcagagcagcucGcucgcug..............................................................   1     1   7y1
..............................aUcagagcagcucccucgcug..............................................................   1     1   7y1
..............................agcagagcagcuccAucgcug..............................................................   2     1   7y1
..............................agcagagcagcucccucgcug..............................................................   273   0   7y1
..............................agcagagAagcucccucgcug..............................................................   1     1   7y1
..............................agcagagcagcuAccucgcug..............................................................   1     1   7y1
..............................aAcagagcagcucccucgcug..............................................................   1     1   7y1
..............................agcagagcagcucAcucgcug..............................................................   1     1   7y1
..............................agcagagcagcucccucgAug..............................................................   1     1   7y1
................................cagagcaUcucccucgcu...............................................................   1     1   7y1
................................cagagcagcucccucgcu...............................................................   129   0   7y1
................................cagCgcagcucccucgcu...............................................................   1     1   7y1
................................cagagcagcucAcucgcu...............................................................   1     1   7y1
................................cagagcagcucccucgcG...............................................................   11    1   7y1
................................cagagcagcuccGucgcu...............................................................   1     1   7y1
................................cagagcagcAcccucgcu...............................................................   1     1   7y1
....................................gcagcuUccucgcugcgaucu........................................................   1     1   7y1
....................................gcagcucAcucgcugcgaucu........................................................   1     1   7y1
....................................gcagcuAccucgcugcgaucu........................................................   3     1   7y1
....................................gAagcucccucgcugcgaucu........................................................   1     1   7y1
....................................gcagcucccucgcuCcgaucu........................................................   1     1   7y1
....................................gcagcucccucgcugcgaucu........................................................   394   0   7y1
....................................gcagcucccucCcugcgaucu........................................................   1     1   7y1
....................................gcagcucccucgAugcgaucu........................................................   1     1   7y1
....................................gcagcucccucgcugcgaucA........................................................   1     1   7y1
....................................gcagcucccucgcAgcgaucu........................................................   2     1   7y1
....................................gcagcucccucgcugcgGucu........................................................   1     1   7y1
....................................gcagAucccucgcugcgaucu........................................................   1     1   7y1
....................................gcagcucccucgGugcgaucu........................................................   1     1   7y1
....................................gcagcucccucgcCgcgaucu........................................................   1     1   7y1
....................................gcagcucccucgcugcgaucuauugaaagu...............................................   224   0   7y1
....................................Ccagcucccucgcugcgaucuauugaaagu...............................................   1     1   7y1
....................................gcagcucccucgcugcgaucuauugaaagG...............................................   1     1   7y1
....................................gcagcucccuAgcugcgaucuauugaaagu...............................................   1     1   7y1
....................................gcagcucccucUcugcgaucuauugaaagu...............................................   1     1   7y1
....................................gcagcucccucgcugcgaucuaAugaaagu...............................................   1     1   7y1
....................................gcagcuUccucgcugcgaucuauugaaagu...............................................   1     1   7y1
....................................Ncagcucccucgcugcgaucuauugaaaguca.............................................   1     1   7y1
....................................gcagcucccucgcugcgaucuauugaaaguca.............................................   182   0   7y1
....................................gcagcucccucgcugcgaucAauugaaaguca.............................................   1     1   7y1
....................................gcagcucAcucgcugcgaucuauugaaaguca.............................................   2     1   7y1
....................................gcagcucccuAgcugcgaucuauugaaaguca.............................................   1     1   7y1
....................................gcagcucccucgcugcgauAuauugaaaguca.............................................   1     1   7y1
....................................gcagcucccucgcugcgaucuauAgaaaguca.............................................   1     1   7y1
....................................gcagGucccucgcugcgaucuauugaaaguca.............................................   1     1   7y1
....................................gcagcucccuUgcugcgaucuauugaaaguca.............................................   1     1   7y1
....................................gcagcucccucgcugAgaucuauugaaaguca.............................................   2     1   7y1
....................................gcagcuAccucgcugcgaucuauugaaaguca.............................................   3     1   7y1
.....................................cagcucccucgcugcgaucAauugaaagucag............................................   1     1   7y1
.....................................cagcucccucgcugcgaucuaGugaaagucag............................................   1     1   7y1
.....................................cagcucccucgcugcgaucuauugaaagucag............................................   188   0   7y1
.....................................cagcucccucgcugcgaAcuauugaaagucag............................................   1     1   7y1
.....................................cagcAcccucgcugcgaucuauugaaagucag............................................   2     1   7y1
.....................................cagcucccucgcugcgaucuauugaaagAcag............................................   1     1   7y1
.....................................cagcuAccucgcugcgaucuauugaaagucag............................................   1     1   7y1
.....................................cagcucccucgcugAgaucuauugaaagucag............................................   1     1   7y1
.....................................cagUucccucgcugcgaucuauugaaagucag............................................   1     1   7y1
.....................................cagcucccuAgcugcgaucuauugaaagucag............................................   1     1   7y1
.....................................cagcucccucAcugcgaucuauugaaagucag............................................   1     1   7y1
.....................................cagcuccAucgcugcgaucuauugaaagucag............................................   2     1   7y1
.....................................Aagcucccucgcugcgaucuauugaaagucag............................................   2     1   7y1
.....................................cagcuUccucgcugcgaucuauugaaagucag............................................   1     1   7y1
......................................agcucccucgcugcNaucu........................................................   1     1   7y1
......................................agcucccucgcugcgUucu........................................................   1     1   7y1
......................................agcucccucgcugcgaucG........................................................   1     1   7y1
......................................agcuccAucgcugcgaucu........................................................   1     1   7y1
......................................agcucccucgcugcgaucu........................................................   330   0   7y1
......................................agcuAccucgcugcgaucu........................................................   1     1   7y1
......................................agAucccucgcugcgaucu........................................................   1     1   7y1
......................................agcucccuAgcugcgaucu........................................................   1     1   7y1
```

Star **Mature**

gaccugcuucugggucgggguuucguacguagcagagcagcucccucgcugcgaucuauugaaagucagcccucgacacaaggguuuguccgcgcgcgcgcgcgcgcgcgugc			
......................................agcucccucgcugAgaucu..	1	1	7y1
......................................Cgcucccucgcugcgaucu..	2	1	7y1
......................................agcucAcucgcugcgaucu..	1	1	7y1
......................................Cgcucccucgcugcgaucuauugaaaguc..	1	1	7y1
......................................agcucccucgcAgcgaucuauugaaaguc..	1	1	7y1
......................................agcucccucgcugcgaucuauugaaaguc..	185	0	7y1
......................................agcucAcucgcugcgaucuauugaaaguc..	2	1	7y1
......................................agcCcccucgcugcgaucuauugaaaguc..	1	1	7y1
......................................agcuAccucgcugcgaucuauugaaaguc..	2	1	7y1
......................................agcuccAucgcugcgaucuauugaaaguc..	1	1	7y1
......................................agcucccucgcugAgaucuauugaaaguc..	1	1	7y1
......................................agcucccucgcugcgaucuauugaaagucag..	1	0	7y1
....gcucccucgcugcgaucG..	1	1	7y1
....Ccucccucgcugcgaucu..	1	1	7y1
....gcucccucgcugcgaucu..	194	0	7y1
....gcucccAcgcugcgaucu..	1	1	7y1
....gcucccucUcugcgaucu..	1	1	7y1
....gcucccucgAugcgaucu..	1	1	7y1
....gcuAccucgcugcgaucu..	1	1	7y1
....gcucAcucgcugcgaucuauug..	3	1	7y1
....gcuAccucgcugcgaucuauug..	1	1	7y1
....gcuccAucgcugcgaucuauug..	2	1	7y1
....Ccucccucgcugcgaucuauug..	1	1	7y1
....gcucccucgcugcgaucuauAg..	1	1	7y1
....gcucccucgcugcgaucuauug..	515	0	7y1
....gcucccucgcugcgaucAauug..	1	1	7y1
....gcucUcucgcugcgaucuauug..	1	1	7y1
....gcucccuAgcugcgaucuauug..	1	1	7y1
....gcucccucgcugcgaucuauCg..	1	1	7y1
....gcucccucgcugcgaucuauuU..	1	1	7y1
....gcucccucgcugcgUucuauug..	1	1	7y1
....gcucccucgcugcgaucuauugaGag..	1	1	7y1
....gcucccucCcugcgaucuauugaaag..	1	1	7y1
....gcucccucgcugcgaucuUuugaaag..	1	1	7y1
....gcucccucgcugcgaucuauugaaGg..	1	1	7y1
....gcucAcucgcugcgaucuauugaaag..	2	1	7y1
....gcucccucgcugcgaucuauGgaaag..	1	1	7y1
....gcucccucgcugcgauAuauugaaag..	1	1	7y1
....gcucccucgcugcUaucuauugaaag..	1	1	7y1
....gcuUccucgcugcgaucuauugaaag..	1	1	7y1
....gcucccucAcugcgaucuauugaaag..	1	1	7y1
....gcucccucgcGgcgaucuauugaaag..	1	1	7y1
....gcucccucgcugcgaucuauugGaag..	1	1	7y1
....gcucccucgcuCcgaucuauugaaag..	1	1	7y1
....gcuAccucgcugcgaucuauugaaag..	2	1	7y1
....gcucccucgcugAgaucuauugaaag..	1	1	7y1
....gNucccucgcugcgaucuauugaaag..	1	1	7y1
....gcucccuAgcugcgaucuauugaaag..	1	1	7y1
....gcucccucgcugcgaucuauugaaaC..	2	1	7y1
....gcucccucgAugcgaucuauugaaag..	1	1	7y1
....gcucccucgcugcgaucuauugaaag..	1199	0	7y1
....Ncucccucgcugcgaucuauugaaag..	1	1	7y1
....gcuccAucgcugcgaucuauugaaag..	1	1	7y1
....gcucccucgcAgcgaucuauugaaag..	1	1	7y1
....gcucccAcgcugcgaucuauugaaag..	2	1	7y1
....Ccucccucgcugcgaucuauugaaag..	2	1	7y1
....gAucccucgcugcgaucuauugaaag..	3	1	7y1
....gcucccucgcugcgaAcuauugaaagu...	1	1	7y1
....gcucccucgcCgcgaucuauugaaagu...	1	1	7y1
....gcucccucgcAgcgaucuauugaaagu...	1	1	7y1
....gcucAcucgcugcgaucuauugaaagu...	2	1	7y1
....gcucccucgcugcgaucCauugaaagu...	1	1	7y1
....gcucccucgcuCcgaucuauugaaagu...	1	1	7y1
....gcucccucgcugcgaucAauugaaagu...	1	1	7y1
....gcuccAucgcugcgaucuauugaaagu...	1	1	7y1
....gcucccucgcugcgaucuauAgaaagu...	1	1	7y1
....gcucccucgcugcgaucuauugaaaUu...	1	1	7y1
....gcucccucgcugcgaucuauugaaagu...	906	0	7y1
....gcuAccucgcugcgaucuauugaaagu...	1	1	7y1
....gcucccucgcugcUaucuauugaaagu...	1	1	7y1

Star **Mature**

```
gaccugcuucugggucgggguuucguacguagcagagcagcucccucgcugcgaucuauugaaagucagcccucgacacaaggguuuguccgcgcgcgcgcgcgcgcgcgugc
.......................................gcucccucgcugcgauGuauugaaagu...............................................	1	1	7y1
.......................................gcucccuAgcugcgaucuauugaaagu...............................................	2	1	7y1
.......................................gcucccucgcugcgaucuauugaaagA...............................................	1	1	7y1
.......................................Ccucccucgcugcgaucuauugaaagu...............................................	2	1	7y1
.......................................Ucucccucgcugcgaucuauugaaagu...............................................	1	1	7y1
.......................................gcucccucgcugcgaucuaGugaaagu...............................................	1	1	7y1
.......................................gcucccucgcugcgaucuauugaaagG...............................................	1	1	7y1
.......................................gcucccucgAugcgaucuauugaaagu...............................................	2	1	7y1
.......................................gcucccucgcugcgaucuauugaaaguU..............................................	1	1	7y1
.......................................gcuGccucgcugcgaucuauugaaaguc..............................................	1	1	7y1
.......................................gcucccucgcugcgaucuauugaaaguc..............................................	229	0	7y1
.......................................gcucccucgcugAgaucuauugaaaguc..............................................	1	1	7y1
.......................................gcucAcucgcugcgaucuauugaaaguc..............................................	1	1	7y1
.......................................gcuccAucgcugcgaucuauugaaaguc..............................................	1	1	7y1
.......................................Ucucccucgcugcgaucuauugaaaguc..............................................	1	1	7y1
........................................cucccucgcugcgaucuaAugaa..................................................	1	1	7y1
........................................cucccAcgcugcgaucuauugaa..................................................	1	1	7y1
........................................cucccucgcugAgaucuauugaa..................................................	2	1	7y1
........................................cucccucgcugcgauAuauugaa..................................................	1	1	7y1
........................................cucccucgcAgcgaucuauugaa..................................................	1	1	7y1
........................................cucccucgcugcgaAcuauugaa..................................................	1	1	7y1
........................................cucccucgcugcCaucuauugaa..................................................	1	1	7y1
........................................cucccucgcugcgaucuauugaa..................................................	144	0	7y1
........................................cucccucgcugcgaucuauugaaa.................................................	244	0	7y1
........................................cucccucgcugcgaucuauGgaaa.................................................	1	1	7y1
........................................cucccucgcugAgaucuauugaaa.................................................	1	1	7y1
........................................cuAccucgcugcgaucuauugaaa.................................................	3	1	7y1
........................................Aucccucgcugcgaucuauugaaa.................................................	2	1	7y1
........................................cucccucgcuCcgaucuauugaaa.................................................	1	1	7y1
........................................cucccuAgcugcgaucuauugaaag................................................	1	1	7y1
........................................cucccucgcuCcgaucuauugaaag................................................	1	1	7y1
........................................cucccucgcuUcgaucuauugaaag................................................	1	1	7y1
........................................cuccAucgcugcgaucuauugaaag................................................	1	1	7y1
........................................cucAcucgcugcgaucuauugaaag................................................	1	1	7y1
........................................cucccucgcugcgaucuauugaaag................................................	185	0	7y1
........................................cucccucgcuAcgaucuauugaaagu...............................................	1	1	7y1
........................................Aucccucgcugcgaucuauugaaagu...............................................	6	1	7y1
........................................cucccucgAugcgaucuauugaaagu...............................................	2	1	7y1
........................................cucAcucgcugcgaucuauugaaagu...............................................	1	1	7y1
........................................cucccucgcugcgaucuauAgaaagu...............................................	1	1	7y1
........................................cucccucgcuCcgaucuauugaaagu...............................................	2	1	7y1
........................................cucccucCcugcgaucuauugaaagu...............................................	2	1	7y1
........................................cucccucgcAgcgaucuauugaaagu...............................................	2	1	7y1
........................................cucccucgcugcUaucuauugaaagu...............................................	1	1	7y1
........................................cucccucgcugcgaucuauugaaaUu...............................................	1	1	7y1
........................................cucccucgcugcgaucuauugaaagA...............................................	5	1	7y1
........................................cucccucUcugcgaucuauugaaagu...............................................	3	1	7y1
........................................cucccucgcugcgaucuaAugaaagu...............................................	1	1	7y1
........................................cuccUucgcugcgaucuauugaaagu...............................................	1	1	7y1
........................................cucccucgcugcgCucuauugaaagu...............................................	1	1	7y1
........................................cucccucgcugcgaucuauugaaagG...............................................	1	1	7y1
........................................cuAccucgcugcgaucuauugaaagu...............................................	9	1	7y1
........................................cucccucgcugAgaucuauugaaagu...............................................	2	1	7y1
........................................cucccuAgcugcgaucuauugaaagu...............................................	4	1	7y1
........................................cucccucgcugcgaucuauugGaagu...............................................	1	1	7y1
........................................cucccucgcugcgaucuauuAaaagu...............................................	1	1	7y1
........................................cAcccucgcugcgaucuauugaaagu...............................................	7	1	7y1
........................................cucccucgcugcgaucuauugaUagu...............................................	1	1	7y1
........................................cucccucgcugcgaucAauugaaagu...............................................	2	1	7y1
........................................cucccucgcugcgaucuauugaaagu...............................................	1893	0	7y1
........................................cucccucgcugcgaucuauugaaaCu...............................................	1	1	7y1
........................................cucccAcgcugcgaucuauugaaagu...............................................	5	1	7y1
........................................cucccucgcugcgaucuauugaUaguc..............................................	1	1	7y1
........................................cucccucgcugcUaucuauugaaaguc..............................................	3	1	7y1
........................................cucccucgcugcgaucuauAgaaaguc..............................................	8	1	7y1
........................................cucccucgcugcgaucuauuCaaaguc..............................................	1	1	7y1
........................................cucccucgcuCcgaucuauugaaaguc..............................................	3	1	7y1
........................................cuAccucgcugcgaucuauugaaaguc..............................................	10	1	7y1
........................................cucccucgcugcgaAcuauugaaaguc..............................................	3	1	7y1
........................................Nucccucgcugcgaucuauugaaaguc..............................................	3	1	7y1
```

Star **Mature**

gaccugcuucugggucgggguuucguacguagcagagcagcucccucgcugcgaucuauugaaagucagcccucgacacaaggguuuguccgcgcgcgcgcgcgcgcgugc			
....cucccucgcugcgaucuauugaaaguA..	4	1	7y1
....cucccuUgcugcgaucuauugaaaguc..	1	1	7y1
....cuGccucgcugcgaucuauugaaaguc..	1	1	7y1
....cucUcucgcugcgaucuauugaaaguc..	2	1	7y1
....cucccucgcugAgaucuauugaaaguc..	12	1	7y1
....cucAcucgcugcgaucuauugaaaguc..	5	1	7y1
....cucccucgcugcgaucuauugGaaguc..	2	1	7y1
....cucccucgAugcgaucuauugaaaguc..	1	1	7y1
....cucccucgcuUcgaucuauugaaaguc..	6	1	7y1
....cucccucgcugcgaGcuauugaaaguc..	2	1	7y1
....cucccucUcugcgaucuauugaaaguc..	7	1	7y1
....cucccucgcugcgaucuaCugaaaguc..	1	1	7y1
....cucccucgcugcgaucuauugaaagAc..	3	1	7y1
....cucccucgcugcgaucuauugaaaAuc..	1	1	7y1
....cucccucgcCgcgaucuauugaaaguc..	1	1	7y1
....cAcccucgcugcgaucuauugaaaguc..	18	1	7y1
....cucccucgcugcgauAuauugaaaguc..	3	1	7y1
....cucccucgcugcgaucuaGugaaaguc..	1	1	7y1
....cucccucgcugcgaucuauuAaaaguc..	1	1	7y1
....cucccAcgcugcgaucuauugaaaguc..	9	1	7y1
....cucccucgGugcgaucuauugaaaguc..	1	1	7y1
....cucccucgcAgcgaucuauugaaaguc..	3	1	7y1
....cucccucgcugcgaucuaAugaaaguc..	7	1	7y1
....cucccucgcugcgaucuauugaaaCuc..	1	1	7y1
....cucccucgcugcgaucuauuUaaaguc..	2	1	7y1
....cucccucgcugcgaucuauugaaaguc..	4185	0	7y1
....cucccucgcuAcgaucuauugaaaguc..	4	1	7y1
....cucccucgcugcgaucuauugaaaguG..	1	1	7y1
....cucccucCcugcgaucuauugaaaguc..	4	1	7y1
....Aucccucgcugcgaucuauugaaaguc..	8	1	7y1
....cucccucgcugcgCucuauugaaaguc..	1	1	7y1
....cucccucgcugcgaucuGuugaaaguc..	1	1	7y1
....cucccucgcugcgaucuauugaaaUuc..	1	1	7y1
....cucccuAgcugcgaucuauugaaaguc..	6	1	7y1
....cuccAucgcugcgaucuauugaaaguc..	2	1	7y1
....cucccucgcugcgaucuauuCaaaguca...	1	1	7y1
....cucccucgcugcgaucuauAgaaaguca...	3	1	7y1
....cucccucgcugcgaucAauugaaaguca...	1	1	7y1
....cucccucgcAgcgaucuauugaaaguca...	5	1	7y1
....cucccAcgcugcgaucuauugaaaguca...	4	1	7y1
....cucccucgcugcgaucuauugaaaguca...	1280	0	7y1
....cucccucgcugcgaucuauugaGaguca...	1	1	7y1
....cAcccucgcugcgaucuauugaaaguca...	1	1	7y1
....cuAccucgcugcgaucuauugaaaguca...	4	1	7y1
....cucccuAgcugcgaucuauugaaaguca...	1	1	7y1
....cucccucgcugcgaucuauugaaagucG...	2	1	7y1
....cucAcucgcugcgaucuauugaaaguca...	1	1	7y1
....Aucccucgcugcgaucuauugaaaguca...	3	1	7y1
....cucccucgcugcgaucuauugaaaguGa...	1	1	7y1
....cucccucgcuCcgaucuauugaaaguca...	1	1	7y1
....cucccucgcugAgaucuauugaaaguca...	2	1	7y1
....cucccucgcugcgauAuauugaaaguca...	2	1	7y1
....cuAccucgcugcgaucuauugaaagucag..	2	1	7y1
....cucccAcgcugcgaucuauugaaagucag..	1	1	7y1
....cucccucgcAgcgaucuauugaaagucag..	1	1	7y1
....Aucccucgcugcgaucuauugaaagucag..	1	1	7y1
....cucccuAgcugcgaucuauugaaagucag..	3	1	7y1
....cucAcucgcugcgaucuauugaaagucag..	1	1	7y1
....cucccucgcugcgaucuauugaaaCucag..	1	1	7y1
....Gucccucgcugcgaucuauugaaagucag..	1	1	7y1
....cucccucgcugcgaucuauugaaagGcag..	1	1	7y1
....cucccucgcugAgaucuauugaaagucag..	4	1	7y1
....cucccucgcugcgaucuauugaaagucag..	378	0	7y1
....cucccucgcugcgaucuauAgaaagucag..	1	1	7y1
....cuccGucgcugcgaucuauugaaagucagcc..	1	1	7y1
....cucccucgAugcgaucuauugaaagucagcc..	1	1	7y1
....cucccucgcugcgaucuauugaaaguAagcc..	1	1	7y1
....Nucccucgcugcgaucuauugaaagucagcc..	1	1	7y1
....cuAccucgcugcgaucuauugaaagucagcc..	1	1	7y1
....cucccucgcugcgaucuauugaaagAcagcc..	1	1	7y1

```
  Star                                                                   Mature
gaccugcuucugggucggggguuucguacguagcagagcagcucccucgcugcgaucuauugaaagucagcccucgacacaaggguuuguccgcgcgcgcgcgcgcgcgcgcgugc
........................................cucccucgcugcgaucuauugaaagucagcc............................................	172	0	7y1
.........................................ucccucgcugcUaucuau........................................................	1	1	7y1
.........................................ucccucgcugcgaAcuau........................................................	1	1	7y1
.........................................Acccucgcugcgaucuau........................................................	1	1	7y1
.........................................ucccucgcugcgaCcuau........................................................	1	1	7y1
.........................................ucccucgcugcgaucuau........................................................	200	0	7y1
.........................................Ncccucgcugcgaucuau........................................................	1	1	7y1
.........................................ucccucgcugcgaucuaG........................................................	1	1	7y1
.........................................uAccucgcugcgaucuauu.......................................................	1	1	7y1
.........................................ucccucgcugcgaucuauu.......................................................	189	0	7y1
.........................................uUccucgcugcgaucuauu.......................................................	1	1	7y1
.........................................Acccucgcugcgaucuauu.......................................................	1	1	7y1
.........................................ucAcucgcugcgaucuauu.......................................................	1	1	7y1
.........................................Gcccucgcugcgaucuauu.......................................................	1	1	7y1
.........................................ucccucgcugcUaucuauu.......................................................	2	1	7y1
.........................................ucccucgcugcgaAcuauug......................................................	1	1	7y1
.........................................ucccucgcugcUaucuauug......................................................	1	1	7y1
.........................................ucccucgcugcgaucuauug......................................................	266	0	7y1
.........................................Acccucgcugcgaucuauug......................................................	4	1	7y1
.........................................ucccucgcugGgaucuauug......................................................	1	1	7y1
.........................................ucccuAgcugcgaucuauug......................................................	1	1	7y1
.........................................Acccucgcugcgaucuauugaaa...................................................	2	1	7y1
.........................................ucccucgcugcgaucuauugaaa...................................................	212	0	7y1
.........................................ucccucgcugcgaucuauuUaaa...................................................	1	1	7y1
.........................................ucccucUcugcgaucuauugaaa...................................................	1	1	7y1
.........................................Gcccucgcugcgaucuauugaaa...................................................	1	1	7y1
.........................................Ccccucgcugcgaucuauugaaa...................................................	1	1	7y1
.........................................ucccuAgcugcgaucuauugaaag..................................................	1	1	7y1
.........................................ucccucgcugcgauAuauugaaag..................................................	1	1	7y1
.........................................ucccucgcugcgaucAauugaaag..................................................	1	1	7y1
.........................................Acccucgcugcgaucuauugaaag..................................................	7	1	7y1
.........................................ucccucgcugcgaucuauAgaaag..................................................	1	1	7y1
.........................................ucccucgcugcgauUuauugaaag..................................................	1	1	7y1
.........................................ucccucgcugcgaucuauuAaaag..................................................	1	1	7y1
.........................................ucccucgcuUcgaucuauugaaag..................................................	1	1	7y1
.........................................ucAcucgcugcgaucuauugaaag..................................................	3	1	7y1
.........................................Ncccucgcugcgaucuauugaaag..................................................	3	1	7y1
.........................................Gcccucgcugcgaucuauugaaag..................................................	1	1	7y1
.........................................uAccucgcugcgaucuauugaaag..................................................	2	1	7y1
.........................................ucccucgcugcgaucuauugCaag..................................................	1	1	7y1
.........................................ucccucgcugcgaucuauugaaag..................................................	935	0	7y1
.........................................ucccucgcugcgaucuauugGaag..................................................	1	1	7y1
.........................................ucccucgcugAgaucuauugaaag..................................................	1	1	7y1
.........................................ucccucUcugcgaucuauugaaag..................................................	2	1	7y1
.........................................ucccucgcAgcgaucuauugaaag..................................................	2	1	7y1
.........................................ucccucgcuCcgaucuauugaaag..................................................	3	1	7y1
.........................................ucccucgcugcgaucuauugaaagu.................................................	682	0	7y1
.........................................ucccucgcAgcgaucuauugaaagu.................................................	2	1	7y1
.........................................ucccucgcugAgaucuauugaaagu.................................................	4	1	7y1
.........................................Acccucgcugcgaucuauugaaagu.................................................	12	1	7y1
.........................................ucccucUcugcgaucuauugaaagu.................................................	1	1	7y1
.........................................uccAucgcugcgaucuauugaaagu.................................................	1	1	7y1
.........................................ucAcucgcugcgaucuauugaaagu.................................................	1	1	7y1
.........................................ucccucgcugcgauAuauugaaagu.................................................	2	1	7y1
.........................................ucccucgcugcUaucuauugaaagu.................................................	1	1	7y1
.........................................ucccucgcugcgaucAauugaaagu.................................................	1	1	7y1
.........................................ucccucgcugcgaucuauugaaagA.................................................	1	1	7y1
.........................................Gcccucgcugcgaucuauugaaagu.................................................	1	1	7y1
.........................................uAccucgcugcgaucuauugaaagu.................................................	1	1	7y1
.........................................ucccucgcugcgaucuaAugaaagu.................................................	1	1	7y1
.........................................ucccucgcuUcgaucuauugaaagu.................................................	1	1	7y1
.........................................Gcccucgcugcgaucuauugaaaguc................................................	2	1	7y1
.........................................ucccucgcugcgaucuauugaaaAuc................................................	1	1	7y1
.........................................ucccucgcugcgaucuaAugaaaguc................................................	1	1	7y1
.........................................ucccucgcuCcgaucuauugaaaguc................................................	2	1	7y1
.........................................ucccucgcugcgaucuauugaaaCuc................................................	3	1	7y1
.........................................ucccucgcugcgaucAauugaaaguc................................................	3	1	7y1
.........................................ucccucgcAgcgaucuauugaaaguc................................................	4	1	7y1
.........................................ucccucgcugcgaucuauAgaaaguc................................................	2	1	7y1
.........................................ucAcucgcugcgaucuauugaaaguc................................................	10	1	7y1
```

Star **Mature**

```
gaccugcuucugggucgggguuucguacguagcagagcagcucccucgcugcgaucuauugaaagucagccccucgacacaaggguuuguccgcgcgcgcgcgcgcgcgcgugc
.........................................ucccucgcugcgaucuauugGaaguc...............................................  1     1  7y1
.........................................uAccucgcugcgaucuauugaaaguc...............................................  3     1  7y1
.........................................ucccucgcugcUaucuauugaaaguc...............................................  1     1  7y1
.........................................ucccucgAugcgaucuauugaaaguc...............................................  3     1  7y1
.........................................ucccucgcugcgaucuauugaaaguc...............................................  2692  0  7y1
.........................................ucccuAgcugcgaucuauugaaaguc...............................................  7     1  7y1
.........................................ucccucgcugcgaucuauugaaaguA...............................................  1     1  7y1
.........................................ucccucgcGgcgaucuauugaaaguc...............................................  1     1  7y1
.........................................ucccucgcuUcgaucuauugaaaguc...............................................  2     1  7y1
.........................................ucccucAcugcgaucuauugaaaguc...............................................  1     1  7y1
.........................................ucccucgcugcgaucuGuugaaaguc...............................................  2     1  7y1
.........................................Acccucgcugcgaucuauugaaaguc...............................................  38    1  7y1
.........................................ucccucgcugAgaucuauugaaaguc...............................................  2     1  7y1
.........................................ucccAcgcugcgaucuauugaaaguc...............................................  1     1  7y1
.........................................ucccucgcugcCaucuauugaaaguc...............................................  3     1  7y1
.........................................ucccucgcugcgaucuauugaaagAc...............................................  2     1  7y1
.........................................ucccucUcugcgaucuauugaaaguc...............................................  2     1  7y1
.........................................ucccucgcugcgauAuauugaaaguc...............................................  4     1  7y1
.........................................uccAucgcugcgaucuauugaaaguc...............................................  2     1  7y1
.........................................Ncccucgcugcgaucuauugaaaguc...............................................  1     1  7y1
.........................................ucccucgGugcgaucuauugaaaguca..............................................  1     1  7y1
.........................................ucccucgcugcgaucuauugaaaguca..............................................  586   0  7y1
.........................................Ncccucgcugcgaucuauugaaaguca..............................................  2     1  7y1
.........................................ucccucgcugcgaucCauugaaaguca..............................................  1     1  7y1
.........................................ucccucgcugcgauAuauugaaaguca..............................................  1     1  7y1
.........................................Gcccucgcugcgaucuauugaaaguca..............................................  1     1  7y1
.........................................ucccucgcugAgaucuauugaaaguca..............................................  1     1  7y1
.........................................ucccucgcugcgaucuauugaaagucU..............................................  1     1  7y1
.........................................ucccucUcugcgaucuauugaaaguca..............................................  1     1  7y1
.........................................ucccucgcuAcgaucuauugaaaguca..............................................  1     1  7y1
.........................................ucccucgcuCcgaucuauugaaaguca..............................................  1     1  7y1
.........................................uccAucgcugcgaucuauugaaaguca..............................................  1     1  7y1
.........................................ucccucgcugcCaucuauugaaaguca..............................................  1     1  7y1
.........................................ucccucgAugcgaucuauugaaaguca..............................................  2     1  7y1
.........................................ucccucCcugcgaucuauugaaaguca..............................................  1     1  7y1
.........................................Acccucgcugcgaucuauugaaaguca..............................................  6     1  7y1
.........................................ucccucgcuUcgaucuauugaaagucag.............................................  1     1  7y1
.........................................ucAcucgcugcgaucuauugaaagucag.............................................  1     1  7y1
.........................................ucccucgcugcgaucuauAgaaagucag.............................................  1     1  7y1
.........................................ucccucgcugcgaucuauugaaagucag.............................................  159   0  7y1
.........................................Acccucgcugcgaucuauugaaagucag.............................................  2     1  7y1
.........................................ucccucgcugcgUucuauugaaagucag.............................................  1     1  7y1
.........................................ucccAcgcugcgaucuauugaaagucag.............................................  1     1  7y1
..........................................cccucgcugcgaucuauu......................................................  172   0  7y1
..........................................cccucgcugcgaucuaAu......................................................  1     1  7y1
..........................................Gccucgcugcgaucuauu......................................................  1     1  7y1
..........................................ccAucgcugcgaucuauu......................................................  1     1  7y1
..........................................cAcucgcugcgaucuauu......................................................  1     1  7y1
..........................................cccucgcugcCaucuauu......................................................  2     1  7y1
..........................................cccucgcAgcgaucuauu......................................................  1     1  7y1
..........................................cccucgcugcgaucuauuga....................................................  157   0  7y1
..........................................Accucgcugcgaucuauuga....................................................  1     1  7y1
..........................................ccAucgcugcgaucuauuga....................................................  1     1  7y1
..........................................cccucgAugcgaucuauuga....................................................  1     1  7y1
..........................................cAcucgcugcgaucuauuga....................................................  2     1  7y1
..........................................cccucgcAgcgaucuauuga....................................................  1     1  7y1
..........................................cccucgcugcgaucuauugG....................................................  1     1  7y1
..........................................Ncccucgcugcgaucuauuga....................................................  1     1  7y1
..........................................cccucgcugcgaucuauuAaa...................................................  1     1  7y1
..........................................cAcucgcugcgaucuauugaaa..................................................  3     1  7y1
..........................................cccucgAugcgaucuauugaaa..................................................  3     1  7y1
..........................................cccucgcugcUaucuauugaaa..................................................  1     1  7y1
..........................................cccucgcugcgaucuaAugaaa..................................................  3     1  7y1
..........................................cccucgcuCcgaucuauugaaa..................................................  2     1  7y1
..........................................cccucgcugcgaucuauAgaaa..................................................  1     1  7y1
..........................................cccucgcuUcgaucuauugaaa..................................................  2     1  7y1
..........................................cccucgcugcCaucuauugaaa..................................................  1     1  7y1
..........................................cUcucgcugcgaucuauugaaa..................................................  1     1  7y1
..........................................ccGucgcugcgaucuauugaaa..................................................  1     1  7y1
..........................................cccucgcugcgaucuauugaaa..................................................  1120  0  7y1
```

Star **Mature**

```
gaccugcuucugggucgggguuucguacguagcagagcagcucccucgcugcgaucuauugaaagucagcccucgacacaaggguuuguccgcgcgcgcgcgcgcgcgcgugc
..........................................cccucgcugcgaAcuauugaaa.................................................   2     1    7y1
..........................................Accucgcugcgaucuauugaaa.................................................   3     1    7y1
..........................................cccucgcugAgaucuauugaaa.................................................   3     1    7y1
..........................................cccucgcugcgauUuauugaaa.................................................   1     1    7y1
..........................................cccucgcugcgaucuauuCaaa.................................................   1     1    7y1
..........................................cccuAgcugcgaucuauugaaa.................................................   1     1    7y1
..........................................cccAcgcugcgaucuauugaaa.................................................   1     1    7y1
..........................................cccucgcugcgauAuauugaaa.................................................   1     1    7y1
..........................................cccucgcugcgaucGauugaaa.................................................   1     1    7y1
..........................................Nccucgcugcgaucuauugaaa.................................................   1     1    7y1
..........................................cccucgcCgcgaucuauugaaa.................................................   1     1    7y1
..........................................cccucgcAgcgaucuauugaaa.................................................   3     1    7y1
..........................................cccucgcugcgaucuaCugaaag................................................   1     1    7y1
..........................................cccucgcAgcgaucuauugaaag................................................   1     1    7y1
..........................................cccucgcugcgaucuauAgaaag................................................   1     1    7y1
..........................................cccucgcuUcgaucuauugaaag................................................   1     1    7y1
..........................................Gccucgcugcgaucuauugaaag................................................   1     1    7y1
..........................................cccAcgcugcgaucuauugaaag................................................   2     1    7y1
..........................................cAcucgcugcgaucuauugaaag................................................   6     1    7y1
..........................................cccucgcugcCaucuauugaaag................................................   3     1    7y1
..........................................cccucgcugcgauAuauugaaag................................................   1     1    7y1
..........................................cccucgcugAgaucuauugaaag................................................   2     1    7y1
..........................................ccUucgcugcgaucuauugaaag................................................   1     1    7y1
..........................................ccGucgcugcgaucuauugaaag................................................   1     1    7y1
..........................................cccucgcugcgaucuaAugaaag................................................   3     1    7y1
..........................................cccucCcugcgaucuauugaaag................................................   1     1    7y1
..........................................Nccucgcugcgaucuauugaaag................................................   1     1    7y1
..........................................cccucgcugcgaucuauugaaag................................................   1154  0    7y1
..........................................cNcucgcugcgaucuauugaaag................................................   1     1    7y1
..........................................cccucgcugcgaucuauuAaaag................................................   1     1    7y1
..........................................Accucgcugcgaucuauugaaag................................................   1     1    7y1
..........................................cccucgcugcgaucuauugUaag................................................   1     1    7y1
..........................................cccucgGugcgaucuauugaaag................................................   1     1    7y1
..........................................cccucgcugcgaucuauGgaaag................................................   1     1    7y1
..........................................ccAucgcugcgaucuauugaaag................................................   5     1    7y1
..........................................ccAucgcugcgaucuauugaaagu...............................................   4     1    7y1
..........................................cccucgAugcgaucuauugaaagu...............................................   5     1    7y1
..........................................cccucgcugcgaucuauuCaaagu...............................................   1     1    7y1
..........................................cccucgcugcgaucCauugaaagu...............................................   1     1    7y1
..........................................cccucgcugcgauAuauugaaagu...............................................   2     1    7y1
..........................................cccucgcugcgaucAauugaaagu...............................................   1     1    7y1
..........................................cAcucgcugcgaucuauugaaagu...............................................   7     1    7y1
..........................................cccucgcugAgaucuauugaaagu...............................................   6     1    7y1
..........................................cccucgcugcCaucuauugaaagu...............................................   1     1    7y1
..........................................Accucgcugcgaucuauugaaagu...............................................   2     1    7y1
..........................................cccucgcugcgaucuauugaaagu...............................................   1043  0    7y1
..........................................cccucUcugcgaucuauugaaagu...............................................   2     1    7y1
..........................................cccucgcugcgaucuauugaaagG...............................................   11    1    7y1
..........................................cccAcgcugcgaucuauugaaagu...............................................   1     1    7y1
..........................................cccucgcuCcgaucuauugaaagu...............................................   1     1    7y1
..........................................cccucgcugcgaucuauugaaagA...............................................   2     1    7y1
..........................................cccucgcugcgaAcuauugaaagu...............................................   1     1    7y1
..........................................cccucgcugcgaucuauAgaaagu...............................................   1     1    7y1
..........................................Nccucgcugcgaucuauugaaaguc..............................................   3     1    7y1
..........................................cccucgcugcgaucuauugaaaguA..............................................   1     1    7y1
..........................................cccucgcugcUaucuauugaaaguc..............................................   1     1    7y1
..........................................cccAcgcugcgaucuauugaaaguc..............................................   1     1    7y1
..........................................cccucgcuUcgaucuauugaaaguc..............................................   1     1    7y1
..........................................cccucgcugcgCucuauugaaaguc..............................................   1     1    7y1
..........................................cccucgcugcgaucuauugaGaguc..............................................   2     1    7y1
..........................................cccucgcugcgaucuaAugaaaguc..............................................   2     1    7y1
..........................................cccucgcugcgaucuauugaaaUuc..............................................   1     1    7y1
..........................................cccuAgcugcgaucuauugaaaguc..............................................   1     1    7y1
..........................................cccucgcugcgaucAauugaaaguc..............................................   3     1    7y1
..........................................cccucgcugcgaucuauugaaaCuc..............................................   1     1    7y1
..........................................cccucgcugAgaucuauugaaaguc..............................................   6     1    7y1
..........................................cNcucgcugcgaucuauugaaaguc..............................................   1     1    7y1
..........................................cccucgcCgcgaucuauugaaaguc..............................................   1     1    7y1
..........................................ccGucgcugcgaucuauugaaaguc..............................................   1     1    7y1
..........................................cccucgcugcgauAuauugaaaguc..............................................   2     1    7y1
```

Star **Mature**

```
gaccugcuucugggucgggguuucguacguagcagagcagcucccucgcugcgaucuauugaaagucagcccucgacacaaggguuuguccgcgcgcgcgcgcgcgcgcgugc
..........................................cccucgcugcgaAcuauugaaaguc..............................................   2      1   7y1
..........................................cccucgUugcgaucuauugaaaguc..............................................   1      1   7y1
..........................................cccucgcAgcgaucuauugaaaguc..............................................   1      1   7y1
..........................................cAcucgcugcgaucuauugaaaguc..............................................   7      1   7y1
..........................................Accucgcugcgaucuauugaaaguc..............................................   7      1   7y1
..........................................cccucgcuCcgaucuauugaaaguc..............................................   2      1   7y1
..........................................cccucUcugcgaucuauugaaaguc..............................................   1      1   7y1
..........................................cccucgcugcgaucuauugaaaguc..............................................   1855   0   7y1
..........................................cccucgcugcgaucuauugaaagAc..............................................   2      1   7y1
..........................................cccucgcugcgaucuauuUaaaguc..............................................   1      1   7y1
..........................................cccucgAugcgaucuauugaaaguc..............................................   8      1   7y1
..........................................ccAucgcugcgaucuauugaaaguc..............................................   8      1   7y1
..........................................cccucgcugcgaucuauAgaaaguc..............................................   1      1   7y1
..........................................Uccucgcugcgaucuauugaaaguc..............................................   2      1   7y1
..........................................cccucgcugcgaucuauuAaaaguc..............................................   1      1   7y1
..........................................cccucgcugcgaucuauCgaaaguc..............................................   1      1   7y1
..........................................cccucgcugGgaucuauugaaaguc..............................................   1      1   7y1
..........................................cccucgcugcgaucuauugaaaguAa.............................................   1      1   7y1
..........................................cccuAgcugcgaucuauugaaaguca.............................................   3      1   7y1
..........................................cccucgcugAgaucuauugaaaguca.............................................   2      1   7y1
..........................................ccAucgcugcgaucuauugaaaguca.............................................   2      1   7y1
..........................................cccuUgcugcgaucuauugaaaguca.............................................   2      1   7y1
..........................................Nccucgcugcgaucuauugaaaguca.............................................   3      1   7y1
..........................................cccucgcugcgaucuauugaaagAca.............................................   3      1   7y1
..........................................Accucgcugcgaucuauugaaaguca.............................................   5      1   7y1
..........................................cccucgcugcgauAuauugaaaguca.............................................   2      1   7y1
..........................................cccucUcugcgaucuauugaaaguca.............................................   2      1   7y1
..........................................cccucgcugcgaAcuauugaaaguca.............................................   1      1   7y1
..........................................cccucgcugcgaucuauugaaaguca.............................................   592    0   7y1
..........................................cccAcgcugcgaucuauugaaaguca.............................................   1      1   7y1
..........................................cccucAcugcgaucuauugaaaguca.............................................   1      1   7y1
..........................................cccucgcAgcgaucuauugaaaguca.............................................   2      1   7y1
..........................................cccucgcugcgaucuauugaaaUuca.............................................   1      1   7y1
..........................................cccucgcugcUaucuauugaaaguca.............................................   1      1   7y1
...........................................ccucgAugcgaucuauug....................................................   1      1   7y1
...........................................cAucgcugcgaucuauug....................................................   2      1   7y1
...........................................ccucgcCgcgaucuauug....................................................   1      1   7y1
...........................................ccucgcugcgaucuauug....................................................   209    0   7y1
...........................................ccucgcuUcgaucuauug....................................................   1      1   7y1
...........................................ccucgcugcgaucuauuC....................................................   1      1   7y1
...........................................ccucgcGgcgaucuauug....................................................   1      1   7y1
...........................................Ncucgcugcgaucuauug....................................................   2      1   7y1
...........................................ccucgcugcgaucGauugaa..................................................   1      1   7y1
...........................................ccucgcugcUaucuauugaa..................................................   2      1   7y1
...........................................ccucgcugcgaucuauAgaa..................................................   1      1   7y1
...........................................ccucgcugcgaucuauugaa..................................................   602    0   7y1
...........................................Ncucgcugcgaucuauugaa..................................................   1      1   7y1
...........................................ccuAgcugcgaucuauugaa..................................................   1      1   7y1
...........................................cAucgcugcgaucuauugaa..................................................   3      1   7y1
...........................................ccAcgcugcgaucuauugaa..................................................   1      1   7y1
...........................................ccucgcAgcgaucuauugaa..................................................   2      1   7y1
...........................................ccucCcugcgaucuauugaa..................................................   1      1   7y1
...........................................ccucgcugcgaucuauuCaa..................................................   1      1   7y1
...........................................ccucgcugcgaucuauuAaa..................................................   1      1   7y1
...........................................Acucgcugcgaucuauugaa..................................................   1      1   7y1
...........................................ccucgcugcgauAuauugaaa.................................................   4      1   7y1
...........................................Acucgcugcgaucuauugaaa.................................................   6      1   7y1
...........................................ccuAgcugcgaucuauugaaa.................................................   1      1   7y1
...........................................ccucgAugcgaucuauugaaa.................................................   6      1   7y1
...........................................ccucgcugcgaucuaAugaaa.................................................   1      1   7y1
...........................................ccucgcAgcgaucuauugaaa.................................................   9      1   7y1
...........................................ccucgcugcUaucuauugaaa.................................................   5      1   7y1
...........................................ccucgcugAgaucuauugaaa.................................................   1      1   7y1
...........................................cAucgcugcgaucuauugaaa.................................................   2      1   7y1
...........................................Gcucgcugcgaucuauugaaa.................................................   1      1   7y1
...........................................ccucgcugcgaucuauugaGa.................................................   1      1   7y1
...........................................ccucgcuUcgaucuauugaaa.................................................   1      1   7y1
...........................................ccucgcugcgaucAauugaaa.................................................   2      1   7y1
...........................................ccucgcugcgaucuauugaaa.................................................   1604   0   7y1
...........................................ccucgcugcgaucuauugaUa.................................................   1      1   7y1
```

Star **Mature**

gaccugcuucugggucgggguuucguacguagcagagcagcucccucgcugcgaucuauugaaagucagcccucgacacaagguuuguccgcgcgcgcgcgcgcgcgcgugc			
....ccucgcugcgUucuauugaaa..	1	1	7y1
....ccucgcugAgaucuauugaaag...	5	1	7y1
....ccucgcugcgaucuauCgaaag...	1	1	7y1
....ccuGgcugcgaucuauugaaag...	1	1	7y1
....ccucgcugcCaucuauugaaag...	2	1	7y1
....Acucgcugcgaucuauugaaag...	2	1	7y1
....ccucgcugcgaAcuauugaaag...	1	1	7y1
....ccucgcugcgaucAauugaaag...	1	1	7y1
....ccucgcuCcgaucuauugaaag...	1	1	7y1
....cAucgcugcgaucuauugaaag...	4	1	7y1
....ccucgcugcgaucuauugaaag...	1408	0	7y1
....ccucgcugcgaucuauugaaaU...	1	1	7y1
....ccucgcugcgaucuauugaaGg...	1	1	7y1
....ccAcgcugcgaucuauugaaag...	2	1	7y1
....ccuAgcugcgaucuauugaaag...	1	1	7y1
....ccucUcugcgaucuauugaaag...	1	1	7y1
....ccucgAugcgaucuauugaaag...	2	1	7y1
....ccucgcugcgaucCauugaaag...	1	1	7y1
....ccucgcugcgauAuauugaaag...	2	1	7y1
....ccucgcugcgaucuaAugaaag...	1	1	7y1
....ccucCcugcgaucuauugaaag...	2	1	7y1
....ccucgcAgcgaucuauugaaag...	3	1	7y1
....Ncucgcugcgaucuauugaaag...	1	1	7y1
....Gcucgcugcgaucuauugaaag...	2	1	7y1
....ccucgcuUcgaucuauugaaag...	1	1	7y1
....ccucgcugcgaucuauAgaaag...	2	1	7y1
....ccucgcuAcgaucuauugaaag...	1	1	7y1
....ccucUcugcgaucuauugaaagu..	1	1	7y1
....ccucgcugcgaucuauugaaagu..	1291	0	7y1
....ccucgcugcgaucuauAgaaagu..	1	1	7y1
....ccuAgcugcgaucuauugaaagu..	1	1	7y1
....Gcucgcugcgaucuauugaaagu..	1	1	7y1
....ccucgcugcgaucuauugaaaUu..	1	1	7y1
....ccucgcuUcgaucuauugaaagu..	1	1	7y1
....ccucgcugcgaucuauugaaagG..	1	1	7y1
....ccucgcugcgaucuaAugaaagu..	1	1	7y1
....ccucgcugcgaucAauugaaagu..	1	1	7y1
....cUucgcugcgaucuauugaaagu..	1	1	7y1
....ccucgcugcUaucuauugaaagu..	2	1	7y1
....ccucgcCgcgaucuauugaaagu..	1	1	7y1
....ccAcgcugcgaucuauugaaagu..	3	1	7y1
....ccucgAugcgaucuauugaaagu..	2	1	7y1
....ccucgcugcgaAcuauugaaagu..	1	1	7y1
....ccucgcugAgaucuauugaaagu..	6	1	7y1
....ccucgcuCcgaucuauugaaagu..	1	1	7y1
....Ncucgcugcgaucuauugaaagu..	1	1	7y1
....ccucgcugcCaucuauugaaagu..	1	1	7y1
....ccucgcugcgauAuauugaaagu..	1	1	7y1
....ccucgcugcgaucuauugaGagu..	1	1	7y1
....Acucgcugcgaucuauugaaagu..	2	1	7y1
....ccucgcAgcgaucuauugaaagu..	1	1	7y1
....cAucgcugcgaucuauugaaagu..	2	1	7y1
....ccucAcugcgaucuauugaaagu..	2	1	7y1
....ccucgcugcgaucuauugaaagA..	3	1	7y1
....ccucgcugcgauGuauugaaagu..	1	1	7y1
....ccuGgcugcgaucuauugaaagu..	1	1	7y1
....ccucgcugcgaucuauugaaagAc...	4	1	7y1
....ccGcgcugcgaucuauugaaaguc...	1	1	7y1
....ccucgcugcgaucuUuugaaaguc...	1	1	7y1
....ccuAgcugcgaucuauugaaaguc...	4	1	7y1
....ccucgcugcgaucuauAgaaaguc...	4	1	7y1
....ccucgcugcgaucuauuCaaaguc...	1	1	7y1
....ccucgcugAgaucuauugaaaguc...	9	1	7y1
....ccucgcugcUaucuauugaaaguc...	2	1	7y1
....ccucgcugcgaGcuauugaaaguc...	1	1	7y1
....ccucgcugcgaucuauCgaaaguc...	3	1	7y1
....ccucCcugcgaucuauugaaaguc...	2	1	7y1
....ccucgcugcgaucuauugaaaguc...	4155	0	7y1
....ccucgcuUcgaucuauugaaaguc...	5	1	7y1
....ccucgNugcgaucuauugaaaguc...	1	1	7y1

Star Mature

```
gaccugcuucugggucgggguuucguacguagcagagcagcucccucgcugcgaucuauugaaagucagcccucgacacaaggguuuguccgcgcgcgcgcgcgcgcgcgcgugc
...........................................ccucgcugcgaucuaAugaaaguc................................................	3	1	7y1
...........................................ccAcgcugcgaucuauugaaaguc................................................	2	1	7y1
...........................................ccucgcugcgaucuauuUaaaguc................................................	1	1	7y1
...........................................ccucgcugcgaucCauugaaaguc................................................	1	1	7y1
...........................................ccucgcugcgauAuauugaaaguc................................................	7	1	7y1
...........................................ccucgcugcgUucuauugaaaguc................................................	1	1	7y1
...........................................ccucgcugcgaucuauugaGaguc................................................	1	1	7y1
...........................................ccucgcugcgaAcuauugaaaguc................................................	1	1	7y1
...........................................Gcucgcugcgaucuauugaaaguc................................................	2	1	7y1
...........................................ccucgcuCcgaucuauugaaaguc................................................	1	1	7y1
...........................................ccCcgcugcgaucuauugaaaguc................................................	1	1	7y1
...........................................Acucgcugcgaucuauugaaaguc................................................	7	1	7y1
...........................................ccucgcugcCaucuauugaaaguc................................................	4	1	7y1
...........................................ccucgcugcgaucuauugaaaCuc................................................	5	1	7y1
...........................................ccucgcugcgaucuauGgaaaguc................................................	1	1	7y1
...........................................ccucgcugcgaucuauugaaaUuc................................................	1	1	7y1
...........................................ccucgcugcgaucuauugGaaguc................................................	1	1	7y1
...........................................ccucgcAgcgaucuauugaaaguc................................................	16	1	7y1
...........................................ccucgcugcgaucAauugaaaguc................................................	3	1	7y1
...........................................ccucgcCgcgaucuauugaaaguc................................................	1	1	7y1
...........................................ccucgAugcgaucuauugaaaguc................................................	6	1	7y1
...........................................cAucgcugcgaucuauugaaaguc................................................	9	1	7y1
...........................................ccucgcugcgaucuauugaaGguc................................................	1	1	7y1
...........................................ccucgcugcgGucuauugaaaguc................................................	1	1	7y1
...........................................ccucUcugcgaucuauugaaaguc................................................	3	1	7y1
...........................................Ncucgcugcgaucuauugaaaguc................................................	1	1	7y1
...........................................ccucgcugcgaucuauugaaaguA................................................	2	1	7y1
...........................................ccucgcugcgaucuauuAaaaguc................................................	1	1	7y1
...........................................cAucgcugcgaucuauugaaaguca...............................................	2	1	7y1
...........................................Ncucgcugcgaucuauugaaaguca...............................................	1	1	7y1
...........................................ccucgcAgcgaucuauugaaaguca...............................................	1	1	7y1
...........................................ccucgcugAgaucuauugaaaguca...............................................	2	1	7y1
...........................................Acucgcugcgaucuauugaaaguca...............................................	3	1	7y1
...........................................Gcucgcugcgaucuauugaaaguca...............................................	1	1	7y1
...........................................ccucgcugcgaucuauugaaagAca...............................................	1	1	7y1
...........................................ccucgcugcgaucuauugaaaCuca...............................................	1	1	7y1
...........................................ccucgcugcgaucuauugaaaAuca...............................................	1	1	7y1
...........................................ccucgcugcgaucuauugaaaguca...............................................	761	0	7y1
...........................................ccucgcuUcgaucuauugaaaguca...............................................	1	1	7y1
...........................................ccucUcugcgaucuauugaaaguca...............................................	1	1	7y1
...........................................ccucgAugcgaucuauugaaaguca...............................................	1	1	7y1
...........................................ccucgcugcgauAuauugaaaguca...............................................	1	1	7y1
...........................................ccucgcugcgaucuaAugaaaguca...............................................	1	1	7y1
...........................................ccucgcugcgaucuauugaaaUuca...............................................	1	1	7y1
...........................................ccucgcugcgaucuauugaaagucG...............................................	1	1	7y1
...........................................Acucgcugcgaucuauugaaagucag.............................................	1	1	7y1
...........................................ccucgcuCcgaucuauugaaagucag.............................................	1	1	7y1
...........................................cAucgcugcgaucuauugaaagucag.............................................	1	1	7y1
...........................................ccucgcugcgaucuauAgaaagucag.............................................	1	1	7y1
...........................................ccucgcugcUaucuauugaaagucag.............................................	1	1	7y1
...........................................Ncucgcugcgaucuauugaaagucag.............................................	1	1	7y1
...........................................ccucgcugcgaucAauugaaagucag.............................................	1	1	7y1
...........................................ccucgcAgcgaucuauugaaagucag.............................................	2	1	7y1
...........................................ccucAcugcgaucuauugaaagucag.............................................	1	1	7y1
...........................................ccucgcugAgaucuauugaaagucag.............................................	1	1	7y1
...........................................ccucgcugcgaucuauugaaagucag.............................................	163	0	7y1
...........................................ccucgcugcgaucuauugaaagucagUcc..........................................	1	1	7y1
...........................................ccucgcugcgaucuauugaaagucagUccu.........................................	253	1	7y1
............................................cucgAugcgaucuauugaaa...................................................	1	1	7y1
............................................cucgcugcgaucuauAgaaa...................................................	2	1	7y1
............................................cucgcugcgauAuauugaaa...................................................	1	1	7y1
............................................cucgcugcgaucuauugaaa...................................................	413	0	7y1
............................................cucUcugcgaucuauugaaa...................................................	2	1	7y1
............................................cucgcugAgaucuauugaaa...................................................	2	1	7y1
............................................cucgcugcUaucuauugaaa...................................................	1	1	7y1
............................................Aucgcugcgaucuauugaaa...................................................	2	1	7y1
............................................cuAgcugcgaucuauugaaa...................................................	3	1	7y1
............................................cucgcugcCaucuauugaaa...................................................	1	1	7y1
............................................cAcgcugcgaucuauugaaa...................................................	2	1	7y1
............................................cuUgcugcgaucuauugaaag..................................................	1	1	7y1
```

Star **Mature**

gaccugcuucugggucggggguucguacguagcagagcagcucccucgcugcgaucuauugaaagucagcccucgacacaaggguuuguccgcgcgcgcgcgcgcgcgcgugc			
....cucgcugcUaucuauugaaag...	2	1	7y1
....cucgcugcgaucuauugUaag...	1	1	7y1
....cAcgcugcgaucuauugaaag...	12	1	7y1
....cucAcugcgaucuauugaaag...	2	1	7y1
....Gucgcugcgaucuauugaaag...	4	1	7y1
....cucgcugcgaucuauuUaaag...	1	1	7y1
....cucgcAgcgaucuauugaaag...	12	1	7y1
....cucgAugcgaucuauugaaag...	3	1	7y1
....cuAgcugcgaucuauugaaag...	10	1	7y1
....cucgcugcgaucuauAgaaag...	2	1	7y1
....cucgcugcgaucAauugaaag...	1	1	7y1
....cucgcugcgaucuauugaaaC...	2	1	7y1
....cucgcugcgaucuauugaaag...	2182	0	7y1
....cucgcugcgaucuauugGaag...	1	1	7y1
....cucCcugcgaucuauugaaag...	1	1	7y1
....cucgcugcgaucuaAugaaag...	1	1	7y1
....cucgcugcgauUuauugaaag...	1	1	7y1
....cucgcugcgaucuauugaGag...	167	1	7y1
....cucgcugAgaucuauugaaag...	8	1	7y1
....cucgcugcgaucuauugaaaU...	2	1	7y1
....cucgcugcgaAcuauugaaag...	2	1	7y1
....Aucgcugcgaucuauugaaag...	2	1	7y1
....cucgcugcgauAuauugaaag...	6	1	7y1
....cucgcuUcgaucuauugaaag...	2	1	7y1
....cucgcugcgaucuauGgaaag...	1	1	7y1
....cucgcugGgaucuauugaaag...	2	1	7y1
....cucgcuCcgaucuauugaaagu..	1	1	7y1
....cucgcCgcgaucuauugaaagu..	1	1	7y1
....cucgcugcgauAuauugaaagu..	1	1	7y1
....cAcgcugcgaucuauugaaagu..	7	1	7y1
....cucgcugcCaucuauugaaagu..	1	1	7y1
....cucgcugcgaucuauugaaagu..	1949	0	7y1
....cucUcugcgaucuauugaaagu..	1	1	7y1
....cucgcuUcgaucuauugaaagu..	1	1	7y1
....cucgcNgcgaucuauugaaagu..	1	1	7y1
....cucgcugcgaucuUuugaaagu..	1	1	7y1
....cucgcugcUaucuauugaaagu..	2	1	7y1
....cucgUugcgaucuauugaaagu..	1	1	7y1
....cucgcugcgaucuauugaaagG..	1	1	7y1
....cucgcugcgaucCauugaaagu..	1	1	7y1
....cucgcugcgaucuauugaaaUu..	1	1	7y1
....cuAgcugcgaucuauugaaagu..	4	1	7y1
....cucgcAgcgaucuauugaaagu..	4	1	7y1
....cucgcugcgaucAauugaaagu..	1	1	7y1
....cucgcugcgaucuauuUaaagu..	1	1	7y1
....cucgcugcgaAcuauugaaagu..	3	1	7y1
....Nucgcugcgaucuauugaaagu..	1	1	7y1
....cucgcugcgaucuauAgaaagu..	2	1	7y1
....cucgcugcgaucuauuCaaagu..	1	1	7y1
....cucgcugAgaucuauugaaagu..	3	1	7y1
....Aucgcugcgaucuauugaaagu..	6	1	7y1
....cucgcugcgauGuauugaaagu..	1	1	7y1
....cNcgcugcgaucuauugaaagu..	1	1	7y1
....cucgGugcgaucuauugaaaguc...	1	1	7y1
....cucgcuAcgaucuauugaaaguc...	1	1	7y1
....cAcgcugcgaucuauugaaaguc...	7	1	7y1
....cucgcuUcgaucuauugaaaguc...	1	1	7y1
....cucgcugcgaucuauuUaaaguc...	1	1	7y1
....cucgcugcgaucuauAgaaaguc...	1	1	7y1
....Nucgcugcgaucuauugaaaguc...	1	1	7y1
....Gucgcugcgaucuauugaaaguc...	1	1	7y1
....cucgcugcgaucuaAugaaaguc...	5	1	7y1
....cucgcugcgaucuauugaaaCuc...	1	1	7y1
....cucCcugcgaucuauugaaaguc...	2	1	7y1
....cuAgcugcgaucuauugaaaguc...	5	1	7y1
....cucgcugcgaCcuauugaaaguc...	1	1	7y1
....cucgcugcUaucuauugaaaguc...	1	1	7y1
....cucgAugcgaucuauugaaaguc...	3	1	7y1
....cucgcugcgaucuaCugaaaguc...	1	1	7y1
....cucgcugcgaGcuauugaaaguc...	1	1	7y1

Star Mature

```
gaccugcuucugggucggggguucguacguagcagagcagcucccucgcugcgaucuauugaaagucagcccucgacacaaggguuuguccgcgcgcgcgcgcgcgcgcgugc
...........................................cucgcugcgaucuauugaaaguc...............................................   2329   0   7y1
...........................................cucgcugcgGucuauugaaaguc...............................................   1      1   7y1
...........................................cucUcugcgaucuauugaaaguc...............................................   1      1   7y1
...........................................cuGgcugcgaucuauugaaaguc...............................................   2      1   7y1
...........................................cucgcugcgaucuauuCaaaguc...............................................   4      1   7y1
...........................................cucgcAgcgaucuauugaaaguc...............................................   12     1   7y1
...........................................cucgcugAgaucuauugaaaguc...............................................   8      1   7y1
...........................................cucgcuCcgaucuauugaaaguc...............................................   2      1   7y1
...........................................cucgcugcgauAuauugaaaguc...............................................   4      1   7y1
...........................................Aucgcugcgaucuauugaaaguc...............................................   10     1   7y1
...........................................cucgcugcgaucuauugaaaguA...............................................   2      1   7y1
...........................................cucgcugcgaucuauugaaaguca..............................................   238    0   7y1
...........................................cucgcugcgaAcuauugaaaguca..............................................   1      1   7y1
...........................................cucgcugAgaucuauugaaaguca..............................................   1      1   7y1
...........................................Aucgcugcgaucuauugaaaguca..............................................   1      1   7y1
...........................................cucgcugcgaucuauugaaaguAa..............................................   3      1   7y1
...........................................cuAgcugcgaucuauugaaaguca..............................................   1      1   7y1
............................................ucgcAgcgaucuauugaa...................................................   1      1   7y1
............................................ucgcugAgaucuauugaa...................................................   1      1   7y1
............................................Acgcugcgaucuauugaa...................................................   3      1   7y1
............................................ucgcugcgaucuauAgaa...................................................   1      1   7y1
............................................ucgcugcgaucuauugaa...................................................   152    0   7y1
............................................ucgcugcgaucuauugaaG..................................................   2      1   7y1
............................................ucgcugcgaucuaCugaaag.................................................   1      1   7y1
............................................ucgcugcgauGuauugaaag.................................................   1      1   7y1
............................................Ncgcugcgaucuauugaaag.................................................   2      1   7y1
............................................ucgcAgcgaucuauugaaag.................................................   3      1   7y1
............................................ucgcugcgNucuauugaaag.................................................   1      1   7y1
............................................ucgcugcgaucuUuugaaag.................................................   1      1   7y1
............................................ucgcugcgaucuauAgaaag.................................................   4      1   7y1
............................................Gcgcugcgaucuauugaaag.................................................   1      1   7y1
............................................ucgcugAgaucuauugaaag.................................................   17     1   7y1
............................................ucgcugcgaucuauugaaaC.................................................   3      1   7y1
............................................ucgcugcgaucuauugaaUg.................................................   1      1   7y1
............................................ucgcCgcgaucuauugaaag.................................................   2      1   7y1
............................................ucgcuCcgaucuauugaaag.................................................   1      1   7y1
............................................ucgcugcgaucuauuCaaag.................................................   2      1   7y1
............................................ucgcugcgaucAauugaaag.................................................   6      1   7y1
............................................ucgAugcgaucuauugaaag.................................................   4      1   7y1
............................................ucgcugcgCucuauugaaag.................................................   1      1   7y1
............................................ucgcugcgaucGauugaaag.................................................   1      1   7y1
............................................ucgcugcgaucuaAugaaag.................................................   3      1   7y1
............................................ucgcugcgaucuauugaaGg.................................................   3      1   7y1
............................................ucgcugcgaucuauugaaag.................................................   3906   0   7y1
............................................ucgcuUcgaucuauugaaag.................................................   5      1   7y1
............................................ucgcugcgaucuauugaaCg.................................................   1      1   7y1
............................................uAgcugcgaucuauugaaag.................................................   12     1   7y1
............................................ucgcugcCaucuauugaaag.................................................   1      1   7y1
............................................ucgcugcgaucuauuUaaag.................................................   2      1   7y1
............................................ucgGugcgaucuauugaaag.................................................   2      1   7y1
............................................ucgcugGgaucuauugaaag.................................................   2      1   7y1
............................................ucgcugcgauNuauugaaag.................................................   1      1   7y1
............................................ucUcugcgaucuauugaaag.................................................   1      1   7y1
............................................ucgcugcgauAuauugaaag.................................................   4      1   7y1
............................................uGgcugcgaucuauugaaag.................................................   2      1   7y1
............................................ucgcugcgaAcuauugaaag.................................................   11     1   7y1
............................................Acgcugcgaucuauugaaag.................................................   60     1   7y1
............................................ucgcuAcgaucuauugaaag.................................................   2      1   7y1
............................................ucgcGgcgaucuauugaaag.................................................   1      1   7y1
............................................ucgcugcUaucuauugaaag.................................................   2      1   7y1
............................................ucgcugcgaucuGuugaaag.................................................   1      1   7y1
............................................Acgcugcgaucuauugaaagu................................................   4      1   7y1
............................................ucgcugcCaucuauugaaagu................................................   1      1   7y1
............................................ucgcugcgaAcuauugaaagu................................................   1      1   7y1
............................................ucgcugcgaucuauugaaagu................................................   138    0   7y1
............................................ucgcugcgaucAauugaaagu................................................   1      1   7y1
............................................ucgcugcgaucuauugaaagucagcccucgaG.....................................   1      1   7y1
............................................ucgcugcgaucuauugaaagucagcUcucgac.....................................   1      1   7y1
............................................ucgcugcgaucuauugaaagucagccAucgac.....................................   1      1   7y1
............................................Ncgcugcgaucuauugaaagucagcccucgac.....................................   1      1   7y1
```

```
 Star                                                     Mature
gaccugcuucugggucgggguuucguacguagcagagcagcucccucgcugcgaucuauugaaagucagcccucgacacaaggguuuguccgcgcgcgcgcgcgcgcgcgcgugc
.............................................ucgcugcgaucuaGugaaagucagcccucgac......................................      1      1      7y1
.............................................ucgcugcgaucuaAugaaagucagcccucgac......................................      1      1      7y1
.............................................ucgcugcgaucuauugaaagucagcccucgac......................................      506    0      7y1
.............................................ucgcugcgaucuauuCaaagucagcccucgac......................................      1      1      7y1
.............................................ucgcugcgaucuauugaaagucagcccucgaA......................................      1      1      7y1
.............................................ucgcugcUaucuauugaaagucagcccucgac......................................      1      1      7y1
.............................................Acgcugcgaucuauugaaagucagcccucgac......................................      7      1      7y1
..............................................cgcugcgaucuaAugaaa...................................................      1      1      7y1
..............................................cUcugcgaucuauugaaa...................................................      1      1      7y1
..............................................cgcugAgaucuauugaaa...................................................      1      1      7y1
..............................................cCcugcgaucuauugaaa...................................................      2      1      7y1
..............................................cgcugcgaucuauugaaa...................................................      357    0      7y1
..............................................cgcuAcgaucuauugaaa...................................................      1      1      7y1
..............................................cgcugcgaAcuauugaaa...................................................      1      1      7y1
..............................................cgcugcUaucuauugaaa...................................................      1      1      7y1
..............................................cgcugcgaucuauugaaG...................................................      1      1      7y1
..............................................cgcugcgaGcuauugaaag..................................................      2      1      7y1
..............................................cgcugcgaucuUuugaaag..................................................      1      1      7y1
..............................................cgcugcgaucuauuCaaag..................................................      1      1      7y1
..............................................cgAugcgaucuauugaaag..................................................      20     1      7y1
..............................................cgcugcgaucuauugaUag..................................................      4      1      7y1
..............................................cgcugcgaucuauCgaaag..................................................      1      1      7y1
..............................................cgcugcgaucuauugaaaC..................................................      1      1      7y1
..............................................cgcGgcgaucuauugaaag..................................................      2      1      7y1
..............................................cgcugcgaucuauuUaaag..................................................      150    1      7y1
..............................................Agcugcgaucuauugaaag..................................................      16     1      7y1
..............................................cgGugcgaucuauugaaag..................................................      3      1      7y1
..............................................cgcugcgaucuauAgaaag..................................................      9      1      7y1
..............................................Ugcugcgaucuauugaaag..................................................      2      1      7y1
..............................................cgUugcgaucuauugaaag..................................................      1      1      7y1
..............................................cgcugAgaucuauugaaag..................................................      29     1      7y1
..............................................cgcugcgauGuauugaaag..................................................      2      1      7y1
..............................................cCcugcgaucuauugaaag..................................................      4      1      7y1
..............................................cgcugcgUucuauugaaag..................................................      2      1      7y1
..............................................cgcCgcgaucuauugaaag..................................................      1      1      7y1
..............................................cgcugcgaucuauugaGag..................................................      2      1      7y1
..............................................cgcugcgaucuauugaaag..................................................      6574   0      7y1
..............................................cgcugcgaucAauugaaag..................................................      4      1      7y1
..............................................Ngcugcgaucuauugaaag..................................................      1      1      7y1
..............................................cgcugGgaucuauugaaag..................................................      2      1      7y1
..............................................cUcugcgaucuauugaaag..................................................      8      1      7y1
..............................................cgcugcCaucuauugaaag..................................................      4      1      7y1
..............................................cgcuCcgaucuauugaaag..................................................      2      1      7y1
..............................................cgcugcgaucGauugaaag..................................................      1      1      7y1
..............................................cgcAgcgaucuauugaaag..................................................      11     1      7y1
..............................................cgcugcgauAuauugaaag..................................................      14     1      7y1
..............................................cgcugcgaucuauugaaaU..................................................      4      1      7y1
..............................................cgcugcgaucuauugUaag..................................................      2      1      7y1
..............................................cAcugcgaucuauugaaag..................................................      1      1      7y1
..............................................cgcuUcgaucuauugaaag..................................................      7      1      7y1
..............................................cgcugcgaAcuauugaaag..................................................      9      1      7y1
..............................................cgcugcgaucuauugaaUg..................................................      1      1      7y1
..............................................Ggcugcgaucuauugaaag..................................................      4      1      7y1
..............................................cgcugcgaucuaAugaaag..................................................      7      1      7y1
..............................................cgcuAcgaucuauugaaag..................................................      1      1      7y1
..............................................cgcugcUaucuauugaaag..................................................      10     1      7y1
..............................................cgcugcgaucuauugaaaA..................................................      1      1      7y1
..............................................cUcugcgaucuauugaaagu.................................................      3      1      7y1
..............................................cgcugcgaucuauugaaaCu.................................................      1      1      7y1
..............................................cgcugcgaucuauugaaagu.................................................      160    0      7y1
..............................................Ggcugcgaucuauugaaagu.................................................      1      1      7y1
..............................................cgcugcgaucuauAgaaagu.................................................      1      1      7y1
..............................................cgcugAgaucuauugaaagu.................................................      2      1      7y1
..............................................cgcugcgaucuaAugaaagu.................................................      1      1      7y1
..............................................cgcugcgaucuauugaaagAc................................................      1      1      7y1
..............................................cgcugcgaucuauAgaaaguc................................................      2      1      7y1
..............................................cgcugcgauAuauugaaaguc................................................      2      1      7y1
..............................................cUcugcgaucuauugaaaguc................................................      4      1      7y1
..............................................cgcugcgaAcuauugaaaguc................................................      1      1      7y1
..............................................Agcugcgaucuauugaaaguc................................................      2      1      7y1
```

```
     Star                                                                       Mature
gaccugcuucugggucgggguuucguacguagcagagcagcucccucgcugcgaucuauugaaagucagcccucgacacaaggguuuguccgcgcgcgcgcgcgcgcgcgcgugc
..............................................cgcugcgaucuauugGaaguc................................................    1     1     7y1
..............................................cgcugcgaucuauugaGaguc................................................    1     1     7y1
..............................................cgcugcgaucuUuugaaaguc................................................    1     1     7y1
..............................................cgcugAgaucuauugaaaguc................................................    2     1     7y1
..............................................cgcugcgauUuauugaaaguc................................................    1     1     7y1
..............................................cgcugcgaucuauugaaaguA................................................    1     1     7y1
..............................................cgcugcgaucuauugaaaguc................................................    553   0     7y1
..............................................cgcAgcgaucuauugaaaguc................................................    1     1     7y1
..............................................cgcugcgaucuauugaaGgucag..............................................    2     1     7y1
..............................................cgcugcgaucuauugaaagucag..............................................    184   0     7y1
..............................................cgcugcgaucuauugaaagucaU..............................................    1     1     7y1
..............................................cgcugcgaucuauAgaaagucag..............................................    1     1     7y1
..............................................cgcugcgaucuaAugaaagucag..............................................    1     1     7y1
..............................................cgcugAgaucuauugaaagucag..............................................    1     1     7y1
..............................................cUcugcgaucuauugaaagucag..............................................    1     1     7y1
..............................................Agcugcgaucuauugaaagucagcccucgac......................................    1     1     7y1
..............................................cgAugcgaucuauugaaagucagcccucgac......................................    1     1     7y1
..............................................cgcugAgaucuauugaaagucagcccucgac......................................    1     1     7y1
..............................................cgcugcgaucuauugaaagucagcccucgCc......................................    1     1     7y1
..............................................cgcugcgaucuauugaaagucagcccucgac......................................    162   0     7y1
...............................................gcuUcgaucuauugaaag..................................................    4     1     7y1
...............................................gcugcgaucAauugaaag..................................................    7     1     7y1
...............................................Ucugcgaucuauugaaag..................................................    12    1     7y1
...............................................gcGgcgaucuauugaaag..................................................    2     1     7y1
...............................................gcugcgaucuauuUaaag..................................................    1     1     7y1
...............................................gcugcUaucuauugaaag..................................................    5     1     7y1
...............................................gcugcgauAuauugaaag..................................................    3     1     7y1
...............................................gcugcgaucuauugaaaC..................................................    2     1     7y1
...............................................gcugcgaucuaAugaaag..................................................    4     1     7y1
...............................................gcugcCaucuauugaaag..................................................    2     1     7y1
...............................................gAugcgaucuauugaaag..................................................    6     1     7y1
...............................................gcugcgaucuauugaaag..................................................    2699  0     7y1
...............................................gcuCcgaucuauugaaag..................................................    1     1     7y1
...............................................gcugcgaucuGuugaaag..................................................    1     1     7y1
...............................................gcugcgaucuauuCaaag..................................................    1     1     7y1
...............................................gcugcgaucuauugGaag..................................................    1     1     7y1
...............................................gcugcgaAcuauugaaag..................................................    1     1     7y1
...............................................gcAgcgaucuauugaaag..................................................    4     1     7y1
...............................................gcugcgaucuauugaUag..................................................    2     1     7y1
...............................................gGugcgaucuauugaaag..................................................    2     1     7y1
...............................................Ccugcgaucuauugaaag..................................................    8     1     7y1
...............................................gcugcgCucuauugaaag..................................................    1     1     7y1
...............................................gcugGgaucuauugaaag..................................................    1     1     7y1
...............................................gcugcgaucuauAgaaag..................................................    8     1     7y1
...............................................gcugcgaucuUuugaaag..................................................    1     1     7y1
...............................................gcugcgaucuauugaGag..................................................    2     1     7y1
...............................................Acugcgaucuauugaaag..................................................    1     1     7y1
...............................................gcugcgaucuaCugaaag..................................................    1     1     7y1
...............................................Ncugcgaucuauugaaag..................................................    2     1     7y1
...............................................gcugAgaucuauugaaag..................................................    6     1     7y1
...............................................gcuAcgaucuauugaaag..................................................    1     1     7y1
...............................................gcugcgaucuauugaaaU..................................................    1     1     7y1
...............................................gcugcgaucuauugaaUg..................................................    1     1     7y1
...............................................gcAgcgaucuauugaaagu.................................................    2     1     7y1
...............................................gcugcgaucuauugaaagu.................................................    111   0     7y1
...............................................gcugcgaucuauugaaagA.................................................    1     1     7y1
...............................................gcugcgaAcuauugaaagu.................................................    1     1     7y1
...............................................gAugcgaucuauugaaagu.................................................    2     1     7y1
...............................................gcugcgaucuauAgaaagu.................................................    1     1     7y1
...............................................gcugcgaucuauugaaagAc................................................    2     1     7y1
...............................................gcugAgaucuauugaaaguc................................................    1     1     7y1
...............................................gcugcgaAcuauugaaaguc................................................    1     1     7y1
...............................................gcugcgaucuauugaaaguU................................................    1     1     7y1
...............................................gcugcgaucuauAgaaaguc................................................    1     1     7y1
...............................................gcuAcgaucuauugaaaguc................................................    1     1     7y1
...............................................gAugcgaucuauugaaaguc................................................    1     1     7y1
...............................................gcugcgaucuauugaaaUuc................................................    1     1     7y1
...............................................Ucugcgaucuauugaaaguc................................................    2     1     7y1
...............................................gcugcgaucuaAugaaaguc................................................    1     1     7y1
...............................................gcugcgaucGauugaaaguc................................................    1     1     7y1
```

Star Mature

```
gaccugcuucugggucgggguuucguacguagcagagcagcucccucgcugcgaucuauugaaagucagcccucgacacaaggguuuguccgcgcgcgcgcgcgcgcgcgugc
...............................................gcugcgaucuauuNaaaguc..............................................   1     1   7y1
...............................................gcugcgaucuaCugaaaguc..............................................   1     1   7y1
...............................................gcugcgaucAauugaaaguc..............................................   1     1   7y1
...............................................gcugcgaGcuauugaaaguc..............................................   1     1   7y1
...............................................gcugcgaucuauugaaaguA..............................................   1     1   7y1
...............................................gcuCcgaucuauugaaaguc..............................................   1     1   7y1
...............................................gcugcgaucuauugaaaguc..............................................   562   0   7y1
...............................................Acugcgaucuauugaaagucag............................................   1     1   7y1
...............................................gcugcgaucuauugaaagucag............................................   181   0   7y1
...............................................gAugcgaucuauugaaagucag............................................   2     1   7y1
................................................cugcgaAcuauugaaagu...............................................   2     1   7y1
................................................cugcgaucuauugaaagA...............................................   1     1   7y1
................................................Nugcgaucuauugaaagu...............................................   1     1   7y1
................................................cugcgaucuaAugaaagu...............................................   1     1   7y1
................................................cuAcgaucuauugaaagu...............................................   1     1   7y1
................................................cugcgaucuauugaaagu...............................................   301   0   7y1
................................................cugcgaucuauugaaaUu...............................................   1     1   7y1
................................................Gugcgaucuauugaaagu...............................................   1     1   7y1
................................................cAgcgaucuauugaaagu...............................................   1     1   7y1
................................................Augcgaucuauugaaagu...............................................   1     1   7y1
................................................cugAgaucuauugaaaguc..............................................   1     1   7y1
................................................cugcgaucuauugaaaguU..............................................   1     1   7y1
................................................cugcgaucuauAgaaaguc..............................................   1     1   7y1
................................................cuUcgaucuauugaaaguc..............................................   1     1   7y1
................................................cugcgaAcuauugaaaguc..............................................   1     1   7y1
................................................cAgcgaucuauugaaaguc..............................................   1     1   7y1
................................................cugcgauAuauugaaaguc..............................................   1     1   7y1
................................................cugcgaGcuauugaaaguc..............................................   1     1   7y1
................................................Augcgaucuauugaaaguc..............................................   3     1   7y1
................................................cugcgaucAauugaaaguc..............................................   1     1   7y1
................................................cugcgaucuauugaaagAc..............................................   1     1   7y1
................................................cugGgaucuauugaaaguc..............................................   1     1   7y1
................................................cugcUaucuauugaaaguc..............................................   1     1   7y1
................................................cugcgaucuauugaaaguc..............................................   436   0   7y1
................................................cugcgaucAauugaaagucagc...........................................   3     1   7y1
................................................cugcgaucCauugaaagucagc...........................................   337   1   7y1
................................................cugcgaucuauugaaagucagcccA........................................   1     1   7y1
................................................cuAcgaucuauugaaagucagcccu........................................   1     1   7y1
................................................Augcgaucuauugaaagucagcccu........................................   1     1   7y1
................................................cugcgaucuauugaaagucagcccu........................................   172   0   7y1
................................................cuCcgaucuauugaaagucagcccu........................................   1     1   7y1
................................................cugcgaucAauugaaagucagcccu........................................   1     1   7y1
................................................cuUcgaucuauugaaagucagcccu........................................   1     1   7y1
................................................cuUcgaucuauugaaagucagcccuc.......................................   169   1   7y1
................................................cugUgaucuauugaaagucagcccucgac....................................   1     1   7y1
................................................cugcgaucuauugaaagucagcccucgac....................................   218   0   7y1
................................................cugcgaucuauugaaaCucagcccucgac....................................   1     1   7y1
................................................cugcgaucuauugaaagAcagcccucgac....................................   1     1   7y1
................................................cugcgaucuauugaaagucagcccucgaA....................................   1     1   7y1
.................................................ugAgaucuauugaaagucagc...........................................   1     1   7y1
.................................................ugcgaucuauugaaagucagc...........................................   702   0   7y1
.................................................ugcgaucuauugaaaguAagc...........................................   1     1   7y1
.................................................uCcgaucuauugaaagucagc...........................................   2     1   7y1
.................................................ugcgaucuauugaCagucagc...........................................   1     1   7y1
.................................................ugcgauAuauugaaagucagc...........................................   2     1   7y1
.................................................ugcgaucuauugaaagucagG...........................................   1     1   7y1
.................................................Ggcgaucuauugaaagucagc...........................................   2     1   7y1
.................................................ugcgaucuauugaaagucagU...........................................   1     1   7y1
.................................................ugcgaucuauCgaaagucagc...........................................   1     1   7y1
.................................................ugcgaucuauugaaagAcagc...........................................   1     1   7y1
.................................................Agcgaucuauugaaagucagc...........................................   10    1   7y1
.................................................ugcgaucuauuAaaagucagc...........................................   1     1   7y1
.................................................ugcgaucuauuCaaagucagc...........................................   1     1   7y1
.................................................ugcgaucuauugaaagucagcAcucgacac..................................   1     1   7y1
.................................................ugcgaucuauugaaagucagcccucgacac..................................   154   0   7y1
.................................................ugcgaucuauugaaagucagcccuUgacac..................................   1     1   7y1
.................................................Agcgaucuauugaaagucagcccucgacac..................................   1     1   7y1
.................................................ugAgaucuauugaaagucagcccucgacac..................................   1     1   7y1
.................................................ugcgaucuauugaaagucagcccucgUcac..................................   1     1   7y1
.................................................ugcgaucuauugaaagucagcccucgacaA..................................   1     1   7y1
```

Star **Mature**

```
gaccugcuucugggucgggguuucguacguagcagagcagcucccucgcugcgaucuauugaaagucagcccucgacacaaggguuuguccgcgcgcgcgcgcgcgcgcgugc
..................................................gcgaucuauAgaaagucagc...........................................	1	1	7y1
..................................................gcgaucuauuCaaagucagc...........................................	1	1	7y1
..................................................gcgaucAauugaaagucagc...........................................	1	1	7y1
..................................................gcgaucuauugaaagCcagc...........................................	1	1	7y1
..................................................gcgaucCauugaaagucagc...........................................	157	1	7y1
..................................................gcgaucuauugaaagucagc...........................................	259	0	7y1
..................................................gcgaucuauugaGagucagc...........................................	390	1	7y1
..................................................gcgaucuauAgaaagucagcc..........................................	2	1	7y1
..................................................gcgaucuauuAaaagucagcc..........................................	1	1	7y1
..................................................gcgaucuauugaaaCucagcc..........................................	1	1	7y1
..................................................gcgaucuauugaaagucagcc..........................................	149	0	7y1
...................................................cgaucuauugaaagucagA...........................................	3	1	7y1
...................................................cgaucuauAgaaagucagc...........................................	4	1	7y1
...................................................cgaucuauugaaGgucagc...........................................	1	1	7y1
...................................................cgaucuauuUaaagucagc...........................................	1	1	7y1
...................................................cgauAuauugaaagucagc...........................................	1	1	7y1
...................................................Agaucuauugaaagucagc...........................................	1	1	7y1
...................................................cgaucuauugUaagucagc...........................................	1	1	7y1
...................................................cgaucuauugaaagucagc...........................................	565	0	7y1
...................................................cgaucAauugaaagucagc...........................................	2	1	7y1
...................................................cgaucuauGgaaagucagc...........................................	1	1	7y1
...................................................cgaucuauugaaaguGagc...........................................	1	1	7y1
...................................................cgaucuauugaaaguAagc...........................................	1	1	7y1
...................................................cUaucuauugaaagucagc...........................................	1	1	7y1
...................................................cgaucuauCgaaagucagc...........................................	1	1	7y1
...................................................cgaucuauugaaagucCgcccu........................................	1	1	7y1
...................................................cgaucuauugaaaUucagcccu........................................	1	1	7y1
...................................................cgaucuauugaaagucagcUcu........................................	1	1	7y1
...................................................cgaucAauugaaagucagcccu........................................	1	1	7y1
...................................................cgaucuauAgaaagucagcccu........................................	1	1	7y1
...................................................cgaucuauugaaagucagcccu........................................	243	0	7y1
...................................................cgaucuauugaaaguGagcccu........................................	1	1	7y1
...................................................cUaucuauugaaagucagcccu........................................	2	1	7y1
...................................................cgaucuaAugaaagucagcccu........................................	1	1	7y1
...................................................Ggaucuauugaaagucagcccucg......................................	1	1	7y1
...................................................cgauAuauugaaagucagcccucg......................................	1	1	7y1
...................................................Agaucuauugaaagucagcccucg......................................	2	1	7y1
...................................................cgaucAauugaaagucagcccucg......................................	2	1	7y1
...................................................cgaucuauugaaagAcagcccucg......................................	1	1	7y1
...................................................cgaucuauCgaaagucagcccucg......................................	1	1	7y1
...................................................cgaAcuauugaaagucagcccucg......................................	1	1	7y1
...................................................cgaucuauAgaaagucagcccucg......................................	1	1	7y1
...................................................cgaucuaGugaaagucagcccucg......................................	1	1	7y1
...................................................cgaucuauugaGagucagcccucg......................................	1	1	7y1
...................................................cCaucuauugaaagucagcccucg......................................	1	1	7y1
...................................................cgaucuauugaaagucagAccucg......................................	1	1	7y1
...................................................cgaucuauugaaagucagcccucg......................................	478	0	7y1
...................................................Ngaucuauugaaagucagcccucg......................................	1	1	7y1
...................................................cgUucuauugaaagucagcccucg......................................	1	1	7y1
...................................................Ngaucuauugaaagucagcccucgacacaa................................	1	1	7y1
...................................................Agaucuauugaaagucagcccucgacacaa................................	1	1	7y1
...................................................cgaucuauugaaagucagcccucgacacaG................................	1	1	7y1
...................................................cgaucAauugaaagucagcccucgacacaa................................	1	1	7y1
...................................................cgaucuauugaaagucagcccucgacacaa................................	237	0	7y1
...................................................cgaucuauugaaagucagcccucgCcacaa................................	1	1	7y1
...................................................cgauAuauugaaagucagcccucgacacaa................................	2	1	7y1
...................................................cgaucuauugaaagAcagcccucgacacaa................................	1	1	7y1
....................................................gaucuauuUaaagucagc...........................................	1	1	7y1
....................................................gaucCauugaaagucagc...........................................	142	1	7y1
....................................................gaucuauugaaagucagc...........................................	390	0	7y1
....................................................gaAcuauugaaagucagc...........................................	1	1	7y1
....................................................gaucAauugaaagucagc...........................................	2	1	7y1
....................................................gaucuauugaaagucagG...........................................	1	1	7y1
....................................................gauAuauugaaagucagc...........................................	1	1	7y1
....................................................gaucAauugaaagucagcc..........................................	2	1	7y1
....................................................gaucuauugaaaUucagcc..........................................	1	1	7y1
....................................................gaucuauuCaaagucagcc..........................................	2	1	7y1
....................................................gaucuaAugaaagucagcc..........................................	1	1	7y1
....................................................gaucuauugaaagucaCcc..........................................	1	1	7y1
....................................................gaucuauugaaagucGgcc..........................................	1	1	7y1
```

Star **Mature**

gaccugcuucugggucgggguuucguacguagcagagcagcucccucgcugcgaucuauugaaagucagcccucgacacaaggguuuguccgcgcgcgcgcgcgcgcgcgugc			
....gauGuauugaaagucagcc..	1	1	7y1
....gauAuauugaaagucagcc..	3	1	7y1
....gaucuauugaaagucagcA..	1	1	7y1
....gaucuauugaaagucagcG..	1	1	7y1
....gGucuauugaaagucagcc..	1	1	7y1
....gaucuauugaaaCucagcc..	2	1	7y1
....Caucuauugaaagucagcc..	1	1	7y1
....gaucuauugaaagucagcc..	683	0	7y1
....gaucuUuugaaagucagcc..	1	1	7y1
....gaucAauugaaagucagcccucg......................................	2	1	7y1
....gaucCauugaaagucagcccucg......................................	193	1	7y1
....aucuauugaaagucagcA..	13	1	7y1
....aucuauugaaagucagcc..	5120	0	7y1
....aucuauugaaagucaCcc..	5	1	7y1
....aucuauugaaaAucagcc..	1	1	7y1
....aucCauugaaagucagcc..	1243	1	7y1
....aucuauugaaagucaUcc..	2	1	7y1
....aucuUuugaaagucagcc..	1	1	7y1
....aucuauugaaagGcagcc..	1	1	7y1
....aucuauugaaaCucagcc..	4	1	7y1
....aucuauugaaagucagUc..	2	1	7y1
....aucuauugaaagAcagcc..	6	1	7y1
....aucuaAugaaagucagcc..	23	1	7y1
....auGuauugaaagucagcc..	1	1	7y1
....aAcuauugaaagucagcc..	19	1	7y1
....aucuauugaUagucagcc..	1	1	7y1
....auAuauugaaagucagcc..	27	1	7y1
....aucuauAgaaagucagcc..	21	1	7y1
....aucuaCugaaagucagcc..	1	1	7y1
....aucAauugaaagucagcc..	10	1	7y1
....aucuauugaaagucagcG..	2	1	7y1
....aucuauuCaaagucagcc..	4	1	7y1
....aucuauugaaGgucagcc..	1	1	7y1
....aucuauuUaaagucagcc..	3	1	7y1
....Uucuauugaaagucagcc..	3	1	7y1
....Nucuauugaaagucagcc..	5	1	7y1
....aucuauugaaaUucagcc..	2	1	7y1
....Gucuauugaaagucagcc..	4	1	7y1
....aucuauugaaUgucagcc..	1	1	7y1
....aucuauuAaaagucagcc..	2	1	7y1
....aucuauugaaaguAagcc..	12	1	7y1
....aucuauAgaaagucagccc...	8	1	7y1
....aucuUuugaaagucagccc...	1	1	7y1
....aucuauugaaagucaUccc...	1	1	7y1
....aucuauugaaagucagccU...	237	1	7y1
....aucuaCugaaagucagccc...	1	1	7y1
....aucuauugaaagucagccc...	1694	0	7y1
....aucuauugaaagAcagccc...	1	1	7y1
....aucuaAugaaagucagccc...	5	1	7y1
....auGuauugaaagucagccc...	2	1	7y1
....aucGauugaaagucagccc...	2	1	7y1
....aucuauugaaaUucagccc...	2	1	7y1
....aucuauuUaaagucagccc...	1	1	7y1
....aucuauugaaagucagccA...	6	1	7y1
....aucuauugaaagucagcAc...	4	1	7y1
....aucuauugaUagucagccc...	1	1	7y1
....aAcuauugaaagucagccc...	10	1	7y1
....aucuauugUaagucagccc...	1	1	7y1
....aucuauugaaaCucagccc...	1	1	7y1
....aucAauugaaagucagccc...	1	1	7y1
....aucCauugaaagucagccc...	209	1	7y1
....Nucuauugaaagucagccc...	1	1	7y1
....auAuauugaaagucagccc...	7	1	7y1
....aucuauugaaaguAagccc...	5	1	7y1
....Gucuauugaaagucagccc...	2	1	7y1
....aucuauugaaagucagccG...	3	1	7y1
....aucuauugaaagucagcGc...	2	1	7y1
....Nucuauugaaagucagcccu..	1	1	7y1
....aucuauugaaagAcagcccu..	1	1	7y1
....aucuauugaaUgucagcccu..	1	1	7y1

Star **Mature**

```
gaccugcuucugggucgggguuucguacguagcagagcagcucccucgcugcgaucuauugaaagucagcccucgacacaaggguuuguccgcgcgcgcgcgcgcgcgcgugc
.....................................................aucuauugaaagucagcAcu..........................................   2     1   7y1
.....................................................aucuauuCaaagucagcccu..........................................   1     1   7y1
.....................................................aucuauugaaagucaUcccu..........................................   1     1   7y1
.....................................................auAuauugaaagucagcccu..........................................   2     1   7y1
.....................................................aucuauugaaagucagcccG..........................................   2     1   7y1
.....................................................aucuaAugaaagucagcccu..........................................   4     1   7y1
.....................................................aucuauugaaagucagcccA..........................................   1     1   7y1
.....................................................aucuauugaaagucagcccu..........................................   755   0   7y1
.....................................................aucuauAgaaagucagcccu..........................................   3     1   7y1
.....................................................aAcuauugaaagucagcccu..........................................   2     1   7y1
.....................................................aucuauugaaagucagccAu..........................................   1     1   7y1
.....................................................aucCauugaaagucagcccu..........................................   223   1   7y1
.....................................................aucuauugaaaguAagcccu..........................................   1     1   7y1
.....................................................aucCauugaaagucagcccuc.........................................   1285  1   7y1
.....................................................aucAauugaaagucagcccuc.........................................   2     1   7y1
.....................................................aucuauAgaaagucagcccucg........................................   4     1   7y1
.....................................................aucuauugaaagucagcccucg........................................   1785  0   7y1
.....................................................aucuauugaaagucagcccucU........................................   1     1   7y1
.....................................................aucuauugaaagucagcAcucg........................................   2     1   7y1
.....................................................aucuauugaaagucagAccucg........................................   6     1   7y1
.....................................................aCcuauugaaagucagcccucg........................................   1     1   7y1
.....................................................aucuauuUaaagucagcccucg........................................   1     1   7y1
.....................................................aucuauugaaaguAagcccucg........................................   5     1   7y1
.....................................................aucuauugNaagucagcccucg........................................   1     1   7y1
.....................................................aucuauugaaagAcagcccucg........................................   2     1   7y1
.....................................................aucuaAugaaagucagcccucg........................................   6     1   7y1
.....................................................aAcuauugaaagucagcccucg........................................   5     1   7y1
.....................................................auAuauugaaagucagcccucg........................................   8     1   7y1
.....................................................aucuauugaaagucagcccAcg........................................   1     1   7y1
.....................................................aucuauugaaagucagUccucg........................................   2     1   7y1
.....................................................aucuauugaaagucagGccucg........................................   1     1   7y1
.....................................................aucuauugaaaUucagcccucg........................................   1     1   7y1
.....................................................Gucuauugaaagucagcccucg........................................   1     1   7y1
.....................................................aucuauugaUagucagcccucg........................................   1     1   7y1
.....................................................aucuauugaaagucGgcccucg........................................   2     1   7y1
.....................................................aucCauugaaagucagcccucg........................................   914   1   7y1
.....................................................aucuauuCaaagucagcccucg........................................   2     1   7y1
.....................................................aucuauugaaagucagccAucg........................................   2     1   7y1
.....................................................aucuauugaaagucaCcccucg........................................   1     1   7y1
.....................................................auUuauugaaagucagcccucg........................................   249   1   7y1
.....................................................aucuauugGaagucagcccucg........................................   1     1   7y1
.....................................................aucuauugaaagucagcccucC........................................   2     1   7y1
.....................................................Nucuauugaaagucagcccucg........................................   1     1   7y1
.....................................................aucuauugaaagucagcccuAg........................................   4     1   7y1
.....................................................aucuauugaaaguGagcccucg........................................   1     1   7y1
.....................................................aucuauugaGagucagcccucg........................................   1     1   7y1
.....................................................aucAauugaaagucagcccucg........................................   1     1   7y1
.....................................................aucuauugaaagucaUcccucg........................................   1     1   7y1
.....................................................aucuauugaaagucagcAcucga.......................................   2     1   7y1
.....................................................aucuauugaaaguAagcccucga.......................................   1     1   7y1
.....................................................aucuauugaaagucagccAucga.......................................   2     1   7y1
.....................................................aucuauugaaagucagcUcucga.......................................   1     1   7y1
.....................................................aucuauugaaagucagcccucga.......................................   460   0   7y1
.....................................................auAuauugaaagucagcccucga.......................................   2     1   7y1
.....................................................aucuauugaaagucagcccuAga.......................................   1     1   7y1
.....................................................aucuauugaaagucagccGucga.......................................   1     1   7y1
.....................................................aucuauAgaaagucagcccucga.......................................   6     1   7y1
.....................................................aucuaAugaaagucagcccucga.......................................   2     1   7y1
.....................................................aAcuauugaaagucagcccucga.......................................   3     1   7y1
.....................................................aucuauCgaaagucagcccucga.......................................   1     1   7y1
.....................................................aucuauugUaagucagcccucga.......................................   1     1   7y1
.....................................................aucuauugaaagucagcccucgac......................................   272   0   7y1
.....................................................Uucuauugaaagucagcccucgac......................................   2     1   7y1
.....................................................aucuauAgaaagucagcccucgac......................................   2     1   7y1
.....................................................aucuaAugaaagucagcccucgac......................................   2     1   7y1
.....................................................aucuauugaaagucagcccucgaA......................................   1     1   7y1
.....................................................aAcuauugaaagucagcccucgac......................................   1     1   7y1
.....................................................aucuauugaaagucaAcccucgacacaag.................................   1     1   7y1
.....................................................aucuauugaaagucagcccucUacacaag.................................   1     1   7y1
.....................................................aucuauugaaagucagccAucgacacaag.................................   1     1   7y1
```

Star **Mature**

gaccugcuucugggucgggguuucguacguagcagagcagcucccucgcugcgaucuauugaaagucagcccucgacacaaggguuuguccgcgcgcgcgcgcgcgcgcgcgugc			
....Nucuauugaaagucagcccucgacacaag.................................	1	1	7y1
....aAcuauugaaagucagcccucgacacaag.................................	1	1	7y1
....aucuauugaaagucagcccAcgacacaag.................................	1	1	7y1
....aucuauugaaagucagcccucgCcacaag.................................	1	1	7y1
....aucuaAugaaagucagcccucgacacaag.................................	1	1	7y1
....aucuauugaaagucCgcccucgacacaag.................................	1	1	7y1
....Uucuauugaaagucagcccucgacacaag.................................	1	1	7y1
....aucuauugaaagucagcccucgacUcaag.................................	1	1	7y1
....auAuauugaaagucagcccucgacacaag.................................	4	1	7y1
....aucuauugaaagucagcccucgacacaag.................................	494	0	7y1
....aucAauugaaagucagcccucgacacaag.................................	1	1	7y1
....aucuauugaaagucagcGcucgacacaag.................................	1	1	7y1
....aucuauugaaagucagcccucgacaGaag.................................	1	1	7y1
....aucuauuUaaagucagcccucgacacaag.................................	1	1	7y1
....ucuauugaaagucagGcc...	1	1	7y1
....ucuauugaaagucagccA...	6	1	7y1
....ucuauugaaagucaUccc...	2	1	7y1
....ucuauugaaagucagccc...	1308	0	7y1
....ucuauugaaaCucagccc...	1	1	7y1
....ucuauugaaagucaAccc...	1	1	7y1
....ucuauugaaagucCgccc...	1	1	7y1
....ucuauuUaaagucagccc...	3	1	7y1
....ucuauugaaaguAagccc...	3	1	7y1
....ucuauuAaaagucagccc...	2	1	7y1
....ucuaAugaaagucagccc...	3	1	7y1
....Gcuauugaaagucagccc...	2	1	7y1
....ucuauugaaagucagcAc...	1	1	7y1
....ucuGuugaaagucagccc...	1	1	7y1
....ucuauugaaagucagUcc...	4	1	7y1
....ucuauAgaaagucagccc...	3	1	7y1
....ucuauugaGagucagccc...	227	1	7y1
....uAuauugaaagucagccc...	5	1	7y1
....ucuauugaaagucagccG...	1	1	7y1
....ucAauugaaagucagccc...	3	1	7y1
....Acuauugaaagucagccc...	16	1	7y1
....ucuauugaaagucagccAu..	5	1	7y1
....ucuauugaaagGcagcccu..	1	1	7y1
....ucuaAugaaagucagcccu..	1	1	7y1
....ucuauugaaagucagcAcu..	3	1	7y1
....Ncuauugaaagucagcccu..	1	1	7y1
....ucuauugaaagucCgcccu..	1	1	7y1
....ucGauugaaagucagcccu..	1	1	7y1
....Acuauugaaagucagcccu..	6	1	7y1
....ucuauAgaaagucagcccu..	3	1	7y1
....ucuauugaaagucagcccG..	1	1	7y1
....ucAauugaaagucagcccu..	1	1	7y1
....ucuauugaaagucagUccu..	1	1	7y1
....ucuauugaaagucagcccA..	1	1	7y1
....ucuauuAaaagucagcccu..	1	1	7y1
....ucuauugaaagucagcccu..	861	0	7y1
....ucuauugaaaCucagcccu..	1	1	7y1
....ucAauugaaagucagcccuc...	1	1	7y1
....ucCauugaaagucagcccuc...	278	1	7y1
....ucuauugaaagucCgcccucg..	1	1	7y1
....ucAauugaaagucagcccucg..	1	1	7y1
....ucuauugaaagucagccGucg..	1	1	7y1
....ucuauAgaaagucagcccucg..	3	1	7y1
....ucuauCgaaagucagcccucg..	1	1	7y1
....ucuauugaaaUucagcccucg..	1	1	7y1
....ucuauugaaagucagUccucg..	1	1	7y1
....Gcuauugaaagucagcccucg..	1	1	7y1
....ucCauugaaagucagcccucg..	282	1	7y1
....ucuaAugaaagucagcccucg..	2	1	7y1
....uAuauugaaagucagcccucg..	1	1	7y1
....Ccuauugaaagucagcccucg..	1	1	7y1
....ucuauugaaagucagcccucU..	1	1	7y1
....ucuauugaaaguAagcccucg..	2	1	7y1
....ucuauugaaagucagcAcucg..	3	1	7y1
....ucuauugaaagucaCcccucg..	1	1	7y1
....ucuauugaaagucagcUcucg..	2	1	7y1

```
  Star                                                                   Mature
gaccugcuucugggucgggguuucguacguagcagagcagcucccucgcugcgaucuauugaaagucagcccucgacacaaggguuuguccgcgcgcgcgcgcgcgcgcgugc
......................................................Acuauugaaagucagcccucg........................................   9     1   7y1
......................................................ucuauugaaagucagccAucg........................................   2     1   7y1
......................................................ucuauugaaagucagcccucA........................................   1     1   7y1
......................................................ucuauugaaagucagcccucg........................................   1220  0   7y1
......................................................ucuauugaaagucagcUcucga.......................................   1     1   7y1
......................................................Gcuauugaaagucagcccucga.......................................   2     1   7y1
......................................................ucuauugaaGgucagcccucga.......................................   1     1   7y1
......................................................ucAauugaaagucagcccucga.......................................   1     1   7y1
......................................................uAuauugaaagucagcccucga.......................................   3     1   7y1
......................................................Acuauugaaagucagcccucga.......................................   3     1   7y1
......................................................ucuaCugaaagucagcccucga.......................................   1     1   7y1
......................................................ucuaAugaaagucagcccucga.......................................   1     1   7y1
......................................................ucuauuUaaagucagcccucga.......................................   1     1   7y1
......................................................ucuauugaaagucagcccucga.......................................   428   0   7y1
......................................................ucuauugaaagucagcccucgG.......................................   2     1   7y1
......................................................ucuauugaaaguAagcccucga.......................................   1     1   7y1
......................................................ucuauAgaaagucagcccucga.......................................   2     1   7y1
......................................................Gcuauugaaagucagcccucgac......................................   1     1   7y1
......................................................ucuauugaUagucagcccucgac......................................   1     1   7y1
......................................................uAuauugaaagucagcccucgac......................................   1     1   7y1
......................................................ucuauugaaagucagcccucgac......................................   299   0   7y1
......................................................ucuauugaaagucagccAucgac......................................   1     1   7y1
......................................................ucAauugaaagucagcccucgac......................................   1     1   7y1
......................................................ucuauugaaagucagcccAcgac......................................   1     1   7y1
......................................................Acuauugaaagucagcccucgac......................................   2     1   7y1
.......................................................cuauCgaaagucagcccu..........................................   1     1   7y1
.......................................................cuauugaaagucagcccG..........................................   1     1   7y1
.......................................................cuauugaaagucagcccu..........................................   416   0   7y1
.......................................................cuaAugaaagucagcccu..........................................   1     1   7y1
.......................................................cAauugaaagucagcccu..........................................   2     1   7y1
.......................................................cuauugaaaAucagcccu..........................................   1     1   7y1
.......................................................Auauugaaagucagcccu..........................................   1     1   7y1
.......................................................cuauugaaaguAagcccu..........................................   1     1   7y1
.......................................................cuauugaaaCucagcccu..........................................   1     1   7y1
.......................................................cuauugaaagAcagcccuc.........................................   2     1   7y1
.......................................................cuauugaaagucagcccuc.........................................   186   0   7y1
.......................................................cCauugaaagucagcccuc.........................................   488   1   7y1
.......................................................cuauugaUagucagcccuc.........................................   1     1   7y1
.......................................................cAauugaaagucagcccuc.........................................   5     1   7y1
.......................................................Guauugaaagucagcccuc.........................................   1     1   7y1
.......................................................cCauugaaagucagcccucg........................................   701   1   7y1
.......................................................cAauugaaagucagcccucg........................................   4     1   7y1
.......................................................cAauugaaagucagcccucgac......................................   1     1   7y1
.......................................................cuauugaaagucagcccucgac......................................   273   0   7y1
.......................................................cuauugaaagucagcAcucgac......................................   1     1   7y1
.......................................................cuauugaaagucGgcccucgac......................................   1     1   7y1
.......................................................cuauugaaagucagccAucgac......................................   3     1   7y1
.......................................................Auauugaaagucagcccucgac......................................   1     1   7y1
.......................................................cuauugaaagAcagcccucgac......................................   1     1   7y1
.......................................................cuauugaaagucagcccucgacacaaggguuu............................   1     0   7y1
........................................................uauugaaaUucagcccucg........................................   1     1   7y1
........................................................Aauugaaagucagcccucg........................................   6     1   7y1
........................................................uauugaaagucagcccucC........................................   1     1   7y1
........................................................uauugaaagucagcGcucg........................................   1     1   7y1
........................................................uauugaaagucagcccAcg........................................   2     1   7y1
........................................................uauugaaagucagcccucg........................................   490   0   7y1
........................................................uauugaaGgucagcccucg........................................   1     1   7y1
........................................................uauugaaagucagcccuAg........................................   1     1   7y1
........................................................uauugaaagAcagcccucg........................................   1     1   7y1
........................................................uauugCaagucagcccucga.......................................   1     1   7y1
........................................................uauugaaagucagcccucgG.......................................   2     1   7y1
........................................................uauugaaagucaCcccucga.......................................   1     1   7y1
........................................................uauugaaUgucagcccucga.......................................   1     1   7y1
........................................................Aauugaaagucagcccucga.......................................   2     1   7y1
........................................................Gauugaaagucagcccucga.......................................   1     1   7y1
........................................................uauugaaagucagcccucga.......................................   197   0   7y1
........................................................uauugaaagucagcccucgac......................................   549   0   7y1
........................................................uaAugaaagucagcccucgac......................................   4     1   7y1
........................................................uauugaaagAcagcccucgac......................................   1     1   7y1
........................................................uauugaaaUucagcccucgac......................................   1     1   7y1
```

```
 Star                                                                      Mature
gaccugcuucugggucgggguuucguacguagcagagcagcucccucgcugcgaucuauugaaagucagcccucgacacaaggguuuguccgcgcgcgcgcgcgcgcgcgcgugc
........................................................uauugaaagucagcAcucgac......................................   1     1     7y1
........................................................uauAgaaagucagcccucgac......................................   2     1     7y1
........................................................Aauugaaagucagcccucgac......................................   11    1     7y1
........................................................uauugaaagucagcccucUac......................................   1     1     7y1
........................................................uauugaaagucagcccCcgac......................................   1     1     7y1
........................................................uauugaaagucaCcccucgac......................................   1     1     7y1
........................................................uauugaaagucaUcccucgac......................................   1     1     7y1
........................................................uauugaaagucagAccucgac......................................   1     1     7y1
........................................................uauugaaagucagcccucgaGacaaggguu.............................   1     1     7y1
........................................................uauuCaaagucagcccucgacacaaggguu.............................   1     1     7y1
........................................................uauugaaaguAagcccucgacacaaggguu.............................   1     1     7y1
........................................................uauugaaagucagcAcucgacacaaggguu.............................   2     1     7y1
........................................................uauugaaagucagcccucgacacaaggAuu.............................   1     1     7y1
........................................................Aauugaaagucagcccucgacacaaggguu.............................   1     1     7y1
........................................................uauugaaagucagcccucgacacaaggguu.............................   155   0     7y1
........................................................uauugaaagucagcccAcgacacaaggguu.............................   1     1     7y1
........................................................uauugaaaUucagcccucgacacaaggguu.............................   1     1     7y1
........................................................uauugaaagucagcccucgacacaaggCuu.............................   1     1     7y1
........................................................uauugaaagucagcccucgacacaaggguG.............................   1     1     7y1
........................................................uauugaaagucagcccucgacaUaaggguuu............................   1     1     7y1
........................................................uauugaaagucagcccucgacacaagggAuu............................   1     1     7y1
........................................................uauugaaagucagAccucgacacaaggguuu............................   1     1     7y1
........................................................uauugaaagucagcccucgacacaaggguuu............................   220   0     7y1
........................................................uauugaaagucagcccAcgacacaaggguuu............................   1     1     7y1
........................................................Aauugaaagucagcccucgacacaaggguuu............................   7     1     7y1
........................................................uauugaaagucagccAucgacacaaggguuug...........................   1     1     7y1
........................................................uauugaaagucagcccuAgacacaaggguuug...........................   3     1     7y1
........................................................uauugaaagucagcccucgacacaaCgguuug...........................   1     1     7y1
........................................................uauugaaagucagcccucgacacaagggAuug...........................   2     1     7y1
........................................................uauugaaagucagcccucgacaAaaggguuug...........................   3     1     7y1
........................................................Nauugaaagucagcccucgacacaaggguuug...........................   1     1     7y1
........................................................uauugaaagucagcccucgacacaagggGuug...........................   1     1     7y1
........................................................Gauugaaagucagcccucgacacaaggguuug...........................   2     1     7y1
........................................................uauugaaaguAagcccucgacacaaggguuug...........................   2     1     7y1
........................................................uauugaaagucagcccucgacacaaggguuAg...........................   1     1     7y1
........................................................uauugaaagucagcccucgacacaagCguuug...........................   1     1     7y1
........................................................uauugaaagucagGccucgacacaaggguuug...........................   1     1     7y1
........................................................uauugaaagucGgcccucgacacaaggguuug...........................   1     1     7y1
........................................................uauuCaaagucagcccucgacacaaggguuug...........................   1     1     7y1
........................................................uauugaaagucagcccCcgacacaaggguuug...........................   1     1     7y1
........................................................uauugaaagucagcccAcgacacaaggguuug...........................   2     1     7y1
........................................................uauugaaagucagcGcucgacacaaggguuug...........................   1     1     7y1
........................................................uauugaaagucaCcccucgacacaaggguuug...........................   2     1     7y1
........................................................uauugaaagucagcccucgacacaagUguuug...........................   1     1     7y1
........................................................uauugaaagucagcAcucgacacaaggguuug...........................   6     1     7y1
........................................................uauugaaagucagAccucgacacaaggguuug...........................   1     1     7y1
........................................................uauugaaaUucagcccucgacacaaggguuug...........................   1     1     7y1
........................................................uauugaaagucagcccucgacacaaggguuug...........................   684   0     7y1
........................................................uauuAaaagucagcccucgacacaaggguuug...........................   1     1     7y1
........................................................Aauugaaagucagcccucgacacaaggguuug...........................   13    1     7y1
.........................................................auugaaagucagccAucg........................................   1     1     7y1
.........................................................auuUaaagucagcccucg........................................   1     1     7y1
.........................................................auAgaaagucagcccucg........................................   1     1     7y1
.........................................................auuCaaagucagcccucg........................................   1     1     7y1
.........................................................auugaaagucagcccucg........................................   487   0     7y1
.........................................................aAugaaagucagcccucg........................................   2     1     7y1
.........................................................auugaGagucagcccucg........................................   1     1     7y1
.........................................................auugaaaguAagcccucg........................................   2     1     7y1
.........................................................auugaaagucagUccucg........................................   1     1     7y1
.........................................................Uuugaaagucagcccucg........................................   1     1     7y1
.........................................................auugaaagucagcAcucg........................................   2     1     7y1
.........................................................auugaaagucagAccucg........................................   3     1     7y1
.........................................................auugaaagucagcccucC........................................   1     1     7y1
.........................................................auugaaagucagcccuAga.......................................   2     1     7y1
.........................................................auugaaagucaUcccucga.......................................   1     1     7y1
.........................................................auugaaagucagcccucgC.......................................   1     1     7y1
.........................................................auugaaagucagAccucga.......................................   1     1     7y1
.........................................................auugaaagucagcccAcga.......................................   1     1     7y1
.........................................................auugaaagucagccAucga.......................................   1     1     7y1
.........................................................auugaaagAcagcccucga.......................................   1     1     7y1
```

Star **Mature**

```
gaccugcuucugggucgggguuucguacguagcagagcagcucccucgcugcgaucuauugaaagucagcccucgacacaaggguuuguccgcgcgcgcgcgcgcgcgcgugc
.........................................................auugaaaguGagcccucga.....................................  1    1  7y1
.........................................................auugaaagucagcccucga.....................................  452  0  7y1
.........................................................aAugaaagucagcccucga.....................................  1    1  7y1
.........................................................auugaaagucagAccucgac....................................  1    1  7y1
.........................................................auugaaagucagccAucgac....................................  3    1  7y1
.........................................................aAugaaagucagcccucgac....................................  1    1  7y1
.........................................................auugaaagucagcccucgac....................................  419  0  7y1
.........................................................auugaaagucagUccucgac....................................  2    1  7y1
.........................................................auugaaagucagcccucgCc....................................  2    1  7y1
.........................................................auugaaagAcagcccucgac....................................  2    1  7y1
.........................................................auugaaaguAagcccucgac....................................  2    1  7y1
.........................................................auugaaagucagcccucgaca...................................  189  0  7y1
.........................................................aAugaaagucagcccucgaca...................................  2    1  7y1
.........................................................auugaaagucagcccucgaGa...................................  1    1  7y1
.........................................................auugaaaguAagcccucgaca...................................  2    1  7y1
.........................................................auugaaagucagcccucgacG...................................  1    1  7y1
.........................................................auugaaagucagcAcucgacaca.................................  1    1  7y1
.........................................................auugaaaguAagcccucgacaca.................................  1    1  7y1
.........................................................auugaaagucagcccucgacaca.................................  165  0  7y1
.........................................................auugaaagucagcccucgCcaca.................................  4    1  7y1
.........................................................aAugaaagucagcccucgacaca.................................  1    1  7y1
.........................................................auugaaagucagcccucgUcaca.................................  1    1  7y1
.........................................................Uuugaaagucagcccucgacaca.................................  1    1  7y1
.........................................................auugaaagucagccAucgacaca.................................  2    1  7y1
.........................................................auugaaagucagcccAcgacacaag...............................  1    1  7y1
.........................................................auuCaaagucagcccucgacacaag...............................  1    1  7y1
.........................................................auugaaagucagccAucgacacaag...............................  2    1  7y1
.........................................................Uuugaaagucagcccucgacacaag...............................  1    1  7y1
.........................................................auugaaagucagcccucgCcacaag...............................  1    1  7y1
.........................................................auugaaaguAagcccucgacacaag...............................  1    1  7y1
.........................................................auugaaagucUgcccucgacacaag...............................  1    1  7y1
.........................................................auugaaaCucagcccucgacacaag...............................  1    1  7y1
.........................................................auugaaagucagcccucgacacaag...............................  209  0  7y1
.........................................................auugaaagucagcccucgacacaaC...............................  1    1  7y1
.........................................................Guugaaagucagcccucgacacaag...............................  2    1  7y1
.........................................................auugaaagucagcccucCacacaag...............................  1    1  7y1
.........................................................auugaaagucagcccucgacacaaggguu...........................  1    0  7y1
.........................................................auugaaaUucagcccucgacacaaggguuug.........................  1    1  7y1
.........................................................auugaaagucagccAucgacacaaggguuug.........................  1    1  7y1
.........................................................auugaaagucagcccucgacacaaggguAug.........................  1    1  7y1
.........................................................auugaaagucagcccuAgacacaaggguuug.........................  1    1  7y1
.........................................................auugaaagucagcccucgacacaaggguuAg.........................  2    1  7y1
.........................................................auugaaagucagcccAcgacacaaggguuug.........................  1    1  7y1
.........................................................auugaaagucagcccucgacacaaggguuug.........................  187  0  7y1
.........................................................auugaaagucagcccucgacacaagggGuug.........................  2    1  7y1
.........................................................auugaaagucagAccucgacacaaggguuug.........................  2    1  7y1
.........................................................auugaaagucagcccucgacGcaaggguuugu........................  3    1  7y1
.........................................................auugaaagucagcccucgacaGaaggguuugu........................  1    1  7y1
.........................................................auugaaagucagcccucgacacaaCgguuugu........................  2    1  7y1
.........................................................auugaaagucagcccucUacacaaggguuugu........................  1    1  7y1
.........................................................auugaaagucagccGucgacacaaggguuugu........................  1    1  7y1
.........................................................auugaaagucaUcccucgacacaaggguuugu........................  4    1  7y1
.........................................................auugaGagucagcccucgacacaaggguuugu........................  2    1  7y1
.........................................................auugaaagucagcccucgacacaagCguuugu........................  11   1  7y1
.........................................................auugaaagucagcUcucgacacaaggguuugu........................  3    1  7y1
.........................................................Nuugaaagucagcccucgacacaaggguuugu........................  4    1  7y1
.........................................................auugaaagAcagcccucgacacaaggguuugu........................  6    1  7y1
.........................................................auugaaagucagcccucgacacaaggguuugG........................  12   1  7y1
.........................................................auugaaaguUagcccucgacacaaggguuugu........................  2    1  7y1
.........................................................auugaaagucagcccucgacacaGggguuugu........................  2    1  7y1
.........................................................auugaaagucagcccucgacacaaggguGugu........................  5    1  7y1
.........................................................auugaaagucCgcccucgacacaaggguuugu........................  2    1  7y1
.........................................................aAugaaagucagcccucgacacaaggguuugu........................  23   1  7y1
.........................................................auugaaagucagcccucgaAacaaggguuugu........................  2    1  7y1
.........................................................auugaaagucagcccucgaUacaaggguuugu........................  211  1  7y1
.........................................................auuAaaagucagcccucgacacaaggguuugu........................  1    1  7y1
.........................................................auugaaagucagcccuAgacacaaggguuugu........................  9    1  7y1
.........................................................auugaaagucagcccucgacaAaggguuugu.........................  2    1  7y1
.........................................................Guugaaagucagcccucgacacaaggguuugu........................  6    1  7y1
.........................................................auugaaagucagcccucgacacaaggguuugC........................  1    1  7y1
```

Star **Mature**

gaccugcuucugggucgggguuucguacguagcagagcagcucccucgcugcgaucuauugaaagucagcccucgacacaaggguuuguccgcgcgcgcgcgcgcgcgugc			
....auugGaagucagcccucgacacaaggguuugu......................	1	1	7y1
....auugaaagucagcccucgacacUaggguuugu......................	1	1	7y1
....auugaaagucagcccucgaGacaaggguuugu......................	2	1	7y1
....auuUaaagucagcccucgacacaaggguuugu......................	1	1	7y1
....auugaaagucagcccucgCcacaaggguuugu......................	19	1	7y1
....Uuugaaagucagcccucgacacaaggguuugu......................	8	1	7y1
....auAgaaagucagcccucgacacaaggguuugu......................	4	1	7y1
....auugaaagucagcccucgacacaaggUuuugu......................	3	1	7y1
....auugaaagucagcccucgacacaaggguuAgu......................	10	1	7y1
....auugaaagucagcccucgacacaaggguuugA......................	19	1	7y1
....auugaaagucagcccucgacacaagggAuugu......................	28	1	7y1
....auugaaagucagcccucgacacCaggguuugu......................	1	1	7y1
....auugaaagucagcccucgacacaaggguuuCu......................	11	1	7y1
....auugaaagucagcccucgacacaaUgguuugu......................	5	1	7y1
....auugaaagucagcccucgacCcaaggguuugu......................	1	1	7y1
....auugaaagucagccUucgacacaaggguuugu......................	1	1	7y1
....auugaaagCcagcccucgacacaaggguuugu......................	1	1	7y1
....auugaaagucagcAcucgacacaaggguuugu......................	22	1	7y1
....auugaaagucagAccucgacacaaggguuugu......................	16	1	7y1
....auugaaagucagcccAcgacacaaggguuugu......................	13	1	7y1
....auugaaagucagcccucgacacaaggguAugu......................	18	1	7y1
....auugaaagucagcccucgacacaagggCuugu......................	1	1	7y1
....auugaaagucagcccucgacacaaggAuuugu......................	1	1	7y1
....auugaaagucagcccucgacacaaggguuugu......................	7347	0	7y1
....auugaaaCucagcccucgacacaaggguuugu......................	4	1	7y1
....auGgaaagucagcccucgacacaaggguuugu......................	1	1	7y1
....auugCaagucagcccucgacacaaggguuugu......................	1	1	7y1
....auugaaagucagcccucgacacaaggCuuugu......................	13	1	7y1
....auugaaagucagcccucgacacaaggguuuUu......................	7	1	7y1
....auugaaagucagcGcucgacacaaggguuugu......................	1	1	7y1
....auuCaaagucagcccucgacacaaggguuugu......................	1	1	7y1
....auugaaaguAagcccucgacacaaggguuugu......................	13	1	7y1
....auugaaagucagcccucgUcacaaggguuugu......................	1	1	7y1
....auugaaagucagcccucgacacaagUguuugu......................	3	1	7y1
....auugaaagucGgcccucgacacaaggguuugu......................	1	1	7y1
....auugaaagucaAcccucgacacaaggguuugu......................	1	1	7y1
....auugaaagucagcccucgacacaagggGuugu......................	12	1	7y1
....auugaaagucagcccucCacacaaggguuugu......................	2	1	7y1
....auugaaagucUgcccucgacacaaggguuugu......................	1	1	7y1
....auCgaaagucagcccucgacacaaggguuugu......................	1	1	7y1
....auugaaagucagccAucgacacaaggguuugu......................	15	1	7y1
....auugaaaguGagcccucgacacaaggguuugu......................	4	1	7y1
....auugaaagucagcccucgacacGaggguuugu......................	2	1	7y1
....aCugaaagucagcccucgacacaaggguuugu......................	1	1	7y1
....uugaaagucagcccucga...................................	208	0	7y1
....Augaaagucagcccucga...................................	4	1	7y1
....uugaaagucagccAucga...................................	3	1	7y1
....uAgaaagucagcccucga...................................	1	1	7y1
....uugaaaguAagcccucgac..................................	1	1	7y1
....Augaaagucagcccucgac..................................	4	1	7y1
....uugaaagucagcAcucgac..................................	1	1	7y1
....uuUaaagucagcccucgac..................................	1	1	7y1
....uugaaagucagcccucgac..................................	211	0	7y1
....uugaaagucagcAcucgacacaaggguuug.......................	1	1	7y1
....uugaaagucagcccucgacacaagUguuug.......................	1	1	7y1
....uugaaagucagcccuAgacacaaggguuug.......................	1	1	7y1
....uugaaagucagcccucgacacaaggguuug.......................	182	0	7y1
....uugaaagucagGccucgacacaaggguuug.......................	1	1	7y1
....uugaaagucagccAucgacacaaggguuug.......................	1	1	7y1
....uugaaaguAagcccucgacacaaggguuug.......................	1	1	7y1
....uAgaaagucagcccucgacacaaggguuug.......................	1	1	7y1
....uugaaagucagcccucgacacaaCgguuug.......................	1	1	7y1
....uugaaagucagcccucgacUcaaggguuugu......................	1	1	7y1
....uugaaagucagcccucgacacaagggAuugu......................	6	1	7y1
....uugaaagucagAccucgacacaaggguuugu......................	2	1	7y1
....uugaaagucagcccucgacacaaggguuuCu......................	3	1	7y1
....uugaaagucagcccucgacacaaggguuugG......................	3	1	7y1
....uugaaagucagcccucgCcacaaggguuugu......................	4	1	7y1
....uugaaaguAagcccucgacacaaggguuugu......................	4	1	7y1
....uugaaagucagcccucgacacaaggguuAgu......................	6	1	7y1

Star **Mature**

gaccugcuucugggucgggguuucguacguagcagagcagcucccucgcugcgaucuauugaaagucagcccucgacacaaggguuuguccgcgcgcgcgcgcgcgcgcgugc			
....uugaaagucagcccucgacacaagCguuugu........................	1	1	7y1
....uugaaagucagcAcucgacacaaggguuugu........................	1	1	7y1
....Gugaaagucagcccucgacacaaggguuugu........................	3	1	7y1
....uugaaagucagccAucgacacaaggguuugu........................	3	1	7y1
....uugaaagucagcccucgacacaaggguuugu........................	1439	0	7y1
....uugaaagucagcccuAgacacaaggguuugu........................	2	1	7y1
....uugaaagucagcccucgacacaagUguuugu........................	1	1	7y1
....Augaaagucagcccucgacacaaggguuugu........................	10	1	7y1
....uugaaagucagcccucgacacaaggguuuUu........................	2	1	7y1
....uugaaagucagcccucgacacaaggCuuugu........................	1	1	7y1
....uugaaagucagGccucgacacaaggguuugu........................	1	1	7y1
....uugaaagucagcccucgacacaaUgguuugu........................	1	1	7y1
....uugaaagucagccUucgacacaaggguuugu........................	1	1	7y1
....uugaaagucagcccucgacacaaggguAugu........................	4	1	7y1
....uugaaagucGgcccucgacacaaggguuugu........................	1	1	7y1
....uuCaaagucagcccucgacacaaggguuugu........................	2	1	7y1
....uugaaagucagcccucgacacaaggguGugu........................	1	1	7y1
....uugaaaCucagcccucgacacaaggguuugu........................	1	1	7y1
....uugaaagAcagcccucgacacaaggguuugu........................	1	1	7y1
....uugaaagucagcccucgacacaaggguuugA........................	4	1	7y1
....uAgaaagucagcccucgacacaaggguuugu........................	2	1	7y1
....uugaaagucagcccucgacacaagggGuugu........................	3	1	7y1
....uugaaagucagcccucgacacaaCgguuugu........................	2	1	7y1
....uCgaaagucagcccucgacacaaggguuugu........................	2	1	7y1
....Agaaagucagcccucgac....................................	4	1	7y1
....ugaaagAcagcccucgac....................................	1	1	7y1
....ugaaaguAagcccucgac....................................	2	1	7y1
....ugaaagucagcccucgac....................................	191	0	7y1
....ugaaagucagUccucgac....................................	1	1	7y1
....ugaaagucagcccucgCc....................................	1	1	7y1
....ugaaagucagcccucCacacaaggguuug.........................	1	1	7y1
....ugaaagucagcccucgacacaagggAuug.........................	2	1	7y1
....ugaaagucagcccGcgacacaaggguuug.........................	1	1	7y1
....ugaaagAcagcccucgacacaaggguuug.........................	1	1	7y1
....ugaaagucagccGucgacacaaggguuug.........................	1	1	7y1
....ugaaagucagcccucgacacaaggUuuug.........................	1	1	7y1
....ugaaagucagcccucgCcacaaggguuug.........................	1	1	7y1
....ugaaagucagcccucgacacaaUgguuug.........................	1	1	7y1
....uAaaagucagcccucgacacaaggguuug.........................	1	1	7y1
....ugaaagucagcccucgacacGaggguuug.........................	1	1	7y1
....ugaaagucagcccucgacacaaggguuug.........................	462	0	7y1
....ugaaagucagAccucgacacaaggguuug.........................	1	1	7y1
....ugaaagucagcccucgacacaaggguAug.........................	1	1	7y1
....ugaaagucagcccucgacacaaggguGug.........................	1	1	7y1
....Ggaaagucagcccucgacacaaggguuug.........................	1	1	7y1
....ugaaagucUgcccucgacacaaggguuug.........................	1	1	7y1
....ugaaagucagcccucgacacaaggguuGg.........................	2	1	7y1
....gaaagucagcccucgacaUaaggguuugu........................	1	1	7y1
....gaaagucagcccucgacCcaaggguuugu........................	1	1	7y1
....gaaagucagAccucgacacaaggguuugu........................	1	1	7y1
....gaaagucagcccAcgacacaaggguuugu........................	1	1	7y1
....gaaagucagcccucgacacaaggguuAgu........................	1	1	7y1
....Caaagucagcccucgacacaaggguuugu........................	1	1	7y1
....gaaagucagcccucgacacaaggguuugu........................	170	0	7y1
....gaGagucagcccucgacacaaggguuugu........................	1	1	7y1
....gaaagucagcccucgacacaagggGuugu........................	1	1	7y1
....aagucagcccucgacacaaggguuuC.........................	2	1	7y1
....aagucagcccucgacacaaggguuug.........................	211	0	7y1
....aagucagcccucgacUcaaggguuug.........................	2	1	7y1
....aagucagccAucgacacaaggguuug.........................	2	1	7y1
....aagucagcccucgacacaaggUuuug.........................	1	1	7y1
....uAagcccucgacacaaggguuug.........................	1	1	7y1
....ucagcccucgGcacaaggguuug.........................	1	1	7y1
....Acagcccucgacacaaggguuug.........................	3	1	7y1
....Ncagcccucgacacaaggguuug.........................	1	1	7y1
....ucagccAucgacacaaggguuug.........................	1	1	7y1
....ucagcccucgacacaaggguuug.........................	244	0	7y1
....ucagcccucgacacaagggAuug.........................	1	1	7y1
....Gcagcccucgacacaaggguuug.........................	1	1	7y1
....cagcUcucgacacaaggguuugu........................	211	1	7y1

Star **Mature**

```
gaccugcuucugggucgggguuucguacguagcagagcagcucccucgcugcgaucuauugaaagucagcccucgacacaaggguuuguccgcgcgcgcgcgcgcgcgcgcgugc
...................................................................agccUucgacacaaggguuug........................... 132 1 7y1
....................................................................gcAcucgacacaaggguuug........................... 1 1 7y1
....................................................................gccAucgacacaaggguuug........................... 1 1 7y1
....................................................................Ncccucgacacaaggguuug........................... 1 1 7y1
....................................................................gAccucgacacaaggguuug........................... 2 1 7y1
....................................................................gcccucgaAacaaggguuug........................... 2 1 7y1
....................................................................gcccucgacacaaggguuug........................... 188 0 7y1
....................................................................gcccucAacacaaggguuug........................... 1 1 7y1
.....................................................................cccucgacacaaggguuuU........................... 1 1 7y1
.....................................................................ccAucgacacaaggguuugu.......................... 2 1 7y1
.....................................................................Nccucgacacaaggguuugu.......................... 1 1 7y1
.....................................................................cccucgacacaaggguuugu.......................... 289 0 7y1
.....................................................................cccucgacacaagggAuugu.......................... 2 1 7y1
.....................................................................cccucgacacaaggCuuugu.......................... 2 1 7y1
.....................................................................cccucgacaAaaggguuugu.......................... 1 1 7y1
.....................................................................cccucgacacaaggguuugG.......................... 2 1 7y1
.....................................................................cccucgacUcaaggguuugu.......................... 1 1 7y1
.....................................................................cccucgacacaaUgguuugu.......................... 1 1 7y1
......................................................................Gcucgacacaaggguuug........................... 4 1 7y1
......................................................................ccucgacacaagAguuug........................... 1 1 7y1
......................................................................ccucgacaUaaggguuug........................... 1 1 7y1
......................................................................ccucUacacaaggguuug........................... 3 1 7y1
......................................................................cAucgacacaaggguuug........................... 6 1 7y1
......................................................................ccucgacUcaaggguuug........................... 2 1 7y1
......................................................................ccCcgacacaaggguuug........................... 1 1 7y1
......................................................................ccucgacacaaggguAug........................... 4 1 7y1
......................................................................ccucgacacaaggguuGg........................... 1 1 7y1
......................................................................ccucgaAacaaggguuug........................... 3 1 7y1
......................................................................ccAcgacacaaggguuug........................... 1 1 7y1
......................................................................ccucgacacaagCguuug........................... 2 1 7y1
......................................................................ccucgacacaaUgguuug........................... 1 1 7y1
......................................................................Ncucgacacaaggguuug........................... 2 1 7y1
......................................................................ccuGgacacaaggguuug........................... 1 1 7y1
......................................................................Acucgacacaaggguuug........................... 8 1 7y1
......................................................................ccucgacacaaggguuAg........................... 1 1 7y1
......................................................................ccucgacacaaggguuuA........................... 1 1 7y1
......................................................................ccucgacacaaggguuuC........................... 1 1 7y1
......................................................................ccucgacacaUggguuug........................... 1 1 7y1
......................................................................ccucgacacaaggCuuug........................... 1 1 7y1
......................................................................ccucgacacaaggguuug........................... 1954 0 7y1
......................................................................ccucgCcacaaggguuug........................... 2 1 7y1
......................................................................ccucgacacGaggguuug........................... 1 1 7y1
......................................................................ccucgacacaaUgguuugu.......................... 5 1 7y1
......................................................................ccucgacacNaggguuugu.......................... 1 1 7y1
......................................................................ccucgacacaaggguAugu.......................... 8 1 7y1
......................................................................ccucgacacaGggguuugu.......................... 1 1 7y1
......................................................................ccucUacacaaggguuugu.......................... 7 1 7y1
......................................................................ccucgacacaaggguuuAu.......................... 1 1 7y1
......................................................................ccucgacacaaggguuugA.......................... 12 1 7y1
......................................................................ccucgacacaagUguuugu.......................... 6 1 7y1
......................................................................ccucgUcacaaggguuugu.......................... 1 1 7y1
......................................................................ccucgacaUaaggguuugu.......................... 153 1 7y1
......................................................................Ucucgacacaaggguuugu.......................... 358 1 7y1
......................................................................Acucgacacaaggguuugu.......................... 24 1 7y1
......................................................................ccucgacacaaggguuuCu.......................... 3 1 7y1
......................................................................ccucgacGcaaggguuugu.......................... 3 1 7y1
......................................................................ccucgacacaaggUuuugu.......................... 9 1 7y1
......................................................................cAucgacacaaggguuugu.......................... 17 1 7y1
......................................................................ccucgacacaaCgguuugu.......................... 3 1 7y1
......................................................................ccucgacacaaggCuuugu.......................... 13 1 7y1
......................................................................ccucgCcacaaggguuugu.......................... 7 1 7y1
......................................................................ccucgacacaaggguuugG.......................... 17 1 7y1
......................................................................ccuGgacacaaggguuugu.......................... 1 1 7y1
......................................................................Ncucgacacaaggguuugu.......................... 3 1 7y1
......................................................................ccucgacacaagCguuugu.......................... 4 1 7y1
......................................................................ccucgaUacaaggguuugu.......................... 1 1 7y1
......................................................................ccucgacacaaggguuugC.......................... 1 1 7y1
......................................................................ccAcgacacaaggguuugu.......................... 14 1 7y1
......................................................................ccucgaAacaaggguuugu.......................... 9 1 7y1
......................................................................ccucgacaAaaggguuugu.......................... 8 1 7y1
```

Star **Mature**

```
gaccugcuucugggucggggguuucguacguagcagagcagcucccucgcugcgaucuauugaaagucagcccucgacacaaggguuuguccgcgcgcgcgcgcgcgcgugc
......................................................................ccucgacacGaggguuugu......................  1      1  7y1
......................................................................ccucgacacaaggguuugu......................  6344   0  7y1
......................................................................ccucgacacaaggguuuUu......................  5      1  7y1
......................................................................ccucgacacaagggGuugu......................  4      1  7y1
......................................................................ccucgGcacaaggguuugu......................  1      1  7y1
......................................................................Gcucgacacaaggguuugu......................  5      1  7y1
......................................................................ccucAacacaaggguuugu......................  4      1  7y1
......................................................................ccucgacacaagggAuugu......................  8      1  7y1
......................................................................ccucgacacaaggguuAgu......................  9      1  7y1
......................................................................ccucgacacaaggguCugu......................  1      1  7y1
......................................................................cNucgacacaaggguuuguccgcgc................  1      1  7y1
......................................................................ccucgacacaaggguuuguccgcgA................  1      1  7y1
......................................................................ccAcgacacaaggguuuguccgcgc................  1      1  7y1
......................................................................ccucgacacaaggguuuguccgcgc................  277    0  7y1
......................................................................ccucgacacaaggguAuguccgcgc................  1      1  7y1
......................................................................ccucgacacaaggCuuuguccgcgc................  1      1  7y1
......................................................................ccucgacacaaggUuuuguccgcgc................  1      1  7y1
......................................................................ccucgacacaagggAuuguccgcgc................  1      1  7y1
......................................................................ccuUgacacaaggguuuguccgcgc................  1      1  7y1
......................................................................cAucgacacaaggguuuguccgcgc................  1      1  7y1
.......................................................................cucgacacaaggguuAgu......................  30     1  7y1
.......................................................................cucgacacGaggguuugu......................  5      1  7y1
.......................................................................cucgacacaagggGuugu......................  22     1  7y1
.......................................................................cucgacacaaggguCugu......................  2      1  7y1
.......................................................................Gucgacacaaggguuugu......................  18     1  7y1
.......................................................................cNcgacacaaggguuugu......................  2      1  7y1
.......................................................................cucAacacaaggguuugu......................  13     1  7y1
.......................................................................Nucgacacaaggguuugu......................  10     1  7y1
.......................................................................cucgacUcaaggguuugu......................  9      1  7y1
.......................................................................cucgacacaaggguuugA......................  39     1  7y1
.......................................................................cucUacacaaggguuugu......................  14     1  7y1
.......................................................................cucgacacaaggguuCgu......................  1      1  7y1
.......................................................................cucgacacaaggguuuUu......................  19     1  7y1
.......................................................................cucgaUacaaggguuugu......................  3      1  7y1
.......................................................................cucgacacaaggguuugu......................  25979  0  7y1
.......................................................................cuGgacacaaggguuugu......................  197    1  7y1
.......................................................................cucgacacaaggCuuugu......................  17     1  7y1
.......................................................................cucgacacaagCguuugu......................  18     1  7y1
.......................................................................cucgacaUaaggguuugu......................  3      1  7y1
.......................................................................cucgacacaagUguuugu......................  175    1  7y1
.......................................................................cucgacacaaggguuugC......................  1      1  7y1
.......................................................................cucgacacaaCgguuugu......................  17     1  7y1
.......................................................................cucgacGcaaggguuugu......................  6      1  7y1
.......................................................................cucgacacaGggguuugu......................  1      1  7y1
.......................................................................cucgacacaaAgguuugu......................  197    1  7y1
.......................................................................cucgacacaaggguuugG......................  34     1  7y1
.......................................................................cucgacacaaggguuGgu......................  1      1  7y1
.......................................................................cucgacacaaUgguuugu......................  17     1  7y1
.......................................................................cucgaAacaaggguuugu......................  34     1  7y1
.......................................................................cucgacCcaaggguuugu......................  1      1  7y1
.......................................................................cucgacacCaggguuugu......................  1      1  7y1
.......................................................................cAcgacacaaggguuugu......................  75     1  7y1
.......................................................................cucgacacaaggguuuCu......................  23     1  7y1
.......................................................................cucgaNacaaggguuugu......................  1      1  7y1
.......................................................................cGcgacacaaggguuugu......................  2      1  7y1
.......................................................................cucgacacaaggguuuAu......................  7      1  7y1
.......................................................................cucgacacaaggguGugu......................  5      1  7y1
.......................................................................cucgacacaaggguAugu......................  35     1  7y1
.......................................................................cCcgacacaaggguuugu......................  2      1  7y1
.......................................................................cucgacacaagggAuugu......................  43     1  7y1
.......................................................................cucgacacaaggUuuugu......................  13     1  7y1
.......................................................................cucgacacaaggAuuugu......................  3      1  7y1
.......................................................................cucgacacUaggguuugu......................  2      1  7y1
.......................................................................cucgaGacaaggguuugu......................  2      1  7y1
.......................................................................cucgCcacaaggguuugu......................  4      1  7y1
.......................................................................cucgacaAaaggguuugu......................  21     1  7y1
.......................................................................cucgacacaUggguuugu......................  2      1  7y1
.......................................................................cucgacaGaaggguuugu......................  1      1  7y1
.......................................................................cucgUcacaaggguuugu......................  2      1  7y1
.......................................................................cucgacacaaggguuuguccgcgc................  1      0  7y1
```

Star **Mature**

gaccugcuucugggucgggguuucguacguagcagagcagcucccucgcugcgaucuauugaaagucagcccucgacacaaggguuuguccgcgcgcgcgcgcgcgcgugc
