# Supplementary material for: SEAweb: the small RNA Expression Atlas web application
Source: Nucleic Acids Res. 2019 Oct 10;48(D1):D204–19. doi: 10.1093/nar/gkz869 (PMC6943056; doi:10.1093/nar/gkz869)
Supplement: gkz869_Supplemental_Files [file gkz869_supplemental_files.zip › p-hsa-miR-235-2.pdf]

Provisional ID	: chr22_KI270733v1_random_12920
Score total	: 144078.7
Score for star read(s)	: 3.9
Score for read counts	: 144071.6
Score for mfe	: 2.2
Score for randfold	: 1.6
Score for cons. seed	: -0.6
Total read count	: 282602
Mature read count	: 36506
Loop read count	: 245911
Star read count	: 185

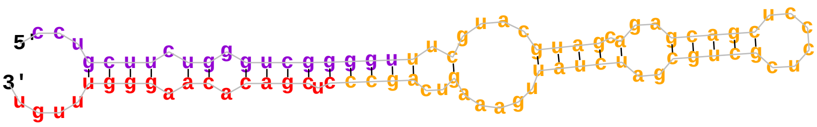

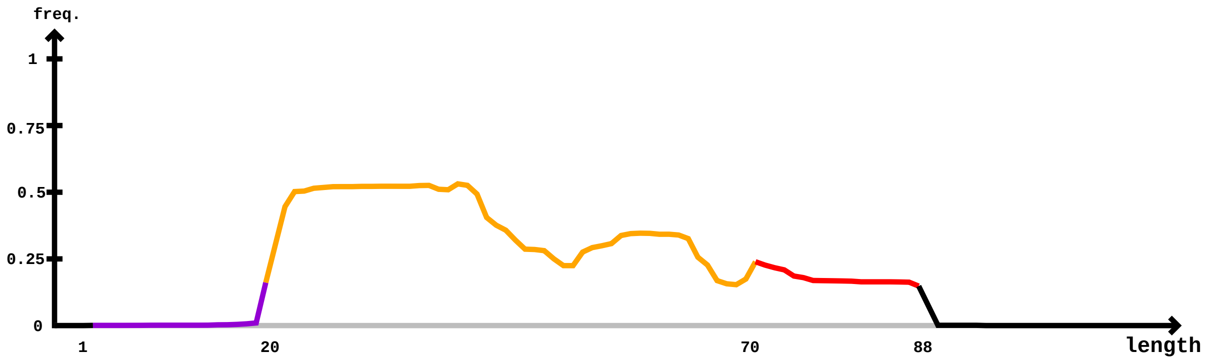

Star **Mature**

```
5'- gaccugcuucugggucggggguuucguacguagcagagcagcagcucccucgcugcgaucuauugaaagucagcccucgacacaaggguuuguccgcgcgcgcgcgcgcgcgugcgu   -3'   obs
    gaccugcuucugggucggggguuucguacguagcagagcagcagcucccucgcugcgaucuauugaaagucagcccucgacacaaggguuuguccgcgcgcgcgcgcgcgcgugcgu          exp
    (((..((((.((.(((((((((.(....((((.(..(((((......)))))..))))).....)..))))).)))).)).)))).)))((((((((((....)))))))))).   reads   mm   sample
    ..ccugcuucugggucgggg................................................................................................   7       0    7y1
    ..ccugcuucugggucggggA...............................................................................................   1       1    7y1
    ..ccugcuucugggucggggG...............................................................................................   6       1    7y1
    ..cAugcuucugggucggggu...............................................................................................   1       1    7y1
    ..ccugcuucugggucggggu...............................................................................................   164     0    7y1
    ...cugcuucuggguAgggguuucgu..........................................................................................   2       1    7y1
    ...cugcuucugggucgggguAucgu..........................................................................................   1       1    7y1
    ...cugcuucugUgucgggguuucgu..........................................................................................   1       1    7y1
    ...cugcuucugggucgggUuuucgu..........................................................................................   2       1    7y1
    ....ugcuucAgggucgggguu..............................................................................................   1       1    7y1
    .....gcuucuggCucgggguuucgu..........................................................................................   1       1    7y1
    .....gcuucugggAcgggguuucgu..........................................................................................   1       1    7y1
    .....gcuucuggguAgggguuucgu..........................................................................................   1       1    7y1
    .....gcuucuggUucgggguuucgu..........................................................................................   1       1    7y1
    ......cuucugggucgggguuuG............................................................................................   1       1    7y1
    ......cuucugggAcgggguuuc............................................................................................   1       1    7y1
    ......cuucugggucgggguGuc............................................................................................   1       1    7y1
    ......cuucuAggucgggguuuc............................................................................................   1       1    7y1
    ......cuucugggucggCguuuc............................................................................................   1       1    7y1
    ......cuucuUggucgggguuucgu..........................................................................................   1       1    7y1
    ......cuucugggAcgggguuucgu..........................................................................................   2       1    7y1
    ......cuucuCggucgggguuucgu..........................................................................................   1       1    7y1
    ......cuucuggguAgggguuucgu..........................................................................................   4       1    7y1
    ......cuucugggucCggguuucgu..........................................................................................   1       1    7y1
    ......cuucugggucggCguuucgu..........................................................................................   1       1    7y1
    ......cuucugggucUggguuucgu..........................................................................................   1       1    7y1
    ......cuucugggucggggGuucgu..........................................................................................   2       1    7y1
    ......cuucugggucgggAuuucgu..........................................................................................   1       1    7y1
    ......cuucugggucgggUuuucgu..........................................................................................   1       1    7y1
    ......cuucugggucgggguuucUu..........................................................................................   3       1    7y1
    ......cuucugggucgggguCucgu..........................................................................................   1       1    7y1
    ......cuucugggucgggguAucgu..........................................................................................   1       1    7y1
    ......cuucugggucggggAuucgu..........................................................................................   1       1    7y1
```

Star   Mature

```
gaccugcuucugggucgggguuucguacguagcagagcagcucccucgcugcgaucuauugaaagucagcccucgacacaaggguuuguccgcgcgcgcgcgcgcgcgugcgu
......cuucuggguGgggguuucgu.......................................................................................   1    1    7y1
......cuucugggucggUguuucgu.......................................................................................   1    1    7y1
.......uucugggAcgggguuucgu.......................................................................................   3    1    7y1
.......uucugggucgUgguuucgu.......................................................................................   2    1    7y1
.......uucuggguAgggguuucgu.......................................................................................   6    1    7y1
.......uucugCgucgggguuucgu.......................................................................................   4    1    7y1
.......uucugggucUggguuucgu.......................................................................................   2    1    7y1
.......uucugggucggggCuucgu.......................................................................................   1    1    7y1
.......uucugggucggCguuucgu.......................................................................................   1    1    7y1
.......uucuUggucgggguuucgu.......................................................................................   2    1    7y1
.......uucugggucggUguuucgu.......................................................................................   1    1    7y1
.......uucugggucgggCuuucgu.......................................................................................   4    1    7y1
.......uucugggucgggguAucgu.......................................................................................   3    1    7y1
.......uucugggucggggGuucgu.......................................................................................   1    1    7y1
.......uucuggUucgggguuucgu.......................................................................................   2    1    7y1
.......uucugggucgggguCucgu.......................................................................................   2    1    7y1
.......uucugUgucgggguuucgu.......................................................................................   1    1    7y1
........ucuggCucgggguuucgu.......................................................................................   4    1    7y1
........ucugggucgggCuuucgu.......................................................................................   1    1    7y1
........ucugCgucgggguuucgu.......................................................................................   1    1    7y1
........ucugggucgUgguuucgu.......................................................................................   1    1    7y1
........ucuggguAgggguuucgu.......................................................................................   4    1    7y1
........ucugggucgCgguuucgu.......................................................................................   8    1    7y1
........ucuggAucgggguuucgu.......................................................................................   1    1    7y1
........ucugggucggggAuucgu.......................................................................................   1    1    7y1
........ucugggucggCguuucgu.......................................................................................   6    1    7y1
........ucugggucgggguAucgu.......................................................................................   4    1    7y1
........ucugggucCggguuucgu.......................................................................................   2    1    7y1
........ucugggucgggguCucgu.......................................................................................   1    1    7y1
........ucugUgucgggguuucgu.......................................................................................   2    1    7y1
........ucugggucgggUuuucgu.......................................................................................   1    1    7y1
........ucuAggucgggguuucgu.......................................................................................   2    1    7y1
........ucuggUucgggguuucgu.......................................................................................   7    1    7y1
........ucugggucggUguuucgu.......................................................................................   3    1    7y1
........ucugggucUggguuucgu.......................................................................................   2    1    7y1
........ucugggAcgggguuucgu.......................................................................................   4    1    7y1
........ucuggguGgggguuucgu.......................................................................................   1    1    7y1
........ucugggucgggguuAcgu.......................................................................................   1    1    7y1
........ucugggucgggUuuucgua......................................................................................   2    1    7y1
........ucugggucggggAuucgua......................................................................................   1    1    7y1
........ucugggucgggguuAcgua......................................................................................   1    1    7y1
........ucugggucUggguuucgua......................................................................................   3    1    7y1
........ucuggguAgggguuucgua......................................................................................   4    1    7y1
........ucugggucgggCuuucgua......................................................................................   1    1    7y1
........ucugggucgUgguuucgua......................................................................................   3    1    7y1
........ucuggguGgggguuucgua......................................................................................   1    1    7y1
........ucugggucgggguuGcgua......................................................................................   1    1    7y1
........ucugggucggUguuucgua......................................................................................   4    1    7y1
........ucugggucggCguuucgua......................................................................................   2    1    7y1
........ucugUgucgggguuucgua......................................................................................   2    1    7y1
........ucugggucgCgguuucgua......................................................................................   6    1    7y1
........ucuggAucgggguuucgua......................................................................................   1    1    7y1
........ucugggAcgggguuucgua......................................................................................   12   1    7y1
........ucugggucggAguuucgua......................................................................................   1    1    7y1
........ucugggucCggguuucgua......................................................................................   2    1    7y1
........ucugCgucgggguuucgua......................................................................................   2    1    7y1
........ucuggUucgggguuucgua......................................................................................   5    1    7y1
........ucugggucgggguAucgua......................................................................................   2    1    7y1
.........cugggucgggguuuAgua......................................................................................   5    1    7y1
.........cugggucgggUuuucgua......................................................................................   1    1    7y1
.........cugggucgCgguuucgua......................................................................................   1    1    7y1
.........cugggucgAgguuucgua......................................................................................   1    1    7y1
.........cugggucgggguuuGgua......................................................................................   1    1    7y1
.........cugCgucgggguuucgua......................................................................................   1    1    7y1
.........cuggguAgggguuucgua......................................................................................   5    1    7y1
.........cugggucUggguuucgua......................................................................................   1    1    7y1
.........cugggucggUguuucgua......................................................................................   1    1    7y1
.........cugggucggggGuucgua......................................................................................   1    1    7y1
.........cugggucgggguuAcguac.....................................................................................   1    1    7y1
.........cugggucgggguuuGguac.....................................................................................   1    1    7y1
```

Star **Mature**

gaccugcuucugggucggggguuucguacguagcagagcagcucccucgcugcgaucuauugaaagucagcccucgacacaaggguuuguccgcgcgcgcgcgcgcgcgugcgu			
.........cugggucgggguuuAguac...	1	1	7y1
.........cuggguAgggguuucguac...	1	1	7y1
.........cugggucCggguuucguac...	1	1	7y1
.........cugggucggCguuucguac...	1	1	7y1
.........cugggAcgggguuucguac...	1	1	7y1
.........cugggucgggUuuucguac...	1	1	7y1
..............ucgggguAucguacguag...	1	1	7y1
..............ucggggAuucguacguag...	1	1	7y1
..............ucggggAuucguacguagc..	1	1	7y1
..............ucgggUuuucguacguagc..	1	1	7y1
..............ucggggCuucguacguagc..	1	1	7y1
..............ucgggguAucguacguagc..	1	1	7y1
..............ucggggGuucguacguagc..	1	1	7y1
..............ucgggguuucguaUguagc..	1	1	7y1
..............ucgggguuucAuacguagcagagc...	2	1	7y1
..............ucgggguGucguacguagcagagcagcuc..	1	1	7y1
..............ucgggguCucguacguagcagagcagcuc..	1	1	7y1
..............ucgggCuuucguacguagcagagcagcuc..	6	1	7y1
..............ucgggUuuucguacguagcagagcagcuc..	4	1	7y1
..............ucggggCuucguacguagcagagcagcuc..	2	1	7y1
..............ucggggAuucguacguagcagagcagcuc..	23	1	7y1
...............cgggguuucgCacguagc..	1	1	7y1
...............cggAguuucguacguagc..	1	1	7y1
...............cggggAuucguacguagc..	2	1	7y1
...............cgggguuucguaAguagc..	1	1	7y1
...............cgggguuAcguacguagc..	1	1	7y1
...............cgggguuucUuacguagc..	1	1	7y1
...............cggCguuucguacguagc..	1	1	7y1
...............cggggu Aucguacguagc..	3	1	7y1
...............cgggguuucguacAuagca...	1	1	7y1
...............cggggAuucguacguagca...	2	1	7y1
...............cgggguuucUuacguagca...	1	1	7y1
...............cgggguuucguaAguagca...	1	1	7y1
...............cgggguuucCuacguagcag..	1	1	7y1
...............cgggguuucguacCuagcag..	1	1	7y1
...............Agggguuucguacguagcag..	1	1	7y1
...............cgggguuucguacgAagcag..	1	1	7y1
...............cggggAuucguacguagcag..	1	1	7y1
...............cgggguuuAguacguagcag..	1	1	7y1
...............cgggUuuucguacguagcag..	1	1	7y1
...............cggggu Gucguacguagcaga...	1	1	7y1
...............cgggguuAcguacguagcaga...	2	1	7y1
...............cgggguuuAguacguagcaga...	1	1	7y1
...............cgggguuucUuacguagcaga...	1	1	7y1
...............cgggguuucCuacguagcaga...	1	1	7y1
...............cgggguuucguaGguagcaga...	1	1	7y1
...............cgggguuucguacUuagcaga...	4	1	7y1
...............cgggCuuucguacguagcaga...	3	1	7y1
...............cgggguuucguaAguagcaga...	3	1	7y1
...............cgggguuuGguacguagcaga...	1	1	7y1
...............cgggguuucguacgCagcaga...	1	1	7y1
...............cggggu Aucguacguagcaga...	4	1	7y1
...............cggggAuucguacguagcaga...	5	1	7y1
...............cgggguuuUguacguagcaga...	1	1	7y1
...............cggggGuucguacguagcaga...	1	1	7y1
...............cgggguuucAuacguagcagag..	1	1	7y1
...............cgggguuuAguacguagcagag..	1	1	7y1
...............cgggguuucguacUuagcagag..	1	1	7y1
...............cgggUuuucguacguagcagag..	1	1	7y1
...............cgggguuucUuacguagcagag..	1	1	7y1
...............cggggu Aucguacguagcagag..	2	1	7y1
...............cgggguuucguaAguagcagag..	3	1	7y1
...............cgggguuucgAacguagcagag..	1	1	7y1
...............cgggguuucguacUuagcagagc...	2	1	7y1
...............cgggCuuucguacguagcagagc...	3	1	7y1
...............cgggguuuAguacguagcagagc...	7	1	7y1
...............cgggguuucgGacguagcagagc...	1	1	7y1
...............cgggguuucCuacguagcagagc...	2	1	7y1
...............cggggGuucguacguagcagagc...	3	1	7y1
...............cgggguuucgAacguagcagagc...	3	1	7y1

Star **Mature**

```
gaccugcuucugggucgggguuucguacguagcagagcagcucccucgcugcgaucuauugaaagucagcccucgacacaaggguuuguccgcgcgcgcgcgcgcgcgugcgu
...............cgggguuucguacCuagcagagc...........................................................................    1    1    7y1
...............cgggguuucguaAguagcagagc...........................................................................    11   1    7y1
...............cggggAuucguacguagcagagc...........................................................................    27   1    7y1
...............cgggguuucUuacguagcagagc...........................................................................    1    1    7y1
...............cgggguuCcguacguagcagagc...........................................................................    2    1    7y1
...............cgggguuucAuacguagcagagc...........................................................................    1    1    7y1
...............cgggguuucguacgAagcagagc...........................................................................    3    1    7y1
...............cgggguuucgCacguagcagagc...........................................................................    3    1    7y1
...............cggggUAucguacguagcagagc...........................................................................    14   1    7y1
...............cgggUuuucguacguagcagagc...........................................................................    2    1    7y1
...............cgggguuucguGcguagcagagc...........................................................................    1    1    7y1
...............cgggguuucguacAuagcagagc...........................................................................    1    1    7y1
...............cgggguuuGguacguagcagagc...........................................................................    1    1    7y1
...............cggggUCucguacguagcagagc...........................................................................    1    1    7y1
...............cgggguuAcguacguagcagagc...........................................................................    3    1    7y1
...............cgggguuucguaUguagcagagc...........................................................................    1    1    7y1
...............cgggguuucguacgAagcagagca..........................................................................    3    1    7y1
...............cgggguuucgAacguagcagagca..........................................................................    3    1    7y1
...............cgggguuucCuacguagcagagca..........................................................................    2    1    7y1
...............cgggguuucguaAguagcagagca..........................................................................    4    1    7y1
...............cgggUuuucguacguagcagagca..........................................................................    1    1    7y1
...............cgggguuucgCacguagcagagca..........................................................................    1    1    7y1
...............cgggguuucguacUuagcagagca..........................................................................    2    1    7y1
...............cgggguuucguacgGagcagagca..........................................................................    1    1    7y1
...............cgggguuucguGcguagcagagca..........................................................................    2    1    7y1
...............cgggguuAcguacguagcagagca..........................................................................    6    1    7y1
...............cgggCuuucguacguagcagagca..........................................................................    3    1    7y1
...............cgggguuuAguacguagcagagca..........................................................................    5    1    7y1
...............cggggAuucguacguagcagagca..........................................................................    10   1    7y1
...............cgggguuucAuacguagcagagca..........................................................................    1    1    7y1
...............cggggGuucguacguagcagagca..........................................................................    1    1    7y1
...............cgggguuucgGacguagcagagca..........................................................................    1    1    7y1
...............cgggguuuUguacguagcagagca..........................................................................    1    1    7y1
...............cgggguuucUuacguagcagagca..........................................................................    4    1    7y1
...............cggggUAucguacguagcagagca..........................................................................    10   1    7y1
...............cgggAuuucguacguagcagagca..........................................................................    1    1    7y1
...............cggggUCucguacguagcagagca..........................................................................    1    1    7y1
...............cgggCuuucguacguagcagagcag.........................................................................    1    1    7y1
...............cgggguuucguacUuagcagagcag.........................................................................    1    1    7y1
...............cgggguuucgAacguagcagagcag.........................................................................    1    1    7y1
...............cgggguuucCuacguagcagagcag.........................................................................    1    1    7y1
...............cgggguuuAguacguagcagagcag.........................................................................    4    1    7y1
...............cggggUAucguacguagcagagcag.........................................................................    1    1    7y1
...............cgggguuAcguacguagcagagcag.........................................................................    1    1    7y1
...............cgggUuuucguacguagcagagcag.........................................................................    2    1    7y1
...............cgggguuuGguacguagcagagcag.........................................................................    1    1    7y1
...............cgggguuucguaAguagcagagcag.........................................................................    1    1    7y1
...............cggggCuucguacguagcagagcagc........................................................................    1    1    7y1
...............cgggguuuAguacguagcagagcagc........................................................................    2    1    7y1
...............cgggguuucguaAguagcagagcagc........................................................................    5    1    7y1
...............cgggguuucguacCuagcagagcagc........................................................................    1    1    7y1
...............cggggUAucguacguagcagagcagc........................................................................    9    1    7y1
...............cggggUGucguacguagcagagcagc........................................................................    1    1    7y1
...............cgggguuAcguacguagcagagcagc........................................................................    2    1    7y1
...............cgggguuucguaGguagcagagcagc........................................................................    1    1    7y1
...............cgggguuucgAacguagcagagcagc........................................................................    4    1    7y1
...............cgggguuCcguacguagcagagcagc........................................................................    1    1    7y1
...............cgggguuucAuacguagcagagcagc........................................................................    2    1    7y1
...............cgggguuucguacgAagcagagcagc........................................................................    2    1    7y1
...............cggggGuucguacguagcagagcagc........................................................................    1    1    7y1
...............cggggAuucguacguagcagagcagc........................................................................    10   1    7y1
...............cgggguuucgGacguagcagagcagc........................................................................    1    1    7y1
...............cgggguuucguacUuagcagagcagc........................................................................    2    1    7y1
...............cgggguuuUguacguagcagagcagc........................................................................    1    1    7y1
...............cgggguuucguacUuagcagagcagcu.......................................................................    2    1    7y1
...............cgggguuuAguacguagcagagcagcu.......................................................................    1    1    7y1
...............cggggAuucguacguagcagagcagcu.......................................................................    10   1    7y1
...............cgggguuucguGcguagcagagcagcu.......................................................................    1    1    7y1
...............cgggUuuucguacguagcagagcagcu.......................................................................    2    1    7y1
...............cggggUAucguacguagcagagcagcu.......................................................................    12   1    7y1
```

Star **Mature**

```
gaccugcuucugggucggggguuucguacguagcagagcagcucccucgcugcgaucuauugaaagucagcccucgacacaaggguuuguccgcgcgcgcgcgcgcgcgugcgu
...............cgggguuucguacgAagcagagcagcu.......................................................................	1	1	7y1
...............cgggguuucgAacguagcagagcagcu.......................................................................	4	1	7y1
...............cgggguuucCuacguagcagagcagcu.......................................................................	1	1	7y1
...............cgggguuucAuacguagcagagcagcu.......................................................................	2	1	7y1
...............cgggguuucgGacguagcagagcagcu.......................................................................	1	1	7y1
...............cgggguuuUguacguagcagagcagcu.......................................................................	1	1	7y1
...............cgggguCucguacguagcagagcagcu.......................................................................	1	1	7y1
...............cgggguuucguaAguagcagagcagcu.......................................................................	2	1	7y1
...............cgggguuAcguacguagcagagcagcu.......................................................................	4	1	7y1
...............cggggGuucguacguagcagagcagcu.......................................................................	3	1	7y1
...............cggggCuucguacguagcagagcagcu.......................................................................	1	1	7y1
...............cgggguuucUuacguagcagagcagcu.......................................................................	1	1	7y1
...............cgggguGucguacguagcagagcagcuc......................................................................	2	1	7y1
...............cggggGuucguacguagcagagcagcuc......................................................................	4	1	7y1
...............cgggguuucUuacguagcagagcagcuc......................................................................	1	1	7y1
...............cgggguuuGguacguagcagagcagcuc......................................................................	1	1	7y1
...............cgggguuucguacUuagcagagcagcuc......................................................................	3	1	7y1
...............cgggguuucCuacguagcagagcagcuc......................................................................	1	1	7y1
...............cggggAuucguacguagcagagcagcuc......................................................................	12	1	7y1
...............cgggguuucguacCuagcagagcagcuc......................................................................	1	1	7y1
...............cgggguAucguacguagcagagcagcuc......................................................................	16	1	7y1
...............cgggguuucguacgAagcagagcagcuc......................................................................	1	1	7y1
...............cgggguuucAuacguagcagagcagcuc......................................................................	2	1	7y1
...............cgggUuuucguacguagcagagcagcuc......................................................................	2	1	7y1
...............cgggguuAcguacguagcagagcagcuc......................................................................	7	1	7y1
...............cgggguuucgAacguagcagagcagcuc......................................................................	2	1	7y1
...............cgggCuuucguacguagcagagcagcuc......................................................................	2	1	7y1
...............cgggguuucgCacguagcagagcagcuc......................................................................	4	1	7y1
...............cgggguuucguaAguagcagagcagcuc......................................................................	1	1	7y1
...............cgggguuucguaUguagcagagcagcuc......................................................................	1	1	7y1
...............cgggguuuAguacguagcagagcagcuc......................................................................	5	1	7y1
...............cggggAuucguacguagcagagcagcucccuc..................................................................	1	1	7y1
................gggguuAcguacguagcaga.............................................................................	2	1	7y1
................gggguuucguaAguagcaga.............................................................................	1	1	7y1
................gggguuucgAacguagcagag............................................................................	1	1	7y1
................gggguuuAguacguagcagag............................................................................	1	1	7y1
................gggguuuAguacguagcagagc...........................................................................	2	1	7y1
................gggguuucUuacguagcagagc...........................................................................	1	1	7y1
................gggguuAcguacguagcagagc...........................................................................	3	1	7y1
................gggUuuucguacguagcagagc...........................................................................	3	1	7y1
................ggggguAucguacguagcagagc..........................................................................	5	1	7y1
................gggguuucgAacguagcagagca..........................................................................	1	1	7y1
................gggguuucguaAguagcagagca..........................................................................	1	1	7y1
................gggguuuAguacguagcagagca..........................................................................	1	1	7y1
................gggguAucguacguagcagagca..........................................................................	2	1	7y1
................gggUuuucguacguagcagagca..........................................................................	1	1	7y1
................gggguuAcguacguagcagagca..........................................................................	2	1	7y1
................gggguuAcguacguagcagagcagc........................................................................	3	1	7y1
................gggguuucgAacguagcagagcagc........................................................................	1	1	7y1
................gggguuucguUcguagcagagcagc........................................................................	2	1	7y1
................gggguuucguacgCagcagagcagc........................................................................	1	1	7y1
................ggggGuucguacguagcagagcagc........................................................................	1	1	7y1
................gggguAucguacguagcagagcagc........................................................................	3	1	7y1
................gggguGucguacguagcagagcagc........................................................................	1	1	7y1
................gggguuCcguacguagcagagcagc........................................................................	1	1	7y1
................gggguuuAguacguagcagagcagc........................................................................	3	1	7y1
................gggguuucguacgAagcagagcagc........................................................................	1	1	7y1
................gggguuucguacguGgcagagcagc........................................................................	1	1	7y1
................gggguuucCuacguagcagagcagcu.......................................................................	1	1	7y1
................gggguuucguacgCagcagagcagcu.......................................................................	2	1	7y1
................gggguuucguacUuagcagagcagcu.......................................................................	1	1	7y1
................ggggAuucguacguagcagagcagcu.......................................................................	2	1	7y1
................gggguuucguGcguagcagagcagcu.......................................................................	1	1	7y1
................gggCuuucguacguagcagagcagcu.......................................................................	1	1	7y1
................ggggGuucguacguagcagagcagcu.......................................................................	1	1	7y1
................gggguuucguaAguagcagagcagcu.......................................................................	1	1	7y1
................gggguAucguacguagcagagcagcu.......................................................................	6	1	7y1
................gggguuAcguacguagcagagcagcu.......................................................................	1	1	7y1
................gggguuuAguacguagcagagcagcu.......................................................................	1	1	7y1
................gggguuucguacAuagcagagcagcuc......................................................................	2	1	7y1
```

Star **Mature**

gaccugcuucugggucgggguuucguacguagcagagcagcucccucgcugcgaucuauugaaagucagcccucgacacaaggguuuguccgcgcgcgcgcgcgcgcgugcgu			
................gggguuucAuacguagcagagcagcuc..	1	1	7y1
................gggguAucguacguagcagagcagcuc..	9	1	7y1
................gggguuucguacUuagcagagcagcuc..	3	1	7y1
................gggguCucguacguagcagagcagcuc..	2	1	7y1
................gggguuGcguacguagcagagcagcuc..	1	1	7y1
................gggguuucguacguGgcagagcagcuc..	1	1	7y1
................gggguuucguaAguagcagagcagcuc..	3	1	7y1
................gggUuuucguacguagcagagcagcuc..	1	1	7y1
................gggguuuAguacguagcagagcagcuc..	8	1	7y1
................gggguuucCuacguagcagagcagcuc..	1	1	7y1
................gggguuucguacguUgcagagcagcuc..	1	1	7y1
................gggguuucguGcguagcagagcagcuc..	1	1	7y1
................ggggAuucguacguagcagagcagcuc..	1	1	7y1
................gggguuAcguacguagcagagcagcuc..	4	1	7y1
................gggguuucgAacguagcagagcagcuc..	2	1	7y1
.................ggguuucguaAguagcaga...	1	1	7y1
.................ggguuAcguacguagcaga...	1	1	7y1
.................ggguuucguacgCagcaga...	1	1	7y1
.................ggguuuAguacguagcaga...	1	1	7y1
.................ggguuucAuacguagcagagc...	1	1	7y1
.................ggguAucguacguagcagagc...	2	1	7y1
.................gggGuucguacguagcagagc...	5	1	7y1
.................ggguuAcguacguagcagagc...	1	1	7y1
.................ggguuucguaAguagcagagc...	1	1	7y1
.................ggguuuAguacguagcagagc...	5	1	7y1
.................gggAuucguacguagcagagc...	1	1	7y1
.................ggguuucgAacguagcagagc...	2	1	7y1
.................ggguuucguaUguagcagagc...	1	1	7y1
.................ggguuucgGacguagcagagca..	1	1	7y1
.................ggguuucUuacguagcagagca..	1	1	7y1
.................gggGuucguacguagcagagca..	1	1	7y1
.................ggCuuucguacguagcagagca..	269	1	7y1
.................ggguuucgAacguagcagagca..	1	1	7y1
.................ggguAucguacguagcagagca..	1	1	7y1
.................ggguuucguaAguagcagagca..	1	1	7y1
.................ggguCucguacguagcagagca..	1	1	7y1
.................ggguuuGguacguagcagagca..	1	1	7y1
.................ggguuucguacguGgcagagca..	1	1	7y1
.................ggguuCcguacguagcagagca..	1	1	7y1
.................ggguuuAguacguagcagagca..	3	1	7y1
.................ggguuAcguacguagcagagca..	1	1	7y1
.................ggguGucguacguagcagagca..	1	1	7y1
.................ggguuucguacguaCcagagca..	1	1	7y1
.................ggguuucguacguaUcagagca..	1	1	7y1
.................ggguuucguaAguagcagagcagc..	1	1	7y1
.................ggguuucgAacguagcagagcagc..	1	1	7y1
.................ggguuuAguacguagcagagcagc..	2	1	7y1
.................gggGuucguacguagcagagcagc..	1	1	7y1
.................ggguuucguacCuagcagagcagcu...	1	1	7y1
.................ggguAucguacguagcagagcagcu...	2	1	7y1
.................ggguuuAguacguagcagagcagcu...	6	1	7y1
.................ggCuuucguacguagcagagcagcu...	1	1	7y1
.................gggGuucguacguagcagagcagcu...	2	1	7y1
.................ggguuucguacguaUcagagcagcu...	1	1	7y1
.................ggguuucCuacguagcagagcagcu...	1	1	7y1
.................ggguuucgAacguagcagagcagcu...	1	1	7y1
.................ggguuAcguacguagcagagcagcu...	5	1	7y1
.................ggguuucguaUguagcagagcagcu...	1	1	7y1
.................ggguuucguaAguagcagagcagcu...	1	1	7y1
.................gggGuucguacguagcagagcagcuc..	2	1	7y1
.................ggguuucguacUuagcagagcagcuc..	1	1	7y1
.................ggguuAcguacguagcagagcagcuc..	6	1	7y1
.................ggguAucguacguagcagagcagcuc..	5	1	7y1
.................ggguuucguacgAagcagagcagcuc..	2	1	7y1
.................ggguuCcguacguagcagagcagcuc..	1	1	7y1
.................ggguuuAguacguagcagagcagcuc..	9	1	7y1
.................ggguuucguaUguagcagagcagcuc..	1	1	7y1
.................gggCuucguacguagcagagcagcuc..	1	1	7y1
.................ggguuucguacCuagcagagcagcuc..	1	1	7y1
.................ggguuucguaAguagcagagcagcuc..	2	1	7y1

```
  Star                                                                   Mature
gaccugcuucugggucgggguuucguacguagcagagcagcucccucgcugcgaucuauugaaagucagcccucgacacaaggguuuguccgcgcgcgcgcgcgcgcgcgugcgu
.................ggguuucUuacguagcagagcagcuc........................................................................    1     1     7y1
.................ggguuucAuacguagcagagcagcuc........................................................................    2     1     7y1
.................ggguuucguacguCgcagagcagcuc........................................................................    1     1     7y1
.................ggguuucgAacguagcagagcagcuc........................................................................    1     1     7y1
.................ggguuucguacgAagcagagcagcucc.......................................................................    1     1     7y1
.................gggGuucguacguagcagagcagcucccu.....................................................................    1     1     7y1
.................ggguuucgAacguagcagagcagcucccu.....................................................................    1     1     7y1
.................ggguuucguacguGgcagagcagcucccu.....................................................................    1     1     7y1
.................ggguuAcguacguagcagagcagcucccu.....................................................................    1     1     7y1
.................ggguuuAguacguagcagagcagcucccu.....................................................................    1     1     7y1
..................gguuAcguacguagcagagc.............................................................................    1     1     7y1
..................gguuucCuacguagcagagc.............................................................................    4     1     7y1
..................gguuucguacguaCcagagc.............................................................................    3     1     7y1
..................gguuucAuacguagcagagc.............................................................................    3     1     7y1
..................gguuucguacUuagcagagc.............................................................................    1     1     7y1
..................gUuuucguacguagcagagc.............................................................................    3     1     7y1
..................gguuucguacguagAagagc.............................................................................    5     1     7y1
..................gguuucguUcguagcagagc.............................................................................    1     1     7y1
..................gguuucguaGguagcagagc.............................................................................    1     1     7y1
..................gguuucgAacguagcagagc.............................................................................    9     1     7y1
..................gguuucguacguGgcagagc.............................................................................    1     1     7y1
..................gguuucguacCuagcagagc.............................................................................    2     1     7y1
..................ggGuucguacguagcagagc.............................................................................    1     1     7y1
..................ggAuucguacguagcagagc.............................................................................    4     1     7y1
..................gguGucguacguagcagagc.............................................................................    1     1     7y1
..................gguuucguacguaUcagagc.............................................................................    2     1     7y1
..................gguuucguaAguagcagagc.............................................................................    16    1     7y1
..................gguCucguacguagcagagc.............................................................................    1     1     7y1
..................gguuuAguacguagcagagc.............................................................................    11    1     7y1
..................gguuucguacguagGagagc.............................................................................    1     1     7y1
..................gguuucguacguUgcagagc.............................................................................    1     1     7y1
..................gguAucguacguagcagagc.............................................................................    3     1     7y1
..................gguuucguacgGagcagagc.............................................................................    1     1     7y1
..................gguuuGguacguagcagagc.............................................................................    2     1     7y1
..................gCuuucguacguagcagagc.............................................................................    2     1     7y1
..................gguuucguacgAagcagagc.............................................................................    5     1     7y1
..................gguuAcguacguagcagagca............................................................................    1     1     7y1
..................gguuucguacCuagcagagca............................................................................    1     1     7y1
..................gCuuucguacguagcagagca............................................................................    1     1     7y1
..................gguuucguaUguagcagagca............................................................................    1     1     7y1
..................gguuuAguacguagcagagca............................................................................    3     1     7y1
..................gguuucgGacguagcagagca............................................................................    1     1     7y1
..................gguuucguacguaAcagagca............................................................................    1     1     7y1
..................gguuucgAacguagcagagca............................................................................    1     1     7y1
..................gguCucguacguagcagagca............................................................................    1     1     7y1
..................gguuucguacguaCcagagca............................................................................    2     1     7y1
..................gguuucCuacguagcagagca............................................................................    2     1     7y1
..................gguuucguacguagUagagca............................................................................    1     1     7y1
..................gguuucguaAguagcagagca............................................................................    3     1     7y1
..................ggAuucguacguagcagagca............................................................................    1     1     7y1
..................gUuuucguacguagcagagcag...........................................................................    1     1     7y1
..................gguAucguacguagcagagcag...........................................................................    1     1     7y1
..................gguuucCuacguagcagagcag...........................................................................    1     1     7y1
..................gguuucgAacguagcagagcag...........................................................................    2     1     7y1
..................gguuuAguacguagcagagcag...........................................................................    1     1     7y1
..................ggAuucguacguagcagagcag...........................................................................    1     1     7y1
..................gguuucguacguagGagagcagc..........................................................................    3     1     7y1
..................gCuuucguacguagcagagcagc..........................................................................    3     1     7y1
..................gguuucguacCuagcagagcagc..........................................................................    1     1     7y1
..................gguuucguaAguagcagagcagc..........................................................................    5     1     7y1
..................gguuucguacgAagcagagcagc..........................................................................    6     1     7y1
..................gguuucguacguagUagagcagc..........................................................................    2     1     7y1
..................ggAuucguacguagcagagcagc..........................................................................    7     1     7y1
..................gguuucgAacguagcagagcagc..........................................................................    8     1     7y1
..................gguuucUuacguagcagagcagc..........................................................................    4     1     7y1
..................gguuucguaGguagcagagcagc..........................................................................    2     1     7y1
..................gguuucguacguaCcagagcagc..........................................................................    2     1     7y1
..................gAuuucguacguagcagagcagc..........................................................................    1     1     7y1
..................gguuucguacUuagcagagcagc..........................................................................    3     1     7y1
..................gguuucguacgCagcagagcagc..........................................................................    1     1     7y1
```

Star / Mature			
gaccugcuucugggucgggguuucguacguagcagagcagcucccucgcugcgaucuauugaaagucagcccucgacacaaggguuuguccgcgcgcgcgcgcgcgcgcgugcgu			
..................gguuucguacguaUcagagcagc..	4	1	7y1
..................gguuucguacguagAagagcagc..	4	1	7y1
..................ggGuucguacguagcagagcagc..	1	1	7y1
..................gguuucCuacguagcagagcagc..	3	1	7y1
..................gguuucguaUguagcagagcagc..	1	1	7y1
..................gUuuucguacguagcagagcagc..	6	1	7y1
..................gguAucguacguagcagagcagc..	2	1	7y1
..................gguuuAguacguagcagagcagc..	7	1	7y1
..................gguCucguacguagcagagcagc..	2	1	7y1
..................gguuAcguacguagcagagcagcu...	5	1	7y1
..................gUuuucguacguagcagagcagcu...	4	1	7y1
..................gguuucguacgCagcagagcagcu...	1	1	7y1
..................gguuuUguacguagcagagcagcu...	1	1	7y1
..................gguuucguUcguagcagagcagcu...	2	1	7y1
..................gguAucguacguagcagagcagcu...	5	1	7y1
..................gCuuucguacguagcagagcagcu...	2	1	7y1
..................ggAuucguacguagcagagcagcu...	6	1	7y1
..................gguuucguaGguagcagagcagcu...	1	1	7y1
..................gguuucguacAuagcagagcagcu...	4	1	7y1
..................gguuucguacUuagcagagcagcu...	1	1	7y1
..................gguuucgAacguagcagagcagcu...	10	1	7y1
..................gguuGcguacguagcagagcagcu...	1	1	7y1
..................gguuucUuacguagcagagcagcu...	4	1	7y1
..................gguuucguacguagGagagcagcu...	2	1	7y1
..................gguuucguacguaUcagagcagcu...	4	1	7y1
..................gguuucguacguGgcagagcagcu...	3	1	7y1
..................gguuucguGcguagcagagcagcu...	1	1	7y1
..................gguuucAuacguagcagagcagcu...	2	1	7y1
..................gguuucCuacguagcagagcagcu...	2	1	7y1
..................gguuuGguacguagcagagcagcu...	2	1	7y1
..................gguuucguaAguagcagagcagcu...	20	1	7y1
..................gguuucguacgAagcagagcagcu...	6	1	7y1
..................ggCuucguacguagcagagcagcu...	1	1	7y1
..................gguuCcguacguagcagagcagcu...	3	1	7y1
..................gguuucguacguagAagagcagcu...	10	1	7y1
..................gguuuAguacguagcagagcagcu...	20	1	7y1
..................gguuucguacCuagcagagcagcu...	7	1	7y1
..................gguuucguacguaAcagagcagcu...	1	1	7y1
..................gguuucguaUguagcagagcagcuc..	1	1	7y1
..................gguuucAuacguagcagagcagcuc..	1	1	7y1
..................gguuuAguacguagcagagcagcuc..	3	1	7y1
..................ggGuucguacguagcagagcagcuc..	2	1	7y1
..................gguuucguacgAagcagagcagcuc..	4	1	7y1
..................gguuucUuacguagcagagcagcuc..	1	1	7y1
..................gguuucguacCuagcagagcagcuc..	2	1	7y1
..................gCuuucguacguagcagagcagcuc..	3	1	7y1
..................gguuucCuacguagcagagcagcuc..	1	1	7y1
..................gguuucguaAguagcagagcagcuc..	6	1	7y1
..................gguuucguacguagUagagcagcuc..	1	1	7y1
..................gguuucguacguaUcagagcagcuc..	1	1	7y1
..................gguuucguacAuagcagagcagcuc..	2	1	7y1
..................gguuucgCacguagcagagcagcuc..	1	1	7y1
..................gguuucUuacguagcagagcagcucc...	1	1	7y1
..................gguuucguacguaUcagagcagcucc...	1	1	7y1
..................gUuuucguacguagcagagcagcucc...	1	1	7y1
..................gguAucguacguagcagagcagcucc...	1	1	7y1
..................ggAuucguacguagcagagcagcucc...	4	1	7y1
..................gguuucguacgAagcagagcagcucc...	2	1	7y1
..................gguuGcguacguagcagagcagcucc...	1	1	7y1
..................gguuucguCcguagcagagcagcucc...	1	1	7y1
..................gguuucgCacguagcagagcagcucc...	1	1	7y1
..................gguuAcguacguagcagagcagcucc...	2	1	7y1
..................gguuucguacguagAagagcagcucc...	1	1	7y1
..................gguuucguacAuagcagagcagcucc...	1	1	7y1
..................gguuucguNcguagcagagcagcucc...	1	1	7y1
..................gguuucCuacguagcagagcagcucc...	1	1	7y1
..................gguuucguaAguagcagagcagcucc...	2	1	7y1
..................gguuucguacCuagcagagcagcucc...	2	1	7y1
..................ggGuucguacguagcagagcagcucc...	2	1	7y1
..................gguuuAguacguagcagagcagcucc...	5	1	7y1

Star **Mature**

gaccugcuucugggucggggguuucguacguagcagagcagcucccucgcugcgaucuauugaaagucagcccucgacacaaggguuuguccgcgcgcgcgcgcgcgcgugcgu			
..................gCuuucguacguagcagagcagcucc...	1	1	7y1
..................gguuucguUcguagcagagcagcucc...	1	1	7y1
..................gguAucguacguagcagagcagcuccc..	2	1	7y1
..................gguuucUuacguagcagagcagcuccc..	4	1	7y1
..................gguuucguacguagGagagcagcuccc..	1	1	7y1
..................gguuucguacguaCcagagcagcuccc..	3	1	7y1
..................gguuucguacguaUcagagcagcuccc..	1	1	7y1
..................gguuucguacgAagcagagcagcuccc..	2	1	7y1
..................gguuuUguacguagcagagcagcuccc..	1	1	7y1
..................gguuucCuacguagcagagcagcuccc..	3	1	7y1
..................gguuucguacUuagcagagcagcuccc..	1	1	7y1
..................ggGuucguacguagcagagcagcuccc..	3	1	7y1
..................gguuucgGacguagcagagcagcuccc..	2	1	7y1
..................gguuucguacguagAagagcagcuccc..	5	1	7y1
..................gguuucguaGguagcagagcagcuccc..	2	1	7y1
..................ggCuucguacguagcagagcagcuccc..	1	1	7y1
..................gCuuucguacguagcagagcagcuccc..	1	1	7y1
..................gguuucguacgGagcagagcagcuccc..	1	1	7y1
..................gguuuAguacguagcagagcagcuccc..	15	1	7y1
..................ggAuucguacguagcagagcagcuccc..	3	1	7y1
..................gguuucguaAguagcagagcagcuccc..	11	1	7y1
..................gguuucguacCuagcagagcagcuccc..	4	1	7y1
..................gguuucgAacguagcagagcagcuccc..	2	1	7y1
..................gguuucguacAuagcagagcagcuccc..	1	1	7y1
..................gUuuucguacguagcagagcagcuccc..	2	1	7y1
..................gguuGcguacguagcagagcagcuccc..	1	1	7y1
..................gguuucguacgCagcagagcagcuccc..	1	1	7y1
..................gguuucguacguCgcagagcagcucccu...	1	1	7y1
..................gguuucguacgAagcagagcagcucccu...	5	1	7y1
..................gguuucguacCuagcagagcagcucccu...	2	1	7y1
..................gguuucguacguGgcagagcagcucccu...	2	1	7y1
..................gguuucguacguagAagagcagcucccu...	5	1	7y1
..................gguuucguacUuagcagagcagcucccu...	1	1	7y1
..................gguuuAguacguagcagagcagcucccu...	9	1	7y1
..................ggAuucguacguagcagagcagcucccu...	4	1	7y1
..................gCuuucguacguagcagagcagcucccu...	2	1	7y1
..................ggCuucguacguagcagagcagcucccu...	1	1	7y1
..................gguuucguacguagGagagcagcucccu...	1	1	7y1
..................gguuucguacguaUcagagcagcucccu...	3	1	7y1
..................ggGuucguacguagcagagcagcucccu...	2	1	7y1
..................gguuucguGcguagcagagcagcucccu...	1	1	7y1
..................gguuucguaAguagcagagcagcucccu...	12	1	7y1
..................gguuAcguacguagcagagcagcucccu...	2	1	7y1
..................gAuuucguacguagcagagcagcucccu...	1	1	7y1
..................gguuucgAacguagcagagcagcucccu...	7	1	7y1
..................gguuucgCacguagcagagcagcucccu...	1	1	7y1
..................gguuuUguacguagcagagcagcucccu...	1	1	7y1
..................gguuucguacguaAcagagcagcucccu...	2	1	7y1
..................gguuucguCcguagcagagcagcucccu...	2	1	7y1
..................gguuucUuacguagcagagcagcucccu...	5	1	7y1
..................gguuucguaUguagcagagcagcucccu...	1	1	7y1
..................gguuucAuacguagcagagcagcucccu...	1	1	7y1
..................gUuuucguacguagcagagcagcucccu...	1	1	7y1
..................gguAucguacguagcagagcagcucccu...	3	1	7y1
..................gguuucUuacguagcagagcagcucccuc..	1	1	7y1
..................gguuAcguacguagcagagcagcucccuc..	1	1	7y1
..................gguuucguGcguagcagagcagcucccuc..	1	1	7y1
..................gguuuGguacguagcagagcagcucccuc..	1	1	7y1
..................gguuuAguacguagcagagcagcucccuc..	1	1	7y1
..................gguuucguacAuagcagagcagcucccuc..	2	1	7y1
..................gguuucguaAguagcagagcagcucccuc..	1	1	7y1
..................ggCuucguacguagcagagcagcucccuc..	1	1	7y1
..................gguuucgAacguagcagagcagcucccuc..	2	1	7y1
..................ggAuucguacguagcagagcagcucccuc..	2	1	7y1
..................gguuucguacUuagcagagcagcucccuc..	2	1	7y1
..................gguuucguacguagAagagcagcucccuc..	1	1	7y1
..................gguuucCuacguagcagagcagcucccuc..	2	1	7y1
..................gguuucguacgAagcagagcagcucccuc..	1	1	7y1
..................gguuucguacguaCcagagcagcucccuc..	1	1	7y1
..................gguuucguacguaUcagagcagcucccuc..	1	1	7y1

Star **Mature**

gaccugcuucugggucggggguuucguacguagcagagcagcucccucgcugcgaucuauugaaagucagcccucgacacaaggguuuguccgcgcgcgcgcgcgcgcgugcgu			
..................gguuucguaGguagcagagcagcucccuc..	1	1	7y1
..................gguuucgGacguagcagagcagcucccuc..	1	1	7y1
..................gguuucguacguGgcagagcagcucccuc..	1	1	7y1
..................gCuuucguacguagcagagcagcucccuc..	1	1	7y1
..................gguuucguacguaCcagagcagcucccucg...	1	1	7y1
..................gguuucguacgAagcagagcagcucccucg...	3	1	7y1
..................gguuAcguacguagcagagcagcucccucg...	1	1	7y1
..................ggAuucguacguagcagagcagcucccucg...	3	1	7y1
..................gguuucguUcguagcagagcagcucccucg...	1	1	7y1
..................gguuucgGacguagcagagcagcucccucg...	1	1	7y1
..................gguuucguacguagUagagcagcucccucg...	1	1	7y1
..................gguuuAguacguagcagagcagcucccucg...	3	1	7y1
..................gCuuucguacguagcagagcagcucccucg...	1	1	7y1
..................gguuucguacguagAagagcagcucccucg...	2	1	7y1
..................gguuucCuacguagcagagcagcucccucg...	2	1	7y1
..................gguuucguacguagGagagcagcucccucg...	1	1	7y1
..................gguuucguaAguagcagagcagcucccucg...	1	1	7y1
..................gguuucguaUguagcagagcagcucccucg...	2	1	7y1
..................gguAucguacguagcagagcagcucccucg...	2	1	7y1
..................gguuucguacguaAcagagcagcucccucgc..	1	1	7y1
..................gguCucguacguagcagagcagcucccucgc..	1	1	7y1
..................gCuuucguacguagcagagcagcucccucgc..	1	1	7y1
..................gguuucAuacguagcagagcagcucccucgc..	1	1	7y1
..................ggGuucguacguagcagagcagcucccucgc..	2	1	7y1
..................gguuucguacUuagcagagcagcucccucgc..	1	1	7y1
..................gguuucguacguagUagagcagcucccucgc..	1	1	7y1
..................gguuucguacgAagcagagcagcucccucgc..	2	1	7y1
..................gguuuAguacguagcagagcagcucccucgc..	1	1	7y1
..................gguuucguacguGgcagagcagcucccucgc..	1	1	7y1
..................gguGucguacguagcagagcagcucccucgc..	1	1	7y1
..................ggAuucguacguagcagagcagcucccucgc..	2	1	7y1
..................gguAucguacguagcagagcagcucccucgc..	1	1	7y1
..................gguuucCuacguagcagagcagcucccucgc..	4	1	7y1
..................gUuuucguacguagcagagcagcucccucgc..	2	1	7y1
..................gguuucgAacguagcagagcagcucccucgc..	4	1	7y1
..................gguuCcguacguagcagagcagcucccucgc..	1	1	7y1
..................gguuucguaAguagcagagcagcucccucgc..	5	1	7y1
..................gguuucguacguagAagagcagcucccucgcu...	1	1	7y1
..................gguuucUuacguagcagagcagcucccucgcu...	1	1	7y1
..................ggAuucguacguagcagagcagcucccucgcu...	1	1	7y1
..................gguuucguacCuagcagagcagcucccucgcu...	1	1	7y1
..................gguuucguGcguagcagagcagcucccucgcu...	1	1	7y1
..................gguuucguacguaUcagagcagcucccucgcu...	1	1	7y1
..................gguuucguacguaCcagagcagcucccucgcu...	1	1	7y1
..................gUuuucguacguagcagagcagcucccucgcu...	1	1	7y1
..................gguuucAuacguagcagagcagcucccucgcu...	1	1	7y1
...................guuGcguacguagcagagc...	1	1	7y1
...................guuucguaAguagcagagc...	5	1	7y1
...................Nuuucguacguagcagagc...	1	1	7y1
...................Uuuucguacguagcagagc...	1	1	7y1
...................guuuAguacguagcagagc...	1	1	7y1
...................guuucCuacguagcagagc...	1	1	7y1
...................Cuuucguacguagcagagc...	3	1	7y1
...................guuucgAacguagcagagc...	3	1	7y1
...................guuucUuacguagcagagc...	1	1	7y1
...................guuAcguacguagcagagca..	1	1	7y1
...................guuuAguacguagcagagca..	1	1	7y1
...................guuucguacguagAagagca..	2	1	7y1
...................gAuucguacguagcagagca..	1	1	7y1
...................guAucguacguagcagagca..	1	1	7y1
...................guuCcguacguagcagagca..	1	1	7y1
...................guuucCuacguagcagagca..	2	1	7y1
...................guuucguacCuagcagagca..	2	1	7y1
...................Uuuucguacguagcagagca..	1	1	7y1
...................guuucgAacguagcagagca..	5	1	7y1
...................Cuuucguacguagcagagca..	2	1	7y1
...................guuucguacgAagcagagcagc..	1	1	7y1
...................Auuucguacguagcagagcagc..	1	1	7y1
...................guuucguaAguagcagagcagc..	2	1	7y1
...................gAuucguacguagcagagcagc..	5	1	7y1

Star **Mature**

gaccugcuucugggucgggguuucguacguagcagagcagcucccucgcugcgaucuauugaaagucagcccucgacacaaggguuuguccgcgcgcgcgcgcgcgcgugcgu

...................guAucguacguagcagagcagc..	3	1	7y1
...................Uuuucguacguagcagagcagc..	286	1	7y1
...................guuucgAacguagcagagcagc..	2	1	7y1
...................guuucguUcguagcagagcagc..	1	1	7y1
...................guAucguacguagcagagcagcu...	4	1	7y1
...................guuucguacgAagcagagcagcu...	3	1	7y1
...................Uuuucguacguagcagagcagcu...	3	1	7y1
...................gGuucguacguagcagagcagcu...	1	1	7y1
...................Cuuucguacguagcagagcagcu...	2	1	7y1
...................guuucgAacguagcagagcagcu...	3	1	7y1
...................guuucguacUuagcagagcagcu...	1	1	7y1
...................Nuuucguacguagcagagcagcu...	3	1	7y1
...................gAuucguacguagcagagcagcu...	6	1	7y1
...................guuucguacguGgcagagcagcu...	1	1	7y1
...................guuAcguacguagcagagcagcu...	2	1	7y1
...................guuucguacguagAagagcagcu...	1	1	7y1
...................guuucUuacguagcagagcagcu...	1	1	7y1
...................guuuAguacguagcagagcagcu...	1	1	7y1
...................gAuucguacguagcagagcagcuc..	2	1	7y1
...................guuuUguacguagcagagcagcuc..	1	1	7y1
...................guAucguacguagcagagcagcuc..	3	1	7y1
...................guCucguacguagcagagcagcuc..	1	1	7y1
...................gGuucguacguagcagagcagcuc..	1	1	7y1
...................Uuuucguacguagcagagcagcucc...	1	1	7y1
...................guuucguacgAagcagagcagcucc...	1	1	7y1
...................guAucguacguagcagagcagcucc...	1	1	7y1
...................guuucguacguaAcagagcagcucc...	1	1	7y1
...................guuucguacguaUcagagcagcucc...	179	1	7y1
...................guuucguGcguagcagagcagcucc...	1	1	7y1
...................gAuucguacguagcagagcagcuccc..	4	1	7y1
...................guuucguacguagAagagcagcuccc..	5	1	7y1
...................guuucguacguCgcagagcagcuccc..	1	1	7y1
...................guAucguacguagcagagcagcuccc..	2	1	7y1
...................Cuuucguacguagcagagcagcuccc..	2	1	7y1
...................guuucguacCuagcagagcagcuccc..	3	1	7y1
...................guuucguaAguagcagagcagcuccc..	2	1	7y1
...................guuAcguacguagcagagcagcuccc..	1	1	7y1
...................guuucgAacguagcagagcagcuccc..	2	1	7y1
...................guuucCuacguagcagagcagcuccc..	2	1	7y1
...................gCuucguacguagcagagcagcuccc..	2	1	7y1
...................guuucguacguaAcagagcagcuccc..	2	1	7y1
...................guuucguacgGagcagagcagcuccc..	1	1	7y1
...................guuucguacgAagcagagcagcuccc..	1	1	7y1
...................guuuAguacguagcagagcagcuccc..	3	1	7y1
...................guuuUguacguagcagagcagcuccc..	1	1	7y1
...................guuucguacguaCcagagcagcuccc..	3	1	7y1
...................guuucguacguaUcagagcagcuccc..	1	1	7y1
...................guuuAguacguagcagagcagcucccu...	1	1	7y1
...................guuAcguacguagcagagcagcucccu...	2	1	7y1
...................guuucCuacguagcagagcagcucccu...	1	1	7y1
...................guuucguacgGagcagagcagcucccu...	1	1	7y1
...................guuucguacAuagcagagcagcucccu...	2	1	7y1
...................gGuucguacguagcagagcagcucccu...	295	1	7y1
...................guuucguacgCagcagagcagcucccu...	1	1	7y1
...................guAucguacguagcagagcagcucccu...	2	1	7y1
...................guuucguacguagcGgagcagcucccu...	2	1	7y1
...................Cuuucguacguagcagagcagcucccu...	3	1	7y1
...................guuucguacCuagcagagcagcucccu...	1	1	7y1
...................guuucgAacguagcagagcagcucccu...	7	1	7y1
...................gAuucguacguagcagagcagcucccu...	7	1	7y1
...................guuucguacguagAagagcagcucccu...	3	1	7y1
...................Uuuucguacguagcagagcagcucccu...	2	1	7y1
...................guuucguacguagcGgagcagcucccuc..	1	1	7y1
...................guuucguGcguagcagagcagcucccuc..	2	1	7y1
...................gGuucguacguagcagagcagcucccuc..	1	1	7y1
...................guuucguacgAagcagagcagcucccuc..	1	1	7y1
...................guuucguaAguagcagagcagcucccuc..	1	1	7y1
...................guuucguacUuagcagagcagcucccuc..	3	1	7y1
...................guuucgAacguagcagagcagcucccuc..	2	1	7y1
...................guuucguacAuagcagagcagcucccuc..	1	1	7y1

```
Star                                                                   Mature
gaccugcuucugggucgggguuucguacguagcagagcagcucccucgcugcgaucuauugaaagucagcccucgacacaaggguuuguccgcgcgcgcgcgcgcgcgugcgu
...................guuCcguacguagcagagcagcucccuc..................................................................    1      1    7y1
...................guAucguacguagcagagcagcucccuc..................................................................    1      1    7y1
...................Cuuucguacguagcagagcagcucccuc..................................................................    1      1    7y1
...................guuucguacguagAagagcagcucccuc..................................................................    2      1    7y1
...................guuucguacguaUcagagcagcucccuc..................................................................    1      1    7y1
...................guuucguacCuagcagagcagcucccuc..................................................................    1      1    7y1
...................Uuuucguacguagcagagcagcucccuc..................................................................    3      1    7y1
...................guuucguacguaUcagagcagcucccucg.................................................................    1      1    7y1
...................guuucgAacguagcagagcagcucccucg.................................................................    4      1    7y1
...................gAuucguacguagcagagcagcucccucg.................................................................    1      1    7y1
...................guAucguacguagcagagcagcucccucg.................................................................    1      1    7y1
...................guuucguacAuagcagagcagcucccucg.................................................................    1      1    7y1
...................guuucguacguagcGgagcagcucccucg.................................................................    1      1    7y1
...................guuucguacgCagcagagcagcucccucgc................................................................    1      1    7y1
...................guuucCuacguagcagagcagcucccucgc................................................................    1      1    7y1
...................guuucguacCuagcagagcagcucccucgc................................................................    1      1    7y1
...................guuucguacUuagcagagcagcucccucgc................................................................    2      1    7y1
...................Uuuucguacguagcagagcagcucccucgc................................................................    1      1    7y1
...................guuucguaAguagcagagcagcucccucgc................................................................    3      1    7y1
...................gAuucguacguagcagagcagcucccucgc................................................................    1      1    7y1
...................guuucgGacguagcagagcagcucccucgc................................................................    1      1    7y1
...................gGuucguacguagcagagcagcucccucgc................................................................    1      1    7y1
...................guuucguacguaUcagagcagcucccucgc................................................................    2      1    7y1
...................Nuuucguacguagcagagcagcucccucgc................................................................    1      1    7y1
...................guAucguacguagcagagcagcucccucgc................................................................    2      1    7y1
...................guuucguacgAagcagagcagcucccucgc................................................................    1      1    7y1
...................guuucgAacguagcagagcagcucccucgc................................................................    4      1    7y1
...................guuAcguacguagcagagcagcucccucgc................................................................    2      1    7y1
...................Cuuucguacguagcagagcagcucccucgc................................................................    1      1    7y1
...................guuucUuacguagcagagcagcucccucgc................................................................    1      1    7y1
...................guuucguacguagAagagcagcucccucgc................................................................    1      1    7y1
...................guuucguacguagAagagcagcucccucgcu...............................................................    1      1    7y1
...................guuucguaGguagcagagcagcucccucgcu...............................................................    1      1    7y1
...................guuucUuacguagcagagcagcucccucgcu...............................................................    1      1    7y1
...................gAuucguacguagcagagcagcucccucgcu...............................................................    2      1    7y1
...................Cuuucguacguagcagagcagcucccucgcu...............................................................    2      1    7y1
...................gAuucguacguagcagagcagcucccucgcug..............................................................    1      1    7y1
...................guuucguacguagAagagcagcucccucgcug..............................................................    1      1    7y1
...................guuucguacUuagcagagcagcucccucgcug..............................................................    1      1    7y1
....................uucguacgCagcagagc............................................................................    1      1    7y1
....................uucguacguagcagaUc............................................................................    5      1    7y1
....................Auucguacguagcagagc...........................................................................    52     1    7y1
....................uuAcguacguagcagagc...........................................................................    2      1    7y1
....................uuucCuacguagcagagc...........................................................................    192    1    7y1
....................uucguacguaCcagagc............................................................................    1      1    7y1
....................uucguacgAagcagagc............................................................................    11     1    7y1
....................uucguacguagcGgagc............................................................................    2      1    7y1
....................uucgGacguagcagagc............................................................................    1      1    7y1
....................Guucguacguagcagagc...........................................................................    9      1    7y1
....................uucguacguaUcagagc............................................................................    2      1    7y1
....................Nuucguacguagcagagc...........................................................................    3      1    7y1
....................uucguacguagcagagc............................................................................    4344   0    7y1
....................uucAuacguagcagagc............................................................................    1      1    7y1
....................uucguacguagcUgagc............................................................................    2      1    7y1
....................uucUuacguagcagagc............................................................................    1      1    7y1
....................uucgAacguagcagagc............................................................................    8      1    7y1
....................Cuucguacguagcagagc...........................................................................    2      1    7y1
....................uucguacCuagcagagc............................................................................    4      1    7y1
....................uucguacguagcaUagc............................................................................    1      1    7y1
....................uAucguacguagcagagc...........................................................................    9      1    7y1
....................uuAguacguagcagagc............................................................................    5      1    7y1
....................uucguacguagcagaCc............................................................................    2      1    7y1
....................uucguacUuagcagagc............................................................................    1      1    7y1
....................uucguacguGgcagagc............................................................................    2      1    7y1
....................uucguaUguagcagagc............................................................................    1      1    7y1
....................uucguacguagcagagA............................................................................    7      1    7y1
....................uucguacguagAagagc............................................................................    4      1    7y1
....................uuGguacguagcagagc............................................................................    3      1    7y1
....................uucguaAguagcagagc............................................................................    8      1    7y1
....................uucguaGguagcagagca...........................................................................    1      1    7y1
```

Star Mature

gaccugcuucugggucgggguuucguacguagcagagcagcucccucgcugcgaucuauugaaagucagcccucgacacaaggguuuguccgcgcgcgcgcgcgcgcgugcgu			
....................uuucguacguagcagagAa..	4	1	7y1
....................uuucguacguagAagagca..	3	1	7y1
....................uCucguacguagcagagca..	1	1	7y1
....................uuucguacguagcagCgca..	3	1	7y1
....................uuuAguacguagcagagca..	2	1	7y1
....................uuucguaAguagcagagca..	2	1	7y1
....................Nuucguacguagcagagca..	3	1	7y1
....................Auucguacguagcagagca..	24	1	7y1
....................uuucguacguagcagaUca..	1	1	7y1
....................uuucguacguaCcagagca..	1	1	7y1
....................uuucguacguagcagagca..	1926	0	7y1
....................uuucguGcguagcagagca..	1	1	7y1
....................uuucguacguUgcagagca..	2	1	7y1
....................uuucguacguagcagaCca..	3	1	7y1
....................uuucguacguagcagagGa..	1	1	7y1
....................uuucguacguagUagagca..	1	1	7y1
....................uuucgAacguagcagagca..	2	1	7y1
....................uuucguacguaUcagagca..	2	1	7y1
....................uuucguacCuagcagagca..	2	1	7y1
....................uuucguacgGagcagagca..	1	1	7y1
....................uuucguacguagcGgagca..	1	1	7y1
....................uuucCuacguagcagagca..	1	1	7y1
....................uuucguCcguagcagagca..	1	1	7y1
....................uuucguacUuagcagagca..	1	1	7y1
....................uuucguacgCagcagagca..	1	1	7y1
....................uuucguacguagcUgagca..	1	1	7y1
....................Guucguacguagcagagca..	4	1	7y1
....................uuucgCacguagcagagca..	1	1	7y1
....................uAucguacguagcagagca..	5	1	7y1
....................uuucguacguagcaUagca..	1	1	7y1
....................uuucguacguagcaUagcag...	1	1	7y1
....................uuucguacguagcagagcaU...	2	1	7y1
....................uuCcguacguagcagagcag...	1	1	7y1
....................uuucguacgAagcagagcag...	1	1	7y1
....................uuAcguacguagcagagcag...	1	1	7y1
....................uAucguacguagcagagcag...	4	1	7y1
....................uuucguacguagcagagAag...	1	1	7y1
....................uuucguacguagcGgagcag...	1	1	7y1
....................uuucguacguagcagaUcag...	1	1	7y1
....................uuucguacguagcagagcaC...	2	1	7y1
....................Auucguacguagcagagcag...	11	1	7y1
....................uuucgCacguagcagagcag...	1	1	7y1
....................uuucguacguagcagagcag...	641	0	7y1
....................uuucguacguagcagagGagc..	2	1	7y1
....................uuucgAacguagcagagcagc..	3	1	7y1
....................uuucgGacguagcagagcagc..	3	1	7y1
....................uuucguGcguagcagagcagc..	1	1	7y1
....................uuucguaAguagcagagcagc..	5	1	7y1
....................uuucguaGguagcagagcagc..	1	1	7y1
....................uuucguacguagcagagcaAc..	1	1	7y1
....................uuucguaUguagcagagcagc..	1	1	7y1
....................uuucguacguagAagagcagc..	2	1	7y1
....................uuucguCcguagcagagcagc..	1	1	7y1
....................uuucguacguagcagagcagc..	2127	0	7y1
....................Cuucguacguagcagagcagc..	237	1	7y1
....................uuucgCacguagcagagcagc..	1	1	7y1
....................Auucguacguagcagagcagc..	18	1	7y1
....................uuucguacgAagcagagcagc..	2	1	7y1
....................uuucguacguagUagagcagc..	1	1	7y1
....................uuucguacguagcGgagcagc..	1	1	7y1
....................uuucguacUuagcagagcagc..	1	1	7y1
....................uAucguacguagcagagcagc..	5	1	7y1
....................Nuucguacguagcagagcagc..	1	1	7y1
....................uuucguacguagcagagcaCc..	1	1	7y1
....................uuucguacguagcagagcaUc..	1	1	7y1
....................uuucguacguagGagagcagc..	1	1	7y1
....................uuucguacguagcagagAagc..	3	1	7y1
....................uuucguacguagcaUagcagc..	1	1	7y1
....................uuucguacguagcagagcagA..	1	1	7y1
....................uuuGguacguagcagagcagc..	2	1	7y1

Star Mature

```
gaccugcuucugggucggggguuucguacguagcagagcagcucccucgcugcgaucuauugaaagucagcccucgacacaaggguuuguccgcgcgcgcgcgcgcgcgugcgu
....................Guucguacguagcagagcagc........................................................................ 3 1 7y1
....................uuucguacguagcagCgcagc........................................................................ 5 1 7y1
....................uuucguacguagcUgagcagcu....................................................................... 1 1 7y1
....................uuucguacUuagcagagcagcu....................................................................... 1 1 7y1
....................uuucguacguagcagGgcagcu....................................................................... 1 1 7y1
....................uuucguacguagcaUagcagcu....................................................................... 272 1 7y1
....................uuucguacguagcagagcagAu....................................................................... 1 1 7y1
....................Nuucguacguagcagagcagcu....................................................................... 2 1 7y1
....................uuucguacguagcagagcagcC....................................................................... 1 1 7y1
....................uuucguacguagcagagGagcu....................................................................... 2 1 7y1
....................uAucguacguagcagagcagcu....................................................................... 10 1 7y1
....................uuucguacguaAcagagcagcu....................................................................... 1 1 7y1
....................uuAcguacguagcagagcagcu....................................................................... 3 1 7y1
....................uuucAuacguagcagagcagcu....................................................................... 2 1 7y1
....................uuucguaUguagcagagcagcu....................................................................... 1 1 7y1
....................uuucguacguagcagagAagcu....................................................................... 9 1 7y1
....................uuuUguacguagcagagcagcu....................................................................... 2 1 7y1
....................uuucguaAguagcagagcagcu....................................................................... 8 1 7y1
....................uuucguacAuagcagagcagcu....................................................................... 1 1 7y1
....................uuucguacguaCcagagcagcu....................................................................... 1 1 7y1
....................uuuAguacguagcagagcagcu....................................................................... 7 1 7y1
....................Cuucguacguagcagagcagcu....................................................................... 3 1 7y1
....................uuucguacguagAagagcagcu....................................................................... 2 1 7y1
....................uuucguacguagcagagcagcG....................................................................... 22 1 7y1
....................Auucguacguagcagagcagcu....................................................................... 37 1 7y1
....................uuucguacguagcagaCcagcu....................................................................... 1 1 7y1
....................uuucgAacguagcagagcagcu....................................................................... 4 1 7y1
....................uuucguacguagcagagcagcA....................................................................... 3 1 7y1
....................uuucguacCuagcagagcagcu....................................................................... 4 1 7y1
....................uuucguacguagcagCgcagcu....................................................................... 4 1 7y1
....................uuucguacguagcagagcagcu....................................................................... 4088 0 7y1
....................uuucguacguagcagagcaUcu....................................................................... 4 1 7y1
....................uuucguacguagcagagcCgcu....................................................................... 3 1 7y1
....................uuucguacguagUagagcagcu....................................................................... 1 1 7y1
....................uuucgCacguagcagagcagcu....................................................................... 1 1 7y1
....................uuucguacguagcagagcaCcu....................................................................... 1 1 7y1
....................uuucguGcguagcagagcagcu....................................................................... 1 1 7y1
....................uuucguacgAagcagagcagcu....................................................................... 11 1 7y1
....................Guucguacguagcagagcagcu....................................................................... 7 1 7y1
....................uuucguacguagcagUgcagcu....................................................................... 2 1 7y1
....................uuucguacguagcaCagcagcu....................................................................... 2 1 7y1
....................uuucCuacguagcagagcagcu....................................................................... 2 1 7y1
....................uuucguacguagcagagcagGuc...................................................................... 1 1 7y1
....................uuucguacguagcagagcagcuc...................................................................... 7935 0 7y1
....................uuucAuacguagcagagcagcuc...................................................................... 1 1 7y1
....................uuucguacguagcagagAagcuc...................................................................... 10 1 7y1
....................uuucguacguagcagagcUgcuc...................................................................... 1 1 7y1
....................uuucUuacguagcagagcagcuc...................................................................... 6 1 7y1
....................uuucguacguagcUgagcagcuc...................................................................... 1 1 7y1
....................uuucguacguagcagUgcagcuc...................................................................... 1 1 7y1
....................uuucgAacguagcagagcagcuc...................................................................... 16 1 7y1
....................uuucguacguagcagagcCgcuc...................................................................... 3 1 7y1
....................uuucguacguagcagagcagcuA...................................................................... 8 1 7y1
....................uuucguacguagcagaUcagcuc...................................................................... 3 1 7y1
....................uuucguacguagcagaCcagcuc...................................................................... 4 1 7y1
....................Auucguacguagcagagcagcuc...................................................................... 89 1 7y1
....................uuucguacguagcagagcGgcuc...................................................................... 2 1 7y1
....................uuucguacguagcagagcaCcuc...................................................................... 4 1 7y1
....................uuuUguacguagcagagcagcuc...................................................................... 1 1 7y1
....................uuucguacguaCcagagcagcuc...................................................................... 2 1 7y1
....................uuucguacguagcagagGagcuc...................................................................... 1 1 7y1
....................uuucguacgCagcagagcagcuc...................................................................... 1 1 7y1
....................uuucguacguagcagGgcagcuc...................................................................... 1 1 7y1
....................uuucguacguagAagagcagcuc...................................................................... 9 1 7y1
....................uuGcguacguagcagagcagcuc...................................................................... 1 1 7y1
....................uuucguacguagcaCagcagcuc...................................................................... 1 1 7y1
....................uuuAguacguagcagagcagcuc...................................................................... 12 1 7y1
....................uuucCuacguagcagagcagcuc...................................................................... 2 1 7y1
....................uuucguacCuagcagagcagcuc...................................................................... 4 1 7y1
....................uuucguacguagcagagcagcGc...................................................................... 1 1 7y1
```

```
   Star                                                                Mature
gaccugcuucugggucgggguuucguacguagcagagcagcucccucgcugcgaucuauugaaagucagcccucgacacaaggguuuguccgcgcgcgcgcgcgcgcgugcgu
....................uuucguacguagcagagcagcuG......................................................................  1     1  7y1
....................uuucguacguagcCgagcagcuc......................................................................  1     1  7y1
....................uuucguacAuagcagagcagcuc......................................................................  1     1  7y1
....................Guucguacguagcagagcagcuc......................................................................  13    1  7y1
....................uuucguaAguagcagagcagcuc......................................................................  9     1  7y1
....................uuucguacguagcagagcagcAc......................................................................  2     1  7y1
....................uuCcguacguagcagagcagcuc......................................................................  1     1  7y1
....................uuucguacguagcagagcaUcuc......................................................................  7     1  7y1
....................uuucguacguGgcagagcagcuc......................................................................  2     1  7y1
....................uuucguacguagcGgagcagcuc......................................................................  1     1  7y1
....................uuucguacguagcagagcagcuU......................................................................  1     1  7y1
....................uuucgCacguagcagagcagcuc......................................................................  1     1  7y1
....................uuucguacgAagcagagcagcuc......................................................................  11    1  7y1
....................uuucguacUuagcagagcagcuc......................................................................  3     1  7y1
....................uuucguaUguagcagagcagcuc......................................................................  1     1  7y1
....................uuucguacguagcagCgcagcuc......................................................................  7     1  7y1
....................uuuGguacguagcagagcagcuc......................................................................  3     1  7y1
....................uuucguCcguagcagagcagcuc......................................................................  2     1  7y1
....................uuucguacguagcagagcagAuc......................................................................  5     1  7y1
....................Nuucguacguagcagagcagcuc......................................................................  4     1  7y1
....................uAucguacguagcagagcagcuc......................................................................  11    1  7y1
....................uuucguacguaUcagagcagcuc......................................................................  1     1  7y1
....................Cuucguacguagcagagcagcuc......................................................................  175   1  7y1
....................uuucguGcguagcagagcagcuc......................................................................  1     1  7y1
....................uuucguacguagUagagcagcuc......................................................................  2     1  7y1
....................uuucguacguagcaUagcagcuc......................................................................  2     1  7y1
....................uuAcguacguagcagagcagcuc......................................................................  3     1  7y1
....................uCucguacguagcagagcagcuc......................................................................  1     1  7y1
....................uuucguacguagcagagcagcucG.....................................................................  1     1  7y1
....................uuucguacguagcagGgcagcucc.....................................................................  4     1  7y1
....................uuucguacgGagcagagcagcucc.....................................................................  1     1  7y1
....................uuucguacguagcagagcagcuUc.....................................................................  1     1  7y1
....................uuucguaAguagcagagcagcucc.....................................................................  7     1  7y1
....................uuucguacguagcagagcagcAcc.....................................................................  1     1  7y1
....................uuucguacguagcaUagcagcucc.....................................................................  1     1  7y1
....................uuucguacCuagcagagcagcucc.....................................................................  4     1  7y1
....................uuucguacguagcaCagcagcucc.....................................................................  1     1  7y1
....................uuucguacguaUcagagcagcucc.....................................................................  3     1  7y1
....................uuucguacguagcagagcCgcucc.....................................................................  1     1  7y1
....................uuucguacguagcagagcaUcucc.....................................................................  3     1  7y1
....................uuucCuacguagcagagcagcucc.....................................................................  1     1  7y1
....................uuucguacguagcagagcagcucc.....................................................................  5981  0  7y1
....................uuucguacguagcagaCcagcucc.....................................................................  3     1  7y1
....................uuucguacguagcagagcagcucU.....................................................................  2     1  7y1
....................Auucguacguagcagagcagcucc.....................................................................  67    1  7y1
....................uuucguacguagcagagcGgcucc.....................................................................  1     1  7y1
....................uuucguacguagcagagcagcCcc.....................................................................  2     1  7y1
....................Nuucguacguagcagagcagcucc.....................................................................  3     1  7y1
....................uuuGguacguagcagagcagcucc.....................................................................  2     1  7y1
....................uuucguacUuagcagagcagcucc.....................................................................  2     1  7y1
....................uuucguacguagcagCgcagcucc.....................................................................  5     1  7y1
....................uuucguacguagcagagcagcucA.....................................................................  2     1  7y1
....................uuucguacgAagcagagcagcucc.....................................................................  7     1  7y1
....................uuucUuacguagcagagcagcucc.....................................................................  2     1  7y1
....................uuucgCacguagcagagcagcucc.....................................................................  2     1  7y1
....................uuAcguacguagcagagcagcucc.....................................................................  1     1  7y1
....................uuCcguacguagcagagcagcucc.....................................................................  2     1  7y1
....................uuGcguacguagcagagcagcucc.....................................................................  1     1  7y1
....................Guucguacguagcagagcagcucc.....................................................................  14    1  7y1
....................uuucgAacguagcagagcagcucc.....................................................................  11    1  7y1
....................uuucguacguagcagagcagAucc.....................................................................  4     1  7y1
....................uuucguacguagcagagcaCcucc.....................................................................  3     1  7y1
....................uuucguacguUgcagagcagcucc.....................................................................  1     1  7y1
....................uAucguacguagcagagcagcucc.....................................................................  9     1  7y1
....................uuucguacgCagcagagcagcucc.....................................................................  1     1  7y1
....................uuucguacguagAagagcagcucc.....................................................................  9     1  7y1
....................uuucguacguagcGgagcagcucc.....................................................................  1     1  7y1
....................uuucguacguagcagagAagcucc.....................................................................  7     1  7y1
....................uuucguaGguagcagagcagcucc.....................................................................  2     1  7y1
....................uuucguaUguagcagagcagcucc.....................................................................  1     1  7y1
```

```
Star                                                          Mature
gaccugcuucugggucgggguuucguacguagcagagcagcucccucgcugcgaucuauugaaagucagcccucgacacaaggguuuguccgcgcgcgcgcgcgcgcgugcgu
....................uuucguacguagcagagcagcuAc.....................................................................   15     1   7y1
....................Cuucguacguagcagagcagcucc.....................................................................   1      1   7y1
....................uuucguUcguagcagagcagcucc.....................................................................   2      1   7y1
....................uuuAguacguagcagagcagcucc.....................................................................   6      1   7y1
....................uNucguacguagcagagcagcucc.....................................................................   1      1   7y1
....................uuucguacguagcagaUcagcucc.....................................................................   1      1   7y1
....................uuuUguacguagcagagcagcucc.....................................................................   1      1   7y1
....................uuucguacguagcagagcaAcucc.....................................................................   1      1   7y1
....................uuucguacguagcagUgcagcucc.....................................................................   3      1   7y1
....................uuucguacguagcagagcagAuccc....................................................................   1      1   7y1
....................uuucUuacguagcagagcagcuccc....................................................................   1      1   7y1
....................uuucguacguagcagagcagGuccc....................................................................   1      1   7y1
....................uuucguacCuagcagagcagcuccc....................................................................   1      1   7y1
....................uuucguacguagcagUgcagcuccc....................................................................   2      1   7y1
....................uuucguacguagcagagcagcuccA....................................................................   4      1   7y1
....................uuucguacguagUagagcagcuccc....................................................................   277    1   7y1
....................uuuGguacguagcagagcagcuccc....................................................................   3      1   7y1
....................uuucguacguagcagaCcagcuccc....................................................................   1      1   7y1
....................uuucguacguagcagagcagcAccc....................................................................   1      1   7y1
....................uuucguacguagcGgagcagcuccc....................................................................   1      1   7y1
....................uuucguaAguagcagagcagcuccc....................................................................   3      1   7y1
....................uuucAuacguagcagagcagcuccc....................................................................   1      1   7y1
....................Auucguacguagcagagcagcuccc....................................................................   17     1   7y1
....................uuucguacguagcagCgcagcuccc....................................................................   4      1   7y1
....................uuucguacguagcagagcaUcuccc....................................................................   3      1   7y1
....................uuucCuacguagcagagcagcuccc....................................................................   1      1   7y1
....................uAucguacguagcagagcagcuccc....................................................................   3      1   7y1
....................uuucguacguagcagagcagcuccG....................................................................   1      1   7y1
....................uuucguacUuagcagagcagcuccc....................................................................   1      1   7y1
....................uuucgCacguagcagagcagcuccc....................................................................   1      1   7y1
....................uuucguacguagcagagcagcuAcc....................................................................   11     1   7y1
....................uuucguacguagcagagAagcuccc....................................................................   2      1   7y1
....................uuucguacguagcagagcaCcuccc....................................................................   2      1   7y1
....................Guucguacguagcagagcagcuccc....................................................................   2      1   7y1
....................uuucguaGguagcagagcagcuccc....................................................................   1      1   7y1
....................uuucguacguagAagagcagcuccc....................................................................   2      1   7y1
....................uuucguacguagcagagcagcuccc....................................................................   2072   0   7y1
....................uuucguacgAagcagagcagcuccc....................................................................   3      1   7y1
....................uuucguacguaUcagagcagcuccc....................................................................   1      1   7y1
....................uuuAguacguagcagagcagcuccc....................................................................   2      1   7y1
....................uuAcguacguagcagagcagcuccc....................................................................   2      1   7y1
....................uuucguacguagGagagcagcuccc....................................................................   1      1   7y1
....................uuucgAacguagcagagcagcuccc....................................................................   4      1   7y1
....................uuucguacguaCcagagcagcucccu...................................................................   1      1   7y1
....................uuucguacguagcagagcCgcucccu...................................................................   1      1   7y1
....................uuucguacguagcagagcagAucccu...................................................................   2      1   7y1
....................uuucUuacguagcagagcagcucccu...................................................................   3      1   7y1
....................uuucguacguagcagaUcagcucccu...................................................................   1      1   7y1
....................uAucguacguagcagagcagcucccu...................................................................   9      1   7y1
....................uuucguacguagcagagcagcucccC...................................................................   152    1   7y1
....................uuucguacguagcagagcGgcucccu...................................................................   1      1   7y1
....................uCucguacguagcagagcagcucccu...................................................................   1      1   7y1
....................uuucguacguagcUgagcagcucccu...................................................................   1      1   7y1
....................uuucguacguagcGgagcagcucccu...................................................................   1      1   7y1
....................uuucguacguagcagagAagcucccu...................................................................   6      1   7y1
....................uGucguacguagcagagcagcucccu...................................................................   1      1   7y1
....................uuucguacUuagcagagcagcucccu...................................................................   7      1   7y1
....................uuucguacguaAcagagcagcucccu...................................................................   2      1   7y1
....................uuucCuacguagcagagcagcucccu...................................................................   1      1   7y1
....................uuucguacCuagcagagcagcucccu...................................................................   1      1   7y1
....................uuucguacguGgcagagcagcucccu...................................................................   2      1   7y1
....................Auucguacguagcagagcagcucccu...................................................................   42     1   7y1
....................uuucguacguaUcagagcagcucccu...................................................................   1      1   7y1
....................uuuAguacguagcagagcagcucccu...................................................................   6      1   7y1
....................uuucguacguagcagGgcagcucccu...................................................................   2      1   7y1
....................uuucguacguagcaUagcagcucccu...................................................................   1      1   7y1
....................uuucguacguagcagagcagcucccu...................................................................   4551   0   7y1
....................uuucguacgAagcagagcagcucccu...................................................................   7      1   7y1
....................uuAcguacguagcagagcagcucccu...................................................................   4      1   7y1
....................uuucguacguagcagagcagcAcccu...................................................................   1      1   7y1
```

Star **Mature**

```
gaccugcuucugggucgggguuucguacguagcagagcagcucccucgcugcgaucuauugaaagucagcccucgacacaaggguuuguccgcgcgcgcgcgcgcgcgugcgu
...................uuucguGcguagcagagcagcucccu....................................................................   1      1   7y1
...................uuucguacguagcagagcagcCcccu....................................................................   1      1   7y1
...................uuucguacAuagcagagcagcucccu....................................................................   3      1   7y1
...................Guucguacguagcagagcagcucccu....................................................................   14     1   7y1
...................uuucguacguagcaAagcagcucccu....................................................................   1      1   7y1
...................uuucguaUguagcagagcagcucccu....................................................................   2      1   7y1
...................uuucguacguagcagagcagcuccAu....................................................................   4      1   7y1
...................uuucguacguagcagagcagcuccccG....................................................................   17     1   7y1
...................uuucgAacguagcagagcagcucccu....................................................................   9      1   7y1
...................uuGcguacguagcagagcagcucccu....................................................................   1      1   7y1
...................uuucguacguagcagagcagcucccA....................................................................   8      1   7y1
...................uuucguaAguagcagagcagcucccu....................................................................   4      1   7y1
...................uuucguacguagUagagcagcucccu....................................................................   225    1   7y1
...................uuucguacguagcagagcagcucAcu....................................................................   3      1   7y1
...................uuucguacguagAagagcagcucccu....................................................................   2      1   7y1
...................Nuucguacguagcagagcagcucccu....................................................................   1      1   7y1
...................uuucguacguagcagCgcagcucccu....................................................................   10     1   7y1
...................Cuucguacguagcagagcagcucccu....................................................................   2      1   7y1
...................uuucguacguagcagaCcagcucccu....................................................................   2      1   7y1
...................uuucguacguagcagagcagcuAccu....................................................................   10     1   7y1
...................uuAcguacguagcagagcagcucccuc...................................................................   3      1   7y1
...................uuucCuacguagcagagcagcucccuc...................................................................   2      1   7y1
...................uuucguacguagcagagAagcucccuc...................................................................   6      1   7y1
...................uuucguacguagcagagcaCcucccuc...................................................................   1      1   7y1
...................Cuucguacguagcagagcagcucccuc...................................................................   1      1   7y1
...................uuucguacguagcagCgcagcucccuc...................................................................   10     1   7y1
...................uuucguacguagcagagcagcuAccuc...................................................................   7      1   7y1
...................uuucguacguagcagagcCgcucccuc...................................................................   1      1   7y1
...................Nuucguacguagcagagcagcucccuc...................................................................   6      1   7y1
...................uuucguacguagcagagcagcCcccuc...................................................................   1      1   7y1
...................uuucguacguaAcagagcagcucccuc...................................................................   1      1   7y1
...................uuucguacguagcagUgcagcucccuc...................................................................   2      1   7y1
...................uuucguacguagcagagcaUcucccuc...................................................................   2      1   7y1
...................uuucguacguagcagagcagcuccAuc...................................................................   5      1   7y1
...................uuucguacguagcaUagcagcucccuc...................................................................   2      1   7y1
...................uuucgCacguagcagagcagcucccuc...................................................................   1      1   7y1
...................uuucguacguagcagagcGgcucccuc...................................................................   2      1   7y1
...................uuucguaUguagcagagcagcucccuc...................................................................   1      1   7y1
...................uuucguacguagcagagcagcuGccuc...................................................................   1      1   7y1
...................uuucguacguagcagagcagcucAcuc...................................................................   6      1   7y1
...................uuucguacguaUcagagcagcucccuc...................................................................   1      1   7y1
...................uuucguacguagcagagcagAucccuc...................................................................   4      1   7y1
...................uuucguacguagcagagcagcucccuc...................................................................   4379   0   7y1
...................uuucUuacguagcagagcagcucccuc...................................................................   2      1   7y1
...................uuucguacguagcagagcUgcucccuc...................................................................   1      1   7y1
...................uuucguacguagAagagcagcucccuc...................................................................   5      1   7y1
...................uAucguacguagcagagcagcucccuc...................................................................   9      1   7y1
...................uuucguacguagUagagcagcucccuc...................................................................   1      1   7y1
...................Guucguacguagcagagcagcucccuc...................................................................   12     1   7y1
...................Auucguacguagcagagcagcucccuc...................................................................   39     1   7y1
...................uuucgAacguagcagagcagcucccuc...................................................................   9      1   7y1
...................uuucguacgAagcagagcagcucccuc...................................................................   7      1   7y1
...................uuuGguacguagcagagcagcucccuc...................................................................   1      1   7y1
...................uuucguaAguagcagagcagcucccuc...................................................................   3      1   7y1
...................uuucguacguagcagagcagcucccuA...................................................................   3      1   7y1
...................uuucguacguagcagagGagcucccuc...................................................................   1      1   7y1
...................uuucguacguagcagagcagcucUcuc...................................................................   1      1   7y1
...................uuucguacguagcaCagcagcucccuc...................................................................   2      1   7y1
...................uuucguacguagcagagcagcucccAc...................................................................   4      1   7y1
...................uuucguacguagcagagcagcAcccuc...................................................................   2      1   7y1
...................uuuAguacguagcagagcagcucccuc...................................................................   11     1   7y1
...................uuucguacguagcagagcagcuUccuc...................................................................   1      1   7y1
...................uuucguacguagcagagcagcucGcuc...................................................................   2      1   7y1
...................uuucguacCuagcagagcagcucccuc...................................................................   1      1   7y1
...................uuucguacUuagcagagcagcucccuc...................................................................   2      1   7y1
...................uuAcguacguagcagagcagcucccucgc.................................................................   1      1   7y1
...................uuucguacguagcagCgcagcucccucgc.................................................................   1      1   7y1
...................uuuAguacguagcagagcagcucccucgc.................................................................   1      1   7y1
...................uuucguacguagUagagcagcucccucgc.................................................................   1      1   7y1
...................uuucguacguagcagagcagcucAcucgc.................................................................   1      1   7y1
```

```
  Star                                                                  Mature
gaccugcuucugggucgggguuucguacguagcagagcagcucccucgcugcgaucuauugaaagucagcccucgacacaaggguuuguccgcgcgcgcgcgcgcgcgugcgu
....................uuucguacguagcagaUcagcucccucgc................................................................   1      1      7y1
....................uuucguacguagcagagAagcucccucgc................................................................   1      1      7y1
....................uuuGguacguagcagagcagcucccucgc................................................................   1      1      7y1
....................uuucguacguagcagagcagcucccAcgc................................................................   1      1      7y1
....................uuucguacguagcagagcUgcucccucgc................................................................   1      1      7y1
....................Guucguacguagcagagcagcucccucgc................................................................   2      1      7y1
....................uuucguacguagcagagcaCcucccucgc................................................................   1      1      7y1
....................uuucguacguagcagagcagcucccucgc................................................................   614    0      7y1
....................uuucguacgAagcagagcagcucccucgc................................................................   1      1      7y1
....................uAucguacguagcagagcagcucccucgc................................................................   1      1      7y1
....................uuucgAacguagcagagcagcucccucgc................................................................   1      1      7y1
....................uuucguacCuagcagagcagcucccucgc................................................................   1      1      7y1
....................uuucguacguagcagagcagcuAccucgc................................................................   1      1      7y1
....................Auucguacguagcagagcagcucccucgc................................................................   4      1      7y1
....................uuuAguacguagcagagcagcucccucgcu...............................................................   4      1      7y1
....................uuucguacguagGagagcagcucccucgcu...............................................................   1      1      7y1
....................uuucguacguagcagagcagcucccuGgcu...............................................................   1      1      7y1
....................uuucguacguagcagaCcagcucccucgcu...............................................................   1      1      7y1
....................Auucguacguagcagagcagcucccucgcu...............................................................   20     1      7y1
....................uuucguacguagcagagcagcCcccucgcu...............................................................   1      1      7y1
....................uuucguacguagAagagcagcucccucgcu...............................................................   3      1      7y1
....................uuucguacCuagcagagcagcucccucgcu...............................................................   1      1      7y1
....................uuucguacguagcCgagcagcucccucgcu...............................................................   1      1      7y1
....................uuucguacguagcagagcCgcucccucgcu...............................................................   2      1      7y1
....................uuucguacguaCcagagcagcucccucgcu...............................................................   1      1      7y1
....................uuucguacguagcagagcagcucUcucgcu...............................................................   2      1      7y1
....................uuucguUcguagcagagcagcucccucgcu...............................................................   1      1      7y1
....................uuucguacguagcagagAagcucccucgcu...............................................................   2      1      7y1
....................uuucguacguagcagagcGgcucccucgcu...............................................................   1      1      7y1
....................uuucguacguagcagagcaUcucccucgcu...............................................................   1      1      7y1
....................uuucguacguagcagUgcagcucccucgcu...............................................................   1      1      7y1
....................uuucguacguagcagagcUgcucccucgcu...............................................................   1      1      7y1
....................uuucguacguagcagaUcagcucccucgcu...............................................................   1      1      7y1
....................uuucguacguagcagagcagcuAccucgcu...............................................................   7      1      7y1
....................uuucguacguagcagagcagcucccuAgcu...............................................................   3      1      7y1
....................uuucguacguagcagGgcagcucccucgcu...............................................................   2      1      7y1
....................uuucguacguGgcagagcagcucccucgcu...............................................................   1      1      7y1
....................uuucgAacguagcagagcagcucccucgcu...............................................................   4      1      7y1
....................uuucguacguagcagagcagcucccucgcu...............................................................   2277   0      7y1
....................uuucguaAguagcagagcagcucccucgcu...............................................................   2      1      7y1
....................Guucguacguagcagagcagcucccucgcu...............................................................   178    1      7y1
....................uAucguacguagcagagcagcucccucgcu...............................................................   1      1      7y1
....................uuucguacguagcagagcagcucGcucgcu...............................................................   1      1      7y1
....................uuGcguacguagcagagcagcucccucgcu...............................................................   1      1      7y1
....................uuAcguacguagcagagcagcucccucgcu...............................................................   1      1      7y1
....................uuucguacguagcagCgcagcucccucgcu...............................................................   2      1      7y1
....................uuucguacgCagcagagcagcucccucgcu...............................................................   1      1      7y1
....................uuucguacgAagcagagcagcucccucgcu...............................................................   6      1      7y1
....................uuucguacguagcaCagcagcucccucgcu...............................................................   2      1      7y1
....................uuucguacUuagcagagcagcucccucgcu...............................................................   1      1      7y1
....................uuucguacguagcagagcagUucccucgcu...............................................................   1      1      7y1
....................uuucguacguagcagagcagcucccucAcu...............................................................   2      1      7y1
....................uuucAuacguagcagagcagcucccucgcu...............................................................   2      1      7y1
....................uuucguacguagcagagcagcucccucgAu...............................................................   3      1      7y1
....................uuucguacguagcagagcagcucccucgcG...............................................................   20     1      7y1
....................Nuucguacguagcagagcagcucccucgcu...............................................................   2      1      7y1
....................uuucguacguagcagagcagcucccAcgcu...............................................................   2      1      7y1
....................uuucguacguagcagagcagcucAcucgcu...............................................................   1      1      7y1
....................uuucguacCuagcagagcagcucccucgcug..............................................................   1      1      7y1
....................uuucAuacguagcagagcagcucccucgcug..............................................................   1      1      7y1
....................uuucguacguagcUgagcagcucccucgcug..............................................................   1      1      7y1
....................uuucguacguagcagagcGgcucccucgcug..............................................................   1      1      7y1
....................uuucguacguagcagagcagcucccucCcug..............................................................   1      1      7y1
....................uuucguacguagcagagcagcucccucgcGg..............................................................   9      1      7y1
....................Auucguacguagcagagcagcucccucgcug..............................................................   6      1      7y1
....................uuucguacguagcagagcagcucccAcgcug..............................................................   1      1      7y1
....................uuucguUcguagcagagcagcucccucgcug..............................................................   1      1      7y1
....................uuucguacguagcagagcagcucccucgcug..............................................................   855    0      7y1
....................uuucguacguagcagagcagcucccucUcug..............................................................   1      1      7y1
....................uuucguacgAagcagagcagcucccucgcug..............................................................   1      1      7y1
```

Star **Mature**

gaccugcuucugggucgggguuucguacguagcagagcagcucccucgcugcgaucuauugaaagucagcccucgacacaaggguuuguccgcgcgcgcgcgcgcgcgugcgu			
....................uuucguacguagcagagcaAcucccucgcug..	1	1	7y1
....................uuucUuacguagcagagcagcucccucgcug..	1	1	7y1
....................uuucguacguagcagagcagcuAccucgcug..	2	1	7y1
....................uuucguacguagcagagAagcucccucgcug..	2	1	7y1
....................uAucguacguagcagagcagcucccucgcug..	2	1	7y1
....................uuucguCcguagcagagcagcucccucgcug..	1	1	7y1
....................uuucguacguagcagagcagcucccucgGug..	1	1	7y1
....................uuucgAacguagcagagcagcucccucgcug..	2	1	7y1
....................uuucguacguagAagagcagcucccucgcug..	1	1	7y1
....................uuucguaAguagcagagcagcucccucgcug..	1	1	7y1
....................uuucguacguagcagagcagAucccucgcugc...	1	1	7y1
....................uAucguacguagcagagcagcucccucgcugc...	1	1	7y1
....................uuucguacguagcagagcagcAcccucgcugc...	1	1	7y1
....................uuucguacCuagcagagcagcucccucgcugc...	1	1	7y1
....................Guucguacguagcagagcagcucccucgcugc...	1	1	7y1
....................uuucguacguagcagagcagcucccucgcugc...	499	0	7y1
....................uuucguacguagcagagcagcucccucgcuCc...	1	1	7y1
....................uuucguacgAagcagagcagcucccucgcugc...	1	1	7y1
....................Auucguacguagcagagcagcucccucgcugc...	5	1	7y1
....................uuuAguacguagcagagcagcucccucgcugc...	1	1	7y1
....................uuucguacguagcagagcagcuAccucgcugc...	1	1	7y1
....................Nuucguacguagcagagcagcucccucgcugc...	2	1	7y1
....................uuucguacguagcagagcagcucccucgAugc...	1	1	7y1
.....................uucguacguagcaUagca..	1	1	7y1
.....................uucguacguagcaCagca..	1	1	7y1
.....................uucguacguagcagagAa..	3	1	7y1
.....................uucguacguaCcagagca..	1	1	7y1
.....................uucguacguaAcagagca..	1	1	7y1
.....................uucguacguagcagGgca..	1	1	7y1
.....................uucguacguagcagagGa..	1	1	7y1
.....................uucguacguagAagagca..	1	1	7y1
.....................uucguacgAagcagagca..	3	1	7y1
.....................uucAuacguagcagagca..	1	1	7y1
.....................uAcguacguagcagagca..	4	1	7y1
.....................Nucguacguagcagagca..	1	1	7y1
.....................uucguaAguagcagagca..	8	1	7y1
.....................Gucguacguagcagagca..	2	1	7y1
.....................uuAguacguagcagagca..	4	1	7y1
.....................uucguacguagcagaAca..	1	1	7y1
.....................uucguacguagcagagcG..	1	1	7y1
.....................uNcguacguagcagagca..	1	1	7y1
.....................Aucguacguagcagagca..	12	1	7y1
.....................uucguacCuagcagagca..	2	1	7y1
.....................uucguacguagcagagca..	1212	0	7y1
.....................Cucguacguagcagagca..	1	1	7y1
.....................uucguacguagcagagcC..	1	1	7y1
.....................uucguacguagcagaUca..	2	1	7y1
.....................uucguacAuagcagagcag...	1	1	7y1
.....................uucguacguagcaCagcag...	1	1	7y1
.....................Aucguacguagcagagcag...	16	1	7y1
.....................uAcguacguagcagagcag...	3	1	7y1
.....................Gucguacguagcagagcag...	2	1	7y1
.....................uucguacguagcagagcag...	1378	0	7y1
.....................uucguacguagAagagcag...	1	1	7y1
.....................uucguaAguagcagagcag...	4	1	7y1
.....................uucgCacguagcagagcag...	1	1	7y1
.....................uucguacgAagcagagcag...	2	1	7y1
.....................uucguacguagcagagAag...	1	1	7y1
.....................uucguacguGgcagagcag...	1	1	7y1
.....................uucguacguagcagagcaU...	2	1	7y1
.....................uucguacCuagcagagcag...	1	1	7y1
.....................uucguacguagcagagcUg...	1	1	7y1
.....................uucguacguaUcagagcag...	1	1	7y1
.....................uuAguacguagcagagcag...	4	1	7y1
.....................uucguUcguagcagagcagc..	2	1	7y1
.....................uucguacgAagcagagcagc..	1	1	7y1
.....................uucguacAuagcagagcagc..	1	1	7y1
.....................Nucguacguagcagagcagc..	1	1	7y1
.....................uucguacUuagcagagcagc..	2	1	7y1
.....................uucguaGguagcagagcagc..	2	1	7y1

Star Mature

gaccugcuucugggucggggguuucguacguagcagagcagcucccucgcugcgaucuauugaaagucagcccucgacacaaggguuuguccgcgcgcgcgcgcgcgcgugcgu			
.....................uAcguacguagcagagcagc..	2	1	7y1
.....................uucguacguagcaCagcagc..	1	1	7y1
.....................uucCuacguagcagagcagc..	1	1	7y1
.....................uucguacCuagcagagcagc..	1	1	7y1
.....................uucguacguagcagagcaUc..	3	1	7y1
.....................uucguacguagcagaUcagc..	1	1	7y1
.....................uucguacguagcagaCcagc..	1	1	7y1
.....................Aucguacguagcagagcagc..	33	1	7y1
.....................uucguacguGgcagagcagc..	2	1	7y1
.....................uucguacguagcagagcagA..	2	1	7y1
.....................uuUguacguagcagagcagc..	1	1	7y1
.....................uucguacguagcaUagcagc..	1	1	7y1
.....................uucgAacguagcagagcagc..	1	1	7y1
.....................uucguacguagcUgagcagc..	1	1	7y1
.....................uucguaAguagcagagcagc..	2	1	7y1
.....................uucguacguaAcagagcagc..	1	1	7y1
.....................uCcguacguagcagagcagc..	1	1	7y1
.....................uucguacgCagcagagcagc..	1	1	7y1
.....................uucguacguagcaAagcagc..	1	1	7y1
.....................uucguacguagcagagcagc..	1790	0	7y1
.....................uucguacguagcagGgcagc..	1	1	7y1
.....................uuAguacguagcagagcagc..	5	1	7y1
.....................uucguacguagcagagcGgcu...	5	1	7y1
.....................uucguacguagcagagAagcu...	9	1	7y1
.....................uucguacguagUagagcagcu...	185	1	7y1
.....................uucguacguagcagagcagcA...	4	1	7y1
.....................uucguacguagcagagcagAu...	4	1	7y1
.....................uucguacguagcGgagcagcu...	1	1	7y1
.....................Nucguacguagcagagcagcu...	2	1	7y1
.....................uucguGcguagcagagcagcu...	1	1	7y1
.....................uucguacguaUcagagcagcu...	3	1	7y1
.....................uucguacguagcUgagcagcu...	2	1	7y1
.....................uucguacguagcagagcUgcu...	1	1	7y1
.....................uucCuacguagcagagcagcu...	1	1	7y1
.....................uucguacgAagcagagcagcu...	8	1	7y1
.....................uucUuacguagcagagcagcu...	5	1	7y1
.....................uucguacguagcagagcaUcu...	3	1	7y1
.....................uucguacguagcagagcaAcu...	1	1	7y1
.....................uucguacguagcagaCcagcu...	5	1	7y1
.....................uuGguacguagcagagcagcu...	2	1	7y1
.....................uucguacguagcagagcagGu...	2	1	7y1
.....................uucguacguagcagGgcagcu...	1	1	7y1
.....................uucguacguagcagagcaCcu...	3	1	7y1
.....................uucgGacguagcagagcagcu...	1	1	7y1
.....................uucguacguagcagagcagcu...	7198	0	7y1
.....................Aucguacguagcagagcagcu...	80	1	7y1
.....................uucguacguagcagagcagcG...	40	1	7y1
.....................uucgCacguagcagagcagcu...	3	1	7y1
.....................uucguacguagAagagcagcu...	3	1	7y1
.....................uucguacguagcagCgcagcu...	7	1	7y1
.....................uucguacguGgcagagcagcu...	2	1	7y1
.....................uucgAacguagcagagcagcu...	9	1	7y1
.....................uucguacguagcagagGagcu...	4	1	7y1
.....................uucguacguagcagagcagcC...	1	1	7y1
.....................uucguacguUgcagagcagcu...	1	1	7y1
.....................uucguaUguagcagagcagcu...	3	1	7y1
.....................Gucguacguagcagagcagcu...	8	1	7y1
.....................uucguacCuagcagagcagcu...	4	1	7y1
.....................uucguacguagcaAagcagcu...	1	1	7y1
.....................uucguUcguagcagagcagcu...	1	1	7y1
.....................uuAguacguagcagagcagcu...	24	1	7y1
.....................uucguacguaCcagagcagcu...	3	1	7y1
.....................uAcguacguagcagagcagcu...	18	1	7y1
.....................uucguacguagcagagUagcu...	1	1	7y1
.....................uucguacguagcaUagcagcu...	5	1	7y1
.....................uucguaGguagcagagcagcu...	1	1	7y1
.....................uucguacguagcaCagcagcu...	1	1	7y1
.....................uucguacAuagcagagcagcu...	1	1	7y1
.....................uucguaAguagcagagcagcu...	22	1	7y1
.....................uucguacguagcagagcCgcu...	1	1	7y1

```
  Star                                                              Mature
gaccugcuucugggucggggguuucguacguagcagagcagcucccucgcugcgaucuauugaaagucagcccucgacacaaggguuuguccgcgcgcgcgcgcgcgcgugcgu
.....................uucguacguagGagagcagcu.......................................................................  1      1  7y1
.....................uucguacUuagcagagcagcu.......................................................................  2      1  7y1
.....................Cucguacguagcagagcagcu.......................................................................  2      1  7y1
.....................uucguacguagcagagcagcuU......................................................................  1      1  7y1
.....................uucguaUguagcagagcagcuc......................................................................  3      1  7y1
.....................uucguacguagcagagcagAuc......................................................................  9      1  7y1
.....................uucguacguagcagagcagcAc......................................................................  4      1  7y1
.....................uucguacguagcagaUcagcuc......................................................................  6      1  7y1
.....................uucAuacguagcagagcagcuc......................................................................  6      1  7y1
.....................uucguacguaUcagagcagcuc......................................................................  11     1  7y1
.....................uucguacgCagcagagcagcuc......................................................................  1      1  7y1
.....................uucguaGguagcagagcagcuc......................................................................  1      1  7y1
.....................uucguacguagcagagcUgcuc......................................................................  1      1  7y1
.....................uucguacguagcagagcGgcuc......................................................................  3      1  7y1
.....................uAcguacguagcagagcagcuc......................................................................  27     1  7y1
.....................uucgCacguagcagagcagcuc......................................................................  2      1  7y1
.....................uucguacguagcagagcCgcuc......................................................................  1      1  7y1
.....................uucCuacguagcagagcagcuc......................................................................  2      1  7y1
.....................uucguacguagUagagcagcuc......................................................................  1      1  7y1
.....................uucguacguagcagagcaCcuc......................................................................  5      1  7y1
.....................uucguacguagcagagAagcuc......................................................................  19     1  7y1
.....................uucguacUuagcagagcagcuc......................................................................  5      1  7y1
.....................uucgAacguagcagagcagcuc......................................................................  8      1  7y1
.....................uucguacguagcaUagcagcuc......................................................................  5      1  7y1
.....................uuUguacguagcagagcagcuc......................................................................  2      1  7y1
.....................Aucguacguagcagagcagcuc......................................................................  123    1  7y1
.....................uucguacguagGagagcagcuc......................................................................  3      1  7y1
.....................uucguacguagcagagcagcuc......................................................................  12187  0  7y1
.....................uucguGcguagcagagcagcuc......................................................................  1      1  7y1
.....................uucguacguagAagagcagcuc......................................................................  13     1  7y1
.....................uucguacguagcagagcaUcuc......................................................................  8      1  7y1
.....................uucguacguaCcagagcagcuc......................................................................  9      1  7y1
.....................uucguacguagcaCagcagcuc......................................................................  5      1  7y1
.....................uCcguacguagcagagcagcuc......................................................................  200    1  7y1
.....................uucguacguagcagUgcagcuc......................................................................  2      1  7y1
.....................uucguacguagcagagUagcuc......................................................................  1      1  7y1
.....................uucguacAuagcagagcagcuc......................................................................  3      1  7y1
.....................uucguacguagcGgagcagcuc......................................................................  1      1  7y1
.....................uucguacguagcagagcagcuA......................................................................  8      1  7y1
.....................uucgGacguagcagagcagcuc......................................................................  1      1  7y1
.....................Nucguacguagcagagcagcuc......................................................................  5      1  7y1
.....................uucguacguagcagagcagcGc......................................................................  1      1  7y1
.....................uucguacguagcagagcagcCc......................................................................  1      1  7y1
.....................uucguUcguagcagagcagcuc......................................................................  2      1  7y1
.....................uuAguacguagcagagcagcuc......................................................................  33     1  7y1
.....................uucguacgAagcagagcagcuc......................................................................  15     1  7y1
.....................uucguacguagcagagGagcuc......................................................................  2      1  7y1
.....................uucguacguaAcagagcagcuc......................................................................  2      1  7y1
.....................uucguacguagcagagcagcuG......................................................................  1      1  7y1
.....................Gucguacguagcagagcagcuc......................................................................  29     1  7y1
.....................uucguacCuagcagagcagcuc......................................................................  7      1  7y1
.....................uucguacguGgcagagcagcuc......................................................................  2      1  7y1
.....................uucguacguagcagagcagUuc......................................................................  1      1  7y1
.....................uucguacguagcagagcagGuc......................................................................  1      1  7y1
.....................uucguacguagcagaAcagcuc......................................................................  1      1  7y1
.....................uucguCcguagcagagcagcuc......................................................................  1      1  7y1
.....................uucguacguagcagaCcagcuc......................................................................  8      1  7y1
.....................uucguacguagcagCgcagcuc......................................................................  16     1  7y1
.....................uucguaAguagcagagcagcuc......................................................................  55     1  7y1
.....................uucguacguagcagCgcagcucc.....................................................................  4      1  7y1
.....................uucguacguagcagagcagAucc.....................................................................  5      1  7y1
.....................uucguacguagcaUagcagcucc.....................................................................  4      1  7y1
.....................uucguacAuagcagagcagcucc.....................................................................  1      1  7y1
.....................uucguacguagcagaCcagcucc.....................................................................  2      1  7y1
.....................uucguacguagcagagcagcCcc.....................................................................  1      1  7y1
.....................uucguacguagcGgagcagcucc.....................................................................  5      1  7y1
.....................uucUuacguagcagagcagcucc.....................................................................  3      1  7y1
.....................uucguacguagcagagcagGucc.....................................................................  1      1  7y1
.....................Gucguacguagcagagcagcucc.....................................................................  17     1  7y1
.....................uucAuacguagcagagcagcucc.....................................................................  5      1  7y1
```

Star Mature

gaccugcuucugggucggggguuucguacguagcagagcagcucccucgcugcgaucuauugaaagucagcccucgacacaaggguuuguccgcgcgcgcgcgcgcgcgcgugcgu			
.....................uucguacguagcagagcagcucA...	13	1	7y1
.....................uucguacguagcUgagcagcucc...	2	1	7y1
.....................uucguacgGagcagagcagcucc...	1	1	7y1
.....................uucguacguagcagagcagcuAc...	9	1	7y1
.....................uucguacguagcagagcagcGcc...	1	1	7y1
.....................uucguacguaCcagagcagcucc...	3	1	7y1
.....................uuUguacguagcagagcagcucc...	1	1	7y1
.....................uucguacguagcagagcCgcucc...	1	1	7y1
.....................uucguacguagcagagGagcucc...	2	1	7y1
.....................uucguacguagcagagUagcucc...	1	1	7y1
.....................Nucguacguagcagagcagcucc...	4	1	7y1
.....................uucguacCuagcagagcagcucc...	6	1	7y1
.....................uucguaGguagcagagcagcucc...	1	1	7y1
.....................uNcguacguagcagagcagcucc...	1	1	7y1
.....................uucgAacguagcagagcagcucc...	1	1	7y1
.....................uAcguacguagcagagcagcucc...	17	1	7y1
.....................uuAguacguagcagagcagcucc...	16	1	7y1
.....................uucguUcguagcagagcagcucc...	1	1	7y1
.....................uucguacguagcagagAagcucc...	8	1	7y1
.....................uuGguacguagcagagcagcucc...	2	1	7y1
.....................uucguacgAagcagagcagcucc...	6	1	7y1
.....................Aucguacguagcagagcagcucc...	71	1	7y1
.....................uucguacUuagcagagcagcucc...	2	1	7y1
.....................uucguacguagcagagcagcucc...	7274	0	7y1
.....................uucguaAguagcagagcagcucc...	26	1	7y1
.....................uucguacguagAagagcagcucc...	11	1	7y1
.....................uucguacguaUcagagcagcucc...	8	1	7y1
.....................uucguacguagcagagcagcAcc...	1	1	7y1
.....................uucguacguGgcagagcagcucc...	2	1	7y1
.....................uucguCcguagcagagcagcucc...	1	1	7y1
.....................uucguacguagcagagcaAcucc...	1	1	7y1
.....................uucguacguagcagagcagcucG...	1	1	7y1
.....................uucgCacguagcagagcagcucc...	3	1	7y1
.....................uucguacguagcagagcaUcucc...	3	1	7y1
.....................uucguacguagcaCagcagcucc...	1	1	7y1
.....................uucguacguagGagagcagcucc...	1	1	7y1
.....................uucguacguagcagaUcagcucc...	5	1	7y1
.....................uucguacguagUagagcagcucc...	1	1	7y1
.....................uucguacguagcagagcaCcucc...	1	1	7y1
.....................uucguacguGgcagagcagcuccc..	1	1	7y1
.....................uucguacguaCcagagcagcuccc..	2	1	7y1
.....................uucguacguagcagagcCgcuccc..	2	1	7y1
.....................uucguacUuagcagagcagcuccc..	1	1	7y1
.....................uucguacguaUcagagcagcuccc..	1	1	7y1
.....................uucguacguagcagagcagcuAcc..	7	1	7y1
.....................uucguacguagcagagcagcuccA..	2	1	7y1
.....................uAcguacguagcagagcagcuccc..	6	1	7y1
.....................Nucguacguagcagagcagcuccc..	1	1	7y1
.....................uucguacguagcagagcagcuccc..	3826	0	7y1
.....................uucguacCuagcagagcagcuccc..	1	1	7y1
.....................Aucguacguagcagagcagcuccc..	31	1	7y1
.....................uucguacguagcagagcagcuUcc..	1	1	7y1
.....................uucguacguagcagagcNgcuccc..	1	1	7y1
.....................uucguCcguagcagagcagcuccc..	1	1	7y1
.....................uucguacguagcagagcagcCccc..	1	1	7y1
.....................uuAguacguagcagagcagcuccc..	10	1	7y1
.....................uucguacguagcagagcagcucAc..	16	1	7y1
.....................uucguacguagcGgagcagcuccc..	1	1	7y1
.....................uucguacguagcagagcagcuGcc..	1	1	7y1
.....................uucAuacguagcagagcagcuccc..	1	1	7y1
.....................uucguacgAagcagagcagcuccc..	2	1	7y1
.....................uucUuacguagcagagcagcuccc..	1	1	7y1
.....................uucguacguagcagagAagcuccc..	4	1	7y1
.....................uucgAacguagcagagcagcuccc..	4	1	7y1
.....................Gucguacguagcagagcagcuccc..	13	1	7y1
.....................uucguaAguagcagagcagcuccc..	11	1	7y1
.....................uucguacguagAagagcagcuccc..	1	1	7y1
.....................uucguacguagcaCagcagcuccc..	1	1	7y1
.....................uucguacguagcagaCcagcuccc..	1	1	7y1
.....................uucguacguagcagagcaCcuccc..	4	1	7y1

Star **Mature**

```
gaccugcuucugggucgggguuucguacguagcagagcagcucccucgcugcgaucuauugaaagucagcccucgacacaaggguuuguccgcgcgcgcgcgcgcgcgugcgu
.....................uucguacguagcaUagcagcuccc....................................................................  6   1  7y1
.....................uucguacguagcagagUagcuccc....................................................................  1   1  7y1
.....................uucguacguagcagagcaUcuccc....................................................................  5   1  7y1
.....................uucguacguagcagagcaAcuccc....................................................................  1   1  7y1
.....................uucguacguagcagaUcagcuccc....................................................................  3   1  7y1
.....................uucguacguagcagagcagcAccc....................................................................  2   1  7y1
.....................uucguacguagcagagcagcucGc....................................................................  1   1  7y1
.....................uucCuacguagcagagcagcuccc....................................................................  1   1  7y1
.....................uucguacguagcagagcagcuUccu...................................................................  1   1  7y1
.....................uucUuacguagcagagcagcucccu...................................................................  4   1  7y1
.....................uucguacguagcagCgcagcucccu...................................................................  4   1  7y1
.....................uucguacguagcagagcagcGcccu...................................................................  2   1  7y1
.....................uucguacgCagcagagcagcucccu...................................................................  2   1  7y1
.....................uucguacguagcUgagcagcucccu...................................................................  1   1  7y1
.....................uucguacguagcagagcUgcucccu...................................................................  6   1  7y1
.....................uucguacguagcagagcagcucUcu...................................................................  5   1  7y1
.....................Gucguacguagcagagcagcucccu...................................................................  24  1  7y1
.....................uucguacguaAcagagcagcucccu...................................................................  1   1  7y1
.....................uucguacguagcagGgcagcucccu...................................................................  1   1  7y1
.....................Cucguacguagcagagcagcucccu...................................................................  1   1  7y1
.....................uucguacguagcagagcagcAcccu...................................................................  6   1  7y1
.....................uucguacguagcagagcagcuAccu...................................................................  23  1  7y1
.....................uucguacguagcagagAagcucccu...................................................................  11  1  7y1
.....................uucguacguagcagagcagcucAcu...................................................................  24  1  7y1
.....................uucguacguagcGgagcagcucccu...................................................................  2   1  7y1
.....................uucguacguagcagagcagcucccA...................................................................  13  1  7y1
.....................uNcguacguagcagagcagcucccu...................................................................  1   1  7y1
.....................uucguacguagcagagcCgcucccu...................................................................  2   1  7y1
.....................uucguacguagcagaCcagcucccu...................................................................  3   1  7y1
.....................uucguUcguagcagagcagcucccu...................................................................  1   1  7y1
.....................Nucguacguagcagagcagcucccu...................................................................  8   1  7y1
.....................uucguacgGagcagagcagcucccu...................................................................  1   1  7y1
.....................uucguacguagAagagcagcucccu...................................................................  7   1  7y1
.....................uucguacguagcagagcagcuccGu...................................................................  2   1  7y1
.....................uucguacgAagcagagcagcucccu...................................................................  9   1  7y1
.....................uuGguacguagcagagcagcucccu...................................................................  2   1  7y1
.....................uucCuacguagcagagcagcucccu...................................................................  1   1  7y1
.....................uucguaUguagcagagcagcucccu...................................................................  1   1  7y1
.....................uucguacguagcagUgcagcucccu...................................................................  1   1  7y1
.....................uucgCacguagcagagcagcucccu...................................................................  2   1  7y1
.....................uucguacguagcCgagcagcucccu...................................................................  2   1  7y1
.....................uucguacguagcagagcagAucccu...................................................................  5   1  7y1
.....................uucguacguagcagagcaAcucccu...................................................................  1   1  7y1
.....................uucguacguagcaUagcagcucccu...................................................................  5   1  7y1
.....................uucguacguagUagagcagcucccu...................................................................  1   1  7y1
.....................Aucguacguagcagagcagcucccu...................................................................  78  1  7y1
.....................uucguacguagcaCagcagcucccu...................................................................  3   1  7y1
.....................uucguacguagcagagcaCcucccu...................................................................  1   1  7y1
.....................uucguacguagcagagGagcucccu...................................................................  2   1  7y1
.....................uucguacguagcagagUagcucccu...................................................................  1   1  7y1
.....................uucguacguGgcagagcagcucccu...................................................................  1   1  7y1
.....................uucgAacguagcagagcagcucccu...................................................................  5   1  7y1
.....................uucguaGguagcagagcagcucccu...................................................................  1   1  7y1
.....................uucguacguagcagagcaUcucccu...................................................................  5   1  7y1
.....................uucguacguagcagagcagcCcccu...................................................................  2   1  7y1
.....................uucguacguagcagagcagcuGccu...................................................................  1   1  7y1
.....................uucguacguagcagagcagcucccG...................................................................  35  1  7y1
.....................uucguacguaUcagagcagcucccu...................................................................  5   1  7y1
.....................uucguacguaCcagagcagcucccu...................................................................  3   1  7y1
.....................uucguacguagcagagcGgcucccu...................................................................  8   1  7y1
.....................uucguacguagcagagcagcucccC...................................................................  1   1  7y1
.....................uucguacUuagcagagcagcucccu...................................................................  4   1  7y1
.....................uucguacguagcagagcagcuccAu...................................................................  6   1  7y1
.....................uucguacCuagcagagcagcucccu...................................................................  5   1  7y1
.....................uucguacguagGagagcagcucccu...................................................................  1   1  7y1
.....................uucguGcguagcagagcagcucccu...................................................................  1   1  7y1
.....................uucguaAguagcagagcagcucccu...................................................................  40  1  7y1
.....................uucguacAuagcagagcagcucccu...................................................................  3   1  7y1
.....................uuAguacguagcagagcagcucccu...................................................................  23  1  7y1
.....................uAcguacguagcagagcagcucccu...................................................................  21  1  7y1
```

Star **Mature**

gaccugcuucugggucgggguuucguacguagcagagcagcucccucgcugcgaucuauugaaagucagcccucgacacaaggguuuguccgcgcgcgcgcgcgcgcgugcgu			
.....................uucguacguagcagaUcagcucccu...	2	1	7y1
.....................uucguacguagcaAagcagcucccu...	2	1	7y1
.....................uCcguacguagcagagcagcucccu...	2	1	7y1
.....................uucguacguagcagagcagcucccu...	9826	0	7y1
.....................uucAuacguagcagagcagcucccu...	1	1	7y1
.....................uucguacguagcaUagcagcucccuc..	4	1	7y1
.....................uucguacguagcagagcagcuAccuc..	12	1	7y1
.....................uucguacguagcGgagcagcucccuc..	1	1	7y1
.....................uucguacguagcagagcaUcucccuc..	1	1	7y1
.....................uucCuacguagcagagcagcucccuc..	2	1	7y1
.....................uucguacguagcagagcagcuccAuc..	6	1	7y1
.....................uucguacguaUcagagcagcucccuc..	3	1	7y1
.....................uucguacguagcagGgcagcucccuc..	1	1	7y1
.....................uucguacguaCcagagcagcucccuc..	1	1	7y1
.....................uucguacAuagcagagcagcucccuc..	1	1	7y1
.....................uucguaAguagcagagcagcucccuc..	33	1	7y1
.....................uucguacguaAcagagcagcucccuc..	1	1	7y1
.....................uucguacgCagcagagcagcucccuc..	1	1	7y1
.....................Nucguacguagcagagcagcucccuc..	5	1	7y1
.....................uucguacguagcagagcagcGcccuc..	1	1	7y1
.....................uucguacguagcagagcaCcucccuc..	1	1	7y1
.....................uucguacguagcagagcagcucccuA..	1	1	7y1
.....................uucguacguagcagagcagcucccAc..	1	1	7y1
.....................uucguacguagcagagUagcucccuc..	1	1	7y1
.....................uucguacguagcagagcagcucccuc..	7376	0	7y1
.....................uucguacguagcagaUcagcucccuc..	4	1	7y1
.....................uucguacUuagcagagcagcucccuc..	2	1	7y1
.....................uucguacgAagcagagcagcucccuc..	6	1	7y1
.....................Gucguacguagcagagcagcucccuc..	10	1	7y1
.....................uucguacguagcagagcagcuUccuc..	1	1	7y1
.....................uucguacguagcagagcagcucccCc..	2	1	7y1
.....................uucguacguagcagUgcagcucccuc..	2	1	7y1
.....................uucguacguagcagagcUgcucccuc..	1	1	7y1
.....................uucguacguagcagagcCgcucccuc..	3	1	7y1
.....................uucguacguagcagagcagcAcccuc..	5	1	7y1
.....................uucguacguagcagagcaAcucccuc..	2	1	7y1
.....................uucguGcguagcagagcagcucccuc..	1	1	7y1
.....................uucguUcguagcagagcagcucccuc..	1	1	7y1
.....................uucguacCuagcagagcagcucccuc..	4	1	7y1
.....................uucguacguagcagagcagcucccuG..	1	1	7y1
.....................uucgAacguagcagagcagcucccuc..	4	1	7y1
.....................uucguacguagcUgagcagcucccuc..	2	1	7y1
.....................Cucguacguagcagagcagcucccuc..	1	1	7y1
.....................uucguacguagcagCgcagcucccuc..	8	1	7y1
.....................uucguacguagAagagcagcucccuc..	5	1	7y1
.....................Aucguacguagcagagcagcucccuc..	59	1	7y1
.....................uuGguacguagcagagcagcucccuc..	1	1	7y1
.....................uAcguacguagcagagcagcucccuc..	11	1	7y1
.....................uucguacguagcagagAagcucccuc..	11	1	7y1
.....................uuUguacguagcagagcagcucccuc..	1	1	7y1
.....................uucguacguagcagagcagAucccuc..	5	1	7y1
.....................uuAguacguagcagagcagcucccuc..	12	1	7y1
.....................uucUuacguagcagagcagcucccuc..	2	1	7y1
.....................uCcguacguagcagagcagcucccuc..	1	1	7y1
.....................uucgCacguagcagagcagcucccuc..	253	1	7y1
.....................uucguacguagcaAagcagcucccuc..	1	1	7y1
.....................uucguacguagcagaCcagcucccuc..	2	1	7y1
.....................uucguacguagcagagcagcucccGc..	1	1	7y1
.....................uucgGacguagcagagcagcucccuc..	1	1	7y1
.....................uucguacguagcagagcGgcucccuc..	2	1	7y1
.....................uucguacguagcagagcagcuGccuc..	4	1	7y1
.....................uucguacguagcagagcagcucAcuc..	19	1	7y1
.....................uucCuacguagcagagcagcucccucg...	1	1	7y1
.....................uucguCcguagcagagcagcucccucg...	1	1	7y1
.....................uAcguacguagcagagcagcucccucg...	2	1	7y1
.....................uucguacguagcagagcagcucccucC...	1	1	7y1
.....................uucguacguagcagGgcagcucccucg...	2	1	7y1
.....................uucguacguagcagagcagcucccucA...	1	1	7y1
.....................uucguacguagcagaUcagcucccucg...	3	1	7y1
.....................Nucguacguagcagagcagcucccucg...	1	1	7y1

Star **Mature**

gaccugcuucugggucggggguuucguacguagcagagcagcucccucgcugcgaucuauugaaagucagcccucgacacaaggguuuguccgcgcgcgcgcgcgcgcgugcgu			
.....................uucguacguagcagagcagcucAcucg...	4	1	7y1
.....................uucguacguagcagagcaCcucccucg...	1	1	7y1
.....................uucguacCuagcagagcagcucccucg...	3	1	7y1
.....................uucgAacguagcagagcagcucccucg...	2	1	7y1
.....................uucguacguaCcagagcagcucccucg...	1	1	7y1
.....................uucguacguagcagagcagcucccGcg...	1	1	7y1
.....................uuAguacguagcagagcagcucccucg...	3	1	7y1
.....................Gucguacguagcagagcagcucccucg...	1	1	7y1
.....................uucguaAguagcagagcagcucccucg...	5	1	7y1
.....................uucguacguagcagCgcagcucccucg...	2	1	7y1
.....................uucguGcguagcagagcagcucccucg...	1	1	7y1
.....................uucguacgCagcagagcagcucccucg...	1	1	7y1
.....................uucguacguagcagagcagcuAccucg...	1	1	7y1
.....................uucguacguagcagagcagcuccAucg...	1	1	7y1
.....................uucguacguagcagagcagcucccucg...	1431	0	7y1
.....................Aucguacguagcagagcagcucccucg...	13	1	7y1
.....................uucguacguagcagagcagcucccAcg...	1	1	7y1
.....................uucgCacguagcagagcagcucccucg...	1	1	7y1
.....................uucguacguagcagagAagcucccucg...	1	1	7y1
.....................uucguacguagcagagUagcucccucg...	1	1	7y1
.....................uucguacguagcagagcagAucccucgc..	1	1	7y1
.....................uuAguacguagcagagcagcucccucgc..	2	1	7y1
.....................uucguacguagcagagcaAcucccucgc..	1	1	7y1
.....................uucguacguagcagagcaCcucccucgc..	1	1	7y1
.....................uucguaAguagcagagcagcucccucgc..	3	1	7y1
.....................uucguacguagcagagcagcucccucgA..	1	1	7y1
.....................Cucguacguagcagagcagcucccucgc..	1	1	7y1
.....................uucguacguagcagagcagcucccucgc..	923	0	7y1
.....................uucgCacguagcagagcagcucccucgc..	1	1	7y1
.....................uucguacguagcagagcagGucccucgc..	1	1	7y1
.....................uAcguacguagcagagcagcucccucgc..	1	1	7y1
.....................uucguacguagcagagcagcuGccucgc..	1	1	7y1
.....................uucguacguaCcagagcagcucccucgc..	1	1	7y1
.....................uucguacguagcagagcagcuAccucgc..	2	1	7y1
.....................Aucguacguagcagagcagcucccucgc..	15	1	7y1
.....................uucguacgAagcagagcagcucccucgc..	1	1	7y1
.....................Gucguacguagcagagcagcucccucgc..	1	1	7y1
.....................uucguacguagcagagcGgcucccucgc..	1	1	7y1
.....................uucguacUuagcagagcagcucccucgc..	1	1	7y1
.....................uucguacguagcagagcagcucAcucgc..	1	1	7y1
.....................Nucguacguagcagagcagcucccucgc..	1	1	7y1
.....................uucguacguagcagagcagcucccuAgc..	1	1	7y1
.....................uucguacguagcagagcagcucccucgcA...	2	1	7y1
.....................uucguacUuagcagagcagcucccucgcu...	3	1	7y1
.....................uucguacguagcagagcagcCcccucgcu...	1	1	7y1
.....................uCcguacguagcagagcagcucccucgcu...	2	1	7y1
.....................uucguacguagcagagAagcucccucgcu...	3	1	7y1
.....................uucguacguagcagagcUgcucccucgcu...	2	1	7y1
.....................uucguacguagcagagcagcucAcucgcu...	9	1	7y1
.....................uucguacguagcagagcaUcucccucgcu...	2	1	7y1
.....................uucguaAguagcagagcagcucccucgcu...	13	1	7y1
.....................uucguacguagcagagcGgcucccucgcu...	2	1	7y1
.....................uucguacguagcagagcagcucGcucgcu...	1	1	7y1
.....................uucguacguagAagagcagcucccucgcu...	6	1	7y1
.....................uucguacCuagcagagcagcucccucgcu...	1	1	7y1
.....................uucguacguagcagagcagcucccucgGu...	1	1	7y1
.....................uucAuacguagcagagcagcucccucgcu...	1	1	7y1
.....................uucguGcguagcagagcagcucccucgcu...	1	1	7y1
.....................uucCuacguagcagagcagcucccucgcu...	4	1	7y1
.....................uucguacguagcagagcagcucccucgcu...	4044	0	7y1
.....................Gucguacguagcagagcagcucccucgcu...	6	1	7y1
.....................uucguacguagcagagcagcuUccucgcu...	1	1	7y1
.....................uucguacguagcagagcagAucccucgcu...	1	1	7y1
.....................uucguacguagcagagcagcucccAcgcu...	2	1	7y1
.....................uGcguacguagcagagcagcucccucgcu...	1	1	7y1
.....................uucguacguagcGgagcagcucccucgcu...	2	1	7y1
.....................uucguacguagcagagcagcucccucUcu...	1	1	7y1
.....................uucguacguagcagagcagcucccucgAu...	3	1	7y1
.....................uucguacguagcaUagcagcucccucgcu...	4	1	7y1
.....................uucguacAuagcagagcagcucccucgcu...	2	1	7y1

Star **Mature**

gaccugcuucugggucgggguuucguacguagcagagcagcucccucgcugcgaucuauugaaagucagcccucgacacaaggguuuguccgcgcgcgcgcgcgcgcgugcgu			
.....................uucguacgAagcagagcagcucccucgcu...	4	1	7y1
.....................uucguacguagcagagcagcuAccucgcu...	5	1	7y1
.....................uAcguacguagcagagcagcucccucgcu...	6	1	7y1
.....................uuGguacguagcagagcagcucccucgcu...	2	1	7y1
.....................uucguacguagcUgagcagcucccucgcu...	1	1	7y1
.....................uucguacguaCcagagcagcucccucgcu...	1	1	7y1
.....................uucguacguagcagagcagcucccucCcu...	4	1	7y1
.....................uucguacguagcagagcCgcucccucgcu...	1	1	7y1
.....................Aucguacguagcagagcagcucccucgcu...	36	1	7y1
.....................uNcguacguagcagagcagcucccucgcu...	1	1	7y1
.....................uuAguacguagcagagcagcucccucgcu...	8	1	7y1
.....................uucguacguagcagagcagcucccucgcG...	23	1	7y1
.....................uucguaUguagcagagcagcucccucgcu...	1	1	7y1
.....................uucguacguagcagagcagcuccAucgcu...	2	1	7y1
.....................uucguacguagcagagcagcucccuAgcu...	1	1	7y1
.....................uucguacguagcagagcagcAcccucgcu...	1	1	7y1
.....................uucgAacguagcagagcagcucccucgcu...	1	1	7y1
.....................uucgGacguagcagagcagcucccucgcu...	1	1	7y1
.....................Nucguacguagcagagcagcucccucgcu...	2	1	7y1
.....................uucCuacguagcagagcagcucccucgcug..	1	1	7y1
.....................uucguacguagcagagcagcucccuAgcug..	2	1	7y1
.....................uucguacgAagcagagcagcucccucgcug..	1	1	7y1
.....................uucguaAguagcagagcagcucccucgcug..	4	1	7y1
.....................uucguacUuagcagagcagcucccucgcug..	1	1	7y1
.....................Nucguacguagcagagcagcucccucgcug..	1	1	7y1
.....................uucguacguagcagaCcagcucccucgcug..	1	1	7y1
.....................Aucguacguagcagagcagcucccucgcug..	5	1	7y1
.....................uucguacguagcagagcagcucccucgcGg..	1	1	7y1
.....................uucguacguagcagagcagAucccucgcug..	1	1	7y1
.....................uucguacguagcagagcagcucccucgcug..	459	0	7y1
.....................uAcguacguagcagagcagcucccucgcug..	2	1	7y1
.....................uucguacCuagcagagcagcucccucgcug..	2	1	7y1
.....................Gucguacguagcagagcagcucccucgcugc...	1	1	7y1
.....................uucguGcguagcagagcagcucccucgcugc...	1	1	7y1
.....................uucguacguagcagagcagcucccucgcuCc...	1	1	7y1
.....................uucguacguagcaUagcagcucccucgcugc...	1	1	7y1
.....................uucguacUuagcagagcagcucccucgcugc...	1	1	7y1
.....................Aucguacguagcagagcagcucccucgcugc...	9	1	7y1
.....................uucguacguaCcagagcagcucccucgcugc...	1	1	7y1
.....................uucguacguagcagagcagcucAcucgcugc...	2	1	7y1
.....................uucguaAguagcagagcagcucccucgcugc...	1	1	7y1
.....................uucguacguagcagagcCgcucccucgcugc...	1	1	7y1
.....................uucguacguagcagagcagcuccAucgcugc...	1	1	7y1
.....................uucguacguagAagagcagcucccucgcugc...	2	1	7y1
.....................uuAguacguagcagagcagcucccucgcugc...	1	1	7y1
.....................uucguacguagcagaCcagcucccucgcugc...	1	1	7y1
.....................uucguacguagcagagcagcucccucgcugA...	1	1	7y1
.....................uAcguacguagcagagcagcucccucgcugc...	3	1	7y1
.....................uucguacguagcagGgcagcucccucgcugc...	1	1	7y1
.....................uucguacguagcagagcagcucccucgcugc...	924	0	7y1
......................ucguacCuagcagagcagc..	1	1	7y1
......................ucguacguagcagagcagc..	1251	0	7y1
......................ucguacguagAagagcagc..	3	1	7y1
......................ucguacguagcagagcagA..	1	1	7y1
......................ucguacgAagcagagcagc..	3	1	7y1
......................Acguacguagcagagcagc..	13	1	7y1
......................uAguacguagcagagcagc..	2	1	7y1
......................ucguaAguagcagagcagc..	2	1	7y1
......................ucguacguagcagCgcagc..	4	1	7y1
......................Gcguacguagcagagcagc..	1	1	7y1
......................ucguacUuagcagagcagc..	1	1	7y1
......................ucguacguagcagagcagG..	1	1	7y1
......................ucguacguagcagagcaUc..	2	1	7y1
......................ucguacguagcaCagcagc..	1	1	7y1
......................ucguacguagcGgagcagc..	1	1	7y1
......................ucguacguagcaCagcagcu...	2	1	7y1
......................uNguacguagcagagcagcu...	1	1	7y1
......................uAguacguagcagagcagcu...	6	1	7y1
......................ucUuacguagcagagcagcu...	2	1	7y1
......................ucguacguagcagagcagcG...	20	1	7y1

Star **Mature**

```
gaccugcuucugggucgggguuucguacguagcagagcagcucccucgcugcgaucuauugaaagucagcccucgacacaaggguuuguccgcgcgcgcgcgcgcgcgugcgu
......................ucguacguagcagagAagcu.......................................................................	6	1	7y1
......................ucguacguagAagagcagcu.......................................................................	5	1	7y1
......................ucguacguagcagagcaUcu.......................................................................	4	1	7y1
......................ucguacguagcagCgcagcu.......................................................................	4	1	7y1
......................Gcguacguagcagagcagcu.......................................................................	6	1	7y1
......................ucguacguagcagagcagcu.......................................................................	2186	0	7y1
......................ucguacguagcagagcagcA.......................................................................	1	1	7y1
......................uUguacguagcagagcagcu.......................................................................	1	1	7y1
......................ucguacguagcagaUcagcu.......................................................................	2	1	7y1
......................ucguacguaCcagagcagcu.......................................................................	1	1	7y1
......................ucguaAguagcagagcagcu.......................................................................	6	1	7y1
......................ucguacguagcagagcGgcu.......................................................................	1	1	7y1
......................ucguacguagcagUgcagcu.......................................................................	1	1	7y1
......................Acguacguagcagagcagcu.......................................................................	36	1	7y1
......................ucguacguaUcagagcagcu.......................................................................	2	1	7y1
......................ucguGcguagcagagcagcu.......................................................................	1	1	7y1
......................ucguacguagcagagcUgcu.......................................................................	1	1	7y1
......................ucguacgAagcagagcagcu.......................................................................	2	1	7y1
......................uAguacguagcagagcagcuc......................................................................	19	1	7y1
......................ucguacguagcagaUcagcuc......................................................................	6	1	7y1
......................ucguacguagcagagcaCcuc......................................................................	6	1	7y1
......................ucgAacguagcagagcagcuc......................................................................	1	1	7y1
......................ucguacguaCcagagcagcuc......................................................................	6	1	7y1
......................ucguacCuagcagagcagcuc......................................................................	4	1	7y1
......................ucUuacguagcagagcagcuc......................................................................	5	1	7y1
......................ucguacguagcagagcagcuc......................................................................	6627	0	7y1
......................Acguacguagcagagcagcuc......................................................................	79	1	7y1
......................ucguacguagcagagGagcuc......................................................................	1	1	7y1
......................ucguacguagAagagcagcuc......................................................................	9	1	7y1
......................ucguacguagcagGgcagcuc......................................................................	2	1	7y1
......................ucguacguagcagagcagcuA......................................................................	7	1	7y1
......................ucguacguagcagagUagcuc......................................................................	1	1	7y1
......................ucguacguagcaCagcagcuc......................................................................	238	1	7y1
......................ucguacguagcagUgcagcuc......................................................................	1	1	7y1
......................ucguacguagUagagcagcuc......................................................................	1	1	7y1
......................ucguacgAagcagagcagcuc......................................................................	9	1	7y1
......................ucguacUuagcagagcagcuc......................................................................	6	1	7y1
......................Ncguacguagcagagcagcuc......................................................................	4	1	7y1
......................ucguacgCagcagagcagcuc......................................................................	2	1	7y1
......................ucguacguagcagagcGgcuc......................................................................	1	1	7y1
......................uGguacguagcagagcagcuc......................................................................	1	1	7y1
......................uUguacguagcagagcagcuc......................................................................	1	1	7y1
......................ucguacguagcagagcagGuc......................................................................	1	1	7y1
......................ucguacguagcagCgcagcuc......................................................................	5	1	7y1
......................ucguacguaAcagagcagcuc......................................................................	1	1	7y1
......................ucguacguagcagaCcagcuc......................................................................	1	1	7y1
......................ucguacguagcagagcagcGc......................................................................	3	1	7y1
......................ucguacAuagcagagcagcuc......................................................................	1	1	7y1
......................ucguacguagcagagcagcAc......................................................................	4	1	7y1
......................ucCuacguagcagagcagcuc......................................................................	5	1	7y1
......................ucguacguagcagagcCgcuc......................................................................	1	1	7y1
......................ucguacguaUcagagcagcuc......................................................................	3	1	7y1
......................ucguacguagcagagcUgcuc......................................................................	2	1	7y1
......................ucguaAguagcagagcagcuc......................................................................	15	1	7y1
......................Gcguacguagcagagcagcuc......................................................................	7	1	7y1
......................ucguacguagcagagcaUcuc......................................................................	3	1	7y1
......................ucguUcguagcagagcagcuc......................................................................	1	1	7y1
......................ucguacguagcGgagcagcuc......................................................................	2	1	7y1
......................ucguacguagcagagAagcuc......................................................................	15	1	7y1
......................ucguaUguagcagagcagcuc......................................................................	2	1	7y1
......................ucguaGguagcagagcagcuc......................................................................	1	1	7y1
......................ucguacguagcagagcagcuG......................................................................	1	1	7y1
......................ucAuacguagcagagcagcuc......................................................................	1	1	7y1
......................ucguacguagcagagcagAuc......................................................................	3	1	7y1
......................ucguacguGgcagagcagcucc.....................................................................	1	1	7y1
......................ucguacgAagcagagcagcucc.....................................................................	1	1	7y1
......................ucguacguagAagagcagcucc.....................................................................	1	1	7y1
......................ucguacguagcGgagcagcucc.....................................................................	1	1	7y1
......................uGguacguagcagagcagcucc.....................................................................	1	1	7y1
......................ucguacguagcagagUagcucc.....................................................................	1	1	7y1
```

Star **Mature**

```
gaccugcuucugggucgggguuucguacguagcagagcagcucccucgcugcgaucuauugaaagucagcccucgacacaaggguuuguccgcgcgcgcgcgcgcgcgugcgu
......................Ncguacguagcagagcagcucc.....................................................................   1     1   7y1
......................ucguacguagcagaUcagcucc.....................................................................   1     1   7y1
......................ucguacguagcagagcagcucA.....................................................................   6     1   7y1
......................ucguacguagcagagcagcucU.....................................................................   1     1   7y1
......................ucUuacguagcagagcagcucc.....................................................................   2     1   7y1
......................ucguacguagcagagAagcucc.....................................................................   2     1   7y1
......................ucguacguagcagagcagAucc.....................................................................   1     1   7y1
......................Acguacguagcagagcagcucc.....................................................................   15    1   7y1
......................Gcguacguagcagagcagcucc.....................................................................   5     1   7y1
......................ucguacguagcagaCcagcucc.....................................................................   1     1   7y1
......................ucguacguagcagagcagcucc.....................................................................   1758  0   7y1
......................ucguacguagcagagcagcCcc.....................................................................   1     1   7y1
......................ucguaAguagcagagcagcucc.....................................................................   4     1   7y1
......................ucguacguaUcagagcagcucc.....................................................................   2     1   7y1
......................ucguaUguagcagagcagcucc.....................................................................   1     1   7y1
......................ucguacguagcagagcagcuAc.....................................................................   4     1   7y1
......................ucguacguagcagagcagcuUc.....................................................................   1     1   7y1
......................uAguacguagcagagcagcucc.....................................................................   1     1   7y1
......................ucguacguagcagGgcagcucc.....................................................................   1     1   7y1
......................ucguGcguagcagagcagcucc.....................................................................   1     1   7y1
......................ucguacguaCcagagcagcucc.....................................................................   4     1   7y1
......................ucguacguagcagaAcagcucc.....................................................................   1     1   7y1
......................ucCuacguagcagagcagcucc.....................................................................   2     1   7y1
......................ucguacguagcagUgcagcuccc....................................................................   1     1   7y1
......................Gcguacguagcagagcagcuccc....................................................................   4     1   7y1
......................ucguacguagcagagcagcuAcc....................................................................   3     1   7y1
......................ucguacguagcagagcagcuccc....................................................................   2876  0   7y1
......................ucguacguagcagagcagcAccc....................................................................   1     1   7y1
......................ucguacguagcagagcagcucAc....................................................................   5     1   7y1
......................ucguacguagAagagcagcuccc....................................................................   3     1   7y1
......................ucguaAguagcagagcagcuccc....................................................................   8     1   7y1
......................uAguacguagcagagcagcuccc....................................................................   2     1   7y1
......................ucguacguagcagagcaUcuccc....................................................................   1     1   7y1
......................ucguacguaUcagagcagcuccc....................................................................   1     1   7y1
......................uUguacguagcagagcagcuccc....................................................................   2     1   7y1
......................ucguacguagcagagcaCcuccc....................................................................   2     1   7y1
......................ucguacguagcagagAagcuccc....................................................................   4     1   7y1
......................ucguacguaCcagagcagcuccc....................................................................   2     1   7y1
......................ucguacguagcagCgcagcuccc....................................................................   7     1   7y1
......................ucguacguagcagagcagcuccA....................................................................   2     1   7y1
......................ucAuacguagcagagcagcuccc....................................................................   2     1   7y1
......................ucUuacguagcagagcagcuccc....................................................................   1     1   7y1
......................ucgAacguagcagagcagcuccc....................................................................   1     1   7y1
......................ucguacguagcagagcagcuGcc....................................................................   1     1   7y1
......................ucguUcguagcagagcagcuccc....................................................................   1     1   7y1
......................ucguacguagcagagcagAuccc....................................................................   4     1   7y1
......................Acguacguagcagagcagcuccc....................................................................   26    1   7y1
......................uGguacguagcagagcagcuccc....................................................................   1     1   7y1
......................ucguacCuagcagagcagcuccc....................................................................   1     1   7y1
......................ucguacgAagcagagcagcuccc....................................................................   1     1   7y1
......................ucguacguagcagagGagcuccc....................................................................   1     1   7y1
......................ucguacguagcagaAcagcuccc....................................................................   1     1   7y1
......................ucguacguagcaCagcagcuccc....................................................................   2     1   7y1
......................ucguacgAagcagagcagcucccu...................................................................   3     1   7y1
......................Ccguacguagcagagcagcucccu...................................................................   1     1   7y1
......................ucguacguagcagCgcagcucccu...................................................................   6     1   7y1
......................uUguacguagcagagcagcucccu...................................................................   1     1   7y1
......................ucguacguagcagagcagcucGcu...................................................................   1     1   7y1
......................ucguacguagcUgagcagcucccu...................................................................   1     1   7y1
......................ucguacguagcagagcagcuGccu...................................................................   1     1   7y1
......................ucguacguagcagagcagUucccu...................................................................   1     1   7y1
......................ucguacguaUcagagcagcucccu...................................................................   1     1   7y1
......................ucguacguagcagagcagcuAccu...................................................................   2     1   7y1
......................Gcguacguagcagagcagcucccu...................................................................   5     1   7y1
......................ucguacguagcagaCcagcucccu...................................................................   2     1   7y1
......................ucguacguagcaUagcagcucccu...................................................................   2     1   7y1
......................uAguacguagcagagcagcucccu...................................................................   6     1   7y1
......................ucguacguagcagagAagcucccu...................................................................   1     1   7y1
......................ucguacguagcagagcagcCcccu...................................................................   1     1   7y1
......................ucguacguagcagagcCgcucccu...................................................................   2     1   7y1
```

Star **Mature**

gaccugcuucugggucggggu uucguacguagcagagcagcucccucgcugcgaucuauugaaagucagcc cucgacacaaggguuugu ccgcgcgcgcgcgcgcgcgcgugcgu			
......................ucguacguGgcagagcagcucccu...	2	1	7y1
......................ucguacguagcagagcagAucccu...	1	1	7y1
......................ucguGcguagcagagcagcucccu...	1	1	7y1
......................ucguacguaCcagagcagcucccu...	1	1	7y1
......................ucguaAguagcagagcagcucccu...	3	1	7y1
......................ucguacguagcagagcagcucccG...	9	1	7y1
......................ucguacguagcGgagcagcucccu...	2	1	7y1
......................ucgAacguagcagagcagcucccu...	1	1	7y1
......................ucguacguagcagagcagcucccu...	2486	0	7y1
......................ucguacguagcagagcagcucAcu...	4	1	7y1
......................ucCuacguagcagagcagcucccu...	2	1	7y1
......................ucguacguagcagagUagcucccu...	1	1	7y1
......................ucguacguagcagagcagcuUccu...	1	1	7y1
......................ucguacguagcagagcagcucccA...	5	1	7y1
......................ucguacguagcagagcagGucccu...	1	1	7y1
......................ucguacguagAagagcagcucccu...	1	1	7y1
......................Acguacguagcagagcagcucccu...	21	1	7y1
......................ucguacguagcagagcagcuccAu...	6	1	7y1
......................ucguacUuagcagagcagcucccu...	3	1	7y1
......................ucguacCuagcagagcagcucccu...	1	1	7y1
......................uUguacguagcagagcagcucccuc..	1	1	7y1
......................ucguacCuagcagagcagcucccuc..	1	1	7y1
......................ucgAacguagcagagcagcucccuc..	1	1	7y1
......................ucguacguagcagagcagcuAccuc..	1	1	7y1
......................ucguacguagcagagcagcuUccuc..	1	1	7y1
......................ucguacguagAagagcagcucccuc..	1	1	7y1
......................ucCuacguagcagagcagcucccuc..	1	1	7y1
......................ucguacguagcagagcagcucccuG..	1	1	7y1
......................ucguacguagcagagcagcucccuc..	555	0	7y1
......................Acguacguagcagagcagcucccuc..	7	1	7y1
......................ucguacguaCcagagcagcucccuc..	1	1	7y1
......................ucguaAguagcagagcagcucccuc..	1	1	7y1
......................Ncguacguagcagagcagcucccuc..	1	1	7y1
......................ucguacguagcagagcagcAcccuc..	1	1	7y1
......................ucguacguagcagagcGgcucccuc..	1	1	7y1
......................ucguacguagcagagcagcucAcuc..	1	1	7y1
......................ucguacguagcagCgcagcucccucg...	1	1	7y1
......................Ncguacguagcagagcagcucccucg...	1	1	7y1
......................ucguacguagcagagcagcuccAucg...	1	1	7y1
......................ucguacguaCcagagcagcucccucg...	1	1	7y1
......................ucguacguagcagaUcagcucccucg...	1	1	7y1
......................ucguacgAagcagagcagcucccucg...	1	1	7y1
......................ucCuacguagcagagcagcucccucg...	1	1	7y1
......................ucguacguagcagagcaCcucccucg...	1	1	7y1
......................ucguacguagcCgagcagcucccucg...	1	1	7y1
......................ucguacguagcagagcagcucccucg...	219	0	7y1
......................ucguacAuagcagagcagcucccucg...	1	1	7y1
......................uAguacguagcagagcagcucccucg...	1	1	7y1
......................ucguaAguagcagagcagcucccucgc..	2	1	7y1
......................ucguacguagcagagcagcucccuAgc..	1	1	7y1
......................ucguacguagcagagcagcuAccucgc..	1	1	7y1
......................ucguacguagcagagcagcucccucgc..	270	0	7y1
......................Acguacguagcagagcagcucccucgc..	4	1	7y1
......................uAguacguagcagagcagcucccucgc..	2	1	7y1
......................ucguacguagcagagAagcucccucgc..	1	1	7y1
......................ucguacguagcagagcagcucccucAcu...	1	1	7y1
......................ucguacguagcagaAcagcucccucgcu...	1	1	7y1
......................ucguacguaAcagagcagcucccucgcu...	1	1	7y1
......................ucguacguagcagagcagcCcccucgcu...	1	1	7y1
......................ucguacguaCcagagcagcucccucgcu...	1	1	7y1
......................ucguacguagcagagcagcucccucgcA...	1	1	7y1
......................ucguacguagcagagcagcuAccucgcu...	1	1	7y1
......................ucguacUuagcagagcagcucccucgcu...	1	1	7y1
......................ucguacguagcagUgcagcucccucgcu...	1	1	7y1
......................ucguacguagcagagcagcucccAcgcu...	1	1	7y1
......................ucguacguagAagagcagcucccucgcu...	1	1	7y1
......................ucguacguagcagagcagcucccucgcu...	1342	0	7y1
......................uUguacguagcagagcagcucccucgcu...	1	1	7y1
......................ucguacguagcagagAagcucccucgcu...	3	1	7y1
......................ucguacAuagcagagcagcucccucgcu...	2	1	7y1

```
Star                                                                Mature
gaccugcuucugggucgggguuucguacguagcagagcagcucccucgcugcgaucuauugaaagucagcccucgacacaagguuuguccgcgcgcgcgcgcgcgcgugcgu
......................ucguacgAagcagagcagcucccucgcu..............................................................	4	1	7y1
......................ucguacguagcagagcagcucccucUcu..............................................................	1	1	7y1
......................ucguacguagcagagcagcuUccucgcu..............................................................	1	1	7y1
......................ucguaUguagcagagcagcucccucgcu..............................................................	1	1	7y1
......................ucguacguagcagagcagcucccucgcG..............................................................	16	1	7y1
......................ucguacguaUcagagcagcucccucgcu..............................................................	1	1	7y1
......................ucguacguagcagagcagcucGcucgcu..............................................................	1	1	7y1
......................ucguacCuagcagagcagcucccucgcu..............................................................	1	1	7y1
......................ucguacguagcagagcagcucAcucgcu..............................................................	1	1	7y1
......................ucguacguagcagagcagAucccucgcu..............................................................	2	1	7y1
......................ucguacguagcagagcagcucccuAgcu..............................................................	3	1	7y1
......................ucguacguagcagagcagcucccucCcu..............................................................	2	1	7y1
......................ucguaAguagcagagcagcucccucgcu..............................................................	1	1	7y1
......................ucguacguagcagagcagcuccAucgcu..............................................................	1	1	7y1
......................Acguacguagcagagcagcucccucgcu..............................................................	11	1	7y1
......................Ncguacguagcagagcagcucccucgcu..............................................................	3	1	7y1
......................uAguacguagcagagcagcucccucgcu..............................................................	2	1	7y1
......................ucguacguagcGgagcagcucccucgcu..............................................................	1	1	7y1
......................ucguacguagcagagcagcuAccucgcug.............................................................	3	1	7y1
......................ucguacguagcagagcagUucccucgcug.............................................................	1	1	7y1
......................uAguacguagcagagcagcucccucgcug.............................................................	3	1	7y1
......................Acguacguagcagagcagcucccucgcug.............................................................	17	1	7y1
......................ucguacguagcagagcagcuccAucgcug.............................................................	4	1	7y1
......................ucguacguagcagagcagcucccucUcug.............................................................	1	1	7y1
......................ucguacguagGagagcagcucccucgcug.............................................................	1	1	7y1
......................ucguacguaUcagagcagcucccucgcug.............................................................	1	1	7y1
......................ucguacguagcagagcagcucccuUgcug.............................................................	1	1	7y1
......................ucguacguagAagagcagcucccucgcug.............................................................	3	1	7y1
......................ucgAacguagcagagcagcucccucgcug.............................................................	1	1	7y1
......................ucguacguagcagagcagcucccucgcuC.............................................................	1	1	7y1
......................ucguGcguagcagagcagcucccucgcug.............................................................	1	1	7y1
......................ucguacguGgcagagcagcucccucgcug.............................................................	1	1	7y1
......................Gcguacguagcagagcagcucccucgcug.............................................................	3	1	7y1
......................ucguacCuagcagagcagcucccucgcug.............................................................	3	1	7y1
......................uGguacguagcagagcagcucccucgcug.............................................................	2	1	7y1
......................ucguacguaCcagagcagcucccucgcug.............................................................	1	1	7y1
......................ucguacguagcagagcagcuUccucgcug.............................................................	1	1	7y1
......................ucUuacguagcagagcagcucccucgcug.............................................................	1	1	7y1
......................ucguacguagcagagcagcucccAcgcug.............................................................	4	1	7y1
......................ucguaAguagcagagcagcucccucgcug.............................................................	2	1	7y1
......................ucguacguagcagagcagcucAcucgcug.............................................................	3	1	7y1
......................ucguacguagcaCagcagcucccucgcug.............................................................	1	1	7y1
......................ucCuacguagcagagcagcucccucgcug.............................................................	3	1	7y1
......................ucguacguagcagagcagcucccucCcug.............................................................	1	1	7y1
......................ucguacguagcagagcagcucccucgcug.............................................................	2333	0	7y1
......................ucguacgAagcagagcagcucccucgcug.............................................................	4	1	7y1
......................Ncguacguagcagagcagcucccucgcug.............................................................	1	1	7y1
......................ucguacguagcagagcagcucccucgcuU.............................................................	6	1	7y1
......................Ccguacguagcagagcagcucccucgcug.............................................................	1	1	7y1
......................ucguacguagcagagcagcCcccucgcug.............................................................	2	1	7y1
......................ucguacguagcagagcagcucccucgcGg.............................................................	8	1	7y1
......................ucguacguagUagagcagcucccucgcug.............................................................	1	1	7y1
......................ucguacguagcagagcagcucccucgAug.............................................................	1	1	7y1
......................ucguacguagcagCgcagcucccucgcug.............................................................	2	1	7y1
.......................cguacguagUagagcagc.......................................................................	1	1	7y1
.......................cguacguagcaUagcagc.......................................................................	1	1	7y1
.......................cguacguagcagaUcagc.......................................................................	2	1	7y1
.......................cguacguaAcagagcagc.......................................................................	1	1	7y1
.......................cguacguagcagagcCgc.......................................................................	1	1	7y1
.......................cguacguagcagagcagc.......................................................................	1761	0	7y1
.......................cguacguaUcagagcagc.......................................................................	3	1	7y1
.......................cguacguagcUgagcagc.......................................................................	1	1	7y1
.......................cguacguagcGgagcagc.......................................................................	2	1	7y1
.......................cguacAuagcagagcagc.......................................................................	1	1	7y1
.......................cguacguagcagagAagc.......................................................................	2	1	7y1
.......................cgAacguagcagagcagc.......................................................................	2	1	7y1
.......................cUuacguagcagagcagc.......................................................................	3	1	7y1
.......................cguacguagcagagcaUc.......................................................................	1	1	7y1
.......................Gguacguagcagagcagc.......................................................................	2	1	7y1
.......................cguacgCagcagagcagc.......................................................................	2	1	7y1
```

Star **Mature**

```
gaccugcuucugggucgggguuucguacguagcagagcagcucccucgcugcgaucuauugaaagucagcccucgacacaaggguuuguccgcgcgcgcgcgcgcgcgugcgu
.......................cguacguagcagCgcagc........................................................................ 2 1 7y1
.......................cguacguagAagagcagc........................................................................ 2 1 7y1
.......................cguacgAagcagagcagc........................................................................ 1 1 7y1
.......................cguCcguagcagagcagc........................................................................ 1 1 7y1
.......................cguacguagcagagcGgc........................................................................ 1 1 7y1
.......................cguacNuagcagagcagc........................................................................ 1 1 7y1
.......................cguacguagcaAagcagc........................................................................ 1 1 7y1
.......................cCuacguagcagagcagc........................................................................ 1 1 7y1
.......................cguacguagcagagcagA........................................................................ 2 1 7y1
.......................Aguacguagcagagcagc........................................................................ 6 1 7y1
.......................Nguacguagcagagcagc........................................................................ 2 1 7y1
.......................cAuacguagcagagcagc........................................................................ 1 1 7y1
.......................cguaAguagcagagcagc........................................................................ 1 1 7y1
.......................cCuacguagcagagcagcu....................................................................... 1 1 7y1
.......................Aguacguagcagagcagcu....................................................................... 5 1 7y1
.......................cguacguagUagagcagcu....................................................................... 1 1 7y1
.......................cguacguagcagagGagcu....................................................................... 2 1 7y1
.......................cUuacguagcagagcagcu....................................................................... 5 1 7y1
.......................cguacguagcagagcagcA....................................................................... 1 1 7y1
.......................cgAacguagcagagcagcu....................................................................... 1 1 7y1
.......................cAuacguagcagagcagcu....................................................................... 2 1 7y1
.......................cguacguagcagaAcagcu....................................................................... 1 1 7y1
.......................cguacguagcagaCcagcu....................................................................... 1 1 7y1
.......................cguacUuagcagagcagcu....................................................................... 1 1 7y1
.......................cguacguagcagagcagcu....................................................................... 1200 0 7y1
.......................cguacguagcagagcagAu....................................................................... 3 1 7y1
.......................cgCacguagcagagcagcu....................................................................... 1 1 7y1
.......................cguacguagAagagcagcu....................................................................... 3 1 7y1
.......................cguaAguagcagagcagcu....................................................................... 2 1 7y1
.......................cguacguagcagagcagcG....................................................................... 4 1 7y1
.......................cguacguagcaUagcagcu....................................................................... 1 1 7y1
.......................Gguacguagcagagcagcu....................................................................... 1 1 7y1
.......................cguacguagcagagAagcu....................................................................... 3 1 7y1
.......................cguacguagcagagcaUcu....................................................................... 1 1 7y1
.......................cguacguagcagCgcagcu....................................................................... 1 1 7y1
.......................Nguacguagcagagcagcu....................................................................... 1 1 7y1
.......................cguacguagcagagcaCcu....................................................................... 2 1 7y1
.......................cguacgAagcagagcagcu....................................................................... 3 1 7y1
.......................cguacguagcagagcaUcuc...................................................................... 5 1 7y1
.......................cguacguagcagagcaCcuc...................................................................... 4 1 7y1
.......................cguacguaAcagagcagcuc...................................................................... 1 1 7y1
.......................cguacguagUagagcagcuc...................................................................... 1 1 7y1
.......................cguacguagcagagcagUuc...................................................................... 1 1 7y1
.......................cguacguagcagagcagcAc...................................................................... 5 1 7y1
.......................cguacguagAagagcagcuc...................................................................... 22 1 7y1
.......................cguGcguagcagagcagcuc...................................................................... 2 1 7y1
.......................cguacguagcagUgcagcuc...................................................................... 1 1 7y1
.......................cUuacguagcagagcagcuc...................................................................... 5 1 7y1
.......................cguacguagcagagcagcGc...................................................................... 1 1 7y1
.......................cguacguaUcagagcagcuc...................................................................... 4 1 7y1
.......................cguacguagcagagUagcuc...................................................................... 1 1 7y1
.......................cguacguaCcagagcagcuc...................................................................... 2 1 7y1
.......................cguacguagcaUagcagcuc...................................................................... 3 1 7y1
.......................cguacguagcagagcagcuA...................................................................... 8 1 7y1
.......................cguacguagcagagAagcuc...................................................................... 29 1 7y1
.......................cguaGguagcagagcagcuc...................................................................... 1 1 7y1
.......................cguacguagcagagGagcuc...................................................................... 2 1 7y1
.......................cguacguagcagGgcagcuc...................................................................... 3 1 7y1
.......................cguacguagcagagcUgcuc...................................................................... 1 1 7y1
.......................cgAacguagcagagcagcuc...................................................................... 12 1 7y1
.......................Aguacguagcagagcagcuc...................................................................... 25 1 7y1
.......................cguacguagGagagcagcuc...................................................................... 1 1 7y1
.......................cguacAuagcagagcagcuc...................................................................... 4 1 7y1
.......................cguacguagcagagcagcuG...................................................................... 1 1 7y1
.......................cguacgAagcagagcagcuc...................................................................... 23 1 7y1
.......................cguacguagcaCagcagcuc...................................................................... 2 1 7y1
.......................cguacCuagcagagcagcuc...................................................................... 2 1 7y1
.......................Nguacguagcagagcagcuc...................................................................... 2 1 7y1
.......................cguacguagcagaCcagcuc...................................................................... 4 1 7y1
.......................cCuacguagcagagcagcuc...................................................................... 11 1 7y1
```

```
   Star                                                         Mature
gaccugcuucugggucggggguuucguacguagcagagcagcucccucgcugcgaucuauugaaagucagcccucgacacaaggguuuguccgcgcgcgcgcgcgcgcgcgugcgu
.......................cguacguagcagagcagcuc........................................................................    7571    0    7y1
.......................cguacguagcagagcGgcuc........................................................................    1       1    7y1
.......................cguacgCagcagagcagcuc........................................................................    2       1    7y1
.......................Gguacguagcagagcagcuc........................................................................    3       1    7y1
.......................cguaAguagcagagcagcuc........................................................................    10      1    7y1
.......................cguacUuagcagagcagcuc........................................................................    5       1    7y1
.......................cguacguagcagagcagAuc........................................................................    9       1    7y1
.......................Uguacguagcagagcagcuc........................................................................    1       1    7y1
.......................cguacguagcagCgcagcuc........................................................................    4       1    7y1
.......................cAuacguagcagagcagcuc........................................................................    1       1    7y1
.......................cguacguagcagaUcagcuc........................................................................    4       1    7y1
.......................cguacguagcGgagcagcuc........................................................................    2       1    7y1
.......................cguacguagcagagcagcAcc.......................................................................    1       1    7y1
.......................cguacguagcagagAagcucc.......................................................................    1       1    7y1
.......................cguacguagcagagcagcuAc.......................................................................    1       1    7y1
.......................cguacguagcagCgcagcucc.......................................................................    1       1    7y1
.......................Aguacguagcagagcagcucc.......................................................................    1       1    7y1
.......................cguacguagcagagcagcucc.......................................................................    434     0    7y1
.......................cUuacguagcagagcagcucc.......................................................................    1       1    7y1
.......................cguacguagcagagNagcucc.......................................................................    1       1    7y1
.......................cguacguagcagaUcagcucc.......................................................................    1       1    7y1
.......................cguacguagcagagGagcucc.......................................................................    1       1    7y1
.......................cguacguaCcagagcagcucc.......................................................................    1       1    7y1
.......................cAuacguagcagagcagcucc.......................................................................    1       1    7y1
.......................cguaAguagcagagcagcucc.......................................................................    1       1    7y1
.......................cguacguagcagagAagcuccc......................................................................    2       1    7y1
.......................cUuacguagcagagcagcuccc......................................................................    3       1    7y1
.......................cguacguagcagagcagcAccc......................................................................    1       1    7y1
.......................cguacguagcagagcagcuccA......................................................................    2       1    7y1
.......................cguacgAagcagagcagcuccc......................................................................    1       1    7y1
.......................cguacguagcagagcagcuccc......................................................................    660     0    7y1
.......................Aguacguagcagagcagcuccc......................................................................    3       1    7y1
.......................cguacguagcagagcaUcuccc......................................................................    2       1    7y1
.......................cguacguagAagagcagcuccc......................................................................    1       1    7y1
.......................cguacguagcagUgcagcuccc......................................................................    1       1    7y1
.......................cguaAguagcagagcagcuccc......................................................................    1       1    7y1
.......................cguacguagcagagcagcuGcc......................................................................    1       1    7y1
.......................cguacguagcagagcagcuAcc......................................................................    1       1    7y1
.......................cguacguagcagagcaUcucccu.....................................................................    2       1    7y1
.......................cguacguagcagagcagcucccA.....................................................................    1       1    7y1
.......................cguaUguagcagagcagcucccu.....................................................................    1       1    7y1
.......................cguacguagcagagcagcucccG.....................................................................    6       1    7y1
.......................cguacguagcagagcagcuccAu.....................................................................    2       1    7y1
.......................cguacguagcagagcGgcucccu.....................................................................    2       1    7y1
.......................cguacguagcagagcagcuAccu.....................................................................    2       1    7y1
.......................cguacguagcagagAagcucccu.....................................................................    1       1    7y1
.......................cAuacguagcagagcagcucccu.....................................................................    1       1    7y1
.......................cgAacguagcagagcagcucccu.....................................................................    1       1    7y1
.......................cguacguagcagagcagcAcccu.....................................................................    1       1    7y1
.......................cUuacguagcagagcagcucccu.....................................................................    2       1    7y1
.......................cCuacguagcagagcagcucccu.....................................................................    1       1    7y1
.......................cguacguagcagagcagcucccu.....................................................................    943     0    7y1
.......................cguacguagcagaUcagcucccu.....................................................................    1       1    7y1
.......................cguacguagcagagcUgcucccu.....................................................................    1       1    7y1
.......................cguacguagcaUagcagcucccu.....................................................................    1       1    7y1
.......................cguacguagcagagcagcucGcu.....................................................................    1       1    7y1
.......................cguacguagAagagcagcucccu.....................................................................    1       1    7y1
.......................cguacguagcagagcCgcucccu.....................................................................    1       1    7y1
.......................cguaAguagcagagcagcucccu.....................................................................    1       1    7y1
.......................cguacguagcagagcagGucccuc....................................................................    1       1    7y1
.......................cguacCuagcagagcagcucccuc....................................................................    2       1    7y1
.......................cguacguagcagagcagcucccAc....................................................................    2       1    7y1
.......................cguacAuagcagagcagcucccuc....................................................................    1       1    7y1
.......................cguacgAagcagagcagcucccuc....................................................................    4       1    7y1
.......................cguacguagcagaUcagcucccuc....................................................................    2       1    7y1
.......................cguacguagcagagcagcucAcuc....................................................................    2       1    7y1
.......................Nguacguagcagagcagcucccuc....................................................................    1       1    7y1
.......................cguacguagcagGgcagcucccuc....................................................................    1       1    7y1
.......................cUuacguagcagagcagcucccuc....................................................................    3       1    7y1
.......................cguacguagcagCgcagcucccuc....................................................................    2       1    7y1
```

Star **Mature**

gaccugcuucugggucgggguuucguacguagcagagcagcucccucgcugcgaucuauugaaagucagcccucgacacaaggguuuguccgcgcgcgcgcgcgcgcgugcgu			
.......................cCuacguagcagagcagcucccuc..	1	1	7y1
.......................cguacguaCcagagcagcucccuc..	1	1	7y1
.......................cguacguagcagagcagcucccuc..	744	0	7y1
.......................cguacguagcagagcagcAcccuc..	1	1	7y1
.......................cguacguagcaCagcagcucccuc..	1	1	7y1
.......................cguacguagcagagAagcucccuc..	4	1	7y1
.......................cguacguagcagaAcagcucccuc..	2	1	7y1
.......................cguacgGagcagagcagcucccuc..	1	1	7y1
.......................cguacguagcagagcagcuAccuc..	1	1	7y1
.......................cguacgAagcagagcagcucccucg...	1	1	7y1
.......................cguacguaUcagagcagcucccucg...	1	1	7y1
.......................cguacguagcagaUcagcucccucg...	1	1	7y1
.......................cguacguagcagGgcagcucccucg...	1	1	7y1
.......................cguacguagcagagcagcucccucg...	297	0	7y1
.......................cguacguagcagagAagcucccucg...	1	1	7y1
.......................cguacUuagcagagcagcucccucg...	1	1	7y1
.......................cUuacguagcagagcagcucccucg...	1	1	7y1
.......................Aguacguagcagagcagcucccucg...	2	1	7y1
.......................cguaAguagcagagcagcucccucgc..	1	1	7y1
.......................cguacguagcagUgcagcucccucgc..	1	1	7y1
.......................cguacCuagcagagcagcucccucgc..	1	1	7y1
.......................cguacguagcagagcagcucccucCc..	1	1	7y1
.......................cguacguagcagagcagAucccucgc..	1	1	7y1
.......................cguacguagcagagcagcucccucgc..	264	0	7y1
.......................cguacguagcagagAagcucccucgc..	1	1	7y1
.......................cguacgAagcagagcagcucccucgc..	1	1	7y1
.......................cguacguagcagagcagAucccucgcu...	1	1	7y1
.......................cguacguagcagagcagcucccGcgcu...	1	1	7y1
.......................cguacguagcaUagcagcucccucgcu...	1	1	7y1
.......................cguacguagAagagcagcucccucgcu...	1	1	7y1
.......................cUuacguagcagagcagcucccucgcu...	3	1	7y1
.......................cguacguagcagagcagcucccucgcu...	407	0	7y1
.......................Aguacguagcagagcagcucccucgcu...	2	1	7y1
.......................cguacgAagcagagcagcucccucgcu...	2	1	7y1
.......................cguaUguagcagagcagcucccucgcu...	264	1	7y1
.......................cguaAguagcagagcagcucccucgcu...	2	1	7y1
.......................cCuacguagcagagcagcucccucgcu...	1	1	7y1
.......................cguacguagcagagcagcucccucgcG...	3	1	7y1
.......................cguacguagcagagcagcuGccucgcu...	1	1	7y1
.......................cAuacguagcagagcagcucccucgcug..	2	1	7y1
.......................cguacguagcagagcagcucAcucgcug..	1	1	7y1
.......................cguacguagcagagcagcucccucgcuC..	1	1	7y1
.......................cguacguaCcagagcagcucccucgcug..	2	1	7y1
.......................Aguacguagcagagcagcucccucgcug..	7	1	7y1
.......................cguacguagcagagcagcucccucUcug..	2	1	7y1
.......................cguacguagcagagcagcucccucgcGg..	8	1	7y1
.......................cCuacguagcagagcagcucccucgcug..	1	1	7y1
.......................cUuacguagcagagcagcucccucgcug..	2	1	7y1
.......................Nguacguagcagagcagcucccucgcug..	2	1	7y1
.......................cguacguagcagagcagcuAccucgcug..	2	1	7y1
.......................cguacguagcGgagcagcucccucgcug..	1	1	7y1
.......................Gguacguagcagagcagcucccucgcug..	1	1	7y1
.......................cgCacguagcagagcagcucccucgcug..	1	1	7y1
.......................cguacguagcagagcagcucccucgcuA..	1	1	7y1
.......................cguacguagcagagcagcucccucgcug..	1992	0	7y1
.......................cguaAguagcagagcagcucccucgcug..	1	1	7y1
.......................cguacguagcagagcagcuccAucgcug..	1	1	7y1
.......................cguacguagcagCgcagcucccucgcug..	1	1	7y1
.......................cguacgAagcagagcagcucccucgcug..	2	1	7y1
.......................cguacUuagcagagcagcucccucgcug..	1	1	7y1
.......................cguacguagcagagAagcucccucgcug..	2	1	7y1
.......................cguacguagcagagcagcucccucgcuU..	1	1	7y1
.......................cguacCuagcagagcagcucccucgcug..	2	1	7y1
.......................cguacguagcagagcagcuGccucgcug..	1	1	7y1
.......................cgGacguagcagagcagcucccucgcug..	1	1	7y1
.......................cguacguagcagagcagcucccucgcAg..	4	1	7y1
.......................cgAacguagcagagcagcucccucgcug..	2	1	7y1
.......................cguacguagcagagcagcucGcucgcug..	1	1	7y1
.......................cguacguagcagagcagcucccuAgcug..	1	1	7y1
.......................cguacguagcaUagcagcucccucgcug..	1	1	7y1

Star **Mature**

```
gaccugcuucugggucgggguuucguacguagcagagcagcucccucgcugcgaucuauugaaagucagcccucgacacaaggguuuguccgcgcgcgcgcgcgcgcgugcgu
.......................cguacguagAagagcagcucccucgcug..............................................................  3    1  7y1
.......................cguacguagcagagcagcucccucgAug..............................................................  2    1  7y1
........................gAacguagcagagcagcu.......................................................................  2    1  7y1
........................guaAguagcagagcagcu.......................................................................  1    1  7y1
........................guacguagcagagcagcu.......................................................................  154  0  7y1
........................Cuacguagcagagcagcuc......................................................................  2    1  7y1
........................guacguagcagagcagAuc......................................................................  1    1  7y1
........................guacguagcagagcagcuc......................................................................  173  0  7y1
........................guacgAagcagagcagcuc......................................................................  1    1  7y1
........................Cuacguagcagagcagcucccucgc................................................................  1    1  7y1
........................guacguagcagagcagcucccucgcu...............................................................  164  0  7y1
........................guacguagcagagcagAucccucgcu...............................................................  2    1  7y1
........................guacguagcagagAagcucccucgcu...............................................................  1    1  7y1
........................Uuacguagcagagcagcucccucgcu...............................................................  1    1  7y1
........................guacguagcagagcagcucccucgcG...............................................................  3    1  7y1
........................guacgAagcagagcagcucccucgcu...............................................................  1    1  7y1
........................guacguaUcagagcagcucccucgcu...............................................................  1    1  7y1
........................guGcguagcagagcagcucccucgcu...............................................................  1    1  7y1
........................guacguagcagagcagGucccucgcu...............................................................  1    1  7y1
........................guacguagcagagcGgcucccucgcug..............................................................  1    1  7y1
........................guaAguagcagagcagcucccucgcug..............................................................  1    1  7y1
........................guacguagcagagcagcucccucgcug..............................................................  61   0  7y1
.........................uacguagcagagcagcuA......................................................................  2    1  7y1
.........................uacguagcagagcaAcuc......................................................................  1    1  7y1
.........................uacguagcagagAagcuc......................................................................  1    1  7y1
.........................uacguagcagagcagcuc......................................................................  616  0  7y1
.........................uacguaUcagagcagcuc......................................................................  2    1  7y1
.........................Aacguagcagagcagcuc......................................................................  15   1  7y1
.........................uaAguagcagagcagcuc......................................................................  1    1  7y1
.........................uacguagcagagGagcuc......................................................................  1    1  7y1
.........................uacguagcagagcagcAc......................................................................  2    1  7y1
.........................uacguagcagagcCgcuc......................................................................  1    1  7y1
.........................uacguagcagagcagcuG......................................................................  1    1  7y1
.........................Gacguagcagagcagcuccc....................................................................  1    1  7y1
.........................uacguagcGgagcagcuccc....................................................................  1    1  7y1
.........................uacguagcagCgcagcuccc....................................................................  1    1  7y1
.........................uacguagcagagcagcAccc....................................................................  1    1  7y1
.........................Aacguagcagagcagcuccc....................................................................  3    1  7y1
.........................uacguagcagagcagcuccc....................................................................  193  0  7y1
.........................uacguagcagagcaCcucccucgcu...............................................................  1    1  7y1
.........................uaAguagcagagcagcucccucgcu...............................................................  1    1  7y1
.........................uacguagcagagcUgcucccucgcu...............................................................  1    1  7y1
.........................uacguagcagagcagcucccucgcu...............................................................  493  0  7y1
.........................Nacguagcagagcagcucccucgcu...............................................................  1    1  7y1
.........................uacguagcagagcagcuAccucgcu...............................................................  1    1  7y1
.........................uacguagcagagAagcucccucgcu...............................................................  2    1  7y1
.........................Aacguagcagagcagcucccucgcu...............................................................  5    1  7y1
.........................uacguagcagagcagcucccucUcu...............................................................  1    1  7y1
.........................uacguagcagagcagcucccucgcG...............................................................  4    1  7y1
.........................uacguagcaCagcagcucccucgcu...............................................................  2    1  7y1
.........................uacguagcagagcagcucccuUgcu...............................................................  1    1  7y1
.........................uacguaCcagagcagcucccucgcu...............................................................  2    1  7y1
.........................uacguagcagagcagcucccucgGu...............................................................  1    1  7y1
.........................uacguagcagagcaAcucccucgcug..............................................................  1    1  7y1
.........................uGcguagcagagcagcucccucgcug..............................................................  1    1  7y1
.........................uacguagcagagcagcucccAcgcug..............................................................  1    1  7y1
.........................uacguagcagagcagcucAcucgcug..............................................................  4    1  7y1
.........................uacguagcagagcagcucccucgcuA..............................................................  1    1  7y1
.........................uacguagcagagcagcucccucgcuC..............................................................  2    1  7y1
.........................uacguGgcagagcagcucccucgcug..............................................................  1    1  7y1
.........................uacgAagcagagcagcucccucgcug..............................................................  2    1  7y1
.........................uacguagcagagcagcAcccucgcug..............................................................  1    1  7y1
.........................uacguagcagagcagcucccuAgcug..............................................................  1    1  7y1
.........................uacguagcagagcagcuccGucgcug..............................................................  1    1  7y1
.........................uacguagcagagcaUcucccucgcug..............................................................  2    1  7y1
.........................uacguagcagagcagcucGcucgcug..............................................................  1    1  7y1
.........................uacguagcagagcagcuAccucgcug..............................................................  2    1  7y1
.........................uacguagcUgagcagcucccucgcug..............................................................  1    1  7y1
.........................uacguagcagagcagcucccucgcGg..............................................................  19   1  7y1
.........................uacguagcagagcaNcucccucgcug..............................................................  1    1  7y1
```

Star **Mature**

gaccugcuucugggucggggguuucguacguagcagagcagcucccucgcugcgaucuauugaaagucagcccucgacacaaggguuuguccgcgcgcgcgcgcgcgcgugcgu			
.........................uacguagcagagcagcucccucgcAg..	1	1	7y1
.........................uacguagcagagcagcucccucgAug..	2	1	7y1
.........................Aacguagcagagcagcucccucgcug..	17	1	7y1
.........................uacguUgcagagcagcucccucgcug..	1	1	7y1
.........................uacguagcagagcagcucccucgcCg..	1	1	7y1
.........................uUcguagcagagcagcucccucgcug..	1	1	7y1
.........................uacguagcagagcagcucccucgcug..	1768	0	7y1
.........................uacguaCcagagcagcucccucgcug..	1	1	7y1
.........................uacguagAagagcagcucccucgcug..	3	1	7y1
.........................uaAguagcagagcagcucccucgcug..	5	1	7y1
.........................uacguagcagagcCgcucccucgcug..	1	1	7y1
.........................uaGguagcagagcagcucccucgcug..	3	1	7y1
.........................uacguagcagagcagcucccucgcuU..	1	1	7y1
.........................Gacguagcagagcagcucccucgcug..	2	1	7y1
.........................uacguaUcagagcagcucccucgcug..	1	1	7y1
..........................acguagcagaUcagcucccucgcu...	1	1	7y1
..........................acguagcagagcagcucccucgcu...	416	0	7y1
..........................acguagcagagcagcucccucgcG...	9	1	7y1
..........................Gcguagcagagcagcucccucgcu...	5	1	7y1
..........................acguagcagagcagAucccucgcu...	1	1	7y1
..........................acguagcGgagcagcucccucgcu...	2	1	7y1
..........................acguagcagagcagcucccAcgcu...	1	1	7y1
..........................acguagcagagcaUcucccucgcu...	3	1	7y1
..........................acguaCcagagcagcucccucgcu...	1	1	7y1
..........................acgAagcagagcagcucccucgcu...	1	1	7y1
..........................acguaUcagagcagcucccucgcu...	1	1	7y1
..........................acguagcagagAagcucccucgcu...	1	1	7y1
..........................aAguagcagagcagcucccucgcu...	2	1	7y1
..........................acguagcagagcagcuAccucgcu...	1	1	7y1
..........................acguagAagagcagcucccucgcu...	2	1	7y1
..........................acguagcagagcCgcucccucgcu...	1	1	7y1
..........................acguagcaUagcagcucccucgcug..	1	1	7y1
..........................acguagAagagcagcucccucgcug..	2	1	7y1
..........................acguagcagaUcagcucccucgcug..	2	1	7y1
..........................acguagcagagAagcucccucgcug..	1	1	7y1
..........................acguagcagagcagcucccucgcGg..	5	1	7y1
..........................acguagcagagcGgcucccucgcug..	1	1	7y1
..........................acguagcagagcagcucccucgcug..	537	0	7y1
..........................acgAagcagagcagcucccucgcug..	1	1	7y1
..........................acCuagcagagcagcucccucgcug..	1	1	7y1
..........................acguagcagagcagcucccucgcuC..	1	1	7y1
..........................acguagcagagcagcuccAucgcug..	2	1	7y1
..........................Gcguagcagagcagcucccucgcug..	1	1	7y1
...........................cguagcagagcagcuccc..	199	0	7y1
...........................cguagcagagcagcucGc..	1	1	7y1
...........................cguagcagagcagcucAcu...	1	1	7y1
...........................cguagAagagcagcucccu...	3	1	7y1
...........................cguagcagagcagcuAccu...	2	1	7y1
...........................cguagcagaUcagcucccu...	1	1	7y1
...........................cgGagcagagcagcucccu...	1	1	7y1
...........................cguagcagagcagcucccu...	204	0	7y1
...........................cguagcagaCcagcucccu...	1	1	7y1
...........................cguaCcagagcagcucccucgcug..	1	1	7y1
...........................cCuagcagagcagcucccucgcug..	1	1	7y1
...........................cguagcagagcagcuUccucgcug..	1	1	7y1
...........................cguagcagagcagcucccucAcug..	1	1	7y1
...........................cguagcagCgcagcucccucgcug..	1	1	7y1
...........................cguagcagagcagcucccucgAug..	1	1	7y1
...........................cguagcagagcagcucccucgcuU..	2	1	7y1
...........................cguagcagagcagcucccucgcug..	541	0	7y1
...........................cguaUcagagcagcucccucgcug..	1	1	7y1
...........................cguagcagagcagAucccucgcug..	1	1	7y1
...........................Nguagcagagcagcucccucgcug..	2	1	7y1
...........................cguagcagagcagcucccAcgcug..	1	1	7y1
...........................cgAagcagagcagcucccucgcug..	1	1	7y1
...........................Gguagcagagcagcucccucgcug..	1	1	7y1
...........................cguagcagagcagcuGccucgcug..	1	1	7y1
...........................cguagcagagcagcAcccucgcug..	1	1	7y1
............................guagcagagcagcucccucgc..	2	0	7y1
............................guagcagagcagcucccucgcug..	73	0	7y1

Star **Mature**

```
gaccugcuucugggucgggguuucguacguagcagagcagcucccucgcugcgaucuauugaaagucagcccucgacacaaggguuuguccgcgcgcgcgcgcgcgcgugcgu
..............................agcagagcagcucGcucgcug..............................................................   1     1   7y1
..............................aUcagagcagcucccucgcug..............................................................   1     1   7y1
..............................agcagagcagcuccAucgcug..............................................................   2     1   7y1
..............................agcagagcagcucccucgcug..............................................................   273   0   7y1
..............................agcagagAagcucccucgcug..............................................................   1     1   7y1
..............................agcagagcagcuAccucgcug..............................................................   1     1   7y1
..............................aAcagagcagcucccucgcug..............................................................   1     1   7y1
..............................agcagagcagcucAcucgcug..............................................................   1     1   7y1
..............................agcagagcagcucccucgAug..............................................................   1     1   7y1
................................cagagcaUcucccucgcu...............................................................   1     1   7y1
................................cagagcagcucccucgcu...............................................................   129   0   7y1
................................cagCgcagcucccucgcu...............................................................   1     1   7y1
................................cagagcagcucAcucgcu...............................................................   1     1   7y1
................................cagagcagcucccucgcG...............................................................   11    1   7y1
................................cagagcagcuccGucgcu...............................................................   1     1   7y1
................................cagagcagcAcccucgcu...............................................................   1     1   7y1
....................................gcagcuUccucgcugcgaucu........................................................   1     1   7y1
....................................gcagcucAcucgcugcgaucu........................................................   1     1   7y1
....................................gcagcuAccucgcugcgaucu........................................................   3     1   7y1
....................................gAagcucccucgcugcgaucu........................................................   1     1   7y1
....................................gcagcucccucgcuCcgaucu........................................................   1     1   7y1
....................................gcagcucccucgcugcgaucu........................................................   394   0   7y1
....................................gcagcucccucCcugcgaucu........................................................   1     1   7y1
....................................gcagcucccucgAugcgaucu........................................................   1     1   7y1
....................................gcagcucccucgcugcgaucA........................................................   1     1   7y1
....................................gcagcucccucgcAgcgaucu........................................................   2     1   7y1
....................................gcagcucccucgcugcgGucu........................................................   1     1   7y1
....................................gcagAucccucgcugcgaucu........................................................   1     1   7y1
....................................gcagcucccucgGugcgaucu........................................................   1     1   7y1
....................................gcagcucccucgcCgcgaucu........................................................   1     1   7y1
....................................gcagcucccucgcugcgaucuauugaaagu...............................................   224   0   7y1
....................................Ccagcucccucgcugcgaucuauugaaagu...............................................   1     1   7y1
....................................gcagcucccucgcugcgaucuauugaaagG...............................................   1     1   7y1
....................................gcagcucccuAgcugcgaucuauugaaagu...............................................   1     1   7y1
....................................gcagcucccucUcugcgaucuauugaaagu...............................................   1     1   7y1
....................................gcagcucccucgcugcgaucuaAugaaagu...............................................   1     1   7y1
....................................gcagcuUccucgcugcgaucuauugaaagu...............................................   1     1   7y1
....................................Ncagcucccucgcugcgaucuauugaaaguca.............................................   1     1   7y1
....................................gcagcucccucgcugcgaucuauugaaaguca.............................................   182   0   7y1
....................................gcagcucccucgcugcgaucAauugaaaguca.............................................   1     1   7y1
....................................gcagcucAcucgcugcgaucuauugaaaguca.............................................   2     1   7y1
....................................gcagcucccuAgcugcgaucuauugaaaguca.............................................   1     1   7y1
....................................gcagcucccucgcugcgauAuauugaaaguca.............................................   1     1   7y1
....................................gcagcucccucgcugcgaucuauAgaaaguca.............................................   1     1   7y1
....................................gcagGucccucgcugcgaucuauugaaaguca.............................................   1     1   7y1
....................................gcagcucccuUgcugcgaucuauugaaaguca.............................................   1     1   7y1
....................................gcagcucccucgcugAgaucuauugaaaguca.............................................   2     1   7y1
....................................gcagcuAccucgcugcgaucuauugaaaguca.............................................   3     1   7y1
.....................................cagcucccucgcugcgaucAauugaaagucag............................................   1     1   7y1
.....................................cagcucccucgcugcgaucuaGugaaagucag............................................   1     1   7y1
.....................................cagcucccucgcugcgaucuauugaaagucag............................................   188   0   7y1
.....................................cagcucccucgcugcgaAcuauugaaagucag............................................   1     1   7y1
.....................................cagcAcccucgcugcgaucuauugaaagucag............................................   2     1   7y1
.....................................cagcucccucgcugcgaucuauugaaagAcag............................................   1     1   7y1
.....................................cagcuAccucgcugcgaucuauugaaagucag............................................   1     1   7y1
.....................................cagcucccucgcugAgaucuauugaaagucag............................................   1     1   7y1
.....................................cagUucccucgcugcgaucuauugaaagucag............................................   1     1   7y1
.....................................cagcucccuAgcugcgaucuauugaaagucag............................................   1     1   7y1
.....................................cagcucccucAcugcgaucuauugaaagucag............................................   1     1   7y1
.....................................cagcuccAucgcugcgaucuauugaaagucag............................................   2     1   7y1
.....................................Aagcucccucgcugcgaucuauugaaagucag............................................   2     1   7y1
.....................................cagcuUccucgcugcgaucuauugaaagucag............................................   1     1   7y1
......................................agcucccucgcugcNaucu........................................................   1     1   7y1
......................................agcucccucgcugcgUucu........................................................   1     1   7y1
......................................agcucccucgcugcgaucG........................................................   1     1   7y1
......................................agcuccAucgcugcgaucu........................................................   1     1   7y1
......................................agcucccucgcugcgaucu........................................................   330   0   7y1
......................................agcuAccucgcugcgaucu........................................................   1     1   7y1
......................................agAucccucgcugcgaucu........................................................   1     1   7y1
......................................agcucccuAgcugcgaucu........................................................   1     1   7y1
```

Star **Mature**

```
gaccugcuucugggucgggguuucguacguagcagagcagcucccucgcugcgaucuauugaaagucagcccucgacacaaggguuuguccgcgcgcgcgcgcgcgcgcgugcgu
......................................agcucccucgcugAgaucu..........................................................  1     1  7y1
......................................Cgcucccucgcugcgaucu..........................................................  2     1  7y1
......................................agcucAcucgcugcgaucu..........................................................  1     1  7y1
......................................Cgcucccucgcugcgaucuauugaaaguc................................................  1     1  7y1
......................................agcucccucgcAgcgaucuauugaaaguc................................................  1     1  7y1
......................................agcucccucgcugcgaucuauugaaaguc................................................  185   0  7y1
......................................agcucAcucgcugcgaucuauugaaaguc................................................  2     1  7y1
......................................agcCcccucgcugcgaucuauugaaaguc................................................  1     1  7y1
......................................agcuAccucgcugcgaucuauugaaaguc................................................  2     1  7y1
......................................agcuccAucgcugcgaucuauugaaaguc................................................  1     1  7y1
......................................agcucccucgcugAgaucuauugaaaguc................................................  1     1  7y1
......................................agcucccucgcugcgaucuauugaaagucag..............................................  1     0  7y1
.......................................gcucccucgcugcgaucG..........................................................  1     1  7y1
.......................................Ccucccucgcugcgaucu..........................................................  1     1  7y1
.......................................gcucccucgcugcgaucu..........................................................  194   0  7y1
.......................................gcucccAcgcugcgaucu..........................................................  1     1  7y1
.......................................gcucccucUcugcgaucu..........................................................  1     1  7y1
.......................................gcucccucgAugcgaucu..........................................................  1     1  7y1
.......................................gcuAccucgcugcgaucu..........................................................  1     1  7y1
.......................................gcucAcucgcugcgaucuauug......................................................  3     1  7y1
.......................................gcuAccucgcugcgaucuauug......................................................  1     1  7y1
.......................................gcuccAucgcugcgaucuauug......................................................  2     1  7y1
.......................................Ccucccucgcugcgaucuauug......................................................  1     1  7y1
.......................................gcucccucgcugcgaucuauAg......................................................  1     1  7y1
.......................................gcucccucgcugcgaucuauug......................................................  515   0  7y1
.......................................gcucccucgcugcgaucAauug......................................................  1     1  7y1
.......................................gcucUcucgcugcgaucuauug......................................................  1     1  7y1
.......................................gcucccuAgcugcgaucuauug......................................................  1     1  7y1
.......................................gcucccucgcugcgaucuauCg......................................................  1     1  7y1
.......................................gcucccucgcugcgaucuauuU......................................................  1     1  7y1
.......................................gcucccucgcugcgUucuauug......................................................  1     1  7y1
.......................................gcucccucgcugcgaucuauugaGag..................................................  1     1  7y1
.......................................gcucccucCcugcgaucuauugaaag..................................................  1     1  7y1
.......................................gcucccucgcugcgaucuUuugaaag..................................................  1     1  7y1
.......................................gcucccucgcugcgaucuauugaaGg..................................................  1     1  7y1
.......................................gcucAcucgcugcgaucuauugaaag..................................................  2     1  7y1
.......................................gcucccucgcugcgaucuauGgaaag..................................................  1     1  7y1
.......................................gcucccucgcugcgauAuauugaaag..................................................  1     1  7y1
.......................................gcucccucgcugcUaucuauugaaag..................................................  1     1  7y1
.......................................gcuUccucgcugcgaucuauugaaag..................................................  1     1  7y1
.......................................gcucccucAcugcgaucuauugaaag..................................................  1     1  7y1
.......................................gcucccucgcGgcgaucuauugaaag..................................................  1     1  7y1
.......................................gcucccucgcugcgaucuauugGaag..................................................  1     1  7y1
.......................................gcucccucgcuCcgaucuauugaaag..................................................  1     1  7y1
.......................................gcuAccucgcugcgaucuauugaaag..................................................  2     1  7y1
.......................................gcucccucgcugAgaucuauugaaag..................................................  1     1  7y1
.......................................gNucccucgcugcgaucuauugaaag..................................................  1     1  7y1
.......................................gcucccuAgcugcgaucuauugaaag..................................................  1     1  7y1
.......................................gcucccucgcugcgaucuauugaaaC..................................................  2     1  7y1
.......................................gcucccucgAugcgaucuauugaaag..................................................  1     1  7y1
.......................................gcucccucgcugcgaucuauugaaag..................................................  1199  0  7y1
.......................................Ncucccucgcugcgaucuauugaaag..................................................  1     1  7y1
.......................................gcuccAucgcugcgaucuauugaaag..................................................  1     1  7y1
.......................................gcucccucgcAgcgaucuauugaaag..................................................  1     1  7y1
.......................................gcucccAcgcugcgaucuauugaaag..................................................  2     1  7y1
.......................................Ccucccucgcugcgaucuauugaaag..................................................  2     1  7y1
.......................................gAucccucgcugcgaucuauugaaag..................................................  3     1  7y1
.......................................gcucccucgcugcgaAcuauugaaagu.................................................  1     1  7y1
.......................................gcucccucgcCgcgaucuauugaaagu.................................................  1     1  7y1
.......................................gcucccucgcAgcgaucuauugaaagu.................................................  1     1  7y1
.......................................gcucAcucgcugcgaucuauugaaagu.................................................  2     1  7y1
.......................................gcucccucgcugcgaucCauugaaagu.................................................  1     1  7y1
.......................................gcucccucgcuCcgaucuauugaaagu.................................................  1     1  7y1
.......................................gcucccucgcugcgaucAauugaaagu.................................................  1     1  7y1
.......................................gcuccAucgcugcgaucuauugaaagu.................................................  1     1  7y1
.......................................gcucccucgcugcgaucuauAgaaagu.................................................  1     1  7y1
.......................................gcucccucgcugcgaucuauugaaaUu.................................................  1     1  7y1
.......................................gcucccucgcugcgaucuauugaaagu.................................................  906   0  7y1
.......................................gcuAccucgcugcgaucuauugaaagu.................................................  1     1  7y1
.......................................gcucccucgcugcUaucuauugaaagu.................................................  1     1  7y1
```

Star **Mature**

gaccugcuucugggucgggguuucguacguagcagagcagcucccucgcugcgaucuauugaaagucagcccucgacacaaggguuuguccgcgcgcgcgcgcgcgcgugcgu			
....gcucccucgcugcgauGuauugaaagu...	1	1	7y1
....gcucccuAgcugcgaucuauugaaagu...	2	1	7y1
....gcucccucgcugcgaucuauugaaagA...	1	1	7y1
....Ccucccucgcugcgaucuauugaaagu...	2	1	7y1
....Ucucccucgcugcgaucuauugaaagu...	1	1	7y1
....gcucccucgcugcgaucuaGugaaagu...	1	1	7y1
....gcucccucgcugcgaucuauugaaagG...	1	1	7y1
....gcucccucgAugcgaucuauugaaagu...	2	1	7y1
....gcucccucgcugcgaucuauugaaaguU..	1	1	7y1
....gcuGccucgcugcgaucuauugaaaguc..	1	1	7y1
....gcucccucgcugcgaucuauugaaaguc..	229	0	7y1
....gcucccucgcugAgaucuauugaaaguc..	1	1	7y1
....gcucAcucgcugcgaucuauugaaaguc..	1	1	7y1
....gcuccAucgcugcgaucuauugaaaguc..	1	1	7y1
....Ucucccucgcugcgaucuauugaaaguc..	1	1	7y1
....cucccucgcugcgaucuaAugaa..	1	1	7y1
....cucccAcgcugcgaucuauugaa..	1	1	7y1
....cucccucgcugAgaucuauugaa..	2	1	7y1
....cucccucgcugcgauAuauugaa..	1	1	7y1
....cucccucgcAgcgaucuauugaa..	1	1	7y1
....cucccucgcugcgaAcuauugaa..	1	1	7y1
....cucccucgcugcCaucuauugaa..	1	1	7y1
....cucccucgcugcgaucuauugaa..	144	0	7y1
....cucccucgcugcgaucuauugaaa...	244	0	7y1
....cucccucgcugcgaucuauGgaaa...	1	1	7y1
....cucccucgcugAgaucuauugaaa...	1	1	7y1
....cuAccucgcugcgaucuauugaaa...	3	1	7y1
....Aucccucgcugcgaucuauugaaa...	2	1	7y1
....cucccucgcuCcgaucuauugaaa...	1	1	7y1
....cucccuAgcugcgaucuauugaaag..	1	1	7y1
....cucccucgcuCcgaucuauugaaag..	1	1	7y1
....cucccucgcuUcgaucuauugaaag..	1	1	7y1
....cuccAucgcugcgaucuauugaaag..	1	1	7y1
....cucAcucgcugcgaucuauugaaag..	1	1	7y1
....cucccucgcugcgaucuauugaaag..	185	0	7y1
....cucccucgcuAcgaucuauugaaagu...	1	1	7y1
....Aucccucgcugcgaucuauugaaagu...	6	1	7y1
....cucccucgAugcgaucuauugaaagu...	2	1	7y1
....cucAcucgcugcgaucuauugaaagu...	1	1	7y1
....cucccucgcugcgaucuauAgaaagu...	1	1	7y1
....cucccucgcuCcgaucuauugaaagu...	2	1	7y1
....cucccucCcugcgaucuauugaaagu...	2	1	7y1
....cucccucgcAgcgaucuauugaaagu...	2	1	7y1
....cucccucgcugcUaucuauugaaagu...	1	1	7y1
....cucccucgcugcgaucuauugaaaUu...	1	1	7y1
....cucccucgcugcgaucuauugaaagA...	5	1	7y1
....cucccucUcugcgaucuauugaaagu...	3	1	7y1
....cucccucgcugcgaucuaAugaaagu...	1	1	7y1
....cuccUucgcugcgaucuauugaaagu...	1	1	7y1
....cucccucgcugcgCucuauugaaagu...	1	1	7y1
....cucccucgcugcgaucuauugaaagG...	1	1	7y1
....cuAccucgcugcgaucuauugaaagu...	9	1	7y1
....cucccucgcugAgaucuauugaaagu...	2	1	7y1
....cucccuAgcugcgaucuauugaaagu...	4	1	7y1
....cucccucgcugcgaucuauugGaagu...	1	1	7y1
....cucccucgcugcgaucuauuAaaagu...	1	1	7y1
....cAcccucgcugcgaucuauugaaagu...	7	1	7y1
....cucccucgcugcgaucuauugaUagu...	1	1	7y1
....cucccucgcugcgaucAauugaaagu...	2	1	7y1
....cucccucgcugcgaucuauugaaagu...	1893	0	7y1
....cucccucgcugcgaucuauugaaaCu...	1	1	7y1
....cucccAcgcugcgaucuauugaaagu...	5	1	7y1
....cucccucgcugcgaucuauugaUaguc..	1	1	7y1
....cucccucgcugcUaucuauugaaaguc..	3	1	7y1
....cucccucgcugcgaucuauAgaaaguc..	8	1	7y1
....cucccucgcugcgaucuauuCaaaguc..	1	1	7y1
....cucccucgcuCcgaucuauugaaaguc..	3	1	7y1
....cuAccucgcugcgaucuauugaaaguc..	10	1	7y1
....cucccucgcugcgaAcuauugaaaguc..	3	1	7y1
....Nucccucgcugcgaucuauugaaaguc..	3	1	7y1

Star **Mature**

```
gaccugcuucugggucgggguuucguacguagcagagcagcucccucgcugcgaucuauugaaagucagcccucgacacaaggguuuguccgcgcgcgcgcgcgcgcgcgugcgu
........................................cucccucgcugcgaucuauugaaaguA................................................    4      1    7y1
........................................cuccccuUgcugcgaucuauugaaaguc...............................................    1      1    7y1
........................................cuGccucgcugcgaucuauugaaaguc................................................    1      1    7y1
........................................cucUcucgcugcgaucuauugaaaguc................................................    2      1    7y1
........................................cucccucgcugAgaucuauugaaaguc................................................    12     1    7y1
........................................cucAcucgcugcgaucuauugaaaguc................................................    5      1    7y1
........................................cucccucgcugcgaucuauugGaaguc................................................    2      1    7y1
........................................cucccucgAugcgaucuauugaaaguc................................................    1      1    7y1
........................................cucccucgcuUcgaucuauugaaaguc................................................    6      1    7y1
........................................cucccucgcugcgaGcuauugaaaguc................................................    2      1    7y1
........................................cucccucUcugcgaucuauugaaaguc................................................    7      1    7y1
........................................cucccucgcugcgaucuaCugaaaguc................................................    1      1    7y1
........................................cucccucgcugcgaucuauugaaagAc................................................    3      1    7y1
........................................cucccucgcugcgaucuauugaaaAuc................................................    1      1    7y1
........................................cucccucgcCgcgaucuauugaaaguc................................................    1      1    7y1
........................................cAcccucgcugcgaucuauugaaaguc................................................    18     1    7y1
........................................cucccucgcugcgauAuauugaaaguc................................................    3      1    7y1
........................................cucccucgcugcgaucuaGugaaaguc................................................    1      1    7y1
........................................cucccucgcugcgaucuauuAaaaguc................................................    1      1    7y1
........................................cucccAcgcugcgaucuauugaaaguc................................................    9      1    7y1
........................................cucccucgGugcgaucuauugaaaguc................................................    1      1    7y1
........................................cucccucgcAgcgaucuauugaaaguc................................................    3      1    7y1
........................................cucccucgcugcgaucuaAugaaaguc................................................    7      1    7y1
........................................cucccucgcugcgaucuauugaaaCuc................................................    1      1    7y1
........................................cucccucgcugcgaucuauuUaaaguc................................................    2      1    7y1
........................................cucccucgcugcgaucuauugaaaguc................................................    4185   0    7y1
........................................cucccucgcuAcgaucuauugaaaguc................................................    4      1    7y1
........................................cucccucgcugcgaucuauugaaaguG................................................    1      1    7y1
........................................cucccucCcugcgaucuauugaaaguc................................................    4      1    7y1
........................................Aucccucgcugcgaucuauugaaaguc................................................    8      1    7y1
........................................cucccucgcugcgCucuauugaaaguc................................................    1      1    7y1
........................................cucccucgcugcgaucuGuugaaaguc................................................    1      1    7y1
........................................cucccucgcugcgaucuauugaaaUuc................................................    1      1    7y1
........................................cucccuAgcugcgaucuauugaaaguc................................................    6      1    7y1
........................................cuccAucgcugcgaucuauugaaaguc................................................    2      1    7y1
........................................cucccucgcugcgaucuauuCaaaguca...............................................    1      1    7y1
........................................cucccucgcugcgaucuauAgaaaguca...............................................    3      1    7y1
........................................cucccucgcugcgaucAauugaaaguca...............................................    1      1    7y1
........................................cucccucgcAgcgaucuauugaaaguca...............................................    5      1    7y1
........................................cucccAcgcugcgaucuauugaaaguca...............................................    4      1    7y1
........................................cucccucgcugcgaucuauugaaaguca...............................................    1280   0    7y1
........................................cucccucgcugcgaucuauugaGaguca...............................................    1      1    7y1
........................................cAcccucgcugcgaucuauugaaaguca...............................................    1      1    7y1
........................................cuAccucgcugcgaucuauugaaaguca...............................................    4      1    7y1
........................................cucccuAgcugcgaucuauugaaaguca...............................................    1      1    7y1
........................................cucccucgcugcgaucuauugaaagucG...............................................    2      1    7y1
........................................cucAcucgcugcgaucuauugaaaguca...............................................    1      1    7y1
........................................Aucccucgcugcgaucuauugaaaguca...............................................    3      1    7y1
........................................cucccucgcugcgaucuauugaaaguGa...............................................    1      1    7y1
........................................cucccucgcuCcgaucuauugaaaguca...............................................    1      1    7y1
........................................cucccucgcugAgaucuauugaaaguca...............................................    2      1    7y1
........................................cucccucgcugcgauAuauugaaaguca...............................................    2      1    7y1
........................................cuAccucgcugcgaucuauugaaagucag..............................................    2      1    7y1
........................................cucccAcgcugcgaucuauugaaagucag..............................................    1      1    7y1
........................................cucccucgcAgcgaucuauugaaagucag..............................................    1      1    7y1
........................................Aucccucgcugcgaucuauugaaagucag..............................................    1      1    7y1
........................................cucccuAgcugcgaucuauugaaagucag..............................................    3      1    7y1
........................................cucAcucgcugcgaucuauugaaagucag..............................................    1      1    7y1
........................................cucccucgcugcgaucuauugaaaCucag..............................................    1      1    7y1
........................................Gucccucgcugcgaucuauugaaagucag..............................................    1      1    7y1
........................................cucccucgcugcgaucuauugaaagGcag..............................................    1      1    7y1
........................................cucccucgcugAgaucuauugaaagucag..............................................    4      1    7y1
........................................cucccucgcugcgaucuauugaaagucag..............................................    378    0    7y1
........................................cucccucgcugcgaucuauAgaaagucag..............................................    1      1    7y1
........................................cuccGucgcugcgaucuauugaaagucagcc............................................    1      1    7y1
........................................cucccucgAugcgaucuauugaaagucagcc............................................    1      1    7y1
........................................cucccucgcugcgaucuauugaaaguAagcc............................................    1      1    7y1
........................................Nucccucgcugcgaucuauugaaagucagcc............................................    1      1    7y1
........................................cuAccucgcugcgaucuauugaaagucagcc............................................    1      1    7y1
........................................cucccucgcugcgaucuauugaaagAcagcc............................................    1      1    7y1
```

Star **Mature**

```
gaccugcuucugggucggggguuucguacguagcagagcagcucccucgcugcgaucuauugaaagucagcccucgacacaaggguuuguccgcgcgcgcgcgcgcgcgugcgu
........................................cucccucgcugcgaucuauugaaagucagcc..........................................  172  0  7y1
.........................................ucccucgcugcUaucuau......................................................  1  1  7y1
.........................................ucccucgcugcgaAcuau......................................................  1  1  7y1
.........................................Acccucgcugcgaucuau......................................................  1  1  7y1
.........................................ucccucgcugcgaCcuau......................................................  1  1  7y1
.........................................ucccucgcugcgaucuau......................................................  200  0  7y1
.........................................Ncccucgcugcgaucuau......................................................  1  1  7y1
.........................................ucccucgcugcgaucuaG......................................................  1  1  7y1
.........................................uAccucgcugcgaucuauu.....................................................  1  1  7y1
.........................................ucccucgcugcgaucuauu.....................................................  189  0  7y1
.........................................uUccucgcugcgaucuauu.....................................................  1  1  7y1
.........................................Acccucgcugcgaucuauu.....................................................  1  1  7y1
.........................................ucAcucgcugcgaucuauu.....................................................  1  1  7y1
.........................................Gcccucgcugcgaucuauu.....................................................  1  1  7y1
.........................................ucccucgcugcUaucuauu.....................................................  2  1  7y1
.........................................ucccucgcugcgaAcuauug....................................................  1  1  7y1
.........................................ucccucgcugcUaucuauug....................................................  1  1  7y1
.........................................ucccucgcugcgaucuauug....................................................  266  0  7y1
.........................................Acccucgcugcgaucuauug....................................................  4  1  7y1
.........................................ucccucgcugGgaucuauug....................................................  1  1  7y1
.........................................ucccuAgcugcgaucuauug....................................................  1  1  7y1
.........................................Acccucgcugcgaucuauugaaa.................................................  2  1  7y1
.........................................ucccucgcugcgaucuauugaaa.................................................  212  0  7y1
.........................................ucccucgcugcgaucuauuUaaa.................................................  1  1  7y1
.........................................ucccucUcugcgaucuauugaaa.................................................  1  1  7y1
.........................................Gcccucgcugcgaucuauugaaa.................................................  1  1  7y1
.........................................Ccccucgcugcgaucuauugaaa.................................................  1  1  7y1
.........................................ucccuAgcugcgaucuauugaaag................................................  1  1  7y1
.........................................ucccucgcugcgauAuauugaaag................................................  1  1  7y1
.........................................ucccucgcugcgaucAauugaaag................................................  1  1  7y1
.........................................Acccucgcugcgaucuauugaaag................................................  7  1  7y1
.........................................ucccucgcugcgaucuauAgaaag................................................  1  1  7y1
.........................................ucccucgcugcgauUuauugaaag................................................  1  1  7y1
.........................................ucccucgcugcgaucuauuAaaag................................................  1  1  7y1
.........................................ucccucgcuUcgaucuauugaaag................................................  1  1  7y1
.........................................ucAcucgcugcgaucuauugaaag................................................  3  1  7y1
.........................................Ncccucgcugcgaucuauugaaag................................................  3  1  7y1
.........................................Gcccucgcugcgaucuauugaaag................................................  1  1  7y1
.........................................uAccucgcugcgaucuauugaaag................................................  2  1  7y1
.........................................ucccucgcugcgaucuauugCaag................................................  1  1  7y1
.........................................ucccucgcugcgaucuauugaaag................................................  935  0  7y1
.........................................ucccucgcugcgaucuauugGaag................................................  1  1  7y1
.........................................ucccucgcugAgaucuauugaaag................................................  1  1  7y1
.........................................ucccucUcugcgaucuauugaaag................................................  2  1  7y1
.........................................ucccucgcAgcgaucuauugaaag................................................  2  1  7y1
.........................................ucccucgcuCcgaucuauugaaag................................................  3  1  7y1
.........................................ucccucgcugcgaucuauugaaagu...............................................  682  0  7y1
.........................................ucccucgcAgcgaucuauugaaagu...............................................  2  1  7y1
.........................................ucccucgcugAgaucuauugaaagu...............................................  4  1  7y1
.........................................Acccucgcugcgaucuauugaaagu...............................................  12  1  7y1
.........................................ucccucUcugcgaucuauugaaagu...............................................  1  1  7y1
.........................................uccAucgcugcgaucuauugaaagu...............................................  1  1  7y1
.........................................ucAcucgcugcgaucuauugaaagu...............................................  1  1  7y1
.........................................ucccucgcugcgauAuauugaaagu...............................................  2  1  7y1
.........................................ucccucgcugcUaucuauugaaagu...............................................  1  1  7y1
.........................................ucccucgcugcgaucAauugaaagu...............................................  1  1  7y1
.........................................ucccucgcugcgaucuauugaaagA...............................................  1  1  7y1
.........................................Gcccucgcugcgaucuauugaaagu...............................................  1  1  7y1
.........................................uAccucgcugcgaucuauugaaagu...............................................  1  1  7y1
.........................................ucccucgcugcgaucuaAugaaagu...............................................  1  1  7y1
.........................................ucccucgcuUcgaucuauugaaagu...............................................  1  1  7y1
.........................................Gcccucgcugcgaucuauugaaaguc..............................................  2  1  7y1
.........................................ucccucgcugcgaucuauugaaaAuc..............................................  1  1  7y1
.........................................ucccucgcugcgaucuaAugaaaguc..............................................  1  1  7y1
.........................................ucccucgcuCcgaucuauugaaaguc..............................................  2  1  7y1
.........................................ucccucgcugcgaucuauugaaaCuc..............................................  3  1  7y1
.........................................ucccucgcugcgaucAauugaaaguc..............................................  3  1  7y1
.........................................ucccucgcAgcgaucuauugaaaguc..............................................  4  1  7y1
.........................................ucccucgcugcgaucuauAgaaaguc..............................................  2  1  7y1
.........................................ucAcucgcugcgaucuauugaaaguc..............................................  10  1  7y1
```

Star **Mature**

```
gaccugcuucugggucgggguuucguacguagcagagcagcucccucgcugcgaucuauugaaagucagcccucgacacaaggguuuguccgcgcgcgcgcgcgcgugcgu
.........................................ucccucgcugcgaucuauugGaaguc............................................ 1 1 7y1
.........................................uAccucgcugcgaucuauugaaaguc............................................ 3 1 7y1
.........................................ucccucgcugcUaucuauugaaaguc............................................ 1 1 7y1
.........................................ucccucgAugcgaucuauugaaaguc............................................ 3 1 7y1
.........................................ucccucgcugcgaucuauugaaaguc............................................ 2692 0 7y1
.........................................ucccuAgcugcgaucuauugaaaguc............................................ 7 1 7y1
.........................................ucccucgcugcgaucuauugaaaguA............................................ 1 1 7y1
.........................................ucccucgcGgcgaucuauugaaaguc............................................ 1 1 7y1
.........................................ucccucgcuUcgaucuauugaaaguc............................................ 2 1 7y1
.........................................ucccucAcugcgaucuauugaaaguc............................................ 1 1 7y1
.........................................ucccucgcugcgaucuGuugaaaguc............................................ 2 1 7y1
.........................................Acccucgcugcgaucuauugaaaguc............................................ 38 1 7y1
.........................................ucccucgcugAgaucuauugaaaguc............................................ 2 1 7y1
.........................................ucccAcgcugcgaucuauugaaaguc............................................ 1 1 7y1
.........................................ucccucgcugcCaucuauugaaaguc............................................ 3 1 7y1
.........................................ucccucgcugcgaucuauugaaagAc............................................ 2 1 7y1
.........................................ucccucUcugcgaucuauugaaaguc............................................ 2 1 7y1
.........................................ucccucgcugcgauAuauugaaaguc............................................ 4 1 7y1
.........................................uccAucgcugcgaucuauugaaaguc............................................ 2 1 7y1
.........................................Ncccucgcugcgaucuauugaaaguc............................................ 1 1 7y1
.........................................ucccucgGugcgaucuauugaaaguca........................................... 1 1 7y1
.........................................ucccucgcugcgaucuauugaaaguca........................................... 586 0 7y1
.........................................Ncccucgcugcgaucuauugaaaguca........................................... 2 1 7y1
.........................................ucccucgcugcgaucCauugaaaguca........................................... 1 1 7y1
.........................................ucccucgcugcgauAuauugaaaguca........................................... 1 1 7y1
.........................................Gcccucgcugcgaucuauugaaaguca........................................... 1 1 7y1
.........................................ucccucgcugAgaucuauugaaaguca........................................... 1 1 7y1
.........................................ucccucgcugcgaucuauugaaagucU........................................... 1 1 7y1
.........................................ucccucUcugcgaucuauugaaaguca........................................... 1 1 7y1
.........................................ucccucgcuAcgaucuauugaaaguca........................................... 1 1 7y1
.........................................ucccucgcuCcgaucuauugaaaguca........................................... 1 1 7y1
.........................................uccAucgcugcgaucuauugaaaguca........................................... 1 1 7y1
.........................................ucccucgcugcCaucuauugaaaguca........................................... 1 1 7y1
.........................................ucccucgAugcgaucuauugaaaguca........................................... 2 1 7y1
.........................................ucccucCcugcgaucuauugaaaguca........................................... 1 1 7y1
.........................................Acccucgcugcgaucuauugaaaguca........................................... 6 1 7y1
.........................................ucccucgcuUcgaucuauugaaagucag.......................................... 1 1 7y1
.........................................ucAcucgcugcgaucuauugaaagucag.......................................... 1 1 7y1
.........................................ucccucgcugcgaucuauAgaaagucag.......................................... 1 1 7y1
.........................................ucccucgcugcgaucuauugaaagucag.......................................... 159 0 7y1
.........................................Acccucgcugcgaucuauugaaagucag.......................................... 2 1 7y1
.........................................ucccucgcugcgUucuauugaaagucag.......................................... 1 1 7y1
.........................................ucccAcgcugcgaucuauugaaagucag.......................................... 1 1 7y1
..........................................cccucgcugcgaucuauu................................................... 172 0 7y1
..........................................cccucgcugcgaucuaAu................................................... 1 1 7y1
..........................................Gccucgcugcgaucuauu................................................... 1 1 7y1
..........................................ccAucgcugcgaucuauu................................................... 1 1 7y1
..........................................cAcucgcugcgaucuauu................................................... 1 1 7y1
..........................................cccucgcugcCaucuauu................................................... 2 1 7y1
..........................................cccucgcAgcgaucuauu................................................... 1 1 7y1
..........................................cccucgcugcgaucuauuga................................................. 157 0 7y1
..........................................Accucgcugcgaucuauuga................................................. 1 1 7y1
..........................................ccAucgcugcgaucuauuga................................................. 1 1 7y1
..........................................cccucgAugcgaucuauuga................................................. 1 1 7y1
..........................................cAcucgcugcgaucuauuga................................................. 2 1 7y1
..........................................cccucgcAgcgaucuauuga................................................. 1 1 7y1
..........................................cccucgcugcgaucuauugG................................................. 1 1 7y1
..........................................Nccucgcugcgaucuauuga................................................. 1 1 7y1
..........................................cccucgcugcgaucuauuAaa................................................ 1 1 7y1
..........................................cAcucgcugcgaucuauugaaa............................................... 3 1 7y1
..........................................cccucgAugcgaucuauugaaa............................................... 3 1 7y1
..........................................cccucgcugcUaucuauugaaa............................................... 1 1 7y1
..........................................cccucgcugcgaucuaAugaaa............................................... 3 1 7y1
..........................................cccucgcugcgaucuauAgaaa............................................... 1 1 7y1
..........................................cccucgcuUcgaucuauugaaa............................................... 2 1 7y1
..........................................cccucgcugcCaucuauugaaa............................................... 1 1 7y1
..........................................cUcucgcugcgaucuauugaaa............................................... 1 1 7y1
..........................................ccGucgcugcgaucuauugaaa............................................... 1 1 7y1
..........................................cccucgcugcgaucuauugaaa............................................... 1120 0 7y1
..........................................cccucgcugcgaAcuauugaaa............................................... 2 1 7y1
```

```
  Star                                                                     Mature
gaccugcuucugggucggggguucguacguagcagagcagcucccucgcugcgaucuauugaaagucagcccucgacacaaggguuuguccgcgcgcgcgcgcgcgcgugcgu
.........................................Accucgcugcgaucuauugaaa..................................................    3      1    7y1
.........................................cccucgcugAgaucuauugaaa..................................................    3      1    7y1
.........................................cccucgcugcgauUuauugaaa..................................................    1      1    7y1
.........................................cccucgcuCcgaucuauugaaa..................................................    2      1    7y1
.........................................cccucgcugcgaucuauuCaaa..................................................    1      1    7y1
.........................................cccuAgcugcgaucuauugaaa..................................................    1      1    7y1
.........................................cccAcgcugcgaucuauugaaa..................................................    1      1    7y1
.........................................cccucgcugcgauAuauugaaa..................................................    1      1    7y1
.........................................cccucgcugcgaucGauugaaa..................................................    1      1    7y1
.........................................Nccucgcugcgaucuauugaaa..................................................    1      1    7y1
.........................................cccucgcCgcgaucuauugaaa..................................................    1      1    7y1
.........................................cccucgcAgcgaucuauugaaa..................................................    3      1    7y1
.........................................cccucgcugcgaucuaCugaaag.................................................    1      1    7y1
.........................................cccucgcAgcgaucuauugaaag.................................................    1      1    7y1
.........................................cccucgcugcgaucuauAgaaag.................................................    1      1    7y1
.........................................cccucgcuUcgaucuauugaaag.................................................    1      1    7y1
.........................................Gccucgcugcgaucuauugaaag.................................................    1      1    7y1
.........................................cccAcgcugcgaucuauugaaag.................................................    2      1    7y1
.........................................cAcucgcugcgaucuauugaaag.................................................    6      1    7y1
.........................................cccucgcugcCaucuauugaaag.................................................    3      1    7y1
.........................................cccucgcugcgauAuauugaaag.................................................    1      1    7y1
.........................................cccucgcugAgaucuauugaaag.................................................    2      1    7y1
.........................................ccUucgcugcgaucuauugaaag.................................................    1      1    7y1
.........................................ccGucgcugcgaucuauugaaag.................................................    1      1    7y1
.........................................cccucgcugcgaucuaAugaaag.................................................    3      1    7y1
.........................................cccucCcugcgaucuauugaaag.................................................    1      1    7y1
.........................................Nccucgcugcgaucuauugaaag.................................................    1      1    7y1
.........................................cccucgcugcgaucuauugaaag.................................................    1154   0    7y1
.........................................cNcucgcugcgaucuauugaaag.................................................    1      1    7y1
.........................................cccucgcugcgaucuauuAaaag.................................................    1      1    7y1
.........................................Accucgcugcgaucuauugaaag.................................................    1      1    7y1
.........................................cccucgcugcgaucuauugUaag.................................................    1      1    7y1
.........................................cccucgGugcgaucuauugaaag.................................................    1      1    7y1
.........................................cccucgcugcgaucuauGgaaag.................................................    1      1    7y1
.........................................ccAucgcugcgaucuauugaaag.................................................    5      1    7y1
.........................................ccAucgcugcgaucuauugaaagu................................................    4      1    7y1
.........................................Accucgcugcgaucuauugaaagu................................................    2      1    7y1
.........................................cccucgAugcgaucuauugaaagu................................................    5      1    7y1
.........................................cccucgcugcgaucuauuCaaagu................................................    1      1    7y1
.........................................cccucgcugcgaucCauugaaagu................................................    1      1    7y1
.........................................cccucgcugcgauAuauugaaagu................................................    2      1    7y1
.........................................cccucgcugcgaucAauugaaagu................................................    1      1    7y1
.........................................cAcucgcugcgaucuauugaaagu................................................    7      1    7y1
.........................................cccucgcugAgaucuauugaaagu................................................    6      1    7y1
.........................................cccucgcugcCaucuauugaaagu................................................    1      1    7y1
.........................................cccucgcugcgaucuauugaaagu................................................    1043   0    7y1
.........................................cccucUcugcgaucuauugaaagu................................................    2      1    7y1
.........................................cccucgcugcgaucuauugaaagG................................................    11     1    7y1
.........................................cccAcgcugcgaucuauugaaagu................................................    1      1    7y1
.........................................cccucgcuCcgaucuauugaaagu................................................    1      1    7y1
.........................................cccucgcugcgaucuauugaaagA................................................    2      1    7y1
.........................................cccucgcugcgaAcuauugaaagu................................................    1      1    7y1
.........................................cccucgcugcgaucuauAgaaagu................................................    1      1    7y1
.........................................Nccucgcugcgaucuauugaaaguc...............................................    3      1    7y1
.........................................cccucgcugcgaucuauugaaaguA...............................................    1      1    7y1
.........................................cccucgcugcUaucuauugaaaguc...............................................    1      1    7y1
.........................................cccAcgcugcgaucuauugaaaguc...............................................    1      1    7y1
.........................................cccucgcuUcgaucuauugaaaguc...............................................    1      1    7y1
.........................................cccucgcugcgCucuauugaaaguc...............................................    1      1    7y1
.........................................cccucgcugcgaucuauugaGaguc...............................................    2      1    7y1
.........................................cccucgcugcgaucuaAugaaaguc...............................................    2      1    7y1
.........................................cccucgcugcgaucuauugaaaUuc...............................................    1      1    7y1
.........................................cccuAgcugcgaucuauugaaaguc...............................................    1      1    7y1
.........................................cccucgcugcgaucAauugaaaguc...............................................    3      1    7y1
.........................................cccucgcugcgaucuauugaaaCuc...............................................    1      1    7y1
.........................................cccucgcugAgaucuauugaaaguc...............................................    6      1    7y1
.........................................cNcucgcugcgaucuauugaaaguc...............................................    1      1    7y1
.........................................cccucgcCgcgaucuauugaaaguc...............................................    1      1    7y1
.........................................ccGucgcugcgaucuauugaaaguc...............................................    1      1    7y1
.........................................cccucgcugcgauAuauugaaaguc...............................................    2      1    7y1
```

Star Mature

```
gaccugcuucugggucgggguuucguacguagcagagcagcucccucgcugcgaucuauugaaagucagcccucgacacaaggguuuguccgcgcgcgcgcgcgcgugcgu
..........................................cccucgcugcgaAcuauugaaaguc............................................ 2 1 7y1
..........................................cccucgUugcgaucuauugaaaguc............................................ 1 1 7y1
..........................................cccucgcAgcgaucuauugaaaguc............................................ 1 1 7y1
..........................................cAcucgcugcgaucuauugaaaguc............................................ 7 1 7y1
..........................................Accucgcugcgaucuauugaaaguc............................................ 7 1 7y1
..........................................cccucgcuCcgaucuauugaaaguc............................................ 2 1 7y1
..........................................cccucUcugcgaucuauugaaaguc............................................ 1 1 7y1
..........................................cccucgcugcgaucuauugaaaguc............................................ 1855 0 7y1
..........................................cccucgcugcgaucuauugaaagAc............................................ 2 1 7y1
..........................................cccucgcugcgaucuauuUaaaguc............................................ 1 1 7y1
..........................................cccucgAugcgaucuauugaaaguc............................................ 8 1 7y1
..........................................ccAucgcugcgaucuauugaaaguc............................................ 8 1 7y1
..........................................cccucgcugcgaucuauAgaaaguc............................................ 1 1 7y1
..........................................Uccucgcugcgaucuauugaaaguc............................................ 2 1 7y1
..........................................cccucgcugcgaucuauuAaaaguc............................................ 1 1 7y1
..........................................cccucgcugcgaucuauCgaaaguc............................................ 1 1 7y1
..........................................cccucgcugGgaucuauugaaaguc............................................ 1 1 7y1
..........................................cccucgcugcgaucuauugaaaguAa........................................... 1 1 7y1
..........................................cccuAgcugcgaucuauugaaaguca........................................... 3 1 7y1
..........................................cccucgcugAgaucuauugaaaguca........................................... 2 1 7y1
..........................................ccAucgcugcgaucuauugaaaguca........................................... 2 1 7y1
..........................................cccuUgcugcgaucuauugaaaguca........................................... 2 1 7y1
..........................................Nccucgcugcgaucuauugaaaguca........................................... 3 1 7y1
..........................................cccucgcugcgaucuauugaaagAca........................................... 3 1 7y1
..........................................Accucgcugcgaucuauugaaaguca........................................... 5 1 7y1
..........................................cccucgcugcgauAuauugaaaguca........................................... 2 1 7y1
..........................................cccucUcugcgaucuauugaaaguca........................................... 2 1 7y1
..........................................cccucgcugcgaAcuauugaaaguca........................................... 1 1 7y1
..........................................cccucgcugcgaucuauugaaaguca........................................... 592 0 7y1
..........................................cccAcgcugcgaucuauugaaaguca........................................... 1 1 7y1
..........................................cccucAcugcgaucuauugaaaguca........................................... 1 1 7y1
..........................................cccucgcAgcgaucuauugaaaguca........................................... 2 1 7y1
..........................................cccucgcugcgaucuauugaaaUuca........................................... 1 1 7y1
..........................................cccucgcugcUaucuauugaaaguca........................................... 1 1 7y1
...........................................ccucgAugcgaucuauug.................................................. 1 1 7y1
...........................................cAucgcugcgaucuauug.................................................. 2 1 7y1
...........................................ccucgcCgcgaucuauug.................................................. 1 1 7y1
...........................................ccucgcugcgaucuauug.................................................. 209 0 7y1
...........................................ccucgcuUcgaucuauug.................................................. 1 1 7y1
...........................................ccucgcugcgaucuauuC.................................................. 1 1 7y1
...........................................ccucgcGgcgaucuauug.................................................. 1 1 7y1
...........................................Ncucgcugcgaucuauug.................................................. 2 1 7y1
...........................................ccucgcugcgaucGauugaa................................................ 1 1 7y1
...........................................ccucgcugcUaucuauugaa................................................ 2 1 7y1
...........................................ccucgcugcgaucuauAgaa................................................ 1 1 7y1
...........................................ccucgcugcgaucuauugaa................................................ 602 0 7y1
...........................................Ncucgcugcgaucuauugaa................................................ 1 1 7y1
...........................................ccuAgcugcgaucuauugaa................................................ 1 1 7y1
...........................................cAucgcugcgaucuauugaa................................................ 3 1 7y1
...........................................ccAcgcugcgaucuauugaa................................................ 1 1 7y1
...........................................ccucgcAgcgaucuauugaa................................................ 2 1 7y1
...........................................ccucCcugcgaucuauugaa................................................ 1 1 7y1
...........................................ccucgcugcgaucuauuCaa................................................ 1 1 7y1
...........................................ccucgcugcgaucuauuAaa................................................ 1 1 7y1
...........................................Acucgcugcgaucuauugaa................................................ 1 1 7y1
...........................................ccucgcugcgauAuauugaaa............................................... 4 1 7y1
...........................................Acucgcugcgaucuauugaaa............................................... 6 1 7y1
...........................................ccuAgcugcgaucuauugaaa............................................... 1 1 7y1
...........................................ccucgAugcgaucuauugaaa............................................... 6 1 7y1
...........................................ccucgcugcgaucuaAugaaa............................................... 1 1 7y1
...........................................ccucgcAgcgaucuauugaaa............................................... 9 1 7y1
...........................................ccucgcugcUaucuauugaaa............................................... 5 1 7y1
...........................................ccucgcugAgaucuauugaaa............................................... 1 1 7y1
...........................................cAucgcugcgaucuauugaaa............................................... 2 1 7y1
...........................................Gcucgcugcgaucuauugaaa............................................... 1 1 7y1
...........................................ccucgcugcgaucuauugaGa............................................... 1 1 7y1
...........................................ccucgcuUcgaucuauugaaa............................................... 1 1 7y1
...........................................ccucgcugcgaucAauugaaa............................................... 2 1 7y1
...........................................ccucgcugcgaucuauugaaa............................................... 1604 0 7y1
...........................................ccucgcugcgaucuauugaUa............................................... 1 1 7y1
```

Star Mature

```
gaccugcuucugggucgggguuucguacguagcagagcagcucccucgcugcgaucuauugaaagucagcccucgacacaaggguuuguccgcgcgcgcgcgcgcgcgugcgu
..........................................ccucgcugcgUucuauugaaa...............................................	1	1	7y1
..........................................ccucgcugAgaucuauugaaag..............................................	5	1	7y1
..........................................ccucgcugcgaucuauCgaaag..............................................	1	1	7y1
..........................................ccuGgcugcgaucuauugaaag..............................................	1	1	7y1
..........................................ccucgcugcCaucuauugaaag..............................................	2	1	7y1
..........................................Acucgcugcgaucuauugaaag..............................................	2	1	7y1
..........................................ccucgcugcgaAcuauugaaag..............................................	1	1	7y1
..........................................ccucgcugcgaucAauugaaag..............................................	1	1	7y1
..........................................ccucgcuCcgaucuauugaaag..............................................	1	1	7y1
..........................................cAucgcugcgaucuauugaaag..............................................	4	1	7y1
..........................................ccucgcugcgaucuauugaaag..............................................	1408	0	7y1
..........................................ccucgcugcgaucuauugaaaU..............................................	1	1	7y1
..........................................ccucgcugcgaucuauugaaGg..............................................	1	1	7y1
..........................................ccAcgcugcgaucuauugaaag..............................................	2	1	7y1
..........................................ccuAgcugcgaucuauugaaag..............................................	1	1	7y1
..........................................ccucUcugcgaucuauugaaag..............................................	1	1	7y1
..........................................ccucgAugcgaucuauugaaag..............................................	2	1	7y1
..........................................ccucgcugcgaucCauugaaag..............................................	1	1	7y1
..........................................ccucgcugcgauAuauugaaag..............................................	2	1	7y1
..........................................ccucgcugcgaucuaAugaaag..............................................	1	1	7y1
..........................................ccucCcugcgaucuauugaaag..............................................	2	1	7y1
..........................................ccucgcAgcgaucuauugaaag..............................................	3	1	7y1
..........................................Ncucgcugcgaucuauugaaag..............................................	1	1	7y1
..........................................Gcucgcugcgaucuauugaaag..............................................	2	1	7y1
..........................................ccucgcuUcgaucuauugaaag..............................................	1	1	7y1
..........................................ccucgcugcgaucuauAgaaag..............................................	2	1	7y1
..........................................ccucgcuAcgaucuauugaaag..............................................	1	1	7y1
..........................................ccucUcugcgaucuauugaaagu.............................................	1	1	7y1
..........................................ccucgcugcgaucuauugaaagu.............................................	1291	0	7y1
..........................................ccucgcugcgaucuauAgaaagu.............................................	1	1	7y1
..........................................ccuAgcugcgaucuauugaaagu.............................................	1	1	7y1
..........................................Gcucgcugcgaucuauugaaagu.............................................	1	1	7y1
..........................................ccucgcugcgaucuauugaaaUu.............................................	1	1	7y1
..........................................ccucgcuUcgaucuauugaaagu.............................................	1	1	7y1
..........................................ccucgcugcgaucuauugaaagG.............................................	1	1	7y1
..........................................ccucgcugcgaucuaAugaaagu.............................................	1	1	7y1
..........................................ccucgcugcgaucAauugaaagu.............................................	1	1	7y1
..........................................cUucgcugcgaucuauugaaagu.............................................	1	1	7y1
..........................................ccucgcugcUaucuauugaaagu.............................................	2	1	7y1
..........................................ccucgcCgcgaucuauugaaagu.............................................	1	1	7y1
..........................................ccAcgcugcgaucuauugaaagu.............................................	3	1	7y1
..........................................ccucgAugcgaucuauugaaagu.............................................	2	1	7y1
..........................................ccucgcugcgaAcuauugaaagu.............................................	1	1	7y1
..........................................ccucgcugAgaucuauugaaagu.............................................	6	1	7y1
..........................................ccucgcuCcgaucuauugaaagu.............................................	1	1	7y1
..........................................Ncucgcugcgaucuauugaaagu.............................................	1	1	7y1
..........................................ccucgcugcCaucuauugaaagu.............................................	1	1	7y1
..........................................ccucgcugcgauAuauugaaagu.............................................	1	1	7y1
..........................................ccucgcugcgaucuauugaGagu.............................................	1	1	7y1
..........................................Acucgcugcgaucuauugaaagu.............................................	2	1	7y1
..........................................ccucgcAgcgaucuauugaaagu.............................................	1	1	7y1
..........................................cAucgcugcgaucuauugaaagu.............................................	2	1	7y1
..........................................ccucAcugcgaucuauugaaagu.............................................	2	1	7y1
..........................................ccucgcugcgaucuauugaaagA.............................................	3	1	7y1
..........................................ccucgcugcgauGuauugaaagu.............................................	1	1	7y1
..........................................ccuGgcugcgaucuauugaaagu.............................................	1	1	7y1
..........................................ccucgcugcgaucuauugaaagAc............................................	4	1	7y1
..........................................ccGcgcugcgaucuauugaaaguc............................................	1	1	7y1
..........................................ccucgcugcgaucuUuugaaaguc............................................	1	1	7y1
..........................................ccuAgcugcgaucuauugaaaguc............................................	4	1	7y1
..........................................ccucgcugcgaucuauAgaaaguc............................................	4	1	7y1
..........................................ccucgcugcgaucuauuCaaaguc............................................	1	1	7y1
..........................................ccucgcugAgaucuauugaaaguc............................................	9	1	7y1
..........................................ccucgcugcUaucuauugaaaguc............................................	2	1	7y1
..........................................ccucgcugcgaGcuauugaaaguc............................................	1	1	7y1
..........................................ccucgcugcgaucuauCgaaaguc............................................	3	1	7y1
..........................................ccucCcugcgaucuauugaaaguc............................................	2	1	7y1
..........................................ccucgcugcgaucuauugaaaguc............................................	4155	0	7y1
..........................................ccucgcuUcgaucuauugaaaguc............................................	5	1	7y1
..........................................ccucgNugcgaucuauugaaaguc............................................	1	1	7y1
```

```
 Star                                                                    Mature
gaccugcuucugggucgggguuucguacguagcagagcagcucccucgcugcgaucuauugaaagucagcccucgacacaaggguuuguccgcgcgcgcgcgcgcgcgugcgu
...........................................ccucgcugcgaucuaAugaaaguc................................................   3    1    7y1
...........................................ccAcgcugcgaucuauugaaaguc................................................   2    1    7y1
...........................................ccucgcugcgaucuauuUaaaguc................................................   1    1    7y1
...........................................ccucgcugcgaucCauugaaaguc................................................   1    1    7y1
...........................................ccucgcugcgauAuauugaaaguc................................................   7    1    7y1
...........................................ccucgcugcgUucuauugaaaguc................................................   1    1    7y1
...........................................ccucgcugcgaucuauugaGaguc................................................   1    1    7y1
...........................................ccucgcugcgaAcuauugaaaguc................................................   1    1    7y1
...........................................Gcucgcugcgaucuauugaaaguc................................................   2    1    7y1
...........................................ccucgcuCcgaucuauugaaaguc................................................   1    1    7y1
...........................................ccCcgcugcgaucuauugaaaguc................................................   1    1    7y1
...........................................Acucgcugcgaucuauugaaaguc................................................   7    1    7y1
...........................................ccucgcugcCaucuauugaaaguc................................................   4    1    7y1
...........................................ccucgcugcgaucuauugaaaCuc................................................   5    1    7y1
...........................................ccucgcugcgaucuauGgaaaguc................................................   1    1    7y1
...........................................ccucgcugcgaucuauugaaaUuc................................................   1    1    7y1
...........................................ccucgcugcgaucuauugGaaguc................................................   1    1    7y1
...........................................ccucgcAgcgaucuauugaaaguc................................................   16   1    7y1
...........................................ccucgcugcgaucAauugaaaguc................................................   3    1    7y1
...........................................ccucgcCgcgaucuauugaaaguc................................................   1    1    7y1
...........................................ccucgAugcgaucuauugaaaguc................................................   6    1    7y1
...........................................cAucgcugcgaucuauugaaaguc................................................   9    1    7y1
...........................................ccucgcugcgaucuauugaaGguc................................................   1    1    7y1
...........................................ccucgcugcgGucuauugaaaguc................................................   1    1    7y1
...........................................ccucUcugcgaucuauugaaaguc................................................   3    1    7y1
...........................................Ncucgcugcgaucuauugaaaguc................................................   1    1    7y1
...........................................ccucgcugcgaucuauugaaaguA................................................   2    1    7y1
...........................................ccucgcugcgaucuauuAaaaguc................................................   1    1    7y1
...........................................cAucgcugcgaucuauugaaaguca...............................................   2    1    7y1
...........................................Ncucgcugcgaucuauugaaaguca...............................................   1    1    7y1
...........................................ccucgcAgcgaucuauugaaaguca...............................................   1    1    7y1
...........................................ccucgcugAgaucuauugaaaguca...............................................   2    1    7y1
...........................................Acucgcugcgaucuauugaaaguca...............................................   3    1    7y1
...........................................Gcucgcugcgaucuauugaaaguca...............................................   1    1    7y1
...........................................ccucgcugcgaucuauugaaagAca...............................................   1    1    7y1
...........................................ccucgcugcgaucuauugaaaCuca...............................................   1    1    7y1
...........................................ccucgcugcgaucuauugaaaAuca...............................................   1    1    7y1
...........................................ccucgcugcgaucuauugaaaguca...............................................   761  0    7y1
...........................................ccucgcuUcgaucuauugaaaguca...............................................   1    1    7y1
...........................................ccucUcugcgaucuauugaaaguca...............................................   1    1    7y1
...........................................ccucgAugcgaucuauugaaaguca...............................................   1    1    7y1
...........................................ccucgcugcgauAuauugaaaguca...............................................   1    1    7y1
...........................................ccucgcugcgaucuaAugaaaguca...............................................   1    1    7y1
...........................................ccucgcugcgaucuauugaaaUuca...............................................   1    1    7y1
...........................................ccucgcugcgaucuauugaaagucG...............................................   1    1    7y1
...........................................Acucgcugcgaucuauugaaagucag..............................................   1    1    7y1
...........................................ccucgcuCcgaucuauugaaagucag..............................................   1    1    7y1
...........................................cAucgcugcgaucuauugaaagucag..............................................   1    1    7y1
...........................................ccucgcugcgaucuauAgaaagucag..............................................   1    1    7y1
...........................................ccucgcugcUaucuauugaaagucag..............................................   1    1    7y1
...........................................Ncucgcugcgaucuauugaaagucag..............................................   1    1    7y1
...........................................ccucgcugcgaucAauugaaagucag..............................................   1    1    7y1
...........................................ccucgcAgcgaucuauugaaagucag..............................................   2    1    7y1
...........................................ccucAcugcgaucuauugaaagucag..............................................   1    1    7y1
...........................................ccucgcugAgaucuauugaaagucag..............................................   1    1    7y1
...........................................ccucgcugcgaucuauugaaagucag..............................................   163  0    7y1
...........................................ccucgcugcgaucuauugaaagucagUcc...........................................   1    1    7y1
...........................................ccucgcugcgaucuauugaaagucagUccu..........................................   253  1    7y1
............................................cucgAugcgaucuauugaaa...................................................   1    1    7y1
............................................cucgcugcgaucuauAgaaa...................................................   2    1    7y1
............................................cucgcugcgauAuauugaaa...................................................   1    1    7y1
............................................cucgcugcgaucuauugaaa...................................................   413  0    7y1
............................................cucUcugcgaucuauugaaa...................................................   2    1    7y1
............................................cucgcugAgaucuauugaaa...................................................   2    1    7y1
............................................cucgcugcUaucuauugaaa...................................................   1    1    7y1
............................................Aucgcugcgaucuauugaaa...................................................   2    1    7y1
............................................cuAgcugcgaucuauugaaa...................................................   3    1    7y1
............................................cucgcugcCaucuauugaaa...................................................   1    1    7y1
............................................cAcgcugcgaucuauugaaa...................................................   2    1    7y1
............................................cuUgcugcgaucuauugaaag..................................................   1    1    7y1
```

```
 Star                                                                  Mature
gaccugcuucugggucgggguuucguacguagcagagcagcucccucgcugcgaucuauugaaagucagcccucgacacaaggguuuguccgcgcgcgcgcgcgcgugcgu
............................................cucgcugcUaucuauugaaag..............................................    2     1    7y1
............................................cucgcugcgaucuauugUaag..............................................    1     1    7y1
............................................cAcgcugcgaucuauugaaag..............................................    12    1    7y1
............................................cucAcugcgaucuauugaaag..............................................    2     1    7y1
............................................Gucgcugcgaucuauugaaag..............................................    4     1    7y1
............................................cucgcugcgaucuauuUaaag..............................................    1     1    7y1
............................................cucgcAgcgaucuauugaaag..............................................    12    1    7y1
............................................cucgAugcgaucuauugaaag..............................................    3     1    7y1
............................................cuAgcugcgaucuauugaaag..............................................    10    1    7y1
............................................cucgcugcgaucuauAgaaag..............................................    2     1    7y1
............................................cucgcugcgaucAauugaaag..............................................    1     1    7y1
............................................cucgcugcgaucuauugaaaC..............................................    2     1    7y1
............................................cucgcugcgaucuauugaaag..............................................    2182  0    7y1
............................................cucgcugcgaucuauugGaag..............................................    1     1    7y1
............................................cucCcugcgaucuauugaaag..............................................    1     1    7y1
............................................cucgcugcgaucuaAugaaag..............................................    1     1    7y1
............................................cucgcugcgauUuauugaaag..............................................    1     1    7y1
............................................cucgcugcgaucuauugaGag..............................................    167   1    7y1
............................................cucgcugAgaucuauugaaag..............................................    8     1    7y1
............................................cucgcugcgaucuauugaaaU..............................................    2     1    7y1
............................................cucgcugcgaAcuauugaaag..............................................    2     1    7y1
............................................Aucgcugcgaucuauugaaag..............................................    2     1    7y1
............................................cucgcugcgauAuauugaaag..............................................    6     1    7y1
............................................cucgcuUcgaucuauugaaag..............................................    2     1    7y1
............................................cucgcugcgaucuauGgaaag..............................................    1     1    7y1
............................................cucgcugGgaucuauugaaag..............................................    2     1    7y1
............................................cucgcuCcgaucuauugaaagu.............................................    1     1    7y1
............................................cucgcCgcgaucuauugaaagu.............................................    1     1    7y1
............................................cucgcugcCaucuauugaaagu.............................................    1     1    7y1
............................................cucgcugcgauAuauugaaagu.............................................    1     1    7y1
............................................cAcgcugcgaucuauugaaagu.............................................    7     1    7y1
............................................cucgcugcgaucuauugaaagu.............................................    1949  0    7y1
............................................cucUcugcgaucuauugaaagu.............................................    1     1    7y1
............................................cucgcuUcgaucuauugaaagu.............................................    1     1    7y1
............................................cucgcNgcgaucuauugaaagu.............................................    1     1    7y1
............................................cucgcugcgaucuUuugaaagu.............................................    1     1    7y1
............................................cucgcugcUaucuauugaaagu.............................................    2     1    7y1
............................................cucgUugcgaucuauugaaagu.............................................    1     1    7y1
............................................cucgcugcgaucuauugaaagG.............................................    1     1    7y1
............................................cucgcugcgaucCauugaaagu.............................................    1     1    7y1
............................................cucgcugcgaucuauugaaaUu.............................................    1     1    7y1
............................................cuAgcugcgaucuauugaaagu.............................................    4     1    7y1
............................................cucgcAgcgaucuauugaaagu.............................................    4     1    7y1
............................................cucgcugcgaucAauugaaagu.............................................    1     1    7y1
............................................cucgcugcgaucuauuUaaagu.............................................    1     1    7y1
............................................cucgcugcgaAcuauugaaagu.............................................    3     1    7y1
............................................Nucgcugcgaucuauugaaagu.............................................    1     1    7y1
............................................cucgcugcgaucuauAgaaagu.............................................    2     1    7y1
............................................cucgcugcgaucuauuCaaagu.............................................    1     1    7y1
............................................cucgcugAgaucuauugaaagu.............................................    3     1    7y1
............................................Aucgcugcgaucuauugaaagu.............................................    6     1    7y1
............................................cucgcugcgauGuauugaaagu.............................................    1     1    7y1
............................................cNcgcugcgaucuauugaaagu.............................................    1     1    7y1
............................................cucgGugcgaucuauugaaaguc............................................    1     1    7y1
............................................cucgcuAcgaucuauugaaaguc............................................    1     1    7y1
............................................cAcgcugcgaucuauugaaaguc............................................    7     1    7y1
............................................cucgcugcgaucuauuUaaaguc............................................    1     1    7y1
............................................cucgcugcgaucuauAgaaaguc............................................    1     1    7y1
............................................Nucgcugcgaucuauugaaaguc............................................    1     1    7y1
............................................Gucgcugcgaucuauugaaaguc............................................    1     1    7y1
............................................cucgcugcgaucuaAugaaaguc............................................    5     1    7y1
............................................cucgcuUcgaucuauugaaaguc............................................    1     1    7y1
............................................cucgcugcgaucuauugaaaCuc............................................    1     1    7y1
............................................cucCcugcgaucuauugaaaguc............................................    2     1    7y1
............................................cuAgcugcgaucuauugaaaguc............................................    5     1    7y1
............................................cucgcugcgaCcuauugaaaguc............................................    1     1    7y1
............................................cucgcugcUaucuauugaaaguc............................................    1     1    7y1
............................................cucgAugcgaucuauugaaaguc............................................    3     1    7y1
............................................cucgcugcgaucuaCugaaaguc............................................    1     1    7y1
............................................cucgcugcgaGcuauugaaaguc............................................    1     1    7y1
```

Star **Mature**

gaccugcuucugggucgggguuucguacguagcagagcagcucccucgcugcgaucuauugaaagucagcccucgacacaaggguuuguccgcgcgcgcgcgcgcgcgugcgu			
....cucgcugcgaucuauugaaaguc...	2329	0	7y1
....cucgcugcgGucuauugaaaguc...	1	1	7y1
....cucUcugcgaucuauugaaaguc...	1	1	7y1
....cuGgcugcgaucuauugaaaguc...	2	1	7y1
....cucgcugcgaucuauuCaaaguc...	4	1	7y1
....cucgcAgcgaucuauugaaaguc...	12	1	7y1
....cucgcugAgaucuauugaaaguc...	8	1	7y1
....cucgcuCcgaucuauugaaaguc...	2	1	7y1
....cucgcugcgauAuauugaaaguc...	4	1	7y1
....Aucgcugcgaucuauugaaaguc...	10	1	7y1
....cucgcugcgaucuauugaaaguA...	2	1	7y1
....cucgcugcgaucuauugaaaguca..	238	0	7y1
....cucgcugcgaAcuauugaaaguca..	1	1	7y1
....cucgcugAgaucuauugaaaguca..	1	1	7y1
....Aucgcugcgaucuauugaaaguca..	1	1	7y1
....cucgcugcgaucuauugaaaguAa..	3	1	7y1
....cuAgcugcgaucuauugaaaguca..	1	1	7y1
....ucgcAgcgaucuauugaa...	1	1	7y1
....ucgcugAgaucuauugaa...	1	1	7y1
....Acgcugcgaucuauugaa...	3	1	7y1
....ucgcugcgaucuauAgaa...	1	1	7y1
....ucgcugcgaucuauugaa...	152	0	7y1
....ucgcugcgaucuauugaaG..	2	1	7y1
....ucgcugcgaucuaCugaaag...	1	1	7y1
....ucgcugcgauGuauugaaag...	1	1	7y1
....Ncgcugcgaucuauugaaag...	2	1	7y1
....ucgcAgcgaucuauugaaag...	3	1	7y1
....ucgcugcgNucuauugaaag...	1	1	7y1
....ucgcugcgaucuUuugaaag...	1	1	7y1
....ucgcugcgaucuauAgaaag...	4	1	7y1
....Gcgcugcgaucuauugaaag...	1	1	7y1
....ucgcugAgaucuauugaaag...	17	1	7y1
....ucgcugcgaucuauugaaaC...	3	1	7y1
....ucgcugcgaucuauugaaUg...	1	1	7y1
....ucgcCgcgaucuauugaaag...	2	1	7y1
....ucgcuCcgaucuauugaaag...	1	1	7y1
....ucgcugcgaucuauuCaaag...	2	1	7y1
....ucgcugcgaucAauugaaag...	6	1	7y1
....ucgAugcgaucuauugaaag...	4	1	7y1
....ucgcugcgCucuauugaaag...	1	1	7y1
....ucgcugcgaucGauugaaag...	1	1	7y1
....ucgcugcgaucuaAugaaag...	3	1	7y1
....ucgcugcgaucuauugaaGg...	3	1	7y1
....ucgcugcgaucuauugaaag...	3906	0	7y1
....ucgcuUcgaucuauugaaag...	5	1	7y1
....ucgcugcgaucuauugaaCg...	1	1	7y1
....uAgcugcgaucuauugaaag...	12	1	7y1
....ucgcugcCaucuauugaaag...	1	1	7y1
....ucgcugcgaucuauuUaaag...	2	1	7y1
....ucgGugcgaucuauugaaag...	2	1	7y1
....ucgcugGgaucuauugaaag...	2	1	7y1
....ucgcugcgauNuauugaaag...	1	1	7y1
....ucUcugcgaucuauugaaag...	1	1	7y1
....ucgcugcgauAuauugaaag...	4	1	7y1
....uGgcugcgaucuauugaaag...	2	1	7y1
....ucgcugcgaAcuauugaaag...	11	1	7y1
....Acgcugcgaucuauugaaag...	60	1	7y1
....ucgcuAcgaucuauugaaag...	2	1	7y1
....ucgcGgcgaucuauugaaag...	1	1	7y1
....ucgcugcUaucuauugaaag...	2	1	7y1
....ucgcugcgaucuGuugaaag...	1	1	7y1
....Acgcugcgaucuauugaaagu..	4	1	7y1
....ucgcugcCaucuauugaaagu..	1	1	7y1
....ucgcugcgaAcuauugaaagu..	1	1	7y1
....ucgcugcgaucuauugaaagu..	138	0	7y1
....ucgcugcgaucAauugaaagu..	1	1	7y1
....ucgcugcgaucuauugaaagucagcccucgaG.....................................	1	1	7y1
....ucgcugcgaucuauugaaagucagcUcucgac.....................................	1	1	7y1
....ucgcugcgaucuauugaaagucagccAucgac.....................................	1	1	7y1
....Ncgcugcgaucuauugaaagucagcccucgac.....................................	1	1	7y1

Star **Mature**

gaccugcuucugggucgggguuucguacguagcagagcagcucccucgcugcgaucuauugaaagucagcccucgacacaaggguuuguccgcgcgcgcgcgcgcgcgugcgu

....ucgcugcgaucuaGugaaagucagcccucgac....................................	1	1	7y1
....ucgcugcgaucuaAugaaagucagcccucgac....................................	1	1	7y1
....ucgcugcgaucuauugaaagucagcccucgac....................................	506	0	7y1
....ucgcugcgaucuauuCaaagucagcccucgac....................................	1	1	7y1
....ucgcugcgaucuauugaaagucagcccucgaA....................................	1	1	7y1
....ucgcugcUaucuauugaaagucagcccucgac....................................	1	1	7y1
....Acgcugcgaucuauugaaagucagcccucgac....................................	7	1	7y1
....cgcugcgaucuaAugaaa...	1	1	7y1
....cUcugcgaucuauugaaa...	1	1	7y1
....cgcugAgaucuauugaaa...	1	1	7y1
....cCcugcgaucuauugaaa...	2	1	7y1
....cgcugcgaucuauugaaa...	357	0	7y1
....cgcuAcgaucuauugaaa...	1	1	7y1
....cgcugcgaAcuauugaaa...	1	1	7y1
....cgcugcUaucuauugaaa...	1	1	7y1
....cgcugcgaucuauugaaG...	1	1	7y1
....cgcugcgaGcuauugaaag..	2	1	7y1
....cgcugcgaucuUuugaaag..	1	1	7y1
....cgcugcgaucuauuCaaag..	1	1	7y1
....cgAugcgaucuauugaaag..	20	1	7y1
....cgcugcgaucuauugaUag..	4	1	7y1
....cgcugcgaucuauCgaaag..	1	1	7y1
....cgcugcgaucuauugaaaC..	1	1	7y1
....cgcGgcgaucuauugaaag..	2	1	7y1
....cgcugcgaucuauuUaaag..	150	1	7y1
....Agcugcgaucuauugaaag..	16	1	7y1
....cgGugcgaucuauugaaag..	3	1	7y1
....cgcugcgaucuauAgaaag..	9	1	7y1
....Ugcugcgaucuauugaaag..	2	1	7y1
....cgUugcgaucuauugaaag..	1	1	7y1
....cgcugAgaucuauugaaag..	29	1	7y1
....cgcugcgauGuauugaaag..	2	1	7y1
....cCcugcgaucuauugaaag..	4	1	7y1
....cgcugcgUucuauugaaag..	2	1	7y1
....cgcCgcgaucuauugaaag..	1	1	7y1
....cgcugcgaucuauugaGag..	2	1	7y1
....cgcugcgaucuauugaaag..	6574	0	7y1
....cgcugcgaucAauugaaag..	4	1	7y1
....Ngcugcgaucuauugaaag..	1	1	7y1
....cgcugGgaucuauugaaag..	2	1	7y1
....cUcugcgaucuauugaaag..	8	1	7y1
....cgcugcCaucuauugaaag..	4	1	7y1
....cgcuCcgaucuauugaaag..	2	1	7y1
....cgcugcgaucGauugaaag..	1	1	7y1
....cgcAgcgaucuauugaaag..	11	1	7y1
....cgcugcgauAuauugaaag..	14	1	7y1
....cgcugcgaucuauugaaaU..	4	1	7y1
....cgcugcgaucuauugUaag..	2	1	7y1
....cAcugcgaucuauugaaag..	1	1	7y1
....cgcuUcgaucuauugaaag..	7	1	7y1
....cgcugcgaAcuauugaaag..	9	1	7y1
....cgcugcgaucuauugaaUg..	1	1	7y1
....Ggcugcgaucuauugaaag..	4	1	7y1
....cgcugcgaucuaAugaaag..	7	1	7y1
....cgcuAcgaucuauugaaag..	1	1	7y1
....cgcugcUaucuauugaaag..	10	1	7y1
....cgcugcgaucuauugaaaA..	1	1	7y1
....cUcugcgaucuauugaaagu...	3	1	7y1
....cgcugcgaucuauugaaaCu...	1	1	7y1
....cgcugcgaucuauugaaagu...	160	0	7y1
....Ggcugcgaucuauugaaagu...	1	1	7y1
....cgcugcgaucuauAgaaagu...	1	1	7y1
....cgcugAgaucuauugaaagu...	2	1	7y1
....cgcugcgaucuaAugaaagu...	1	1	7y1
....cgcugcgaucuauugaaagAc..	1	1	7y1
....cgcugcgaucuauAgaaaguc..	2	1	7y1
....cgcugcgauAuauugaaaguc..	2	1	7y1
....cUcugcgaucuauugaaaguc..	4	1	7y1
....cgcugcgaAcuauugaaaguc..	1	1	7y1
....Agcugcgaucuauugaaaguc..	2	1	7y1

Star **Mature**

```
gaccugcuucugggucggggguuucguacguagcagagcagcucccucgcugcgaucuauugaaagucagcccucgacacaaggguuuguccgcgcgcgcgcgcgcgcgugcgu
..............................................cgcugcgaucuauugGaaguc..............................................	1	1	7y1
..............................................cgcugcgaucuauugaGaguc..............................................	1	1	7y1
..............................................cgcugcgaucuUuugaaaguc..............................................	1	1	7y1
..............................................cgcugAgaucuauugaaaguc..............................................	2	1	7y1
..............................................cgcugcgauUuauugaaaguc..............................................	1	1	7y1
..............................................cgcugcgaucuauugaaaguA..............................................	1	1	7y1
..............................................cgcugcgaucuauugaaaguc..............................................	553	0	7y1
..............................................cgcAgcgaucuauugaaaguc..............................................	1	1	7y1
..............................................cgcugcgaucuauugaaGgucag............................................	2	1	7y1
..............................................cgcugcgaucuauugaaagucag............................................	184	0	7y1
..............................................cgcugcgaucuauugaaagucaU............................................	1	1	7y1
..............................................cgcugcgaucuauAgaaagucag............................................	1	1	7y1
..............................................cgcugcgaucuaAugaaagucag............................................	1	1	7y1
..............................................cgcugAgaucuauugaaagucag............................................	1	1	7y1
..............................................cUcugcgaucuauugaaagucag............................................	1	1	7y1
..............................................Agcugcgaucuauugaaagucagcccucgac....................................	1	1	7y1
..............................................cgAugcgaucuauugaaagucagcccucgac....................................	1	1	7y1
..............................................cgcugAgaucuauugaaagucagcccucgac....................................	1	1	7y1
..............................................cgcugcgaucuauugaaagucagcccucgCc....................................	1	1	7y1
..............................................cgcugcgaucuauugaaagucagcccucgac....................................	162	0	7y1
...............................................gcuUcgaucuauugaaag................................................	4	1	7y1
...............................................gcugcgaucAauugaaag................................................	7	1	7y1
...............................................Ucugcgaucuauugaaag................................................	12	1	7y1
...............................................gcGgcgaucuauugaaag................................................	2	1	7y1
...............................................gcugcgaucuauuUaaag................................................	1	1	7y1
...............................................gcugcUaucuauugaaag................................................	5	1	7y1
...............................................gcugcgauAuauugaaag................................................	3	1	7y1
...............................................gcugcgaucuauugaaaC................................................	2	1	7y1
...............................................gcugcgaucuaAugaaag................................................	4	1	7y1
...............................................gcugcCaucuauugaaag................................................	2	1	7y1
...............................................gAugcgaucuauugaaag................................................	6	1	7y1
...............................................gcugcgaucuauugaaag................................................	2699	0	7y1
...............................................gcuCcgaucuauugaaag................................................	1	1	7y1
...............................................gcugcgaucuGuugaaag................................................	1	1	7y1
...............................................gcugcgaucuauuCaaag................................................	1	1	7y1
...............................................gcugcgaucuauugGaag................................................	1	1	7y1
...............................................gcugcgaAcuauugaaag................................................	1	1	7y1
...............................................gcAgcgaucuauugaaag................................................	4	1	7y1
...............................................gcugcgaucuauugaUag................................................	2	1	7y1
...............................................gGugcgaucuauugaaag................................................	2	1	7y1
...............................................Ccugcgaucuauugaaag................................................	8	1	7y1
...............................................gcugcgCucuauugaaag................................................	1	1	7y1
...............................................gcugGgaucuauugaaag................................................	1	1	7y1
...............................................gcugcgaucuauAgaaag................................................	8	1	7y1
...............................................gcugcgaucuUuugaaag................................................	1	1	7y1
...............................................gcugcgaucuauugaGag................................................	2	1	7y1
...............................................Acugcgaucuauugaaag................................................	1	1	7y1
...............................................gcugcgaucuaCugaaag................................................	1	1	7y1
...............................................Ncugcgaucuauugaaag................................................	2	1	7y1
...............................................gcugAgaucuauugaaag................................................	6	1	7y1
...............................................gcuAcgaucuauugaaag................................................	1	1	7y1
...............................................gcugcgaucuauugaaaU................................................	1	1	7y1
...............................................gcugcgaucuauugaaUg................................................	1	1	7y1
...............................................gcAgcgaucuauugaaagu...............................................	2	1	7y1
...............................................gcugcgaucuauugaaagu...............................................	111	0	7y1
...............................................gcugcgaucuauugaaagA...............................................	1	1	7y1
...............................................gcugcgaAcuauugaaagu...............................................	1	1	7y1
...............................................gAugcgaucuauugaaagu...............................................	2	1	7y1
...............................................gcugcgaucuauAgaaagu...............................................	1	1	7y1
...............................................gcugcgaucuauugaaagAc..............................................	2	1	7y1
...............................................gcugAgaucuauugaaaguc..............................................	1	1	7y1
...............................................gcugcgaAcuauugaaaguc..............................................	1	1	7y1
...............................................gcugcgaucuauugaaaguU..............................................	1	1	7y1
...............................................gcugcgaucuauAgaaaguc..............................................	1	1	7y1
...............................................gcuAcgaucuauugaaaguc..............................................	1	1	7y1
...............................................gAugcgaucuauugaaaguc..............................................	1	1	7y1
...............................................gcugcgaucuauugaaaUuc..............................................	1	1	7y1
...............................................Ucugcgaucuauugaaaguc..............................................	2	1	7y1
...............................................gcugcgaucuaAugaaaguc..............................................	1	1	7y1
...............................................gcugcgaucGauugaaaguc..............................................	1	1	7y1
```

Star **Mature**

```
gaccugcuucugggucgggguuucguacguagcagagcagcucccucgcugcgaucuauugaaagucagcccucgacacaaggguuuguccgcgcgcgcgcgcgcgcgcgugcgu
...............................................gcugcgaucuauuNaaaguc................................................ 1 1 7y1
...............................................gcugcgaucuaCugaaaguc................................................ 1 1 7y1
...............................................gcugcgaucAauugaaaguc................................................ 1 1 7y1
...............................................gcugcgaGcuauugaaaguc................................................ 1 1 7y1
...............................................gcugcgaucuauugaaaguA................................................ 1 1 7y1
...............................................gcuCcgaucuauugaaaguc................................................ 1 1 7y1
...............................................gcugcgaucuauugaaaguc................................................ 562 0 7y1
...............................................Acugcgaucuauugaaagucag.............................................. 1 1 7y1
...............................................gcugcgaucuauugaaagucag.............................................. 181 0 7y1
...............................................gAugcgaucuauugaaagucag.............................................. 2 1 7y1
................................................cugcgaAcuauugaaagu................................................. 2 1 7y1
................................................cugcgaucuauugaaagA................................................. 1 1 7y1
................................................Nugcgaucuauugaaagu................................................. 1 1 7y1
................................................cugcgaucuaAugaaagu................................................. 1 1 7y1
................................................cuAcgaucuauugaaagu................................................. 1 1 7y1
................................................cugcgaucuauugaaagu................................................. 301 0 7y1
................................................cugcgaucuauugaaaUu................................................. 1 1 7y1
................................................Gugcgaucuauugaaagu................................................. 1 1 7y1
................................................cAgcgaucuauugaaagu................................................. 1 1 7y1
................................................Augcgaucuauugaaagu................................................. 1 1 7y1
................................................cugAgaucuauugaaaguc................................................ 1 1 7y1
................................................cugcgaucuauugaaaguU................................................ 1 1 7y1
................................................cugcgaucuauAgaaaguc................................................ 1 1 7y1
................................................cuUcgaucuauugaaaguc................................................ 1 1 7y1
................................................cugcgaAcuauugaaaguc................................................ 1 1 7y1
................................................cAgcgaucuauugaaaguc................................................ 1 1 7y1
................................................cugcgauAuauugaaaguc................................................ 1 1 7y1
................................................cugcgaGcuauugaaaguc................................................ 1 1 7y1
................................................Augcgaucuauugaaaguc................................................ 3 1 7y1
................................................cugcgaucAauugaaaguc................................................ 1 1 7y1
................................................cugcgaucuauugaaagAc................................................ 1 1 7y1
................................................cugGgaucuauugaaaguc................................................ 1 1 7y1
................................................cugcUaucuauugaaaguc................................................ 1 1 7y1
................................................cugcgaucuauugaaaguc................................................ 436 0 7y1
................................................cugcgaucAauugaaagucagc............................................. 3 1 7y1
................................................cugcgaucCauugaaagucagc............................................. 337 1 7y1
................................................cugcgaucuauugaaagucagcccA.......................................... 1 1 7y1
................................................cuAcgaucuauugaaagucagcccu.......................................... 1 1 7y1
................................................Augcgaucuauugaaagucagcccu.......................................... 1 1 7y1
................................................cugcgaucuauugaaagucagcccu.......................................... 172 0 7y1
................................................cuCcgaucuauugaaagucagcccu.......................................... 1 1 7y1
................................................cugcgaucAauugaaagucagcccu.......................................... 1 1 7y1
................................................cuUcgaucuauugaaagucagcccu.......................................... 1 1 7y1
................................................cuUcgaucuauugaaagucagcccuc......................................... 169 1 7y1
................................................cugUgaucuauugaaagucagcccucgac...................................... 1 1 7y1
................................................cugcgaucuauugaaagucagcccucgac...................................... 218 0 7y1
................................................cugcgaucuauugaaaCucagcccucgac...................................... 1 1 7y1
................................................cugcgaucuauugaaagAcagcccucgac...................................... 1 1 7y1
................................................cugcgaucuauugaaagucagcccucgaA...................................... 1 1 7y1
.................................................ugAgaucuauugaaagucagc............................................. 1 1 7y1
.................................................ugcgaucuauugaaagucagc............................................. 702 0 7y1
.................................................ugcgaucuauugaaaguAagc............................................. 1 1 7y1
.................................................uCcgaucuauugaaagucagc............................................. 2 1 7y1
.................................................ugcgaucuauugaCagucagc............................................. 1 1 7y1
.................................................ugcgauAuauugaaagucagc............................................. 2 1 7y1
.................................................ugcgaucuauugaaagucagG............................................. 1 1 7y1
.................................................Ggcgaucuauugaaagucagc............................................. 2 1 7y1
.................................................ugcgaucuauugaaagucagU............................................. 1 1 7y1
.................................................ugcgaucuauCgaaagucagc............................................. 1 1 7y1
.................................................ugcgaucuauugaaagAcagc............................................. 1 1 7y1
.................................................Agcgaucuauugaaagucagc............................................. 10 1 7y1
.................................................ugcgaucuauuAaaagucagc............................................. 1 1 7y1
.................................................ugcgaucuauuCaaagucagc............................................. 1 1 7y1
.................................................ugcgaucuauugaaagucagcAcucgacac.................................... 1 1 7y1
.................................................ugcgaucuauugaaagucagcccucgacac.................................... 154 0 7y1
.................................................ugcgaucuauugaaagucagcccuUgacac.................................... 1 1 7y1
.................................................Agcgaucuauugaaagucagcccucgacac.................................... 1 1 7y1
.................................................ugAgaucuauugaaagucagcccucgacac.................................... 1 1 7y1
.................................................ugcgaucuauugaaagucagcccucgUcac.................................... 1 1 7y1
.................................................ugcgaucuauugaaagucagcccucgacaA.................................... 1 1 7y1
```

Star Mature

```
gaccugcuucugggucgggguuucguacguagcagagcagcucccucgcugcgaucuauugaaagucagcccucgacacaaggguuuguccgcgcgcgcgcgcgcgcgugcgu
..................................................gcgaucuauAgaaagucagc...........................................  1    1  7y1
..................................................gcgaucuauuCaaagucagc...........................................  1    1  7y1
..................................................gcgaucAauugaaagucagc...........................................  1    1  7y1
..................................................gcgaucuauugaaagCcagc...........................................  1    1  7y1
..................................................gcgaucCauugaaagucagc...........................................  157  1  7y1
..................................................gcgaucuauugaaagucagc...........................................  259  0  7y1
..................................................gcgaucuauugaGagucagc...........................................  390  1  7y1
..................................................gcgaucuauAgaaagucagcc..........................................  2    1  7y1
..................................................gcgaucuauuAaaagucagcc..........................................  1    1  7y1
..................................................gcgaucuauugaaaCucagcc..........................................  1    1  7y1
..................................................gcgaucuauugaaagucagcc..........................................  149  0  7y1
...................................................cgaucuauugaaagucagA...........................................  3    1  7y1
...................................................cgaucuauAgaaagucagc...........................................  4    1  7y1
...................................................cgaucuauugaaGgucagc...........................................  1    1  7y1
...................................................cgaucuauuUaaagucagc...........................................  1    1  7y1
...................................................cgauAuauugaaagucagc...........................................  1    1  7y1
...................................................Agaucuauugaaagucagc...........................................  1    1  7y1
...................................................cgaucuauugUaagucagc...........................................  1    1  7y1
...................................................cgaucuauugaaagucagc...........................................  565  0  7y1
...................................................cgaucAauugaaagucagc...........................................  2    1  7y1
...................................................cgaucuauGgaaagucagc...........................................  1    1  7y1
...................................................cgaucuauugaaaguGagc...........................................  1    1  7y1
...................................................cgaucuauugaaaguAagc...........................................  1    1  7y1
...................................................cUaucuauugaaagucagc...........................................  1    1  7y1
...................................................cgaucuauCgaaagucagc...........................................  1    1  7y1
...................................................cgaucuauugaaagucCgcccu........................................  1    1  7y1
...................................................cgaucuauugaaaUucagcccu........................................  1    1  7y1
...................................................cgaucuauugaaagucagcUcu........................................  1    1  7y1
...................................................cgaucAauugaaagucagcccu........................................  1    1  7y1
...................................................cgaucuauAgaaagucagcccu........................................  1    1  7y1
...................................................cgaucuauugaaagucagcccu........................................  243  0  7y1
...................................................cgaucuauugaaaguGagcccu........................................  1    1  7y1
...................................................cUaucuauugaaagucagcccu........................................  2    1  7y1
...................................................cgaucuaAugaaagucagcccu........................................  1    1  7y1
...................................................Ggaucuauugaaagucagcccucg......................................  1    1  7y1
...................................................cgauAuauugaaagucagcccucg......................................  1    1  7y1
...................................................Agaucuauugaaagucagcccucg......................................  2    1  7y1
...................................................cgaucAauugaaagucagcccucg......................................  2    1  7y1
...................................................cgaucuauugaaagAcagcccucg......................................  1    1  7y1
...................................................cgaucuauCgaaagucagcccucg......................................  1    1  7y1
...................................................cgaAcuauugaaagucagcccucg......................................  1    1  7y1
...................................................cgaucuauAgaaagucagcccucg......................................  1    1  7y1
...................................................cgaucuaGugaaagucagcccucg......................................  1    1  7y1
...................................................cgaucuauugaGagucagcccucg......................................  1    1  7y1
...................................................cCaucuauugaaagucagcccucg......................................  1    1  7y1
...................................................cgaucuauugaaagucagAccucg......................................  1    1  7y1
...................................................cgaucuauugaaagucagcccucg......................................  478  0  7y1
...................................................Ngaucuauugaaagucagcccucg......................................  1    1  7y1
...................................................cgUucuauugaaagucagcccucg......................................  1    1  7y1
...................................................Ngaucuauugaaagucagcccucgacacaa................................  1    1  7y1
...................................................Agaucuauugaaagucagcccucgacacaa................................  1    1  7y1
...................................................cgaucuauugaaagucagcccucgacacaG................................  1    1  7y1
...................................................cgaucAauugaaagucagcccucgacacaa................................  1    1  7y1
...................................................cgaucuauugaaagucagcccucgacacaa................................  237  0  7y1
...................................................cgaucuauugaaagucagcccucgCcacaa................................  1    1  7y1
...................................................cgauAuauugaaagucagcccucgacacaa................................  2    1  7y1
...................................................cgaucuauugaaagAcagcccucgacacaa................................  1    1  7y1
....................................................gaucuauuUaaagucagc...........................................  1    1  7y1
....................................................gaucCauugaaagucagc...........................................  142  1  7y1
....................................................gaucuauugaaagucagc...........................................  390  0  7y1
....................................................gaAcuauugaaagucagc...........................................  1    1  7y1
....................................................gaucAauugaaagucagc...........................................  2    1  7y1
....................................................gaucuauugaaagucagG...........................................  1    1  7y1
....................................................gauAuauugaaagucagc...........................................  1    1  7y1
....................................................gaucAauugaaagucagcc..........................................  2    1  7y1
....................................................gaucuauugaaaUucagcc..........................................  1    1  7y1
....................................................gaucuauuCaaagucagcc..........................................  2    1  7y1
....................................................gaucuaAugaaagucagcc..........................................  1    1  7y1
....................................................gaucuauugaaagucaCcc..........................................  1    1  7y1
....................................................gaucuauugaaagucGgcc..........................................  1    1  7y1
```

Star **Mature**

gaccugcuucugggucggggguuucguacguagcagagcagcucccucgcugcgaucuauugaaagucagcccucgacacaaggguuuguccgcgcgcgcgcgcgcgcgugcgu			
....gauGuauugaaagucagcc..	1	1	7y1
....gauAuauugaaagucagcc..	3	1	7y1
....gaucuauugaaagucagcA..	1	1	7y1
....gaucuauugaaagucagcG..	1	1	7y1
....gGucuauugaaagucagcc..	1	1	7y1
....gaucuauugaaaCucagcc..	2	1	7y1
....Caucuauugaaagucagcc..	1	1	7y1
....gaucuauugaaagucagcc..	683	0	7y1
....gaucuUuugaaagucagcc..	1	1	7y1
....gaucAauugaaagucagcccucg......................................	2	1	7y1
....gaucCauugaaagucagcccucg......................................	193	1	7y1
....aucuauugaaagucagcA..	13	1	7y1
....aucuauugaaagucagcc..	5120	0	7y1
....aucuauugaaagucaCcc..	5	1	7y1
....aucuauugaaaAucagcc..	1	1	7y1
....aucCauugaaagucagcc..	1243	1	7y1
....aucuauugaaagucaUcc..	2	1	7y1
....aucuUuugaaagucagcc..	1	1	7y1
....aucuauugaaagGcagcc..	1	1	7y1
....aucuauugaaaCucagcc..	4	1	7y1
....aucuauugaaagucagUc..	2	1	7y1
....aucuauugaaagAcagcc..	6	1	7y1
....aucuaAugaaagucagcc..	23	1	7y1
....auGuauugaaagucagcc..	1	1	7y1
....aAcuauugaaagucagcc..	19	1	7y1
....aucuauugaUagucagcc..	1	1	7y1
....auAuauugaaagucagcc..	27	1	7y1
....aucuauAgaaagucagcc..	21	1	7y1
....aucuaCugaaagucagcc..	1	1	7y1
....aucAauugaaagucagcc..	10	1	7y1
....aucuauugaaagucagcG..	2	1	7y1
....aucuauuCaaagucagcc..	4	1	7y1
....aucuauugaaGgucagcc..	1	1	7y1
....aucuauuUaaagucagcc..	3	1	7y1
....Uucuauugaaagucagcc..	3	1	7y1
....Nucuauugaaagucagcc..	5	1	7y1
....aucuauugaaaUucagcc..	2	1	7y1
....Gucuauugaaagucagcc..	4	1	7y1
....aucuauugaaUgucagcc..	1	1	7y1
....aucuauuAaaagucagcc..	2	1	7y1
....aucuauugaaaguAagcc..	12	1	7y1
....aucuauAgaaagucagccc...	8	1	7y1
....aucuUuugaaagucagccc...	1	1	7y1
....aucuauugaaagucaUccc...	1	1	7y1
....aucuauugaaagucagccU...	237	1	7y1
....aucuaCugaaagucagccc...	1	1	7y1
....aucuauugaaagucagccc...	1694	0	7y1
....aucuauugaaagAcagccc...	1	1	7y1
....aucuaAugaaagucagccc...	5	1	7y1
....auGuauugaaagucagccc...	2	1	7y1
....aucGauugaaagucagccc...	2	1	7y1
....aucuauugaaaUucagccc...	2	1	7y1
....aucuauuUaaagucagccc...	1	1	7y1
....aucuauugaaagucagccA...	6	1	7y1
....aucuauugaaagucagcAc...	4	1	7y1
....aucuauugaUagucagccc...	1	1	7y1
....aAcuauugaaagucagccc...	10	1	7y1
....aucuauugUaagucagccc...	1	1	7y1
....aucuauugaaaCucagccc...	1	1	7y1
....aucAauugaaagucagccc...	1	1	7y1
....aucCauugaaagucagccc...	209	1	7y1
....Nucuauugaaagucagccc...	1	1	7y1
....auAuauugaaagucagccc...	7	1	7y1
....aucuauugaaaguAagccc...	5	1	7y1
....Gucuauugaaagucagccc...	2	1	7y1
....aucuauugaaagucagccG...	3	1	7y1
....aucuauugaaagucagcGc...	2	1	7y1
....Nucuauugaaagucagcccu..	1	1	7y1
....aucuauugaaagAcagcccu..	1	1	7y1
....aucuauugaaUgucagcccu..	1	1	7y1

Star

Mature

gaccugcuucugggucgggguuucguacguagcagagcagcucccucgcugcgaucuauugaaagucagccccucgacacaaggguuuguccgcgcgcgcgcgcgcgcgugcgu			
....aucuauugaaagucagcAcu..	2	1	7y1
....aucuauuCaaagucagcccu..	1	1	7y1
....aucuauugaaagucaUcccu..	1	1	7y1
....auAuauugaaagucagcccu..	2	1	7y1
....aucuauugaaagucagcccG..	2	1	7y1
....aucuaAugaaagucagcccu..	4	1	7y1
....aucuauugaaagucagcccA..	1	1	7y1
....aucuauugaaagucagcccu..	755	0	7y1
....aucuauAgaaagucagcccu..	3	1	7y1
....aAcuauugaaagucagcccu..	2	1	7y1
....aucuauugaaagucagccAu..	1	1	7y1
....aucCauugaaagucagcccu..	223	1	7y1
....aucuauugaaaguAagcccu..	1	1	7y1
....aucCauugaaagucagcccuc.......................................	1285	1	7y1
....aucAauugaaagucagcccuc.......................................	2	1	7y1
....aucuauAgaaagucagcccucg......................................	4	1	7y1
....aucuauugaaagucagcccucg......................................	1785	0	7y1
....aucuauugaaagucagcccucU......................................	1	1	7y1
....aucuauugaaagucagcAcucg......................................	2	1	7y1
....aucuauugaaagucagAccucg......................................	6	1	7y1
....aCcuauugaaagucagcccucg......................................	1	1	7y1
....aucuauuUaaagucagcccucg......................................	1	1	7y1
....aucuauugaaaguAagcccucg......................................	5	1	7y1
....aucuauugNaagucagcccucg......................................	1	1	7y1
....aucuauugaaagAcagcccucg......................................	2	1	7y1
....aucuaAugaaagucagcccucg......................................	6	1	7y1
....aAcuauugaaagucagcccucg......................................	5	1	7y1
....auAuauugaaagucagcccucg......................................	8	1	7y1
....aucuauugaaagucagcccAcg......................................	1	1	7y1
....aucuauugaaagucagUccucg......................................	2	1	7y1
....aucuauugaaagucagGccucg......................................	1	1	7y1
....aucuauugaaaUucagcccucg......................................	1	1	7y1
....Gucuauugaaagucagcccucg......................................	1	1	7y1
....aucuauugaUagucagcccucg......................................	1	1	7y1
....aucuauugaaagucGgcccucg......................................	2	1	7y1
....aucCauugaaagucagcccucg......................................	914	1	7y1
....aucuauuCaaagucagcccucg......................................	2	1	7y1
....aucuauugaaagucagccAucg......................................	2	1	7y1
....aucuauugaaagucaCcccucg......................................	1	1	7y1
....auUuauugaaagucagcccucg......................................	249	1	7y1
....aucuauugGaagucagcccucg......................................	1	1	7y1
....aucuauugaaagucagcccucC......................................	2	1	7y1
....Nucuauugaaagucagcccucg......................................	1	1	7y1
....aucuauugaaagucagcccuAg......................................	4	1	7y1
....aucuauugaaaguGagcccucg......................................	1	1	7y1
....aucuauugaGagucagcccucg......................................	1	1	7y1
....aucAauugaaagucagcccucg......................................	1	1	7y1
....aucuauugaaagucaUcccucg......................................	1	1	7y1
....aucuauugaaagucagcAcucga.....................................	2	1	7y1
....aucuauugaaaguAagcccucga.....................................	1	1	7y1
....aucuauugaaagucagccAucga.....................................	2	1	7y1
....aucuauugaaagucagcUcucga.....................................	1	1	7y1
....aucuauugaaagucagcccucga.....................................	460	0	7y1
....auAuauugaaagucagcccucga.....................................	2	1	7y1
....aucuauugaaagucagcccuAga.....................................	1	1	7y1
....aucuauugaaagucagccGucga.....................................	1	1	7y1
....aucuauAgaaagucagcccucga.....................................	6	1	7y1
....aucuaAugaaagucagcccucga.....................................	2	1	7y1
....aAcuauugaaagucagcccucga.....................................	3	1	7y1
....aucuauCgaaagucagcccucga.....................................	1	1	7y1
....aucuauugUaagucagcccucga.....................................	1	1	7y1
....aucuauugaaagucagcccucgac....................................	272	0	7y1
....Uucuauugaaagucagcccucgac....................................	2	1	7y1
....aucuauAgaaagucagcccucgac....................................	2	1	7y1
....aucuaAugaaagucagcccucgac....................................	2	1	7y1
....aucuauugaaagucagcccucgaA....................................	1	1	7y1
....aAcuauugaaagucagcccucgac....................................	1	1	7y1
....aucuauugaaagucaAcccucgacacaag...............................	1	1	7y1
....aucuauugaaagucagcccucUacacaag...............................	1	1	7y1
....aucuauugaaagucagccAucgacacaag...............................	1	1	7y1

Star **Mature**

```
gaccugcuucugggucgggguuucguacguagcagagcagcucccucgcugcgaucuauugaaagucagcccucgacacaaggguuuguccgcgcgcgcgcgcgcgcgugcgu
.....................................................Nucuauugaaagucagcccucgacacaag...............................   1     1   7y1
.....................................................aAcuauugaaagucagcccucgacacaag...............................   1     1   7y1
.....................................................aucuauugaaagucagcccAcgacacaag...............................   1     1   7y1
.....................................................aucuauugaaagucagcccucgCcacaag...............................   1     1   7y1
.....................................................aucuaAugaaagucagcccucgacacaag...............................   1     1   7y1
.....................................................aucuauugaaagucCgcccucgacacaag...............................   1     1   7y1
.....................................................Uucuauugaaagucagcccucgacacaag...............................   1     1   7y1
.....................................................aucuauugaaagucagcccucgacUcaag...............................   1     1   7y1
.....................................................auAuauugaaagucagcccucgacacaag...............................   4     1   7y1
.....................................................aucuauugaaagucagcccucgacacaag...............................   494   0   7y1
.....................................................aucAauugaaagucagcccucgacacaag...............................   1     1   7y1
.....................................................aucuauugaaagucagcGcucgacacaag...............................   1     1   7y1
.....................................................aucuauugaaagucagcccucgacaGaag...............................   1     1   7y1
.....................................................aucuauuUaaagucagcccucgacacaag...............................   1     1   7y1
......................................................ucuauugaaagucagGcc.........................................   1     1   7y1
......................................................ucuauugaaagucagccA.........................................   6     1   7y1
......................................................ucuauugaaagucaUccc.........................................   2     1   7y1
......................................................ucuauugaaagucagccc.........................................   1308  0   7y1
......................................................ucuauugaaaCucagccc.........................................   1     1   7y1
......................................................ucuauugaaagucaAccc.........................................   1     1   7y1
......................................................ucuauugaaagucCgccc.........................................   1     1   7y1
......................................................ucuauuUaaagucagccc.........................................   3     1   7y1
......................................................ucuauugaaaguAagccc.........................................   3     1   7y1
......................................................ucuauuAaaagucagccc.........................................   2     1   7y1
......................................................ucuaAugaaagucagccc.........................................   3     1   7y1
......................................................Gcuauugaaagucagccc.........................................   2     1   7y1
......................................................ucuauugaaagucagcAc.........................................   1     1   7y1
......................................................ucuGuugaaagucagccc.........................................   1     1   7y1
......................................................ucuauugaaagucagUcc.........................................   4     1   7y1
......................................................ucuauAgaaagucagccc.........................................   3     1   7y1
......................................................ucuauugaGagucagccc.........................................   227   1   7y1
......................................................uAuauugaaagucagccc.........................................   5     1   7y1
......................................................ucuauugaaagucagccG.........................................   1     1   7y1
......................................................ucAauugaaagucagccc.........................................   3     1   7y1
......................................................Acuauugaaagucagccc.........................................   16    1   7y1
......................................................ucuauugaaagucagccAu........................................   5     1   7y1
......................................................ucuauugaaagGcagcccu........................................   1     1   7y1
......................................................ucuaAugaaagucagcccu........................................   1     1   7y1
......................................................ucuauugaaagucagcAcu........................................   3     1   7y1
......................................................Ncuauugaaagucagcccu........................................   1     1   7y1
......................................................ucuauugaaagucCgcccu........................................   1     1   7y1
......................................................ucGauugaaagucagcccu........................................   1     1   7y1
......................................................Acuauugaaagucagcccu........................................   6     1   7y1
......................................................ucuauAgaaagucagcccu........................................   3     1   7y1
......................................................ucuauugaaagucagcccG........................................   1     1   7y1
......................................................ucAauugaaagucagcccu........................................   1     1   7y1
......................................................ucuauugaaagucagUccu........................................   1     1   7y1
......................................................ucuauugaaagucagcccA........................................   1     1   7y1
......................................................ucuauuAaaagucagcccu........................................   1     1   7y1
......................................................ucuauugaaagucagcccu........................................   861   0   7y1
......................................................ucuauugaaaCucagcccu........................................   1     1   7y1
......................................................ucAauugaaagucagcccuc.......................................   1     1   7y1
......................................................ucCauugaaagucagcccuc.......................................   278   1   7y1
......................................................ucuauugaaagucCgcccucg......................................   1     1   7y1
......................................................ucAauugaaagucagcccucg......................................   1     1   7y1
......................................................ucuauugaaagucagccGucg......................................   1     1   7y1
......................................................ucuauAgaaagucagcccucg......................................   3     1   7y1
......................................................ucuauCgaaagucagcccucg......................................   1     1   7y1
......................................................ucuauugaaaUucagcccucg......................................   1     1   7y1
......................................................ucuauugaaagucagUccucg......................................   1     1   7y1
......................................................Gcuauugaaagucagcccucg......................................   1     1   7y1
......................................................ucCauugaaagucagcccucg......................................   282   1   7y1
......................................................ucuaAugaaagucagcccucg......................................   2     1   7y1
......................................................uAuauugaaagucagcccucg......................................   1     1   7y1
......................................................Ccuauugaaagucagcccucg......................................   1     1   7y1
......................................................ucuauugaaagucagcccucU......................................   1     1   7y1
......................................................ucuauugaaaguAagcccucg......................................   2     1   7y1
......................................................ucuauugaaagucagcAcucg......................................   3     1   7y1
......................................................ucuauugaaagucaCcccucg......................................   1     1   7y1
......................................................ucuauugaaagucagcUcucg......................................   2     1   7y1
```

Star **Mature**

```
gaccugcuucugggucggggguuucguacguagcagagcagcucccucgcugcgaucuauugaaagucagcccucgacacaaggguuuguccgcgcgcgcgcgcgcgcgcgugcgu
......................................................Acuauugaaagucagcccucg........................................   9     1   7y1
......................................................ucuauugaaagucagccAucg........................................   2     1   7y1
......................................................ucuauugaaagucagcccucA........................................   1     1   7y1
......................................................ucuauugaaagucagcccucg........................................   1220  0   7y1
......................................................ucuauugaaagucagcUcucga.......................................   1     1   7y1
......................................................Gcuauugaaagucagcccucga.......................................   2     1   7y1
......................................................ucuauugaaGgucagcccucga.......................................   1     1   7y1
......................................................ucAauugaaagucagcccucga.......................................   1     1   7y1
......................................................uAuauugaaagucagcccucga.......................................   3     1   7y1
......................................................Acuauugaaagucagcccucga.......................................   3     1   7y1
......................................................ucuaCugaaagucagcccucga.......................................   1     1   7y1
......................................................ucuaAugaaagucagcccucga.......................................   1     1   7y1
......................................................ucuauuUaaagucagcccucga.......................................   1     1   7y1
......................................................ucuauugaaagucagcccucga.......................................   428   0   7y1
......................................................ucuauugaaagucagcccucgG.......................................   2     1   7y1
......................................................ucuauugaaaguAagcccucga.......................................   1     1   7y1
......................................................ucuauAgaaagucagcccucga.......................................   2     1   7y1
......................................................Gcuauugaaagucagcccucgac......................................   1     1   7y1
......................................................ucuauugaUagucagcccucgac......................................   1     1   7y1
......................................................uAuauugaaagucagcccucgac......................................   1     1   7y1
......................................................ucuauugaaagucagcccucgac......................................   299   0   7y1
......................................................ucuauugaaagucagccAucgac......................................   1     1   7y1
......................................................ucAauugaaagucagcccucgac......................................   1     1   7y1
......................................................ucuauugaaagucagcccAcgac......................................   1     1   7y1
......................................................Acuauugaaagucagcccucgac......................................   2     1   7y1
.......................................................cuauCgaaagucagcccu..........................................   1     1   7y1
.......................................................cuauugaaagucagcccG..........................................   1     1   7y1
.......................................................cuauugaaagucagcccu..........................................   416   0   7y1
.......................................................cuaAugaaagucagcccu..........................................   1     1   7y1
.......................................................cAauugaaagucagcccu..........................................   2     1   7y1
.......................................................cuauugaaaAucagcccu..........................................   1     1   7y1
.......................................................Auauugaaagucagcccu..........................................   1     1   7y1
.......................................................cuauugaaaguAagcccu..........................................   1     1   7y1
.......................................................cuauugaaaCucagcccu..........................................   1     1   7y1
.......................................................cuauugaaagAcagcccuc.........................................   2     1   7y1
.......................................................cuauugaaagucagcccuc.........................................   186   0   7y1
.......................................................cCauugaaagucagcccuc.........................................   488   1   7y1
.......................................................cuauugaUagucagcccuc.........................................   1     1   7y1
.......................................................cAauugaaagucagcccuc.........................................   5     1   7y1
.......................................................Guauugaaagucagcccuc.........................................   1     1   7y1
.......................................................cCauugaaagucagcccucg........................................   701   1   7y1
.......................................................cAauugaaagucagcccucg........................................   4     1   7y1
.......................................................cAauugaaagucagcccucgac......................................   1     1   7y1
.......................................................cuauugaaagucagcccucgac......................................   273   0   7y1
.......................................................cuauugaaagucagcAcucgac......................................   1     1   7y1
.......................................................cuauugaaagucGgcccucgac......................................   1     1   7y1
.......................................................cuauugaaagucagccAucgac......................................   3     1   7y1
.......................................................Auauugaaagucagcccucgac......................................   1     1   7y1
.......................................................cuauugaaagAcagcccucgac......................................   1     1   7y1
.......................................................cuauugaaagucagcccucgacacaaggguuu............................   1     0   7y1
........................................................uauugaaaUucagcccucg........................................   1     1   7y1
........................................................Aauugaaagucagcccucg........................................   6     1   7y1
........................................................uauugaaagucagcccucC........................................   1     1   7y1
........................................................uauugaaagucagcGcucg........................................   1     1   7y1
........................................................uauugaaagucagcccAcg........................................   2     1   7y1
........................................................uauugaaagucagcccucg........................................   490   0   7y1
........................................................uauugaaGgucagcccucg........................................   1     1   7y1
........................................................uauugaaagucagcccuAg........................................   1     1   7y1
........................................................uauugaaagAcagcccucg........................................   1     1   7y1
........................................................uauugCaagucagcccucga.......................................   1     1   7y1
........................................................uauugaaagucagcccucgG.......................................   2     1   7y1
........................................................uauugaaagucaCcccucga.......................................   1     1   7y1
........................................................uauugaaUgucagcccucga.......................................   1     1   7y1
........................................................Aauugaaagucagcccucga.......................................   2     1   7y1
........................................................Gauugaaagucagcccucga.......................................   1     1   7y1
........................................................uauugaaagucagcccucga.......................................   197   0   7y1
........................................................uauugaaagucagcccucgac......................................   549   0   7y1
........................................................uaAugaaagucagcccucgac......................................   4     1   7y1
........................................................uauugaaagAcagcccucgac......................................   1     1   7y1
........................................................uauugaaaUucagcccucgac......................................   1     1   7y1
```

Star **Mature**

```
gaccugcuucugggucgggguuucguacguagcagagcagcucccucgcugcgaucuauugaaagucagcccucgacacaaggguuuguccgcgcgcgcgcgcgcgcgugcgu
.......................................................uauugaaagucagcAcucgac.....................................    1     1     7y1
.......................................................uauAgaaagucagcccucgac.....................................    2     1     7y1
.......................................................Aauugaaagucagcccucgac.....................................    11    1     7y1
.......................................................uauugaaagucagcccucUac.....................................    1     1     7y1
.......................................................uauugaaagucagcccCcgac.....................................    1     1     7y1
.......................................................uauugaaagucaCcccucgac.....................................    1     1     7y1
.......................................................uauugaaagucaUcccucgac.....................................    1     1     7y1
.......................................................uauugaaagucagAccucgac.....................................    1     1     7y1
.......................................................uauugaaagucagcccucgaGacaaggguu............................    1     1     7y1
.......................................................uauuCaaagucagcccucgacacaaggguu............................    1     1     7y1
.......................................................uauugaaaguAagcccucgacacaaggguu............................    1     1     7y1
.......................................................uauugaaagucagcAcucgacacaaggguu............................    2     1     7y1
.......................................................uauugaaagucagcccucgacacaaggAuu............................    1     1     7y1
.......................................................Aauugaaagucagcccucgacacaaggguu............................    1     1     7y1
.......................................................uauugaaagucagcccucgacacaaggguu............................    155   0     7y1
.......................................................uauugaaagucagcccAcgacacaaggguu............................    1     1     7y1
.......................................................uauugaaaUucagcccucgacacaaggguu............................    1     1     7y1
.......................................................uauugaaagucagcccucgacacaaggCuu............................    1     1     7y1
.......................................................uauugaaagucagcccucgacacaaggguG............................    1     1     7y1
.......................................................uauugaaagucagcccucgacaUaaggguuu...........................    1     1     7y1
.......................................................uauugaaagucagcccucgacacaagggAuu...........................    1     1     7y1
.......................................................uauugaaagucagAccucgacacaaggguuu...........................    1     1     7y1
.......................................................uauugaaagucagcccucgacacaaggguuu...........................    220   0     7y1
.......................................................uauugaaagucagcccAcgacacaaggguuu...........................    1     1     7y1
.......................................................Aauugaaagucagcccucgacacaaggguuu...........................    7     1     7y1
.......................................................uauugaaagucagccAucgacacaaggguuug..........................    1     1     7y1
.......................................................uauugaaagucagcccuAgacacaaggguuug..........................    3     1     7y1
.......................................................uauugaaagucagcccucgacacaaCgguuug..........................    1     1     7y1
.......................................................uauugaaagucagcccucgacacaagggAuug..........................    2     1     7y1
.......................................................uauugaaagucagcccucgacaAaaggguuug..........................    3     1     7y1
.......................................................Nauugaaagucagcccucgacacaaggguuug..........................    1     1     7y1
.......................................................uauugaaagucagcccucgacacaagggGuug..........................    1     1     7y1
.......................................................Gauugaaagucagcccucgacacaaggguuug..........................    2     1     7y1
.......................................................uauugaaaguAagcccucgacacaaggguuug..........................    2     1     7y1
.......................................................uauugaaagucagcccucgacacaaggguuAg..........................    1     1     7y1
.......................................................uauugaaagucagcccucgacacaagCguuug..........................    1     1     7y1
.......................................................uauugaaagucagGccucgacacaaggguuug..........................    1     1     7y1
.......................................................uauugaaagucGgcccucgacacaaggguuug..........................    1     1     7y1
.......................................................uauuCaaagucagcccucgacacaaggguuug..........................    1     1     7y1
.......................................................uauugaaagucagcccCcgacacaaggguuug..........................    1     1     7y1
.......................................................uauugaaagucagcccAcgacacaaggguuug..........................    2     1     7y1
.......................................................uauugaaagucagcGcucgacacaaggguuug..........................    1     1     7y1
.......................................................uauugaaagucaCcccucgacacaaggguuug..........................    2     1     7y1
.......................................................uauugaaagucagcccucgacacaagUguuug..........................    1     1     7y1
.......................................................uauugaaagucagcAcucgacacaaggguuug..........................    6     1     7y1
.......................................................uauugaaagucagAccucgacacaaggguuug..........................    1     1     7y1
.......................................................uauugaaaUucagcccucgacacaaggguuug..........................    1     1     7y1
.......................................................uauugaaagucagcccucgacacaaggguuug..........................    684   0     7y1
.......................................................uauuAaaagucagcccucgacacaaggguuug..........................    1     1     7y1
.......................................................Aauugaaagucagcccucgacacaaggguuug..........................    13    1     7y1
........................................................auugaaagucagccAucg.......................................    1     1     7y1
........................................................auuUaaagucagcccucg.......................................    1     1     7y1
........................................................auAgaaagucagcccucg.......................................    1     1     7y1
........................................................auuCaaagucagcccucg.......................................    1     1     7y1
........................................................auugaaagucagcccucg.......................................    487   0     7y1
........................................................aAugaaagucagcccucg.......................................    2     1     7y1
........................................................auugaGagucagcccucg.......................................    1     1     7y1
........................................................auugaaaguAagcccucg.......................................    2     1     7y1
........................................................auugaaagucagUccucg.......................................    1     1     7y1
........................................................Uuugaaagucagcccucg.......................................    1     1     7y1
........................................................auugaaagucagcAcucg.......................................    2     1     7y1
........................................................auugaaagucagAccucg.......................................    3     1     7y1
........................................................auugaaagucagcccucC.......................................    1     1     7y1
........................................................auugaaagucagcccuAga......................................    2     1     7y1
........................................................auugaaagucaUcccucga......................................    1     1     7y1
........................................................auugaaagucagcccucgC......................................    1     1     7y1
........................................................auugaaagucagAccucga......................................    1     1     7y1
........................................................auugaaagucagcccAcga......................................    1     1     7y1
........................................................auugaaagucagccAucga......................................    1     1     7y1
........................................................auugaaagAcagcccucga......................................    1     1     7y1
```

Star **Mature**

```
gaccugcuucugggucgggguuucguacguagcagagcagcucccucgcugcgaucuauugaaagucagcccucgacacaaggguuuguccgcgcgcgcgcgcgcgcgcgugcgu
.........................................................auugaaaguGagcccucga.......................................	1	1	7y1
.........................................................auugaaagucagcccucga.......................................	452	0	7y1
.........................................................aAugaaagucagcccucga.......................................	1	1	7y1
.........................................................auugaaagucagAccucgac......................................	1	1	7y1
.........................................................auugaaagucagccAucgac......................................	3	1	7y1
.........................................................aAugaaagucagcccucgac......................................	1	1	7y1
.........................................................auugaaagucagcccucgac......................................	419	0	7y1
.........................................................auugaaagucagUccucgac......................................	2	1	7y1
.........................................................auugaaagucagcccucgCc......................................	2	1	7y1
.........................................................auugaaagAcagcccucgac......................................	2	1	7y1
.........................................................auugaaaguAagcccucgac......................................	2	1	7y1
.........................................................auugaaagucagcccucgaca.....................................	189	0	7y1
.........................................................aAugaaagucagcccucgaca.....................................	2	1	7y1
.........................................................auugaaagucagcccucgaGa.....................................	1	1	7y1
.........................................................auugaaaguAagcccucgaca.....................................	2	1	7y1
.........................................................auugaaagucagcccucgacG.....................................	1	1	7y1
.........................................................auugaaagucagcAcucgacaca...................................	1	1	7y1
.........................................................auugaaaguAagcccucgacaca...................................	1	1	7y1
.........................................................auugaaagucagcccucgacaca...................................	165	0	7y1
.........................................................auugaaagucagcccucgCcaca...................................	4	1	7y1
.........................................................aAugaaagucagcccucgacaca...................................	1	1	7y1
.........................................................auugaaagucagcccucgUcaca...................................	1	1	7y1
.........................................................Uuugaaagucagcccucgacaca...................................	1	1	7y1
.........................................................auugaaagucagccAucgacaca...................................	2	1	7y1
.........................................................auugaaagucagcccAcgacacaag.................................	1	1	7y1
.........................................................auuCaaagucagcccucgacacaag.................................	1	1	7y1
.........................................................auugaaagucagccAucgacacaag.................................	2	1	7y1
.........................................................Uuugaaagucagcccucgacacaag.................................	1	1	7y1
.........................................................auugaaagucagcccucgCcacaag.................................	1	1	7y1
.........................................................auugaaaguAagcccucgacacaag.................................	1	1	7y1
.........................................................auugaaagucUgcccucgacacaag.................................	1	1	7y1
.........................................................auugaaaCucagcccucgacacaag.................................	1	1	7y1
.........................................................auugaaagucagcccucgacacaag.................................	209	0	7y1
.........................................................auugaaagucagcccucgacacaaC.................................	1	1	7y1
.........................................................Guugaaagucagcccucgacacaag.................................	2	1	7y1
.........................................................auugaaagucagcccucCacacaag.................................	1	1	7y1
.........................................................auugaaagucagcccucgacacaaggguu.............................	1	0	7y1
.........................................................auugaaaUucagcccucgacacaaggguuug...........................	1	1	7y1
.........................................................auugaaagucagccAucgacacaaggguuug...........................	1	1	7y1
.........................................................auugaaagucagcccucgacacaaggguAug...........................	1	1	7y1
.........................................................auugaaagucagcccuAgacacaaggguuug...........................	1	1	7y1
.........................................................auugaaagucagcccucgacacaaggguuAg...........................	2	1	7y1
.........................................................auugaaagucagcccAcgacacaaggguuug...........................	1	1	7y1
.........................................................auugaaagucagcccucgacacaaggguuug...........................	187	0	7y1
.........................................................auugaaagucagcccucgacacaagggGuug...........................	2	1	7y1
.........................................................auugaaagucagAccucgacacaaggguuug...........................	2	1	7y1
.........................................................auugaaagucagcccucgacGcaaggguuugu..........................	3	1	7y1
.........................................................auugaaagucagcccucgacaGaaggguuugu..........................	1	1	7y1
.........................................................auugaaagucagcccucgacacaaCgguuugu..........................	2	1	7y1
.........................................................auugaaagucagcccucUacacaaggguuugu..........................	1	1	7y1
.........................................................auugaaagucagccGucgacacaaggguuugu..........................	1	1	7y1
.........................................................auugaaagucaUcccucgacacaaggguuugu..........................	4	1	7y1
.........................................................auugaGagucagcccucgacacaaggguuugu..........................	2	1	7y1
.........................................................auugaaagucagcccucgacacaagCguuugu..........................	11	1	7y1
.........................................................auugaaagucagcUcucgacacaaggguuugu..........................	3	1	7y1
.........................................................Nuugaaagucagcccucgacacaaggguuugu..........................	4	1	7y1
.........................................................auugaaagAcagcccucgacacaaggguuugu..........................	6	1	7y1
.........................................................auugaaagucagcccucgacacaaggguuugG..........................	12	1	7y1
.........................................................auugaaaguUagcccucgacacaaggguuugu..........................	2	1	7y1
.........................................................auugaaagucagcccucgacacaGggguuugu..........................	2	1	7y1
.........................................................auugaaagucagcccucgacacaaggguGugu..........................	5	1	7y1
.........................................................auugaaagucCgcccucgacacaaggguuugu..........................	2	1	7y1
.........................................................aAugaaagucagcccucgacacaaggguuugu..........................	23	1	7y1
.........................................................auugaaagucagcccucgaAacaaggguuugu..........................	2	1	7y1
.........................................................auugaaagucagcccucgaUacaaggguuugu..........................	211	1	7y1
.........................................................auuAaaagucagcccucgacacaaggguuugu..........................	1	1	7y1
.........................................................auugaaagucagcccuAgacacaaggguuugu..........................	9	1	7y1
.........................................................auugaaagucagcccucgacaAaaggguuugu..........................	2	1	7y1
.........................................................Guugaaagucagcccucgacacaaggguuugu..........................	6	1	7y1
.........................................................auugaaagucagcccucgacacaaggguuugC..........................	1	1	7y1
```

Star **Mature**

```
gaccugcuucugggucgggguuucguacguagcagagcagcucccucgcugcgaucuauugaaagucagccccucgacacaaggguuuguccgcgcgcgcgcgcgcgcgugcgu
.........................................................auugGaagucagcccucgacacaaggguuugu........................   1     1   7y1
.........................................................auugaaagucagcccucgacacUaggguuugu........................   1     1   7y1
.........................................................auugaaagucagcccucgaGacaaggguuugu........................   2     1   7y1
.........................................................auuUaaagucagcccucgacacaaggguuugu........................   1     1   7y1
.........................................................auugaaagucagcccucgCcacaaggguuugu........................   19    1   7y1
.........................................................Uuugaaagucagcccucgacacaaggguuugu........................   8     1   7y1
.........................................................auAgaaagucagcccucgacacaaggguuugu........................   4     1   7y1
.........................................................auugaaagucagcccucgacacaaggUuuugu........................   3     1   7y1
.........................................................auugaaagucagcccucgacacaaggguuAgu........................   10    1   7y1
.........................................................auugaaagucagcccucgacacaaggguuugA........................   19    1   7y1
.........................................................auugaaagucagcccucgacacaagggAuugu........................   28    1   7y1
.........................................................auugaaagucagcccucgacacCaggguuugu........................   1     1   7y1
.........................................................auugaaagucagcccucgacacaaggguuuCu........................   11    1   7y1
.........................................................auugaaagucagcccucgacacaaUgguuugu........................   5     1   7y1
.........................................................auugaaagucagcccucgacCcaaggguuugu........................   1     1   7y1
.........................................................auugaaagucagccUucgacacaaggguuugu........................   1     1   7y1
.........................................................auugaaagCcagcccucgacacaaggguuugu........................   1     1   7y1
.........................................................auugaaagucagcAcucgacacaaggguuugu........................   22    1   7y1
.........................................................auugaaagucagAccucgacacaaggguuugu........................   16    1   7y1
.........................................................auugaaagucagcccAcgacacaaggguuugu........................   13    1   7y1
.........................................................auugaaagucagcccucgacacaaggguAugu........................   18    1   7y1
.........................................................auugaaagucagcccucgacacaagggCuugu........................   1     1   7y1
.........................................................auugaaagucagcccucgacacaaggAuuugu........................   1     1   7y1
.........................................................auugaaagucagcccucgacacaaggguuugu........................   7347  0   7y1
.........................................................auugaaaCucagcccucgacacaaggguuugu........................   4     1   7y1
.........................................................auGgaaagucagcccucgacacaaggguuugu........................   1     1   7y1
.........................................................auugCaagucagcccucgacacaaggguuugu........................   1     1   7y1
.........................................................auugaaagucagcccucgacacaaggCuuugu........................   13    1   7y1
.........................................................auugaaagucagcccucgacacaaggguuuUu........................   7     1   7y1
.........................................................auugaaagucagcGcucgacacaaggguuugu........................   1     1   7y1
.........................................................auuCaaagucagcccucgacacaaggguuugu........................   1     1   7y1
.........................................................auugaaaguAagcccucgacacaaggguuugu........................   13    1   7y1
.........................................................auugaaagucagcccucgUcacaaggguuugu........................   1     1   7y1
.........................................................auugaaagucagcccucgacacaagUguuugu........................   3     1   7y1
.........................................................auugaaagucGgcccucgacacaaggguuugu........................   1     1   7y1
.........................................................auugaaagucaAcccucgacacaaggguuugu........................   1     1   7y1
.........................................................auugaaagucagcccucgacacaagggGuugu........................   12    1   7y1
.........................................................auugaaagucagcccucCacacaaggguuugu........................   2     1   7y1
.........................................................auugaaagucUgcccucgacacaaggguuugu........................   1     1   7y1
.........................................................auCgaaagucagcccucgacacaaggguuugu........................   1     1   7y1
.........................................................auugaaagucagccAucgacacaaggguuugu........................   15    1   7y1
.........................................................auugaaaguGagcccucgacacaaggguuugu........................   4     1   7y1
.........................................................auugaaagucagcccucgacacGaggguuugu........................   2     1   7y1
.........................................................aCugaaagucagcccucgacacaaggguuugu........................   1     1   7y1
..........................................................uugaaagucagcccucga.....................................   208   0   7y1
..........................................................Augaaagucagcccucga.....................................   4     1   7y1
..........................................................uugaaagucagccAucga.....................................   3     1   7y1
..........................................................uAgaaagucagcccucga.....................................   1     1   7y1
..........................................................uugaaaguAagcccucgac....................................   1     1   7y1
..........................................................Augaaagucagcccucgac....................................   4     1   7y1
..........................................................uugaaagucagcAcucgac....................................   1     1   7y1
..........................................................uuUaaagucagcccucgac....................................   1     1   7y1
..........................................................uugaaagucagcccucgac....................................   211   0   7y1
..........................................................uugaaagucagcAcucgacacaaggguuug.........................   1     1   7y1
..........................................................uugaaagucagcccucgacacaagUguuug.........................   1     1   7y1
..........................................................uugaaagucagcccuAgacacaaggguuug.........................   1     1   7y1
..........................................................uugaaagucagcccucgacacaaggguuug.........................   182   0   7y1
..........................................................uugaaagucagGccucgacacaaggguuug.........................   1     1   7y1
..........................................................uugaaagucagccAucgacacaaggguuug.........................   1     1   7y1
..........................................................uugaaaguAagcccucgacacaaggguuug.........................   1     1   7y1
..........................................................uAgaaagucagcccucgacacaaggguuug.........................   1     1   7y1
..........................................................uugaaagucagcccucgacacaaCgguuug.........................   1     1   7y1
..........................................................uugaaagucagcccucgacUcaaggguuugu........................   1     1   7y1
..........................................................uugaaagucagcccucgacacaagggAuugu........................   6     1   7y1
..........................................................uugaaagucagAccucgacacaaggguuugu........................   2     1   7y1
..........................................................uugaaagucagcccucgacacaaggguuuCu........................   3     1   7y1
..........................................................uugaaagucagcccucgacacaaggguuugG........................   3     1   7y1
..........................................................uugaaagucagcccucgCcacaaggguuugu........................   4     1   7y1
..........................................................uugaaagAcagcccucgacacaaggguuugu........................   1     1   7y1
..........................................................uugaaagucagcccucgacacaaggguuAgu........................   6     1   7y1
```

Star **Mature**

```
gaccugcuucugggucgggguuucguacguagcagagcagcucccucgcugcgaucuauugaaagucagcccucgacacaaggguuuguccgcgcgcgcgcgcgcgugcgu
..........................................................uugaaagucagcccucgacacaagCguuugu......................   1     1   7y1
..........................................................uugaaagucagcAcucgacacaaggguuugu......................   1     1   7y1
..........................................................Gugaaagucagcccucgacacaaggguuugu......................   3     1   7y1
..........................................................uugaaagucagccAucgacacaaggguuugu......................   3     1   7y1
..........................................................uugaaagucagcccucgacacaaggguuugu......................   1439  0   7y1
..........................................................uugaaagucagcccuAgacacaaggguuugu......................   2     1   7y1
..........................................................uugaaagucagcccucgacacaagUguuugu......................   1     1   7y1
..........................................................Augaaagucagcccucgacacaaggguuugu......................   10    1   7y1
..........................................................uugaaagucagcccucgacacaaggguuuUu......................   2     1   7y1
..........................................................uugaaagucagcccucgacacaaggCuuugu......................   1     1   7y1
..........................................................uugaaagucagGccucgacacaaggguuugu......................   1     1   7y1
..........................................................uugaaagucagcccucgacacaaUgguuugu......................   1     1   7y1
..........................................................uugaaagucagccUucgacacaaggguuugu......................   1     1   7y1
..........................................................uugaaagucagcccucgacacaaggguAugu......................   4     1   7y1
..........................................................uugaaagucGgcccucgacacaaggguuugu......................   1     1   7y1
..........................................................uuCaaagucagcccucgacacaaggguuugu......................   2     1   7y1
..........................................................uugaaagucagcccucgacacaaggguGugu......................   1     1   7y1
..........................................................uugaaaCucagcccucgacacaaggguuugu......................   1     1   7y1
..........................................................uugaaaguAagcccucgacacaaggguuugu......................   4     1   7y1
..........................................................uugaaagucagcccucgacacaaggguuugA......................   4     1   7y1
..........................................................uAgaaagucagcccucgacacaaggguuugu......................   2     1   7y1
..........................................................uugaaagucagcccucgacacaagggGuugu......................   3     1   7y1
..........................................................uugaaagucagcccucgacacaaCgguuugu......................   2     1   7y1
..........................................................uCgaaagucagcccucgacacaaggguuugu......................   2     1   7y1
...........................................................Agaaagucagcccucgac..................................   4     1   7y1
...........................................................ugaaagAcagcccucgac..................................   1     1   7y1
...........................................................ugaaaguAagcccucgac..................................   2     1   7y1
...........................................................ugaaagucagcccucgac..................................   191   0   7y1
...........................................................ugaaagucagUccucgac..................................   1     1   7y1
...........................................................ugaaagucagcccucgCc..................................   1     1   7y1
...........................................................ugaaagucagcccucCacacaaggguuug.......................   1     1   7y1
...........................................................ugaaagucagcccucgacacaagggAuug.......................   2     1   7y1
...........................................................ugaaagucagcccGcgacacaaggguuug.......................   1     1   7y1
...........................................................ugaaagAcagcccucgacacaaggguuug.......................   1     1   7y1
...........................................................ugaaagucagccGucgacacaaggguuug.......................   1     1   7y1
...........................................................ugaaagucagcccucgacacaaggUuuug.......................   1     1   7y1
...........................................................ugaaagucagcccucgCcacaaggguuug.......................   1     1   7y1
...........................................................ugaaagucagcccucgacacaaUgguuug.......................   1     1   7y1
...........................................................uAaaagucagcccucgacacaaggguuug.......................   1     1   7y1
...........................................................ugaaagucagcccucgacacGaggguuug.......................   1     1   7y1
...........................................................ugaaagucagcccucgacacaaggguuug.......................   462   0   7y1
...........................................................ugaaagucagAccucgacacaaggguuug.......................   1     1   7y1
...........................................................ugaaagucagcccucgacacaaggguAug.......................   1     1   7y1
...........................................................ugaaagucagcccucgacacaaggguGug.......................   1     1   7y1
...........................................................Ggaaagucagcccucgacacaaggguuug.......................   1     1   7y1
...........................................................ugaaagucUgcccucgacacaaggguuug.......................   1     1   7y1
...........................................................ugaaagucagcccucgacacaaggguuGg.......................   2     1   7y1
............................................................gaaagucagcccucgacaUaaggguuugu......................   1     1   7y1
............................................................gaaagucagcccucgacCcaaggguuugu......................   1     1   7y1
............................................................gaaagucagAccucgacacaaggguuugu......................   1     1   7y1
............................................................gaaagucagcccAcgacacaaggguuugu......................   1     1   7y1
............................................................gaaagucagcccucgacacaaggguuAgu......................   1     1   7y1
............................................................Caaagucagcccucgacacaaggguuugu......................   1     1   7y1
............................................................gaaagucagcccucgacacaaggguuugu......................   170   0   7y1
............................................................gaGagucagcccucgacacaaggguuugu......................   1     1   7y1
............................................................gaaagucagcccucgacacaagggGuugu......................   1     1   7y1
..............................................................aagucagcccucgacacaaggguuuC.......................   2     1   7y1
..............................................................aagucagcccucgacacaaggguuug.......................   211   0   7y1
..............................................................aagucagcccucgacUcaaggguuug.......................   2     1   7y1
..............................................................aagucagccAucgacacaaggguuug.......................   2     1   7y1
..............................................................aagucagcccucgacacaaggUuuug.......................   1     1   7y1
.................................................................uAagcccucgacacaaggguuug.......................   1     1   7y1
.................................................................ucagcccucgGcacaaggguuug.......................   1     1   7y1
.................................................................Acagcccucgacacaaggguuug.......................   3     1   7y1
.................................................................Ncagcccucgacacaaggguuug.......................   1     1   7y1
.................................................................ucagccAucgacacaaggguuug.......................   1     1   7y1
.................................................................ucagcccucgacacaaggguuug.......................   244   0   7y1
.................................................................ucagcccucgacacaagggAuug.......................   1     1   7y1
.................................................................Gcagcccucgacacaaggguuug.......................   1     1   7y1
..................................................................cagcUcucgacacaaggguuugu......................   211   1   7y1
```

```
 Star                                                                   Mature
gaccugcuucugggucggggguuucguacguagcagagcagcucccucgcugcgaucuauugaaagucagcccucgacacaaggguuuguccgcgcgcgcgcgcgcgcgugcgu
...................................................................agccUucgacacaaggguuug...........................  132   1  7y1
....................................................................gcAcucgacacaaggguuug...........................  1     1  7y1
....................................................................gccAucgacacaaggguuug...........................  1     1  7y1
....................................................................Ncccucgacacaaggguuug...........................  1     1  7y1
....................................................................gAccucgacacaaggguuug...........................  2     1  7y1
....................................................................gcccucgaAacaaggguuug...........................  2     1  7y1
....................................................................gcccucgacacaaggguuug...........................  188   0  7y1
....................................................................gcccucAacacaaggguuug...........................  1     1  7y1
.....................................................................cccucgacacaaggguuuU...........................  1     1  7y1
.....................................................................ccAucgacacaaggguuugu..........................  2     1  7y1
.....................................................................Nccucgacacaaggguuugu..........................  1     1  7y1
.....................................................................cccucgacacaaggguuugu..........................  289   0  7y1
.....................................................................cccucgacacaagggAuugu..........................  2     1  7y1
.....................................................................cccucgacacaaggCuuugu..........................  2     1  7y1
.....................................................................cccucgacaAaaggguuugu..........................  1     1  7y1
.....................................................................cccucgacUcaaggguuugu..........................  1     1  7y1
.....................................................................cccucgacacaaggguuugG..........................  2     1  7y1
.....................................................................cccucgacacaaUgguuugu..........................  1     1  7y1
......................................................................Gcucgacacaaggguuug...........................  4     1  7y1
......................................................................ccucgacacaagAguuug...........................  1     1  7y1
......................................................................ccucgacaUaaggguuug...........................  1     1  7y1
......................................................................ccucUacacaaggguuug...........................  3     1  7y1
......................................................................cAucgacacaaggguuug...........................  6     1  7y1
......................................................................ccucgacUcaaggguuug...........................  2     1  7y1
......................................................................ccCcgacacaaggguuug...........................  1     1  7y1
......................................................................ccucgacacaaggguAug...........................  4     1  7y1
......................................................................ccucgacacaaggguuGg...........................  1     1  7y1
......................................................................ccucgaAacaaggguuug...........................  3     1  7y1
......................................................................ccAcgacacaaggguuug...........................  1     1  7y1
......................................................................ccucgacacaagCguuug...........................  2     1  7y1
......................................................................ccucgacacaaUgguuug...........................  1     1  7y1
......................................................................Ncucgacacaaggguuug...........................  2     1  7y1
......................................................................ccuGgacacaaggguuug...........................  1     1  7y1
......................................................................Acucgacacaaggguuug...........................  8     1  7y1
......................................................................ccucgacacaaggguuAg...........................  1     1  7y1
......................................................................ccucgacacaaggguuuA...........................  1     1  7y1
......................................................................ccucgacacaaggguuuC...........................  1     1  7y1
......................................................................ccucgacacaUggguuug...........................  1     1  7y1
......................................................................ccucgacacaaggCuuug...........................  1     1  7y1
......................................................................ccucgacacaaggguuug...........................  1954  0  7y1
......................................................................ccucgCcacaaggguuug...........................  2     1  7y1
......................................................................ccucgacacGaggguuug...........................  1     1  7y1
......................................................................ccucgacacaaUgguuugu..........................  5     1  7y1
......................................................................ccucgacacNaggguuugu..........................  1     1  7y1
......................................................................ccucgacacaaggguAugu..........................  8     1  7y1
......................................................................ccucgacacaGggguuugu..........................  1     1  7y1
......................................................................ccucUacacaaggguuugu..........................  7     1  7y1
......................................................................ccucgacacaaggguuuAu..........................  1     1  7y1
......................................................................ccucgacacaaggguuugA..........................  12    1  7y1
......................................................................ccucgacacaagUguuugu..........................  6     1  7y1
......................................................................ccucgUcacaaggguuugu..........................  1     1  7y1
......................................................................ccucgacaUaaggguuugu..........................  153   1  7y1
......................................................................Ucucgacacaaggguuugu..........................  358   1  7y1
......................................................................Acucgacacaaggguuugu..........................  24    1  7y1
......................................................................ccucgacacaaggguuuCu..........................  3     1  7y1
......................................................................ccucgacGcaaggguuugu..........................  3     1  7y1
......................................................................ccucgacacaaggUuuugu..........................  9     1  7y1
......................................................................cAucgacacaaggguuugu..........................  17    1  7y1
......................................................................ccucgacacaaCgguuugu..........................  3     1  7y1
......................................................................ccucgacacaaggCuuugu..........................  13    1  7y1
......................................................................ccucgCcacaaggguuugu..........................  7     1  7y1
......................................................................ccucgacacaaggguuugG..........................  17    1  7y1
......................................................................ccuGgacacaaggguuugu..........................  1     1  7y1
......................................................................Ncucgacacaaggguuugu..........................  3     1  7y1
......................................................................ccucgacacaagCguuugu..........................  4     1  7y1
......................................................................ccucgaUacaaggguuugu..........................  1     1  7y1
......................................................................ccucgacacaaggguuugC..........................  1     1  7y1
......................................................................ccAcgacacaaggguuugu..........................  14    1  7y1
......................................................................ccucgaAacaaggguuugu..........................  9     1  7y1
......................................................................ccucgacaAaaggguuugu..........................  8     1  7y1
```

```
Star                                                                   Mature
gaccugcuucugggucggggguucguacguagcagagcagcucccucgcugcgaucuauugaaagucagcccucgacacaaggguuuguccgcgcgcgcgcgcgcgugcgu
......................................................................ccucgacacGaggguuugu......................    1      1    7y1
......................................................................ccucgacacaaggguuugu......................    6344   0    7y1
......................................................................ccucgacacaaggguuuUu......................    5      1    7y1
......................................................................ccucgacacaagggGuugu......................    4      1    7y1
......................................................................ccucgGcacaaggguuugu......................    1      1    7y1
......................................................................Gcucgacacaaggguuugu......................    5      1    7y1
......................................................................ccucAacacaaggguuugu......................    4      1    7y1
......................................................................ccucgacacaagggAuugu......................    8      1    7y1
......................................................................ccucgacacaaggguuAgu......................    9      1    7y1
......................................................................ccucgacacaaggguCugu......................    1      1    7y1
......................................................................cNucgacacaaggguuuguccgcgc................    1      1    7y1
......................................................................ccucgacacaaggguuuguccgcgA................    1      1    7y1
......................................................................ccAcgacacaaggguuuguccgcgc................    1      1    7y1
......................................................................ccucgacacaaggguuuguccgcgc................    277    0    7y1
......................................................................ccucgacacaaggguAuguccgcgc................    1      1    7y1
......................................................................ccucgacacaaggCuuuguccgcgc................    1      1    7y1
......................................................................ccucgacacaaggUuuuguccgcgc................    1      1    7y1
......................................................................ccucgacacaagggAuuguccgcgc................    1      1    7y1
......................................................................ccuUgacacaaggguuuguccgcgc................    1      1    7y1
......................................................................cAucgacacaaggguuuguccgcgc................    1      1    7y1
.......................................................................cucgacacaaggguuAgu......................    30     1    7y1
.......................................................................cucgacacGaggguuugu......................    5      1    7y1
.......................................................................cucgacacaagggGuugu......................    22     1    7y1
.......................................................................cucgacacaaggguCugu......................    2      1    7y1
.......................................................................Gucgacacaaggguuugu......................    18     1    7y1
.......................................................................cNcgacacaaggguuugu......................    2      1    7y1
.......................................................................cucAacacaaggguuugu......................    13     1    7y1
.......................................................................Nucgacacaaggguuugu......................    10     1    7y1
.......................................................................cucgacUcaaggguuugu......................    9      1    7y1
.......................................................................cucgacacaaggguuugA......................    39     1    7y1
.......................................................................cucUacacaaggguuugu......................    14     1    7y1
.......................................................................cucgacacaaggguuCgu......................    1      1    7y1
.......................................................................cucgacacaaggguuuUu......................    19     1    7y1
.......................................................................cucgaUacaaggguuugu......................    3      1    7y1
.......................................................................cucgacacaaggguuugu......................    25979  0    7y1
.......................................................................cuGgacacaaggguuugu......................    197    1    7y1
.......................................................................cucgacacaaggCuuugu......................    17     1    7y1
.......................................................................cucgacacaagCguuugu......................    18     1    7y1
.......................................................................cucgacaUaaggguuugu......................    3      1    7y1
.......................................................................cucgacacaagUguuugu......................    175    1    7y1
.......................................................................cucgacacaaggguuugC......................    1      1    7y1
.......................................................................cucgacacaaCgguuugu......................    17     1    7y1
.......................................................................cucgacGcaaggguuugu......................    6      1    7y1
.......................................................................cucgacacaGggguuugu......................    1      1    7y1
.......................................................................cucgacacaaAgguuugu......................    197    1    7y1
.......................................................................cucgacacaaggguuugG......................    34     1    7y1
.......................................................................cucgacacaaggguuGgu......................    1      1    7y1
.......................................................................cucgacacaaUgguuugu......................    17     1    7y1
.......................................................................cucgaAacaaggguuugu......................    34     1    7y1
.......................................................................cucgacCcaaggguuugu......................    1      1    7y1
.......................................................................cucgacacCaggguuugu......................    1      1    7y1
.......................................................................cAcgacacaaggguuugu......................    75     1    7y1
.......................................................................cucgacacaaggguuuCu......................    23     1    7y1
.......................................................................cucgaNacaaggguuugu......................    1      1    7y1
.......................................................................cGcgacacaaggguuugu......................    2      1    7y1
.......................................................................cucgacacaaggguuuAu......................    7      1    7y1
.......................................................................cucgacacaaggguGugu......................    5      1    7y1
.......................................................................cucgacacaaggguAugu......................    35     1    7y1
.......................................................................cCcgacacaaggguuugu......................    2      1    7y1
.......................................................................cucgacacaagggAuugu......................    43     1    7y1
.......................................................................cucgacacaaggUuuugu......................    13     1    7y1
.......................................................................cucgacacaaggAuuugu......................    3      1    7y1
.......................................................................cucgacacUaggguuugu......................    2      1    7y1
.......................................................................cucgaGacaaggguuugu......................    2      1    7y1
.......................................................................cucgCcacaaggguuugu......................    4      1    7y1
.......................................................................cucgacaAaaggguuugu......................    21     1    7y1
.......................................................................cucgacacaUggguuugu......................    2      1    7y1
.......................................................................cucgacaGaaggguuugu......................    1      1    7y1
.......................................................................cucgUcacaaggguuugu......................    2      1    7y1
.......................................................................cucgacacaaggguuuguccgcgc................    1      0    7y1
```

Star **Mature**

gaccugcuucugggucgggguuucguacguagcagagcagcucccucgcugcgaucuauugaaagucagcccucgacacaaggguuuguccgcgcgcgcgcgcgcgcgugcgu
