# Supplementary material for: SEAweb: the small RNA Expression Atlas web application
Source: Nucleic Acids Res. 2019 Oct 10;48(D1):D204–19. doi: 10.1093/nar/gkz869 (PMC6943056; doi:10.1093/nar/gkz869)
Supplement: gkz869_Supplemental_Files [file gkz869_supplemental_files.zip › p-hsa-miR-235-3.pdf]

Provisional ID : chr21_12210
Score total : 144078.6
Score for star read(s) : 3.9
Score for read counts : 144071.6
Score for mfe : 2.1
Score for randfold : 1.6
Score for cons. seed : -0.6
Total read count : 282602
Mature read count : 36506
Loop read count : 245911
Star read count : 185

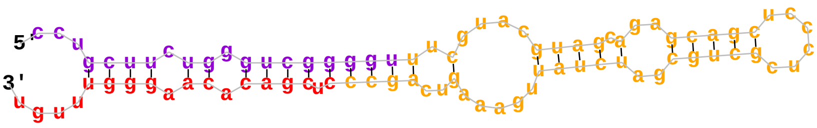


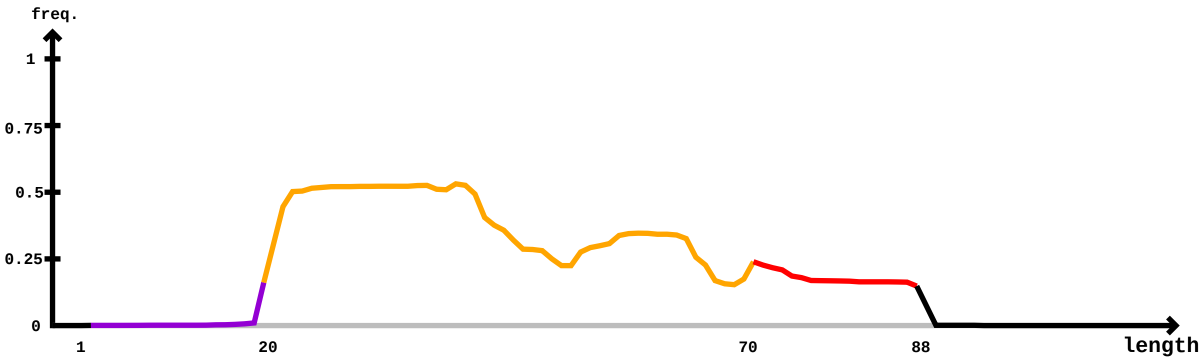


**Star** **Mature**

```
5'- gaccugcuucugggucgggguuucguacguagcagagcagcucccucgcugcgaucuauugaaagucagcccucgacacaaggguuuguccgcgcgcgcgcgcgcgugcgugc  -3'   obs
    gaccugcuucugggucgggguuucguacguagcagagcagcucccucgcugcgaucuauugaaagucagcccucgacacaaggguuuguccgcgcgcgcgcgcgcgugcgugc         exp
    (((..((((.((.(((((((((.(....((((.(..(((((......)))))..)))))).....)..))))).)))).)).)))).))).((((((((((....))))))))))   reads   mm   sample
    ..ccugcuucugggucgggg.............................................................................................   7       0    7y1
    ..ccugcuucugggucggggA............................................................................................   1       1    7y1
    ..ccugcuucugggucggggG............................................................................................   6       1    7y1
    ..cAugcuucugggucggggu............................................................................................   1       1    7y1
    ..ccugcuucugggucggggu............................................................................................   164     0    7y1
    ...cugcuucuggguAgggguuucgu.......................................................................................   2       1    7y1
    ...cugcuucugggucgggguAucgu.......................................................................................   1       1    7y1
    ...cugcuucugUgucgggguuucgu.......................................................................................   1       1    7y1
    ...cugcuucugggucgggUuuucgu.......................................................................................   2       1    7y1
    ....ugcuucAgggucgggguu...........................................................................................   1       1    7y1
    .....gcuucuggCucgggguuucgu.......................................................................................   1       1    7y1
    .....gcuucugggAcgggguuucgu.......................................................................................   1       1    7y1
    .....gcuucuggguAgggguuucgu.......................................................................................   1       1    7y1
    .....gcuucuggUucgggguuucgu.......................................................................................   1       1    7y1
    ......cuucugggucgggguuuG.........................................................................................   1       1    7y1
    ......cuucugggAcgggguuuc.........................................................................................   1       1    7y1
    ......cuucugggucgggguGuc.........................................................................................   1       1    7y1
    ......cuucuAggucgggguuuc.........................................................................................   1       1    7y1
    ......cuucugggucggCguuuc.........................................................................................   1       1    7y1
    ......cuucuUggucgggguuucgu.......................................................................................   1       1    7y1
    ......cuucugggAcgggguuucgu.......................................................................................   2       1    7y1
    ......cuucuCggucgggguuucgu.......................................................................................   1       1    7y1
    ......cuucuggguAgggguuucgu.......................................................................................   4       1    7y1
    ......cuucugggucCggguuucgu.......................................................................................   1       1    7y1
    ......cuucugggucggCguuucgu.......................................................................................   1       1    7y1
    ......cuucugggucUggguuucgu.......................................................................................   1       1    7y1
    ......cuucugggucggggGuucgu.......................................................................................   2       1    7y1
    ......cuucugggucgggAuuucgu.......................................................................................   1       1    7y1
    ......cuucugggucgggUuuucgu.......................................................................................   1       1    7y1
    ......cuucugggucgggguuucUu.......................................................................................   3       1    7y1
    ......cuucugggucgggguCucgu.......................................................................................   1       1    7y1
    ......cuucugggucgggguAucgu.......................................................................................   1       1    7y1
    ......cuucugggucggggAuucgu.......................................................................................   1       1    7y1
```

**Star** **Mature**

```
gaccugcuucugggucgggguuucguacguagcagagcagcucccucgcugcgaucuauugaaagucagcccucgacacaaggguuuguccgcgcgcgcgcgcgcgugcgugc
......cuucuggguGgggguuucgu.......................................................................................    1    1    7y1
......cuucugggucggUguuucgu.......................................................................................    1    1    7y1
.......uucugggAcgggguuucgu.......................................................................................    3    1    7y1
.......uucugggucgUgguuucgu.......................................................................................    2    1    7y1
.......uucuggguAggggguuucgu......................................................................................    6    1    7y1
.......uucugCgucgggguuucgu.......................................................................................    4    1    7y1
.......uucugggucUggguuucgu.......................................................................................    2    1    7y1
.......uucugggucggggCuucgu.......................................................................................    1    1    7y1
.......uucugggucggCguuucgu.......................................................................................    1    1    7y1
.......uucuUggucgggguuucgu.......................................................................................    2    1    7y1
.......uucugggucggUguuucgu.......................................................................................    1    1    7y1
.......uucugggucgggCuuucgu.......................................................................................    4    1    7y1
.......uucugggucgggguAucgu.......................................................................................    3    1    7y1
.......uucugggucggggGuucgu.......................................................................................    1    1    7y1
.......uucuggUucgggguuucgu.......................................................................................    2    1    7y1
.......uucugggucgggguCucgu.......................................................................................    2    1    7y1
.......uucugUgucgggguuucgu.......................................................................................    1    1    7y1
........ucuggCucgggguuucgu.......................................................................................    4    1    7y1
........ucugggucgggCuuucgu.......................................................................................    1    1    7y1
........ucugCgucgggguuucgu.......................................................................................    1    1    7y1
........ucugggucgUgguuucgu.......................................................................................    1    1    7y1
........ucuggguAgggguuucgu.......................................................................................    4    1    7y1
........ucugggucgCgguuucgu.......................................................................................    8    1    7y1
........ucuggAucgggguuucgu.......................................................................................    1    1    7y1
........ucugggucggggAuucgu.......................................................................................    1    1    7y1
........ucugggucggCguuucgu.......................................................................................    6    1    7y1
........ucugggucgggguAucgu.......................................................................................    4    1    7y1
........ucugggucCggguuucgu.......................................................................................    2    1    7y1
........ucugggucgggguCucgu.......................................................................................    1    1    7y1
........ucugUgucgggguuucgu.......................................................................................    2    1    7y1
........ucugggucgggUuuucgu.......................................................................................    1    1    7y1
........ucuAggucgggguuucgu.......................................................................................    2    1    7y1
........ucuggUucgggguuucgu.......................................................................................    7    1    7y1
........ucugggucggUguuucgu.......................................................................................    3    1    7y1
........ucugggucUggguuucgu.......................................................................................    2    1    7y1
........ucugggAcgggguuucgu.......................................................................................    4    1    7y1
........ucuggguGgggguuucgu.......................................................................................    1    1    7y1
........ucugggucgggguuAcgu.......................................................................................    1    1    7y1
........ucugggucgggUuuucgua......................................................................................    2    1    7y1
........ucugggucggggAuucgua......................................................................................    1    1    7y1
........ucugggucgggguuAcgua......................................................................................    1    1    7y1
........ucugggucUggguuucgua......................................................................................    3    1    7y1
........ucuggguAgggguuucgua......................................................................................    4    1    7y1
........ucugggucgggCuuucgua......................................................................................    1    1    7y1
........ucugggucgUgguuucgua......................................................................................    3    1    7y1
........ucuggguGgggguuucgua......................................................................................    1    1    7y1
........ucugggucgggguuGcgua......................................................................................    1    1    7y1
........ucugggucggUguuucgua......................................................................................    4    1    7y1
........ucugggucggCguuucgua......................................................................................    2    1    7y1
........ucugUgucgggguuucgua......................................................................................    2    1    7y1
........ucugggucgCgguuucgua......................................................................................    6    1    7y1
........ucuggAucgggguuucgua......................................................................................    1    1    7y1
........ucugggAcgggguuucgua......................................................................................    12   1    7y1
........ucugggucggAguuucgua......................................................................................    1    1    7y1
........ucugggucCggguuucgua......................................................................................    2    1    7y1
........ucugCgucgggguuucgua......................................................................................    2    1    7y1
........ucuggUucgggguuucgua......................................................................................    5    1    7y1
........ucugggucgggguAucgua......................................................................................    2    1    7y1
.........cugggucgggguuuAgua......................................................................................    5    1    7y1
.........cugggucgggUuuucgua......................................................................................    1    1    7y1
.........cugggucgCgguuucgua......................................................................................    1    1    7y1
.........cugggucgAgguuucgua......................................................................................    1    1    7y1
.........cugggucgggguuuGgua......................................................................................    1    1    7y1
.........cugCgucgggguuucgua......................................................................................    1    1    7y1
.........cuggguAgggguuucgua......................................................................................    5    1    7y1
.........cugggucUggguuucgua......................................................................................    1    1    7y1
.........cugggucggUguuucgua......................................................................................    1    1    7y1
.........cugggucggggGuucgua......................................................................................    1    1    7y1
.........cugggucgggguuAcguac.....................................................................................    1    1    7y1
.........cugggucgggguuuGguac.....................................................................................    1    1    7y1
```

**Star** **Mature**

```
gaccugcuucugggucgggguuucguacguagcagagcagcucccucgcugcgaucuauugaaagucagcccucgacacaaggguuuguccgcgcgcgcgcgcgcgugcgugc
.........cugggucgggguuuAguac.....................................................................................   1   1   7y1
.........cuggguAgggguuucguac.....................................................................................   1   1   7y1
.........cugggucCggguuucguac.....................................................................................   1   1   7y1
.........cugggucggCguuucguac.....................................................................................   1   1   7y1
.........cugggAcgggguuucguac.....................................................................................   1   1   7y1
.........cugggucgggUuuucguac.....................................................................................   1   1   7y1
..............ucgggguAucguacguag.................................................................................   1   1   7y1
..............ucggggAuucguacguag.................................................................................   1   1   7y1
..............ucggggAuucguacguagc................................................................................   1   1   7y1
..............ucgggUuuucguacguagc................................................................................   1   1   7y1
..............ucggggCuucguacguagc................................................................................   1   1   7y1
..............ucgggguAucguacguagc................................................................................   1   1   7y1
..............ucggggGuucguacguagc................................................................................   1   1   7y1
..............ucgggguuucguaUguagc................................................................................   1   1   7y1
..............ucgggguuucAuacguagcagagc...........................................................................   2   1   7y1
..............ucgggguGucguacguagcagagcagcuc......................................................................   1   1   7y1
..............ucgggguCucguacguagcagagcagcuc......................................................................   1   1   7y1
..............ucgggCuuucguacguagcagagcagcuc......................................................................   6   1   7y1
..............ucgggUuuucguacguagcagagcagcuc......................................................................   4   1   7y1
..............ucggggCuucguacguagcagagcagcuc......................................................................   2   1   7y1
..............ucggggAuucguacguagcagagcagcuc......................................................................   23  1   7y1
...............cgggguuucgCacguagc................................................................................   1   1   7y1
...............cggAguuucguacguagc................................................................................   1   1   7y1
...............cggggAuucguacguagc................................................................................   2   1   7y1
...............cgggguuucguaAguagc................................................................................   1   1   7y1
...............cgggguuAcguacguagc................................................................................   1   1   7y1
...............cgggguuucUuacguagc................................................................................   1   1   7y1
...............cggCguuucguacguagc................................................................................   1   1   7y1
...............cgggguAucguacguagc................................................................................   3   1   7y1
...............cgggguuucguacAuagca...............................................................................   1   1   7y1
...............cggggAuucguacguagca...............................................................................   2   1   7y1
...............cgggguuucUuacguagca...............................................................................   1   1   7y1
...............cgggguuucguaAguagca...............................................................................   1   1   7y1
...............cgggguuucCuacguagcag..............................................................................   1   1   7y1
...............cgggguuucguacCuagcag..............................................................................   1   1   7y1
...............Agggguuucguacguagcag..............................................................................   1   1   7y1
...............cgggguuucguacgAagcag..............................................................................   1   1   7y1
...............cggggAuucguacguagcag..............................................................................   1   1   7y1
...............cgggguuuAguacguagcag..............................................................................   1   1   7y1
...............cgggUuuucguacguagcag..............................................................................   1   1   7y1
...............cgggguGucguacguagcaga.............................................................................   1   1   7y1
...............cgggguuAcguacguagcaga.............................................................................   2   1   7y1
...............cgggguuuAguacguagcaga.............................................................................   1   1   7y1
...............cgggguuucUuacguagcaga.............................................................................   1   1   7y1
...............cgggguuucCuacguagcaga.............................................................................   1   1   7y1
...............cgggguuucguaGguagcaga.............................................................................   1   1   7y1
...............cgggguuucguacUuagcaga.............................................................................   4   1   7y1
...............cgggCuuucguacguagcaga.............................................................................   3   1   7y1
...............cgggguuucguaAguagcaga.............................................................................   3   1   7y1
...............cgggguuuGguacguagcaga.............................................................................   1   1   7y1
...............cgggguuucguacgCagcaga.............................................................................   1   1   7y1
...............cgggguAucguacguagcaga.............................................................................   4   1   7y1
...............cggggAuucguacguagcaga.............................................................................   5   1   7y1
...............cgggguuuUguacguagcaga.............................................................................   1   1   7y1
...............cggggGuucguacguagcaga.............................................................................   1   1   7y1
...............cgggguuucAuacguagcagag............................................................................   1   1   7y1
...............cgggguuuAguacguagcagag............................................................................   1   1   7y1
...............cgggguuucguacUuagcagag............................................................................   1   1   7y1
...............cgggUuuucguacguagcagag............................................................................   1   1   7y1
...............cgggguuucUuacguagcagag............................................................................   1   1   7y1
...............cgggguAucguacguagcagag............................................................................   2   1   7y1
...............cgggguuucguaAguagcagag............................................................................   3   1   7y1
...............cgggguuucgAacguagcagag............................................................................   1   1   7y1
...............cgggguuucguacUuagcagagc...........................................................................   2   1   7y1
...............cgggCuuucguacguagcagagc...........................................................................   3   1   7y1
...............cgggguuuAguacguagcagagc...........................................................................   7   1   7y1
...............cgggguuucgGacguagcagagc...........................................................................   1   1   7y1
...............cgggguuucCuacguagcagagc...........................................................................   2   1   7y1
...............cggggGuucguacguagcagagc...........................................................................   3   1   7y1
...............cgggguuucgAacguagcagagc...........................................................................   3   1   7y1
```

**Star** **Mature**

```
gaccugcuucugggucgggguuucguacguagcagagcagcucccucgcugcgaucuauugaaagucagcccucgacacaaggguuuguccgcgcgcgcgcgcgcgugcgugc
...............cgggguuucguacCuagcagagc...........................................................................    1    1    7y1
...............cgggguuucguaAguagcagagc...........................................................................    11   1    7y1
...............cggggAuucguacguagcagagc...........................................................................    27   1    7y1
...............cgggguuucUuacguagcagagc...........................................................................    1    1    7y1
...............cgggguuCcguacguagcagagc...........................................................................    2    1    7y1
...............cgggguuucAuacguagcagagc...........................................................................    1    1    7y1
...............cgggguuucguacgAagcagagc...........................................................................    3    1    7y1
...............cgggguuucgCacguagcagagc...........................................................................    3    1    7y1
...............cgggguAucguacguagcagagc...........................................................................    14   1    7y1
...............cgggUuuucguacguagcagagc...........................................................................    2    1    7y1
...............cgggguuucguGcguagcagagc...........................................................................    1    1    7y1
...............cgggguuucguacAuagcagagc...........................................................................    1    1    7y1
...............cgggguuuGguacguagcagagc...........................................................................    1    1    7y1
...............cgggguCucguacguagcagagc...........................................................................    1    1    7y1
...............cgggguuAcguacguagcagagc...........................................................................    3    1    7y1
...............cgggguuucguaUguagcagagc...........................................................................    1    1    7y1
...............cgggguuucguacgAagcagagca..........................................................................    3    1    7y1
...............cgggguuucgAacguagcagagca..........................................................................    3    1    7y1
...............cgggguuucCuacguagcagagca..........................................................................    2    1    7y1
...............cgggguuucguaAguagcagagca..........................................................................    4    1    7y1
...............cgggUuuucguacguagcagagca..........................................................................    1    1    7y1
...............cgggguuucgCacguagcagagca..........................................................................    1    1    7y1
...............cgggguuucguacUuagcagagca..........................................................................    2    1    7y1
...............cgggguuucguacgGagcagagca..........................................................................    1    1    7y1
...............cgggguuucguGcguagcagagca..........................................................................    2    1    7y1
...............cgggguuAcguacguagcagagca..........................................................................    6    1    7y1
...............cgggCuuucguacguagcagagca..........................................................................    3    1    7y1
...............cgggguuuAguacguagcagagca..........................................................................    5    1    7y1
...............cggggAuucguacguagcagagca..........................................................................    10   1    7y1
...............cgggguuucAuacguagcagagca..........................................................................    1    1    7y1
...............cggggGuucguacguagcagagca..........................................................................    1    1    7y1
...............cgggguuucgGacguagcagagca..........................................................................    1    1    7y1
...............cgggguuuUguacguagcagagca..........................................................................    1    1    7y1
...............cgggguuucUuacguagcagagca..........................................................................    4    1    7y1
...............cgggguAucguacguagcagagca..........................................................................    10   1    7y1
...............cgggAuuucguacguagcagagca..........................................................................    1    1    7y1
...............cgggguCucguacguagcagagca..........................................................................    1    1    7y1
...............cgggCuuucguacguagcagagcag.........................................................................    1    1    7y1
...............cgggguuucguacUuagcagagcag.........................................................................    1    1    7y1
...............cgggguuucgAacguagcagagcag.........................................................................    1    1    7y1
...............cgggguuucCuacguagcagagcag.........................................................................    1    1    7y1
...............cgggguuuAguacguagcagagcag.........................................................................    4    1    7y1
...............cgggguAucguacguagcagagcag.........................................................................    1    1    7y1
...............cgggguuAcguacguagcagagcag.........................................................................    1    1    7y1
...............cgggUuuucguacguagcagagcag.........................................................................    2    1    7y1
...............cgggguuuGguacguagcagagcag.........................................................................    1    1    7y1
...............cgggguuucguaAguagcagagcag.........................................................................    1    1    7y1
...............cggggCuucguacguagcagagcagc........................................................................    1    1    7y1
...............cgggguuuAguacguagcagagcagc........................................................................    2    1    7y1
...............cgggguuucguaAguagcagagcagc........................................................................    5    1    7y1
...............cgggguuucguacCuagcagagcagc........................................................................    1    1    7y1
...............cgggguAucguacguagcagagcagc........................................................................    9    1    7y1
...............cgggguGucguacguagcagagcagc........................................................................    1    1    7y1
...............cgggguuAcguacguagcagagcagc........................................................................    2    1    7y1
...............cgggguuucguaGguagcagagcagc........................................................................    1    1    7y1
...............cgggguuucgAacguagcagagcagc........................................................................    4    1    7y1
...............cgggguuCcguacguagcagagcagc........................................................................    1    1    7y1
...............cgggguuucAuacguagcagagcagc........................................................................    2    1    7y1
...............cgggguuucguacgAagcagagcagc........................................................................    2    1    7y1
...............cggggGuucguacguagcagagcagc........................................................................    1    1    7y1
...............cggggAuucguacguagcagagcagc........................................................................    10   1    7y1
...............cgggguuucgGacguagcagagcagc........................................................................    1    1    7y1
...............cgggguuucguacUuagcagagcagc........................................................................    2    1    7y1
...............cgggguuuUguacguagcagagcagc........................................................................    1    1    7y1
...............cgggguuucguacUuagcagagcagcu.......................................................................    2    1    7y1
...............cgggguuuAguacguagcagagcagcu.......................................................................    1    1    7y1
...............cggggAuucguacguagcagagcagcu.......................................................................    10   1    7y1
...............cgggguuucguGcguagcagagcagcu.......................................................................    1    1    7y1
...............cgggUuuucguacguagcagagcagcu.......................................................................    2    1    7y1
...............cgggguAucguacguagcagagcagcu.......................................................................    12   1    7y1
```

**Star** **Mature**

| | | | |
|---|---|---|---|
| gaccugcuucugggucgggguuucguacguagcagagcagcucccucgcugcgaucuauugaaagucagcccucgacacaagguuuguccgcgcgcgcgcgcgcgugcgugc | | | |
| ...............cgggguuucguacgAagcagagcagcu...................................................................... | 1 | 1 | 7y1 |
| ...............cgggguuucgAacguagcagagcagcu...................................................................... | 4 | 1 | 7y1 |
| ...............cgggguuucCuacguagcagagcagcu...................................................................... | 1 | 1 | 7y1 |
| ...............cgggguuucAuacguagcagagcagcu...................................................................... | 2 | 1 | 7y1 |
| ...............cgggguuucgGacguagcagagcagcu...................................................................... | 1 | 1 | 7y1 |
| ...............cgggguuuUguacguagcagagcagcu...................................................................... | 1 | 1 | 7y1 |
| ...............cgggguCucguacguagcagagcagcu...................................................................... | 1 | 1 | 7y1 |
| ...............cgggguuucguaAguagcagagcagcu...................................................................... | 2 | 1 | 7y1 |
| ...............cgggguuAcguacguagcagagcagcu...................................................................... | 4 | 1 | 7y1 |
| ...............cggggGuucguacguagcagagcagcu...................................................................... | 3 | 1 | 7y1 |
| ...............cggggCuucguacguagcagagcagcu...................................................................... | 1 | 1 | 7y1 |
| ...............cgggguuucUuacguagcagagcagcu...................................................................... | 1 | 1 | 7y1 |
| ...............cgggguGucguacguagcagagcagcuc.................................................................... | 2 | 1 | 7y1 |
| ...............cggggGuucguacguagcagagcagcuc.................................................................... | 4 | 1 | 7y1 |
| ...............cgggguuucUuacguagcagagcagcuc.................................................................... | 1 | 1 | 7y1 |
| ...............cgggguuuGguacguagcagagcagcuc.................................................................... | 1 | 1 | 7y1 |
| ...............cgggguuucguacUuagcagagcagcuc.................................................................... | 3 | 1 | 7y1 |
| ...............cgggguuucCuacguagcagagcagcuc.................................................................... | 1 | 1 | 7y1 |
| ...............cggggAuucguacguagcagagcagcuc.................................................................... | 12 | 1 | 7y1 |
| ...............cgggguuucguacCuagcagagcagcuc.................................................................... | 1 | 1 | 7y1 |
| ...............cgggguAucguacguagcagagcagcuc.................................................................... | 16 | 1 | 7y1 |
| ...............cgggguuucguacgAagcagagcagcuc.................................................................... | 1 | 1 | 7y1 |
| ...............cgggguuucAuacguagcagagcagcuc.................................................................... | 2 | 1 | 7y1 |
| ...............cgggUuuucguacguagcagagcagcuc.................................................................... | 2 | 1 | 7y1 |
| ...............cgggguuAcguacguagcagagcagcuc.................................................................... | 7 | 1 | 7y1 |
| ...............cgggguuucgAacguagcagagcagcuc.................................................................... | 2 | 1 | 7y1 |
| ...............cgggCuuucguacguagcagagcagcuc.................................................................... | 2 | 1 | 7y1 |
| ...............cgggguuucgCacguagcagagcagcuc.................................................................... | 4 | 1 | 7y1 |
| ...............cgggguuucguaAguagcagagcagcuc.................................................................... | 1 | 1 | 7y1 |
| ...............cgggguuucguaUguagcagagcagcuc.................................................................... | 1 | 1 | 7y1 |
| ...............cgggguuuAguacguagcagagcagcuc.................................................................... | 5 | 1 | 7y1 |
| ...............cggggAuucguacguagcagagcagcucccuc................................................................ | 1 | 1 | 7y1 |
| ................gggguuAcguacguagcaga............................................................................ | 2 | 1 | 7y1 |
| ................gggguuucguaAguagcaga............................................................................ | 1 | 1 | 7y1 |
| ................gggguuucgAacguagcagag........................................................................... | 1 | 1 | 7y1 |
| ................gggguuuAguacguagcagag........................................................................... | 1 | 1 | 7y1 |
| ................gggguuuAguacguagcagagc.......................................................................... | 2 | 1 | 7y1 |
| ................gggguuucUuacguagcagagc.......................................................................... | 1 | 1 | 7y1 |
| ................gggguuAcguacguagcagagc.......................................................................... | 3 | 1 | 7y1 |
| ................gggUuuucguacguagcagagc.......................................................................... | 3 | 1 | 7y1 |
| ................gggguAucguacguagcagagc.......................................................................... | 5 | 1 | 7y1 |
| ................gggguuucgAacguagcagagca......................................................................... | 1 | 1 | 7y1 |
| ................gggguuucguaAguagcagagca......................................................................... | 1 | 1 | 7y1 |
| ................gggguuuAguacguagcagagca......................................................................... | 1 | 1 | 7y1 |
| ................gggguAucguacguagcagagca......................................................................... | 2 | 1 | 7y1 |
| ................gggUuuucguacguagcagagca......................................................................... | 1 | 1 | 7y1 |
| ................gggguuAcguacguagcagagca......................................................................... | 2 | 1 | 7y1 |
| ................gggguuAcguacguagcagagcagc....................................................................... | 3 | 1 | 7y1 |
| ................gggguuucgAacguagcagagcagc....................................................................... | 1 | 1 | 7y1 |
| ................gggguuucguUcguagcagagcagc....................................................................... | 2 | 1 | 7y1 |
| ................gggguuucguacgCagcagagcagc....................................................................... | 1 | 1 | 7y1 |
| ................ggggGuucguacguagcagagcagc....................................................................... | 1 | 1 | 7y1 |
| ................gggguAucguacguagcagagcagc....................................................................... | 3 | 1 | 7y1 |
| ................gggguGucguacguagcagagcagc....................................................................... | 1 | 1 | 7y1 |
| ................gggguuCcguacguagcagagcagc....................................................................... | 1 | 1 | 7y1 |
| ................gggguuuAguacguagcagagcagc....................................................................... | 3 | 1 | 7y1 |
| ................gggguuucguacgAagcagagcagc....................................................................... | 1 | 1 | 7y1 |
| ................gggguuucguacguGgcagagcagc....................................................................... | 1 | 1 | 7y1 |
| ................gggguuucCuacguagcagagcagcu...................................................................... | 1 | 1 | 7y1 |
| ................gggguuucguacgCagcagagcagcu...................................................................... | 2 | 1 | 7y1 |
| ................gggguuucguacUuagcagagcagcu...................................................................... | 1 | 1 | 7y1 |
| ................ggggAuucguacguagcagagcagcu...................................................................... | 2 | 1 | 7y1 |
| ................gggguuucguGcguagcagagcagcu...................................................................... | 1 | 1 | 7y1 |
| ................gggCuuucguacguagcagagcagcu...................................................................... | 1 | 1 | 7y1 |
| ................ggggGuucguacguagcagagcagcu...................................................................... | 1 | 1 | 7y1 |
| ................gggguuucguaAguagcagagcagcu...................................................................... | 1 | 1 | 7y1 |
| ................gggguAucguacguagcagagcagcu...................................................................... | 6 | 1 | 7y1 |
| ................gggguuAcguacguagcagagcagcu...................................................................... | 1 | 1 | 7y1 |
| ................gggguuuAguacguagcagagcagcu...................................................................... | 1 | 1 | 7y1 |
| ................gggguuucguacAuagcagagcagcuc..................................................................... | 2 | 1 | 7y1 |

**Star** **Mature**

| | | | |
|---|---|---|---|
| gaccugcuucugggucgggguuucguacguagcagagcagcucccucgcugcgaucuauugaaagucagcccucgacacaagguuuguccgcgcgcgcgcgcgcgugcgugc | | | |
| ................gggguuucAuacguagcagagcagcuc..................................................................... | 1 | 1 | 7y1 |
| ................gggguAucguacguagcagagcagcuc..................................................................... | 9 | 1 | 7y1 |
| ................gggguuucguacUuagcagagcagcuc..................................................................... | 3 | 1 | 7y1 |
| ................gggguCucguacguagcagagcagcuc..................................................................... | 2 | 1 | 7y1 |
| ................gggguuGcguacguagcagagcagcuc..................................................................... | 1 | 1 | 7y1 |
| ................gggguuucguacguGgcagagcagcuc..................................................................... | 1 | 1 | 7y1 |
| ................gggguuucguaAguagcagagcagcuc..................................................................... | 3 | 1 | 7y1 |
| ................gggUuuucguacguagcagagcagcuc..................................................................... | 1 | 1 | 7y1 |
| ................gggguuuAguacguagcagagcagcuc..................................................................... | 8 | 1 | 7y1 |
| ................gggguuucCuacguagcagagcagcuc..................................................................... | 1 | 1 | 7y1 |
| ................gggguuucguacguUgcagagcagcuc..................................................................... | 1 | 1 | 7y1 |
| ................gggguuucguGcguagcagagcagcuc..................................................................... | 1 | 1 | 7y1 |
| ................ggggAuucguacguagcagagcagcuc..................................................................... | 1 | 1 | 7y1 |
| ................gggguuAcguacguagcagagcagcuc..................................................................... | 4 | 1 | 7y1 |
| ................gggguuucgAacguagcagagcagcuc..................................................................... | 2 | 1 | 7y1 |
| .................ggguuucguaAguagcaga............................................................................ | 1 | 1 | 7y1 |
| .................ggguuAcguacguagcaga............................................................................ | 1 | 1 | 7y1 |
| .................ggguuucguacgCagcaga............................................................................ | 1 | 1 | 7y1 |
| .................ggguuuAguacguagcaga............................................................................ | 1 | 1 | 7y1 |
| .................ggguuucAuacguagcagagc.......................................................................... | 1 | 1 | 7y1 |
| .................ggguAucguacguagcagagc.......................................................................... | 2 | 1 | 7y1 |
| .................gggGuucguacguagcagagc.......................................................................... | 5 | 1 | 7y1 |
| .................ggguuAcguacguagcagagc.......................................................................... | 1 | 1 | 7y1 |
| .................ggguuucguaAguagcagagc.......................................................................... | 1 | 1 | 7y1 |
| .................ggguuuAguacguagcagagc.......................................................................... | 5 | 1 | 7y1 |
| .................gggAuucguacguagcagagc.......................................................................... | 1 | 1 | 7y1 |
| .................ggguuucgAacguagcagagc.......................................................................... | 2 | 1 | 7y1 |
| .................ggguuucguaUguagcagagc.......................................................................... | 1 | 1 | 7y1 |
| .................ggguuucgGacguagcagagca......................................................................... | 1 | 1 | 7y1 |
| .................ggguuucUuacguagcagagca......................................................................... | 1 | 1 | 7y1 |
| .................gggGuucguacguagcagagca......................................................................... | 1 | 1 | 7y1 |
| .................ggCuuucguacguagcagagca......................................................................... | 269 | 1 | 7y1 |
| .................ggguuucgAacguagcagagca......................................................................... | 1 | 1 | 7y1 |
| .................ggguAucguacguagcagagca......................................................................... | 1 | 1 | 7y1 |
| .................ggguuucguaAguagcagagca......................................................................... | 1 | 1 | 7y1 |
| .................ggguCucguacguagcagagca......................................................................... | 1 | 1 | 7y1 |
| .................ggguuuGguacguagcagagca......................................................................... | 1 | 1 | 7y1 |
| .................ggguuucguacguGgcagagca......................................................................... | 1 | 1 | 7y1 |
| .................ggguuCcguacguagcagagca......................................................................... | 1 | 1 | 7y1 |
| .................ggguuuAguacguagcagagca......................................................................... | 3 | 1 | 7y1 |
| .................ggguuAcguacguagcagagca......................................................................... | 1 | 1 | 7y1 |
| .................ggguGucguacguagcagagca......................................................................... | 1 | 1 | 7y1 |
| .................ggguuucguacguaCcagagca......................................................................... | 1 | 1 | 7y1 |
| .................ggguuucguacguaUcagagca......................................................................... | 1 | 1 | 7y1 |
| .................ggguuucguaAguagcagagcagc....................................................................... | 1 | 1 | 7y1 |
| .................ggguuucgAacguagcagagcagc....................................................................... | 1 | 1 | 7y1 |
| .................ggguuuAguacguagcagagcagc....................................................................... | 2 | 1 | 7y1 |
| .................gggGuucguacguagcagagcagc....................................................................... | 1 | 1 | 7y1 |
| .................ggguuucguacCuagcagagcagcu...................................................................... | 1 | 1 | 7y1 |
| .................ggguAucguacguagcagagcagcu...................................................................... | 2 | 1 | 7y1 |
| .................ggguuuAguacguagcagagcagcu...................................................................... | 6 | 1 | 7y1 |
| .................ggCuuucguacguagcagagcagcu...................................................................... | 1 | 1 | 7y1 |
| .................gggGuucguacguagcagagcagcu...................................................................... | 2 | 1 | 7y1 |
| .................ggguuucguacguaUcagagcagcu...................................................................... | 1 | 1 | 7y1 |
| .................ggguuucCuacguagcagagcagcu...................................................................... | 1 | 1 | 7y1 |
| .................ggguuucgAacguagcagagcagcu...................................................................... | 1 | 1 | 7y1 |
| .................ggguuAcguacguagcagagcagcu...................................................................... | 5 | 1 | 7y1 |
| .................ggguuucguaUguagcagagcagcu...................................................................... | 1 | 1 | 7y1 |
| .................ggguuucguaAguagcagagcagcu...................................................................... | 1 | 1 | 7y1 |
| .................gggGuucguacguagcagagcagcuc..................................................................... | 2 | 1 | 7y1 |
| .................ggguuucguacUuagcagagcagcuc..................................................................... | 1 | 1 | 7y1 |
| .................ggguuAcguacguagcagagcagcuc..................................................................... | 6 | 1 | 7y1 |
| .................ggguAucguacguagcagagcagcuc..................................................................... | 5 | 1 | 7y1 |
| .................ggguuucguacgAagcagagcagcuc..................................................................... | 2 | 1 | 7y1 |
| .................ggguuCcguacguagcagagcagcuc..................................................................... | 1 | 1 | 7y1 |
| .................ggguuuAguacguagcagagcagcuc..................................................................... | 9 | 1 | 7y1 |
| .................ggguuucguaUguagcagagcagcuc..................................................................... | 1 | 1 | 7y1 |
| .................gggCuucguacguagcagagcagcuc..................................................................... | 1 | 1 | 7y1 |
| .................ggguuucguacCuagcagagcagcuc..................................................................... | 1 | 1 | 7y1 |
| .................ggguuucguaAguagcagagcagcuc..................................................................... | 2 | 1 | 7y1 |

**Star** **Mature**

```
gaccugcuucugggucgggguuucguacguagcagagcagcucccucgcugcgaucuauugaaagucagcccucgacacaaggguuuguccgcgcgcgcgcgcgcgugcgugc
.................ggguuucUuacguagcagagcagcuc......................................................................	1	1	7y1
.................ggguuucAuacguagcagagcagcuc......................................................................	2	1	7y1
.................ggguuucguacguCgcagagcagcuc......................................................................	1	1	7y1
.................ggguuucgAacguagcagagcagcuc......................................................................	1	1	7y1
.................ggguuucguacgAagcagagcagcucc.....................................................................	1	1	7y1
.................gggGuucguacguagcagagcagcucccu...................................................................	1	1	7y1
.................ggguuucgAacguagcagagcagcucccu...................................................................	1	1	7y1
.................ggguuucguacguGgcagagcagcucccu...................................................................	1	1	7y1
.................ggguuAcguacguagcagagcagcucccu...................................................................	1	1	7y1
.................ggguuuAguacguagcagagcagcucccu...................................................................	1	1	7y1
..................gguuAcguacguagcagagc...........................................................................	1	1	7y1
..................gguuucCuacguagcagagc...........................................................................	4	1	7y1
..................gguuucguacguaCcagagc...........................................................................	3	1	7y1
..................gguuucAuacguagcagagc...........................................................................	3	1	7y1
..................gguuucguacUuagcagagc...........................................................................	1	1	7y1
..................gUuuucguacguagcagagc...........................................................................	3	1	7y1
..................gguuucguacguagAagagc...........................................................................	5	1	7y1
..................gguuucguUcguagcagagc...........................................................................	1	1	7y1
..................gguuucguaGguagcagagc...........................................................................	1	1	7y1
..................gguuucgAacguagcagagc...........................................................................	9	1	7y1
..................gguuucguacguGgcagagc...........................................................................	1	1	7y1
..................gguuucguacCuagcagagc...........................................................................	2	1	7y1
..................ggGuucguacguagcagagc...........................................................................	1	1	7y1
..................ggAuucguacguagcagagc...........................................................................	4	1	7y1
..................gguGucguacguagcagagc...........................................................................	1	1	7y1
..................gguuucguacguaUcagagc...........................................................................	2	1	7y1
..................gguuucguaAguagcagagc...........................................................................	16	1	7y1
..................gguCucguacguagcagagc...........................................................................	1	1	7y1
..................gguuuAguacguagcagagc...........................................................................	11	1	7y1
..................gguuucguacguagGagagc...........................................................................	1	1	7y1
..................gguuucguacguUgcagagc...........................................................................	1	1	7y1
..................gguAucguacguagcagagc...........................................................................	3	1	7y1
..................gguuucguacgGagcagagc...........................................................................	1	1	7y1
..................gguuuGguacguagcagagc...........................................................................	2	1	7y1
..................gCuuucguacguagcagagc...........................................................................	2	1	7y1
..................gguuucguacgAagcagagc...........................................................................	5	1	7y1
..................gguuAcguacguagcagagca..........................................................................	1	1	7y1
..................gguuucguacCuagcagagca..........................................................................	1	1	7y1
..................gCuuucguacguagcagagca..........................................................................	1	1	7y1
..................gguuucguaUguagcagagca..........................................................................	1	1	7y1
..................gguuuAguacguagcagagca..........................................................................	3	1	7y1
..................gguuucgGacguagcagagca..........................................................................	1	1	7y1
..................gguuucguacguaAcagagca..........................................................................	1	1	7y1
..................gguuucgAacguagcagagca..........................................................................	1	1	7y1
..................gguCucguacguagcagagca..........................................................................	1	1	7y1
..................gguuucguacguaCcagagca..........................................................................	2	1	7y1
..................gguuucCuacguagcagagca..........................................................................	2	1	7y1
..................gguuucguacguagUagagca..........................................................................	1	1	7y1
..................gguuucguaAguagcagagca..........................................................................	3	1	7y1
..................ggAuucguacguagcagagca..........................................................................	1	1	7y1
..................gUuuucguacguagcagagcag.........................................................................	1	1	7y1
..................gguAucguacguagcagagcag.........................................................................	1	1	7y1
..................gguuucCuacguagcagagcag.........................................................................	1	1	7y1
..................gguuucgAacguagcagagcag.........................................................................	2	1	7y1
..................gguuuAguacguagcagagcag.........................................................................	1	1	7y1
..................ggAuucguacguagcagagcag.........................................................................	1	1	7y1
..................gguuucguacguagGagagcagc........................................................................	3	1	7y1
..................gCuuucguacguagcagagcagc........................................................................	3	1	7y1
..................gguuucguacCuagcagagcagc........................................................................	1	1	7y1
..................gguuucguaAguagcagagcagc........................................................................	5	1	7y1
..................gguuucguacgAagcagagcagc........................................................................	6	1	7y1
..................gguuucguacguagUagagcagc........................................................................	2	1	7y1
..................ggAuucguacguagcagagcagc........................................................................	7	1	7y1
..................gguuucgAacguagcagagcagc........................................................................	8	1	7y1
..................gguuucUuacguagcagagcagc........................................................................	4	1	7y1
..................gguuucguaGguagcagagcagc........................................................................	2	1	7y1
..................gguuucguacguaCcagagcagc........................................................................	2	1	7y1
..................gAuuucguacguagcagagcagc........................................................................	1	1	7y1
..................gguuucguacUuagcagagcagc........................................................................	3	1	7y1
..................gguuucguacgCagcagagcagc........................................................................	1	1	7y1
```

**Star** **Mature**

```
gaccugcuucugggucggggguuucguacguagcagagcagcucccucgcugcgaucuauugaaagucagcccucgacacaaggguuuguccgcgcgcgcgcgcgcgugcgugc
..................gguuucguacguaUcagagcagc........................................................................ 4 1 7y1
..................gguuucguacguagAagagcagc........................................................................ 4 1 7y1
..................ggGuucguacguagcagagcagc........................................................................ 1 1 7y1
..................gguuucCuacguagcagagcagc........................................................................ 3 1 7y1
..................gguuucguaUguagcagagcagc........................................................................ 1 1 7y1
..................gUuuucguacguagcagagcagc........................................................................ 6 1 7y1
..................gguAucguacguagcagagcagc........................................................................ 2 1 7y1
..................gguuuAguacguagcagagcagc........................................................................ 7 1 7y1
..................gguCucguacguagcagagcagc........................................................................ 2 1 7y1
..................gguuAcguacguagcagagcagcu....................................................................... 5 1 7y1
..................gUuuucguacguagcagagcagcu....................................................................... 4 1 7y1
..................gguuucguacgCagcagagcagcu....................................................................... 1 1 7y1
..................gguuuUguacguagcagagcagcu....................................................................... 1 1 7y1
..................gguuucguUcguagcagagcagcu....................................................................... 2 1 7y1
..................gguAucguacguagcagagcagcu....................................................................... 5 1 7y1
..................gCuuucguacguagcagagcagcu....................................................................... 2 1 7y1
..................ggAuucguacguagcagagcagcu....................................................................... 6 1 7y1
..................gguuucguaGguagcagagcagcu....................................................................... 1 1 7y1
..................gguuucguacAuagcagagcagcu....................................................................... 4 1 7y1
..................gguuucguacUuagcagagcagcu....................................................................... 1 1 7y1
..................gguuucgAacguagcagagcagcu....................................................................... 10 1 7y1
..................gguuGcguacguagcagagcagcu....................................................................... 1 1 7y1
..................gguuucUuacguagcagagcagcu....................................................................... 4 1 7y1
..................gguuucguacguagGagagcagcu....................................................................... 2 1 7y1
..................gguuucguacguaUcagagcagcu....................................................................... 4 1 7y1
..................gguuucguacguGgcagagcagcu....................................................................... 3 1 7y1
..................gguuucguGcguagcagagcagcu....................................................................... 1 1 7y1
..................gguuucAuacguagcagagcagcu....................................................................... 2 1 7y1
..................gguuucCuacguagcagagcagcu....................................................................... 2 1 7y1
..................gguuuGguacguagcagagcagcu....................................................................... 2 1 7y1
..................gguuucguaAguagcagagcagcu....................................................................... 20 1 7y1
..................gguuucguacgAagcagagcagcu....................................................................... 6 1 7y1
..................ggCuucguacguagcagagcagcu....................................................................... 1 1 7y1
..................gguuCcguacguagcagagcagcu....................................................................... 3 1 7y1
..................gguuucguacguagAagagcagcu....................................................................... 10 1 7y1
..................gguuuAguacguagcagagcagcu....................................................................... 20 1 7y1
..................gguuucguacCuagcagagcagcu....................................................................... 7 1 7y1
..................gguuucguacguaAcagagcagcu....................................................................... 1 1 7y1
..................gguuucguaUguagcagagcagcuc...................................................................... 1 1 7y1
..................gguuucAuacguagcagagcagcuc...................................................................... 1 1 7y1
..................gguuuAguacguagcagagcagcuc...................................................................... 3 1 7y1
..................ggGuucguacguagcagagcagcuc...................................................................... 2 1 7y1
..................gguuucguacgAagcagagcagcuc...................................................................... 4 1 7y1
..................gguuucUuacguagcagagcagcuc...................................................................... 1 1 7y1
..................gguuucguacCuagcagagcagcuc...................................................................... 2 1 7y1
..................gCuuucguacguagcagagcagcuc...................................................................... 3 1 7y1
..................gguuucCuacguagcagagcagcuc...................................................................... 1 1 7y1
..................gguuucguaAguagcagagcagcuc...................................................................... 6 1 7y1
..................gguuucguacguagUagagcagcuc...................................................................... 1 1 7y1
..................gguuucguacguaUcagagcagcuc...................................................................... 1 1 7y1
..................gguuucguacAuagcagagcagcuc...................................................................... 2 1 7y1
..................gguuucgCacguagcagagcagcuc...................................................................... 1 1 7y1
..................gguuucUuacguagcagagcagcucc..................................................................... 1 1 7y1
..................gguuucguacguaUcagagcagcucc..................................................................... 1 1 7y1
..................gUuuucguacguagcagagcagcucc..................................................................... 1 1 7y1
..................gguAucguacguagcagagcagcucc..................................................................... 1 1 7y1
..................ggAuucguacguagcagagcagcucc..................................................................... 4 1 7y1
..................gguuucguacgAagcagagcagcucc..................................................................... 2 1 7y1
..................gguuGcguacguagcagagcagcucc..................................................................... 1 1 7y1
..................gguuucguCcguagcagagcagcucc..................................................................... 1 1 7y1
..................gguuucgCacguagcagagcagcucc..................................................................... 1 1 7y1
..................gguuAcguacguagcagagcagcucc..................................................................... 2 1 7y1
..................gguuucguacguagAagagcagcucc..................................................................... 1 1 7y1
..................gguuucguacAuagcagagcagcucc..................................................................... 1 1 7y1
..................gguuucguNcguagcagagcagcucc..................................................................... 1 1 7y1
..................gguuucCuacguagcagagcagcucc..................................................................... 1 1 7y1
..................gguuucguaAguagcagagcagcucc..................................................................... 2 1 7y1
..................gguuucguacCuagcagagcagcucc..................................................................... 2 1 7y1
..................ggGuucguacguagcagagcagcucc..................................................................... 2 1 7y1
..................gguuuAguacguagcagagcagcucc..................................................................... 5 1 7y1
```

**Star** **Mature**

```
gaccugcuucugggucggggguuucguacguagcagagcagcucccucgcugcgaucuauugaaagucagcccucgacacaaggguuuguccgcgcgcgcgcgcgcgugcgugc
..................gCuuucguacguagcagagcagcucc.....................................................................   1   1   7y1
..................gguuucguUcguagcagagcagcucc.....................................................................   1   1   7y1
..................gguAucguacguagcagagcagcuccc....................................................................   2   1   7y1
..................gguuucUuacguagcagagcagcuccc....................................................................   4   1   7y1
..................gguuucguacguagGagagcagcuccc....................................................................   1   1   7y1
..................gguuucguacguaCcagagcagcuccc....................................................................   3   1   7y1
..................gguuucguacguaUcagagcagcuccc....................................................................   1   1   7y1
..................gguuucguacgAagcagagcagcuccc....................................................................   2   1   7y1
..................gguuuUguacguagcagagcagcuccc....................................................................   1   1   7y1
..................gguuucCuacguagcagagcagcuccc....................................................................   3   1   7y1
..................gguuucguacUuagcagagcagcuccc....................................................................   1   1   7y1
..................ggGuucguacguagcagagcagcuccc....................................................................   3   1   7y1
..................gguuucgGacguagcagagcagcuccc....................................................................   2   1   7y1
..................gguuucguacguagAagagcagcuccc....................................................................   5   1   7y1
..................gguuucguaGguagcagagcagcuccc....................................................................   2   1   7y1
..................ggCuucguacguagcagagcagcuccc....................................................................   1   1   7y1
..................gCuuucguacguagcagagcagcuccc....................................................................   1   1   7y1
..................gguuucguacgGagcagagcagcuccc....................................................................   1   1   7y1
..................gguuuAguacguagcagagcagcuccc....................................................................   15  1   7y1
..................ggAuucguacguagcagagcagcuccc....................................................................   3   1   7y1
..................gguuucguaAguagcagagcagcuccc....................................................................   11  1   7y1
..................gguuucguacCuagcagagcagcuccc....................................................................   4   1   7y1
..................gguuucgAacguagcagagcagcuccc....................................................................   2   1   7y1
..................gguuucguacAuagcagagcagcuccc....................................................................   1   1   7y1
..................gUuuucguacguagcagagcagcuccc....................................................................   2   1   7y1
..................gguuGcguacguagcagagcagcuccc....................................................................   1   1   7y1
..................gguuucguacgCagcagagcagcuccc....................................................................   1   1   7y1
..................gguuucguacguCgcagagcagcucccu...................................................................   1   1   7y1
..................gguuucguacgAagcagagcagcucccu...................................................................   5   1   7y1
..................gguuucguacCuagcagagcagcucccu...................................................................   2   1   7y1
..................gguuucguacguGgcagagcagcucccu...................................................................   2   1   7y1
..................gguuucguacguagAagagcagcucccu...................................................................   5   1   7y1
..................gguuucguacUuagcagagcagcucccu...................................................................   1   1   7y1
..................gguuuAguacguagcagagcagcucccu...................................................................   9   1   7y1
..................ggAuucguacguagcagagcagcucccu...................................................................   4   1   7y1
..................gCuuucguacguagcagagcagcucccu...................................................................   2   1   7y1
..................ggCuucguacguagcagagcagcucccu...................................................................   1   1   7y1
..................gguuucguacguagGagagcagcucccu...................................................................   1   1   7y1
..................gguuucguacguaUcagagcagcucccu...................................................................   3   1   7y1
..................ggGuucguacguagcagagcagcucccu...................................................................   2   1   7y1
..................gguuucguGcguagcagagcagcucccu...................................................................   1   1   7y1
..................gguuucguaAguagcagagcagcucccu...................................................................   12  1   7y1
..................gguuAcguacguagcagagcagcucccu...................................................................   2   1   7y1
..................gAuuucguacguagcagagcagcucccu...................................................................   1   1   7y1
..................gguuucgAacguagcagagcagcucccu...................................................................   7   1   7y1
..................gguuucgCacguagcagagcagcucccu...................................................................   1   1   7y1
..................gguuuUguacguagcagagcagcucccu...................................................................   1   1   7y1
..................gguuucguacguaAcagagcagcucccu...................................................................   2   1   7y1
..................gguuucguCcguagcagagcagcucccu...................................................................   2   1   7y1
..................gguuucUuacguagcagagcagcucccu...................................................................   5   1   7y1
..................gguuucguaUguagcagagcagcucccu...................................................................   1   1   7y1
..................gguuucAuacguagcagagcagcucccu...................................................................   1   1   7y1
..................gUuuucguacguagcagagcagcucccu...................................................................   1   1   7y1
..................gguAucguacguagcagagcagcucccu...................................................................   3   1   7y1
..................gguuucUuacguagcagagcagcucccuc..................................................................   1   1   7y1
..................gguuAcguacguagcagagcagcucccuc..................................................................   1   1   7y1
..................gguuucguGcguagcagagcagcucccuc..................................................................   1   1   7y1
..................gguuuGguacguagcagagcagcucccuc..................................................................   1   1   7y1
..................gguuuAguacguagcagagcagcucccuc..................................................................   1   1   7y1
..................gguuucguacAuagcagagcagcucccuc..................................................................   2   1   7y1
..................gguuucguaAguagcagagcagcucccuc..................................................................   1   1   7y1
..................ggCuucguacguagcagagcagcucccuc..................................................................   1   1   7y1
..................gguuucgAacguagcagagcagcucccuc..................................................................   2   1   7y1
..................ggAuucguacguagcagagcagcucccuc..................................................................   2   1   7y1
..................gguuucguacUuagcagagcagcucccuc..................................................................   2   1   7y1
..................gguuucguacguagAagagcagcucccuc..................................................................   1   1   7y1
..................gguuucCuacguagcagagcagcucccuc..................................................................   2   1   7y1
..................gguuucguacgAagcagagcagcucccuc..................................................................   1   1   7y1
..................gguuucguacguaCcagagcagcucccuc..................................................................   1   1   7y1
..................gguuucguacguaUcagagcagcucccuc..................................................................   1   1   7y1
```

**Star** **Mature**

```
gaccugcuucugggucggggguuucguacguagcagagcagcucccucgcugcgaucuauugaaagucagcccucgacacaaggguuuguccgcgcgcgcgcgcgugcgugc
..................gguuucguaGguagcagagcagcucccuc................................................................	1	1	7y1
..................gguuucgGacguagcagagcagcucccuc................................................................	1	1	7y1
..................gguuucguacguGgcagagcagcucccuc................................................................	1	1	7y1
..................gCuuucguacguagcagagcagcucccuc................................................................	1	1	7y1
..................gguuucguacguaCcagagcagcucccucg...............................................................	1	1	7y1
..................gguuucguacgAagcagagcagcucccucg...............................................................	3	1	7y1
..................gguuAcguacguagcagagcagcucccucg...............................................................	1	1	7y1
..................ggAuucguacguagcagagcagcucccucg...............................................................	3	1	7y1
..................gguuucguUcguagcagagcagcucccucg...............................................................	1	1	7y1
..................gguuucgGacguagcagagcagcucccucg...............................................................	1	1	7y1
..................gguuucguacguagUagagcagcucccucg...............................................................	1	1	7y1
..................gguuuAguacguagcagagcagcucccucg...............................................................	3	1	7y1
..................gCuuucguacguagcagagcagcucccucg...............................................................	1	1	7y1
..................gguuucguacguagAagagcagcucccucg...............................................................	2	1	7y1
..................gguuucCuacguagcagagcagcucccucg...............................................................	2	1	7y1
..................gguuucguacguagGagagcagcucccucg...............................................................	1	1	7y1
..................gguuucguaAguagcagagcagcucccucg...............................................................	1	1	7y1
..................gguuucguaUguagcagagcagcucccucg...............................................................	2	1	7y1
..................gguAucguacguagcagagcagcucccucg...............................................................	2	1	7y1
..................gguuucguacguaAcagagcagcucccucgc..............................................................	1	1	7y1
..................gguCucguacguagcagagcagcucccucgc..............................................................	1	1	7y1
..................gCuuucguacguagcagagcagcucccucgc..............................................................	1	1	7y1
..................gguuucAuacguagcagagcagcucccucgc..............................................................	1	1	7y1
..................ggGuucguacguagcagagcagcucccucgc..............................................................	2	1	7y1
..................gguuucguacUuagcagagcagcucccucgc..............................................................	1	1	7y1
..................gguuucguacguagUagagcagcucccucgc..............................................................	1	1	7y1
..................gguuucguacgAagcagagcagcucccucgc..............................................................	2	1	7y1
..................gguuuAguacguagcagagcagcucccucgc..............................................................	1	1	7y1
..................gguuucguacguGgcagagcagcucccucgc..............................................................	1	1	7y1
..................gguGucguacguagcagagcagcucccucgc..............................................................	1	1	7y1
..................ggAuucguacguagcagagcagcucccucgc..............................................................	2	1	7y1
..................gguAucguacguagcagagcagcucccucgc..............................................................	1	1	7y1
..................gguuucCuacguagcagagcagcucccucgc..............................................................	4	1	7y1
..................gUuuucguacguagcagagcagcucccucgc..............................................................	2	1	7y1
..................gguuucgAacguagcagagcagcucccucgc..............................................................	4	1	7y1
..................gguuCcguacguagcagagcagcucccucgc..............................................................	1	1	7y1
..................gguuucguaAguagcagagcagcucccucgc..............................................................	5	1	7y1
..................gguuucguacguagAagagcagcucccucgcu.............................................................	1	1	7y1
..................gguuucUuacguagcagagcagcucccucgcu.............................................................	1	1	7y1
..................ggAuucguacguagcagagcagcucccucgcu.............................................................	1	1	7y1
..................gguuucguacCuagcagagcagcucccucgcu.............................................................	1	1	7y1
..................gguuucguGcguagcagagcagcucccucgcu.............................................................	1	1	7y1
..................gguuucguacguaUcagagcagcucccucgcu.............................................................	1	1	7y1
..................gguuucguacguaCcagagcagcucccucgcu.............................................................	1	1	7y1
..................gUuuucguacguagcagagcagcucccucgcu.............................................................	1	1	7y1
..................gguuucAuacguagcagagcagcucccucgcu.............................................................	1	1	7y1
...................guuGcguacguagcagagc.........................................................................	1	1	7y1
...................guuucguaAguagcagagc.........................................................................	5	1	7y1
...................Nuuucguacguagcagagc.........................................................................	1	1	7y1
...................Uuuucguacguagcagagc.........................................................................	1	1	7y1
...................guuuAguacguagcagagc.........................................................................	1	1	7y1
...................guuucCuacguagcagagc.........................................................................	1	1	7y1
...................Cuuucguacguagcagagc.........................................................................	3	1	7y1
...................guuucgAacguagcagagc.........................................................................	3	1	7y1
...................guuucUuacguagcagagc.........................................................................	1	1	7y1
...................guuAcguacguagcagagca........................................................................	1	1	7y1
...................guuuAguacguagcagagca........................................................................	1	1	7y1
...................guuucguacguagAagagca........................................................................	2	1	7y1
...................gAuucguacguagcagagca........................................................................	1	1	7y1
...................guAucguacguagcagagca........................................................................	1	1	7y1
...................guuCcguacguagcagagca........................................................................	1	1	7y1
...................guuucCuacguagcagagca........................................................................	2	1	7y1
...................guuucguacCuagcagagca........................................................................	2	1	7y1
...................Uuuucguacguagcagagca........................................................................	1	1	7y1
...................guuucgAacguagcagagca........................................................................	5	1	7y1
...................Cuuucguacguagcagagca........................................................................	2	1	7y1
...................guuucguacgAagcagagcagc......................................................................	1	1	7y1
...................Auuucguacguagcagagcagc......................................................................	1	1	7y1
...................guuucguaAguagcagagcagc......................................................................	2	1	7y1
...................gAuucguacguagcagagcagc......................................................................	5	1	7y1
```

```
                  Star                                                                     Mature
gaccugcuucugggucggggguucguacguagcagagcagcucccucgcugcgaucuauugaaagucagcccucgacacaaggguuuguccgcgcgcgcgcgcgcgugcgugc
...................guAucguacguagcagagcagc........................................................................   3     1   7y1
...................Uuuucguacguagcagagcagc........................................................................   286   1   7y1
...................guuucgAacguagcagagcagc........................................................................   2     1   7y1
...................guuucguUcguagcagagcagc........................................................................   1     1   7y1
...................guAucguacguagcagagcagcu.......................................................................   4     1   7y1
...................guuucguacgAagcagagcagcu.......................................................................   3     1   7y1
...................Uuuucguacguagcagagcagcu.......................................................................   3     1   7y1
...................gGuucguacguagcagagcagcu.......................................................................   1     1   7y1
...................Cuuucguacguagcagagcagcu.......................................................................   2     1   7y1
...................guuucgAacguagcagagcagcu.......................................................................   3     1   7y1
...................guuucguacUuagcagagcagcu.......................................................................   1     1   7y1
...................Nuuucguacguagcagagcagcu.......................................................................   3     1   7y1
...................gAuucguacguagcagagcagcu.......................................................................   6     1   7y1
...................guuucguacguGgcagagcagcu.......................................................................   1     1   7y1
...................guuAcguacguagcagagcagcu.......................................................................   2     1   7y1
...................guuucguacguagAagagcagcu.......................................................................   1     1   7y1
...................guuucUuacguagcagagcagcu.......................................................................   1     1   7y1
...................guuuAguacguagcagagcagcu.......................................................................   1     1   7y1
...................gAuucguacguagcagagcagcuc......................................................................   2     1   7y1
...................guuuUguacguagcagagcagcuc......................................................................   1     1   7y1
...................guAucguacguagcagagcagcuc......................................................................   3     1   7y1
...................guCucguacguagcagagcagcuc......................................................................   1     1   7y1
...................gGuucguacguagcagagcagcuc......................................................................   1     1   7y1
...................Uuuucguacguagcagagcagcucc.....................................................................   1     1   7y1
...................guuucguacgAagcagagcagcucc.....................................................................   1     1   7y1
...................guAucguacguagcagagcagcucc.....................................................................   1     1   7y1
...................guuucguacguaAcagagcagcucc.....................................................................   1     1   7y1
...................guuucguacguaUcagagcagcucc.....................................................................   179   1   7y1
...................guuucguGcguagcagagcagcucc.....................................................................   1     1   7y1
...................gAuucguacguagcagagcagcuccc....................................................................   4     1   7y1
...................guuucguacguagAagagcagcuccc....................................................................   5     1   7y1
...................guuucguacguCgcagagcagcuccc....................................................................   1     1   7y1
...................guAucguacguagcagagcagcuccc....................................................................   2     1   7y1
...................Cuuucguacguagcagagcagcuccc....................................................................   2     1   7y1
...................guuucguacCuagcagagcagcuccc....................................................................   3     1   7y1
...................guuucguaAguagcagagcagcuccc....................................................................   2     1   7y1
...................guuAcguacguagcagagcagcuccc....................................................................   1     1   7y1
...................guuucgAacguagcagagcagcuccc....................................................................   2     1   7y1
...................guuucCuacguagcagagcagcuccc....................................................................   2     1   7y1
...................gCuucguacguagcagagcagcuccc....................................................................   2     1   7y1
...................guuucguacguaAcagagcagcuccc....................................................................   2     1   7y1
...................guuucguacgGagcagagcagcuccc....................................................................   1     1   7y1
...................guuucguacgAagcagagcagcuccc....................................................................   1     1   7y1
...................guuuAguacguagcagagcagcuccc....................................................................   3     1   7y1
...................guuuUguacguagcagagcagcuccc....................................................................   1     1   7y1
...................guuucguacguaCcagagcagcuccc....................................................................   3     1   7y1
...................guuucguacguaUcagagcagcuccc....................................................................   1     1   7y1
...................guuuAguacguagcagagcagcucccu...................................................................   1     1   7y1
...................guuAcguacguagcagagcagcucccu...................................................................   2     1   7y1
...................guuucCuacguagcagagcagcucccu...................................................................   1     1   7y1
...................guuucguacgGagcagagcagcucccu...................................................................   1     1   7y1
...................guuucguacAuagcagagcagcucccu...................................................................   2     1   7y1
...................gGuucguacguagcagagcagcucccu...................................................................   295   1   7y1
...................guuucguacgCagcagagcagcucccu...................................................................   1     1   7y1
...................guAucguacguagcagagcagcucccu...................................................................   2     1   7y1
...................guuucguacguagcGgagcagcucccu...................................................................   2     1   7y1
...................Cuuucguacguagcagagcagcucccu...................................................................   3     1   7y1
...................guuucguacCuagcagagcagcucccu...................................................................   1     1   7y1
...................guuucgAacguagcagagcagcucccu...................................................................   7     1   7y1
...................gAuucguacguagcagagcagcucccu...................................................................   7     1   7y1
...................guuucguacguagAagagcagcucccu...................................................................   3     1   7y1
...................Uuuucguacguagcagagcagcucccu...................................................................   2     1   7y1
...................guuucguacguagcGgagcagcucccuc..................................................................   1     1   7y1
...................guuucguGcguagcagagcagcucccuc..................................................................   2     1   7y1
...................gGuucguacguagcagagcagcucccuc..................................................................   1     1   7y1
...................guuucguacgAagcagagcagcucccuc..................................................................   1     1   7y1
...................guuucguaAguagcagagcagcucccuc..................................................................   1     1   7y1
...................guuucguacUuagcagagcagcucccuc..................................................................   3     1   7y1
...................guuucgAacguagcagagcagcucccuc..................................................................   2     1   7y1
...................guuucguacAuagcagagcagcucccuc..................................................................   1     1   7y1
```

```
  Star                                                                   Mature
gaccugcuucugggucgggguuucguacguagcagagcagcucccucgcugcgaucuauugaaagucagcccucgacacaaggguuuguccgcgcgcgcgcgcgcgugcgugc
...................guuCcguacguagcagagcagcucccuc..................................................................    1          1          7y1
...................guAucguacguagcagagcagcucccuc..................................................................    1          1          7y1
...................Cuuucguacguagcagagcagcucccuc..................................................................    1          1          7y1
...................guuucguacguagAagagcagcucccuc..................................................................    2          1          7y1
...................guuucguacguaUcagagcagcucccuc..................................................................    1          1          7y1
...................guuucguacCuagcagagcagcucccuc..................................................................    1          1          7y1
...................Uuuucguacguagcagagcagcucccuc..................................................................    3          1          7y1
...................guuucguacguaUcagagcagcucccucg.................................................................    1          1          7y1
...................guuucgAacguagcagagcagcucccucg.................................................................    4          1          7y1
...................gAuucguacguagcagagcagcucccucg.................................................................    1          1          7y1
...................guAucguacguagcagagcagcucccucg.................................................................    1          1          7y1
...................guuucguacAuagcagagcagcucccucg.................................................................    1          1          7y1
...................guuucguacguagcGgagcagcucccucg.................................................................    1          1          7y1
...................guuucguacgCagcagagcagcucccucgc................................................................    1          1          7y1
...................guuucCuacguagcagagcagcucccucgc................................................................    1          1          7y1
...................guuucguacCuagcagagcagcucccucgc................................................................    1          1          7y1
...................guuucguacUuagcagagcagcucccucgc................................................................    2          1          7y1
...................Uuuucguacguagcagagcagcucccucgc................................................................    1          1          7y1
...................guuucguaAguagcagagcagcucccucgc................................................................    3          1          7y1
...................gAuucguacguagcagagcagcucccucgc................................................................    1          1          7y1
...................guuucgGacguagcagagcagcucccucgc................................................................    1          1          7y1
...................gGuucguacguagcagagcagcucccucgc................................................................    1          1          7y1
...................guuucguacguaUcagagcagcucccucgc................................................................    2          1          7y1
...................Nuuucguacguagcagagcagcucccucgc................................................................    1          1          7y1
...................guAucguacguagcagagcagcucccucgc................................................................    2          1          7y1
...................guuucguacgAagcagagcagcucccucgc................................................................    1          1          7y1
...................guuucgAacguagcagagcagcucccucgc................................................................    4          1          7y1
...................guuAcguacguagcagagcagcucccucgc................................................................    2          1          7y1
...................Cuuucguacguagcagagcagcucccucgc................................................................    1          1          7y1
...................guuucUuacguagcagagcagcucccucgc................................................................    1          1          7y1
...................guuucguacguagAagagcagcucccucgc................................................................    1          1          7y1
...................guuucguacguagAagagcagcucccucgcu...............................................................    1          1          7y1
...................guuucguaGguagcagagcagcucccucgcu...............................................................    1          1          7y1
...................guuucUuacguagcagagcagcucccucgcu...............................................................    1          1          7y1
...................gAuucguacguagcagagcagcucccucgcu...............................................................    2          1          7y1
...................Cuuucguacguagcagagcagcucccucgcu...............................................................    2          1          7y1
...................gAuucguacguagcagagcagcucccucgcug..............................................................    1          1          7y1
...................guuucguacguagAagagcagcucccucgcug..............................................................    1          1          7y1
...................guuucguacUuagcagagcagcucccucgcug..............................................................    1          1          7y1
....................uuucguacgCagcagagc...........................................................................    1          1          7y1
....................uuucguacguagcagaUc...........................................................................    5          1          7y1
....................Auucguacguagcagagc...........................................................................    52         1          7y1
....................uuAcguacguagcagagc...........................................................................    2          1          7y1
....................uuucCuacguagcagagc...........................................................................    192        1          7y1
....................uuucguacguaCcagagc...........................................................................    1          1          7y1
....................uuucguacgAagcagagc...........................................................................    11         1          7y1
....................uuucguacguagcGgagc...........................................................................    2          1          7y1
....................uuucgGacguagcagagc...........................................................................    1          1          7y1
....................Guucguacguagcagagc...........................................................................    9          1          7y1
....................uuucguacguaUcagagc...........................................................................    2          1          7y1
....................Nuucguacguagcagagc...........................................................................    3          1          7y1
....................uuucguacguagcagagc...........................................................................    4344       0          7y1
....................uuucAuacguagcagagc...........................................................................    1          1          7y1
....................uuucguacguagcUgagc...........................................................................    2          1          7y1
....................uuucUuacguagcagagc...........................................................................    1          1          7y1
....................uuucgAacguagcagagc...........................................................................    8          1          7y1
....................Cuucguacguagcagagc...........................................................................    2          1          7y1
....................uuucguacCuagcagagc...........................................................................    4          1          7y1
....................uuucguacguagcaUagc...........................................................................    1          1          7y1
....................uAucguacguagcagagc...........................................................................    9          1          7y1
....................uuuAguacguagcagagc...........................................................................    5          1          7y1
....................uuucguacguagcagaCc...........................................................................    2          1          7y1
....................uuucguacUuagcagagc...........................................................................    1          1          7y1
....................uuucguacguGgcagagc...........................................................................    2          1          7y1
....................uuucguaUguagcagagc...........................................................................    1          1          7y1
....................uuucguacguagcagagA...........................................................................    7          1          7y1
....................uuucguacguagAagagc...........................................................................    4          1          7y1
....................uuuGguacguagcagagc...........................................................................    3          1          7y1
....................uuucguaAguagcagagc...........................................................................    8          1          7y1
....................uuucguaGguagcagagca..........................................................................    1          1          7y1
```

**Star** **Mature**

| | | | |
|---|---|---|---|
| gaccugcuucugggucgggguuucguacguagcagagcagcucccucgcugcgaucuauugaaagucagcccucgacacaaggguuuguccgcgcgcgcgcgcgcgugcgugc | | | |
| ....................uuucguacguagcagagAa.......................................................................... | 4 | 1 | 7y1 |
| ....................uuucguacguagAagagca.......................................................................... | 3 | 1 | 7y1 |
| ....................uCucguacguagcagagca.......................................................................... | 1 | 1 | 7y1 |
| ....................uuucguacguagcagCgca.......................................................................... | 3 | 1 | 7y1 |
| ....................uuuAguacguagcagagca.......................................................................... | 2 | 1 | 7y1 |
| ....................uuucguaAguagcagagca.......................................................................... | 2 | 1 | 7y1 |
| ....................Nuucguacguagcagagca.......................................................................... | 3 | 1 | 7y1 |
| ....................Auucguacguagcagagca.......................................................................... | 24 | 1 | 7y1 |
| ....................uuucguacguagcagaUca.......................................................................... | 1 | 1 | 7y1 |
| ....................uuucguacguaCcagagca.......................................................................... | 1 | 1 | 7y1 |
| ....................uuucguacguagcagagca.......................................................................... | 1926 | 0 | 7y1 |
| ....................uuucguGcguagcagagca.......................................................................... | 1 | 1 | 7y1 |
| ....................uuucguacguUgcagagca.......................................................................... | 2 | 1 | 7y1 |
| ....................uuucguacguagcagaCca.......................................................................... | 3 | 1 | 7y1 |
| ....................uuucguacguagcagagGa.......................................................................... | 1 | 1 | 7y1 |
| ....................uuucguacguagUagagca.......................................................................... | 1 | 1 | 7y1 |
| ....................uuucgAacguagcagagca.......................................................................... | 2 | 1 | 7y1 |
| ....................uuucguacguaUcagagca.......................................................................... | 2 | 1 | 7y1 |
| ....................uuucguacCuagcagagca.......................................................................... | 2 | 1 | 7y1 |
| ....................uuucguacgGagcagagca.......................................................................... | 1 | 1 | 7y1 |
| ....................uuucguacguagcGgagca.......................................................................... | 1 | 1 | 7y1 |
| ....................uuucCuacguagcagagca.......................................................................... | 1 | 1 | 7y1 |
| ....................uuucguCcguagcagagca.......................................................................... | 1 | 1 | 7y1 |
| ....................uuucguacUuagcagagca.......................................................................... | 1 | 1 | 7y1 |
| ....................uuucguacgCagcagagca.......................................................................... | 1 | 1 | 7y1 |
| ....................uuucguacguagcUgagca.......................................................................... | 1 | 1 | 7y1 |
| ....................Guucguacguagcagagca.......................................................................... | 4 | 1 | 7y1 |
| ....................uuucgCacguagcagagca.......................................................................... | 1 | 1 | 7y1 |
| ....................uAucguacguagcagagca.......................................................................... | 5 | 1 | 7y1 |
| ....................uuucguacguagcaUagca.......................................................................... | 1 | 1 | 7y1 |
| ....................uuucguacguagcaUagcag......................................................................... | 1 | 1 | 7y1 |
| ....................uuucguacguagcagagcaU......................................................................... | 2 | 1 | 7y1 |
| ....................uuCcguacguagcagagcag......................................................................... | 1 | 1 | 7y1 |
| ....................uuucguacgAagcagagcag......................................................................... | 1 | 1 | 7y1 |
| ....................uuAcguacguagcagagcag......................................................................... | 1 | 1 | 7y1 |
| ....................uAucguacguagcagagcag......................................................................... | 4 | 1 | 7y1 |
| ....................uuucguacguagcagagAag......................................................................... | 1 | 1 | 7y1 |
| ....................uuucguacguagcGgagcag......................................................................... | 1 | 1 | 7y1 |
| ....................uuucguacguagcagaUcag......................................................................... | 1 | 1 | 7y1 |
| ....................uuucguacguagcagagcaC......................................................................... | 2 | 1 | 7y1 |
| ....................Auucguacguagcagagcag......................................................................... | 11 | 1 | 7y1 |
| ....................uuucgCacguagcagagcag......................................................................... | 1 | 1 | 7y1 |
| ....................uuucguacguagcagagcag......................................................................... | 641 | 0 | 7y1 |
| ....................uuucguacguagcagagGagc........................................................................ | 2 | 1 | 7y1 |
| ....................uuucgAacguagcagagcagc........................................................................ | 3 | 1 | 7y1 |
| ....................uuucgGacguagcagagcagc........................................................................ | 3 | 1 | 7y1 |
| ....................uuucguGcguagcagagcagc........................................................................ | 1 | 1 | 7y1 |
| ....................uuucguaAguagcagagcagc........................................................................ | 5 | 1 | 7y1 |
| ....................uuucguaGguagcagagcagc........................................................................ | 1 | 1 | 7y1 |
| ....................uuucguacguagcagagcaAc........................................................................ | 1 | 1 | 7y1 |
| ....................uuucguaUguagcagagcagc........................................................................ | 1 | 1 | 7y1 |
| ....................uuucguacguagAagagcagc........................................................................ | 2 | 1 | 7y1 |
| ....................uuucguCcguagcagagcagc........................................................................ | 1 | 1 | 7y1 |
| ....................uuucguacguagcagagcagc........................................................................ | 2127 | 0 | 7y1 |
| ....................Cuucguacguagcagagcagc........................................................................ | 237 | 1 | 7y1 |
| ....................uuucgCacguagcagagcagc........................................................................ | 1 | 1 | 7y1 |
| ....................Auucguacguagcagagcagc........................................................................ | 18 | 1 | 7y1 |
| ....................uuucguacgAagcagagcagc........................................................................ | 2 | 1 | 7y1 |
| ....................uuucguacguagUagagcagc........................................................................ | 1 | 1 | 7y1 |
| ....................uuucguacguagcGgagcagc........................................................................ | 1 | 1 | 7y1 |
| ....................uuucguacUuagcagagcagc........................................................................ | 1 | 1 | 7y1 |
| ....................uAucguacguagcagagcagc........................................................................ | 5 | 1 | 7y1 |
| ....................Nuucguacguagcagagcagc........................................................................ | 1 | 1 | 7y1 |
| ....................uuucguacguagcagagcaCc........................................................................ | 1 | 1 | 7y1 |
| ....................uuucguacguagcagagcaUc........................................................................ | 1 | 1 | 7y1 |
| ....................uuucguacguagGagagcagc........................................................................ | 1 | 1 | 7y1 |
| ....................uuucguacguagcagagAagc........................................................................ | 3 | 1 | 7y1 |
| ....................uuucguacguagcaUagcagc........................................................................ | 1 | 1 | 7y1 |
| ....................uuucguacguagcagagcagA........................................................................ | 1 | 1 | 7y1 |
| ....................uuuGguacguagcagagcagc........................................................................ | 2 | 1 | 7y1 |

Star Mature

```
gaccugcuucugggucgggguuucguacguagcagagcagcucccucgcugcgaucuauugaaagucagcccucgacacaaggguuuguccgcgcgcgcgcgcgugcgugc
....................Guucguacguagcagagcagc......................................................................	3	1	7y1
....................uuucguacguagcagCgcagc......................................................................	5	1	7y1
....................uuucguacguagcUgagcagcu.....................................................................	1	1	7y1
....................uuucguacUuagcagagcagcu.....................................................................	1	1	7y1
....................uuucguacguagcagGgcagcu.....................................................................	1	1	7y1
....................uuucguacguagcaUagcagcu.....................................................................	272	1	7y1
....................uuucguacguagcagagcagAu.....................................................................	1	1	7y1
....................Nuucguacguagcagagcagcu.....................................................................	2	1	7y1
....................uuucguacguagcagagcagcC.....................................................................	1	1	7y1
....................uuucguacguagcagagGagcu.....................................................................	2	1	7y1
....................uAucguacguagcagagcagcu.....................................................................	10	1	7y1
....................uuucguacguaAcagagcagcu.....................................................................	1	1	7y1
....................uuAcguacguagcagagcagcu.....................................................................	3	1	7y1
....................uuucAuacguagcagagcagcu.....................................................................	2	1	7y1
....................uuucguaUguagcagagcagcu.....................................................................	1	1	7y1
....................uuucguacguagcagagAagcu.....................................................................	9	1	7y1
....................uuuUguacguagcagagcagcu.....................................................................	2	1	7y1
....................uuucguaAguagcagagcagcu.....................................................................	8	1	7y1
....................uuucguacAuagcagagcagcu.....................................................................	1	1	7y1
....................uuucguacguaCcagagcagcu.....................................................................	1	1	7y1
....................uuuAguacguagcagagcagcu.....................................................................	7	1	7y1
....................Cuucguacguagcagagcagcu.....................................................................	3	1	7y1
....................uuucguacguagAagagcagcu.....................................................................	2	1	7y1
....................uuucguacguagcagagcagcG.....................................................................	22	1	7y1
....................Auucguacguagcagagcagcu.....................................................................	37	1	7y1
....................uuucguacguagcagaCcagcu.....................................................................	1	1	7y1
....................uuucgAacguagcagagcagcu.....................................................................	4	1	7y1
....................uuucguacguagcagagcagcA.....................................................................	3	1	7y1
....................uuucguacCuagcagagcagcu.....................................................................	4	1	7y1
....................uuucguacguagcagCgcagcu.....................................................................	4	1	7y1
....................uuucguacguagcagagcagcu.....................................................................	4088	0	7y1
....................uuucguacguagcagagcaUcu.....................................................................	4	1	7y1
....................uuucguacguagcagagcCgcu.....................................................................	3	1	7y1
....................uuucguacguagUagagcagcu.....................................................................	1	1	7y1
....................uuucgCacguagcagagcagcu.....................................................................	1	1	7y1
....................uuucguacguagcagagcaCcu.....................................................................	1	1	7y1
....................uuucguGcguagcagagcagcu.....................................................................	1	1	7y1
....................uuucguacgAagcagagcagcu.....................................................................	11	1	7y1
....................Guucguacguagcagagcagcu.....................................................................	7	1	7y1
....................uuucguacguagcagUgcagcu.....................................................................	2	1	7y1
....................uuucguacguagcaCagcagcu.....................................................................	2	1	7y1
....................uuucCuacguagcagagcagcu.....................................................................	2	1	7y1
....................uuucguacguagcagagcagGuc....................................................................	1	1	7y1
....................uuucguacguagcagagcagcuc....................................................................	7935	0	7y1
....................uuucAuacguagcagagcagcuc....................................................................	1	1	7y1
....................uuucguacguagcagagAagcuc....................................................................	10	1	7y1
....................uuucguacguagcagagcUgcuc....................................................................	1	1	7y1
....................uuucUuacguagcagagcagcuc....................................................................	6	1	7y1
....................uuucguacguagcUgagcagcuc....................................................................	1	1	7y1
....................uuucguacguagcagUgcagcuc....................................................................	1	1	7y1
....................uuucgAacguagcagagcagcuc....................................................................	16	1	7y1
....................uuucguacguagcagagcCgcuc....................................................................	3	1	7y1
....................uuucguacguagcagagcagcuA....................................................................	8	1	7y1
....................uuucguacguagcagaUcagcuc....................................................................	3	1	7y1
....................uuucguacguagcagaCcagcuc....................................................................	4	1	7y1
....................Auucguacguagcagagcagcuc....................................................................	89	1	7y1
....................uuucguacguagcagagcGgcuc....................................................................	2	1	7y1
....................uuucguacguagcagagcaCcuc....................................................................	4	1	7y1
....................uuuUguacguagcagagcagcuc....................................................................	1	1	7y1
....................uuucguacguaCcagagcagcuc....................................................................	2	1	7y1
....................uuucguacguagcagagGagcuc....................................................................	1	1	7y1
....................uuucguacgCagcagagcagcuc....................................................................	1	1	7y1
....................uuucguacguagcagGgcagcuc....................................................................	1	1	7y1
....................uuucguacguagAagagcagcuc....................................................................	9	1	7y1
....................uuGcguacguagcagagcagcuc....................................................................	1	1	7y1
....................uuucguacguagcaCagcagcuc....................................................................	1	1	7y1
....................uuuAguacguagcagagcagcuc....................................................................	12	1	7y1
....................uuucCuacguagcagagcagcuc....................................................................	2	1	7y1
....................uuucguacCuagcagagcagcuc....................................................................	4	1	7y1
....................uuucguacguagcagagcagcGc....................................................................	1	1	7y1
```

**Star** **Mature**

```
gaccugcuucugggucgggguuucguacguagcagagcagcucccucgcugcgaucuauugaaagucagcccucgacacaaggguuuguccgcgcgcgcgcgcgcgugcgugc
....................uuucguacguagcagagcagcuG......................................................................  1     1  7y1
....................uuucguacguagcCgagcagcuc......................................................................  1     1  7y1
....................uuucguacAuagcagagcagcuc......................................................................  1     1  7y1
....................Guucguacguagcagagcagcuc......................................................................  13    1  7y1
....................uuucguaAguagcagagcagcuc......................................................................  9     1  7y1
....................uuucguacguagcagagcagcAc......................................................................  2     1  7y1
....................uuCcguacguagcagagcagcuc......................................................................  1     1  7y1
....................uuucguacguagcagagcaUcuc......................................................................  7     1  7y1
....................uuucguacguGgcagagcagcuc......................................................................  2     1  7y1
....................uuucguacguagcGgagcagcuc......................................................................  1     1  7y1
....................uuucguacguagcagagcagcuU......................................................................  1     1  7y1
....................uuucgCacguagcagagcagcuc......................................................................  1     1  7y1
....................uuucguacgAagcagagcagcuc......................................................................  11    1  7y1
....................uuucguacUuagcagagcagcuc......................................................................  3     1  7y1
....................uuucguaUguagcagagcagcuc......................................................................  1     1  7y1
....................uuucguacguagcagCgcagcuc......................................................................  7     1  7y1
....................uuuGguacguagcagagcagcuc......................................................................  3     1  7y1
....................uuucguCcguagcagagcagcuc......................................................................  2     1  7y1
....................uuucguacguagcagagcagAuc......................................................................  5     1  7y1
....................Nuucguacguagcagagcagcuc......................................................................  4     1  7y1
....................uAucguacguagcagagcagcuc......................................................................  11    1  7y1
....................uuucguacguaUcagagcagcuc......................................................................  1     1  7y1
....................Cuucguacguagcagagcagcuc......................................................................  175   1  7y1
....................uuucguGcguagcagagcagcuc......................................................................  1     1  7y1
....................uuucguacguagUagagcagcuc......................................................................  2     1  7y1
....................uuucguacguagcaUagcagcuc......................................................................  2     1  7y1
....................uuAcguacguagcagagcagcuc......................................................................  3     1  7y1
....................uCucguacguagcagagcagcuc......................................................................  1     1  7y1
....................uuucguacguagcagagcagcucG.....................................................................  1     1  7y1
....................uuucguacguagcagGgcagcucc.....................................................................  4     1  7y1
....................uuucguacgGagcagagcagcucc.....................................................................  1     1  7y1
....................uuucguacguagcagagcagcuUc.....................................................................  1     1  7y1
....................uuucguaAguagcagagcagcucc.....................................................................  7     1  7y1
....................uuucguacguagcagagcagcAcc.....................................................................  1     1  7y1
....................uuucguacguagcaUagcagcucc.....................................................................  1     1  7y1
....................uuucguacCuagcagagcagcucc.....................................................................  4     1  7y1
....................uuucguacguagcaCagcagcucc.....................................................................  1     1  7y1
....................uuucguacguaUcagagcagcucc.....................................................................  3     1  7y1
....................uuucguacguagcagagcCgcucc.....................................................................  1     1  7y1
....................uuucguacguagcagagcaUcucc.....................................................................  3     1  7y1
....................uuucCuacguagcagagcagcucc.....................................................................  1     1  7y1
....................uuucguacguagcagagcagcucc.....................................................................  5981  0  7y1
....................uuucguacguagcagaCcagcucc.....................................................................  3     1  7y1
....................uuucguacguagcagagcagcucU.....................................................................  2     1  7y1
....................Auucguacguagcagagcagcucc.....................................................................  67    1  7y1
....................uuucguacguagcagagcGgcucc.....................................................................  1     1  7y1
....................uuucguacguagcagagcagcCcc.....................................................................  2     1  7y1
....................Nuucguacguagcagagcagcucc.....................................................................  3     1  7y1
....................uuGguacguagcagagcagcucc......................................................................  2     1  7y1
....................uuucguacUuagcagagcagcucc.....................................................................  2     1  7y1
....................uuucguacguagcagCgcagcucc.....................................................................  5     1  7y1
....................uuucguacguagcagagcagcucA.....................................................................  2     1  7y1
....................uuucguacgAagcagagcagcucc.....................................................................  7     1  7y1
....................uuucUuacguagcagagcagcucc.....................................................................  2     1  7y1
....................uuucgCacguagcagagcagcucc.....................................................................  2     1  7y1
....................uuAcguacguagcagagcagcucc.....................................................................  1     1  7y1
....................uuCcguacguagcagagcagcucc.....................................................................  2     1  7y1
....................uuGcguacguagcagagcagcucc.....................................................................  1     1  7y1
....................Guucguacguagcagagcagcucc.....................................................................  14    1  7y1
....................uuucgAacguagcagagcagcucc.....................................................................  11    1  7y1
....................uuucguacguagcagagcagAucc.....................................................................  4     1  7y1
....................uuucguacguagcagagcaCcucc.....................................................................  3     1  7y1
....................uuucguacguUgcagagcagcucc.....................................................................  1     1  7y1
....................uAucguacguagcagagcagcucc.....................................................................  9     1  7y1
....................uuucguacgCagcagagcagcucc.....................................................................  1     1  7y1
....................uuucguacguagAagagcagcucc.....................................................................  9     1  7y1
....................uuucguacguagcGgagcagcucc.....................................................................  1     1  7y1
....................uuucguacguagcagagAagcucc.....................................................................  7     1  7y1
....................uuucguaGguagcagagcagcucc.....................................................................  2     1  7y1
....................uuucguaUguagcagagcagcucc.....................................................................  1     1  7y1
```

**Star** **Mature**

```
gaccugcuucugggucggggguuucguacguagcagagcagcucccucgcugcgaucuauugaaagucagcccucgacacaaggguuuguccgcgcgcgcgcgcgcgugcgugc
....................uuucguacguagcagagcagcuAc.....................................................................    15     1    7y1
....................Cuucguacguagcagagcagcucc.....................................................................    1      1    7y1
....................uuucguUcguagcagagcagcucc.....................................................................    2      1    7y1
....................uuuAguacguagcagagcagcucc.....................................................................    6      1    7y1
....................uNucguacguagcagagcagcucc.....................................................................    1      1    7y1
....................uuucguacguagcagaUcagcucc.....................................................................    1      1    7y1
....................uuuUguacguagcagagcagcucc.....................................................................    1      1    7y1
....................uuucguacguagcagagcaAcucc.....................................................................    1      1    7y1
....................uuucguacguagcagUgcagcucc.....................................................................    3      1    7y1
....................uuucguacguagcagagcagAuccc....................................................................    1      1    7y1
....................uuucUuacguagcagagcagcuccc....................................................................    1      1    7y1
....................uuucguacguagcagagcagGuccc....................................................................    1      1    7y1
....................uuucguacCuagcagagcagcuccc....................................................................    1      1    7y1
....................uuucguacguagcagUgcagcuccc....................................................................    2      1    7y1
....................uuucguacguagcagagcagcuccA....................................................................    4      1    7y1
....................uuucguacguagUagagcagcuccc....................................................................    277    1    7y1
....................uuuGguacguagcagagcagcuccc....................................................................    3      1    7y1
....................uuucguacguagcagaCcagcuccc....................................................................    1      1    7y1
....................uuucguacguagcagagcagcAccc....................................................................    1      1    7y1
....................uuucguacguagcGgagcagcuccc....................................................................    1      1    7y1
....................uuucguaAguagcagagcagcuccc....................................................................    3      1    7y1
....................uuucAuacguagcagagcagcuccc....................................................................    1      1    7y1
....................Auucguacguagcagagcagcuccc....................................................................    17     1    7y1
....................uuucguacguagcagCgcagcuccc....................................................................    4      1    7y1
....................uuucguacguagcagagcaUcuccc....................................................................    3      1    7y1
....................uuucCuacguagcagagcagcuccc....................................................................    1      1    7y1
....................uAucguacguagcagagcagcuccc....................................................................    3      1    7y1
....................uuucguacguagcagagcagcuccG....................................................................    1      1    7y1
....................uuucguacUuagcagagcagcuccc....................................................................    1      1    7y1
....................uuucgCacguagcagagcagcuccc....................................................................    1      1    7y1
....................uuucguacguagcagagcagcuAcc....................................................................    11     1    7y1
....................uuucguacguagcagagAagcuccc....................................................................    2      1    7y1
....................uuucguacguagcagagcaCcuccc....................................................................    2      1    7y1
....................Guucguacguagcagagcagcuccc....................................................................    2      1    7y1
....................uuucguaGguagcagagcagcuccc....................................................................    1      1    7y1
....................uuucguacguagAagagcagcuccc....................................................................    2      1    7y1
....................uuucguacguagcagagcagcuccc....................................................................    2072   0    7y1
....................uuucguacgAagcagagcagcuccc....................................................................    3      1    7y1
....................uuucguacguaUcagagcagcuccc....................................................................    1      1    7y1
....................uuuAguacguagcagagcagcuccc....................................................................    2      1    7y1
....................uuAcguacguagcagagcagcuccc....................................................................    2      1    7y1
....................uuucguacguagGagagcagcuccc....................................................................    1      1    7y1
....................uuucgAacguagcagagcagcuccc....................................................................    4      1    7y1
....................uuucguacguaCcagagcagcucccu...................................................................    1      1    7y1
....................uuucguacguagcagagcCgcucccu...................................................................    1      1    7y1
....................uuucguacguagcagagcagAucccu...................................................................    2      1    7y1
....................uuucUuacguagcagagcagcucccu...................................................................    3      1    7y1
....................uuucguacguagcagaUcagcucccu...................................................................    1      1    7y1
....................uAucguacguagcagagcagcucccu...................................................................    9      1    7y1
....................uuucguacguagcagagcagcucccC...................................................................    152    1    7y1
....................uuucguacguagcagagcGgcucccu...................................................................    1      1    7y1
....................uCucguacguagcagagcagcucccu...................................................................    1      1    7y1
....................uuucguacguagcUgagcagcucccu...................................................................    1      1    7y1
....................uuucguacguagcGgagcagcucccu...................................................................    1      1    7y1
....................uuucguacguagcagagAagcucccu...................................................................    6      1    7y1
....................uGucguacguagcagagcagcucccu...................................................................    1      1    7y1
....................uuucguacUuagcagagcagcucccu...................................................................    7      1    7y1
....................uuucguacguaAcagagcagcucccu...................................................................    2      1    7y1
....................uuucCuacguagcagagcagcucccu...................................................................    1      1    7y1
....................uuucguacCuagcagagcagcucccu...................................................................    1      1    7y1
....................uuucguacguGgcagagcagcucccu...................................................................    2      1    7y1
....................Auucguacguagcagagcagcucccu...................................................................    42     1    7y1
....................uuucguacguaUcagagcagcucccu...................................................................    1      1    7y1
....................uuuAguacguagcagagcagcucccu...................................................................    6      1    7y1
....................uuucguacguagcagGgcagcucccu...................................................................    2      1    7y1
....................uuucguacguagcaUagcagcucccu...................................................................    1      1    7y1
....................uuucguacguagcagagcagcucccu...................................................................    4551   0    7y1
....................uuucguacgAagcagagcagcucccu...................................................................    7      1    7y1
....................uuAcguacguagcagagcagcucccu...................................................................    4      1    7y1
....................uuucguacguagcagagcagcAcccu...................................................................    1      1    7y1
```

**Star** **Mature**

| | | | |
|---|---|---|---|
| gaccugcuucugggucggggguuucguacguagcagagcagcucccucgcugcgaucuauugaaagucagcccucgacacaaggguuuguccgcgcgcgcgcgcgcgugcgugc | | | |
| ....................uuucguGcguagcagagcagcucccu................................................................... | 1 | 1 | 7y1 |
| ....................uuucguacguagcagagcagcCcccu................................................................... | 1 | 1 | 7y1 |
| ....................uuucguacAuagcagagcagcucccu................................................................... | 3 | 1 | 7y1 |
| ....................Guucguacguagcagagcagcucccu................................................................... | 14 | 1 | 7y1 |
| ....................uuucguacguagcaAagcagcucccu................................................................... | 1 | 1 | 7y1 |
| ....................uuucguaUguagcagagcagcucccu................................................................... | 2 | 1 | 7y1 |
| ....................uuucguacguagcagagcagcuccAu................................................................... | 4 | 1 | 7y1 |
| ....................uuucguacguagcagagcagcucccG................................................................... | 17 | 1 | 7y1 |
| ....................uuucgAacguagcagagcagcucccu................................................................... | 9 | 1 | 7y1 |
| ....................uuGcguacguagcagagcagcucccu................................................................... | 1 | 1 | 7y1 |
| ....................uuucguacguagcagagcagcucccA................................................................... | 8 | 1 | 7y1 |
| ....................uuucguaAguagcagagcagcucccu................................................................... | 4 | 1 | 7y1 |
| ....................uuucguacguagUagagcagcucccu................................................................... | 225 | 1 | 7y1 |
| ....................uuucguacguagcagagcagcucAcu................................................................... | 3 | 1 | 7y1 |
| ....................uuucguacguagAagagcagcucccu................................................................... | 2 | 1 | 7y1 |
| ....................Nuucguacguagcagagcagcucccu................................................................... | 1 | 1 | 7y1 |
| ....................uuucguacguagcagCgcagcucccu................................................................... | 10 | 1 | 7y1 |
| ....................Cuucguacguagcagagcagcucccu................................................................... | 2 | 1 | 7y1 |
| ....................uuucguacguagcagaCcagcucccu................................................................... | 2 | 1 | 7y1 |
| ....................uuucguacguagcagagcagcuAccu................................................................... | 10 | 1 | 7y1 |
| ....................uuAcguacguagcagagcagcucccuc.................................................................. | 3 | 1 | 7y1 |
| ....................uuucCuacguagcagagcagcucccuc.................................................................. | 2 | 1 | 7y1 |
| ....................uuucguacguagcagagAagcucccuc.................................................................. | 6 | 1 | 7y1 |
| ....................uuucguacguagcagagcaCcucccuc.................................................................. | 1 | 1 | 7y1 |
| ....................Cuucguacguagcagagcagcucccuc.................................................................. | 1 | 1 | 7y1 |
| ....................uuucguacguagcagCgcagcucccuc.................................................................. | 10 | 1 | 7y1 |
| ....................uuucguacguagcagagcagcuAccuc.................................................................. | 7 | 1 | 7y1 |
| ....................uuucguacguagcagagcCgcucccuc.................................................................. | 1 | 1 | 7y1 |
| ....................Nuucguacguagcagagcagcucccuc.................................................................. | 6 | 1 | 7y1 |
| ....................uuucguacguagcagagcagcCcccuc.................................................................. | 1 | 1 | 7y1 |
| ....................uuucguacguaAcagagcagcucccuc.................................................................. | 1 | 1 | 7y1 |
| ....................uuucguacguagcagUgcagcucccuc.................................................................. | 2 | 1 | 7y1 |
| ....................uuucguacguagcagagcaUcucccuc.................................................................. | 2 | 1 | 7y1 |
| ....................uuucguacguagcagagcagcuccAuc.................................................................. | 5 | 1 | 7y1 |
| ....................uuucguacguagcaUagcagcucccuc.................................................................. | 2 | 1 | 7y1 |
| ....................uuucgCacguagcagagcagcucccuc.................................................................. | 1 | 1 | 7y1 |
| ....................uuucguacguagcagagcGgcucccuc.................................................................. | 2 | 1 | 7y1 |
| ....................uuucguaUguagcagagcagcucccuc.................................................................. | 1 | 1 | 7y1 |
| ....................uuucguacguagcagagcagcuGccuc.................................................................. | 1 | 1 | 7y1 |
| ....................uuucguacguagcagagcagcucAcuc.................................................................. | 6 | 1 | 7y1 |
| ....................uuucguacguaUcagagcagcucccuc.................................................................. | 1 | 1 | 7y1 |
| ....................uuucguacguagcagagcagAucccuc.................................................................. | 4 | 1 | 7y1 |
| ....................uuucguacguagcagagcagcucccuc.................................................................. | 4379 | 0 | 7y1 |
| ....................uuucUuacguagcagagcagcucccuc.................................................................. | 2 | 1 | 7y1 |
| ....................uuucguacguagcagagcUgcucccuc.................................................................. | 1 | 1 | 7y1 |
| ....................uuucguacguagAagagcagcucccuc.................................................................. | 5 | 1 | 7y1 |
| ....................uAucguacguagcagagcagcucccuc.................................................................. | 9 | 1 | 7y1 |
| ....................uuucguacguagUagagcagcucccuc.................................................................. | 1 | 1 | 7y1 |
| ....................Guucguacguagcagagcagcucccuc.................................................................. | 12 | 1 | 7y1 |
| ....................Auucguacguagcagagcagcucccuc.................................................................. | 39 | 1 | 7y1 |
| ....................uuucgAacguagcagagcagcucccuc.................................................................. | 9 | 1 | 7y1 |
| ....................uuucguacgAagcagagcagcucccuc.................................................................. | 7 | 1 | 7y1 |
| ....................uuuGguacguagcagagcagcucccuc.................................................................. | 1 | 1 | 7y1 |
| ....................uuucguaAguagcagagcagcucccuc.................................................................. | 3 | 1 | 7y1 |
| ....................uuucguacguagcagagcagcucccuA.................................................................. | 3 | 1 | 7y1 |
| ....................uuucguacguagcagagGagcucccuc.................................................................. | 1 | 1 | 7y1 |
| ....................uuucguacguagcagagcagcucUcuc.................................................................. | 1 | 1 | 7y1 |
| ....................uuucguacguagcaCagcagcucccuc.................................................................. | 2 | 1 | 7y1 |
| ....................uuucguacguagcagagcagcucccAc.................................................................. | 4 | 1 | 7y1 |
| ....................uuucguacguagcagagcagcAcccuc.................................................................. | 2 | 1 | 7y1 |
| ....................uuuAguacguagcagagcagcucccuc.................................................................. | 11 | 1 | 7y1 |
| ....................uuucguacguagcagagcagcuUccuc.................................................................. | 1 | 1 | 7y1 |
| ....................uuucguacguagcagagcagcucGcuc.................................................................. | 2 | 1 | 7y1 |
| ....................uuucguacCuagcagagcagcucccuc.................................................................. | 1 | 1 | 7y1 |
| ....................uuucguacUuagcagagcagcucccuc.................................................................. | 2 | 1 | 7y1 |
| ....................uuAcguacguagcagagcagcucccucgc................................................................ | 1 | 1 | 7y1 |
| ....................uuucguacguagcagCgcagcucccucgc................................................................ | 1 | 1 | 7y1 |
| ....................uuuAguacguagcagagcagcucccucgc................................................................ | 1 | 1 | 7y1 |
| ....................uuucguacguagUagagcagcucccucgc................................................................ | 1 | 1 | 7y1 |
| ....................uuucguacguagcagagcagcucAcucgc................................................................ | 1 | 1 | 7y1 |

```
    Star                                                                    Mature
gaccugcuucugggucgggguuucguacguagcagagcagcucccucgcugcgaucuauugaaagucagcccucgacacaaggguuuguccgcgcgcgcgcgcgcgugcgugc
....................uuucguacguagcagaUcagcucccucgc................................................................    1      1      7y1
....................uuucguacguagcagagAagcucccucgc................................................................    1      1      7y1
....................uuuGguacguagcagagcagcucccucgc................................................................    1      1      7y1
....................uuucguacguagcagagcagcucccAcgc................................................................    1      1      7y1
....................uuucguacguagcagagcUgcucccucgc................................................................    1      1      7y1
....................Guucguacguagcagagcagcucccucgc................................................................    2      1      7y1
....................uuucguacguagcagagcaCcucccucgc................................................................    1      1      7y1
....................uuucguacguagcagagcagcucccucgc................................................................    614    0      7y1
....................uuucguacgAagcagagcagcucccucgc................................................................    1      1      7y1
....................uAucguacguagcagagcagcucccucgc................................................................    1      1      7y1
....................uuucgAacguagcagagcagcucccucgc................................................................    1      1      7y1
....................uuucguacCuagcagagcagcucccucgc................................................................    1      1      7y1
....................uuucguacguagcagagcagcuAccucgc................................................................    1      1      7y1
....................Auucguacguagcagagcagcucccucgc................................................................    4      1      7y1
....................uuuAguacguagcagagcagcucccucgcu..............................................................    4      1      7y1
....................uuucguacguagGagagcagcucccucgcu..............................................................    1      1      7y1
....................uuucguacguagcagagcagcucccuGgcu..............................................................    1      1      7y1
....................uuucguacguagcagaCcagcucccucgcu..............................................................    1      1      7y1
....................Auucguacguagcagagcagcucccucgcu..............................................................    20     1      7y1
....................uuucguacguagcagagcagcCcccucgcu..............................................................    1      1      7y1
....................uuucguacguagAagagcagcucccucgcu..............................................................    3      1      7y1
....................uuucguacCuagcagagcagcucccucgcu..............................................................    1      1      7y1
....................uuucguacguagcCgagcagcucccucgcu..............................................................    1      1      7y1
....................uuucguacguagcagagcCgcucccucgcu..............................................................    2      1      7y1
....................uuucguacguaCcagagcagcucccucgcu..............................................................    1      1      7y1
....................uuucguacguagcagagcagcucUcucgcu..............................................................    2      1      7y1
....................uuucguUcguagcagagcagcucccucgcu..............................................................    1      1      7y1
....................uuucguacguagcagagAagcucccucgcu..............................................................    2      1      7y1
....................uuucguacguagcagagcGgcucccucgcu..............................................................    1      1      7y1
....................uuucguacguagcagagcaUcucccucgcu..............................................................    1      1      7y1
....................uuucguacguagcagUgcagcucccucgcu..............................................................    1      1      7y1
....................uuucguacguagcagagcUgcucccucgcu..............................................................    1      1      7y1
....................uuucguacguagcagaUcagcucccucgcu..............................................................    1      1      7y1
....................uuucguacguagcagagcagcuAccucgcu..............................................................    7      1      7y1
....................uuucguacguagcagagcagcucccuAgcu..............................................................    3      1      7y1
....................uuucguacguagcagGgcagcucccucgcu..............................................................    2      1      7y1
....................uuucguacguGgcagagcagcucccucgcu..............................................................    1      1      7y1
....................uuucgAacguagcagagcagcucccucgcu..............................................................    4      1      7y1
....................uuucguacguagcagagcagcucccucgcu..............................................................    2277   0      7y1
....................uuucguaAguagcagagcagcucccucgcu..............................................................    2      1      7y1
....................Guucguacguagcagagcagcucccucgcu..............................................................    178    1      7y1
....................uAucguacguagcagagcagcucccucgcu..............................................................    1      1      7y1
....................uuucguacguagcagagcagcucGcucgcu..............................................................    1      1      7y1
....................uuGcguacguagcagagcagcucccucgcu..............................................................    1      1      7y1
....................uuAcguacguagcagagcagcucccucgcu..............................................................    1      1      7y1
....................uuucguacguagcagCgcagcucccucgcu..............................................................    2      1      7y1
....................uuucguacgCagcagagcagcucccucgcu..............................................................    1      1      7y1
....................uuucguacgAagcagagcagcucccucgcu..............................................................    6      1      7y1
....................uuucguacguagcaCagcagcucccucgcu..............................................................    2      1      7y1
....................uuucguacUuagcagagcagcucccucgcu..............................................................    1      1      7y1
....................uuucguacguagcagagcagUucccucgcu..............................................................    1      1      7y1
....................uuucguacguagcagagcagcucccucAcu..............................................................    2      1      7y1
....................uuucAuacguagcagagcagcucccucgcu..............................................................    2      1      7y1
....................uuucguacguagcagagcagcucccucgAu..............................................................    3      1      7y1
....................uuucguacguagcagagcagcucccucgcG..............................................................    20     1      7y1
....................Nuucguacguagcagagcagcucccucgcu..............................................................    2      1      7y1
....................uuucguacguagcagagcagcucccAcgcu..............................................................    2      1      7y1
....................uuucguacguagcagagcagcucAcucgcu..............................................................    1      1      7y1
....................uuucguacCuagcagagcagcucccucgcug.............................................................    1      1      7y1
....................uuucAuacguagcagagcagcucccucgcug.............................................................    1      1      7y1
....................uuucguacguagcUgagcagcucccucgcug.............................................................    1      1      7y1
....................uuucguacguagcagagcGgcucccucgcug.............................................................    1      1      7y1
....................uuucguacguagcagagcagcucccucCcug.............................................................    1      1      7y1
....................uuucguacguagcagagcagcucccucgcGg.............................................................    9      1      7y1
....................Auucguacguagcagagcagcucccucgcug.............................................................    6      1      7y1
....................uuucguacguagcagagcagcucccAcgcug.............................................................    1      1      7y1
....................uuucguUcguagcagagcagcucccucgcug.............................................................    1      1      7y1
....................uuucguacguagcagagcagcucccucgcug.............................................................    855    0      7y1
....................uuucguacguagcagagcagcucccucUcug.............................................................    1      1      7y1
....................uuucguacgAagcagagcagcucccucgcug.............................................................    1      1      7y1
```

**Star** **Mature**

| | | | |
|---|---|---|---|
| gaccugcuucugggucgggguuucguacguagcagagcagcucccucgcugcgaucuauugaaagucagcccucgacacaagguuuguccgcgcgcgcgcgcgcgugcgugc | | | |
| ....................uuucguacguagcagagcaAcucccucgcug............................................................. | 1 | 1 | 7y1 |
| ....................uuucUuacguagcagagcagcucccucgcug............................................................. | 1 | 1 | 7y1 |
| ....................uuucguacguagcagagcagcuAccucgcug............................................................. | 2 | 1 | 7y1 |
| ....................uuucguacguagcagagAagcucccucgcug............................................................. | 2 | 1 | 7y1 |
| ....................uAucguacguagcagagcagcucccucgcug............................................................. | 2 | 1 | 7y1 |
| ....................uuucguCcguagcagagcagcucccucgcug............................................................. | 1 | 1 | 7y1 |
| ....................uuucguacguagcagagcagcucccucgGug............................................................. | 1 | 1 | 7y1 |
| ....................uuucgAacguagcagagcagcucccucgcug............................................................. | 2 | 1 | 7y1 |
| ....................uuucguacguagAagagcagcucccucgcug............................................................. | 1 | 1 | 7y1 |
| ....................uuucguaAguagcagagcagcucccucgcug............................................................. | 1 | 1 | 7y1 |
| ....................uuucguacguagcagagcagAucccucgcugc............................................................ | 1 | 1 | 7y1 |
| ....................uAucguacguagcagagcagcucccucgcugc............................................................ | 1 | 1 | 7y1 |
| ....................uuucguacguagcagagcagcAcccucgcugc............................................................ | 1 | 1 | 7y1 |
| ....................uuucguacCuagcagagcagcucccucgcugc............................................................ | 1 | 1 | 7y1 |
| ....................Guucguacguagcagagcagcucccucgcugc............................................................ | 1 | 1 | 7y1 |
| ....................uuucguacguagcagagcagcucccucgcugc............................................................ | 499 | 0 | 7y1 |
| ....................uuucguacguagcagagcagcucccucgcuCc............................................................ | 1 | 1 | 7y1 |
| ....................uuucguacgAagcagagcagcucccucgcugc............................................................ | 1 | 1 | 7y1 |
| ....................Auucguacguagcagagcagcucccucgcugc............................................................ | 5 | 1 | 7y1 |
| ....................uuuAguacguagcagagcagcucccucgcugc............................................................ | 1 | 1 | 7y1 |
| ....................uuucguacguagcagagcagcuAccucgcugc............................................................ | 1 | 1 | 7y1 |
| ....................Nuucguacguagcagagcagcucccucgcugc............................................................ | 2 | 1 | 7y1 |
| ....................uuucguacguagcagagcagcucccucgAugc............................................................ | 1 | 1 | 7y1 |
| .....................uucguacguagcaUagca......................................................................... | 1 | 1 | 7y1 |
| .....................uucguacguagcaCagca......................................................................... | 1 | 1 | 7y1 |
| .....................uucguacguagcagagAa......................................................................... | 3 | 1 | 7y1 |
| .....................uucguacguaCcagagca......................................................................... | 1 | 1 | 7y1 |
| .....................uucguacguaAcagagca......................................................................... | 1 | 1 | 7y1 |
| .....................uucguacguagcagGgca......................................................................... | 1 | 1 | 7y1 |
| .....................uucguacguagcagagGa......................................................................... | 1 | 1 | 7y1 |
| .....................uucguacguagAagagca......................................................................... | 1 | 1 | 7y1 |
| .....................uucguacgAagcagagca......................................................................... | 3 | 1 | 7y1 |
| .....................uucAuacguagcagagca......................................................................... | 1 | 1 | 7y1 |
| .....................uAcguacguagcagagca......................................................................... | 4 | 1 | 7y1 |
| .....................Nucguacguagcagagca......................................................................... | 1 | 1 | 7y1 |
| .....................uucguaAguagcagagca......................................................................... | 8 | 1 | 7y1 |
| .....................Gucguacguagcagagca......................................................................... | 2 | 1 | 7y1 |
| .....................uuAguacguagcagagca......................................................................... | 4 | 1 | 7y1 |
| .....................uucguacguagcagaAca......................................................................... | 1 | 1 | 7y1 |
| .....................uucguacguagcagagcG......................................................................... | 1 | 1 | 7y1 |
| .....................uNcguacguagcagagca......................................................................... | 1 | 1 | 7y1 |
| .....................Aucguacguagcagagca......................................................................... | 12 | 1 | 7y1 |
| .....................uucguacCuagcagagca......................................................................... | 2 | 1 | 7y1 |
| .....................uucguacguagcagagca......................................................................... | 1212 | 0 | 7y1 |
| .....................Cucguacguagcagagca......................................................................... | 1 | 1 | 7y1 |
| .....................uucguacguagcagagcC......................................................................... | 1 | 1 | 7y1 |
| .....................uucguacguagcagaUca......................................................................... | 2 | 1 | 7y1 |
| .....................uucguacAuagcagagcag........................................................................ | 1 | 1 | 7y1 |
| .....................uucguacguagcaCagcag........................................................................ | 1 | 1 | 7y1 |
| .....................Aucguacguagcagagcag........................................................................ | 16 | 1 | 7y1 |
| .....................uAcguacguagcagagcag........................................................................ | 3 | 1 | 7y1 |
| .....................Gucguacguagcagagcag........................................................................ | 2 | 1 | 7y1 |
| .....................uucguacguagcagagcag........................................................................ | 1378 | 0 | 7y1 |
| .....................uucguacguagAagagcag........................................................................ | 1 | 1 | 7y1 |
| .....................uucguaAguagcagagcag........................................................................ | 4 | 1 | 7y1 |
| .....................uucgCacguagcagagcag........................................................................ | 1 | 1 | 7y1 |
| .....................uucguacgAagcagagcag........................................................................ | 2 | 1 | 7y1 |
| .....................uucguacguagcagagAag........................................................................ | 1 | 1 | 7y1 |
| .....................uucguacguGgcagagcag........................................................................ | 1 | 1 | 7y1 |
| .....................uucguacguagcagagcaU........................................................................ | 2 | 1 | 7y1 |
| .....................uucguacCuagcagagcag........................................................................ | 1 | 1 | 7y1 |
| .....................uucguacguagcagagcUg........................................................................ | 1 | 1 | 7y1 |
| .....................uucguacguaUcagagcag........................................................................ | 1 | 1 | 7y1 |
| .....................uuAguacguagcagagcag........................................................................ | 4 | 1 | 7y1 |
| .....................uucguUcguagcagagcagc....................................................................... | 2 | 1 | 7y1 |
| .....................uucguacgAagcagagcagc....................................................................... | 1 | 1 | 7y1 |
| .....................uucguacAuagcagagcagc....................................................................... | 1 | 1 | 7y1 |
| .....................Nucguacguagcagagcagc....................................................................... | 1 | 1 | 7y1 |
| .....................uucguacUuagcagagcagc....................................................................... | 2 | 1 | 7y1 |
| .....................uucguaGguagcagagcagc....................................................................... | 2 | 1 | 7y1 |

```
Star                                                                  Mature
gaccugcuucugggucgggguuucguacguagcagagcagcucccucgcugcgaucuauugaaagucagcccucgacacaaggguuuguccgcgcgcgcgcgcgcgugcgugc
.....................uAcguacguagcagagcagc........................................................................ 2 1 7y1
.....................uucguacguagcaCagcagc........................................................................ 1 1 7y1
.....................uucCuacguagcagagcagc........................................................................ 1 1 7y1
.....................uucguacCuagcagagcagc........................................................................ 1 1 7y1
.....................uucguacguagcagagcaUc........................................................................ 3 1 7y1
.....................uucguacguagcagaUcagc........................................................................ 1 1 7y1
.....................uucguacguagcagaCcagc........................................................................ 1 1 7y1
.....................Aucguacguagcagagcagc........................................................................ 33 1 7y1
.....................uucguacguGgcagagcagc........................................................................ 2 1 7y1
.....................uucguacguagcagagcagA........................................................................ 2 1 7y1
.....................uuUguacguagcagagcagc........................................................................ 1 1 7y1
.....................uucguacguagcaUagcagc........................................................................ 1 1 7y1
.....................uucgAacguagcagagcagc........................................................................ 1 1 7y1
.....................uucguacguagcUgagcagc........................................................................ 1 1 7y1
.....................uucguaAguagcagagcagc........................................................................ 2 1 7y1
.....................uucguacguaAcagagcagc........................................................................ 1 1 7y1
.....................uCcguacguagcagagcagc........................................................................ 1 1 7y1
.....................uucguacgCagcagagcagc........................................................................ 1 1 7y1
.....................uucguacguagcaAagcagc........................................................................ 1 1 7y1
.....................uucguacguagcagagcagc........................................................................ 1790 0 7y1
.....................uucguacguagcagGgcagc........................................................................ 1 1 7y1
.....................uuAguacguagcagagcagc........................................................................ 5 1 7y1
.....................uucguacguagcagagcGgcu....................................................................... 5 1 7y1
.....................uucguacguagcagagAagcu....................................................................... 9 1 7y1
.....................uucguacguagUagagcagcu....................................................................... 185 1 7y1
.....................uucguacguagcagagcagcA....................................................................... 4 1 7y1
.....................uucguacguagcagagcagAu....................................................................... 4 1 7y1
.....................uucguacguagcGgagcagcu....................................................................... 1 1 7y1
.....................Nucguacguagcagagcagcu....................................................................... 2 1 7y1
.....................uucguGcguagcagagcagcu....................................................................... 1 1 7y1
.....................uucguacguaUcagagcagcu....................................................................... 3 1 7y1
.....................uucguacguagcUgagcagcu....................................................................... 2 1 7y1
.....................uucguacguagcagagcUgcu....................................................................... 1 1 7y1
.....................uucCuacguagcagagcagcu....................................................................... 1 1 7y1
.....................uucguacgAagcagagcagcu....................................................................... 8 1 7y1
.....................uucUuacguagcagagcagcu....................................................................... 5 1 7y1
.....................uucguacguagcagagcaUcu....................................................................... 3 1 7y1
.....................uucguacguagcagagcaAcu....................................................................... 1 1 7y1
.....................uucguacguagcagaCcagcu....................................................................... 5 1 7y1
.....................uuGguacguagcagagcagcu....................................................................... 2 1 7y1
.....................uucguacguagcagagcagGu....................................................................... 2 1 7y1
.....................uucguacguagcagGgcagcu....................................................................... 1 1 7y1
.....................uucguacguagcagagcaCcu....................................................................... 3 1 7y1
.....................uucgGacguagcagagcagcu....................................................................... 1 1 7y1
.....................uucguacguagcagagcagcu....................................................................... 7198 0 7y1
.....................Aucguacguagcagagcagcu....................................................................... 80 1 7y1
.....................uucguacguagcagagcagcG....................................................................... 40 1 7y1
.....................uucgCacguagcagagcagcu....................................................................... 3 1 7y1
.....................uucguacguagAagagcagcu....................................................................... 3 1 7y1
.....................uucguacguagcagCgcagcu....................................................................... 7 1 7y1
.....................uucguacguGgcagagcagcu....................................................................... 2 1 7y1
.....................uucgAacguagcagagcagcu....................................................................... 9 1 7y1
.....................uucguacguagcagagGagcu....................................................................... 4 1 7y1
.....................uucguacguagcagagcagcC....................................................................... 1 1 7y1
.....................uucguacguUgcagagcagcu....................................................................... 1 1 7y1
.....................uucguaUguagcagagcagcu....................................................................... 3 1 7y1
.....................Gucguacguagcagagcagcu....................................................................... 8 1 7y1
.....................uucguacCuagcagagcagcu....................................................................... 4 1 7y1
.....................uucguacguagcaAagcagcu....................................................................... 1 1 7y1
.....................uucguUcguagcagagcagcu....................................................................... 1 1 7y1
.....................uuAguacguagcagagcagcu....................................................................... 24 1 7y1
.....................uucguacguaCcagagcagcu....................................................................... 3 1 7y1
.....................uAcguacguagcagagcagcu....................................................................... 18 1 7y1
.....................uucguacguagcagagUagcu....................................................................... 1 1 7y1
.....................uucguacguagcaUagcagcu....................................................................... 5 1 7y1
.....................uucguaGguagcagagcagcu....................................................................... 1 1 7y1
.....................uucguacguagcaCagcagcu....................................................................... 1 1 7y1
.....................uucguacAuagcagagcagcu....................................................................... 1 1 7y1
.....................uucguaAguagcagagcagcu....................................................................... 22 1 7y1
.....................uucguacguagcagagcCgcu....................................................................... 1 1 7y1
```

**Star** **Mature**

```
gaccugcuucugggucggggguuucguacguagcagagcagcucccucgcugcgaucuauugaaagucagcccucgacacaaggguuuguccgcgcgcgcgcgcgcgugcgugc
.....................uucguacguagGagagcagcu.......................................................................  1      1  7y1
.....................uucguacUuagcagagcagcu.......................................................................  2      1  7y1
.....................Cucguacguagcagagcagcu.......................................................................  2      1  7y1
.....................uucguacguagcagagcagcuU......................................................................  1      1  7y1
.....................uucguaUguagcagagcagcuc......................................................................  3      1  7y1
.....................uucguacguagcagagcagAuc......................................................................  9      1  7y1
.....................uucguacguagcagagcagcAc......................................................................  4      1  7y1
.....................uucguacguagcagaUcagcuc......................................................................  6      1  7y1
.....................uucAuacguagcagagcagcuc......................................................................  6      1  7y1
.....................uucguacguaUcagagcagcuc......................................................................  11     1  7y1
.....................uucguacgCagcagagcagcuc......................................................................  1      1  7y1
.....................uucguaGguagcagagcagcuc......................................................................  1      1  7y1
.....................uucguacguagcagagcUgcuc......................................................................  1      1  7y1
.....................uucguacguagcagagcGgcuc......................................................................  3      1  7y1
.....................uAcguacguagcagagcagcuc......................................................................  27     1  7y1
.....................uucgCacguagcagagcagcuc......................................................................  2      1  7y1
.....................uucguacguagcagagcCgcuc......................................................................  1      1  7y1
.....................uucCuacguagcagagcagcuc......................................................................  2      1  7y1
.....................uucguacguagUagagcagcuc......................................................................  1      1  7y1
.....................uucguacguagcagagcaCcuc......................................................................  5      1  7y1
.....................uucguacguagcagagAagcuc......................................................................  19     1  7y1
.....................uucguacUuagcagagcagcuc......................................................................  5      1  7y1
.....................uucgAacguagcagagcagcuc......................................................................  8      1  7y1
.....................uucguacguagcaUagcagcuc......................................................................  5      1  7y1
.....................uuUguacguagcagagcagcuc......................................................................  2      1  7y1
.....................Aucguacguagcagagcagcuc......................................................................  123    1  7y1
.....................uucguacguagGagagcagcuc......................................................................  3      1  7y1
.....................uucguacguagcagagcagcuc......................................................................  12187  0  7y1
.....................uucguGcguagcagagcagcuc......................................................................  1      1  7y1
.....................uucguacguagAagagcagcuc......................................................................  13     1  7y1
.....................uucguacguagcagagcaUcuc......................................................................  8      1  7y1
.....................uucguacguaCcagagcagcuc......................................................................  9      1  7y1
.....................uucguacguagcaCagcagcuc......................................................................  5      1  7y1
.....................uCcguacguagcagagcagcuc......................................................................  200    1  7y1
.....................uucguacguagcagUgcagcuc......................................................................  2      1  7y1
.....................uucguacguagcagagUagcuc......................................................................  1      1  7y1
.....................uucguacAuagcagagcagcuc......................................................................  3      1  7y1
.....................uucguacguagcGgagcagcuc......................................................................  1      1  7y1
.....................uucguacguagcagagcagcuA......................................................................  8      1  7y1
.....................uucgGacguagcagagcagcuc......................................................................  1      1  7y1
.....................Nucguacguagcagagcagcuc......................................................................  5      1  7y1
.....................uucguacguagcagagcagcGc......................................................................  1      1  7y1
.....................uucguacguagcagagcagcCc......................................................................  1      1  7y1
.....................uucguUcguagcagagcagcuc......................................................................  2      1  7y1
.....................uuAguacguagcagagcagcuc......................................................................  33     1  7y1
.....................uucguacgAagcagagcagcuc......................................................................  15     1  7y1
.....................uucguacguagcagagGagcuc......................................................................  2      1  7y1
.....................uucguacguaAcagagcagcuc......................................................................  2      1  7y1
.....................uucguacguagcagagcagcuG......................................................................  1      1  7y1
.....................Gucguacguagcagagcagcuc......................................................................  29     1  7y1
.....................uucguacCuagcagagcagcuc......................................................................  7      1  7y1
.....................uucguacguGgcagagcagcuc......................................................................  2      1  7y1
.....................uucguacguagcagagcagUuc......................................................................  1      1  7y1
.....................uucguacguagcagagcagGuc......................................................................  1      1  7y1
.....................uucguacguagcagaAcagcuc......................................................................  1      1  7y1
.....................uucguCcguagcagagcagcuc......................................................................  1      1  7y1
.....................uucguacguagcagaCcagcuc......................................................................  8      1  7y1
.....................uucguacguagcagCgcagcuc......................................................................  16     1  7y1
.....................uucguaAguagcagagcagcuc......................................................................  55     1  7y1
.....................uucguacguagcagCgcagcucc.....................................................................  4      1  7y1
.....................uucguacguagcagagcagAucc.....................................................................  5      1  7y1
.....................uucguacguagcaUagcagcucc.....................................................................  4      1  7y1
.....................uucguacAuagcagagcagcucc.....................................................................  1      1  7y1
.....................uucguacguagcagaCcagcucc.....................................................................  2      1  7y1
.....................uucguacguagcagagcagcCcc.....................................................................  1      1  7y1
.....................uucguacguagcGgagcagcucc.....................................................................  5      1  7y1
.....................uucUuacguagcagagcagcucc.....................................................................  3      1  7y1
.....................uucguacguagcagagcagGucc.....................................................................  1      1  7y1
.....................Gucguacguagcagagcagcucc.....................................................................  17     1  7y1
.....................uucAuacguagcagagcagcucc.....................................................................  5      1  7y1
```

# Star                Mature

| | | | |
|---|---|---|---|
| gaccugcuucugggucgggguuucguacguagcagagcagcucccucgcugcgaucuauugaaagucagccccucgacacaaggguuuguccgcgcgcgcgcgcgcgcgugcgugc | | | |
| .....................uucguacguagcagagcagcucA....................................................................... | 13 | 1 | 7y1 |
| .....................uucguacguagcUgagcagcucc....................................................................... | 2 | 1 | 7y1 |
| .....................uucguacgGagcagagcagcucc....................................................................... | 1 | 1 | 7y1 |
| .....................uucguacguagcagagcagcuAc....................................................................... | 9 | 1 | 7y1 |
| .....................uucguacguagcagagcagcGcc....................................................................... | 1 | 1 | 7y1 |
| .....................uucguacguaCcagagcagcucc....................................................................... | 3 | 1 | 7y1 |
| .....................uuUguacguagcagagcagcucc....................................................................... | 1 | 1 | 7y1 |
| .....................uucguacguagcagagcCgcucc....................................................................... | 1 | 1 | 7y1 |
| .....................uucguacguagcagagGagcucc....................................................................... | 2 | 1 | 7y1 |
| .....................uucguacguagcagagUagcucc....................................................................... | 1 | 1 | 7y1 |
| .....................Nucguacguagcagagcagcucc....................................................................... | 4 | 1 | 7y1 |
| .....................uucguacCuagcagagcagcucc....................................................................... | 6 | 1 | 7y1 |
| .....................uucguaGguagcagagcagcucc....................................................................... | 1 | 1 | 7y1 |
| .....................uNcguacguagcagagcagcucc....................................................................... | 1 | 1 | 7y1 |
| .....................uucgAacguagcagagcagcucc....................................................................... | 1 | 1 | 7y1 |
| .....................uAcguacguagcagagcagcucc....................................................................... | 17 | 1 | 7y1 |
| .....................uuAguacguagcagagcagcucc....................................................................... | 16 | 1 | 7y1 |
| .....................uucguUcguagcagagcagcucc....................................................................... | 1 | 1 | 7y1 |
| .....................uucguacguagcagagAagcucc....................................................................... | 8 | 1 | 7y1 |
| .....................uuGguacguagcagagcagcucc....................................................................... | 2 | 1 | 7y1 |
| .....................uucguacgAagcagagcagcucc....................................................................... | 6 | 1 | 7y1 |
| .....................Aucguacguagcagagcagcucc....................................................................... | 71 | 1 | 7y1 |
| .....................uucguacUuagcagagcagcucc....................................................................... | 2 | 1 | 7y1 |
| .....................uucguacguagcagagcagcucc....................................................................... | 7274 | 0 | 7y1 |
| .....................uucguaAguagcagagcagcucc....................................................................... | 26 | 1 | 7y1 |
| .....................uucguacguagAagagcagcucc....................................................................... | 11 | 1 | 7y1 |
| .....................uucguacguaUcagagcagcucc....................................................................... | 8 | 1 | 7y1 |
| .....................uucguacguagcagagcagcAcc....................................................................... | 1 | 1 | 7y1 |
| .....................uucguacguGgcagagcagcucc....................................................................... | 2 | 1 | 7y1 |
| .....................uucguCcguagcagagcagcucc....................................................................... | 1 | 1 | 7y1 |
| .....................uucguacguagcagagcaAcucc....................................................................... | 1 | 1 | 7y1 |
| .....................uucguacguagcagagcagcucG....................................................................... | 1 | 1 | 7y1 |
| .....................uucgCacguagcagagcagcucc....................................................................... | 3 | 1 | 7y1 |
| .....................uucguacguagcagagcaUcucc....................................................................... | 3 | 1 | 7y1 |
| .....................uucguacguagcaCagcagcucc....................................................................... | 1 | 1 | 7y1 |
| .....................uucguacguagGagagcagcucc....................................................................... | 1 | 1 | 7y1 |
| .....................uucguacguagcagaUcagcucc....................................................................... | 5 | 1 | 7y1 |
| .....................uucguacguagUagagcagcucc....................................................................... | 1 | 1 | 7y1 |
| .....................uucguacguagcagagcaCcucc....................................................................... | 1 | 1 | 7y1 |
| .....................uucguacguGgcagagcagcuccc...................................................................... | 1 | 1 | 7y1 |
| .....................uucguacguaCcagagcagcuccc...................................................................... | 2 | 1 | 7y1 |
| .....................uucguacguagcagagcCgcuccc...................................................................... | 2 | 1 | 7y1 |
| .....................uucguacUuagcagagcagcuccc...................................................................... | 1 | 1 | 7y1 |
| .....................uucguacguaUcagagcagcuccc...................................................................... | 1 | 1 | 7y1 |
| .....................uucguacguagcagagcagcuAcc...................................................................... | 7 | 1 | 7y1 |
| .....................uucguacguagcagagcagcuccA...................................................................... | 2 | 1 | 7y1 |
| .....................uAcguacguagcagagcagcuccc...................................................................... | 6 | 1 | 7y1 |
| .....................Nucguacguagcagagcagcuccc...................................................................... | 1 | 1 | 7y1 |
| .....................uucguacguagcagagcagcuccc...................................................................... | 3826 | 0 | 7y1 |
| .....................uucguacCuagcagagcagcuccc...................................................................... | 1 | 1 | 7y1 |
| .....................Aucguacguagcagagcagcuccc...................................................................... | 31 | 1 | 7y1 |
| .....................uucguacguagcagagcagcuUcc...................................................................... | 1 | 1 | 7y1 |
| .....................uucguacguagcagagcNgcuccc...................................................................... | 1 | 1 | 7y1 |
| .....................uucguCcguagcagagcagcuccc...................................................................... | 1 | 1 | 7y1 |
| .....................uucguacguagcagagcagcCccc...................................................................... | 1 | 1 | 7y1 |
| .....................uuAguacguagcagagcagcuccc...................................................................... | 10 | 1 | 7y1 |
| .....................uucguacguagcagagcagcucAc...................................................................... | 16 | 1 | 7y1 |
| .....................uucguacguagcGgagcagcuccc...................................................................... | 1 | 1 | 7y1 |
| .....................uucguacguagcagagcagcuGcc...................................................................... | 1 | 1 | 7y1 |
| .....................uucAuacguagcagagcagcuccc...................................................................... | 1 | 1 | 7y1 |
| .....................uucguacgAagcagagcagcuccc...................................................................... | 2 | 1 | 7y1 |
| .....................uucUuacguagcagagcagcuccc...................................................................... | 1 | 1 | 7y1 |
| .....................uucguacguagcagagAagcuccc...................................................................... | 4 | 1 | 7y1 |
| .....................uucgAacguagcagagcagcuccc...................................................................... | 4 | 1 | 7y1 |
| .....................Gucguacguagcagagcagcuccc...................................................................... | 13 | 1 | 7y1 |
| .....................uucguaAguagcagagcagcuccc...................................................................... | 11 | 1 | 7y1 |
| .....................uucguacguagAagagcagcuccc...................................................................... | 1 | 1 | 7y1 |
| .....................uucguacguagcaCagcagcuccc...................................................................... | 1 | 1 | 7y1 |
| .....................uucguacguagcagaCcagcuccc...................................................................... | 1 | 1 | 7y1 |
| .....................uucguacguagcagagcaCcuccc...................................................................... | 4 | 1 | 7y1 |

**Star** **Mature**

```
gaccugcuucugggucgggguuucguacguagcagagcagcucccucgcugcgaucuauugaaagucagcccucgacacaaggguuuguccgcgcgcgcgcgcgcgugcgugc
.....................uucguacguagcaUagcagcuccc....................................................................  6   1  7y1
.....................uucguacguagcagagUagcuccc....................................................................  1   1  7y1
.....................uucguacguagcagagcaUcuccc....................................................................  5   1  7y1
.....................uucguacguagcagagcaAcuccc....................................................................  1   1  7y1
.....................uucguacguagcagaUcagcuccc....................................................................  3   1  7y1
.....................uucguacguagcagagcagcAccc....................................................................  2   1  7y1
.....................uucguacguagcagagcagcucGc....................................................................  1   1  7y1
.....................uucCuacguagcagagcagcuccc....................................................................  1   1  7y1
.....................uucguacguagcagagcagcuUccu...................................................................  1   1  7y1
.....................uucUuacguagcagagcagcucccu...................................................................  4   1  7y1
.....................uucguacguagcagCgcagcucccu...................................................................  4   1  7y1
.....................uucguacguagcagagcagcGcccu...................................................................  2   1  7y1
.....................uucguacgCagcagagcagcucccu...................................................................  2   1  7y1
.....................uucguacguagcUgagcagcucccu...................................................................  1   1  7y1
.....................uucguacguagcagagcUgcucccu...................................................................  6   1  7y1
.....................uucguacguagcagagcagcucUcu...................................................................  5   1  7y1
.....................Gucguacguagcagagcagcucccu...................................................................  24  1  7y1
.....................uucguacguaAcagagcagcucccu...................................................................  1   1  7y1
.....................uucguacguagcagGgcagcucccu...................................................................  1   1  7y1
.....................Cucguacguagcagagcagcucccu...................................................................  1   1  7y1
.....................uucguacguagcagagcagcAcccu...................................................................  6   1  7y1
.....................uucguacguagcagagcagcuAccu...................................................................  23  1  7y1
.....................uucguacguagcagagAagcucccu...................................................................  11  1  7y1
.....................uucguacguagcagagcagcucAcu...................................................................  24  1  7y1
.....................uucguacguagcGgagcagcucccu...................................................................  2   1  7y1
.....................uucguacguagcagagcagcucccA...................................................................  13  1  7y1
.....................uNcguacguagcagagcagcucccu...................................................................  1   1  7y1
.....................uucguacguagcagagcCgcucccu...................................................................  2   1  7y1
.....................uucguacguagcagaCcagcucccu...................................................................  3   1  7y1
.....................uucguUcguagcagagcagcucccu...................................................................  1   1  7y1
.....................Nucguacguagcagagcagcucccu...................................................................  8   1  7y1
.....................uucguacgGagcagagcagcucccu...................................................................  1   1  7y1
.....................uucguacguagAagagcagcucccu...................................................................  7   1  7y1
.....................uucguacguagcagagcagcuccGu...................................................................  2   1  7y1
.....................uucguacgAagcagagcagcucccu...................................................................  9   1  7y1
.....................uuGguacguagcagagcagcucccu...................................................................  2   1  7y1
.....................uucCuacguagcagagcagcucccu...................................................................  1   1  7y1
.....................uucguaUguagcagagcagcucccu...................................................................  1   1  7y1
.....................uucguacguagcagUgcagcucccu...................................................................  1   1  7y1
.....................uucgCacguagcagagcagcucccu...................................................................  2   1  7y1
.....................uucguacguagcCgagcagcucccu...................................................................  2   1  7y1
.....................uucguacguagcagagcagAucccu...................................................................  5   1  7y1
.....................uucguacguagcagagcaAcucccu...................................................................  1   1  7y1
.....................uucguacguagcaUagcagcucccu...................................................................  5   1  7y1
.....................uucguacguagUagagcagcucccu...................................................................  1   1  7y1
.....................Aucguacguagcagagcagcucccu...................................................................  78  1  7y1
.....................uucguacguagcaCagcagcucccu...................................................................  3   1  7y1
.....................uucguacguagcagagcaCcucccu...................................................................  1   1  7y1
.....................uucguacguagcagagGagcucccu...................................................................  2   1  7y1
.....................uucguacguagcagagUagcucccu...................................................................  1   1  7y1
.....................uucguacguGgcagagcagcucccu...................................................................  1   1  7y1
.....................uucgAacguagcagagcagcucccu...................................................................  5   1  7y1
.....................uucguaGguagcagagcagcucccu...................................................................  1   1  7y1
.....................uucguacguagcagagcaUcucccu...................................................................  5   1  7y1
.....................uucguacguagcagagcagcCcccu...................................................................  2   1  7y1
.....................uucguacguagcagagcagcuGccu...................................................................  1   1  7y1
.....................uucguacguagcagagcagcucccG...................................................................  35  1  7y1
.....................uucguacguaUcagagcagcucccu...................................................................  5   1  7y1
.....................uucguacguaCcagagcagcucccu...................................................................  3   1  7y1
.....................uucguacguagcagagcGgcucccu...................................................................  8   1  7y1
.....................uucguacguagcagagcagcucccC...................................................................  1   1  7y1
.....................uucguacUuagcagagcagcucccu...................................................................  4   1  7y1
.....................uucguacguagcagagcagcuccAu...................................................................  6   1  7y1
.....................uucguacCuagcagagcagcucccu...................................................................  5   1  7y1
.....................uucguacguagGagagcagcucccu...................................................................  1   1  7y1
.....................uucguGcguagcagagcagcucccu...................................................................  1   1  7y1
.....................uucguaAguagcagagcagcucccu...................................................................  40  1  7y1
.....................uucguacAuagcagagcagcucccu...................................................................  3   1  7y1
.....................uuAguacguagcagagcagcucccu...................................................................  23  1  7y1
.....................uAcguacguagcagagcagcucccu...................................................................  21  1  7y1
```

**Star** **Mature**

```
gaccugcuucugggucgggguuucguacguagcagagcagcucccucgcugcgaucuauugaaagucagcccucgacacaaggguuuguccgcgcgcgcgcgcgcgugcgugc
.....................uucguacguagcagaUcagcucccu...................................................................  2     1  7y1
.....................uucguacguagcaAagcagcucccu...................................................................  2     1  7y1
.....................uCcguacguagcagagcagcucccu...................................................................  2     1  7y1
.....................uucguacguagcagagcagcucccu...................................................................  9826  0  7y1
.....................uucAuacguagcagagcagcucccu...................................................................  1     1  7y1
.....................uucguacguagcaUagcagcucccuc..................................................................  4     1  7y1
.....................uucguacguagcagagcagcuAccuc..................................................................  12    1  7y1
.....................uucguacguagcGgagcagcucccuc..................................................................  1     1  7y1
.....................uucguacguagcagagcaUcucccuc..................................................................  1     1  7y1
.....................uucCuacguagcagagcagcucccuc..................................................................  2     1  7y1
.....................uucguacguagcagagcagcuccAuc..................................................................  6     1  7y1
.....................uucguacguaUcagagcagcucccuc..................................................................  3     1  7y1
.....................uucguacguagcagGgcagcucccuc..................................................................  1     1  7y1
.....................uucguacguaCcagagcagcucccuc..................................................................  1     1  7y1
.....................uucguacAuagcagagcagcucccuc..................................................................  1     1  7y1
.....................uucguaAguagcagagcagcucccuc..................................................................  33    1  7y1
.....................uucguacguaAcagagcagcucccuc..................................................................  1     1  7y1
.....................uucguacgCagcagagcagcucccuc..................................................................  1     1  7y1
.....................Nucguacguagcagagcagcucccuc..................................................................  5     1  7y1
.....................uucguacguagcagagcagcGcccuc..................................................................  1     1  7y1
.....................uucguacguagcagagcaCcucccuc..................................................................  1     1  7y1
.....................uucguacguagcagagcagcucccuA..................................................................  1     1  7y1
.....................uucguacguagcagagcagcucccAc..................................................................  1     1  7y1
.....................uucguacguagcagagUagcucccuc..................................................................  1     1  7y1
.....................uucguacguagcagagcagcucccuc..................................................................  7376  0  7y1
.....................uucguacguagcagaUcagcucccuc..................................................................  4     1  7y1
.....................uucguacUuagcagagcagcucccuc..................................................................  2     1  7y1
.....................uucguacgAagcagagcagcucccuc..................................................................  6     1  7y1
.....................Gucguacguagcagagcagcucccuc..................................................................  10    1  7y1
.....................uucguacguagcagagcagcuUccuc..................................................................  1     1  7y1
.....................uucguacguagcagagcagcucccCc..................................................................  2     1  7y1
.....................uucguacguagcagUgcagcucccuc..................................................................  2     1  7y1
.....................uucguacguagcagagcUgcucccuc..................................................................  1     1  7y1
.....................uucguacguagcagagcCgcucccuc..................................................................  3     1  7y1
.....................uucguacguagcagagcagcAcccuc..................................................................  5     1  7y1
.....................uucguacguagcagagcaAcucccuc..................................................................  2     1  7y1
.....................uucguGcguagcagagcagcucccuc..................................................................  1     1  7y1
.....................uucguUcguagcagagcagcucccuc..................................................................  1     1  7y1
.....................uucguacCuagcagagcagcucccuc..................................................................  4     1  7y1
.....................uucguacguagcagagcagcucccuG..................................................................  1     1  7y1
.....................uucgAacguagcagagcagcucccuc..................................................................  4     1  7y1
.....................uucguacguagcUgagcagcucccuc..................................................................  2     1  7y1
.....................Cucguacguagcagagcagcucccuc..................................................................  1     1  7y1
.....................uucguacguagcagCgcagcucccuc..................................................................  8     1  7y1
.....................uucguacguagAagagcagcucccuc..................................................................  5     1  7y1
.....................Aucguacguagcagagcagcucccuc..................................................................  59    1  7y1
.....................uuGguacguagcagagcagcucccuc..................................................................  1     1  7y1
.....................uAcguacguagcagagcagcucccuc..................................................................  11    1  7y1
.....................uucguacguagcagagAagcucccuc..................................................................  11    1  7y1
.....................uuUguacguagcagagcagcucccuc..................................................................  1     1  7y1
.....................uucguacguagcagagcagAucccuc..................................................................  5     1  7y1
.....................uuAguacguagcagagcagcucccuc..................................................................  12    1  7y1
.....................uucUuacguagcagagcagcucccuc..................................................................  2     1  7y1
.....................uCcguacguagcagagcagcucccuc..................................................................  1     1  7y1
.....................uucgCacguagcagagcagcucccuc..................................................................  253   1  7y1
.....................uucguacguagcaAagcagcucccuc..................................................................  1     1  7y1
.....................uucguacguagcagaCcagcucccuc..................................................................  2     1  7y1
.....................uucguacguagcagagcagcucccGc..................................................................  1     1  7y1
.....................uucgGacguagcagagcagcucccuc..................................................................  1     1  7y1
.....................uucguacguagcagagcGgcucccuc..................................................................  2     1  7y1
.....................uucguacguagcagagcagcuGccuc..................................................................  4     1  7y1
.....................uucguacguagcagagcagcucAcuc..................................................................  19    1  7y1
.....................uucCuacguagcagagcagcucccucg.................................................................  1     1  7y1
.....................uucguCcguagcagagcagcucccucg.................................................................  1     1  7y1
.....................uAcguacguagcagagcagcucccucg.................................................................  2     1  7y1
.....................uucguacguagcagagcagcucccucC.................................................................  1     1  7y1
.....................uucguacguagcagGgcagcucccucg.................................................................  2     1  7y1
.....................uucguacguagcagagcagcucccucA.................................................................  1     1  7y1
.....................uucguacguagcagaUcagcucccucg.................................................................  3     1  7y1
.....................Nucguacguagcagagcagcucccucg.................................................................  1     1  7y1
```

**Star** **Mature**

gaccugcuucugggucgggguuucguacguagcagagcagcucccucgcugcgaucuauugaaagucagcccucgacacaaggguuuguccgcgcgcgcgcgcgcgcgugcgugc

| | | | |
|---|---|---|---|
| .....................uucguacguagcagagcagcucAcucg................................................................. | 4 | 1 | 7y1 |
| .....................uucguacguagcagagcaCcucccucg................................................................. | 1 | 1 | 7y1 |
| .....................uucguacCuagcagagcagcucccucg................................................................. | 3 | 1 | 7y1 |
| .....................uucgAacguagcagagcagcucccucg................................................................. | 2 | 1 | 7y1 |
| .....................uucguacguaCcagagcagcucccucg................................................................. | 1 | 1 | 7y1 |
| .....................uucguacguagcagagcagcucccGcg................................................................. | 1 | 1 | 7y1 |
| .....................uuAguacguagcagagcagcucccucg................................................................. | 3 | 1 | 7y1 |
| .....................Gucguacguagcagagcagcucccucg................................................................. | 1 | 1 | 7y1 |
| .....................uucguaAguagcagagcagcucccucg................................................................. | 5 | 1 | 7y1 |
| .....................uucguacguagcagCgcagcucccucg................................................................. | 2 | 1 | 7y1 |
| .....................uucguGcguagcagagcagcucccucg................................................................. | 1 | 1 | 7y1 |
| .....................uucguacgCagcagagcagcucccucg................................................................. | 1 | 1 | 7y1 |
| .....................uucguacguagcagagcagcuAccucg................................................................. | 1 | 1 | 7y1 |
| .....................uucguacguagcagagcagcuccAucg................................................................. | 1 | 1 | 7y1 |
| .....................uucguacguagcagagcagcucccucg................................................................. | 1431 | 0 | 7y1 |
| .....................Aucguacguagcagagcagcucccucg................................................................. | 13 | 1 | 7y1 |
| .....................uucguacguagcagagcagcucccAcg................................................................. | 1 | 1 | 7y1 |
| .....................uucgCacguagcagagcagcucccucg................................................................. | 1 | 1 | 7y1 |
| .....................uucguacguagcagagAagcucccucg................................................................. | 1 | 1 | 7y1 |
| .....................uucguacguagcagagUagcucccucg................................................................. | 1 | 1 | 7y1 |
| .....................uucguacguagcagagcagAucccucgc................................................................ | 1 | 1 | 7y1 |
| .....................uuAguacguagcagagcagcucccucgc................................................................ | 2 | 1 | 7y1 |
| .....................uucguacguagcagagcaAcucccucgc................................................................ | 1 | 1 | 7y1 |
| .....................uucguacguagcagagcaCcucccucgc................................................................ | 1 | 1 | 7y1 |
| .....................uucguaAguagcagagcagcucccucgc................................................................ | 3 | 1 | 7y1 |
| .....................uucguacguagcagagcagcucccucgA................................................................ | 1 | 1 | 7y1 |
| .....................Cucguacguagcagagcagcucccucgc................................................................ | 1 | 1 | 7y1 |
| .....................uucguacguagcagagcagcucccucgc................................................................ | 923 | 0 | 7y1 |
| .....................uucgCacguagcagagcagcucccucgc................................................................ | 1 | 1 | 7y1 |
| .....................uucguacguagcagagcagGucccucgc................................................................ | 1 | 1 | 7y1 |
| .....................uAcguacguagcagagcagcucccucgc................................................................ | 1 | 1 | 7y1 |
| .....................uucguacguagcagagcagcuGccucgc................................................................ | 1 | 1 | 7y1 |
| .....................uucguacguaCcagagcagcucccucgc................................................................ | 1 | 1 | 7y1 |
| .....................uucguacguagcagagcagcuAccucgc................................................................ | 2 | 1 | 7y1 |
| .....................Aucguacguagcagagcagcucccucgc................................................................ | 15 | 1 | 7y1 |
| .....................uucguacgAagcagagcagcucccucgc................................................................ | 1 | 1 | 7y1 |
| .....................Gucguacguagcagagcagcucccucgc................................................................ | 1 | 1 | 7y1 |
| .....................uucguacguagcagagcGgcucccucgc................................................................ | 1 | 1 | 7y1 |
| .....................uucguacUuagcagagcagcucccucgc................................................................ | 1 | 1 | 7y1 |
| .....................uucguacguagcagagcagcucAcucgc................................................................ | 1 | 1 | 7y1 |
| .....................Nucguacguagcagagcagcucccucgc................................................................ | 1 | 1 | 7y1 |
| .....................uucguacguagcagagcagcucccuAgc................................................................ | 1 | 1 | 7y1 |
| .....................uucguacguagcagagcagcucccucgcA............................................................... | 2 | 1 | 7y1 |
| .....................uucguacUuagcagagcagcucccucgcu............................................................... | 3 | 1 | 7y1 |
| .....................uucguacguagcagagcagcCcccucgcu............................................................... | 1 | 1 | 7y1 |
| .....................uCcguacguagcagagcagcucccucgcu............................................................... | 2 | 1 | 7y1 |
| .....................uucguacguagcagagAagcucccucgcu............................................................... | 3 | 1 | 7y1 |
| .....................uucguacguagcagagcUgcucccucgcu............................................................... | 2 | 1 | 7y1 |
| .....................uucguacguagcagagcagcucAcucgcu............................................................... | 9 | 1 | 7y1 |
| .....................uucguacguagcagagcaUcucccucgcu............................................................... | 2 | 1 | 7y1 |
| .....................uucguaAguagcagagcagcucccucgcu............................................................... | 13 | 1 | 7y1 |
| .....................uucguacguagcagagcGgcucccucgcu............................................................... | 2 | 1 | 7y1 |
| .....................uucguacguagcagagcagcucGcucgcu............................................................... | 1 | 1 | 7y1 |
| .....................uucguacguagAagagcagcucccucgcu............................................................... | 6 | 1 | 7y1 |
| .....................uucguacCuagcagagcagcucccucgcu............................................................... | 1 | 1 | 7y1 |
| .....................uucguacguagcagagcagcucccucgGu............................................................... | 1 | 1 | 7y1 |
| .....................uucAuacguagcagagcagcucccucgcu............................................................... | 1 | 1 | 7y1 |
| .....................uucguGcguagcagagcagcucccucgcu............................................................... | 1 | 1 | 7y1 |
| .....................uucCuacguagcagagcagcucccucgcu............................................................... | 4 | 1 | 7y1 |
| .....................uucguacguagcagagcagcucccucgcu............................................................... | 4044 | 0 | 7y1 |
| .....................Gucguacguagcagagcagcucccucgcu............................................................... | 6 | 1 | 7y1 |
| .....................uucguacguagcagagcagcuUccucgcu............................................................... | 1 | 1 | 7y1 |
| .....................uucguacguagcagagcagAucccucgcu............................................................... | 1 | 1 | 7y1 |
| .....................uucguacguagcagagcagcucccAcgcu............................................................... | 2 | 1 | 7y1 |
| .....................uGcguacguagcagagcagcucccucgcu............................................................... | 1 | 1 | 7y1 |
| .....................uucguacguagcGgagcagcucccucgcu............................................................... | 2 | 1 | 7y1 |
| .....................uucguacguagcagagcagcucccucUcu............................................................... | 1 | 1 | 7y1 |
| .....................uucguacguagcagagcagcucccucgAu............................................................... | 3 | 1 | 7y1 |
| .....................uucguacguagcaUagcagcucccucgcu............................................................... | 4 | 1 | 7y1 |
| .....................uucguacAuagcagagcagcucccucgcu............................................................... | 2 | 1 | 7y1 |

**Star** **Mature**

gaccugcuucugggucggggguucguacguagcagagcagcucccucgcugcgaucuauugaaagucagcccucgacacaaggguuuguccgcgcgcgcgcgcgcgugcgugc

| | | | |
|---|---|---|---|
| .....................uucguacgAagcagagcagcucccucgcu............................................................... | 4 | 1 | 7y1 |
| .....................uucguacguagcagagcagcuAccucgcu............................................................... | 5 | 1 | 7y1 |
| .....................uAcguacguagcagagcagcucccucgcu............................................................... | 6 | 1 | 7y1 |
| .....................uuGguacguagcagagcagcucccucgcu............................................................... | 2 | 1 | 7y1 |
| .....................uucguacguagcUgagcagcucccucgcu............................................................... | 1 | 1 | 7y1 |
| .....................uucguacguaCcagagcagcucccucgcu............................................................... | 1 | 1 | 7y1 |
| .....................uucguacguagcagagcagcucccucCcu............................................................... | 4 | 1 | 7y1 |
| .....................uucguacguagcagagcCgcucccucgcu............................................................... | 1 | 1 | 7y1 |
| .....................Aucguacguagcagagcagcucccucgcu............................................................... | 36 | 1 | 7y1 |
| .....................uNcguacguagcagagcagcucccucgcu............................................................... | 1 | 1 | 7y1 |
| .....................uuAguacguagcagagcagcucccucgcu............................................................... | 8 | 1 | 7y1 |
| .....................uucguacguagcagagcagcucccucgcG............................................................... | 23 | 1 | 7y1 |
| .....................uucguaUguagcagagcagcucccucgcu............................................................... | 1 | 1 | 7y1 |
| .....................uucguacguagcagagcagcuccAucgcu............................................................... | 2 | 1 | 7y1 |
| .....................uucguacguagcagagcagcucccuAgcu............................................................... | 1 | 1 | 7y1 |
| .....................uucguacguagcagagcagcAcccucgcu............................................................... | 1 | 1 | 7y1 |
| .....................uucgAacguagcagagcagcucccucgcu............................................................... | 1 | 1 | 7y1 |
| .....................uucgGacguagcagagcagcucccucgcu............................................................... | 1 | 1 | 7y1 |
| .....................Nucguacguagcagagcagcucccucgcu............................................................... | 2 | 1 | 7y1 |
| .....................uucCuacguagcagagcagcucccucgcug.............................................................. | 1 | 1 | 7y1 |
| .....................uucguacguagcagagcagcucccuAgcug.............................................................. | 2 | 1 | 7y1 |
| .....................uucguacgAagcagagcagcucccucgcug.............................................................. | 1 | 1 | 7y1 |
| .....................uucguaAguagcagagcagcucccucgcug.............................................................. | 4 | 1 | 7y1 |
| .....................uucguacUuagcagagcagcucccucgcug.............................................................. | 1 | 1 | 7y1 |
| .....................Nucguacguagcagagcagcucccucgcug.............................................................. | 1 | 1 | 7y1 |
| .....................uucguacguagcagaCcagcucccucgcug.............................................................. | 1 | 1 | 7y1 |
| .....................Aucguacguagcagagcagcucccucgcug.............................................................. | 5 | 1 | 7y1 |
| .....................uucguacguagcagagcagcucccucgcGg.............................................................. | 1 | 1 | 7y1 |
| .....................uucguacguagcagagcagAucccucgcug.............................................................. | 1 | 1 | 7y1 |
| .....................uucguacguagcagagcagcucccucgcug.............................................................. | 459 | 0 | 7y1 |
| .....................uAcguacguagcagagcagcucccucgcug.............................................................. | 2 | 1 | 7y1 |
| .....................uucguacCuagcagagcagcucccucgcug.............................................................. | 2 | 1 | 7y1 |
| .....................Gucguacguagcagagcagcucccucgcugc............................................................. | 1 | 1 | 7y1 |
| .....................uucguGcguagcagagcagcucccucgcugc............................................................. | 1 | 1 | 7y1 |
| .....................uucguacguagcagagcagcucccucgcuCc............................................................. | 1 | 1 | 7y1 |
| .....................uucguacguagcaUagcagcucccucgcugc............................................................. | 1 | 1 | 7y1 |
| .....................uucguacUuagcagagcagcucccucgcugc............................................................. | 1 | 1 | 7y1 |
| .....................Aucguacguagcagagcagcucccucgcugc............................................................. | 9 | 1 | 7y1 |
| .....................uucguacguaCcagagcagcucccucgcugc............................................................. | 1 | 1 | 7y1 |
| .....................uucguacguagcagagcagcucAcucgcugc............................................................. | 2 | 1 | 7y1 |
| .....................uucguaAguagcagagcagcucccucgcugc............................................................. | 1 | 1 | 7y1 |
| .....................uucguacguagcagagcCgcucccucgcugc............................................................. | 1 | 1 | 7y1 |
| .....................uucguacguagcagagcagcuccAucgcugc............................................................. | 1 | 1 | 7y1 |
| .....................uucguacguagAagagcagcucccucgcugc............................................................. | 2 | 1 | 7y1 |
| .....................uuAguacguagcagagcagcucccucgcugc............................................................. | 1 | 1 | 7y1 |
| .....................uucguacguagcagaCcagcucccucgcugc............................................................. | 1 | 1 | 7y1 |
| .....................uucguacguagcagagcagcucccucgcugA............................................................. | 1 | 1 | 7y1 |
| .....................uAcguacguagcagagcagcucccucgcugc............................................................. | 3 | 1 | 7y1 |
| .....................uucguacguagcagGgcagcucccucgcugc............................................................. | 1 | 1 | 7y1 |
| .....................uucguacguagcagagcagcucccucgcugc............................................................. | 924 | 0 | 7y1 |
| ......................ucguacCuagcagagcagc........................................................................ | 1 | 1 | 7y1 |
| ......................ucguacguagcagagcagc........................................................................ | 1251 | 0 | 7y1 |
| ......................ucguacguagAagagcagc........................................................................ | 3 | 1 | 7y1 |
| ......................ucguacguagcagagcagA........................................................................ | 1 | 1 | 7y1 |
| ......................ucguacgAagcagagcagc........................................................................ | 3 | 1 | 7y1 |
| ......................Acguacguagcagagcagc........................................................................ | 13 | 1 | 7y1 |
| ......................uAguacguagcagagcagc........................................................................ | 2 | 1 | 7y1 |
| ......................ucguaAguagcagagcagc........................................................................ | 2 | 1 | 7y1 |
| ......................ucguacguagcagCgcagc........................................................................ | 4 | 1 | 7y1 |
| ......................Gcguacguagcagagcagc........................................................................ | 1 | 1 | 7y1 |
| ......................ucguacUuagcagagcagc........................................................................ | 1 | 1 | 7y1 |
| ......................ucguacguagcagagcagG........................................................................ | 1 | 1 | 7y1 |
| ......................ucguacguagcagagcaUc........................................................................ | 2 | 1 | 7y1 |
| ......................ucguacguagcaCagcagc........................................................................ | 1 | 1 | 7y1 |
| ......................ucguacguagcGgagcagc........................................................................ | 1 | 1 | 7y1 |
| ......................ucguacguagcaCagcagcu....................................................................... | 2 | 1 | 7y1 |
| ......................uNguacguagcagagcagcu....................................................................... | 1 | 1 | 7y1 |
| ......................uAguacguagcagagcagcu....................................................................... | 6 | 1 | 7y1 |
| ......................ucUuacguagcagagcagcu....................................................................... | 2 | 1 | 7y1 |
| ......................ucguacguagcagagcagcG....................................................................... | 20 | 1 | 7y1 |

# Star                Mature

```
gaccugcuucugggucggggguuucguacguagcagagcagcucccucgcugcgaucuauugaaagucagcccucgacacaaggguuuguccgcgcgcgcgcgcgcgugcgugc
......................ucguacguagcagagAagcu.......................................................................   6      1   7y1
......................ucguacguagAagagcagcu.......................................................................   5      1   7y1
......................ucguacguagcagagcaUcu.......................................................................   4      1   7y1
......................ucguacguagcagCgcagcu.......................................................................   4      1   7y1
......................Gcguacguagcagagcagcu.......................................................................   6      1   7y1
......................ucguacguagcagagcagcu.......................................................................   2186   0   7y1
......................ucguacguagcagagcagcA.......................................................................   1      1   7y1
......................uUguacguagcagagcagcu.......................................................................   1      1   7y1
......................ucguacguagcagaUcagcu.......................................................................   2      1   7y1
......................ucguacguaCcagagcagcu.......................................................................   1      1   7y1
......................ucguaAguagcagagcagcu.......................................................................   6      1   7y1
......................ucguacguagcagagcGgcu.......................................................................   1      1   7y1
......................ucguacguagcagUgcagcu.......................................................................   1      1   7y1
......................Acguacguagcagagcagcu.......................................................................   36     1   7y1
......................ucguacguaUcagagcagcu.......................................................................   2      1   7y1
......................ucguGcguagcagagcagcu.......................................................................   1      1   7y1
......................ucguacguagcagagcUgcu.......................................................................   1      1   7y1
......................ucguacgAagcagagcagcu.......................................................................   2      1   7y1
......................uAguacguagcagagcagcuc......................................................................   19     1   7y1
......................ucguacguagcagaUcagcuc......................................................................   6      1   7y1
......................ucguacguagcagagcaCcuc......................................................................   6      1   7y1
......................ucgAacguagcagagcagcuc......................................................................   1      1   7y1
......................ucguacguaCcagagcagcuc......................................................................   6      1   7y1
......................ucguacCuagcagagcagcuc......................................................................   4      1   7y1
......................ucUuacguagcagagcagcuc......................................................................   5      1   7y1
......................ucguacguagcagagcagcuc......................................................................   6627   0   7y1
......................Acguacguagcagagcagcuc......................................................................   79     1   7y1
......................ucguacguagcagagGagcuc......................................................................   1      1   7y1
......................ucguacguagAagagcagcuc......................................................................   9      1   7y1
......................ucguacguagcagGgcagcuc......................................................................   2      1   7y1
......................ucguacguagcagagcagcuA......................................................................   7      1   7y1
......................ucguacguagcagagUagcuc......................................................................   1      1   7y1
......................ucguacguagcaCagcagcuc......................................................................   238    1   7y1
......................ucguacguagcagUgcagcuc......................................................................   1      1   7y1
......................ucguacguagUagagcagcuc......................................................................   1      1   7y1
......................ucguacgAagcagagcagcuc......................................................................   9      1   7y1
......................ucguacUuagcagagcagcuc......................................................................   6      1   7y1
......................Ncguacguagcagagcagcuc......................................................................   4      1   7y1
......................ucguacgCagcagagcagcuc......................................................................   2      1   7y1
......................ucguacguagcagagcGgcuc......................................................................   1      1   7y1
......................uGguacguagcagagcagcuc......................................................................   1      1   7y1
......................uUguacguagcagagcagcuc......................................................................   1      1   7y1
......................ucguacguagcagagcagGuc......................................................................   1      1   7y1
......................ucguacguagcagCgcagcuc......................................................................   5      1   7y1
......................ucguacguaAcagagcagcuc......................................................................   1      1   7y1
......................ucguacguagcagaCcagcuc......................................................................   1      1   7y1
......................ucguacguagcagagcagcGc......................................................................   3      1   7y1
......................ucguacAuagcagagcagcuc......................................................................   1      1   7y1
......................ucguacguagcagagcagcAc......................................................................   4      1   7y1
......................ucCuacguagcagagcagcuc......................................................................   5      1   7y1
......................ucguacguagcagagcCgcuc......................................................................   1      1   7y1
......................ucguacguaUcagagcagcuc......................................................................   3      1   7y1
......................ucguacguagcagagcUgcuc......................................................................   2      1   7y1
......................ucguaAguagcagagcagcuc......................................................................   15     1   7y1
......................Gcguacguagcagagcagcuc......................................................................   7      1   7y1
......................ucguacguagcagagcaUcuc......................................................................   3      1   7y1
......................ucguUcguagcagagcagcuc......................................................................   1      1   7y1
......................ucguacguagcGgagcagcuc......................................................................   2      1   7y1
......................ucguacguagcagagAagcuc......................................................................   15     1   7y1
......................ucguaUguagcagagcagcuc......................................................................   2      1   7y1
......................ucguaGguagcagagcagcuc......................................................................   1      1   7y1
......................ucguacguagcagagcagcuG......................................................................   1      1   7y1
......................ucAuacguagcagagcagcuc......................................................................   1      1   7y1
......................ucguacguagcagagcagAuc......................................................................   3      1   7y1
......................ucguacguGgcagagcagcucc.....................................................................   1      1   7y1
......................ucguacgAagcagagcagcucc.....................................................................   1      1   7y1
......................ucguacguagAagagcagcucc.....................................................................   1      1   7y1
......................ucguacguagcGgagcagcucc.....................................................................   1      1   7y1
......................uGguacguagcagagcagcucc.....................................................................   1      1   7y1
......................ucguacguagcagagUagcucc.....................................................................   1      1   7y1
```

**Star** **Mature**

```
gaccugcuucugggucggggguuucguacguagcagagcagcucccucgcugcgaucuauugaaagucagcccucgacacaaggguuuguccgcgcgcgcgcgcgcgugcgugc
......................Ncguacguagcagagcagcucc.....................................................................  1     1  7y1
......................ucguacguagcagaUcagcucc.....................................................................  1     1  7y1
......................ucguacguagcagagcagcucA.....................................................................  6     1  7y1
......................ucguacguagcagagcagcucU.....................................................................  1     1  7y1
......................ucUuacguagcagagcagcucc.....................................................................  2     1  7y1
......................ucguacguagcagagAagcucc.....................................................................  2     1  7y1
......................ucguacguagcagagcagAucc.....................................................................  1     1  7y1
......................Acguacguagcagagcagcucc.....................................................................  15    1  7y1
......................Gcguacguagcagagcagcucc.....................................................................  5     1  7y1
......................ucguacguagcagaCcagcucc.....................................................................  1     1  7y1
......................ucguacguagcagagcagcucc.....................................................................  1758  0  7y1
......................ucguacguagcagagcagcCcc.....................................................................  1     1  7y1
......................ucguaAguagcagagcagcucc.....................................................................  4     1  7y1
......................ucguacguaUcagagcagcucc.....................................................................  2     1  7y1
......................ucguaUguagcagagcagcucc.....................................................................  1     1  7y1
......................ucguacguagcagagcagcuAc.....................................................................  4     1  7y1
......................ucguacguagcagagcagcuUc.....................................................................  1     1  7y1
......................uAguacguagcagagcagcucc.....................................................................  1     1  7y1
......................ucguacguagcagGgcagcucc.....................................................................  1     1  7y1
......................ucguGcguagcagagcagcucc.....................................................................  1     1  7y1
......................ucguacguaCcagagcagcucc.....................................................................  4     1  7y1
......................ucguacguagcagaAcagcucc.....................................................................  1     1  7y1
......................ucCuacguagcagagcagcucc.....................................................................  2     1  7y1
......................ucguacguagcagUgcagcuccc....................................................................  1     1  7y1
......................Gcguacguagcagagcagcuccc....................................................................  4     1  7y1
......................ucguacguagcagagcagcuAcc....................................................................  3     1  7y1
......................ucguacguagcagagcagcuccc....................................................................  2876  0  7y1
......................ucguacguagcagagcagcAccc....................................................................  1     1  7y1
......................ucguacguagcagagcagcucAc....................................................................  5     1  7y1
......................ucguacguagAagagcagcuccc....................................................................  3     1  7y1
......................ucguaAguagcagagcagcuccc....................................................................  8     1  7y1
......................uAguacguagcagagcagcuccc....................................................................  2     1  7y1
......................ucguacguagcagagcaUcuccc....................................................................  1     1  7y1
......................ucguacguaUcagagcagcuccc....................................................................  1     1  7y1
......................uUguacguagcagagcagcuccc....................................................................  2     1  7y1
......................ucguacguagcagagcaCcuccc....................................................................  2     1  7y1
......................ucguacguagcagagAagcuccc....................................................................  4     1  7y1
......................ucguacguaCcagagcagcuccc....................................................................  2     1  7y1
......................ucguacguagcagCgcagcuccc....................................................................  7     1  7y1
......................ucguacguagcagagcagcuccA....................................................................  2     1  7y1
......................ucAuacguagcagagcagcuccc....................................................................  2     1  7y1
......................ucUuacguagcagagcagcuccc....................................................................  1     1  7y1
......................ucgAacguagcagagcagcuccc....................................................................  1     1  7y1
......................ucguacguagcagagcagcuGcc....................................................................  1     1  7y1
......................ucguUcguagcagagcagcuccc....................................................................  1     1  7y1
......................ucguacguagcagagcagAuccc....................................................................  4     1  7y1
......................Acguacguagcagagcagcuccc....................................................................  26    1  7y1
......................uGguacguagcagagcagcuccc....................................................................  1     1  7y1
......................ucguacCuagcagagcagcuccc....................................................................  1     1  7y1
......................ucguacgAagcagagcagcuccc....................................................................  1     1  7y1
......................ucguacguagcagagGagcuccc....................................................................  1     1  7y1
......................ucguacguagcagaAcagcuccc....................................................................  1     1  7y1
......................ucguacguagcaCagcagcuccc....................................................................  2     1  7y1
......................ucguacgAagcagagcagcucccu...................................................................  3     1  7y1
......................Ccguacguagcagagcagcucccu...................................................................  1     1  7y1
......................ucguacguagcagCgcagcucccu...................................................................  6     1  7y1
......................uUguacguagcagagcagcucccu...................................................................  1     1  7y1
......................ucguacguagcagagcagcucGcu...................................................................  1     1  7y1
......................ucguacguagcUgagcagcucccu...................................................................  1     1  7y1
......................ucguacguagcagagcagcuGccu...................................................................  1     1  7y1
......................ucguacguagcagagcagUucccu...................................................................  1     1  7y1
......................ucguacguaUcagagcagcucccu...................................................................  1     1  7y1
......................ucguacguagcagagcagcuAccu...................................................................  2     1  7y1
......................Gcguacguagcagagcagcucccu...................................................................  5     1  7y1
......................ucguacguagcagaCcagcucccu...................................................................  2     1  7y1
......................ucguacguagcaUagcagcucccu...................................................................  2     1  7y1
......................uAguacguagcagagcagcucccu...................................................................  6     1  7y1
......................ucguacguagcagagAagcucccu...................................................................  1     1  7y1
......................ucguacguagcagagcagcCcccu...................................................................  1     1  7y1
......................ucguacguagcagagcCgcucccu...................................................................  2     1  7y1
```

**Star** **Mature**

| | | | |
|---|---|---|---|
| gaccugcuucugggucgggguuucguacguagcagagcagcucccucgcugcgaucuauugaaagucagcccucgacacaaggguuuguccgcgcgcgcgcgcgcgugcgugc | | | |
| ......................ucguacguGgcagagcagcucccu................................................................... | 2 | 1 | 7y1 |
| ......................ucguacguagcagagcagAucccu................................................................... | 1 | 1 | 7y1 |
| ......................ucguGcguagcagagcagcucccu................................................................... | 1 | 1 | 7y1 |
| ......................ucguacguaCcagagcagcucccu................................................................... | 1 | 1 | 7y1 |
| ......................ucguaAguagcagagcagcucccu................................................................... | 3 | 1 | 7y1 |
| ......................ucguacguagcagagcagcucccG................................................................... | 9 | 1 | 7y1 |
| ......................ucguacguagcGgagcagcucccu................................................................... | 2 | 1 | 7y1 |
| ......................ucgAacguagcagagcagcucccu................................................................... | 1 | 1 | 7y1 |
| ......................ucguacguagcagagcagcucccu................................................................... | 2486 | 0 | 7y1 |
| ......................ucguacguagcagagcagcucAcu................................................................... | 4 | 1 | 7y1 |
| ......................ucCuacguagcagagcagcucccu................................................................... | 2 | 1 | 7y1 |
| ......................ucguacguagcagagUagcucccu................................................................... | 1 | 1 | 7y1 |
| ......................ucguacguagcagagcagcuUccu................................................................... | 1 | 1 | 7y1 |
| ......................ucguacguagcagagcagcucccA................................................................... | 5 | 1 | 7y1 |
| ......................ucguacguagcagagcagGucccu................................................................... | 1 | 1 | 7y1 |
| ......................ucguacguagAagagcagcucccu................................................................... | 1 | 1 | 7y1 |
| ......................Acguacguagcagagcagcucccu................................................................... | 21 | 1 | 7y1 |
| ......................ucguacguagcagagcagcuccAu................................................................... | 6 | 1 | 7y1 |
| ......................ucguacUuagcagagcagcucccu................................................................... | 3 | 1 | 7y1 |
| ......................ucguacCuagcagagcagcucccu................................................................... | 1 | 1 | 7y1 |
| ......................uUguacguagcagagcagcucccuc.................................................................. | 1 | 1 | 7y1 |
| ......................ucguacCuagcagagcagcucccuc.................................................................. | 1 | 1 | 7y1 |
| ......................ucgAacguagcagagcagcucccuc.................................................................. | 1 | 1 | 7y1 |
| ......................ucguacguagcagagcagcuAccuc.................................................................. | 1 | 1 | 7y1 |
| ......................ucguacguagcagagcagcuUccuc.................................................................. | 1 | 1 | 7y1 |
| ......................ucguacguagAagagcagcucccuc.................................................................. | 1 | 1 | 7y1 |
| ......................ucCuacguagcagagcagcucccuc.................................................................. | 1 | 1 | 7y1 |
| ......................ucguacguagcagagcagcucccuG.................................................................. | 1 | 1 | 7y1 |
| ......................ucguacguagcagagcagcucccuc.................................................................. | 555 | 0 | 7y1 |
| ......................Acguacguagcagagcagcucccuc.................................................................. | 7 | 1 | 7y1 |
| ......................ucguacguaCcagagcagcucccuc.................................................................. | 1 | 1 | 7y1 |
| ......................ucguaAguagcagagcagcucccuc.................................................................. | 1 | 1 | 7y1 |
| ......................Ncguacguagcagagcagcucccuc.................................................................. | 1 | 1 | 7y1 |
| ......................ucguacguagcagagcagcAcccuc.................................................................. | 1 | 1 | 7y1 |
| ......................ucguacguagcagagcGgcucccuc.................................................................. | 1 | 1 | 7y1 |
| ......................ucguacguagcagagcagcucAcuc.................................................................. | 1 | 1 | 7y1 |
| ......................ucguacguagcagCgcagcucccucg................................................................. | 1 | 1 | 7y1 |
| ......................Ncguacguagcagagcagcucccucg................................................................. | 1 | 1 | 7y1 |
| ......................ucguacguagcagagcagcuccAucg................................................................. | 1 | 1 | 7y1 |
| ......................ucguacguaCcagagcagcucccucg................................................................. | 1 | 1 | 7y1 |
| ......................ucguacguagcagaUcagcucccucg................................................................. | 1 | 1 | 7y1 |
| ......................ucguacgAagcagagcagcucccucg................................................................. | 1 | 1 | 7y1 |
| ......................ucCuacguagcagagcagcucccucg................................................................. | 1 | 1 | 7y1 |
| ......................ucguacguagcagagcaCcucccucg................................................................. | 1 | 1 | 7y1 |
| ......................ucguacguagcCgagcagcucccucg................................................................. | 1 | 1 | 7y1 |
| ......................ucguacguagcagagcagcucccucg................................................................. | 219 | 0 | 7y1 |
| ......................ucguacAuagcagagcagcucccucg................................................................. | 1 | 1 | 7y1 |
| ......................uAguacguagcagagcagcucccucg................................................................. | 1 | 1 | 7y1 |
| ......................ucguaAguagcagagcagcucccucgc................................................................ | 2 | 1 | 7y1 |
| ......................ucguacguagcagagcagcucccuAgc................................................................ | 1 | 1 | 7y1 |
| ......................ucguacguagcagagcagcuAccucgc................................................................ | 1 | 1 | 7y1 |
| ......................ucguacguagcagagcagcucccucgc................................................................ | 270 | 0 | 7y1 |
| ......................Acguacguagcagagcagcucccucgc................................................................ | 4 | 1 | 7y1 |
| ......................uAguacguagcagagcagcucccucgc................................................................ | 2 | 1 | 7y1 |
| ......................ucguacguagcagagAagcucccucgc................................................................ | 1 | 1 | 7y1 |
| ......................ucguacguagcagagcagcucccucAcu............................................................... | 1 | 1 | 7y1 |
| ......................ucguacguagcagaAcagcucccucgcu............................................................... | 1 | 1 | 7y1 |
| ......................ucguacguaAcagagcagcucccucgcu............................................................... | 1 | 1 | 7y1 |
| ......................ucguacguagcagagcagcCcccucgcu............................................................... | 1 | 1 | 7y1 |
| ......................ucguacguaCcagagcagcucccucgcu............................................................... | 1 | 1 | 7y1 |
| ......................ucguacguagcagagcagcucccucgcA............................................................... | 1 | 1 | 7y1 |
| ......................ucguacguagcagagcagcuAccucgcu............................................................... | 1 | 1 | 7y1 |
| ......................ucguacUuagcagagcagcucccucgcu............................................................... | 1 | 1 | 7y1 |
| ......................ucguacguagcagUgcagcucccucgcu............................................................... | 1 | 1 | 7y1 |
| ......................ucguacguagcagagcagcucccAcgcu............................................................... | 1 | 1 | 7y1 |
| ......................ucguacguagAagagcagcucccucgcu............................................................... | 1 | 1 | 7y1 |
| ......................ucguacguagcagagcagcucccucgcu............................................................... | 1342 | 0 | 7y1 |
| ......................uUguacguagcagagcagcucccucgcu............................................................... | 1 | 1 | 7y1 |
| ......................ucguacguagcagagAagcucccucgcu............................................................... | 3 | 1 | 7y1 |
| ......................ucguacAuagcagagcagcucccucgcu............................................................... | 2 | 1 | 7y1 |

**Star** **Mature**

```
gaccugcuucugggucgggguuucguacguagcagagcagcucccucgcugcgaucuauugaaagucagcccucgacacaaggguuuguccgcgcgcgcgcgcgcgugcgugc
......................ucguacgAagcagagcagcucccucgcu...............................................................   4     1   7y1
......................ucguacguagcagagcagcucccucUcu...............................................................   1     1   7y1
......................ucguacguagcagagcagcuUccucgcu...............................................................   1     1   7y1
......................ucguaUguagcagagcagcucccucgcu...............................................................   1     1   7y1
......................ucguacguagcagagcagcucccucgcG...............................................................   16    1   7y1
......................ucguacguaUcagagcagcucccucgcu...............................................................   1     1   7y1
......................ucguacguagcagagcagcucGcucgcu...............................................................   1     1   7y1
......................ucguacCuagcagagcagcucccucgcu...............................................................   1     1   7y1
......................ucguacguagcagagcagcucAcucgcu...............................................................   1     1   7y1
......................ucguacguagcagagcagAucccucgcu...............................................................   2     1   7y1
......................ucguacguagcagagcagcucccuAgcu...............................................................   3     1   7y1
......................ucguacguagcagagcagcucccucCcu...............................................................   2     1   7y1
......................ucguaAguagcagagcagcucccucgcu...............................................................   1     1   7y1
......................ucguacguagcagagcagcuccAucgcu...............................................................   1     1   7y1
......................Acguacguagcagagcagcucccucgcu...............................................................   11    1   7y1
......................Ncguacguagcagagcagcucccucgcu...............................................................   3     1   7y1
......................uAguacguagcagagcagcucccucgcu...............................................................   2     1   7y1
......................ucguacguagcGgagcagcucccucgcu...............................................................   1     1   7y1
......................ucguacguagcagagcagcuAccucgcug..............................................................   3     1   7y1
......................ucguacguagcagagcagUucccucgcug..............................................................   1     1   7y1
......................uAguacguagcagagcagcucccucgcug..............................................................   3     1   7y1
......................Acguacguagcagagcagcucccucgcug..............................................................   17    1   7y1
......................ucguacguagcagagcagcuccAucgcug..............................................................   4     1   7y1
......................ucguacguagcagagcagcucccucUcug..............................................................   1     1   7y1
......................ucguacguagGagagcagcucccucgcug..............................................................   1     1   7y1
......................ucguacguaUcagagcagcucccucgcug..............................................................   1     1   7y1
......................ucguacguagcagagcagcucccuUgcug..............................................................   1     1   7y1
......................ucguacguagAagagcagcucccucgcug..............................................................   3     1   7y1
......................ucgAacguagcagagcagcucccucgcug..............................................................   1     1   7y1
......................ucguacguagcagagcagcucccucgcuC..............................................................   1     1   7y1
......................ucguGcguagcagagcagcucccucgcug..............................................................   1     1   7y1
......................ucguacguGgcagagcagcucccucgcug..............................................................   1     1   7y1
......................Gcguacguagcagagcagcucccucgcug..............................................................   3     1   7y1
......................ucguacCuagcagagcagcucccucgcug..............................................................   3     1   7y1
......................uGguacguagcagagcagcucccucgcug..............................................................   2     1   7y1
......................ucguacguaCcagagcagcucccucgcug..............................................................   1     1   7y1
......................ucguacguagcagagcagcuUccucgcug..............................................................   1     1   7y1
......................ucUuacguagcagagcagcucccucgcug..............................................................   1     1   7y1
......................ucguacguagcagagcagcucccAcgcug..............................................................   4     1   7y1
......................ucguaAguagcagagcagcucccucgcug..............................................................   2     1   7y1
......................ucguacguagcagagcagcucAcucgcug..............................................................   3     1   7y1
......................ucguacguagcaCagcagcucccucgcug..............................................................   1     1   7y1
......................ucCuacguagcagagcagcucccucgcug..............................................................   3     1   7y1
......................ucguacguagcagagcagcucccucCcug..............................................................   1     1   7y1
......................ucguacguagcagagcagcucccucgcug..............................................................   2333  0   7y1
......................ucguacgAagcagagcagcucccucgcug..............................................................   4     1   7y1
......................Ncguacguagcagagcagcucccucgcug..............................................................   1     1   7y1
......................ucguacguagcagagcagcucccucgcuU..............................................................   6     1   7y1
......................Ccguacguagcagagcagcucccucgcug..............................................................   1     1   7y1
......................ucguacguagcagagcagcCccucgcug...............................................................   2     1   7y1
......................ucguacguagcagagcagcucccucgcGg..............................................................   8     1   7y1
......................ucguacguagUagagcagcucccucgcug..............................................................   1     1   7y1
......................ucguacguagcagagcagcucccucgAug..............................................................   1     1   7y1
......................ucguacguagcagCgcagcucccucgcug..............................................................   2     1   7y1
.......................cguacguagUagagcagc........................................................................   1     1   7y1
.......................cguacguagcaUagcagc........................................................................   1     1   7y1
.......................cguacguagcagaUcagc........................................................................   2     1   7y1
.......................cguacguaAcagagcagc........................................................................   1     1   7y1
.......................cguacguagcagagcCgc........................................................................   1     1   7y1
.......................cguacguagcagagcagc........................................................................   1761  0   7y1
.......................cguacguaUcagagcagc........................................................................   3     1   7y1
.......................cguacguagcUgagcagc........................................................................   1     1   7y1
.......................cguacguagcGgagcagc........................................................................   2     1   7y1
.......................cguacAuagcagagcagc........................................................................   1     1   7y1
.......................cguacguagcagagAagc........................................................................   2     1   7y1
.......................cgAacguagcagagcagc........................................................................   2     1   7y1
.......................cUuacguagcagagcagc........................................................................   3     1   7y1
.......................cguacguagcagagcaUc........................................................................   1     1   7y1
.......................Gguacguagcagagcagc........................................................................   2     1   7y1
.......................cguacgCagcagagcagc........................................................................   2     1   7y1
```

```
      Star                                                                 Mature
gaccugcuucugggucgggguuucguacguagcagagcagcucccucgcugcgaucuauugaaagucagcccucgacacaaggguuuguccgcgcgcgcgcgcgcgugcgugc
.......................cguacguagcagCgcagc........................................................................   2      1    7y1
.......................cguacguagAagagcagc........................................................................   2      1    7y1
.......................cguacgAagcagagcagc........................................................................   1      1    7y1
.......................cguCcguagcagagcagc........................................................................   1      1    7y1
.......................cguacguagcagagcGgc........................................................................   1      1    7y1
.......................cguacNuagcagagcagc........................................................................   1      1    7y1
.......................cguacguagcaAagcagc........................................................................   1      1    7y1
.......................cCuacguagcagagcagc........................................................................   1      1    7y1
.......................cguacguagcagagcagA........................................................................   2      1    7y1
.......................Aguacguagcagagcagc........................................................................   6      1    7y1
.......................Nguacguagcagagcagc........................................................................   2      1    7y1
.......................cAuacguagcagagcagc........................................................................   1      1    7y1
.......................cguaAguagcagagcagc........................................................................   1      1    7y1
.......................cCuacguagcagagcagcu.......................................................................   1      1    7y1
.......................Aguacguagcagagcagcu.......................................................................   5      1    7y1
.......................cguacguagUagagcagcu.......................................................................   1      1    7y1
.......................cguacguagcagagGagcu.......................................................................   2      1    7y1
.......................cUuacguagcagagcagcu.......................................................................   5      1    7y1
.......................cguacguagcagagcagcA.......................................................................   1      1    7y1
.......................cgAacguagcagagcagcu.......................................................................   1      1    7y1
.......................cAuacguagcagagcagcu.......................................................................   2      1    7y1
.......................cguacguagcagaAcagcu.......................................................................   1      1    7y1
.......................cguacguagcagaCcagcu.......................................................................   1      1    7y1
.......................cguacUuagcagagcagcu.......................................................................   1      1    7y1
.......................cguacguagcagagcagcu.......................................................................   1200   0    7y1
.......................cguacguagcagagcagAu.......................................................................   3      1    7y1
.......................cgCacguagcagagcagcu.......................................................................   1      1    7y1
.......................cguacguagAagagcagcu.......................................................................   3      1    7y1
.......................cguaAguagcagagcagcu.......................................................................   2      1    7y1
.......................cguacguagcagagcagcG.......................................................................   4      1    7y1
.......................cguacguagcaUagcagcu.......................................................................   1      1    7y1
.......................Gguacguagcagagcagcu.......................................................................   1      1    7y1
.......................cguacguagcagagAagcu.......................................................................   3      1    7y1
.......................cguacguagcagagcaUcu.......................................................................   1      1    7y1
.......................cguacguagcagCgcagcu.......................................................................   1      1    7y1
.......................Nguacguagcagagcagcu.......................................................................   1      1    7y1
.......................cguacguagcagagcaCcu.......................................................................   2      1    7y1
.......................cguacgAagcagagcagcu.......................................................................   3      1    7y1
.......................cguacguagcagagcaUcuc......................................................................   5      1    7y1
.......................cguacguagcagagcaCcuc......................................................................   4      1    7y1
.......................cguacguaAcagagcagcuc......................................................................   1      1    7y1
.......................cguacguagUagagcagcuc......................................................................   1      1    7y1
.......................cguacguagcagagcagUuc......................................................................   1      1    7y1
.......................cguacguagcagagcagcAc......................................................................   5      1    7y1
.......................cguacguagAagagcagcuc......................................................................   22     1    7y1
.......................cguGcguagcagagcagcuc......................................................................   2      1    7y1
.......................cguacguagcagUgcagcuc......................................................................   1      1    7y1
.......................cUuacguagcagagcagcuc......................................................................   5      1    7y1
.......................cguacguagcagagcagcGc......................................................................   1      1    7y1
.......................cguacguaUcagagcagcuc......................................................................   4      1    7y1
.......................cguacguagcagagUagcuc......................................................................   1      1    7y1
.......................cguacguaCcagagcagcuc......................................................................   2      1    7y1
.......................cguacguagcaUagcagcuc......................................................................   3      1    7y1
.......................cguacguagcagagcagcuA......................................................................   8      1    7y1
.......................cguacguagcagagAagcuc......................................................................   29     1    7y1
.......................cguaGguagcagagcagcuc......................................................................   1      1    7y1
.......................cguacguagcagagGagcuc......................................................................   2      1    7y1
.......................cguacguagcagGgcagcuc......................................................................   3      1    7y1
.......................cguacguagcagagcUgcuc......................................................................   1      1    7y1
.......................cgAacguagcagagcagcuc......................................................................   12     1    7y1
.......................Aguacguagcagagcagcuc......................................................................   25     1    7y1
.......................cguacguagGagagcagcuc......................................................................   1      1    7y1
.......................cguacAuagcagagcagcuc......................................................................   4      1    7y1
.......................cguacguagcagagcagcuG......................................................................   1      1    7y1
.......................cguacgAagcagagcagcuc......................................................................   23     1    7y1
.......................cguacguagcaCagcagcuc......................................................................   2      1    7y1
.......................cguacCuagcagagcagcuc......................................................................   2      1    7y1
.......................Nguacguagcagagcagcuc......................................................................   2      1    7y1
.......................cguacguagcagaCcagcuc......................................................................   4      1    7y1
.......................cCuacguagcagagcagcuc......................................................................   11     1    7y1
```

**Star** **Mature**

```
gaccugcuucugggucgggguuucguacguagcagagcagcucccucgcugcgaucuauugaaagucagcccucgacacaaggguuuguccgcgcgcgcgcgcgcgugcgugc
.......................cguacguagcagagcagcuc......................................................................   7571   0   7y1
.......................cguacguagcagagcGgcuc......................................................................   1      1   7y1
.......................cguacgCagcagagcagcuc......................................................................   2      1   7y1
.......................Gguacguagcagagcagcuc......................................................................   3      1   7y1
.......................cguaAguagcagagcagcuc......................................................................   10     1   7y1
.......................cguacUuagcagagcagcuc......................................................................   5      1   7y1
.......................cguacguagcagagcagAuc......................................................................   9      1   7y1
.......................Uguacguagcagagcagcuc......................................................................   1      1   7y1
.......................cguacguagcagCgcagcuc......................................................................   4      1   7y1
.......................cAuacguagcagagcagcuc......................................................................   1      1   7y1
.......................cguacguagcagaUcagcuc......................................................................   4      1   7y1
.......................cguacguagcGgagcagcuc......................................................................   2      1   7y1
.......................cguacguagcagagcagcAcc.....................................................................   1      1   7y1
.......................cguacguagcagagAagcucc.....................................................................   1      1   7y1
.......................cguacguagcagagcagcuAc.....................................................................   1      1   7y1
.......................cguacguagcagCgcagcucc.....................................................................   1      1   7y1
.......................Aguacguagcagagcagcucc.....................................................................   1      1   7y1
.......................cguacguagcagagcagcucc.....................................................................   434    0   7y1
.......................cUuacguagcagagcagcucc.....................................................................   1      1   7y1
.......................cguacguagcagagNagcucc.....................................................................   1      1   7y1
.......................cguacguagcagaUcagcucc.....................................................................   1      1   7y1
.......................cguacguagcagagGagcucc.....................................................................   1      1   7y1
.......................cguacguaCcagagcagcucc.....................................................................   1      1   7y1
.......................cAuacguagcagagcagcucc.....................................................................   1      1   7y1
.......................cguaAguagcagagcagcucc.....................................................................   1      1   7y1
.......................cguacguagcagagAagcuccc....................................................................   2      1   7y1
.......................cUuacguagcagagcagcuccc....................................................................   3      1   7y1
.......................cguacguagcagagcagcAccc....................................................................   1      1   7y1
.......................cguacguagcagagcagcuccA....................................................................   2      1   7y1
.......................cguacgAagcagagcagcuccc....................................................................   1      1   7y1
.......................cguacguagcagagcagcuccc....................................................................   660    0   7y1
.......................Aguacguagcagagcagcuccc....................................................................   3      1   7y1
.......................cguacguagcagagcaUcuccc....................................................................   2      1   7y1
.......................cguacguagAagagcagcuccc....................................................................   1      1   7y1
.......................cguacguagcagUgcagcuccc....................................................................   1      1   7y1
.......................cguaAguagcagagcagcuccc....................................................................   1      1   7y1
.......................cguacguagcagagcagcuGcc....................................................................   1      1   7y1
.......................cguacguagcagagcagcuAcc....................................................................   1      1   7y1
.......................cguacguagcagagcaUcucccu...................................................................   2      1   7y1
.......................cguacguagcagagcagcucccA...................................................................   1      1   7y1
.......................cguaUguagcagagcagcucccu...................................................................   1      1   7y1
.......................cguacguagcagagcagcucccG...................................................................   6      1   7y1
.......................cguacguagcagagcagcuccAu...................................................................   2      1   7y1
.......................cguacguagcagagcGgcucccu...................................................................   2      1   7y1
.......................cguacguagcagagcagcuAccu...................................................................   2      1   7y1
.......................cguacguagcagagAagcucccu...................................................................   1      1   7y1
.......................cAuacguagcagagcagcucccu...................................................................   1      1   7y1
.......................cgAacguagcagagcagcucccu...................................................................   1      1   7y1
.......................cguacguagcagagcagcAcccu...................................................................   1      1   7y1
.......................cUuacguagcagagcagcucccu...................................................................   2      1   7y1
.......................cCuacguagcagagcagcucccu...................................................................   1      1   7y1
.......................cguacguagcagagcagcucccu...................................................................   943    0   7y1
.......................cguacguagcagaUcagcucccu...................................................................   1      1   7y1
.......................cguacguagcagagcUgcucccu...................................................................   1      1   7y1
.......................cguacguagcaUagcagcucccu...................................................................   1      1   7y1
.......................cguacguagcagagcagcucGcu...................................................................   1      1   7y1
.......................cguacguagAagagcagcucccu...................................................................   1      1   7y1
.......................cguacguagcagagcCgcucccu...................................................................   1      1   7y1
.......................cguaAguagcagagcagcucccu...................................................................   1      1   7y1
.......................cguacguagcagagcagGucccuc..................................................................   1      1   7y1
.......................cguacCuagcagagcagcucccuc..................................................................   2      1   7y1
.......................cguacguagcagagcagcucccAc..................................................................   2      1   7y1
.......................cguacAuagcagagcagcucccuc..................................................................   1      1   7y1
.......................cguacgAagcagagcagcucccuc..................................................................   4      1   7y1
.......................cguacguagcagaUcagcucccuc..................................................................   2      1   7y1
.......................cguacguagcagagcagcucAcuc..................................................................   2      1   7y1
.......................Nguacguagcagagcagcucccuc..................................................................   1      1   7y1
.......................cguacguagcagGgcagcucccuc..................................................................   1      1   7y1
.......................cUuacguagcagagcagcucccuc..................................................................   3      1   7y1
.......................cguacguagcagCgcagcucccuc..................................................................   2      1   7y1
```

**Star** **Mature**

```
gaccugcuucugggucgggguuucguacguagcagagcagcucccucgcugcgaucuauugaaagucagccccucgacacaaggguuuguccgcgcgcgcgcgcgcgugcgugc
.......................cCuacguagcagagcagcucccuc..................................................................  1     1  7y1
.......................cguacguaCcagagcagcucccuc..................................................................  1     1  7y1
.......................cguacguagcagagcagcucccuc..................................................................  744   0  7y1
.......................cguacguagcagagcagcAcccuc..................................................................  1     1  7y1
.......................cguacguagcaCagcagcucccuc..................................................................  1     1  7y1
.......................cguacguagcagagAagcucccuc..................................................................  4     1  7y1
.......................cguacguagcagaAcagcucccuc..................................................................  2     1  7y1
.......................cguacgGagcagagcagcucccuc..................................................................  1     1  7y1
.......................cguacguagcagagcagcuAccuc..................................................................  1     1  7y1
.......................cguacgAagcagagcagcucccucg.................................................................  1     1  7y1
.......................cguacguaUcagagcagcucccucg.................................................................  1     1  7y1
.......................cguacguagcagaUcagcucccucg.................................................................  1     1  7y1
.......................cguacguagcagGgcagcucccucg.................................................................  1     1  7y1
.......................cguacguagcagagcagcucccucg.................................................................  297   0  7y1
.......................cguacguagcagagAagcucccucg.................................................................  1     1  7y1
.......................cguacUuagcagagcagcucccucg.................................................................  1     1  7y1
.......................cUuacguagcagagcagcucccucg.................................................................  1     1  7y1
.......................Aguacguagcagagcagcucccucg.................................................................  2     1  7y1
.......................cguaAguagcagagcagcucccucgc................................................................  1     1  7y1
.......................cguacguagcagUgcagcucccucgc................................................................  1     1  7y1
.......................cguacCuagcagagcagcucccucgc................................................................  1     1  7y1
.......................cguacguagcagagcagcucccucCc................................................................  1     1  7y1
.......................cguacguagcagagcagAucccucgc................................................................  1     1  7y1
.......................cguacguagcagagcagcucccucgc................................................................  264   0  7y1
.......................cguacguagcagagAagcucccucgc................................................................  1     1  7y1
.......................cguacgAagcagagcagcucccucgc................................................................  1     1  7y1
.......................cguacguagcagagcagAucccucgcu...............................................................  1     1  7y1
.......................cguacguagcagagcagcucccGcgcu...............................................................  1     1  7y1
.......................cguacguagcaUagcagcucccucgcu...............................................................  1     1  7y1
.......................cguacguagAagagcagcucccucgcu...............................................................  1     1  7y1
.......................cUuacguagcagagcagcucccucgcu...............................................................  3     1  7y1
.......................cguacguagcagagcagcucccucgcu...............................................................  407   0  7y1
.......................Aguacguagcagagcagcucccucgcu...............................................................  2     1  7y1
.......................cguacgAagcagagcagcucccucgcu...............................................................  2     1  7y1
.......................cguaUguagcagagcagcucccucgcu...............................................................  264   1  7y1
.......................cguaAguagcagagcagcucccucgcu...............................................................  2     1  7y1
.......................cCuacguagcagagcagcucccucgcu...............................................................  1     1  7y1
.......................cguacguagcagagcagcucccucgcG...............................................................  3     1  7y1
.......................cguacguagcagagcagcuGccucgcu...............................................................  1     1  7y1
.......................cAuacguagcagagcagcucccucgcug..............................................................  2     1  7y1
.......................cguacguagcagagcagcucAcucgcug..............................................................  1     1  7y1
.......................cguacguagcagagcagcucccucgcuC..............................................................  1     1  7y1
.......................cguacguaCcagagcagcucccucgcug..............................................................  2     1  7y1
.......................Aguacguagcagagcagcucccucgcug..............................................................  7     1  7y1
.......................cguacguagcagagcagcucccucUcug..............................................................  2     1  7y1
.......................cguacguagcagagcagcucccucgcGg..............................................................  8     1  7y1
.......................cCuacguagcagagcagcucccucgcug..............................................................  1     1  7y1
.......................cUuacguagcagagcagcucccucgcug..............................................................  2     1  7y1
.......................Nguacguagcagagcagcucccucgcug..............................................................  2     1  7y1
.......................cguacguagcagagcagcuAccucgcug..............................................................  2     1  7y1
.......................cguacguagcGgagcagcucccucgcug..............................................................  1     1  7y1
.......................Gguacguagcagagcagcucccucgcug..............................................................  1     1  7y1
.......................cgCacguagcagagcagcucccucgcug..............................................................  1     1  7y1
.......................cguacguagcagagcagcucccucgcuA..............................................................  1     1  7y1
.......................cguacguagcagagcagcucccucgcug..............................................................  1992  0  7y1
.......................cguaAguagcagagcagcucccucgcug..............................................................  1     1  7y1
.......................cguacguagcagagcagcuccAucgcug..............................................................  1     1  7y1
.......................cguacguagcagCgcagcucccucgcug..............................................................  1     1  7y1
.......................cguacgAagcagagcagcucccucgcug..............................................................  2     1  7y1
.......................cguacUuagcagagcagcucccucgcug..............................................................  1     1  7y1
.......................cguacguagcagagAagcucccucgcug..............................................................  2     1  7y1
.......................cguacguagcagagcagcucccucgcuU..............................................................  1     1  7y1
.......................cguacCuagcagagcagcucccucgcug..............................................................  2     1  7y1
.......................cguacguagcagagcagcuGccucgcug..............................................................  1     1  7y1
.......................cgGacguagcagagcagcucccucgcug..............................................................  1     1  7y1
.......................cguacguagcagagcagcucccucgcAg..............................................................  4     1  7y1
.......................cgAacguagcagagcagcucccucgcug..............................................................  2     1  7y1
.......................cguacguagcagagcagcucGcucgcug..............................................................  1     1  7y1
.......................cguacguagcagagcagcucccuAgcug..............................................................  1     1  7y1
.......................cguacguagcaUagcagcucccucgcug..............................................................  1     1  7y1
```

```
Star                                                                   Mature
gaccugcuucugggucggggguuucguacguagcagagcagcucccucgcugcgaucuauugaaagucagcccucgacacaaggguuuguccgcgcgcgcgcgcgcgugcgugc
.......................cguacguagAagagcagcucccucgcug..............................................................	3	1	7y1
.......................cguacguagcagagcagcucccucgAug..............................................................	2	1	7y1
........................gAacguagcagagcagcu.......................................................................	2	1	7y1
........................guaAguagcagagcagcu.......................................................................	1	1	7y1
........................guacguagcagagcagcu.......................................................................	154	0	7y1
........................Cuacguagcagagcagcuc......................................................................	2	1	7y1
........................guacguagcagagcagAuc......................................................................	1	1	7y1
........................guacguagcagagcagcuc......................................................................	173	0	7y1
........................guacgAagcagagcagcuc......................................................................	1	1	7y1
........................Cuacguagcagagcagcucccucgc................................................................	1	1	7y1
........................guacguagcagagcagcucccucgcu...............................................................	164	0	7y1
........................guacguagcagagcagAucccucgcu...............................................................	2	1	7y1
........................guacguagcagagAagcucccucgcu...............................................................	1	1	7y1
........................Uuacguagcagagcagcucccucgcu...............................................................	1	1	7y1
........................guacguagcagagcagcucccucgcG...............................................................	3	1	7y1
........................guacgAagcagagcagcucccucgcu...............................................................	1	1	7y1
........................guacguaUcagagcagcucccucgcu...............................................................	1	1	7y1
........................guGcguagcagagcagcucccucgcu...............................................................	1	1	7y1
........................guacguagcagagcagGucccucgcu...............................................................	1	1	7y1
........................guacguagcagagcGgcucccucgcug..............................................................	1	1	7y1
........................guaAguagcagagcagcucccucgcug..............................................................	1	1	7y1
........................guacguagcagagcagcucccucgcug..............................................................	61	0	7y1
.........................uacguagcagagcagcuA......................................................................	2	1	7y1
.........................uacguagcagagcaAcuc......................................................................	1	1	7y1
.........................uacguagcagagAagcuc......................................................................	1	1	7y1
.........................uacguagcagagcagcuc......................................................................	616	0	7y1
.........................uacguaUcagagcagcuc......................................................................	2	1	7y1
.........................Aacguagcagagcagcuc......................................................................	15	1	7y1
.........................uaAguagcagagcagcuc......................................................................	1	1	7y1
.........................uacguagcagagGagcuc......................................................................	1	1	7y1
.........................uacguagcagagcagcAc......................................................................	2	1	7y1
.........................uacguagcagagcCgcuc......................................................................	1	1	7y1
.........................uacguagcagagcagcuG......................................................................	1	1	7y1
.........................Gacguagcagagcagcuccc....................................................................	1	1	7y1
.........................uacguagcGgagcagcuccc....................................................................	1	1	7y1
.........................uacguagcagCgcagcuccc....................................................................	1	1	7y1
.........................uacguagcagagcagcAccc....................................................................	1	1	7y1
.........................Aacguagcagagcagcuccc....................................................................	3	1	7y1
.........................uacguagcagagcagcuccc....................................................................	193	0	7y1
.........................uacguagcagagcaCcucccucgcu...............................................................	1	1	7y1
.........................uaAguagcagagcagcucccucgcu...............................................................	1	1	7y1
.........................uacguagcagagcUgcucccucgcu...............................................................	1	1	7y1
.........................uacguagcagagcagcucccucgcu...............................................................	493	0	7y1
.........................Nacguagcagagcagcucccucgcu...............................................................	1	1	7y1
.........................uacguagcagagcagcuAccucgcu...............................................................	1	1	7y1
.........................uacguagcagagAagcucccucgcu...............................................................	2	1	7y1
.........................Aacguagcagagcagcucccucgcu...............................................................	5	1	7y1
.........................uacguagcagagcagcucccucUcu...............................................................	1	1	7y1
.........................uacguagcagagcagcucccucgcG...............................................................	4	1	7y1
.........................uacguagcaCagcagcucccucgcu...............................................................	2	1	7y1
.........................uacguagcagagcagcucccuUgcu...............................................................	1	1	7y1
.........................uacguaCcagagcagcucccucgcu...............................................................	2	1	7y1
.........................uacguagcagagcagcucccucgGu...............................................................	1	1	7y1
.........................uacguagcagagcaAcucccucgcug..............................................................	1	1	7y1
.........................uGcguagcagagcagcucccucgcug..............................................................	1	1	7y1
.........................uacguagcagagcagcucccAcgcug..............................................................	1	1	7y1
.........................uacguagcagagcagcucAcucgcug..............................................................	4	1	7y1
.........................uacguagcagagcagcucccucgcuA..............................................................	1	1	7y1
.........................uacguagcagagcagcucccucgcuC..............................................................	2	1	7y1
.........................uacguGgcagagcagcucccucgcug..............................................................	1	1	7y1
.........................uacgAagcagagcagcucccucgcug..............................................................	2	1	7y1
.........................uacguagcagagcagcAcccucgcug..............................................................	1	1	7y1
.........................uacguagcagagcagcucccuAgcug..............................................................	1	1	7y1
.........................uacguagcagagcagcuccGucgcug..............................................................	1	1	7y1
.........................uacguagcagagcaUcucccucgcug..............................................................	2	1	7y1
.........................uacguagcagagcagcucGcucgcug..............................................................	1	1	7y1
.........................uacguagcagagcagcuAccucgcug..............................................................	2	1	7y1
.........................uacguagcUgagcagcucccucgcug..............................................................	1	1	7y1
.........................uacguagcagagcagcucccucgcGg..............................................................	19	1	7y1
.........................uacguagcagagcaNcucccucgcug..............................................................	1	1	7y1
```

**Star** **Mature**

```
gaccugcuucugggucgggguuucguacguagcagagcagcucccucgcugcgaucuauugaaagucagcccucgacacaaggguuuguccgcgcgcgcgcgcgcgugcgugc
.........................uacguagcagagcagcucccucgcAg..............................................................  1     1  7y1
.........................uacguagcagagcagcucccucgAug..............................................................  2     1  7y1
.........................Aacguagcagagcagcucccucgcug..............................................................  17    1  7y1
.........................uacguUgcagagcagcucccucgcug..............................................................  1     1  7y1
.........................uacguagcagagcagcucccucgcCg..............................................................  1     1  7y1
.........................uUcguagcagagcagcucccucgcug..............................................................  1     1  7y1
.........................uacguagcagagcagcucccucgcug..............................................................  1768  0  7y1
.........................uacguaCcagagcagcucccucgcug..............................................................  1     1  7y1
.........................uacguagAagagcagcucccucgcug..............................................................  3     1  7y1
.........................uaAguagcagagcagcucccucgcug..............................................................  5     1  7y1
.........................uacguagcagagcCgcucccucgcug..............................................................  1     1  7y1
.........................uaGguagcagagcagcucccucgcug..............................................................  3     1  7y1
.........................uacguagcagagcagcucccucgcuU..............................................................  1     1  7y1
.........................Gacguagcagagcagcucccucgcug..............................................................  2     1  7y1
.........................uacguaUcagagcagcucccucgcug..............................................................  1     1  7y1
..........................acguagcagaUcagcucccucgcu...............................................................  1     1  7y1
..........................acguagcagagcagcucccucgcu...............................................................  416   0  7y1
..........................acguagcagagcagcucccucgcG...............................................................  9     1  7y1
..........................Gcguagcagagcagcucccucgcu...............................................................  5     1  7y1
..........................acguagcagagcagAucccucgcu...............................................................  1     1  7y1
..........................acguagcGgagcagcucccucgcu...............................................................  2     1  7y1
..........................acguagcagagcagcucccAcgcu...............................................................  1     1  7y1
..........................acguagcagagcaUcucccucgcu...............................................................  3     1  7y1
..........................acguaCcagagcagcucccucgcu...............................................................  1     1  7y1
..........................acgAagcagagcagcucccucgcu...............................................................  1     1  7y1
..........................acguaUcagagcagcucccucgcu...............................................................  1     1  7y1
..........................acguagcagagAagcucccucgcu...............................................................  1     1  7y1
..........................aAguagcagagcagcucccucgcu...............................................................  2     1  7y1
..........................acguagcagagcagcuAccucgcu...............................................................  1     1  7y1
..........................acguagAagagcagcucccucgcu...............................................................  2     1  7y1
..........................acguagcagagcCgcucccucgcu...............................................................  1     1  7y1
..........................acguagcaUagcagcucccucgcug..............................................................  1     1  7y1
..........................acguagAagagcagcucccucgcug..............................................................  2     1  7y1
..........................acguagcagaUcagcucccucgcug..............................................................  2     1  7y1
..........................acguagcagagAagcucccucgcug..............................................................  1     1  7y1
..........................acguagcagagcagcucccucgcGg..............................................................  5     1  7y1
..........................acguagcagagcGgcucccucgcug..............................................................  1     1  7y1
..........................acguagcagagcagcucccucgcug..............................................................  537   0  7y1
..........................acgAagcagagcagcucccucgcug..............................................................  1     1  7y1
..........................acCuagcagagcagcucccucgcug..............................................................  1     1  7y1
..........................acguagcagagcagcucccucgcuC..............................................................  1     1  7y1
..........................acguagcagagcagcuccAucgcug..............................................................  2     1  7y1
..........................Gcguagcagagcagcucccucgcug..............................................................  1     1  7y1
...........................cguagcagagcagcuccc....................................................................  199   0  7y1
...........................cguagcagagcagcucGc....................................................................  1     1  7y1
...........................cguagcagagcagcucAcu...................................................................  1     1  7y1
...........................cguagAagagcagcucccu...................................................................  3     1  7y1
...........................cguagcagagcagcuAccu...................................................................  2     1  7y1
...........................cguagcagaUcagcucccu...................................................................  1     1  7y1
...........................cgGagcagagcagcucccu...................................................................  1     1  7y1
...........................cguagcagagcagcucccu...................................................................  204   0  7y1
...........................cguagcagaCcagcucccu...................................................................  1     1  7y1
...........................cguaCcagagcagcucccucgcug..............................................................  1     1  7y1
...........................cCuagcagagcagcucccucgcug..............................................................  1     1  7y1
...........................cguagcagagcagcuUccucgcug..............................................................  1     1  7y1
...........................cguagcagagcagcucccucAcug..............................................................  1     1  7y1
...........................cguagcagCgcagcucccucgcug..............................................................  1     1  7y1
...........................cguagcagagcagcucccucgAug..............................................................  1     1  7y1
...........................cguagcagagcagcucccucgcuU..............................................................  2     1  7y1
...........................cguagcagagcagcucccucgcug..............................................................  541   0  7y1
...........................cguaUcagagcagcucccucgcug..............................................................  1     1  7y1
...........................cguagcagagcagAucccucgcug..............................................................  1     1  7y1
...........................Nguagcagagcagcucccucgcug..............................................................  2     1  7y1
...........................cguagcagagcagcucccAcgcug..............................................................  1     1  7y1
...........................cgAagcagagcagcucccucgcug..............................................................  1     1  7y1
...........................Gguagcagagcagcucccucgcug..............................................................  1     1  7y1
...........................cguagcagagcagcuGccucgcug..............................................................  1     1  7y1
...........................cguagcagagcagcAcccucgcug..............................................................  1     1  7y1
............................guagcagagcagcucccucgc................................................................  2     0  7y1
............................guagcagagcagcucccucgcug..............................................................  73    0  7y1
```

**Star** **Mature**

```
gaccugcuucugggucgggguuucguacguagcagagcagcucccucgcugcgaucuauugaaagucagcccucgacacaaggguuuguccgcgcgcgcgcgcgcgugcgugc
..............................agcagagcagcucGcucgcug..............................................................  1    1  7y1
..............................aUcagagcagcucccucgcug..............................................................  1    1  7y1
..............................agcagagcagcuccAucgcug..............................................................  2    1  7y1
..............................agcagagcagcucccucgcug..............................................................  273  0  7y1
..............................agcagagAagcucccucgcug..............................................................  1    1  7y1
..............................agcagagcagcuAccucgcug..............................................................  1    1  7y1
..............................aAcagagcagcucccucgcug..............................................................  1    1  7y1
..............................agcagagcagcucAcucgcug..............................................................  1    1  7y1
..............................agcagagcagcucccucgAug..............................................................  1    1  7y1
................................cagagcaUcucccucgcu...............................................................  1    1  7y1
................................cagagcagcucccucgcu...............................................................  129  0  7y1
................................cagCgcagcucccucgcu...............................................................  1    1  7y1
................................cagagcagcucAcucgcu...............................................................  1    1  7y1
................................cagagcagcucccucgcG...............................................................  11   1  7y1
................................cagagcagcuccGucgcu...............................................................  1    1  7y1
................................cagagcagcAcccucgcu...............................................................  1    1  7y1
....................................gcagcuUccucgcugcgaucu........................................................  1    1  7y1
....................................gcagcucAcucgcugcgaucu........................................................  1    1  7y1
....................................gcagcuAccucgcugcgaucu........................................................  3    1  7y1
....................................gAagcucccucgcugcgaucu........................................................  1    1  7y1
....................................gcagcucccucgcuCcgaucu........................................................  1    1  7y1
....................................gcagcucccucgcugcgaucu........................................................  394  0  7y1
....................................gcagcucccucCcugcgaucu........................................................  1    1  7y1
....................................gcagcucccucgAugcgaucu........................................................  1    1  7y1
....................................gcagcucccucgcugcgaucA........................................................  1    1  7y1
....................................gcagcucccucgcAgcgaucu........................................................  2    1  7y1
....................................gcagcucccucgcugcgGucu........................................................  1    1  7y1
....................................gcagAucccucgcugcgaucu........................................................  1    1  7y1
....................................gcagcucccucgGugcgaucu........................................................  1    1  7y1
....................................gcagcucccucgcCgcgaucu........................................................  1    1  7y1
....................................gcagcucccucgcugcgaucuauugaaagu...............................................  224  0  7y1
....................................Ccagcucccucgcugcgaucuauugaaagu...............................................  1    1  7y1
....................................gcagcucccucgcugcgaucuauugaaagG...............................................  1    1  7y1
....................................gcagcucccuAgcugcgaucuauugaaagu...............................................  1    1  7y1
....................................gcagcucccucUcugcgaucuauugaaagu...............................................  1    1  7y1
....................................gcagcucccucgcugcgaucuaAugaaagu...............................................  1    1  7y1
....................................gcagcuUccucgcugcgaucuauugaaagu...............................................  1    1  7y1
....................................Ncagcucccucgcugcgaucuauugaaaguca.............................................  1    1  7y1
....................................gcagcucccucgcugcgaucuauugaaaguca.............................................  182  0  7y1
....................................gcagcucccucgcugcgaucAauugaaaguca.............................................  1    1  7y1
....................................gcagcucAcucgcugcgaucuauugaaaguca.............................................  2    1  7y1
....................................gcagcucccuAgcugcgaucuauugaaaguca.............................................  1    1  7y1
....................................gcagcucccucgcugcgauAuauugaaaguca.............................................  1    1  7y1
....................................gcagcucccucgcugcgaucuauAgaaaguca.............................................  1    1  7y1
....................................gcagGucccucgcugcgaucuauugaaaguca.............................................  1    1  7y1
....................................gcagcucccuUgcugcgaucuauugaaaguca.............................................  1    1  7y1
....................................gcagcucccucgcugAgaucuauugaaaguca.............................................  2    1  7y1
....................................gcagcuAccucgcugcgaucuauugaaaguca.............................................  3    1  7y1
.....................................cagcucccucgcugcgaucAauugaaagucag............................................  1    1  7y1
.....................................cagcucccucgcugcgaucuaGugaaagucag............................................  1    1  7y1
.....................................cagcucccucgcugcgaucuauugaaagucag............................................  188  0  7y1
.....................................cagcucccucgcugcgaAcuauugaaagucag............................................  1    1  7y1
.....................................cagcAcccucgcugcgaucuauugaaagucag............................................  2    1  7y1
.....................................cagcucccucgcugcgaucuauugaaagAcag............................................  1    1  7y1
.....................................cagcuAccucgcugcgaucuauugaaagucag............................................  1    1  7y1
.....................................cagcucccucgcugAgaucuauugaaagucag............................................  1    1  7y1
.....................................cagUucccucgcugcgaucuauugaaagucag............................................  1    1  7y1
.....................................cagcucccuAgcugcgaucuauugaaagucag............................................  1    1  7y1
.....................................cagcucccucAcugcgaucuauugaaagucag............................................  1    1  7y1
.....................................cagcuccAucgcugcgaucuauugaaagucag............................................  2    1  7y1
.....................................Aagcucccucgcugcgaucuauugaaagucag............................................  2    1  7y1
.....................................cagcuUccucgcugcgaucuauugaaagucag............................................  1    1  7y1
......................................agcucccucgcugcNaucu........................................................  1    1  7y1
......................................agcucccucgcugcgUucu........................................................  1    1  7y1
......................................agcucccucgcugcgaucG........................................................  1    1  7y1
......................................agcuccAucgcugcgaucu........................................................  1    1  7y1
......................................agcucccucgcugcgaucu........................................................  330  0  7y1
......................................agcuAccucgcugcgaucu........................................................  1    1  7y1
......................................agAucccucgcugcgaucu........................................................  1    1  7y1
......................................agcucccuAgcugcgaucu........................................................  1    1  7y1
```

**Star** **Mature**

| | | | |
|---|---|---|---|
| gaccugcuucugggucgggguuucguacguagcagagcagcucccucgcugcgaucuauugaaagucagcccucgacacaagguuuguccgcgcgcgcgcgcgcgugcgugc | | | |
| ......................................agcucccucgcugAgaucu....................................................... | 1 | 1 | 7y1 |
| ......................................Cgcucccucgcugcgaucu....................................................... | 2 | 1 | 7y1 |
| ......................................agcucAcucgcugcgaucu....................................................... | 1 | 1 | 7y1 |
| ......................................Cgcucccucgcugcgaucuauugaaaguc............................................. | 1 | 1 | 7y1 |
| ......................................agcucccucgcAgcgaucuauugaaaguc............................................. | 1 | 1 | 7y1 |
| ......................................agcucccucgcugcgaucuauugaaaguc............................................. | 185 | 0 | 7y1 |
| ......................................agcucAcucgcugcgaucuauugaaaguc............................................. | 2 | 1 | 7y1 |
| ......................................agcCcccucgcugcgaucuauugaaaguc............................................. | 1 | 1 | 7y1 |
| ......................................agcuAccucgcugcgaucuauugaaaguc............................................. | 2 | 1 | 7y1 |
| ......................................agcuccAucgcugcgaucuauugaaaguc............................................. | 1 | 1 | 7y1 |
| ......................................agcucccucgcugAgaucuauugaaaguc............................................. | 1 | 1 | 7y1 |
| ......................................agcucccucgcugcgaucuauugaaagucag........................................... | 1 | 0 | 7y1 |
| .......................................gcucccucgcugcgaucG....................................................... | 1 | 1 | 7y1 |
| .......................................Ccucccucgcugcgaucu....................................................... | 1 | 1 | 7y1 |
| .......................................gcucccucgcugcgaucu....................................................... | 194 | 0 | 7y1 |
| .......................................gcucccAcgcugcgaucu....................................................... | 1 | 1 | 7y1 |
| .......................................gcucccucUcugcgaucu....................................................... | 1 | 1 | 7y1 |
| .......................................gcucccucgAugcgaucu....................................................... | 1 | 1 | 7y1 |
| .......................................gcuAccucgcugcgaucu....................................................... | 1 | 1 | 7y1 |
| .......................................gcucAcucgcugcgaucuauug................................................... | 3 | 1 | 7y1 |
| .......................................gcuAccucgcugcgaucuauug................................................... | 1 | 1 | 7y1 |
| .......................................gcuccAucgcugcgaucuauug................................................... | 2 | 1 | 7y1 |
| .......................................Ccucccucgcugcgaucuauug................................................... | 1 | 1 | 7y1 |
| .......................................gcucccucgcugcgaucuauAg................................................... | 1 | 1 | 7y1 |
| .......................................gcucccucgcugcgaucuauug................................................... | 515 | 0 | 7y1 |
| .......................................gcucccucgcugcgaucAauug................................................... | 1 | 1 | 7y1 |
| .......................................gcucUcucgcugcgaucuauug................................................... | 1 | 1 | 7y1 |
| .......................................gcucccuAgcugcgaucuauug................................................... | 1 | 1 | 7y1 |
| .......................................gcucccucgcugcgaucuauCg................................................... | 1 | 1 | 7y1 |
| .......................................gcucccucgcugcgaucuauuU................................................... | 1 | 1 | 7y1 |
| .......................................gcucccucgcugcgUucuauug................................................... | 1 | 1 | 7y1 |
| .......................................gcucccucgcugcgaucuauugaGag............................................... | 1 | 1 | 7y1 |
| .......................................gcucccucCcugcgaucuauugaaag............................................... | 1 | 1 | 7y1 |
| .......................................gcucccucgcugcgaucuUuugaaag............................................... | 1 | 1 | 7y1 |
| .......................................gcucccucgcugcgaucuauugaaGg............................................... | 1 | 1 | 7y1 |
| .......................................gcucAcucgcugcgaucuauugaaag............................................... | 2 | 1 | 7y1 |
| .......................................gcucccucgcugcgaucuauGgaaag............................................... | 1 | 1 | 7y1 |
| .......................................gcucccucgcugcgauAuauugaaag............................................... | 1 | 1 | 7y1 |
| .......................................gcucccucgcugcUaucuauugaaag............................................... | 1 | 1 | 7y1 |
| .......................................gcuUccucgcugcgaucuauugaaag............................................... | 1 | 1 | 7y1 |
| .......................................gcucccucAcugcgaucuauugaaag............................................... | 1 | 1 | 7y1 |
| .......................................gcucccucgcGgcgaucuauugaaag............................................... | 1 | 1 | 7y1 |
| .......................................gcucccucgcugcgaucuauugGaag............................................... | 1 | 1 | 7y1 |
| .......................................gcucccucgcuCcgaucuauugaaag............................................... | 1 | 1 | 7y1 |
| .......................................gcuAccucgcugcgaucuauugaaag............................................... | 2 | 1 | 7y1 |
| .......................................gcucccucgcugAgaucuauugaaag............................................... | 1 | 1 | 7y1 |
| .......................................gNucccucgcugcgaucuauugaaag............................................... | 1 | 1 | 7y1 |
| .......................................gcucccuAgcugcgaucuauugaaag............................................... | 1 | 1 | 7y1 |
| .......................................gcucccucgcugcgaucuauugaaaC............................................... | 2 | 1 | 7y1 |
| .......................................gcucccucgAugcgaucuauugaaag............................................... | 1 | 1 | 7y1 |
| .......................................gcucccucgcugcgaucuauugaaag............................................... | 1199 | 0 | 7y1 |
| .......................................Ncucccucgcugcgaucuauugaaag............................................... | 1 | 1 | 7y1 |
| .......................................gcuccAucgcugcgaucuauugaaag............................................... | 1 | 1 | 7y1 |
| .......................................gcucccucgcAgcgaucuauugaaag............................................... | 1 | 1 | 7y1 |
| .......................................gcucccAcgcugcgaucuauugaaag............................................... | 2 | 1 | 7y1 |
| .......................................Ccucccucgcugcgaucuauugaaag............................................... | 2 | 1 | 7y1 |
| .......................................gAucccucgcugcgaucuauugaaag............................................... | 3 | 1 | 7y1 |
| .......................................gcucccucgcugcgaAcuauugaaagu.............................................. | 1 | 1 | 7y1 |
| .......................................gcucccucgcCgcgaucuauugaaagu.............................................. | 1 | 1 | 7y1 |
| .......................................gcucccucgcAgcgaucuauugaaagu.............................................. | 1 | 1 | 7y1 |
| .......................................gcucAcucgcugcgaucuauugaaagu.............................................. | 2 | 1 | 7y1 |
| .......................................gcucccucgcugcgaucCauugaaagu.............................................. | 1 | 1 | 7y1 |
| .......................................gcucccucgcuCcgaucuauugaaagu.............................................. | 1 | 1 | 7y1 |
| .......................................gcucccucgcugcgaucAauugaaagu.............................................. | 1 | 1 | 7y1 |
| .......................................gcuccAucgcugcgaucuauugaaagu.............................................. | 1 | 1 | 7y1 |
| .......................................gcucccucgcugcgaucuauAgaaagu.............................................. | 1 | 1 | 7y1 |
| .......................................gcucccucgcugcgaucuauugaaaUu.............................................. | 1 | 1 | 7y1 |
| .......................................gcucccucgcugcgaucuauugaaagu.............................................. | 906 | 0 | 7y1 |
| .......................................gcuAccucgcugcgaucuauugaaagu.............................................. | 1 | 1 | 7y1 |
| .......................................gcucccucgcugcUaucuauugaaagu.............................................. | 1 | 1 | 7y1 |

```
Star                                                              Mature
gaccugcuucugggucgggguuucguacguagcagagcagcucccucgcugcgaucuauugaaagucagcccucgacacaaggguuuguccgcgcgcgcgcgcgcgugcgugc
.......................................gcucccucgcugcgauGuauugaaagu...............................................   1     1   7y1
.......................................gcucccuAgcugcgaucuauugaaagu...............................................   2     1   7y1
.......................................gcucccucgcugcgaucuauugaaagA...............................................   1     1   7y1
.......................................Ccucccucgcugcgaucuauugaaagu...............................................   2     1   7y1
.......................................Ucucccucgcugcgaucuauugaaagu...............................................   1     1   7y1
.......................................gcucccucgcugcgaucuaGugaaagu...............................................   1     1   7y1
.......................................gcucccucgcugcgaucuauugaaagG...............................................   1     1   7y1
.......................................gcucccucgAugcgaucuauugaaagu...............................................   2     1   7y1
.......................................gcucccucgcugcgaucuauugaaaguU..............................................   1     1   7y1
.......................................gcuGccucgcugcgaucuauugaaaguc..............................................   1     1   7y1
.......................................gcucccucgcugcgaucuauugaaaguc..............................................   229   0   7y1
.......................................gcucccucgcugAgaucuauugaaaguc..............................................   1     1   7y1
.......................................gcucAcucgcugcgaucuauugaaaguc..............................................   1     1   7y1
.......................................gcuccAucgcugcgaucuauugaaaguc..............................................   1     1   7y1
.......................................Ucucccucgcugcgaucuauugaaaguc..............................................   1     1   7y1
........................................cucccucgcugcgaucuaAugaa..................................................   1     1   7y1
........................................cucccAcgcugcgaucuauugaa..................................................   1     1   7y1
........................................cucccucgcugAgaucuauugaa..................................................   2     1   7y1
........................................cucccucgcugcgauAuauugaa..................................................   1     1   7y1
........................................cucccucgcAgcgaucuauugaa..................................................   1     1   7y1
........................................cucccucgcugcgaAcuauugaa..................................................   1     1   7y1
........................................cucccucgcugcCaucuauugaa..................................................   1     1   7y1
........................................cucccucgcugcgaucuauugaa..................................................   144   0   7y1
........................................cucccucgcugcgaucuauugaaa.................................................   244   0   7y1
........................................cucccucgcugcgaucuauGgaaa.................................................   1     1   7y1
........................................cucccucgcugAgaucuauugaaa.................................................   1     1   7y1
........................................cuAccucgcugcgaucuauugaaa.................................................   3     1   7y1
........................................Aucccucgcugcgaucuauugaaa.................................................   2     1   7y1
........................................cucccucgcuCcgaucuauugaaa.................................................   1     1   7y1
........................................cucccuAgcugcgaucuauugaaag................................................   1     1   7y1
........................................cucccucgcuCcgaucuauugaaag................................................   1     1   7y1
........................................cucccucgcuUcgaucuauugaaag................................................   1     1   7y1
........................................cuccAucgcugcgaucuauugaaag................................................   1     1   7y1
........................................cucAcucgcugcgaucuauugaaag................................................   1     1   7y1
........................................cucccucgcugcgaucuauugaaag................................................   185   0   7y1
........................................cucccucgcuAcgaucuauugaaagu...............................................   1     1   7y1
........................................Aucccucgcugcgaucuauugaaagu...............................................   6     1   7y1
........................................cucccucgAugcgaucuauugaaagu...............................................   2     1   7y1
........................................cucAcucgcugcgaucuauugaaagu...............................................   1     1   7y1
........................................cucccucgcugcgaucuauAgaaagu...............................................   1     1   7y1
........................................cucccucgcuCcgaucuauugaaagu...............................................   2     1   7y1
........................................cucccucCcugcgaucuauugaaagu...............................................   2     1   7y1
........................................cucccucgcAgcgaucuauugaaagu...............................................   2     1   7y1
........................................cucccucgcugcUaucuauugaaagu...............................................   1     1   7y1
........................................cucccucgcugcgaucuauugaaaUu...............................................   1     1   7y1
........................................cucccucgcugcgaucuauugaaagA...............................................   5     1   7y1
........................................cucccucUcugcgaucuauugaaagu...............................................   3     1   7y1
........................................cucccucgcugcgaucuaAugaaagu...............................................   1     1   7y1
........................................cuccUucgcugcgaucuauugaaagu...............................................   1     1   7y1
........................................cucccucgcugcgCucuauugaaagu...............................................   1     1   7y1
........................................cucccucgcugcgaucuauugaaagG...............................................   1     1   7y1
........................................cuAccucgcugcgaucuauugaaagu...............................................   9     1   7y1
........................................cucccucgcugAgaucuauugaaagu...............................................   2     1   7y1
........................................cucccuAgcugcgaucuauugaaagu...............................................   4     1   7y1
........................................cucccucgcugcgaucuauugGaagu...............................................   1     1   7y1
........................................cucccucgcugcgaucuauuAaaagu...............................................   1     1   7y1
........................................cAcccucgcugcgaucuauugaaagu...............................................   7     1   7y1
........................................cucccucgcugcgaucuauugaUagu...............................................   1     1   7y1
........................................cucccucgcugcgaucAauugaaagu...............................................   2     1   7y1
........................................cucccucgcugcgaucuauugaaagu...............................................   1893  0   7y1
........................................cucccucgcugcgaucuauugaaaCu...............................................   1     1   7y1
........................................cucccAcgcugcgaucuauugaaagu...............................................   5     1   7y1
........................................cucccucgcugcgaucuauugaUaguc..............................................   1     1   7y1
........................................cucccucgcugcUaucuauugaaaguc..............................................   3     1   7y1
........................................cucccucgcugcgaucuauAgaaaguc..............................................   8     1   7y1
........................................cucccucgcugcgaucuauuCaaaguc..............................................   1     1   7y1
........................................cucccucgcuCcgaucuauugaaaguc..............................................   3     1   7y1
........................................cuAccucgcugcgaucuauugaaaguc..............................................   10    1   7y1
........................................cucccucgcugcgaAcuauugaaaguc..............................................   3     1   7y1
........................................Nucccucgcugcgaucuauugaaaguc..............................................   3     1   7y1
```

**Star** **Mature**

```
gaccugcuucugggucgggguuucguacguagcagagcagcucccucgcugcgaucuauugaaagucagcccucgacacaaggguuuguccgcgcgcgcgcgcgcgugcgugc
........................................cucccucgcugcgaucuauugaaaguA..............................................	4	1	7y1
........................................cuccuUgcugcgaucuauugaaaguc..............................................	1	1	7y1
........................................cuGccucgcugcgaucuauugaaaguc..............................................	1	1	7y1
........................................cucUcucgcugcgaucuauugaaaguc..............................................	2	1	7y1
........................................cucccucgcugAgaucuauugaaaguc..............................................	12	1	7y1
........................................cucAcucgcugcgaucuauugaaaguc..............................................	5	1	7y1
........................................cucccucgcugcgaucuauugGaaguc..............................................	2	1	7y1
........................................cucccucgAugcgaucuauugaaaguc..............................................	1	1	7y1
........................................cucccucgcuUcgaucuauugaaaguc..............................................	6	1	7y1
........................................cucccucgcugcgaGcuauugaaaguc..............................................	2	1	7y1
........................................cucccucUcugcgaucuauugaaaguc..............................................	7	1	7y1
........................................cucccucgcugcgaucuaCugaaaguc..............................................	1	1	7y1
........................................cucccucgcugcgaucuauugaaagAc..............................................	3	1	7y1
........................................cucccucgcugcgaucuauugaaaAuc..............................................	1	1	7y1
........................................cucccucgcCgcgaucuauugaaaguc..............................................	1	1	7y1
........................................cAcccucgcugcgaucuauugaaaguc..............................................	18	1	7y1
........................................cucccucgcugcgauAuauugaaaguc..............................................	3	1	7y1
........................................cucccucgcugcgaucuaGugaaaguc..............................................	1	1	7y1
........................................cucccucgcugcgaucuauuAaaaguc..............................................	1	1	7y1
........................................cucccAcgcugcgaucuauugaaaguc..............................................	9	1	7y1
........................................cucccucgGugcgaucuauugaaaguc..............................................	1	1	7y1
........................................cucccucgcAgcgaucuauugaaaguc..............................................	3	1	7y1
........................................cucccucgcugcgaucuaAugaaaguc..............................................	7	1	7y1
........................................cucccucgcugcgaucuauugaaaCuc..............................................	1	1	7y1
........................................cucccucgcugcgaucuauuUaaaguc..............................................	2	1	7y1
........................................cucccucgcugcgaucuauugaaaguc..............................................	4185	0	7y1
........................................cucccucgcuAcgaucuauugaaaguc..............................................	4	1	7y1
........................................cucccucgcugcgaucuauugaaaguG..............................................	1	1	7y1
........................................cucccucCcugcgaucuauugaaaguc..............................................	4	1	7y1
........................................Aucccucgcugcgaucuauugaaaguc..............................................	8	1	7y1
........................................cucccucgcugcgCucuauugaaaguc..............................................	1	1	7y1
........................................cucccucgcugcgaucuGuugaaaguc..............................................	1	1	7y1
........................................cucccucgcugcgaucuauugaaaUuc..............................................	1	1	7y1
........................................cucccuAgcugcgaucuauugaaaguc..............................................	6	1	7y1
........................................cuccAucgcugcgaucuauugaaaguc..............................................	2	1	7y1
........................................cucccucgcugcgaucuauuCaaaguca.............................................	1	1	7y1
........................................cucccucgcugcgaucuauAgaaaguca.............................................	3	1	7y1
........................................cucccucgcugcgaucAauugaaaguca.............................................	1	1	7y1
........................................cucccucgcAgcgaucuauugaaaguca.............................................	5	1	7y1
........................................cucccAcgcugcgaucuauugaaaguca.............................................	4	1	7y1
........................................cucccucgcugcgaucuauugaaaguca.............................................	1280	0	7y1
........................................cucccucgcugcgaucuauugaGaguca.............................................	1	1	7y1
........................................cAcccucgcugcgaucuauugaaaguca.............................................	1	1	7y1
........................................cuAccucgcugcgaucuauugaaaguca.............................................	4	1	7y1
........................................cucccuAgcugcgaucuauugaaaguca.............................................	1	1	7y1
........................................cucccucgcugcgaucuauugaaagucG.............................................	2	1	7y1
........................................cucAcucgcugcgaucuauugaaaguca.............................................	1	1	7y1
........................................Aucccucgcugcgaucuauugaaaguca.............................................	3	1	7y1
........................................cucccucgcugcgaucuauugaaaguGa.............................................	1	1	7y1
........................................cucccucgcuCcgaucuauugaaaguca.............................................	1	1	7y1
........................................cucccucgcugAgaucuauugaaaguca.............................................	2	1	7y1
........................................cucccucgcugcgauAuauugaaaguca.............................................	2	1	7y1
........................................cuAccucgcugcgaucuauugaaagucag............................................	2	1	7y1
........................................cucccAcgcugcgaucuauugaaagucag............................................	1	1	7y1
........................................cucccucgcAgcgaucuauugaaagucag............................................	1	1	7y1
........................................Aucccucgcugcgaucuauugaaagucag............................................	1	1	7y1
........................................cucccuAgcugcgaucuauugaaagucag............................................	3	1	7y1
........................................cucAcucgcugcgaucuauugaaagucag............................................	1	1	7y1
........................................cucccucgcugcgaucuauugaaaCucag............................................	1	1	7y1
........................................Gucccucgcugcgaucuauugaaagucag............................................	1	1	7y1
........................................cucccucgcugcgaucuauugaaagGcag............................................	1	1	7y1
........................................cucccucgcugAgaucuauugaaagucag............................................	4	1	7y1
........................................cucccucgcugcgaucuauugaaagucag............................................	378	0	7y1
........................................cucccucgcugcgaucuauAgaaagucag............................................	1	1	7y1
........................................cuccGucgcugcgaucuauugaaagucagcc..........................................	1	1	7y1
........................................cucccucgAugcgaucuauugaaagucagcc..........................................	1	1	7y1
........................................cucccucgcugcgaucuauugaaaguAagcc..........................................	1	1	7y1
........................................Nucccucgcugcgaucuauugaaagucagcc..........................................	1	1	7y1
........................................cuAccucgcugcgaucuauugaaagucagcc..........................................	1	1	7y1
........................................cucccucgcugcgaucuauugaaagAcagcc..........................................	1	1	7y1
```

**Star** **Mature**

| | | | |
|---|---|---|---|
| gaccugcuucugggucggggguuucguacguagcagagcagcucccucgcugcgaucuauugaaagucagcccucgacacaaggguuuguccgcgcgcgcgcgcgcgugcgugc | | | |
| ........................................cucccucgcugcgaucuauugaaagucagcc.......................................... | 172 | 0 | 7y1 |
| .........................................ucccucgcugcUaucuau...................................................... | 1 | 1 | 7y1 |
| .........................................ucccucgcugcgaAcuau...................................................... | 1 | 1 | 7y1 |
| .........................................Acccucgcugcgaucuau...................................................... | 1 | 1 | 7y1 |
| .........................................ucccucgcugcgaCcuau...................................................... | 1 | 1 | 7y1 |
| .........................................ucccucgcugcgaucuau...................................................... | 200 | 0 | 7y1 |
| .........................................Ncccucgcugcgaucuau...................................................... | 1 | 1 | 7y1 |
| .........................................ucccucgcugcgaucuaG...................................................... | 1 | 1 | 7y1 |
| .........................................uAccucgcugcgaucuauu..................................................... | 1 | 1 | 7y1 |
| .........................................ucccucgcugcgaucuauu..................................................... | 189 | 0 | 7y1 |
| .........................................uUccucgcugcgaucuauu..................................................... | 1 | 1 | 7y1 |
| .........................................Acccucgcugcgaucuauu..................................................... | 1 | 1 | 7y1 |
| .........................................ucAcucgcugcgaucuauu..................................................... | 1 | 1 | 7y1 |
| .........................................Gcccucgcugcgaucuauu..................................................... | 1 | 1 | 7y1 |
| .........................................ucccucgcugcUaucuauu..................................................... | 2 | 1 | 7y1 |
| .........................................ucccucgcugcgaAcuauug.................................................... | 1 | 1 | 7y1 |
| .........................................ucccucgcugcUaucuauug.................................................... | 1 | 1 | 7y1 |
| .........................................ucccucgcugcgaucuauug.................................................... | 266 | 0 | 7y1 |
| .........................................Acccucgcugcgaucuauug.................................................... | 4 | 1 | 7y1 |
| .........................................ucccucgcugGgaucuauug.................................................... | 1 | 1 | 7y1 |
| .........................................ucccuAgcugcgaucuauug.................................................... | 1 | 1 | 7y1 |
| .........................................Acccucgcugcgaucuauugaaa................................................. | 2 | 1 | 7y1 |
| .........................................ucccucgcugcgaucuauugaaa................................................. | 212 | 0 | 7y1 |
| .........................................ucccucgcugcgaucuauuUaaa................................................. | 1 | 1 | 7y1 |
| .........................................ucccucUcugcgaucuauugaaa................................................. | 1 | 1 | 7y1 |
| .........................................Gcccucgcugcgaucuauugaaa................................................. | 1 | 1 | 7y1 |
| .........................................Ccccucgcugcgaucuauugaaa................................................. | 1 | 1 | 7y1 |
| .........................................ucccuAgcugcgaucuauugaaag................................................ | 1 | 1 | 7y1 |
| .........................................ucccucgcugcgauAuauugaaag................................................ | 1 | 1 | 7y1 |
| .........................................ucccucgcugcgaucAauugaaag................................................ | 1 | 1 | 7y1 |
| .........................................Acccucgcugcgaucuauugaaag................................................ | 7 | 1 | 7y1 |
| .........................................ucccucgcugcgaucuauAgaaag................................................ | 1 | 1 | 7y1 |
| .........................................ucccucgcugcgauUuauugaaag................................................ | 1 | 1 | 7y1 |
| .........................................ucccucgcugcgaucuauuAaaag................................................ | 1 | 1 | 7y1 |
| .........................................ucccucgcuUcgaucuauugaaag................................................ | 1 | 1 | 7y1 |
| .........................................ucAcucgcugcgaucuauugaaag................................................ | 3 | 1 | 7y1 |
| .........................................Ncccucgcugcgaucuauugaaag................................................ | 3 | 1 | 7y1 |
| .........................................Gcccucgcugcgaucuauugaaag................................................ | 1 | 1 | 7y1 |
| .........................................uAccucgcugcgaucuauugaaag................................................ | 2 | 1 | 7y1 |
| .........................................ucccucgcugcgaucuauugCaag................................................ | 1 | 1 | 7y1 |
| .........................................ucccucgcugcgaucuauugaaag................................................ | 935 | 0 | 7y1 |
| .........................................ucccucgcugcgaucuauugGaag................................................ | 1 | 1 | 7y1 |
| .........................................ucccucgcugAgaucuauugaaag................................................ | 1 | 1 | 7y1 |
| .........................................ucccucUcugcgaucuauugaaag................................................ | 2 | 1 | 7y1 |
| .........................................ucccucgcAgcgaucuauugaaag................................................ | 2 | 1 | 7y1 |
| .........................................ucccucgcuCcgaucuauugaaag................................................ | 3 | 1 | 7y1 |
| .........................................ucccucgcugcgaucuauugaaagu............................................... | 682 | 0 | 7y1 |
| .........................................ucccucgcAgcgaucuauugaaagu............................................... | 2 | 1 | 7y1 |
| .........................................ucccucgcugAgaucuauugaaagu............................................... | 4 | 1 | 7y1 |
| .........................................Acccucgcugcgaucuauugaaagu............................................... | 12 | 1 | 7y1 |
| .........................................ucccucUcugcgaucuauugaaagu............................................... | 1 | 1 | 7y1 |
| .........................................uccAucgcugcgaucuauugaaagu............................................... | 1 | 1 | 7y1 |
| .........................................ucAcucgcugcgaucuauugaaagu............................................... | 1 | 1 | 7y1 |
| .........................................ucccucgcugcgauAuauugaaagu............................................... | 2 | 1 | 7y1 |
| .........................................ucccucgcugcUaucuauugaaagu............................................... | 1 | 1 | 7y1 |
| .........................................ucccucgcugcgaucAauugaaagu............................................... | 1 | 1 | 7y1 |
| .........................................ucccucgcugcgaucuauugaaagA............................................... | 1 | 1 | 7y1 |
| .........................................Gcccucgcugcgaucuauugaaagu............................................... | 1 | 1 | 7y1 |
| .........................................uAccucgcugcgaucuauugaaagu............................................... | 1 | 1 | 7y1 |
| .........................................ucccucgcugcgaucuaAugaaagu............................................... | 1 | 1 | 7y1 |
| .........................................ucccucgcuUcgaucuauugaaagu............................................... | 1 | 1 | 7y1 |
| .........................................Gcccucgcugcgaucuauugaaaguc.............................................. | 2 | 1 | 7y1 |
| .........................................ucccucgcugcgaucuauugaaaAuc.............................................. | 1 | 1 | 7y1 |
| .........................................ucccucgcugcgaucuaAugaaaguc.............................................. | 1 | 1 | 7y1 |
| .........................................ucccucgcuCcgaucuauugaaaguc.............................................. | 2 | 1 | 7y1 |
| .........................................ucccucgcugcgaucuauugaaaCuc.............................................. | 3 | 1 | 7y1 |
| .........................................ucccucgcugcgaucAauugaaaguc.............................................. | 3 | 1 | 7y1 |
| .........................................ucccucgcAgcgaucuauugaaaguc.............................................. | 4 | 1 | 7y1 |
| .........................................ucccucgcugcgaucuauAgaaaguc.............................................. | 2 | 1 | 7y1 |
| .........................................ucAcucgcugcgaucuauugaaaguc.............................................. | 10 | 1 | 7y1 |

**Star** **Mature**

```
gaccugcuucugggucgggguuucguacguagcagagcagcucccucgcugcgaucuauugaaagucagcccucgacacaaggguuuguccgcgcgcgcgcgcgcgugcgugc
.........................................ucccucgcugcgaucuauugGaaguc..............................................  1     1  7y1
.........................................uAccucgcugcgaucuauugaaaguc..............................................  3     1  7y1
.........................................ucccucgcugcUaucuauugaaaguc..............................................  1     1  7y1
.........................................ucccucgAugcgaucuauugaaaguc..............................................  3     1  7y1
.........................................ucccucgcugcgaucuauugaaaguc..............................................  2692  0  7y1
.........................................ucccuAgcugcgaucuauugaaaguc..............................................  7     1  7y1
.........................................ucccucgcugcgaucuauugaaaguA..............................................  1     1  7y1
.........................................ucccucgcGgcgaucuauugaaaguc..............................................  1     1  7y1
.........................................ucccucgcuUcgaucuauugaaaguc..............................................  2     1  7y1
.........................................ucccucAcugcgaucuauugaaaguc..............................................  1     1  7y1
.........................................ucccucgcugcgaucuGuugaaaguc..............................................  2     1  7y1
.........................................Acccucgcugcgaucuauugaaaguc..............................................  38    1  7y1
.........................................ucccucgcugAgaucuauugaaaguc..............................................  2     1  7y1
.........................................ucccAcgcugcgaucuauugaaaguc..............................................  1     1  7y1
.........................................ucccucgcugcCaucuauugaaaguc..............................................  3     1  7y1
.........................................ucccucgcugcgaucuauugaaagAc..............................................  2     1  7y1
.........................................ucccucUcugcgaucuauugaaaguc..............................................  2     1  7y1
.........................................ucccucgcugcgauAuauugaaaguc..............................................  4     1  7y1
.........................................uccAucgcugcgaucuauugaaaguc..............................................  2     1  7y1
.........................................Ncccucgcugcgaucuauugaaaguc..............................................  1     1  7y1
.........................................ucccucgGugcgaucuauugaaaguca.............................................  1     1  7y1
.........................................ucccucgcugcgaucuauugaaaguca.............................................  586   0  7y1
.........................................Ncccucgcugcgaucuauugaaaguca.............................................  2     1  7y1
.........................................ucccucgcugcgaucCauugaaaguca.............................................  1     1  7y1
.........................................ucccucgcugcgauAuauugaaaguca.............................................  1     1  7y1
.........................................Gcccucgcugcgaucuauugaaaguca.............................................  1     1  7y1
.........................................ucccucgcugAgaucuauugaaaguca.............................................  1     1  7y1
.........................................ucccucgcugcgaucuauugaaagucU.............................................  1     1  7y1
.........................................ucccucUcugcgaucuauugaaaguca.............................................  1     1  7y1
.........................................ucccucgcuAcgaucuauugaaaguca.............................................  1     1  7y1
.........................................ucccucgcuCcgaucuauugaaaguca.............................................  1     1  7y1
.........................................uccAucgcugcgaucuauugaaaguca.............................................  1     1  7y1
.........................................ucccucgcugcCaucuauugaaaguca.............................................  1     1  7y1
.........................................ucccucgAugcgaucuauugaaaguca.............................................  2     1  7y1
.........................................ucccucCcugcgaucuauugaaaguca.............................................  1     1  7y1
.........................................Acccucgcugcgaucuauugaaaguca.............................................  6     1  7y1
.........................................ucccucgcuUcgaucuauugaaagucag............................................  1     1  7y1
.........................................ucAcucgcugcgaucuauugaaagucag............................................  1     1  7y1
.........................................ucccucgcugcgaucuauAgaaagucag............................................  1     1  7y1
.........................................ucccucgcugcgaucuauugaaagucag............................................  159   0  7y1
.........................................Acccucgcugcgaucuauugaaagucag............................................  2     1  7y1
.........................................ucccucgcugcgUucuauugaaagucag............................................  1     1  7y1
.........................................ucccAcgcugcgaucuauugaaagucag............................................  1     1  7y1
..........................................cccucgcugcgaucuauu.....................................................  172   0  7y1
..........................................cccucgcugcgaucuaAu.....................................................  1     1  7y1
..........................................Gccucgcugcgaucuauu.....................................................  1     1  7y1
..........................................ccAucgcugcgaucuauu.....................................................  1     1  7y1
..........................................cAcucgcugcgaucuauu.....................................................  1     1  7y1
..........................................cccucgcugcCaucuauu.....................................................  2     1  7y1
..........................................cccucgcAgcgaucuauu.....................................................  1     1  7y1
..........................................cccucgcugcgaucuauuga...................................................  157   0  7y1
..........................................Accucgcugcgaucuauuga...................................................  1     1  7y1
..........................................ccAucgcugcgaucuauuga...................................................  1     1  7y1
..........................................cccucgAugcgaucuauuga...................................................  1     1  7y1
..........................................cAcucgcugcgaucuauuga...................................................  2     1  7y1
..........................................cccucgcAgcgaucuauuga...................................................  1     1  7y1
..........................................cccucgcugcgaucuauugG...................................................  1     1  7y1
..........................................Nccucgcugcgaucuauuga...................................................  1     1  7y1
..........................................cccucgcugcgaucuauuAaa..................................................  1     1  7y1
..........................................cAcucgcugcgaucuauugaaa.................................................  3     1  7y1
..........................................cccucgAugcgaucuauugaaa.................................................  3     1  7y1
..........................................cccucgcugcUaucuauugaaa.................................................  1     1  7y1
..........................................cccucgcugcgaucuaAugaaa.................................................  3     1  7y1
..........................................cccucgcugcgaucuauAgaaa.................................................  1     1  7y1
..........................................cccucgcuUcgaucuauugaaa.................................................  2     1  7y1
..........................................cccucgcugcCaucuauugaaa.................................................  1     1  7y1
..........................................cUcucgcugcgaucuauugaaa.................................................  1     1  7y1
..........................................ccGucgcugcgaucuauugaaa.................................................  1     1  7y1
..........................................cccucgcugcgaucuauugaaa.................................................  1120  0  7y1
..........................................cccucgcugcgaAcuauugaaa.................................................  2     1  7y1
```

**Star** **Mature**

| | | | |
|---|---|---|---|
| gaccugcuucugggucgggguuucguacguagcagagcagcucccucgcugcgaucuauugaaagucagcccucgacacaaggguuuguccgcgcgcgcgcgcgcgugcgugc | | | |
| .........................................Accucgcugcgaucuauugaaa.................................................. | 3 | 1 | 7y1 |
| .........................................cccucgcugAgaucuauugaaa.................................................. | 3 | 1 | 7y1 |
| .........................................cccucgcugcgauUuauugaaa.................................................. | 1 | 1 | 7y1 |
| .........................................cccucgcuCcgaucuauugaaa.................................................. | 2 | 1 | 7y1 |
| .........................................cccucgcugcgaucuauuCaaa.................................................. | 1 | 1 | 7y1 |
| .........................................cccuAgcugcgaucuauugaaa.................................................. | 1 | 1 | 7y1 |
| .........................................cccAcgcugcgaucuauugaaa.................................................. | 1 | 1 | 7y1 |
| .........................................cccucgcugcgauAuauugaaa.................................................. | 1 | 1 | 7y1 |
| .........................................cccucgcugcgaucGauugaaa.................................................. | 1 | 1 | 7y1 |
| .........................................Ncccucgcugcgaucuauugaaa.................................................. | 1 | 1 | 7y1 |
| .........................................cccucgcCgcgaucuauugaaa.................................................. | 1 | 1 | 7y1 |
| .........................................cccucgcAgcgaucuauugaaa.................................................. | 3 | 1 | 7y1 |
| .........................................cccucgcugcgaucuaCugaaag................................................. | 1 | 1 | 7y1 |
| .........................................cccucgcAgcgaucuauugaaag................................................. | 1 | 1 | 7y1 |
| .........................................cccucgcugcgaucuauAgaaag................................................. | 1 | 1 | 7y1 |
| .........................................cccucgcuUcgaucuauugaaag................................................. | 1 | 1 | 7y1 |
| .........................................Gccucgcugcgaucuauugaaag................................................. | 1 | 1 | 7y1 |
| .........................................cccAcgcugcgaucuauugaaag................................................. | 2 | 1 | 7y1 |
| .........................................cAcucgcugcgaucuauugaaag................................................. | 6 | 1 | 7y1 |
| .........................................cccucgcugcCaucuauugaaag................................................. | 3 | 1 | 7y1 |
| .........................................cccucgcugcgauAuauugaaag................................................. | 1 | 1 | 7y1 |
| .........................................cccucgcugAgaucuauugaaag................................................. | 2 | 1 | 7y1 |
| .........................................ccUucgcugcgaucuauugaaag................................................. | 1 | 1 | 7y1 |
| .........................................ccGucgcugcgaucuauugaaag................................................. | 1 | 1 | 7y1 |
| .........................................cccucgcugcgaucuaAugaaag................................................. | 3 | 1 | 7y1 |
| .........................................cccucCcugcgaucuauugaaag................................................. | 1 | 1 | 7y1 |
| .........................................Ncccucgcugcgaucuauugaaag................................................. | 1 | 1 | 7y1 |
| .........................................cccucgcugcgaucuauugaaag................................................. | 1154 | 0 | 7y1 |
| .........................................cNcucgcugcgaucuauugaaag................................................. | 1 | 1 | 7y1 |
| .........................................cccucgcugcgaucuauuAaaag................................................. | 1 | 1 | 7y1 |
| .........................................Accucgcugcgaucuauugaaag................................................. | 1 | 1 | 7y1 |
| .........................................cccucgcugcgaucuauugUaag................................................. | 1 | 1 | 7y1 |
| .........................................cccucgGugcgaucuauugaaag................................................. | 1 | 1 | 7y1 |
| .........................................cccucgcugcgaucuauGgaaag................................................. | 1 | 1 | 7y1 |
| .........................................ccAucgcugcgaucuauugaaag................................................. | 5 | 1 | 7y1 |
| .........................................ccAucgcugcgaucuauugaaagu................................................ | 4 | 1 | 7y1 |
| .........................................Accucgcugcgaucuauugaaagu................................................ | 2 | 1 | 7y1 |
| .........................................cccucgAugcgaucuauugaaagu................................................ | 5 | 1 | 7y1 |
| .........................................cccucgcugcgaucuauuCaaagu................................................ | 1 | 1 | 7y1 |
| .........................................cccucgcugcgaucCauugaaagu................................................ | 1 | 1 | 7y1 |
| .........................................cccucgcugcgauAuauugaaagu................................................ | 2 | 1 | 7y1 |
| .........................................cccucgcugcgaucAauugaaagu................................................ | 1 | 1 | 7y1 |
| .........................................cAcucgcugcgaucuauugaaagu................................................ | 7 | 1 | 7y1 |
| .........................................cccucgcugAgaucuauugaaagu................................................ | 6 | 1 | 7y1 |
| .........................................cccucgcugcCaucuauugaaagu................................................ | 1 | 1 | 7y1 |
| .........................................cccucgcugcgaucuauugaaagu................................................ | 1043 | 0 | 7y1 |
| .........................................cccucUcugcgaucuauugaaagu................................................ | 2 | 1 | 7y1 |
| .........................................cccucgcugcgaucuauugaaagG................................................ | 11 | 1 | 7y1 |
| .........................................cccAcgcugcgaucuauugaaagu................................................ | 1 | 1 | 7y1 |
| .........................................cccucgcuCcgaucuauugaaagu................................................ | 1 | 1 | 7y1 |
| .........................................cccucgcugcgaucuauugaaagA................................................ | 2 | 1 | 7y1 |
| .........................................cccucgcugcgaAcuauugaaagu................................................ | 1 | 1 | 7y1 |
| .........................................cccucgcugcgaucuauAgaaagu................................................ | 1 | 1 | 7y1 |
| .........................................Ncccucgcugcgaucuauugaaaguc............................................... | 3 | 1 | 7y1 |
| .........................................cccucgcugcgaucuauugaaaguA............................................... | 1 | 1 | 7y1 |
| .........................................cccucgcugcUaucuauugaaaguc............................................... | 1 | 1 | 7y1 |
| .........................................cccAcgcugcgaucuauugaaaguc............................................... | 1 | 1 | 7y1 |
| .........................................cccucgcuUcgaucuauugaaaguc............................................... | 1 | 1 | 7y1 |
| .........................................cccucgcugcgCucuauugaaaguc............................................... | 1 | 1 | 7y1 |
| .........................................cccucgcugcgaucuauugaGaguc............................................... | 2 | 1 | 7y1 |
| .........................................cccucgcugcgaucuaAugaaaguc............................................... | 2 | 1 | 7y1 |
| .........................................cccucgcugcgaucuauugaaaUuc............................................... | 1 | 1 | 7y1 |
| .........................................cccuAgcugcgaucuauugaaaguc............................................... | 1 | 1 | 7y1 |
| .........................................cccucgcugcgaucAauugaaaguc............................................... | 3 | 1 | 7y1 |
| .........................................cccucgcugcgaucuauugaaaCuc............................................... | 1 | 1 | 7y1 |
| .........................................cccucgcugAgaucuauugaaaguc............................................... | 6 | 1 | 7y1 |
| .........................................cNcucgcugcgaucuauugaaaguc............................................... | 1 | 1 | 7y1 |
| .........................................cccucgcCgcgaucuauugaaaguc............................................... | 1 | 1 | 7y1 |
| .........................................ccGucgcugcgaucuauugaaaguc............................................... | 1 | 1 | 7y1 |
| .........................................cccucgcugcgauAuauugaaaguc............................................... | 2 | 1 | 7y1 |

**Star** **Mature**

```
gaccugcuucugggucgggguuucguacguagcagagcagcucccucgcugcgaucuauugaaagucagcccucgacacaaggguuuguccgcgcgcgcgcgcgcgcgugcgugc
..........................................cccucgcugcgaAcuauugaaaguc................................................ 2 1 7y1
..........................................cccucgUugcgaucuauugaaaguc................................................ 1 1 7y1
..........................................cccucgcAgcgaucuauugaaaguc................................................ 1 1 7y1
..........................................cAcucgcugcgaucuauugaaaguc................................................ 7 1 7y1
..........................................Accucgcugcgaucuauugaaaguc................................................ 7 1 7y1
..........................................cccucgcuCcgaucuauugaaaguc................................................ 2 1 7y1
..........................................cccucUcugcgaucuauugaaaguc................................................ 1 1 7y1
..........................................cccucgcugcgaucuauugaaaguc................................................ 1855 0 7y1
..........................................cccucgcugcgaucuauugaaagAc................................................ 2 1 7y1
..........................................cccucgcugcgaucuauuUaaaguc................................................ 1 1 7y1
..........................................cccucgAugcgaucuauugaaaguc................................................ 8 1 7y1
..........................................ccAucgcugcgaucuauugaaaguc................................................ 8 1 7y1
..........................................cccucgcugcgaucuauAgaaaguc................................................ 1 1 7y1
..........................................Uccucgcugcgaucuauugaaaguc................................................ 2 1 7y1
..........................................cccucgcugcgaucuauuAaaaguc................................................ 1 1 7y1
..........................................cccucgcugcgaucuauCgaaaguc................................................ 1 1 7y1
..........................................cccucgcugGgaucuauugaaaguc................................................ 1 1 7y1
..........................................cccucgcugcgaucuauugaaaguAa............................................... 1 1 7y1
..........................................cccuAgcugcgaucuauugaaaguca............................................... 3 1 7y1
..........................................cccucgcugAgaucuauugaaaguca............................................... 2 1 7y1
..........................................ccAucgcugcgaucuauugaaaguca............................................... 2 1 7y1
..........................................cccuUgcugcgaucuauugaaaguca............................................... 2 1 7y1
..........................................Nccucgcugcgaucuauugaaaguca............................................... 3 1 7y1
..........................................cccucgcugcgaucuauugaaagAca............................................... 3 1 7y1
..........................................Accucgcugcgaucuauugaaaguca............................................... 5 1 7y1
..........................................cccucgcugcgauAuauugaaaguca............................................... 2 1 7y1
..........................................cccucUcugcgaucuauugaaaguca............................................... 2 1 7y1
..........................................cccucgcugcgaAcuauugaaaguca............................................... 1 1 7y1
..........................................cccucgcugcgaucuauugaaaguca............................................... 592 0 7y1
..........................................cccAcgcugcgaucuauugaaaguca............................................... 1 1 7y1
..........................................cccucAcugcgaucuauugaaaguca............................................... 1 1 7y1
..........................................cccucgcAgcgaucuauugaaaguca............................................... 2 1 7y1
..........................................cccucgcugcgaucuauugaaaUuca............................................... 1 1 7y1
..........................................cccucgcugcUaucuauugaaaguca............................................... 1 1 7y1
...........................................ccucgAugcgaucuauug...................................................... 1 1 7y1
...........................................cAucgcugcgaucuauug...................................................... 2 1 7y1
...........................................ccucgcCgcgaucuauug...................................................... 1 1 7y1
...........................................ccucgcugcgaucuauug...................................................... 209 0 7y1
...........................................ccucgcuUcgaucuauug...................................................... 1 1 7y1
...........................................ccucgcugcgaucuauuC...................................................... 1 1 7y1
...........................................ccucgcGgcgaucuauug...................................................... 1 1 7y1
...........................................Ncucgcugcgaucuauug...................................................... 2 1 7y1
...........................................ccucgcugcgaucGauugaa.................................................... 1 1 7y1
...........................................ccucgcugcUaucuauugaa.................................................... 2 1 7y1
...........................................ccucgcugcgaucuauAgaa.................................................... 1 1 7y1
...........................................ccucgcugcgaucuauugaa.................................................... 602 0 7y1
...........................................Ncucgcugcgaucuauugaa.................................................... 1 1 7y1
...........................................ccuAgcugcgaucuauugaa.................................................... 1 1 7y1
...........................................cAucgcugcgaucuauugaa.................................................... 3 1 7y1
...........................................ccAcgcugcgaucuauugaa.................................................... 1 1 7y1
...........................................ccucgcAgcgaucuauugaa.................................................... 2 1 7y1
...........................................ccucCcugcgaucuauugaa.................................................... 1 1 7y1
...........................................ccucgcugcgaucuauuCaa.................................................... 1 1 7y1
...........................................ccucgcugcgaucuauuAaa.................................................... 1 1 7y1
...........................................Acucgcugcgaucuauugaa.................................................... 1 1 7y1
...........................................ccucgcugcgauAuauugaaa................................................... 4 1 7y1
...........................................Acucgcugcgaucuauugaaa................................................... 6 1 7y1
...........................................ccuAgcugcgaucuauugaaa................................................... 1 1 7y1
...........................................ccucgAugcgaucuauugaaa................................................... 6 1 7y1
...........................................ccucgcugcgaucuaAugaaa................................................... 1 1 7y1
...........................................ccucgcAgcgaucuauugaaa................................................... 9 1 7y1
...........................................ccucgcugcUaucuauugaaa................................................... 5 1 7y1
...........................................ccucgcugAgaucuauugaaa................................................... 1 1 7y1
...........................................cAucgcugcgaucuauugaaa................................................... 2 1 7y1
...........................................Gcucgcugcgaucuauugaaa................................................... 1 1 7y1
...........................................ccucgcugcgaucuauugaGa................................................... 1 1 7y1
...........................................ccucgcuUcgaucuauugaaa................................................... 1 1 7y1
...........................................ccucgcugcgaucAauugaaa................................................... 2 1 7y1
...........................................ccucgcugcgaucuauugaaa................................................... 1604 0 7y1
...........................................ccucgcugcgaucuauugaUa................................................... 1 1 7y1
```

**Star** **Mature**

gaccugcuucugggucgggguuucguacguagcagagcagcucccucgcugcgaucuauugaaagucagcccucgacacaaggguuuguccgcgcgcgcgcgcgcgugcgugc

| | | | |
|---|---|---|---|
| ..........................................ccucgcugcgUucuauugaaa.................................................. | 1 | 1 | 7y1 |
| ..........................................ccucgcugAgaucuauugaaag................................................. | 5 | 1 | 7y1 |
| ..........................................ccucgcugcgaucuauCgaaag................................................. | 1 | 1 | 7y1 |
| ..........................................ccuGgcugcgaucuauugaaag................................................. | 1 | 1 | 7y1 |
| ..........................................ccucgcugcCaucuauugaaag................................................. | 2 | 1 | 7y1 |
| ..........................................Acucgcugcgaucuauugaaag................................................. | 2 | 1 | 7y1 |
| ..........................................ccucgcugcgaAcuauugaaag................................................. | 1 | 1 | 7y1 |
| ..........................................ccucgcugcgaucAauugaaag................................................. | 1 | 1 | 7y1 |
| ..........................................ccucgcuCcgaucuauugaaag................................................. | 1 | 1 | 7y1 |
| ..........................................cAucgcugcgaucuauugaaag................................................. | 4 | 1 | 7y1 |
| ..........................................ccucgcugcgaucuauugaaag................................................. | 1408 | 0 | 7y1 |
| ..........................................ccucgcugcgaucuauugaaaU................................................. | 1 | 1 | 7y1 |
| ..........................................ccucgcugcgaucuauugaaGg................................................. | 1 | 1 | 7y1 |
| ..........................................ccAcgcugcgaucuauugaaag................................................. | 2 | 1 | 7y1 |
| ..........................................ccuAgcugcgaucuauugaaag................................................. | 1 | 1 | 7y1 |
| ..........................................ccucUcugcgaucuauugaaag................................................. | 1 | 1 | 7y1 |
| ..........................................ccucgAugcgaucuauugaaag................................................. | 2 | 1 | 7y1 |
| ..........................................ccucgcugcgaucCauugaaag................................................. | 1 | 1 | 7y1 |
| ..........................................ccucgcugcgauAuauugaaag................................................. | 2 | 1 | 7y1 |
| ..........................................ccucgcugcgaucuaAugaaag................................................. | 1 | 1 | 7y1 |
| ..........................................ccucCcugcgaucuauugaaag................................................. | 2 | 1 | 7y1 |
| ..........................................ccucgcAgcgaucuauugaaag................................................. | 3 | 1 | 7y1 |
| ..........................................Ncucgcugcgaucuauugaaag................................................. | 1 | 1 | 7y1 |
| ..........................................Gcucgcugcgaucuauugaaag................................................. | 2 | 1 | 7y1 |
| ..........................................ccucgcuUcgaucuauugaaag................................................. | 1 | 1 | 7y1 |
| ..........................................ccucgcugcgaucuauAgaaag................................................. | 2 | 1 | 7y1 |
| ..........................................ccucgcuAcgaucuauugaaag................................................. | 1 | 1 | 7y1 |
| ..........................................ccucUcugcgaucuauugaaagu................................................ | 1 | 1 | 7y1 |
| ..........................................ccucgcugcgaucuauugaaagu................................................ | 1291 | 0 | 7y1 |
| ..........................................ccucgcugcgaucuauAgaaagu................................................ | 1 | 1 | 7y1 |
| ..........................................ccuAgcugcgaucuauugaaagu................................................ | 1 | 1 | 7y1 |
| ..........................................Gcucgcugcgaucuauugaaagu................................................ | 1 | 1 | 7y1 |
| ..........................................ccucgcugcgaucuauugaaaUu................................................ | 1 | 1 | 7y1 |
| ..........................................ccucgcuUcgaucuauugaaagu................................................ | 1 | 1 | 7y1 |
| ..........................................ccucgcugcgaucuauugaaagG................................................ | 1 | 1 | 7y1 |
| ..........................................ccucgcugcgaucuaAugaaagu................................................ | 1 | 1 | 7y1 |
| ..........................................ccucgcugcgaucAauugaaagu................................................ | 1 | 1 | 7y1 |
| ..........................................cUucgcugcgaucuauugaaagu................................................ | 1 | 1 | 7y1 |
| ..........................................ccucgcugcUaucuauugaaagu................................................ | 2 | 1 | 7y1 |
| ..........................................ccucgcCgcgaucuauugaaagu................................................ | 1 | 1 | 7y1 |
| ..........................................ccAcgcugcgaucuauugaaagu................................................ | 3 | 1 | 7y1 |
| ..........................................ccucgAugcgaucuauugaaagu................................................ | 2 | 1 | 7y1 |
| ..........................................ccucgcugcgaAcuauugaaagu................................................ | 1 | 1 | 7y1 |
| ..........................................ccucgcugAgaucuauugaaagu................................................ | 6 | 1 | 7y1 |
| ..........................................ccucgcuCcgaucuauugaaagu................................................ | 1 | 1 | 7y1 |
| ..........................................Ncucgcugcgaucuauugaaagu................................................ | 1 | 1 | 7y1 |
| ..........................................ccucgcugcCaucuauugaaagu................................................ | 1 | 1 | 7y1 |
| ..........................................ccucgcugcgauAuauugaaagu................................................ | 1 | 1 | 7y1 |
| ..........................................ccucgcugcgaucuauugaGagu................................................ | 1 | 1 | 7y1 |
| ..........................................Acucgcugcgaucuauugaaagu................................................ | 2 | 1 | 7y1 |
| ..........................................ccucgcAgcgaucuauugaaagu................................................ | 1 | 1 | 7y1 |
| ..........................................cAucgcugcgaucuauugaaagu................................................ | 2 | 1 | 7y1 |
| ..........................................ccucAcugcgaucuauugaaagu................................................ | 2 | 1 | 7y1 |
| ..........................................ccucgcugcgaucuauugaaagA................................................ | 3 | 1 | 7y1 |
| ..........................................ccucgcugcgauGuauugaaagu................................................ | 1 | 1 | 7y1 |
| ..........................................ccuGgcugcgaucuauugaaagu................................................ | 1 | 1 | 7y1 |
| ..........................................ccucgcugcgaucuauugaaagAc............................................... | 4 | 1 | 7y1 |
| ..........................................ccGcgcugcgaucuauugaaaguc............................................... | 1 | 1 | 7y1 |
| ..........................................ccucgcugcgaucuUuugaaaguc............................................... | 1 | 1 | 7y1 |
| ..........................................ccuAgcugcgaucuauugaaaguc............................................... | 4 | 1 | 7y1 |
| ..........................................ccucgcugcgaucuauAgaaaguc............................................... | 4 | 1 | 7y1 |
| ..........................................ccucgcugcgaucuauuCaaaguc............................................... | 1 | 1 | 7y1 |
| ..........................................ccucgcugAgaucuauugaaaguc............................................... | 9 | 1 | 7y1 |
| ..........................................ccucgcugcUaucuauugaaaguc............................................... | 2 | 1 | 7y1 |
| ..........................................ccucgcugcgaGcuauugaaaguc............................................... | 1 | 1 | 7y1 |
| ..........................................ccucgcugcgaucuauCgaaaguc............................................... | 3 | 1 | 7y1 |
| ..........................................ccucCcugcgaucuauugaaaguc............................................... | 2 | 1 | 7y1 |
| ..........................................ccucgcugcgaucuauugaaaguc............................................... | 4155 | 0 | 7y1 |
| ..........................................ccucgcuUcgaucuauugaaaguc............................................... | 5 | 1 | 7y1 |
| ..........................................ccucgNugcgaucuauugaaaguc............................................... | 1 | 1 | 7y1 |

```
  Star                                    Mature
gaccugcuucugggucggggguucguacguagcagagcagcucccucgcugcgaucuauugaaagucagcccucgacacaaggguuuguccgcgcgcgcgcgcgcgugcgugc
...........................................ccucgcugcgaucuaAugaaaguc..............................................   3     1    7y1
...........................................ccAcgcugcgaucuauugaaaguc..............................................   2     1    7y1
...........................................ccucgcugcgaucuauuUaaaguc..............................................   1     1    7y1
...........................................ccucgcugcgaucCauugaaaguc..............................................   1     1    7y1
...........................................ccucgcugcgauAuauugaaaguc..............................................   7     1    7y1
...........................................ccucgcugcgUucuauugaaaguc..............................................   1     1    7y1
...........................................ccucgcugcgaucuauugaGaguc..............................................   1     1    7y1
...........................................ccucgcugcgaAcuauugaaaguc..............................................   1     1    7y1
...........................................Gcucgcugcgaucuauugaaaguc..............................................   2     1    7y1
...........................................ccucgcuCcgaucuauugaaaguc..............................................   1     1    7y1
...........................................ccCcgcugcgaucuauugaaaguc..............................................   1     1    7y1
...........................................Acucgcugcgaucuauugaaaguc..............................................   7     1    7y1
...........................................ccucgcugcCaucuauugaaaguc..............................................   4     1    7y1
...........................................ccucgcugcgaucuauugaaaCuc..............................................   5     1    7y1
...........................................ccucgcugcgaucuauGgaaaguc..............................................   1     1    7y1
...........................................ccucgcugcgaucuauugaaaUuc..............................................   1     1    7y1
...........................................ccucgcugcgaucuauugGaaguc..............................................   1     1    7y1
...........................................ccucgcAgcgaucuauugaaaguc..............................................   16    1    7y1
...........................................ccucgcugcgaucAauugaaaguc..............................................   3     1    7y1
...........................................ccucgcCgcgaucuauugaaaguc..............................................   1     1    7y1
...........................................ccucgAugcgaucuauugaaaguc..............................................   6     1    7y1
...........................................cAucgcugcgaucuauugaaaguc..............................................   9     1    7y1
...........................................ccucgcugcgaucuauugaaGguc..............................................   1     1    7y1
...........................................ccucgcugcgGucuauugaaaguc..............................................   1     1    7y1
...........................................ccucUcugcgaucuauugaaaguc..............................................   3     1    7y1
...........................................Ncucgcugcgaucuauugaaaguc..............................................   1     1    7y1
...........................................ccucgcugcgaucuauugaaaguA..............................................   2     1    7y1
...........................................ccucgcugcgaucuauuAaaaguc..............................................   1     1    7y1
...........................................cAucgcugcgaucuauugaaaguca.............................................   2     1    7y1
...........................................Ncucgcugcgaucuauugaaaguca.............................................   1     1    7y1
...........................................ccucgcAgcgaucuauugaaaguca.............................................   1     1    7y1
...........................................ccucgcugAgaucuauugaaaguca.............................................   2     1    7y1
...........................................Acucgcugcgaucuauugaaaguca.............................................   3     1    7y1
...........................................Gcucgcugcgaucuauugaaaguca.............................................   1     1    7y1
...........................................ccucgcugcgaucuauugaaagAca.............................................   1     1    7y1
...........................................ccucgcugcgaucuauugaaaCuca.............................................   1     1    7y1
...........................................ccucgcugcgaucuauugaaaAuca.............................................   1     1    7y1
...........................................ccucgcugcgaucuauugaaaguca.............................................   761   0    7y1
...........................................ccucgcuUcgaucuauugaaaguca.............................................   1     1    7y1
...........................................ccucUcugcgaucuauugaaaguca.............................................   1     1    7y1
...........................................ccucgAugcgaucuauugaaaguca.............................................   1     1    7y1
...........................................ccucgcugcgauAuauugaaaguca.............................................   1     1    7y1
...........................................ccucgcugcgaucuaAugaaaguca.............................................   1     1    7y1
...........................................ccucgcugcgaucuauugaaaUuca.............................................   1     1    7y1
...........................................ccucgcugcgaucuauugaaagucG.............................................   1     1    7y1
...........................................Acucgcugcgaucuauugaaagucag............................................   1     1    7y1
...........................................ccucgcuCcgaucuauugaaagucag............................................   1     1    7y1
...........................................cAucgcugcgaucuauugaaagucag............................................   1     1    7y1
...........................................ccucgcugcgaucuauAgaaagucag............................................   1     1    7y1
...........................................ccucgcugcUaucuauugaaagucag............................................   1     1    7y1
...........................................Ncucgcugcgaucuauugaaagucag............................................   1     1    7y1
...........................................ccucgcugcgaucAauugaaagucag............................................   1     1    7y1
...........................................ccucgcAgcgaucuauugaaagucag............................................   2     1    7y1
...........................................ccucAcugcgaucuauugaaagucag............................................   1     1    7y1
...........................................ccucgcugAgaucuauugaaagucag............................................   1     1    7y1
...........................................ccucgcugcgaucuauugaaagucag............................................   163   0    7y1
...........................................ccucgcugcgaucuauugaaagucagUcc.........................................   1     1    7y1
...........................................ccucgcugcgaucuauugaaagucagUccu........................................   253   1    7y1
............................................cucgAugcgaucuauugaaa.................................................   1     1    7y1
............................................cucgcugcgaucuauAgaaa.................................................   2     1    7y1
............................................cucgcugcgauAuauugaaa.................................................   1     1    7y1
............................................cucgcugcgaucuauugaaa.................................................   413   0    7y1
............................................cucUcugcgaucuauugaaa.................................................   2     1    7y1
............................................cucgcugAgaucuauugaaa.................................................   2     1    7y1
............................................cucgcugcUaucuauugaaa.................................................   1     1    7y1
............................................Aucgcugcgaucuauugaaa.................................................   2     1    7y1
............................................cuAgcugcgaucuauugaaa.................................................   3     1    7y1
............................................cucgcugcCaucuauugaaa.................................................   1     1    7y1
............................................cAcgcugcgaucuauugaaa.................................................   2     1    7y1
............................................cuUgcugcgaucuauugaaag................................................   1     1    7y1
```

## Star

## Mature

| | | | |
|---|---|---|---|
| gaccugcuucugggucgggguuucguacguagcagagcagcucccucgcugcgaucuauugaaagucagcccucgacacaaggguuuguccgcgcgcgcgcgcgcgugcgugc | | | |
| ............................................cucgcugcUaucuauugaaag................................................ | 2 | 1 | 7y1 |
| ............................................cucgcugcgaucuauugUaag................................................ | 1 | 1 | 7y1 |
| ............................................cAcgcugcgaucuauugaaag................................................ | 12 | 1 | 7y1 |
| ............................................cucAcugcgaucuauugaaag................................................ | 2 | 1 | 7y1 |
| ............................................Gucgcugcgaucuauugaaag................................................ | 4 | 1 | 7y1 |
| ............................................cucgcugcgaucuauuUaaag................................................ | 1 | 1 | 7y1 |
| ............................................cucgcAgcgaucuauugaaag................................................ | 12 | 1 | 7y1 |
| ............................................cucgAugcgaucuauugaaag................................................ | 3 | 1 | 7y1 |
| ............................................cuAgcugcgaucuauugaaag................................................ | 10 | 1 | 7y1 |
| ............................................cucgcugcgaucuauAgaaag................................................ | 2 | 1 | 7y1 |
| ............................................cucgcugcgaucAauugaaag................................................ | 1 | 1 | 7y1 |
| ............................................cucgcugcgaucuauugaaaC................................................ | 2 | 1 | 7y1 |
| ............................................cucgcugcgaucuauugaaag................................................ | 2182 | 0 | 7y1 |
| ............................................cucgcugcgaucuauugGaag................................................ | 1 | 1 | 7y1 |
| ............................................cucCcugcgaucuauugaaag................................................ | 1 | 1 | 7y1 |
| ............................................cucgcugcgaucuaAugaaag................................................ | 1 | 1 | 7y1 |
| ............................................cucgcugcgauUuauugaaag................................................ | 1 | 1 | 7y1 |
| ............................................cucgcugcgaucuauugaGag................................................ | 167 | 1 | 7y1 |
| ............................................cucgcugAgaucuauugaaag................................................ | 8 | 1 | 7y1 |
| ............................................cucgcugcgaucuauugaaaU................................................ | 2 | 1 | 7y1 |
| ............................................cucgcugcgaAcuauugaaag................................................ | 2 | 1 | 7y1 |
| ............................................Aucgcugcgaucuauugaaag................................................ | 2 | 1 | 7y1 |
| ............................................cucgcugcgauAuauugaaag................................................ | 6 | 1 | 7y1 |
| ............................................cucgcuUcgaucuauugaaag................................................ | 2 | 1 | 7y1 |
| ............................................cucgcugcgaucuauGgaaag................................................ | 1 | 1 | 7y1 |
| ............................................cucgcugGgaucuauugaaag................................................ | 2 | 1 | 7y1 |
| ............................................cucgcuCcgaucuauugaaagu............................................... | 1 | 1 | 7y1 |
| ............................................cucgcCgcgaucuauugaaagu............................................... | 1 | 1 | 7y1 |
| ............................................cucgcugcCaucuauugaaagu............................................... | 1 | 1 | 7y1 |
| ............................................cucgcugcgauAuauugaaagu............................................... | 1 | 1 | 7y1 |
| ............................................cAcgcugcgaucuauugaaagu............................................... | 7 | 1 | 7y1 |
| ............................................cucgcugcgaucuauugaaagu............................................... | 1949 | 0 | 7y1 |
| ............................................cucUcugcgaucuauugaaagu............................................... | 1 | 1 | 7y1 |
| ............................................cucgcuUcgaucuauugaaagu............................................... | 1 | 1 | 7y1 |
| ............................................cucgcNgcgaucuauugaaagu............................................... | 1 | 1 | 7y1 |
| ............................................cucgcugcgaucuUugaaagu............................................... | 1 | 1 | 7y1 |
| ............................................cucgcugcUaucuauugaaagu............................................... | 2 | 1 | 7y1 |
| ............................................cucgUugcgaucuauugaaagu............................................... | 1 | 1 | 7y1 |
| ............................................cucgcugcgaucuauugaaagG............................................... | 1 | 1 | 7y1 |
| ............................................cucgcugcgaucCauugaaagu............................................... | 1 | 1 | 7y1 |
| ............................................cucgcugcgaucuauugaaaUu............................................... | 1 | 1 | 7y1 |
| ............................................cuAgcugcgaucuauugaaagu............................................... | 4 | 1 | 7y1 |
| ............................................cucgcAgcgaucuauugaaagu............................................... | 4 | 1 | 7y1 |
| ............................................cucgcugcgaucAauugaaagu............................................... | 1 | 1 | 7y1 |
| ............................................cucgcugcgaucuauuUaaagu............................................... | 1 | 1 | 7y1 |
| ............................................cucgcugcgaAcuauugaaagu............................................... | 3 | 1 | 7y1 |
| ............................................Nucgcugcgaucuauugaaagu............................................... | 1 | 1 | 7y1 |
| ............................................cucgcugcgaucuauAgaaagu............................................... | 2 | 1 | 7y1 |
| ............................................cucgcugcgaucuauuCaaagu............................................... | 1 | 1 | 7y1 |
| ............................................cucgcugAgaucuauugaaagu............................................... | 3 | 1 | 7y1 |
| ............................................Aucgcugcgaucuauugaaagu............................................... | 6 | 1 | 7y1 |
| ............................................cucgcugcgauGuauugaaagu............................................... | 1 | 1 | 7y1 |
| ............................................cNcgcugcgaucuauugaaagu............................................... | 1 | 1 | 7y1 |
| ............................................cucgGugcgaucuauugaaaguc.............................................. | 1 | 1 | 7y1 |
| ............................................cucgcuAcgaucuauugaaaguc.............................................. | 1 | 1 | 7y1 |
| ............................................cAcgcugcgaucuauugaaaguc.............................................. | 7 | 1 | 7y1 |
| ............................................cucgcugcgaucuauuUaaaguc.............................................. | 1 | 1 | 7y1 |
| ............................................cucgcugcgaucuauAgaaaguc.............................................. | 1 | 1 | 7y1 |
| ............................................Nucgcugcgaucuauugaaaguc.............................................. | 1 | 1 | 7y1 |
| ............................................Gucgcugcgaucuauugaaaguc.............................................. | 1 | 1 | 7y1 |
| ............................................cucgcugcgaucuaAugaaaguc.............................................. | 5 | 1 | 7y1 |
| ............................................cucgcuUcgaucuauugaaaguc.............................................. | 1 | 1 | 7y1 |
| ............................................cucgcugcgaucuauugaaaCuc.............................................. | 1 | 1 | 7y1 |
| ............................................cucCcugcgaucuauugaaaguc.............................................. | 2 | 1 | 7y1 |
| ............................................cuAgcugcgaucuauugaaaguc.............................................. | 5 | 1 | 7y1 |
| ............................................cucgcugcgaCcuauugaaaguc.............................................. | 1 | 1 | 7y1 |
| ............................................cucgcugcUaucuauugaaaguc.............................................. | 1 | 1 | 7y1 |
| ............................................cucgAugcgaucuauugaaaguc.............................................. | 3 | 1 | 7y1 |
| ............................................cucgcugcgaucuaCugaaaguc.............................................. | 1 | 1 | 7y1 |
| ............................................cucgcugcgaGcuauugaaaguc.............................................. | 1 | 1 | 7y1 |

Star                                                Mature

```
gaccugcuucugggucgggguuucguacguagcagagcagcucccucgcugcgaucuauugaaagucagcccucgacacaaggguuuguccgcgcgcgcgcgcgcgugcgugc
............................................cucgcugcgaucuauugaaaguc..............................................    2329    0    7y1
............................................cucgcugcgGucuauugaaaguc..............................................    1       1    7y1
............................................cucUcugcgaucuauugaaaguc..............................................    1       1    7y1
............................................cuGgcugcgaucuauugaaaguc..............................................    2       1    7y1
............................................cucgcugcgaucuauuCaaaguc..............................................    4       1    7y1
............................................cucgcAgcgaucuauugaaaguc..............................................    12      1    7y1
............................................cucgcugAgaucuauugaaaguc..............................................    8       1    7y1
............................................cucgcuCcgaucuauugaaaguc..............................................    2       1    7y1
............................................cucgcugcgauAuauugaaaguc..............................................    4       1    7y1
............................................Aucgcugcgaucuauugaaaguc..............................................    10      1    7y1
............................................cucgcugcgaucuauugaaaguA..............................................    2       1    7y1
............................................cucgcugcgaucuauugaaaguca.............................................    238     0    7y1
............................................cucgcugcgaAcuauugaaaguca.............................................    1       1    7y1
............................................cucgcugAgaucuauugaaaguca.............................................    1       1    7y1
............................................Aucgcugcgaucuauugaaaguca.............................................    1       1    7y1
............................................cucgcugcgaucuauugaaaguAa.............................................    3       1    7y1
............................................cuAgcugcgaucuauugaaaguca.............................................    1       1    7y1
.............................................ucgcAgcgaucuauugaa..................................................    1       1    7y1
.............................................ucgcugAgaucuauugaa..................................................    1       1    7y1
.............................................Acgcugcgaucuauugaa..................................................    3       1    7y1
.............................................ucgcugcgaucuauAgaa..................................................    1       1    7y1
.............................................ucgcugcgaucuauugaa..................................................    152     0    7y1
.............................................ucgcugcgaucuauugaaG.................................................    2       1    7y1
.............................................ucgcugcgaucuaCugaaag................................................    1       1    7y1
.............................................ucgcugcgauGuauugaaag................................................    1       1    7y1
.............................................Ncgcugcgaucuauugaaag................................................    2       1    7y1
.............................................ucgcAgcgaucuauugaaag................................................    3       1    7y1
.............................................ucgcugcgNucuauugaaag................................................    1       1    7y1
.............................................ucgcugcgaucuUuugaaag................................................    1       1    7y1
.............................................ucgcugcgaucuauAgaaag................................................    4       1    7y1
.............................................Gcgcugcgaucuauugaaag................................................    1       1    7y1
.............................................ucgcugAgaucuauugaaag................................................    17      1    7y1
.............................................ucgcugcgaucuauugaaaC................................................    3       1    7y1
.............................................ucgcugcgaucuauugaaUg................................................    1       1    7y1
.............................................ucgcCgcgaucuauugaaag................................................    2       1    7y1
.............................................ucgcuCcgaucuauugaaag................................................    1       1    7y1
.............................................ucgcugcgaucuauuCaaag................................................    2       1    7y1
.............................................ucgcugcgaucAauugaaag................................................    6       1    7y1
.............................................ucgAugcgaucuauugaaag................................................    4       1    7y1
.............................................ucgcugcgCucuauugaaag................................................    1       1    7y1
.............................................ucgcugcgaucGauugaaag................................................    1       1    7y1
.............................................ucgcugcgaucuaAugaaag................................................    3       1    7y1
.............................................ucgcugcgaucuauugaaGg................................................    3       1    7y1
.............................................ucgcugcgaucuauugaaag................................................    3906    0    7y1
.............................................ucgcuUcgaucuauugaaag................................................    5       1    7y1
.............................................ucgcugcgaucuauugaaCg................................................    1       1    7y1
.............................................uAgcugcgaucuauugaaag................................................    12      1    7y1
.............................................ucgcugcCaucuauugaaag................................................    1       1    7y1
.............................................ucgcugcgaucuauuUaaag................................................    2       1    7y1
.............................................ucgGugcgaucuauugaaag................................................    2       1    7y1
.............................................ucgcugGgaucuauugaaag................................................    2       1    7y1
.............................................ucgcugcgauNuauugaaag................................................    1       1    7y1
.............................................ucUcugcgaucuauugaaag................................................    1       1    7y1
.............................................ucgcugcgauAuauugaaag................................................    4       1    7y1
.............................................uGgcugcgaucuauugaaag................................................    2       1    7y1
.............................................ucgcugcgaAcuauugaaag................................................    11      1    7y1
.............................................Acgcugcgaucuauugaaag................................................    60      1    7y1
.............................................ucgcuAcgaucuauugaaag................................................    2       1    7y1
.............................................ucgcGgcgaucuauugaaag................................................    1       1    7y1
.............................................ucgcugcUaucuauugaaag................................................    2       1    7y1
.............................................ucgcugcgaucuGuugaaag................................................    1       1    7y1
.............................................Acgcugcgaucuauugaaagu...............................................    4       1    7y1
.............................................ucgcugcCaucuauugaaagu...............................................    1       1    7y1
.............................................ucgcugcgaAcuauugaaagu...............................................    1       1    7y1
.............................................ucgcugcgaucuauugaaagu...............................................    138     0    7y1
.............................................ucgcugcgaucAauugaaagu...............................................    1       1    7y1
.............................................ucgcugcgaucuauugaaagucagcccucgaG....................................    1       1    7y1
.............................................ucgcugcgaucuauugaaagucagcUcucgac....................................    1       1    7y1
.............................................ucgcugcgaucuauugaaagucagccAucgac....................................    1       1    7y1
.............................................Ncgcugcgaucuauugaaagucagcccucgac....................................    1       1    7y1
```

**Star** **Mature**

```
gaccugcuucugggucgggguuucguacguagcagagcagcucccucgcugcgaucuauugaaagucagcccucgacacaaggguuuguccgcgcgcgcgcgcgcgugcgugc
.............................................ucgcugcgaucuaGugaaagucagcccucgac....................................    1      1    7y1
.............................................ucgcugcgaucuaAugaaagucagcccucgac....................................    1      1    7y1
.............................................ucgcugcgaucuauugaaagucagcccucgac....................................    506    0    7y1
.............................................ucgcugcgaucuauuCaaagucagcccucgac....................................    1      1    7y1
.............................................ucgcugcgaucuauugaaagucagcccucgaA....................................    1      1    7y1
.............................................ucgcugcUaucuauugaaagucagcccucgac....................................    1      1    7y1
.............................................Acgcugcgaucuauugaaagucagcccucgac....................................    7      1    7y1
..............................................cgcugcgaucuaAugaaa.................................................    1      1    7y1
..............................................cUcugcgaucuauugaaa.................................................    1      1    7y1
..............................................cgcugAgaucuauugaaa.................................................    1      1    7y1
..............................................cCcugcgaucuauugaaa.................................................    2      1    7y1
..............................................cgcugcgaucuauugaaa.................................................    357    0    7y1
..............................................cgcuAcgaucuauugaaa.................................................    1      1    7y1
..............................................cgcugcgaAcuauugaaa.................................................    1      1    7y1
..............................................cgcugcUaucuauugaaa.................................................    1      1    7y1
..............................................cgcugcgaucuauugaaG.................................................    1      1    7y1
..............................................cgcugcgaGcuauugaaag................................................    2      1    7y1
..............................................cgcugcgaucuUuugaaag................................................    1      1    7y1
..............................................cgcugcgaucuauuCaaag................................................    1      1    7y1
..............................................cgAugcgaucuauugaaag................................................    20     1    7y1
..............................................cgcugcgaucuauugaUag................................................    4      1    7y1
..............................................cgcugcgaucuauCgaaag................................................    1      1    7y1
..............................................cgcugcgaucuauugaaaC................................................    1      1    7y1
..............................................cgcGgcgaucuauugaaag................................................    2      1    7y1
..............................................cgcugcgaucuauuUaaag................................................    150    1    7y1
..............................................Agcugcgaucuauugaaag................................................    16     1    7y1
..............................................cgGugcgaucuauugaaag................................................    3      1    7y1
..............................................cgcugcgaucuauAgaaag................................................    9      1    7y1
..............................................Ugcugcgaucuauugaaag................................................    2      1    7y1
..............................................cgUugcgaucuauugaaag................................................    1      1    7y1
..............................................cgcugAgaucuauugaaag................................................    29     1    7y1
..............................................cgcugcgauGuauugaaag................................................    2      1    7y1
..............................................cCcugcgaucuauugaaag................................................    4      1    7y1
..............................................cgcugcgUucuauugaaag................................................    2      1    7y1
..............................................cgcCgcgaucuauugaaag................................................    1      1    7y1
..............................................cgcugcgaucuauugaGag................................................    2      1    7y1
..............................................cgcugcgaucuauugaaag................................................    6574   0    7y1
..............................................cgcugcgaucAauugaaag................................................    4      1    7y1
..............................................Ngcugcgaucuauugaaag................................................    1      1    7y1
..............................................cgcugGgaucuauugaaag................................................    2      1    7y1
..............................................cUcugcgaucuauugaaag................................................    8      1    7y1
..............................................cgcugcCaucuauugaaag................................................    4      1    7y1
..............................................cgcuCcgaucuauugaaag................................................    2      1    7y1
..............................................cgcugcgaucGauugaaag................................................    1      1    7y1
..............................................cgcAgcgaucuauugaaag................................................    11     1    7y1
..............................................cgcugcgauAuauugaaag................................................    14     1    7y1
..............................................cgcugcgaucuauugaaaU................................................    4      1    7y1
..............................................cgcugcgaucuauugUaag................................................    2      1    7y1
..............................................cAcugcgaucuauugaaag................................................    1      1    7y1
..............................................cgcuUcgaucuauugaaag................................................    7      1    7y1
..............................................cgcugcgaAcuauugaaag................................................    9      1    7y1
..............................................cgcugcgaucuauugaaUg................................................    1      1    7y1
..............................................Ggcugcgaucuauugaaag................................................    4      1    7y1
..............................................cgcugcgaucuaAugaaag................................................    7      1    7y1
..............................................cgcuAcgaucuauugaaag................................................    1      1    7y1
..............................................cgcugcUaucuauugaaag................................................    10     1    7y1
..............................................cgcugcgaucuauugaaaA................................................    1      1    7y1
..............................................cUcugcgaucuauugaaagu...............................................    3      1    7y1
..............................................cgcugcgaucuauugaaaCu...............................................    1      1    7y1
..............................................cgcugcgaucuauugaaagu...............................................    160    0    7y1
..............................................Ggcugcgaucuauugaaagu...............................................    1      1    7y1
..............................................cgcugcgaucuauAgaaagu...............................................    1      1    7y1
..............................................cgcugAgaucuauugaaagu...............................................    2      1    7y1
..............................................cgcugcgaucuaAugaaagu...............................................    1      1    7y1
..............................................cgcugcgaucuauugaaagAc..............................................    1      1    7y1
..............................................cgcugcgaucuauAgaaaguc..............................................    2      1    7y1
..............................................cgcugcgauAuauugaaaguc..............................................    2      1    7y1
..............................................cUcugcgaucuauugaaaguc..............................................    4      1    7y1
..............................................cgcugcgaAcuauugaaaguc..............................................    1      1    7y1
..............................................Agcugcgaucuauugaaaguc..............................................    2      1    7y1
```

```
Star                                                                 Mature
gaccugcuucugggucgggguuucguacguagcagagcagcucccucgcugcgaucuauugaaagucagcccucgacacaaggguuuguccgcgcgcgcgcgcgcgugcgugc
..............................................cgcugcgaucuauugGaaguc..............................................    1     1    7y1
..............................................cgcugcgaucuauugaGaguc..............................................    1     1    7y1
..............................................cgcugcgaucuUuugaaaguc..............................................    1     1    7y1
..............................................cgcugAgaucuauugaaaguc..............................................    2     1    7y1
..............................................cgcugcgauUuauugaaaguc..............................................    1     1    7y1
..............................................cgcugcgaucuauugaaaguA..............................................    1     1    7y1
..............................................cgcugcgaucuauugaaaguc..............................................    553   0    7y1
..............................................cgcAgcgaucuauugaaaguc..............................................    1     1    7y1
..............................................cgcugcgaucuauugaaGgucag............................................    2     1    7y1
..............................................cgcugcgaucuauugaaagucag............................................    184   0    7y1
..............................................cgcugcgaucuauugaaagucaU............................................    1     1    7y1
..............................................cgcugcgaucuauAgaaagucag............................................    1     1    7y1
..............................................cgcugcgaucuaAugaaagucag............................................    1     1    7y1
..............................................cgcugAgaucuauugaaagucag............................................    1     1    7y1
..............................................cUcugcgaucuauugaaagucag............................................    1     1    7y1
..............................................Agcugcgaucuauugaaagucagcccucgac....................................    1     1    7y1
..............................................cgAugcgaucuauugaaagucagcccucgac....................................    1     1    7y1
..............................................cgcugAgaucuauugaaagucagcccucgac....................................    1     1    7y1
..............................................cgcugcgaucuauugaaagucagcccucgCc....................................    1     1    7y1
..............................................cgcugcgaucuauugaaagucagcccucgac....................................    162   0    7y1
...............................................gcuUcgaucuauugaaag................................................    4     1    7y1
...............................................gcugcgaucAauugaaag................................................    7     1    7y1
...............................................Ucugcgaucuauugaaag................................................    12    1    7y1
...............................................gcGgcgaucuauugaaag................................................    2     1    7y1
...............................................gcugcgaucuauuUaaag................................................    1     1    7y1
...............................................gcugcUaucuauugaaag................................................    5     1    7y1
...............................................gcugcgauAuauugaaag................................................    3     1    7y1
...............................................gcugcgaucuauugaaaC................................................    2     1    7y1
...............................................gcugcgaucuaAugaaag................................................    4     1    7y1
...............................................gcugcCaucuauugaaag................................................    2     1    7y1
...............................................gAugcgaucuauugaaag................................................    6     1    7y1
...............................................gcugcgaucuauugaaag................................................    2699  0    7y1
...............................................gcuCcgaucuauugaaag................................................    1     1    7y1
...............................................gcugcgaucuGuugaaag................................................    1     1    7y1
...............................................gcugcgaucuauuCaaag................................................    1     1    7y1
...............................................gcugcgaucuauugGaag................................................    1     1    7y1
...............................................gcugcgaAcuauugaaag................................................    1     1    7y1
...............................................gcAgcgaucuauugaaag................................................    4     1    7y1
...............................................gcugcgaucuauugaUag................................................    2     1    7y1
...............................................gGugcgaucuauugaaag................................................    2     1    7y1
...............................................Ccugcgaucuauugaaag................................................    8     1    7y1
...............................................gcugcgCucuauugaaag................................................    1     1    7y1
...............................................gcugGgaucuauugaaag................................................    1     1    7y1
...............................................gcugcgaucuauAgaaag................................................    8     1    7y1
...............................................gcugcgaucuUuugaaag................................................    1     1    7y1
...............................................gcugcgaucuauugaGag................................................    2     1    7y1
...............................................Acugcgaucuauugaaag................................................    1     1    7y1
...............................................gcugcgaucuaCugaaag................................................    1     1    7y1
...............................................Ncugcgaucuauugaaag................................................    2     1    7y1
...............................................gcugAgaucuauugaaag................................................    6     1    7y1
...............................................gcuAcgaucuauugaaag................................................    1     1    7y1
...............................................gcugcgaucuauugaaaU................................................    1     1    7y1
...............................................gcugcgaucuauugaaUg................................................    1     1    7y1
...............................................gcAgcgaucuauugaaagu...............................................    2     1    7y1
...............................................gcugcgaucuauugaaagu...............................................    111   0    7y1
...............................................gcugcgaucuauugaaagA...............................................    1     1    7y1
...............................................gcugcgaAcuauugaaagu...............................................    1     1    7y1
...............................................gAugcgaucuauugaaagu...............................................    2     1    7y1
...............................................gcugcgaucuauAgaaagu...............................................    1     1    7y1
...............................................gcugcgaucuauugaaagAc..............................................    2     1    7y1
...............................................gcugAgaucuauugaaaguc..............................................    1     1    7y1
...............................................gcugcgaAcuauugaaaguc..............................................    1     1    7y1
...............................................gcugcgaucuauugaaaguU..............................................    1     1    7y1
...............................................gcugcgaucuauAgaaaguc..............................................    1     1    7y1
...............................................gcuAcgaucuauugaaaguc..............................................    1     1    7y1
...............................................gAugcgaucuauugaaaguc..............................................    1     1    7y1
...............................................gcugcgaucuauugaaaUuc..............................................    1     1    7y1
...............................................Ucugcgaucuauugaaaguc..............................................    2     1    7y1
...............................................gcugcgaucuaAugaaaguc..............................................    1     1    7y1
...............................................gcugcgaucGauugaaaguc..............................................    1     1    7y1
```

**Star** **Mature**

```
gaccugcuucugggucgggguuucguacguagcagagcagcucccucgcugcgaucuauugaaagucagcccucgacacaaggguuuguccgcgcgcgcgcgcgcgcgugcgugc
...............................................gcugcgaucuauuNaaaguc................................................ 1 1 7y1
...............................................gcugcgaucuaCugaaaguc................................................ 1 1 7y1
...............................................gcugcgaucAauugaaaguc................................................ 1 1 7y1
...............................................gcugcgaGcuauugaaaguc................................................ 1 1 7y1
...............................................gcugcgaucuauugaaaguA................................................ 1 1 7y1
...............................................gcuCcgaucuauugaaaguc................................................ 1 1 7y1
...............................................gcugcgaucuauugaaaguc................................................ 562 0 7y1
...............................................Acugcgaucuauugaaagucag.............................................. 1 1 7y1
...............................................gcugcgaucuauugaaagucag.............................................. 181 0 7y1
...............................................gAugcgaucuauugaaagucag.............................................. 2 1 7y1
................................................cugcgaAcuauugaaagu................................................. 2 1 7y1
................................................cugcgaucuauugaaagA................................................. 1 1 7y1
................................................Nugcgaucuauugaaagu................................................. 1 1 7y1
................................................cugcgaucuaAugaaagu................................................. 1 1 7y1
................................................cuAcgaucuauugaaagu................................................. 1 1 7y1
................................................cugcgaucuauugaaagu................................................. 301 0 7y1
................................................cugcgaucuauugaaaUu................................................. 1 1 7y1
................................................Gugcgaucuauugaaagu................................................. 1 1 7y1
................................................cAgcgaucuauugaaagu................................................. 1 1 7y1
................................................Augcgaucuauugaaagu................................................. 1 1 7y1
................................................cugAgaucuauugaaaguc................................................ 1 1 7y1
................................................cugcgaucuauugaaaguU................................................ 1 1 7y1
................................................cugcgaucuauAgaaaguc................................................ 1 1 7y1
................................................cuUcgaucuauugaaaguc................................................ 1 1 7y1
................................................cugcgaAcuauugaaaguc................................................ 1 1 7y1
................................................cAgcgaucuauugaaaguc................................................ 1 1 7y1
................................................cugcgauAuauugaaaguc................................................ 1 1 7y1
................................................cugcgaGcuauugaaaguc................................................ 1 1 7y1
................................................Augcgaucuauugaaaguc................................................ 3 1 7y1
................................................cugcgaucAauugaaaguc................................................ 1 1 7y1
................................................cugcgaucuauugaaagAc................................................ 1 1 7y1
................................................cugGgaucuauugaaaguc................................................ 1 1 7y1
................................................cugcUaucuauugaaaguc................................................ 1 1 7y1
................................................cugcgaucuauugaaaguc................................................ 436 0 7y1
................................................cugcgaucAauugaaagucagc............................................. 3 1 7y1
................................................cugcgaucCauugaaagucagc............................................. 337 1 7y1
................................................cugcgaucuauugaaagucagcccA.......................................... 1 1 7y1
................................................cuAcgaucuauugaaagucagcccu.......................................... 1 1 7y1
................................................Augcgaucuauugaaagucagcccu.......................................... 1 1 7y1
................................................cugcgaucuauugaaagucagcccu.......................................... 172 0 7y1
................................................cuCcgaucuauugaaagucagcccu.......................................... 1 1 7y1
................................................cugcgaucAauugaaagucagcccu.......................................... 1 1 7y1
................................................cuUcgaucuauugaaagucagcccu.......................................... 1 1 7y1
................................................cuUcgaucuauugaaagucagcccuc......................................... 169 1 7y1
................................................cugUgaucuauugaaagucagcccucgac...................................... 1 1 7y1
................................................cugcgaucuauugaaagucagcccucgac...................................... 218 0 7y1
................................................cugcgaucuauugaaaCucagcccucgac...................................... 1 1 7y1
................................................cugcgaucuauugaaagAcagcccucgac...................................... 1 1 7y1
................................................cugcgaucuauugaaagucagcccucgaA...................................... 1 1 7y1
.................................................ugAgaucuauugaaagucagc............................................. 1 1 7y1
.................................................ugcgaucuauugaaagucagc............................................. 702 0 7y1
.................................................ugcgaucuauugaaaguAagc............................................. 1 1 7y1
.................................................uCcgaucuauugaaagucagc............................................. 2 1 7y1
.................................................ugcgaucuauugaCagucagc............................................. 1 1 7y1
.................................................ugcgauAuauugaaagucagc............................................. 2 1 7y1
.................................................ugcgaucuauugaaagucagG............................................. 1 1 7y1
.................................................Ggcgaucuauugaaagucagc............................................. 2 1 7y1
.................................................ugcgaucuauugaaagucagU............................................. 1 1 7y1
.................................................ugcgaucuauCgaaagucagc............................................. 1 1 7y1
.................................................ugcgaucuauugaaagAcagc............................................. 1 1 7y1
.................................................Agcgaucuauugaaagucagc............................................. 10 1 7y1
.................................................ugcgaucuauuAaaagucagc............................................. 1 1 7y1
.................................................ugcgaucuauuCaaagucagc............................................. 1 1 7y1
.................................................ugcgaucuauugaaagucagcAcucgacac.................................... 1 1 7y1
.................................................ugcgaucuauugaaagucagcccucgacac.................................... 154 0 7y1
.................................................ugcgaucuauugaaagucagcccuUgacac.................................... 1 1 7y1
.................................................Agcgaucuauugaaagucagcccucgacac.................................... 1 1 7y1
.................................................ugAgaucuauugaaagucagcccucgacac.................................... 1 1 7y1
.................................................ugcgaucuauugaaagucagcccucgUcac.................................... 1 1 7y1
.................................................ugcgaucuauugaaagucagcccucgacaA.................................... 1 1 7y1
```

**Star** **Mature**

```
gaccugcuucugggucggggguucguacguagcagagcagcucccucgcugcgaucuauugaaagucagcccucgacacaaggguuuguccgcgcgcgcgcgcgcgugcgugc
..................................................gcgaucuauAgaaagucagc...........................................    1     1     7y1
..................................................gcgaucuauuCaaagucagc...........................................    1     1     7y1
..................................................gcgaucAauugaaagucagc...........................................    1     1     7y1
..................................................gcgaucuauugaaagCcagc...........................................    1     1     7y1
..................................................gcgaucCauugaaagucagc...........................................    157   1     7y1
..................................................gcgaucuauugaaagucagc...........................................    259   0     7y1
..................................................gcgaucuauugaGagucagc...........................................    390   1     7y1
..................................................gcgaucuauAgaaagucagcc..........................................    2     1     7y1
..................................................gcgaucuauuAaaagucagcc..........................................    1     1     7y1
..................................................gcgaucuauugaaaCucagcc..........................................    1     1     7y1
..................................................gcgaucuauugaaagucagcc..........................................    149   0     7y1
...................................................cgaucuauugaaagucagA...........................................    3     1     7y1
...................................................cgaucuauAgaaagucagc...........................................    4     1     7y1
...................................................cgaucuauugaaGgucagc...........................................    1     1     7y1
...................................................cgaucuauuUaaagucagc...........................................    1     1     7y1
...................................................cgauAuauugaaagucagc...........................................    1     1     7y1
...................................................Agaucuauugaaagucagc...........................................    1     1     7y1
...................................................cgaucuauugUaagucagc...........................................    1     1     7y1
...................................................cgaucuauugaaagucagc...........................................    565   0     7y1
...................................................cgaucAauugaaagucagc...........................................    2     1     7y1
...................................................cgaucuauGgaaagucagc...........................................    1     1     7y1
...................................................cgaucuauugaaaguGagc...........................................    1     1     7y1
...................................................cgaucuauugaaaguAagc...........................................    1     1     7y1
...................................................cUaucuauugaaagucagc...........................................    1     1     7y1
...................................................cgaucuauCgaaagucagc...........................................    1     1     7y1
...................................................cgaucuauugaaagucCgcccu........................................    1     1     7y1
...................................................cgaucuauugaaaUucagcccu........................................    1     1     7y1
...................................................cgaucuauugaaagucagcUcu........................................    1     1     7y1
...................................................cgaucAauugaaagucagcccu........................................    1     1     7y1
...................................................cgaucuauAgaaagucagcccu........................................    1     1     7y1
...................................................cgaucuauugaaagucagcccu........................................    243   0     7y1
...................................................cgaucuauugaaaguGagcccu........................................    1     1     7y1
...................................................cUaucuauugaaagucagcccu........................................    2     1     7y1
...................................................cgaucuaAugaaagucagcccu........................................    1     1     7y1
...................................................Ggaucuauugaaagucagcccucg......................................    1     1     7y1
...................................................cgauAuauugaaagucagcccucg......................................    1     1     7y1
...................................................Agaucuauugaaagucagcccucg......................................    2     1     7y1
...................................................cgaucAauugaaagucagcccucg......................................    2     1     7y1
...................................................cgaucuauugaaagAcagcccucg......................................    1     1     7y1
...................................................cgaucuauCgaaagucagcccucg......................................    1     1     7y1
...................................................cgaAcuauugaaagucagcccucg......................................    1     1     7y1
...................................................cgaucuauAgaaagucagcccucg......................................    1     1     7y1
...................................................cgaucuaGugaaagucagcccucg......................................    1     1     7y1
...................................................cgaucuauugaGagucagcccucg......................................    1     1     7y1
...................................................cCaucuauugaaagucagcccucg......................................    1     1     7y1
...................................................cgaucuauugaaagucagAccucg......................................    1     1     7y1
...................................................cgaucuauugaaagucagcccucg......................................    478   0     7y1
...................................................Ngaucuauugaaagucagcccucg......................................    1     1     7y1
...................................................cgUucuauugaaagucagcccucg......................................    1     1     7y1
...................................................Ngaucuauugaaagucagcccucgacacaa................................    1     1     7y1
...................................................Agaucuauugaaagucagcccucgacacaa................................    1     1     7y1
...................................................cgaucuauugaaagucagcccucgacacaG................................    1     1     7y1
...................................................cgaucAauugaaagucagcccucgacacaa................................    1     1     7y1
...................................................cgaucuauugaaagucagcccucgacacaa................................    237   0     7y1
...................................................cgaucuauugaaagucagcccucgCcacaa................................    1     1     7y1
...................................................cgauAuauugaaagucagcccucgacacaa................................    2     1     7y1
...................................................cgaucuauugaaagAcagcccucgacacaa................................    1     1     7y1
....................................................gaucuauuUaaagucagc...........................................    1     1     7y1
....................................................gaucCauugaaagucagc...........................................    142   1     7y1
....................................................gaucuauugaaagucagc...........................................    390   0     7y1
....................................................gaAcuauugaaagucagc...........................................    1     1     7y1
....................................................gaucAauugaaagucagc...........................................    2     1     7y1
....................................................gaucuauugaaagucagG...........................................    1     1     7y1
....................................................gauAuauugaaagucagc...........................................    1     1     7y1
....................................................gaucAauugaaagucagcc..........................................    2     1     7y1
....................................................gaucuauugaaaUucagcc..........................................    1     1     7y1
....................................................gaucuauuCaaagucagcc..........................................    2     1     7y1
....................................................gaucuaAugaaagucagcc..........................................    1     1     7y1
....................................................gaucuauugaaagucaCcc..........................................    1     1     7y1
....................................................gaucuauugaaagucGgcc..........................................    1     1     7y1
```

**Star** **Mature**

```
gaccugcuucugggucggggguuucguacguagcagagcagcucccucgcugcgaucuauugaaagucagcccucgacacaaggguuuguccgcgcgcgcgcgcgcgugcgugc
...................................................gauGuauugaaagucagcc...........................................  1     1  7y1
...................................................gauAuauugaaagucagcc...........................................  3     1  7y1
...................................................gaucuauugaaagucagcA...........................................  1     1  7y1
...................................................gaucuauugaaagucagcG...........................................  1     1  7y1
...................................................gGucuauugaaagucagcc...........................................  1     1  7y1
...................................................gaucuauugaaaCucagcc...........................................  2     1  7y1
...................................................Caucuauugaaagucagcc...........................................  1     1  7y1
...................................................gaucuauugaaagucagcc...........................................  683   0  7y1
...................................................gaucuUuugaaagucagcc...........................................  1     1  7y1
...................................................gaucAauugaaagucagcccucg.......................................  2     1  7y1
...................................................gaucCauugaaagucagcccucg.......................................  193   1  7y1
....................................................aucuauugaaagucagcA...........................................  13    1  7y1
....................................................aucuauugaaagucagcc...........................................  5120  0  7y1
....................................................aucuauugaaagucaCcc...........................................  5     1  7y1
....................................................aucuauugaaaAucagcc...........................................  1     1  7y1
....................................................aucCauugaaagucagcc...........................................  1243  1  7y1
....................................................aucuauugaaagucaUcc...........................................  2     1  7y1
....................................................aucuUuugaaagucagcc...........................................  1     1  7y1
....................................................aucuauugaaagGcagcc...........................................  1     1  7y1
....................................................aucuauugaaaCucagcc...........................................  4     1  7y1
....................................................aucuauugaaagucagUc...........................................  2     1  7y1
....................................................aucuauugaaagAcagcc...........................................  6     1  7y1
....................................................aucuaAugaaagucagcc...........................................  23    1  7y1
....................................................auGuauugaaagucagcc...........................................  1     1  7y1
....................................................aAcuauugaaagucagcc...........................................  19    1  7y1
....................................................aucuauugaUagucagcc...........................................  1     1  7y1
....................................................auAuauugaaagucagcc...........................................  27    1  7y1
....................................................aucuauAgaaagucagcc...........................................  21    1  7y1
....................................................aucuaCugaaagucagcc...........................................  1     1  7y1
....................................................aucAauugaaagucagcc...........................................  10    1  7y1
....................................................aucuauugaaagucagcG...........................................  2     1  7y1
....................................................aucuauuCaaagucagcc...........................................  4     1  7y1
....................................................aucuauugaaGgucagcc...........................................  1     1  7y1
....................................................aucuauuUaaagucagcc...........................................  3     1  7y1
....................................................Uucuauugaaagucagcc...........................................  3     1  7y1
....................................................Nucuauugaaagucagcc...........................................  5     1  7y1
....................................................aucuauugaaaUucagcc...........................................  2     1  7y1
....................................................Gucuauugaaagucagcc...........................................  4     1  7y1
....................................................aucuauugaaUgucagcc...........................................  1     1  7y1
....................................................aucuauuAaaagucagcc...........................................  2     1  7y1
....................................................aucuauugaaaguAagcc...........................................  12    1  7y1
....................................................aucuauAgaaagucagccc..........................................  8     1  7y1
....................................................aucuUuugaaagucagccc..........................................  1     1  7y1
....................................................aucuauugaaagucaUccc..........................................  1     1  7y1
....................................................aucuauugaaagucagccU..........................................  237   1  7y1
....................................................aucuaCugaaagucagccc..........................................  1     1  7y1
....................................................aucuauugaaagucagccc..........................................  1694  0  7y1
....................................................aucuauugaaagAcagccc..........................................  1     1  7y1
....................................................aucuaAugaaagucagccc..........................................  5     1  7y1
....................................................auGuauugaaagucagccc..........................................  2     1  7y1
....................................................aucGauugaaagucagccc..........................................  2     1  7y1
....................................................aucuauugaaaUucagccc..........................................  2     1  7y1
....................................................aucuauuUaaagucagccc..........................................  1     1  7y1
....................................................aucuauugaaagucagccA..........................................  6     1  7y1
....................................................aucuauugaaagucagcAc..........................................  4     1  7y1
....................................................aucuauugaUagucagccc..........................................  1     1  7y1
....................................................aAcuauugaaagucagccc..........................................  10    1  7y1
....................................................aucuauugUaagucagccc..........................................  1     1  7y1
....................................................aucuauugaaaCucagccc..........................................  1     1  7y1
....................................................aucAauugaaagucagccc..........................................  1     1  7y1
....................................................aucCauugaaagucagccc..........................................  209   1  7y1
....................................................Nucuauugaaagucagccc..........................................  1     1  7y1
....................................................auAuauugaaagucagccc..........................................  7     1  7y1
....................................................aucuauugaaaguAagccc..........................................  5     1  7y1
....................................................Gucuauugaaagucagccc..........................................  2     1  7y1
....................................................aucuauugaaagucagccG..........................................  3     1  7y1
....................................................aucuauugaaagucagcGc..........................................  2     1  7y1
....................................................Nucuauugaaagucagcccu.........................................  1     1  7y1
....................................................aucuauugaaagAcagcccu.........................................  1     1  7y1
....................................................aucuauugaaUgucagcccu.........................................  1     1  7y1
```

**Star** **Mature**

```
gaccugcuucugggucgggguuucguacguagcagagcagcucccucgcugcgaucuauugaaagucagcccucgacacaaggguuuguccgcgcgcgcgcgcgcgugcgugc
.....................................................aucuauugaaagucagcAcu........................................  2     1  7y1
.....................................................aucuauuCaaagucagcccu........................................  1     1  7y1
.....................................................aucuauugaaagucaUcccu........................................  1     1  7y1
.....................................................auAuauugaaagucagcccu........................................  2     1  7y1
.....................................................aucuauugaaagucagcccG........................................  2     1  7y1
.....................................................aucuaAugaaagucagcccu........................................  4     1  7y1
.....................................................aucuauugaaagucagcccA........................................  1     1  7y1
.....................................................aucuauugaaagucagcccu........................................  755   0  7y1
.....................................................aucuauAgaaagucagcccu........................................  3     1  7y1
.....................................................aAcuauugaaagucagcccu........................................  2     1  7y1
.....................................................aucuauugaaagucagccAu........................................  1     1  7y1
.....................................................aucCauugaaagucagcccu........................................  223   1  7y1
.....................................................aucuauugaaaguAagcccu........................................  1     1  7y1
.....................................................aucCauugaaagucagcccuc.......................................  1285  1  7y1
.....................................................aucAauugaaagucagcccuc.......................................  2     1  7y1
.....................................................aucuauAgaaagucagcccucg......................................  4     1  7y1
.....................................................aucuauugaaagucagcccucg......................................  1785  0  7y1
.....................................................aucuauugaaagucagcccucU......................................  1     1  7y1
.....................................................aucuauugaaagucagcAcucg......................................  2     1  7y1
.....................................................aucuauugaaagucagAccucg......................................  6     1  7y1
.....................................................aCcuauugaaagucagcccucg......................................  1     1  7y1
.....................................................aucuauuUaaagucagcccucg......................................  1     1  7y1
.....................................................aucuauugaaaguAagcccucg......................................  5     1  7y1
.....................................................aucuauugNaagucagcccucg......................................  1     1  7y1
.....................................................aucuauugaaagAcagcccucg......................................  2     1  7y1
.....................................................aucuaAugaaagucagcccucg......................................  6     1  7y1
.....................................................aAcuauugaaagucagcccucg......................................  5     1  7y1
.....................................................auAuauugaaagucagcccucg......................................  8     1  7y1
.....................................................aucuauugaaagucagcccAcg......................................  1     1  7y1
.....................................................aucuauugaaagucagUccucg......................................  2     1  7y1
.....................................................aucuauugaaagucagGccucg......................................  1     1  7y1
.....................................................aucuauugaaaUucagcccucg......................................  1     1  7y1
.....................................................Gucuauugaaagucagcccucg......................................  1     1  7y1
.....................................................aucuauugaUagucagcccucg......................................  1     1  7y1
.....................................................aucuauugaaagucGgcccucg......................................  2     1  7y1
.....................................................aucCauugaaagucagcccucg......................................  914   1  7y1
.....................................................aucuauuCaaagucagcccucg......................................  2     1  7y1
.....................................................aucuauugaaagucagccAucg......................................  2     1  7y1
.....................................................aucuauugaaagucaCccucg.......................................  1     1  7y1
.....................................................auUuauugaaagucagcccucg......................................  249   1  7y1
.....................................................aucuauugGaagucagcccucg......................................  1     1  7y1
.....................................................aucuauugaaagucagcccucC......................................  2     1  7y1
.....................................................Nucuauugaaagucagcccucg......................................  1     1  7y1
.....................................................aucuauugaaagucagcccuAg......................................  4     1  7y1
.....................................................aucuauugaaaguGagcccucg......................................  1     1  7y1
.....................................................aucuauugaGagucagcccucg......................................  1     1  7y1
.....................................................aucAauugaaagucagcccucg......................................  1     1  7y1
.....................................................aucuauugaaagucaUcccucg......................................  1     1  7y1
.....................................................aucuauugaaagucagcAcucga.....................................  2     1  7y1
.....................................................aucuauugaaaguAagcccucga.....................................  1     1  7y1
.....................................................aucuauugaaagucagccAucga.....................................  2     1  7y1
.....................................................aucuauugaaagucagcUcucga.....................................  1     1  7y1
.....................................................aucuauugaaagucagcccucga.....................................  460   0  7y1
.....................................................auAuauugaaagucagcccucga.....................................  2     1  7y1
.....................................................aucuauugaaagucagcccuAga.....................................  1     1  7y1
.....................................................aucuauugaaagucagccGucga.....................................  1     1  7y1
.....................................................aucuauAgaaagucagcccucga.....................................  6     1  7y1
.....................................................aucuaAugaaagucagcccucga.....................................  2     1  7y1
.....................................................aAcuauugaaagucagcccucga.....................................  3     1  7y1
.....................................................aucuauCgaaagucagcccucga.....................................  1     1  7y1
.....................................................aucuauugUaagucagcccucga.....................................  1     1  7y1
.....................................................aucuauugaaagucagcccucgac....................................  272   0  7y1
.....................................................Uucuauugaaagucagcccucgac....................................  2     1  7y1
.....................................................aucuauAgaaagucagcccucgac....................................  2     1  7y1
.....................................................aucuaAugaaagucagcccucgac....................................  2     1  7y1
.....................................................aucuauugaaagucagcccucgaA....................................  1     1  7y1
.....................................................aAcuauugaaagucagcccucgac....................................  1     1  7y1
.....................................................aucuauugaaagucaAcccucgacacaag...............................  1     1  7y1
.....................................................aucuauugaaagucagcccucUacacaag...............................  1     1  7y1
.....................................................aucuauugaaagucagccAucgacacaag...............................  1     1  7y1
```

**Star** **Mature**

| | | | |
|---|---|---|---|
| gaccugcuucugggucgggguuucguacguagcagagcagcucccucgcugcgaucuauugaaagucagcccucgacacaaggguuuguccgcgcgcgcgcgcgugcgugc | | | |
| .....................................................Nucuauugaaagucagcccucgacacaag............................. | 1 | 1 | 7y1 |
| .....................................................aAcuauugaaagucagcccucgacacaag............................. | 1 | 1 | 7y1 |
| .....................................................aucuauugaaagucagcccAcgacacaag............................. | 1 | 1 | 7y1 |
| .....................................................aucuauugaaagucagcccucgCcacaag............................. | 1 | 1 | 7y1 |
| .....................................................aucuaAugaaagucagcccucgacacaag............................. | 1 | 1 | 7y1 |
| .....................................................aucuauugaaagucCgcccucgacacaag............................. | 1 | 1 | 7y1 |
| .....................................................Uucuauugaaagucagcccucgacacaag............................. | 1 | 1 | 7y1 |
| .....................................................aucuauugaaagucagcccucgacUcaag............................. | 1 | 1 | 7y1 |
| .....................................................auAuauugaaagucagcccucgacacaag............................. | 4 | 1 | 7y1 |
| .....................................................aucuauugaaagucagcccucgacacaag............................. | 494 | 0 | 7y1 |
| .....................................................aucAauugaaagucagcccucgacacaag............................. | 1 | 1 | 7y1 |
| .....................................................aucuauugaaagucagcGcucgacacaag............................. | 1 | 1 | 7y1 |
| .....................................................aucuauugaaagucagcccucgacaGaag............................. | 1 | 1 | 7y1 |
| .....................................................aucuauuUaaagucagcccucgacacaag............................. | 1 | 1 | 7y1 |
| ......................................................ucuauugaaagucagGcc....................................... | 1 | 1 | 7y1 |
| ......................................................ucuauugaaagucagccA....................................... | 6 | 1 | 7y1 |
| ......................................................ucuauugaaagucaUccc....................................... | 2 | 1 | 7y1 |
| ......................................................ucuauugaaagucagccc....................................... | 1308 | 0 | 7y1 |
| ......................................................ucuauugaaaCucagccc....................................... | 1 | 1 | 7y1 |
| ......................................................ucuauugaaagucaAccc....................................... | 1 | 1 | 7y1 |
| ......................................................ucuauugaaagucCgccc....................................... | 1 | 1 | 7y1 |
| ......................................................ucuauuUaaagucagccc....................................... | 3 | 1 | 7y1 |
| ......................................................ucuauugaaaguAagccc....................................... | 3 | 1 | 7y1 |
| ......................................................ucuauuAaaagucagccc....................................... | 2 | 1 | 7y1 |
| ......................................................ucuaAugaaagucagccc....................................... | 3 | 1 | 7y1 |
| ......................................................Gcuauugaaagucagccc....................................... | 2 | 1 | 7y1 |
| ......................................................ucuauugaaagucagcAc....................................... | 1 | 1 | 7y1 |
| ......................................................ucuGuugaaagucagccc....................................... | 1 | 1 | 7y1 |
| ......................................................ucuauugaaagucagUcc....................................... | 4 | 1 | 7y1 |
| ......................................................ucuauAgaaagucagccc....................................... | 3 | 1 | 7y1 |
| ......................................................ucuauugaGagucagccc....................................... | 227 | 1 | 7y1 |
| ......................................................uAuauugaaagucagccc....................................... | 5 | 1 | 7y1 |
| ......................................................ucuauugaaagucagccG....................................... | 1 | 1 | 7y1 |
| ......................................................ucAauugaaagucagccc....................................... | 3 | 1 | 7y1 |
| ......................................................Acuauugaaagucagccc....................................... | 16 | 1 | 7y1 |
| ......................................................ucuauugaaagucagccAu...................................... | 5 | 1 | 7y1 |
| ......................................................ucuauugaaagGcagcccu...................................... | 1 | 1 | 7y1 |
| ......................................................ucuaAugaaagucagcccu...................................... | 1 | 1 | 7y1 |
| ......................................................ucuauugaaagucagcAcu...................................... | 3 | 1 | 7y1 |
| ......................................................Ncuauugaaagucagcccu...................................... | 1 | 1 | 7y1 |
| ......................................................ucuauugaaagucCgcccu...................................... | 1 | 1 | 7y1 |
| ......................................................ucGauugaaagucagcccu...................................... | 1 | 1 | 7y1 |
| ......................................................Acuauugaaagucagcccu...................................... | 6 | 1 | 7y1 |
| ......................................................ucuauAgaaagucagcccu...................................... | 3 | 1 | 7y1 |
| ......................................................ucuauugaaagucagcccG...................................... | 1 | 1 | 7y1 |
| ......................................................ucAauugaaagucagcccu...................................... | 1 | 1 | 7y1 |
| ......................................................ucuauugaaagucagUccu...................................... | 1 | 1 | 7y1 |
| ......................................................ucuauugaaagucagcccA...................................... | 1 | 1 | 7y1 |
| ......................................................ucuauuAaaagucagcccu...................................... | 1 | 1 | 7y1 |
| ......................................................ucuauugaaagucagcccu...................................... | 861 | 0 | 7y1 |
| ......................................................ucuauugaaaCucagcccu...................................... | 1 | 1 | 7y1 |
| ......................................................ucAauugaaagucagcccuc..................................... | 1 | 1 | 7y1 |
| ......................................................ucCauugaaagucagcccuc..................................... | 278 | 1 | 7y1 |
| ......................................................ucuauugaaagucCgcccucg.................................... | 1 | 1 | 7y1 |
| ......................................................ucAauugaaagucagcccucg.................................... | 1 | 1 | 7y1 |
| ......................................................ucuauugaaagucagccGucg.................................... | 1 | 1 | 7y1 |
| ......................................................ucuauAgaaagucagcccucg.................................... | 3 | 1 | 7y1 |
| ......................................................ucuauCgaaagucagcccucg.................................... | 1 | 1 | 7y1 |
| ......................................................ucuauugaaaUucagcccucg.................................... | 1 | 1 | 7y1 |
| ......................................................ucuauugaaagucagUccucg.................................... | 1 | 1 | 7y1 |
| ......................................................Gcuauugaaagucagcccucg.................................... | 1 | 1 | 7y1 |
| ......................................................ucCauugaaagucagcccucg.................................... | 282 | 1 | 7y1 |
| ......................................................ucuaAugaaagucagcccucg.................................... | 2 | 1 | 7y1 |
| ......................................................uAuauugaaagucagcccucg.................................... | 1 | 1 | 7y1 |
| ......................................................Ccuauugaaagucagcccucg.................................... | 1 | 1 | 7y1 |
| ......................................................ucuauugaaagucagcccucU.................................... | 1 | 1 | 7y1 |
| ......................................................ucuauugaaaguAagcccucg.................................... | 2 | 1 | 7y1 |
| ......................................................ucuauugaaagucagcAcucg.................................... | 3 | 1 | 7y1 |
| ......................................................ucuauugaaagucaCcccucg.................................... | 1 | 1 | 7y1 |
| ......................................................ucuauugaaagucagcUcucg.................................... | 2 | 1 | 7y1 |

**Star** **Mature**

| | | | |
|---|---|---|---|
| gaccugcuucugggucgggguuucguacguagcagagcagcucccucgcugcgaucuauugaaagucagcccucgacacaaggguuuguccgcgcgcgcgcgcgcgugcgugc | | | |
| ......................................................Acuauugaaagucagcccucg...................................... | 9 | 1 | 7y1 |
| ......................................................ucuauugaaagucagccAucg...................................... | 2 | 1 | 7y1 |
| ......................................................ucuauugaaagucagcccucA...................................... | 1 | 1 | 7y1 |
| ......................................................ucuauugaaagucagcccucg...................................... | 1220 | 0 | 7y1 |
| ......................................................ucuauugaaagucagcUcucga..................................... | 1 | 1 | 7y1 |
| ......................................................Gcuauugaaagucagcccucga..................................... | 2 | 1 | 7y1 |
| ......................................................ucuauugaaGgucagcccucga..................................... | 1 | 1 | 7y1 |
| ......................................................ucAauugaaagucagcccucga..................................... | 1 | 1 | 7y1 |
| ......................................................uAuauugaaagucagcccucga..................................... | 3 | 1 | 7y1 |
| ......................................................Acuauugaaagucagcccucga..................................... | 3 | 1 | 7y1 |
| ......................................................ucuaCugaaagucagcccucga..................................... | 1 | 1 | 7y1 |
| ......................................................ucuaAugaaagucagcccucga..................................... | 1 | 1 | 7y1 |
| ......................................................ucuauuUaaagucagcccucga..................................... | 1 | 1 | 7y1 |
| ......................................................ucuauugaaagucagcccucga..................................... | 428 | 0 | 7y1 |
| ......................................................ucuauugaaagucagcccucgG..................................... | 2 | 1 | 7y1 |
| ......................................................ucuauugaaaguAagcccucga..................................... | 1 | 1 | 7y1 |
| ......................................................ucuauAgaaagucagcccucga..................................... | 2 | 1 | 7y1 |
| ......................................................Gcuauugaaagucagcccucgac................................... | 1 | 1 | 7y1 |
| ......................................................ucuauugaUagucagcccucgac................................... | 1 | 1 | 7y1 |
| ......................................................uAuauugaaagucagcccucgac................................... | 1 | 1 | 7y1 |
| ......................................................ucuauugaaagucagcccucgac................................... | 299 | 0 | 7y1 |
| ......................................................ucuauugaaagucagccAucgac................................... | 1 | 1 | 7y1 |
| ......................................................ucAauugaaagucagcccucgac................................... | 1 | 1 | 7y1 |
| ......................................................ucuauugaaagucagcccAcgac................................... | 1 | 1 | 7y1 |
| ......................................................Acuauugaaagucagcccucgac................................... | 2 | 1 | 7y1 |
| .......................................................cuauCgaaagucagcccu........................................ | 1 | 1 | 7y1 |
| .......................................................cuauugaaagucagcccG........................................ | 1 | 1 | 7y1 |
| .......................................................cuauugaaagucagcccu........................................ | 416 | 0 | 7y1 |
| .......................................................cuaAugaaagucagcccu........................................ | 1 | 1 | 7y1 |
| .......................................................cAauugaaagucagcccu........................................ | 2 | 1 | 7y1 |
| .......................................................cuauugaaaAucagcccu........................................ | 1 | 1 | 7y1 |
| .......................................................Auauugaaagucagcccu........................................ | 1 | 1 | 7y1 |
| .......................................................cuauugaaaguAagcccu........................................ | 1 | 1 | 7y1 |
| .......................................................cuauugaaaCucagcccu........................................ | 1 | 1 | 7y1 |
| .......................................................cuauugaaagAcagcccuc....................................... | 2 | 1 | 7y1 |
| .......................................................cuauugaaagucagcccuc....................................... | 186 | 0 | 7y1 |
| .......................................................cCauugaaagucagcccuc....................................... | 488 | 1 | 7y1 |
| .......................................................cuauugaUagucagcccuc....................................... | 1 | 1 | 7y1 |
| .......................................................cAauugaaagucagcccuc....................................... | 5 | 1 | 7y1 |
| .......................................................Guauugaaagucagcccuc....................................... | 1 | 1 | 7y1 |
| .......................................................cCauugaaagucagcccucg...................................... | 701 | 1 | 7y1 |
| .......................................................cAauugaaagucagcccucg...................................... | 4 | 1 | 7y1 |
| .......................................................cAauugaaagucagcccucgac.................................... | 1 | 1 | 7y1 |
| .......................................................cuauugaaagucagcccucgac.................................... | 273 | 0 | 7y1 |
| .......................................................cuauugaaagucagcAcucgac.................................... | 1 | 1 | 7y1 |
| .......................................................cuauugaaagucGgcccucgac.................................... | 1 | 1 | 7y1 |
| .......................................................cuauugaaagucagccAucgac.................................... | 3 | 1 | 7y1 |
| .......................................................Auauugaaagucagcccucgac.................................... | 1 | 1 | 7y1 |
| .......................................................cuauugaaagAcagcccucgac.................................... | 1 | 1 | 7y1 |
| .......................................................cuauugaaagucagcccucgacacaaggguuu.......................... | 1 | 0 | 7y1 |
| ........................................................uauugaaaUucagcccucg...................................... | 1 | 1 | 7y1 |
| ........................................................Aauugaaagucagcccucg...................................... | 6 | 1 | 7y1 |
| ........................................................uauugaaagucagcccucC...................................... | 1 | 1 | 7y1 |
| ........................................................uauugaaagucagcGcucg...................................... | 1 | 1 | 7y1 |
| ........................................................uauugaaagucagcccAcg...................................... | 2 | 1 | 7y1 |
| ........................................................uauugaaagucagcccucg...................................... | 490 | 0 | 7y1 |
| ........................................................uauugaaGgucagcccucg...................................... | 1 | 1 | 7y1 |
| ........................................................uauugaaagucagcccuAg...................................... | 1 | 1 | 7y1 |
| ........................................................uauugaaagAcagcccucg...................................... | 1 | 1 | 7y1 |
| ........................................................uauugCaagucagcccucga..................................... | 1 | 1 | 7y1 |
| ........................................................uauugaaagucagcccucgG..................................... | 2 | 1 | 7y1 |
| ........................................................uauugaaagucaCcccucga..................................... | 1 | 1 | 7y1 |
| ........................................................uauugaaUgucagcccucga..................................... | 1 | 1 | 7y1 |
| ........................................................Aauugaaagucagcccucga..................................... | 2 | 1 | 7y1 |
| ........................................................Gauugaaagucagcccucga..................................... | 1 | 1 | 7y1 |
| ........................................................uauugaaagucagcccucga..................................... | 197 | 0 | 7y1 |
| ........................................................uauugaaagucagcccucgac.................................... | 549 | 0 | 7y1 |
| ........................................................uaAugaaagucagcccucgac.................................... | 4 | 1 | 7y1 |
| ........................................................uauugaaagAcagcccucgac.................................... | 1 | 1 | 7y1 |
| ........................................................uauugaaaUucagcccucgac.................................... | 1 | 1 | 7y1 |

Star Mature

```
gaccugcuucugggucggggguuucguacguagcagagcagcucccucgcugcgaucuauugaaagucagcccucgacacaaggguuuguccgcgcgcgcgcgcgcgugcgugc
........................................................uauugaaagucagcAcucgac....................................    1    1    7y1
........................................................uauAgaaagucagcccucgac....................................    2    1    7y1
........................................................Aauugaaagucagcccucgac....................................    11   1    7y1
........................................................uauugaaagucagcccucUac....................................    1    1    7y1
........................................................uauugaaagucagcccCcgac....................................    1    1    7y1
........................................................uauugaaagucaCcccucgac....................................    1    1    7y1
........................................................uauugaaagucaUcccucgac....................................    1    1    7y1
........................................................uauugaaagucagAccucgac....................................    1    1    7y1
........................................................uauugaaagucagcccucgaGacaaggguu...........................    1    1    7y1
........................................................uauuCaaagucagcccucgacacaaggguu...........................    1    1    7y1
........................................................uauugaaaguAagcccucgacacaaggguu...........................    1    1    7y1
........................................................uauugaaagucagcAcucgacacaaggguu...........................    2    1    7y1
........................................................uauugaaagucagcccucgacacaaggAuu...........................    1    1    7y1
........................................................Aauugaaagucagcccucgacacaaggguu...........................    1    1    7y1
........................................................uauugaaagucagcccucgacacaaggguu...........................    155  0    7y1
........................................................uauugaaagucagcccAcgacacaaggguu...........................    1    1    7y1
........................................................uauugaaaUucagcccucgacacaaggguu...........................    1    1    7y1
........................................................uauugaaagucagcccucgacacaaggCuu...........................    1    1    7y1
........................................................uauugaaagucagcccucgacacaaggguG...........................    1    1    7y1
........................................................uauugaaagucagcccucgacaUaaggguuu..........................    1    1    7y1
........................................................uauugaaagucagcccucgacacaagggAuu..........................    1    1    7y1
........................................................uauugaaagucagAccucgacacaaggguuu..........................    1    1    7y1
........................................................uauugaaagucagcccucgacacaaggguuu..........................    220  0    7y1
........................................................uauugaaagucagcccAcgacacaaggguuu..........................    1    1    7y1
........................................................Aauugaaagucagcccucgacacaaggguuu..........................    7    1    7y1
........................................................uauugaaagucagccAucgacacaaggguuug.........................    1    1    7y1
........................................................uauugaaagucagcccuAgacacaaggguuug.........................    3    1    7y1
........................................................uauugaaagucagcccucgacacaaCgguuug.........................    1    1    7y1
........................................................uauugaaagucagcccucgacacaagggAuug.........................    2    1    7y1
........................................................uauugaaagucagcccucgacaAaaggguuug.........................    3    1    7y1
........................................................Nauugaaagucagcccucgacacaaggguuug.........................    1    1    7y1
........................................................uauugaaagucagcccucgacacaagggGuug.........................    1    1    7y1
........................................................Gauugaaagucagcccucgacacaaggguuug.........................    2    1    7y1
........................................................uauugaaaguAagcccucgacacaaggguuug.........................    2    1    7y1
........................................................uauugaaagucagcccucgacacaaggguuAg.........................    1    1    7y1
........................................................uauugaaagucagcccucgacacaagCguuug.........................    1    1    7y1
........................................................uauugaaagucagGccucgacacaaggguuug.........................    1    1    7y1
........................................................uauugaaagucGgcccucgacacaaggguuug.........................    1    1    7y1
........................................................uauuCaaagucagcccucgacacaaggguuug.........................    1    1    7y1
........................................................uauugaaagucagcccCcgacacaaggguuug.........................    1    1    7y1
........................................................uauugaaagucagcccAcgacacaaggguuug.........................    2    1    7y1
........................................................uauugaaagucagcGcucgacacaaggguuug.........................    1    1    7y1
........................................................uauugaaagucaCcccucgacacaaggguuug.........................    2    1    7y1
........................................................uauugaaagucagcccucgacacaagUguuug.........................    1    1    7y1
........................................................uauugaaagucagcAcucgacacaaggguuug.........................    6    1    7y1
........................................................uauugaaagucagAccucgacacaaggguuug.........................    1    1    7y1
........................................................uauugaaaUucagcccucgacacaaggguuug.........................    1    1    7y1
........................................................uauugaaagucagcccucgacacaaggguuug.........................    684  0    7y1
........................................................uauuAaaagucagcccucgacacaaggguuug.........................    1    1    7y1
........................................................Aauugaaagucagcccucgacacaaggguuug.........................    13   1    7y1
.........................................................auugaaagucagccAucg......................................    1    1    7y1
.........................................................auuUaaagucagcccucg......................................    1    1    7y1
.........................................................auAgaaagucagcccucg......................................    1    1    7y1
.........................................................auuCaaagucagcccucg......................................    1    1    7y1
.........................................................auugaaagucagcccucg......................................    487  0    7y1
.........................................................aAugaaagucagcccucg......................................    2    1    7y1
.........................................................auugaGagucagcccucg......................................    1    1    7y1
.........................................................auugaaaguAagcccucg......................................    2    1    7y1
.........................................................auugaaagucagUccucg......................................    1    1    7y1
.........................................................Uuugaaagucagcccucg......................................    1    1    7y1
.........................................................auugaaagucagcAcucg......................................    2    1    7y1
.........................................................auugaaagucagAccucg......................................    3    1    7y1
.........................................................auugaaagucagcccucC......................................    1    1    7y1
.........................................................auugaaagucagcccuAga.....................................    2    1    7y1
.........................................................auugaaagucaUcccucga.....................................    1    1    7y1
.........................................................auugaaagucagcccucgC.....................................    1    1    7y1
.........................................................auugaaagucagAccucga.....................................    1    1    7y1
.........................................................auugaaagucagcccAcga.....................................    1    1    7y1
.........................................................auugaaagucagccAucga.....................................    1    1    7y1
.........................................................auugaaagAcagcccucga.....................................    1    1    7y1
```

**Star** **Mature**

gaccugcuucugggucggggu uucguacguagcagagcagcucccucgcugcgaucuauugaaagucagcc cucgacacaaggguuugu ccgcgcgcgcgcgcgcgugcgugc

| | | | |
|---|---|---|---|
| .........................................................auugaaaguGagcccucga..................................... | 1 | 1 | 7y1 |
| .........................................................auugaaagucagcccucga..................................... | 452 | 0 | 7y1 |
| .........................................................aAugaaagucagcccucga..................................... | 1 | 1 | 7y1 |
| .........................................................auugaaagucagAccucgac.................................... | 1 | 1 | 7y1 |
| .........................................................auugaaagucagccAucgac.................................... | 3 | 1 | 7y1 |
| .........................................................aAugaaagucagcccucgac.................................... | 1 | 1 | 7y1 |
| .........................................................auugaaagucagcccucgac.................................... | 419 | 0 | 7y1 |
| .........................................................auugaaagucagUccucgac.................................... | 2 | 1 | 7y1 |
| .........................................................auugaaagucagcccucgCc.................................... | 2 | 1 | 7y1 |
| .........................................................auugaaagAcagcccucgac.................................... | 2 | 1 | 7y1 |
| .........................................................auugaaaguAagcccucgac.................................... | 2 | 1 | 7y1 |
| .........................................................auugaaagucagcccucgaca................................... | 189 | 0 | 7y1 |
| .........................................................aAugaaagucagcccucgaca................................... | 2 | 1 | 7y1 |
| .........................................................auugaaagucagcccucgaGa................................... | 1 | 1 | 7y1 |
| .........................................................auugaaaguAagcccucgaca................................... | 2 | 1 | 7y1 |
| .........................................................auugaaagucagcccucgacG................................... | 1 | 1 | 7y1 |
| .........................................................auugaaagucagcAcucgacaca................................. | 1 | 1 | 7y1 |
| .........................................................auugaaaguAagcccucgacaca................................. | 1 | 1 | 7y1 |
| .........................................................auugaaagucagcccucgacaca................................. | 165 | 0 | 7y1 |
| .........................................................auugaaagucagcccucgCcaca................................. | 4 | 1 | 7y1 |
| .........................................................aAugaaagucagcccucgacaca................................. | 1 | 1 | 7y1 |
| .........................................................auugaaagucagcccucgUcaca................................. | 1 | 1 | 7y1 |
| .........................................................Uuugaaagucagcccucgacaca................................. | 1 | 1 | 7y1 |
| .........................................................auugaaagucagccAucgacaca................................. | 2 | 1 | 7y1 |
| .........................................................auugaaagucagcccAcgacacaag............................... | 1 | 1 | 7y1 |
| .........................................................auuCaaagucagcccucgacacaag............................... | 1 | 1 | 7y1 |
| .........................................................auugaaagucagccAucgacacaag............................... | 2 | 1 | 7y1 |
| .........................................................Uuugaaagucagcccucgacacaag............................... | 1 | 1 | 7y1 |
| .........................................................auugaaagucagcccucgCcacaag............................... | 1 | 1 | 7y1 |
| .........................................................auugaaaguAagcccucgacacaag............................... | 1 | 1 | 7y1 |
| .........................................................auugaaagucUgcccucgacacaag............................... | 1 | 1 | 7y1 |
| .........................................................auugaaaCucagcccucgacacaag............................... | 1 | 1 | 7y1 |
| .........................................................auugaaagucagcccucgacacaag............................... | 209 | 0 | 7y1 |
| .........................................................auugaaagucagcccucgacacaaC............................... | 1 | 1 | 7y1 |
| .........................................................Guugaaagucagcccucgacacaag............................... | 2 | 1 | 7y1 |
| .........................................................auugaaagucagcccucCacacaag............................... | 1 | 1 | 7y1 |
| .........................................................auugaaagucagcccucgacacaaggguu........................... | 1 | 0 | 7y1 |
| .........................................................auugaaaUucagcccucgacacaaggguuug......................... | 1 | 1 | 7y1 |
| .........................................................auugaaagucagccAucgacacaaggguuug......................... | 1 | 1 | 7y1 |
| .........................................................auugaaagucagcccucgacacaaggguAug......................... | 1 | 1 | 7y1 |
| .........................................................auugaaagucagcccuAgacacaaggguuug......................... | 1 | 1 | 7y1 |
| .........................................................auugaaagucagcccucgacacaaggguuAg......................... | 2 | 1 | 7y1 |
| .........................................................auugaaagucagcccAcgacacaaggguuug......................... | 1 | 1 | 7y1 |
| .........................................................auugaaagucagcccucgacacaaggguuug......................... | 187 | 0 | 7y1 |
| .........................................................auugaaagucagcccucgacacaagggGuug......................... | 2 | 1 | 7y1 |
| .........................................................auugaaagucagAccucgacacaaggguuug......................... | 2 | 1 | 7y1 |
| .........................................................auugaaagucagcccucgacGcaaggguuugu........................ | 3 | 1 | 7y1 |
| .........................................................auugaaagucagcccucgacaGaaggguuugu........................ | 1 | 1 | 7y1 |
| .........................................................auugaaagucagcccucgacacaaCgguuugu........................ | 2 | 1 | 7y1 |
| .........................................................auugaaagucagcccucUacacaaggguuugu........................ | 1 | 1 | 7y1 |
| .........................................................auugaaagucagccGucgacacaaggguuugu........................ | 1 | 1 | 7y1 |
| .........................................................auugaaagucaUcccucgacacaaggguuugu........................ | 4 | 1 | 7y1 |
| .........................................................auugaGagucagcccucgacacaaggguuugu........................ | 2 | 1 | 7y1 |
| .........................................................auugaaagucagcccucgacacaagCguuugu........................ | 11 | 1 | 7y1 |
| .........................................................auugaaagucagcUcucgacacaaggguuugu........................ | 3 | 1 | 7y1 |
| .........................................................Nuugaaagucagcccucgacacaaggguuugu........................ | 4 | 1 | 7y1 |
| .........................................................auugaaagAcagcccucgacacaaggguuugu........................ | 6 | 1 | 7y1 |
| .........................................................auugaaagucagcccucgacacaaggguuugG........................ | 12 | 1 | 7y1 |
| .........................................................auugaaaguUagcccucgacacaaggguuugu........................ | 2 | 1 | 7y1 |
| .........................................................auugaaagucagcccucgacacaGggguuugu........................ | 2 | 1 | 7y1 |
| .........................................................auugaaagucagcccucgacacaaggguGugu........................ | 5 | 1 | 7y1 |
| .........................................................auugaaagucCgcccucgacacaaggguuugu........................ | 2 | 1 | 7y1 |
| .........................................................aAugaaagucagcccucgacacaaggguuugu........................ | 23 | 1 | 7y1 |
| .........................................................auugaaagucagcccucgaAacaaggguuugu........................ | 2 | 1 | 7y1 |
| .........................................................auugaaagucagcccucgaUacaaggguuugu........................ | 211 | 1 | 7y1 |
| .........................................................auuAaaagucagcccucgacacaaggguuugu........................ | 1 | 1 | 7y1 |
| .........................................................auugaaagucagcccuAgacacaaggguuugu........................ | 9 | 1 | 7y1 |
| .........................................................auugaaagucagcccucgacaAaaggguuugu........................ | 2 | 1 | 7y1 |
| .........................................................Guugaaagucagcccucgacacaaggguuugu........................ | 6 | 1 | 7y1 |
| .........................................................auugaaagucagcccucgacacaaggguuugC........................ | 1 | 1 | 7y1 |

**Star** **Mature**

gaccugcuucugggucgggguuucguacguagcagagcagcucccucgcugcgaucuauugaaagucagcccucgacacaaggguuuguccgcgcgcgcgcgcgcgugcgugc

| | | | |
|---|---|---|---|
| .........................................................auugGaagucagcccucgacacaaggguuugu........................ | 1 | 1 | 7y1 |
| .........................................................auugaaagucagcccucgacacUaggguuugu........................ | 1 | 1 | 7y1 |
| .........................................................auugaaagucagcccucgaGacaaggguuugu........................ | 2 | 1 | 7y1 |
| .........................................................auuUaaagucagcccucgacacaaggguuugu........................ | 1 | 1 | 7y1 |
| .........................................................auugaaagucagcccucgCcacaaggguuugu........................ | 19 | 1 | 7y1 |
| .........................................................Uuugaaagucagcccucgacacaaggguuugu........................ | 8 | 1 | 7y1 |
| .........................................................auAgaaagucagcccucgacacaaggguuugu........................ | 4 | 1 | 7y1 |
| .........................................................auugaaagucagcccucgacacaaggUuuugu........................ | 3 | 1 | 7y1 |
| .........................................................auugaaagucagcccucgacacaaggguuAgu........................ | 10 | 1 | 7y1 |
| .........................................................auugaaagucagcccucgacacaaggguuugA........................ | 19 | 1 | 7y1 |
| .........................................................auugaaagucagcccucgacacaagggAuugu........................ | 28 | 1 | 7y1 |
| .........................................................auugaaagucagcccucgacacCaggguuugu........................ | 1 | 1 | 7y1 |
| .........................................................auugaaagucagcccucgacacaaggguuuCu........................ | 11 | 1 | 7y1 |
| .........................................................auugaaagucagcccucgacacaaUgguuugu........................ | 5 | 1 | 7y1 |
| .........................................................auugaaagucagcccucgacCcaaggguuugu........................ | 1 | 1 | 7y1 |
| .........................................................auugaaagucagccUucgacacaaggguuugu........................ | 1 | 1 | 7y1 |
| .........................................................auugaaagCcagcccucgacacaaggguuugu........................ | 1 | 1 | 7y1 |
| .........................................................auugaaagucagcAcucgacacaaggguuugu........................ | 22 | 1 | 7y1 |
| .........................................................auugaaagucagAccucgacacaaggguuugu........................ | 16 | 1 | 7y1 |
| .........................................................auugaaagucagcccAcgacacaaggguuugu........................ | 13 | 1 | 7y1 |
| .........................................................auugaaagucagcccucgacacaaggguAugu........................ | 18 | 1 | 7y1 |
| .........................................................auugaaagucagcccucgacacaagggCuugu........................ | 1 | 1 | 7y1 |
| .........................................................auugaaagucagcccucgacacaaggAuuugu........................ | 1 | 1 | 7y1 |
| .........................................................auugaaagucagcccucgacacaaggguuugu........................ | 7347 | 0 | 7y1 |
| .........................................................auugaaaCucagcccucgacacaaggguuugu........................ | 4 | 1 | 7y1 |
| .........................................................auGgaaagucagcccucgacacaaggguuugu........................ | 1 | 1 | 7y1 |
| .........................................................auugCaagucagcccucgacacaaggguuugu........................ | 1 | 1 | 7y1 |
| .........................................................auugaaagucagcccucgacacaaggCuuugu........................ | 13 | 1 | 7y1 |
| .........................................................auugaaagucagcccucgacacaaggguuuUu........................ | 7 | 1 | 7y1 |
| .........................................................auugaaagucagcGcucgacacaaggguuugu........................ | 1 | 1 | 7y1 |
| .........................................................auuCaaagucagcccucgacacaaggguuugu........................ | 1 | 1 | 7y1 |
| .........................................................auugaaaguAagcccucgacacaaggguuugu........................ | 13 | 1 | 7y1 |
| .........................................................auugaaagucagcccucgUcacaaggguuugu........................ | 1 | 1 | 7y1 |
| .........................................................auugaaagucagcccucgacacaagUguuugu........................ | 3 | 1 | 7y1 |
| .........................................................auugaaagucGgcccucgacacaaggguuugu........................ | 1 | 1 | 7y1 |
| .........................................................auugaaagucaAcccucgacacaaggguuugu........................ | 1 | 1 | 7y1 |
| .........................................................auugaaagucagcccucgacacaagggGuugu........................ | 12 | 1 | 7y1 |
| .........................................................auugaaagucagcccucCacacaaggguuugu........................ | 2 | 1 | 7y1 |
| .........................................................auugaaagucUgcccucgacacaaggguuugu........................ | 1 | 1 | 7y1 |
| .........................................................auCgaaagucagcccucgacacaaggguuugu........................ | 1 | 1 | 7y1 |
| .........................................................auugaaagucagccAucgacacaaggguuugu........................ | 15 | 1 | 7y1 |
| .........................................................auugaaaguGagcccucgacacaaggguuugu........................ | 4 | 1 | 7y1 |
| .........................................................auugaaagucagcccucgacacGaggguuugu........................ | 2 | 1 | 7y1 |
| .........................................................aCugaaagucagcccucgacacaaggguuugu........................ | 1 | 1 | 7y1 |
| ..........................................................uugaaagucagcccucga..................................... | 208 | 0 | 7y1 |
| ..........................................................Augaaagucagcccucga..................................... | 4 | 1 | 7y1 |
| ..........................................................uugaaagucagccAucga..................................... | 3 | 1 | 7y1 |
| ..........................................................uAgaaagucagcccucga..................................... | 1 | 1 | 7y1 |
| ..........................................................uugaaaguAagcccucgac.................................... | 1 | 1 | 7y1 |
| ..........................................................Augaaagucagcccucgac.................................... | 4 | 1 | 7y1 |
| ..........................................................uugaaagucagcAcucgac.................................... | 1 | 1 | 7y1 |
| ..........................................................uuUaaagucagcccucgac.................................... | 1 | 1 | 7y1 |
| ..........................................................uugaaagucagcccucgac.................................... | 211 | 0 | 7y1 |
| ..........................................................uugaaagucagcAcucgacacaaggguuug......................... | 1 | 1 | 7y1 |
| ..........................................................uugaaagucagcccucgacacaagUguuug......................... | 1 | 1 | 7y1 |
| ..........................................................uugaaagucagcccuAgacacaaggguuug......................... | 1 | 1 | 7y1 |
| ..........................................................uugaaagucagcccucgacacaaggguuug......................... | 182 | 0 | 7y1 |
| ..........................................................uugaaagucagGccucgacacaaggguuug......................... | 1 | 1 | 7y1 |
| ..........................................................uugaaagucagccAucgacacaaggguuug......................... | 1 | 1 | 7y1 |
| ..........................................................uugaaaguAagcccucgacacaaggguuug......................... | 1 | 1 | 7y1 |
| ..........................................................uAgaaagucagcccucgacacaaggguuug......................... | 1 | 1 | 7y1 |
| ..........................................................uugaaagucagcccucgacacaaCgguuug......................... | 1 | 1 | 7y1 |
| ..........................................................uugaaagucagcccucgacUcaaggguuugu........................ | 1 | 1 | 7y1 |
| ..........................................................uugaaagucagcccucgacacaagggAuugu........................ | 6 | 1 | 7y1 |
| ..........................................................uugaaagucagAccucgacacaaggguuugu........................ | 2 | 1 | 7y1 |
| ..........................................................uugaaagucagcccucgacacaaggguuuCu........................ | 3 | 1 | 7y1 |
| ..........................................................uugaaagucagcccucgacacaaggguuugG........................ | 3 | 1 | 7y1 |
| ..........................................................uugaaagucagcccucgCcacaaggguuugu........................ | 4 | 1 | 7y1 |
| ..........................................................uugaaagAcagcccucgacacaaggguuugu........................ | 1 | 1 | 7y1 |
| ..........................................................uugaaagucagcccucgacacaaggguuAgu........................ | 6 | 1 | 7y1 |

```
     Star                                                Mature
gaccugcuucugggucgggguuucguacguagcagagcagcucccucgcugcgaucuauugaaagucagcccucgacacaaggguuuguccgcgcgcgcgcgcgcgugcgugc
..........................................................uugaaagucagcccucgacacaagCguuugu........................   1      1  7y1
..........................................................uugaaagucagcAcucgacacaaggguuugu........................   1      1  7y1
..........................................................Gugaaagucagcccucgacacaaggguuugu........................   3      1  7y1
..........................................................uugaaagucagccAucgacacaaggguuugu........................   3      1  7y1
..........................................................uugaaagucagcccucgacacaaggguuugu........................   1439   0  7y1
..........................................................uugaaagucagcccuAgacacaaggguuugu........................   2      1  7y1
..........................................................uugaaagucagcccucgacacaagUguuugu........................   1      1  7y1
..........................................................Augaaagucagcccucgacacaaggguuugu........................   10     1  7y1
..........................................................uugaaagucagcccucgacacaaggguuuUu........................   2      1  7y1
..........................................................uugaaagucagcccucgacacaaggCuuugu........................   1      1  7y1
..........................................................uugaaagucagGccucgacacaaggguuugu........................   1      1  7y1
..........................................................uugaaagucagcccucgacacaaUgguuugu........................   1      1  7y1
..........................................................uugaaagucagccUucgacacaaggguuugu........................   1      1  7y1
..........................................................uugaaagucagcccucgacacaaggguAugu........................   4      1  7y1
..........................................................uugaaagucGgcccucgacacaaggguuugu........................   1      1  7y1
..........................................................uuCaaagucagcccucgacacaaggguuugu........................   2      1  7y1
..........................................................uugaaagucagcccucgacacaaggguGugu........................   1      1  7y1
..........................................................uugaaaCucagcccucgacacaaggguuugu........................   1      1  7y1
..........................................................uugaaaguAagcccucgacacaaggguuugu........................   4      1  7y1
..........................................................uugaaagucagcccucgacacaaggguuugA........................   4      1  7y1
..........................................................uAgaaagucagcccucgacacaaggguuugu........................   2      1  7y1
..........................................................uugaaagucagcccucgacacaagggGuugu........................   3      1  7y1
..........................................................uugaaagucagcccucgacacaaCgguuugu........................   2      1  7y1
..........................................................uCgaaagucagcccucgacacaaggguuugu........................   2      1  7y1
...........................................................Agaaagucagcccucgac....................................   4      1  7y1
...........................................................ugaaagAcagcccucgac....................................   1      1  7y1
...........................................................ugaaaguAagcccucgac....................................   2      1  7y1
...........................................................ugaaagucagcccucgac....................................   191    0  7y1
...........................................................ugaaagucagUccucgac....................................   1      1  7y1
...........................................................ugaaagucagcccucgCc....................................   1      1  7y1
...........................................................ugaaagucagcccucCacacaaggguuug.........................   1      1  7y1
...........................................................ugaaagucagcccucgacacaagggAuug.........................   2      1  7y1
...........................................................ugaaagucagcccGcgacacaaggguuug.........................   1      1  7y1
...........................................................ugaaagAcagcccucgacacaaggguuug.........................   1      1  7y1
...........................................................ugaaagucagccGucgacacaaggguuug.........................   1      1  7y1
...........................................................ugaaagucagcccucgacacaaggUuuug.........................   1      1  7y1
...........................................................ugaaagucagcccucgCcacaaggguuug.........................   1      1  7y1
...........................................................ugaaagucagcccucgacacaaUgguuug.........................   1      1  7y1
...........................................................uAaaagucagcccucgacacaaggguuug.........................   1      1  7y1
...........................................................ugaaagucagcccucgacacGaggguuug.........................   1      1  7y1
...........................................................ugaaagucagcccucgacacaaggguuug.........................   462    0  7y1
...........................................................ugaaagucagAccucgacacaaggguuug.........................   1      1  7y1
...........................................................ugaaagucagcccucgacacaaggguAug.........................   1      1  7y1
...........................................................ugaaagucagcccucgacacaaggguGug.........................   1      1  7y1
...........................................................Ggaaagucagcccucgacacaaggguuug.........................   1      1  7y1
...........................................................ugaaagucUgcccucgacacaaggguuug.........................   1      1  7y1
...........................................................ugaaagucagcccucgacacaaggguuGg.........................   2      1  7y1
............................................................gaaagucagcccucgacaUaaggguuugu........................   1      1  7y1
............................................................gaaagucagcccucgacCcaaggguuugu........................   1      1  7y1
............................................................gaaagucagAccucgacacaaggguuugu........................   1      1  7y1
............................................................gaaagucagcccAcgacacaaggguuugu........................   1      1  7y1
............................................................gaaagucagcccucgacacaaggguuAgu........................   1      1  7y1
............................................................Caaagucagcccucgacacaaggguuugu........................   1      1  7y1
............................................................gaaagucagcccucgacacaaggguuugu........................   170    0  7y1
............................................................gaGagucagcccucgacacaaggguuugu........................   1      1  7y1
............................................................gaaagucagcccucgacacaagggGuugu........................   1      1  7y1
..............................................................aagucagcccucgacacaaggguuuC.........................   2      1  7y1
..............................................................aagucagcccucgacacaaggguuug.........................   211    0  7y1
..............................................................aagucagcccucgacUcaaggguuug.........................   2      1  7y1
..............................................................aagucagccAucgacacaaggguuug.........................   2      1  7y1
..............................................................aagucagcccucgacacaaggUuuug.........................   1      1  7y1
.................................................................uAagcccucgacacaaggguuug.........................   1      1  7y1
.................................................................ucagcccucgGcacaaggguuug.........................   1      1  7y1
.................................................................Acagcccucgacacaaggguuug.........................   3      1  7y1
.................................................................Ncagcccucgacacaaggguuug.........................   1      1  7y1
.................................................................ucagccAucgacacaaggguuug.........................   1      1  7y1
.................................................................ucagcccucgacacaaggguuug.........................   244    0  7y1
.................................................................ucagcccucgacacaagggAuug.........................   1      1  7y1
.................................................................Gcagcccucgacacaaggguuug.........................   1      1  7y1
..................................................................cagcUcucgacacaaggguuugu........................   211    1  7y1
```

**Star** **Mature**

```
gaccugcuucugggucgggguuucguacguagcagagcagcucccucgcugcgaucuauugaaagucagcccucgacacaaggguuuguccgcgcgcgcgcgcgcgugcgugc
...................................................................agccUucgacacaaggguuug.........................   132    1    7y1
....................................................................gcAcucgacacaaggguuug.........................   1      1    7y1
....................................................................gccAucgacacaaggguuug.........................   1      1    7y1
....................................................................Ncccucgacacaaggguuug.........................   1      1    7y1
....................................................................gAccucgacacaaggguuug.........................   2      1    7y1
....................................................................gcccucgaAacaaggguuug.........................   2      1    7y1
....................................................................gcccucgacacaaggguuug.........................   188    0    7y1
....................................................................gcccucAacacaaggguuug.........................   1      1    7y1
.....................................................................cccucgacacaaggguuuU.........................   1      1    7y1
.....................................................................ccAucgacacaaggguuugu........................   2      1    7y1
.....................................................................Nccucgacacaaggguuugu........................   1      1    7y1
.....................................................................cccucgacacaaggguuugu........................   289    0    7y1
.....................................................................cccucgacacaagggAuugu........................   2      1    7y1
.....................................................................cccucgacacaaggCuuugu........................   2      1    7y1
.....................................................................cccucgacaAaaggguuugu........................   1      1    7y1
.....................................................................cccucgacUcaaggguuugu........................   1      1    7y1
.....................................................................cccucgacacaaggguuugG........................   2      1    7y1
.....................................................................cccucgacacaaUgguuugu........................   1      1    7y1
......................................................................Gcucgacacaaggguuug.........................   4      1    7y1
......................................................................ccucgacacaagAguuug.........................   1      1    7y1
......................................................................ccucgacaUaaggguuug.........................   1      1    7y1
......................................................................ccucUacacaaggguuug.........................   3      1    7y1
......................................................................cAucgacacaaggguuug.........................   6      1    7y1
......................................................................ccucgacUcaaggguuug.........................   2      1    7y1
......................................................................ccCcgacacaaggguuug.........................   1      1    7y1
......................................................................ccucgacacaaggguAug.........................   4      1    7y1
......................................................................ccucgacacaaggguuGg.........................   1      1    7y1
......................................................................ccucgaAacaaggguuug.........................   3      1    7y1
......................................................................ccAcgacacaaggguuug.........................   1      1    7y1
......................................................................ccucgacacaagCguuug.........................   2      1    7y1
......................................................................ccucgacacaaUgguuug.........................   1      1    7y1
......................................................................Ncucgacacaaggguuug.........................   2      1    7y1
......................................................................ccuGgacacaaggguuug.........................   1      1    7y1
......................................................................Acucgacacaaggguuug.........................   8      1    7y1
......................................................................ccucgacacaaggguuAg.........................   1      1    7y1
......................................................................ccucgacacaaggguuuA.........................   1      1    7y1
......................................................................ccucgacacaaggguuuC.........................   1      1    7y1
......................................................................ccucgacacaUggguuug.........................   1      1    7y1
......................................................................ccucgacacaaggCuuug.........................   1      1    7y1
......................................................................ccucgacacaaggguuug.........................   1954   0    7y1
......................................................................ccucgCcacaaggguuug.........................   2      1    7y1
......................................................................ccucgacacGaggguuug.........................   1      1    7y1
......................................................................ccucgacacaaUgguuugu........................   5      1    7y1
......................................................................ccucgacacNaggguuugu........................   1      1    7y1
......................................................................ccucgacacaaggguAugu........................   8      1    7y1
......................................................................ccucgacacaGggguuugu........................   1      1    7y1
......................................................................ccucUacacaaggguuugu........................   7      1    7y1
......................................................................ccucgacacaaggguuuAu........................   1      1    7y1
......................................................................ccucgacacaaggguuugA........................   12     1    7y1
......................................................................ccucgacacaagUguuugu........................   6      1    7y1
......................................................................ccucgUcacaaggguuugu........................   1      1    7y1
......................................................................ccucgacaUaaggguuugu........................   153    1    7y1
......................................................................Ucucgacacaaggguuugu........................   358    1    7y1
......................................................................Acucgacacaaggguuugu........................   24     1    7y1
......................................................................ccucgacacaaggguuuCu........................   3      1    7y1
......................................................................ccucgacGcaaggguuugu........................   3      1    7y1
......................................................................ccucgacacaaggUuuugu........................   9      1    7y1
......................................................................cAucgacacaaggguuugu........................   17     1    7y1
......................................................................ccucgacacaaCgguuugu........................   3      1    7y1
......................................................................ccucgacacaaggCuuugu........................   13     1    7y1
......................................................................ccucgCcacaaggguuugu........................   7      1    7y1
......................................................................ccucgacacaaggguuugG........................   17     1    7y1
......................................................................ccuGgacacaaggguuugu........................   1      1    7y1
......................................................................Ncucgacacaaggguuugu........................   3      1    7y1
......................................................................ccucgacacaagCguuugu........................   4      1    7y1
......................................................................ccucgaUacaaggguuugu........................   1      1    7y1
......................................................................ccucgacacaaggguuugC........................   1      1    7y1
......................................................................ccAcgacacaaggguuugu........................   14     1    7y1
......................................................................ccucgaAacaaggguuugu........................   9      1    7y1
......................................................................ccucgacaAaaggguuugu........................   8      1    7y1
```

**Star** **Mature**

```
gaccugcuucugggucgggguuucguacguagcagagcagcucccucgcugcgaucuauugaaagucagcccucgacacaaggguuuguccgcgcgcgcgcgcgcgugcgugc
.....................................................................ccucgacacGaggguuugu........................ 1 1 7y1
.....................................................................ccucgacacaaggguuugu........................ 6344 0 7y1
.....................................................................ccucgacacaaggguuuUu........................ 5 1 7y1
.....................................................................ccucgacacaagggGuugu........................ 4 1 7y1
.....................................................................ccucgGcacaaggguuugu........................ 1 1 7y1
.....................................................................Gcucgacacaaggguuugu........................ 5 1 7y1
.....................................................................ccucAacacaaggguuugu........................ 4 1 7y1
.....................................................................ccucgacacaagggAuugu........................ 8 1 7y1
.....................................................................ccucgacacaaggguuAgu........................ 9 1 7y1
.....................................................................ccucgacacaaggguCugu........................ 1 1 7y1
.....................................................................cNucgacacaaggguuuguccgcgc.................. 1 1 7y1
.....................................................................ccucgacacaaggguuuguccgcgA.................. 1 1 7y1
.....................................................................ccAcgacacaaggguuuguccgcgc.................. 1 1 7y1
.....................................................................ccucgacacaaggguuuguccgcgc.................. 277 0 7y1
.....................................................................ccucgacacaaggguAuguccgcgc.................. 1 1 7y1
.....................................................................ccucgacacaaggCuuuguccgcgc.................. 1 1 7y1
.....................................................................ccucgacacaaggUuuuguccgcgc.................. 1 1 7y1
.....................................................................ccucgacacaagggAuuguccgcgc.................. 1 1 7y1
.....................................................................ccuUgacacaaggguuuguccgcgc.................. 1 1 7y1
.....................................................................cAucgacacaaggguuuguccgcgc.................. 1 1 7y1
.......................................................................cucgacacaaggguuAgu........................ 30 1 7y1
.......................................................................cucgacacGaggguuugu........................ 5 1 7y1
.......................................................................cucgacacaagggGuugu........................ 22 1 7y1
.......................................................................cucgacacaaggguCugu........................ 2 1 7y1
.......................................................................Gucgacacaaggguuugu........................ 18 1 7y1
.......................................................................cNcgacacaaggguuugu........................ 2 1 7y1
.......................................................................cucAacacaaggguuugu........................ 13 1 7y1
.......................................................................Nucgacacaaggguuugu........................ 10 1 7y1
.......................................................................cucgacUcaaggguuugu........................ 9 1 7y1
.......................................................................cucgacacaaggguuugA........................ 39 1 7y1
.......................................................................cucUacacaaggguuugu........................ 14 1 7y1
.......................................................................cucgacacaaggguuCgu........................ 1 1 7y1
.......................................................................cucgacacaaggguuuUu........................ 19 1 7y1
.......................................................................cucgaUacaaggguuugu........................ 3 1 7y1
.......................................................................cucgacacaaggguuugu........................ 25979 0 7y1
.......................................................................cuGgacacaaggguuugu........................ 197 1 7y1
.......................................................................cucgacacaaggCuuugu........................ 17 1 7y1
.......................................................................cucgacacaagCguuugu........................ 18 1 7y1
.......................................................................cucgacaUaaggguuugu........................ 3 1 7y1
.......................................................................cucgacacaagUguuugu........................ 175 1 7y1
.......................................................................cucgacacaaggguuugC........................ 1 1 7y1
.......................................................................cucgacacaaCgguuugu........................ 17 1 7y1
.......................................................................cucgacGcaaggguuugu........................ 6 1 7y1
.......................................................................cucgacacaGggguuugu........................ 1 1 7y1
.......................................................................cucgacacaaAgguuugu........................ 197 1 7y1
.......................................................................cucgacacaaggguuugG........................ 34 1 7y1
.......................................................................cucgacacaaggguuGgu........................ 1 1 7y1
.......................................................................cucgacacaaUgguuugu........................ 17 1 7y1
.......................................................................cucgaAacaaggguuugu........................ 34 1 7y1
.......................................................................cucgacCcaaggguuugu........................ 1 1 7y1
.......................................................................cucgacacCaggguuugu........................ 1 1 7y1
.......................................................................cAcgacacaaggguuugu........................ 75 1 7y1
.......................................................................cucgacacaaggguuuCu........................ 23 1 7y1
.......................................................................cucgaNacaaggguuugu........................ 1 1 7y1
.......................................................................cGcgacacaaggguuugu........................ 2 1 7y1
.......................................................................cucgacacaaggguuuAu........................ 7 1 7y1
.......................................................................cucgacacaaggguGugu........................ 5 1 7y1
.......................................................................cucgacacaaggguAugu........................ 35 1 7y1
.......................................................................cCcgacacaaggguuugu........................ 2 1 7y1
.......................................................................cucgacacaagggAuugu........................ 43 1 7y1
.......................................................................cucgacacaaggUuuugu........................ 13 1 7y1
.......................................................................cucgacacaaggAuuugu........................ 3 1 7y1
.......................................................................cucgacacUaggguuugu........................ 2 1 7y1
.......................................................................cucgaGacaaggguuugu........................ 2 1 7y1
.......................................................................cucgCcacaaggguuugu........................ 4 1 7y1
.......................................................................cucgacaAaaggguuugu........................ 21 1 7y1
.......................................................................cucgacacaUggguuugu........................ 2 1 7y1
.......................................................................cucgacaGaaggguuugu........................ 1 1 7y1
.......................................................................cucgUcacaaggguuugu........................ 2 1 7y1
.......................................................................cucgacacaaggguuuguccgcgc.................. 1 0 7y1
```

Star Mature

gaccugcuucugggucggggguuucguacguagcagagcagcucccucgcugcgaucuauugaaagucagcccucgacacaaggguuuguccgcgcgcgcgcgcgcgugcgugc
