# Supplementary material for: SEAweb: the small RNA Expression Atlas web application
Source: Nucleic Acids Res. 2019 Oct 10;48(D1):D204–19. doi: 10.1093/nar/gkz869 (PMC6943056; doi:10.1093/nar/gkz869)
Supplement: gkz869_Supplemental_Files [file gkz869_supplemental_files.zip › p-hsa-miR-247.pdf]

Provisional ID	:	chr14_823
Score total	:	510487.8
Score for star read(s)	:	3.9
Score for read counts	:	510477.3
Score for mfe	:	2
Score for randfold	:	1.6
Score for cons. seed	:	3
Total read count	:	1001291
Mature read count	:	1001289
Loop read count	:	1
Star read count	:	1

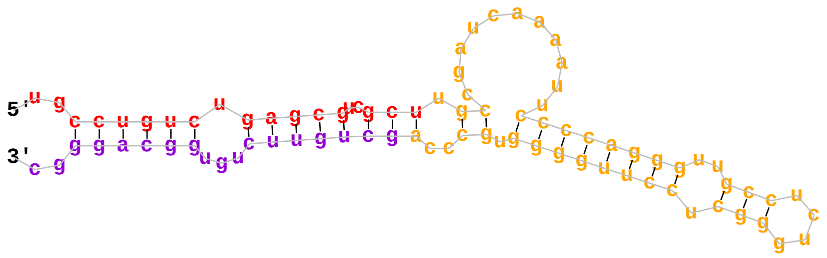

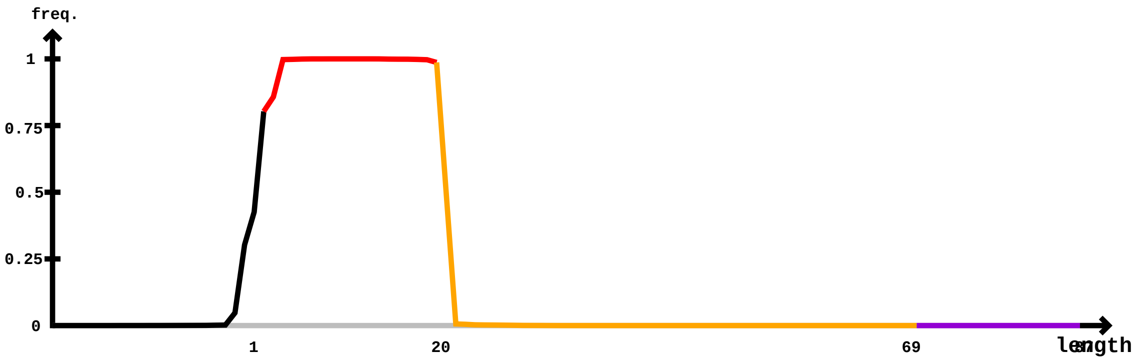

Mature **Star**

```
5'- cuggguuccucccagggcuaugccugucugagcgucgcuugccgaucaaaauuccccaggguugccucugggcuccuuggggugcccagcuguucuguggcagggccc  -3'  obs
    cuggguuccucccagggcuaugccugucugagcgucgcuugccgaucaaaauuccccaggguugccucugggcuccuuggggugcccagcuguucuguggcagggccc       exp
    (((((.....)))))((((.((((.....(((((..(((.((...........((((((((..(((....))).)))))))).))..))))))))...)))).)))).   reads   mm   sample
    ..ggguuccucccGgggcua........................................................................................   2   1   mv7
    ....guuccucccGgggcuaug......................................................................................   1   1   mv7
    ....guuccucccGgggcuaugccugu.................................................................................   1   1   mv7
    ....guuccucccGgggcuaugccuguc................................................................................   1   1   mv7
    ....guuccucccGgggcuaugccugucug..............................................................................   1   1   mv7
    ....guuccucccagggcuaCgccugucugagc...........................................................................   2   1   mv7
    ....guuccucccagggcuaCgccugucugagcguc........................................................................   1   1   mv7
    .....uuccucccGgggcuaugccugucuga.............................................................................   1   1   mv7
    .....uuccucccagggcuaCgccugucugag............................................................................   1   1   mv7
    .....uuccucccagggcuaCgccugucugagc...........................................................................   9   1   mv7
    .....uuccucccGgggcuaugccugucugagc...........................................................................   20  1   mv7
    .....uuccucccGgggcuaugccugucugagcg..........................................................................   1   1   mv7
    .....uuccucccagggcuaCgccugucugagcgu.........................................................................   2   1   mv7
    .....uuccucccagggcuaCgccugucugagcguc........................................................................   2   1   mv7
    .....uuccucccGgggcuaugccugucugagcguc........................................................................   3   1   mv7
    .....uuccucccGgggcuaugccugucugagcgucg.......................................................................   4   1   mv7
    .....uuccucccagggcuaCgccugucugagcgucg.......................................................................   2   1   mv7
    ......uccucccGgggcuaugccugucug..............................................................................   1   1   mv7
    ......uccucccagggcuaCgccugucugag............................................................................   1   1   mv7
    ......uccucccGgggcuaugccugucugag............................................................................   1   1   mv7
    ......uccucccGgggcuaugccugucugagc...........................................................................   14  1   mv7
    ......uccucccagggcuaCgccugucugagc...........................................................................   5   1   mv7
    ......uccucccGgggcuaugccugucugagcg..........................................................................   1   1   mv7
    ......uccucccagggcuaCgccugucugagcgu.........................................................................   1   1   mv7
    ......uccucccGgggcuaugccugucugagcgu.........................................................................   1   1   mv7
    ......uccucccGgggcuaugccugucugagcguc........................................................................   5   1   mv7
    ......uccucccagggcuaCgccugucugagcgucgc......................................................................   2   1   mv7
    ......uccucccGgggcuaugccugucugagcgucgc......................................................................   1   1   mv7
    .......ccucccagggcuaCgccugucuga.............................................................................   2   1   mv7
    .......ccucccGgggcuaugccugucugag............................................................................   1   1   mv7
    .......ccucccagggcuaCgccugucugagc...........................................................................   2   1   mv7
    .......ccucccGgggcuaugccugucugagc...........................................................................   2   1   mv7
    .......ccucccagggcuaCgccugucugagcgu.........................................................................   2   1   mv7
```

```
                    Mature                                                          Star
cuggguuccucccagggcuaugccugucugagcgucgcuugccgaucaaaauuccccaggguugccucugggcuccuuggggugcccagcuguucuguggcagggccc
.......ccucccagggcuaCgccugucugagcguc........................................................................  2   1  mv7
.......ccucccGgggcuaugccugucugagcgucgcu.....................................................................  43  1  mv7
.......ccucccagggcuaCgccugucugagcgucgcu.....................................................................  7   1  mv7
........cucccGgggcuaugccugu.................................................................................  1   1  mv7
........cucccGgggcuaugccugucugagc...........................................................................  2   1  mv7
........cucccagggcuaCgccugucugagcgu.........................................................................  1   1  mv7
........cucccGgggcuaugccugucugagcgucgcu.....................................................................  41  1  mv7
........cucccCgggcuaugccugucugagcgucgcu.....................................................................  1   1  mv7
........cucccagggcuaCgccugucugagcgucgcu.....................................................................  6   1  mv7
........cucccagggcuaCgccugucugagcgucgcuu....................................................................  1   1  mv7
.........ucccGgggcuaugccugucugag............................................................................  1   1  mv7
.........ucccGgggcuaugccugucugagc...........................................................................  1   1  mv7
.........ucccagggcuaCgccugucugagc...........................................................................  1   1  mv7
.........ucccGgggcuaugccugucugagcgu.........................................................................  1   1  mv7
.........ucccagggcuaCgccugucugagcgu.........................................................................  1   1  mv7
.........ucccGgggcuaugccugucugagcgucg.......................................................................  2   1  mv7
.........ucccagggcuaCgccugucugagcgucgc......................................................................  1   1  mv7
.........ucccGgggcuaugccugucugagcgucgc......................................................................  1   1  mv7
.........ucccGgggcuaugccugucugagcgucgcu.....................................................................  19  1  mv7
.........ucccagggcuaCgccugucugagcgucgcu.....................................................................  2   1  mv7
.........ucccGgggcuaugccugucugagcgucgcuu....................................................................  1   1  mv7
..........cccGgggcuaugccugucuga.............................................................................  1   1  mv7
..........cccGgggcuaugccugucugag............................................................................  2   1  mv7
..........cccagggcuaCgccugucugag............................................................................  5   1  mv7
..........cccagggcuaCgccugucugagc...........................................................................  10  1  mv7
..........cccGgggcuaugccugucugagc...........................................................................  6   1  mv7
..........cccagggcuaCgccugucugagcgu.........................................................................  8   1  mv7
..........cccGgggcuaugccugucugagcgu.........................................................................  1   1  mv7
..........cccGgggcuaugccugucugagcguc........................................................................  5   1  mv7
..........cccagggcuaCgccugucugagcguc........................................................................  8   1  mv7
..........cccagggcuaCgccugucugagcgucg.......................................................................  7   1  mv7
..........cccagggcuaugccugucAgagcgucg.......................................................................  1   1  mv7
..........cccagggcuaCgccugucugagcgucgc......................................................................  1   1  mv7
..........cccGgggcuaugccugucugagcgucgcu.....................................................................  18  1  mv7
..........cccCgggcuaugccugucugagcgucgcu.....................................................................  1   1  mv7
..........cccagggcuaCgccugucugagcgucgcu.....................................................................  29  1  mv7
..........cccGgggcuaugccugucugagcgucgcuu....................................................................  6   1  mv7
...........ccGgggcuaugccugucu...............................................................................  2   1  mv7
...........ccagggcuaCgccugucugag............................................................................  2   1  mv7
...........ccGgggcuaugccugucugagc...........................................................................  1   1  mv7
...........ccagggcuaCgccugucugagc...........................................................................  3   1  mv7
...........ccagggcuaCgccugucugagcg..........................................................................  8   1  mv7
...........ccagggcuaCgccugucugagcgu.........................................................................  5   1  mv7
...........ccUgggcuaugccugucugagcgu.........................................................................  1   1  mv7
...........ccGgggcuaugccugucugagcgu.........................................................................  4   1  mv7
...........ccagggcuaCgccugucugagcguc........................................................................  3   1  mv7
...........ccGgggcuaugccugucugagcguc........................................................................  1   1  mv7
...........ccagggcuaCgccugucugagcgucg.......................................................................  1   1  mv7
...........ccGgggcuaugccugucugagcgucg.......................................................................  2   1  mv7
...........ccagggcuaCgccugucugagcgucgc......................................................................  6   1  mv7
...........ccagggcuaCgccugucugagcgucgcu.....................................................................  25  1  mv7
...........ccCgggcuaugccugucugagcgucgcu.....................................................................  1   1  mv7
...........ccGgggcuaugccugucugagcgucgcu.....................................................................  22  1  mv7
...........Ucagggcuaugccugucugagcgucgcu.....................................................................  1   1  mv7
...........ccagggcuaugccugucugagcgucgcuC....................................................................  1   1  mv7
...........ccagggcuaCgccugucugagcgucgcuu....................................................................  2   1  mv7
...........ccGgggcuaugccugucugagcgucgcuu....................................................................  8   1  mv7
............cagggcuaCgccugucugag............................................................................  22  1  mv7
............cGgggcuaugccugucugag............................................................................  16  1  mv7
............cGgggcuaugccugucugagc...........................................................................  5   1  mv7
............cagggcuaCgccugucugagc...........................................................................  2   1  mv7
............cGgggcuaugccugucugagcg..........................................................................  8   1  mv7
............cagggcuaCgccugucugagcg..........................................................................  7   1  mv7
............cagggcuaCgccugucugagcgu.........................................................................  2   1  mv7
............cGgggcuaugccugucugagcgu.........................................................................  2   1  mv7
............cUgggcuaugccugucugagcgu.........................................................................  1   1  mv7
............cagggcuaCgccugucugagcguc........................................................................  1   1  mv7
............cagggcuaCgccugucugagcgucg.......................................................................  1   1  mv7
............cGgggcuaugccugucugagcgucg.......................................................................  1   1  mv7
............cGgggcuaugccugucugagcgucgc......................................................................  2   1  mv7
```

Mature Star

```
cuggguuccucccagggcuaugccugucugagcgucgcuugccgaucaaaauuccccaggguugccucugggcuccuuggggugcccagcuguucuguggcagggccc
............cGgggcuaugccugucugagcgucgcu.....................................................................  21    1  mv7
............cagggcuaCgccugucugagcgucgcu.....................................................................  12    1  mv7
............cGgggcuaugccugucugagcgucgcuu....................................................................  8     1  mv7
............cCgggcuaugccugucugagcgucgcuu....................................................................  1     1  mv7
.............Ggggcuaugccugucuga.............................................................................  1     1  mv7
.............agggcuaCgccugucuga.............................................................................  1     1  mv7
.............agggcuaCgccugucugag............................................................................  6     1  mv7
.............Ggggcuaugccugucugag............................................................................  1     1  mv7
.............agggcuaAgccugucugagc...........................................................................  1     1  mv7
.............Ggggcuaugccugucugagc...........................................................................  4     1  mv7
.............agggcuaCgccugucugagc...........................................................................  5     1  mv7
.............Ggggcuaugccugucugagcg..........................................................................  1     1  mv7
.............agggcuaCgccugucugagcg..........................................................................  1     1  mv7
.............agggcuaCgccugucugagcgu.........................................................................  3     1  mv7
.............agggcuaCgccugucugagcgucgcu.....................................................................  1     1  mv7
.............Ggggcuaugccugucugagcgucgcu.....................................................................  15    1  mv7
..............gggcuaCgccugucugag............................................................................  23    1  mv7
..............gggcuaAgccugucugag............................................................................  1     1  mv7
..............gggcuaCgccugucugagc...........................................................................  43    1  mv7
..............gggcuaCgccugucugagcg..........................................................................  16    1  mv7
..............gggcuaCgccugucugagcgu.........................................................................  15    1  mv7
..............gggcuaCgccugucugagcguc........................................................................  9     1  mv7
..............gggcuaCgccugucugagcgucg.......................................................................  13    1  mv7
..............gggcuaCgccugucugagcgucgc......................................................................  1     1  mv7
..............gggcuaugccugucugagcguUgcu.....................................................................  1     1  mv7
..............gggcuaugccugucugagcgucgcu.....................................................................  4     0  mv7
..............gggcuaCgccugucugagcgucgcu.....................................................................  49    1  mv7
..............gggcuaCgccugucugagcgucgcuu....................................................................  12    1  mv7
...............ggcuaCgccugucugagc...........................................................................  575   1  mv7
...............ggcuaGgccugucugagc...........................................................................  1     1  mv7
...............ggcuaugccugucugagU...........................................................................  1     1  mv7
...............ggcuaugccugucugagc...........................................................................  2     0  mv7
...............ggcuaugccugucGgagc...........................................................................  1     1  mv7
...............ggcuaCgccugucugagcg..........................................................................  28    1  mv7
...............ggcuaCgccugucugagcgu.........................................................................  8     1  mv7
...............ggcuaCgccugucugagcguc........................................................................  11    1  mv7
...............ggcuaCgccugucugagcgucg.......................................................................  13    1  mv7
...............ggcuaCgccugucugagcgucgc......................................................................  5     1  mv7
...............ggcuaCgccugucugagcgucgcu.....................................................................  88    1  mv7
...............ggcuaCgccugucugagcgucgcuu....................................................................  8     1  mv7
...............ggcuaugccugucugagcgucgcuu....................................................................  2     0  mv7
................gcuaugccugucugagcA..........................................................................  1     1  mv7
................gcuaCgccugucugagcg..........................................................................  218   1  mv7
................gcuaCgccugucugagcgu.........................................................................  29    1  mv7
................gcuaCgccugucugagcguc........................................................................  33    1  mv7
................gcuaCgccugucugagcgucg.......................................................................  3     1  mv7
................gcuaCgccugucugagcgucgc......................................................................  1     1  mv7
................gcuaugccugucugagcgucgcC.....................................................................  2     1  mv7
................gcuaugccugucugagcgucgcu.....................................................................  13    0  mv7
................Ucuaugccugucugagcgucgcu.....................................................................  1     1  mv7
................gcuaCgccugucugagcgucgcu.....................................................................  156   1  mv7
................gcuaugccCgucugagcgucgcu.....................................................................  1     1  mv7
................gcuaCgccugucugagcgucgcuu....................................................................  10    1  mv7
................gcuaCgccugucugagcgucgcuugc..................................................................  2     1  mv7
................gcuaCgccugucugagcgucgcuugccg................................................................  1     1  mv7
.................cuaAgccugucugagcgu.........................................................................  2     1  mv7
.................cuaugccugucugagcgu.........................................................................  2     0  mv7
.................cuaCgccugucugagcgu.........................................................................  76    1  mv7
.................cuaCgccugucugagcguc........................................................................  132   1  mv7
.................cuaAgccugucugagcguc........................................................................  2     1  mv7
.................cuauAccugucugagcguc........................................................................  1     1  mv7
.................Guaugccugucugagcguc........................................................................  1     1  mv7
.................cuaCgccugucugagcgucg.......................................................................  16    1  mv7
.................cuaAgccugucugagcgucg.......................................................................  1     1  mv7
.................cuaugccugucugagcgucgc......................................................................  1     0  mv7
.................cuaugccugucugagcgCcgc......................................................................  1     1  mv7
.................cuaGgccugucugagcgucgc......................................................................  1     1  mv7
.................cuaCgccugucugagcgucgc......................................................................  327   1  mv7
.................cuaAgccugucugagcgucgc......................................................................  5     1  mv7
.................cuaAgccugucugagcgucgcu.....................................................................  1051  1  mv7
```

```
                    Mature                                                                   Star
cuggguuccucccagggcuaugccugucugagcgucgcuugccgaucaaaauuccccaggguugccucugggcuccuuggggugcccagcuguucuguggcagggccc
.................cuaGgccugucugagcgucgcu.....................................................................	85	1	mv7
.................cuaugAcugucugagcgucgcu.....................................................................	1	1	mv7
.................Guaugccugucugagcgucgcu.....................................................................	1	1	mv7
.................cuaugccugucuUagcgucgcu.....................................................................	1	1	mv7
.................cuaugccugucugagcgucgcu.....................................................................	156	0	mv7
.................cuaugccuAucugagcgucgcu.....................................................................	1	1	mv7
.................cAaugccugucugagcgucgcu.....................................................................	1	1	mv7
.................cuaugccuguUugagcgucgcu.....................................................................	1	1	mv7
.................cuaugccugucugagcgucgcC.....................................................................	18	1	mv7
.................cCaugccugucugagcgucgcu.....................................................................	1	1	mv7
.................cuaugccugAcugagcgucgcu.....................................................................	1	1	mv7
.................cuaugccugucugagcgucgcG.....................................................................	4	1	mv7
.................Uuaugccugucugagcgucgcu.....................................................................	8	1	mv7
.................cuaugccugucugGgcgucgcu.....................................................................	1	1	mv7
.................cuGugccugucugagcgucgcu.....................................................................	2	1	mv7
.................cuaugccugCcugagcgucgcu.....................................................................	1	1	mv7
.................cuaCgccugucugagcgucgcu.....................................................................	41664	1	mv7
.................Auaugccugucugagcgucgcu.....................................................................	10	1	mv7
.................cuaugccugucugagcgucgcA.....................................................................	6	1	mv7
.................cuaugccugucugagUgucgcu.....................................................................	1	1	mv7
.................cuaAgccugucugagcgucgcuu....................................................................	83	1	mv7
.................cuaugccugucugagcguUgcuu....................................................................	1	1	mv7
.................cuaugccugucAgagcgucgcuu....................................................................	1	1	mv7
.................cCaugccugucugagcgucgcuu....................................................................	1	1	mv7
.................cuaugccugucugagcgucgcuC....................................................................	3	1	mv7
.................Auaugccugucugagcgucgcuu....................................................................	1	1	mv7
.................cuaugccugucugagcgucgcuu....................................................................	27	0	mv7
.................cuaugccugucugagcgucgcuA....................................................................	1	1	mv7
.................cuaugccugucugagcgucgcCu....................................................................	1	1	mv7
.................cuaugccugucugagcgCcgcuu....................................................................	1	1	mv7
.................cuaugccugucugagcgucgcuG....................................................................	2	1	mv7
.................cuaCgccugucugagcgucgcuu....................................................................	4288	1	mv7
.................cuaugccuguAugagcgucgcuu....................................................................	1	1	mv7
.................cuaGgccugucugagcgucgcuu....................................................................	10	1	mv7
.................cuaCgccugucugagcgucgcuug...................................................................	9	1	mv7
.................cuaCgccugucugagcgucgcuugc..................................................................	48	1	mv7
.................cuaGgccugucugagcgucgcuugc..................................................................	1	1	mv7
.................cuaCgccugucugagcgucgcuugcc.................................................................	4	1	mv7
.................cuaCgccugucugagcgucgcuugccg................................................................	11	1	mv7
.................cuaAgccugucugagcgucgcuugccg................................................................	1	1	mv7
.................cuaCgccugucugagcgucgcuugccga...............................................................	16	1	mv7
.................cuaugccugucugagcgucgcuugccga...............................................................	1	0	mv7
.................cuaCgccugucugagcgucgcuugccgau..............................................................	15	1	mv7
.................cuaCgccugucugagcgucgcuugccgauc.............................................................	10	1	mv7
.................cuaAgccugucugagcgucgcuugccgauc.............................................................	1	1	mv7
.................cuaCgccugucugagcgucgcuugccgauca............................................................	7	1	mv7
.................cuaCgccugucugagcgucgcuugccgaucaa...........................................................	5	1	mv7
.................cuaAgccugucugagcgucgcuugccgaucaa...........................................................	1	1	mv7
..................uaCgccugucugagcguc........................................................................	624	1	mv7
..................uaugccugucugagcguc........................................................................	4	0	mv7
..................uaAgccugucugagcguc........................................................................	1	1	mv7
..................uaugccugucugagcgCc........................................................................	1	1	mv7
..................Caugccugucugagcguc........................................................................	1	1	mv7
..................uaGgccugucugagcguc........................................................................	9	1	mv7
..................uaugccugucugagcguU........................................................................	1	1	mv7
..................uaugccugucugGgcgucg.......................................................................	1	1	mv7
..................uaugccugucugagcgucg.......................................................................	5	0	mv7
..................uaGgccugucugagcgucg.......................................................................	1	1	mv7
..................uaAgccugucugagcgucg.......................................................................	1	1	mv7
..................uaCgccugucugagcgucg.......................................................................	563	1	mv7
..................uaGgccugucugagcgucgc......................................................................	10	1	mv7
..................uaugccugucugagcgucgU......................................................................	2	1	mv7
..................uaugccugucugagcgucgc......................................................................	4	0	mv7
..................uaCgccugucugagcgucgc......................................................................	1939	1	mv7
..................uaugcUugucugagcgucgc......................................................................	1	1	mv7
..................uaAgccugucugagcgucgc......................................................................	10	1	mv7
..................uaNgccugucugagcgucgc......................................................................	1	1	mv7
..................uaCgccugucugagcgucgcu.....................................................................	236239	1	mv7
..................uaugccugucugagGgucgcu.....................................................................	1	1	mv7
..................uaNgccugucugagcgucgcu.....................................................................	9	1	mv7
```

```
                 Mature                                                            Star
cuggguuccucccagggcuaugccugucugagcgucgcuugccgaucaaaauuccccaggguugccucugggcuccuuggggugcccagcuguucuguggcagggccc
..................uaugccugucugagcguUgcu.....................................................................   2      1   mv7
..................uauCccugucugagcgucgcu.....................................................................   9      1   mv7
..................uaugccugucugUgcgucgcu.....................................................................   2      1   mv7
..................uaugccugucugagcgucgcG.....................................................................   23     1   mv7
..................uaugccuAucugagcgucgcu.....................................................................   2      1   mv7
..................uaugcUugucugagcgucgcu.....................................................................   3      1   mv7
..................uaugccugucugagcgCcgcu.....................................................................   1      1   mv7
..................uaugccCgucugagcgucgcu.....................................................................   1      1   mv7
..................Aaugccugucugagcgucgcu.....................................................................   5      1   mv7
..................uaugccugucugagcUucgcu.....................................................................   2      1   mv7
..................uaugccugucugagcgucgUu.....................................................................   2      1   mv7
..................uaugccuguUugagcgucgcu.....................................................................   2      1   mv7
..................uaugccugucugagcAucgcu.....................................................................   2      1   mv7
..................uaugccugucCgagcgucgcu.....................................................................   1      1   mv7
..................uCugccugucugagcgucgcu.....................................................................   3      1   mv7
..................uaugccugucugagcgucgcu.....................................................................   739    0   mv7
..................uaugccugucugagcCucgcu.....................................................................   1      1   mv7
..................uauAccugucugagcgucgcu.....................................................................   9      1   mv7
..................uaugccGgucugagcgucgcu.....................................................................   1      1   mv7
..................uaugccugucugagcguAgcu.....................................................................   1      1   mv7
..................uaugccugucugagUgucgcu.....................................................................   3      1   mv7
..................uaAgccugucugagcgucgcu.....................................................................   653    1   mv7
..................uaGgccugucugagcgucgcu.....................................................................   1513   1   mv7
..................uaugccugucugCgcgucgcu.....................................................................   7      1   mv7
..................uaugccugucugagcgucUcu.....................................................................   2      1   mv7
..................Caugccugucugagcgucgcu.....................................................................   3      1   mv7
..................uaugccugucugagcgucgAu.....................................................................   1      1   mv7
..................uaugccugucugagAgucgcu.....................................................................   5      1   mv7
..................uaugccugucugaUcgucgcu.....................................................................   1      1   mv7
..................uaugccugucugaAcgucgcu.....................................................................   2      1   mv7
..................uaugccugucAgagcgucgcu.....................................................................   1      1   mv7
..................uaugccugCcugagcgucgcu.....................................................................   1      1   mv7
..................uaugccugucugagcgucgcA.....................................................................   36     1   mv7
..................Naugccugucugagcgucgcu.....................................................................   1      1   mv7
..................uGugccugucugagcgucgcu.....................................................................   8      1   mv7
..................uaugccugucugagcguGgcu.....................................................................   3      1   mv7
..................uaugccugucugaCcgucgcu.....................................................................   1      1   mv7
..................uaugGcugucugagcgucgcu.....................................................................   1      1   mv7
..................uaugccugucugagcgucgcC.....................................................................   91     1   mv7
..................uaugccugucugUgcgucgcuu....................................................................   1      1   mv7
..................uaugccugucugagcgucUcuu....................................................................   1      1   mv7
..................uaugccugucugaUcgucgcuu....................................................................   1      1   mv7
..................uaugccugucugagcgucgcuG....................................................................   1      1   mv7
..................uCugccugucugagcgucgcuu....................................................................   1      1   mv7
..................uaCgccugucugagcgucgcuu....................................................................   24995  1   mv7
..................uaugccugucugaAcgucgcuu....................................................................   1      1   mv7
..................uauCccugucugagcgucgcuu....................................................................   1      1   mv7
..................Aaugccugucugagcgucgcuu....................................................................   1      1   mv7
..................uaugccugucugCgcgucgcuu....................................................................   1      1   mv7
..................uaGgccugucugagcgucgcuu....................................................................   136    1   mv7
..................uauAccugucugagcgucgcuu....................................................................   1      1   mv7
..................uaugccugucugagcgucgcuu....................................................................   110    0   mv7
..................uaugccugucugagcgucgcCu....................................................................   1      1   mv7
..................uaugccugucugagcgucgcuA....................................................................   3      1   mv7
..................Caugccugucugagcgucgcuu....................................................................   2      1   mv7
..................uaugccugucugagcgucgcuC....................................................................   16     1   mv7
..................uaugccuguAugagcgucgcuu....................................................................   1      1   mv7
..................uaugcUugucugagcgucgcuu....................................................................   3      1   mv7
..................uaAgccugucugagcgucgcuu....................................................................   66     1   mv7
..................uaugccugucugagcgucgcuAg...................................................................   1      1   mv7
..................uaugccugucugagcgucgcuuU...................................................................   1      1   mv7
..................uaNgccugucugagcgucgcuug...................................................................   1      1   mv7
..................uaGgccugucugagcgucgcuug...................................................................   1      1   mv7
..................uaCgccugucugagcgucgcuug...................................................................   115    1   mv7
..................uaCgccugucugagcgucgcuugc..................................................................   238    1   mv7
..................uaAgccugucugagcgucgcuugc..................................................................   1      1   mv7
..................uaCgccugucugagcgucgcuugcc.................................................................   34     1   mv7
..................uaCgccugucugagcgucgcuugccg................................................................   44     1   mv7
..................uaugccugucugagcgucgcuugccga...............................................................   1      0   mv7
..................uaCgccugucugagcgucgcuugccga...............................................................   74     1   mv7
```

```
                                   Mature                                                        Star
cuggguuccucccagggcuaugccugucugagcgucgcuugccgaucaaaauuccccaggguugccucugggcuccuuggggugcccagcuguucuguggcagggccc
..................uaGgccugucugagcgucgcuugccgau..............................................................   1        1   mv7
..................uaCgccugucugagcgucgcuugccgau..............................................................   85       1   mv7
..................uaAgccugucugagcgucgcuugccgau..............................................................   2        1   mv7
..................uaCgccugucugagcgucgcuugccgauc.............................................................   50       1   mv7
..................uaCgccugucugagcgucgcuugccgauca............................................................   12       1   mv7
..................uaCgccugucugagcgucgcuugccgaucaa...........................................................   13       1   mv7
..................uaGgccugucugagcgucgcuugccgaucaaa..........................................................   1        1   mv7
..................uaCgccugucugagcgucgcuugccgaucaaa..........................................................   9        1   mv7
...................augccugucugagcgucg.......................................................................   2        0   mv7
...................aCgccugucugagcgucg.......................................................................   625      1   mv7
...................Cugccugucugagcgucg.......................................................................   1        1   mv7
...................aAgccugucugagcgucg.......................................................................   2        1   mv7
...................aGgccugucugagcgucgc......................................................................   2        1   mv7
...................aNgccugucugagcgucgc......................................................................   1        1   mv7
...................aAgccugucugagcgucgc......................................................................   1        1   mv7
...................augccugucugagcgucgU......................................................................   1        1   mv7
...................augccugucugagcgucgc......................................................................   2        0   mv7
...................aCgccugucugagcgucgc......................................................................   1111     1   mv7
...................augccuguUugagcgucgcu.....................................................................   2        1   mv7
...................auAccugucugagcgucgcu.....................................................................   3        1   mv7
...................augccugucugagAgucgcu.....................................................................   1        1   mv7
...................augccugucCgagcgucgcu.....................................................................   1        1   mv7
...................augccugucugagcUucgcu.....................................................................   3        1   mv7
...................Cugccugucugagcgucgcu.....................................................................   22       1   mv7
...................augccugucugagcgucUcu.....................................................................   2        1   mv7
...................augccugucugagcgucgcu.....................................................................   383      0   mv7
...................augccugucugagcgucgcC.....................................................................   53       1   mv7
...................augccugucugagcgucgUu.....................................................................   1        1   mv7
...................auCccugucugagcgucgcu.....................................................................   3        1   mv7
...................augccugCcugagcgucgcu.....................................................................   2        1   mv7
...................augccugucugagcguUgcu.....................................................................   1        1   mv7
...................augccugucugagcgCcgcu.....................................................................   2        1   mv7
...................augccugucugGgcgucgcu.....................................................................   1        1   mv7
...................augccugucugagcgucgcA.....................................................................   17       1   mv7
...................augccGgucugagcgucgcu.....................................................................   1        1   mv7
...................aAgccugucugagcgucgcu.....................................................................   278      1   mv7
...................aNgccugucugagcgucgcu.....................................................................   1        1   mv7
...................augccugucugagcAucgcu.....................................................................   3        1   mv7
...................auUccugucugagcgucgcu.....................................................................   5        1   mv7
...................aCgccugucugagcgucgcu.....................................................................   113087   1   mv7
...................augccuAucugagcgucgcu.....................................................................   1        1   mv7
...................augccugucugagUgucgcu.....................................................................   2        1   mv7
...................aGgccugucugagcgucgcu.....................................................................   152      1   mv7
...................augccCgucugagcgucgcu.....................................................................   2        1   mv7
...................augccugucuUagcgucgcu.....................................................................   1        1   mv7
...................augAcugucugagcgucgcu.....................................................................   21       1   mv7
...................Gugccugucugagcgucgcu.....................................................................   2        1   mv7
...................augcUugucugagcgucgcu.....................................................................   1        1   mv7
...................augccugucugagcgucgcG.....................................................................   6        1   mv7
...................augccuCucugagcgucgcu.....................................................................   1        1   mv7
...................Gugccugucugagcgucgcuu....................................................................   2        1   mv7
...................aGgccugucugagcgucgcuu....................................................................   11       1   mv7
...................auCccugucugagcgucgcuu....................................................................   1        1   mv7
...................augccCgucugagcgucgcuu....................................................................   2        1   mv7
...................auUccugucugagcgucgcuu....................................................................   3        1   mv7
...................augccugucugagcgucgcuu....................................................................   65       0   mv7
...................augAcugucugagcgucgcuu....................................................................   2        1   mv7
...................augccugucugagcgucgcuA....................................................................   1        1   mv7
...................auAccugucugagcgucgcuu....................................................................   1        1   mv7
...................augccGgucugagcgucgcuu....................................................................   1        1   mv7
...................aCgccugucugagcgucgcuu....................................................................   13635    1   mv7
...................augccAgucugagcgucgcuu....................................................................   1        1   mv7
...................augccugucCgagcgucgcuu....................................................................   1        1   mv7
...................aAgccugucugagcgucgcuu....................................................................   25       1   mv7
...................augccugucugagcgucgcuC....................................................................   6        1   mv7
...................aCgccugucugagcgucgcuug...................................................................   43       1   mv7
...................Cugccugucugagcgucgcuugc..................................................................   1        1   mv7
...................aCgccugucugagcgucgcuugc..................................................................   114      1   mv7
...................augccugucugagcgucgcuugc..................................................................   1        0   mv7
...................aCgccugucugagcgucgcuugcc.................................................................   17       1   mv7
```

```
                     Mature                                                          Star
cuggguuccucccagggcuaugccugucugagcgucgcuugccgaucaaaauuccccaggguugccucugggcuccuuggggugcccagcuguucuguggcagggccc
...................aCgccugucugagcgucgcuugccg................................................................    20     1    mv7
...................aCgccugucugagcgucgcuugccga...............................................................    21     1    mv7
...................aCgccugucugagcgucgcuugccgau..............................................................    25     1    mv7
...................aCgccugucugagcgucgcuugccgauc.............................................................    15     1    mv7
...................aCgccugucugagcgucgcuugccgauca............................................................    5      1    mv7
...................aCgccugucugagcgucgcuugccgaucaa...........................................................    6      1    mv7
...................aCgccugucugagcgucgcuugccgaucaaa..........................................................    7      1    mv7
...................aCgccugucugagcgucgcuugccgaucaaaa.........................................................    3      1    mv7
....................ugccugucugGgcgucgc......................................................................    1      1    mv7
....................ugccugucugagAgucgc......................................................................    2      1    mv7
....................ugccugucugagUgucgc......................................................................    3      1    mv7
....................ugcGugucugagcgucgc......................................................................    3      1    mv7
....................ugccuguUugagcgucgc......................................................................    1      1    mv7
....................ugccugucugagcgucgU......................................................................    35     1    mv7
....................ugccugucugagcgucgA......................................................................    6      1    mv7
....................ugccugucugagcgGcgc......................................................................    1      1    mv7
....................Ggccugucugagcgucgc......................................................................    14     1    mv7
....................ugGcugucugagcgucgc......................................................................    2      1    mv7
....................ugcUugucugagcgucgc......................................................................    1      1    mv7
....................uUccugucugagcgucgc......................................................................    4      1    mv7
....................Cgccugucugagcgucgc......................................................................    6803   1    mv7
....................ugccugucugagcgucgG......................................................................    1      1    mv7
....................Ngccugucugagcgucgc......................................................................    4      1    mv7
....................ugcAugucugagcgucgc......................................................................    13     1    mv7
....................Agccugucugagcgucgc......................................................................    157    1    mv7
....................ugccugucugagcgucgc......................................................................    119    0    mv7
....................ugccugucugagcgCcgc......................................................................    2      1    mv7
....................ugccuguGugagcgucgcu.....................................................................    3      1    mv7
....................ugccugucugagcguUgcu.....................................................................    29     1    mv7
....................ugccuCucugagcgucgcu.....................................................................    4      1    mv7
....................ugccugucugagcgucgcC.....................................................................    859    1    mv7
....................ugccugucugagcUucgcu.....................................................................    6      1    mv7
....................ugccugucCgagcgucgcu.....................................................................    41     1    mv7
....................ugccugucugagcgucgcG.....................................................................    164    1    mv7
....................ugccugucugagcgucUcu.....................................................................    7      1    mv7
....................ugccugucugGgcgucgcu.....................................................................    40     1    mv7
....................ugccugucugagcgucgAu.....................................................................    18     1    mv7
....................ugAcugucugagcgucgcu.....................................................................    13     1    mv7
....................ugccugGcugagcgucgcu.....................................................................    6      1    mv7
....................ugccugucugagAgucgcu.....................................................................    112    1    mv7
....................ugccGgucugagcgucgcu.....................................................................    3      1    mv7
....................uUccugucugagcgucgcu.....................................................................    18     1    mv7
....................ugccugucuUagcgucgcu.....................................................................    13     1    mv7
....................ugccAgucugagcgucgcu.....................................................................    7      1    mv7
....................ugccCgucugagcgucgcu.....................................................................    14     1    mv7
....................uAccugucugagcgucgcu.....................................................................    43     1    mv7
....................ugccugCcugagcgucgcu.....................................................................    19     1    mv7
....................ugccugucugagcgucgcu.....................................................................    6368   0    mv7
....................ugccugucugagcgCcgcu.....................................................................    30     1    mv7
....................ugccugucugagUgucgcu.....................................................................    39     1    mv7
....................ugccugucugagcgucgGu.....................................................................    14     1    mv7
....................Ngccugucugagcgucgcu.....................................................................    179    1    mv7
....................ugccugucugUgcgucgcu.....................................................................    11     1    mv7
....................uCccugucugagcgucgcu.....................................................................    24     1    mv7
....................ugccugucugaCcgucgcu.....................................................................    2      1    mv7
....................ugccugucuCagcgucgcu.....................................................................    3      1    mv7
....................ugcUugucugagcgucgcu.....................................................................    43     1    mv7
....................ugccugucugagcgucgUu.....................................................................    21     1    mv7
....................ugccugucugagcgucgcA.....................................................................    310    1    mv7
....................Ggccugucugagcgucgcu.....................................................................    721    1    mv7
....................ugccugucugCgcgucgcu.....................................................................    16     1    mv7
....................ugcGugucugagcgucgcu.....................................................................    143    1    mv7
....................ugccuguUugagcgucgcu.....................................................................    12     1    mv7
....................ugGcugucugagcgucgcu.....................................................................    166    1    mv7
....................ugccuAucugagcgucgcu.....................................................................    7      1    mv7
....................ugccugucugaAcgucgcu.....................................................................    9      1    mv7
....................ugccugucugagcCucgcu.....................................................................    3      1    mv7
....................ugccugucugagcAucgcu.....................................................................    8      1    mv7
....................ugccugucGgagcgucgcu.....................................................................    4      1    mv7
....................Agccugucugagcgucgcu.....................................................................    8881   1    mv7
```

```
                    Mature                                                             Star
cuggguuccucccagggcuaugccugucugagcgucgcuugccgaucaaaauuccccaggguugccucugggcuccuuggggugcccagcuguucuguggcagggccc
....................Cgccugucugagcgucgcu.....................................................................  319253  1  mv7
....................ugcAugucugagcgucgcu.....................................................................  556     1  mv7
....................ugccugucugagcgucCcu.....................................................................  3       1  mv7
....................ugccuUucugagcgucgcu.....................................................................  27      1  mv7
....................ugccugucugagcguGgcu.....................................................................  3       1  mv7
....................ugccugucAgagcgucgcu.....................................................................  5       1  mv7
....................ugccuguAugagcgucgcu.....................................................................  23      1  mv7
....................ugccugucugaUcgucgcu.....................................................................  9       1  mv7
....................ugccugAcugagcgucgcu.....................................................................  6       1  mv7
....................ugccugucugagcgGcgcu.....................................................................  14      1  mv7
....................ugccugucugCgcgucgcuu....................................................................  1       1  mv7
....................ugccugucugagcgGcgcuu....................................................................  4       1  mv7
....................ugAcugucugagcgucgcuu....................................................................  4       1  mv7
....................ugcAugucugagcgucgcuu....................................................................  77      1  mv7
....................ugccugucugagcgucgcGu....................................................................  2       1  mv7
....................Agccugucugagcgucgcuu....................................................................  1327    1  mv7
....................ugccugucugagcgucgcuA....................................................................  28      1  mv7
....................ugccuguAugagcgucgcuu....................................................................  2       1  mv7
....................ugcGugucugagcgucgcuu....................................................................  17      1  mv7
....................uAccugucugagcgucgcuu....................................................................  9       1  mv7
....................uCccugucugagcgucgcuu....................................................................  6       1  mv7
....................ugccugucugagcUucgcuu....................................................................  1       1  mv7
....................ugccugucCgagcgucgcuu....................................................................  6       1  mv7
....................ugccCgucugagcgucgcuu....................................................................  1       1  mv7
....................ugccugucugagcgCcgcuu....................................................................  1       1  mv7
....................ugccugucugagcguUgcuu....................................................................  5       1  mv7
....................ugccugucugagcgucgUuu....................................................................  1       1  mv7
....................ugccugucugagcAucgcuu....................................................................  1       1  mv7
....................ugccugucugUgcgucgcuu....................................................................  3       1  mv7
....................ugGcugucugagcgucgcuu....................................................................  19      1  mv7
....................ugccugAcugagcgucgcuu....................................................................  1       1  mv7
....................uUccugucugagcgucgcuu....................................................................  6       1  mv7
....................ugccugucugaUcgucgcuu....................................................................  1       1  mv7
....................ugccuguGugagcgucgcuu....................................................................  2       1  mv7
....................ugccugucugagcgucgcuu....................................................................  968     0  mv7
....................ugccuUucugagcgucgcuu....................................................................  4       1  mv7
....................Ngccugucugagcgucgcuu....................................................................  25      1  mv7
....................ugccugucugagcgucgcuC....................................................................  119     1  mv7
....................ugcUugucugagcgucgcuu....................................................................  3       1  mv7
....................ugccugucugagcgucgGuu....................................................................  2       1  mv7
....................ugccugucuCagcgucgcuu....................................................................  1       1  mv7
....................ugccugucugagcguAgcuu....................................................................  3       1  mv7
....................ugccugucugagcgucgcCu....................................................................  2       1  mv7
....................ugccugucugaAcgucgcuu....................................................................  1       1  mv7
....................ugccugucugagcgucgcuG....................................................................  37      1  mv7
....................Cgccugucugagcgucgcuu....................................................................  48360   1  mv7
....................Ggccugucugagcgucgcuu....................................................................  100     1  mv7
....................ugccugucugagcgucUcuu....................................................................  2       1  mv7
....................ugccugucGgagcgucgcuu....................................................................  1       1  mv7
....................ugccugCcugagcgucgcuu....................................................................  6       1  mv7
....................ugccugucugagcguGgcuu....................................................................  1       1  mv7
....................ugccugucugGgcgucgcuu....................................................................  7       1  mv7
....................ugccugucuUagcgucgcuu....................................................................  2       1  mv7
....................ugccugucugagUgucgcuu....................................................................  6       1  mv7
....................ugccugucugagAgucgcuu....................................................................  17      1  mv7
....................ugccuAucugagcgucgcuu....................................................................  1       1  mv7
....................ugccugucugagcgucgcuug...................................................................  4       0  mv7
....................Cgccugucugagcgucgcuug...................................................................  164     1  mv7
....................Agccugucugagcgucgcuug...................................................................  9       1  mv7
....................Cgccugucugagcgucgcuugc..................................................................  343     1  mv7
....................ugccugucugagAgucgcuugc..................................................................  1       1  mv7
....................ugccugucugagcgucgcuugA..................................................................  1       1  mv7
....................Ggccugucugagcgucgcuugc..................................................................  2       1  mv7
....................Agccugucugagcgucgcuugc..................................................................  12      1  mv7
....................ugccugucugagcgucgcuugc..................................................................  1       0  mv7
....................ugccugucugagcgucgcuuCc..................................................................  1       1  mv7
....................ugccugucugagcgucgcuugcA.................................................................  1       1  mv7
....................ugccugucugagcgucgcuugcc.................................................................  2       0  mv7
....................ugcAugucugagcgucgcuugcc.................................................................  1       1  mv7
....................Cgccugucugagcgucgcuugcc.................................................................  32      1  mv7
```

```
                    Mature                                                              Star
cuggguuccucccagggcuaugccugucugagcgucgcuugccgaucaaaauuccccaggguugccucugggcuccuuggggugcccagcuguucuguggcagggccc
....................Agccugucugagcgucgcuugcc.................................................................  1      1  mv7
....................ugccugucugagcgucgcuugcU.................................................................  1      1  mv7
....................Cgccugucugagcgucgcuugccg................................................................  36     1  mv7
....................Agccugucugagcgucgcuugccg................................................................  2      1  mv7
....................ugccugucugagcgucgcuugccg................................................................  2      0  mv7
....................Agccugucugagcgucgcuugccga...............................................................  1      1  mv7
....................Cgccugucugagcgucgcuugccga...............................................................  43     1  mv7
....................ugccugucugagcgucgcuugccgaA..............................................................  1      1  mv7
....................Agccugucugagcgucgcuugccgau..............................................................  1      1  mv7
....................Cgccugucugagcgucgcuugccgau..............................................................  39     1  mv7
....................ugGcugucugagcgucgcuugccgau..............................................................  1      1  mv7
....................Agccugucugagcgucgcuugccgauc.............................................................  1      1  mv7
....................Cgccugucugagcgucgcuugccgauc.............................................................  37     1  mv7
....................ugccugucugagcgucgcuugccgauA.............................................................  1      1  mv7
....................Agccugucugagcgucgcuugccgauca............................................................  2      1  mv7
....................Cgccugucugagcgucgcuugccgauca............................................................  17     1  mv7
....................Cgccugucugagcgucgcuugccgaucaa...........................................................  10     1  mv7
....................Cgccugucugagcgucgcuugccgaucaaa..........................................................  18     1  mv7
....................Cgccugucugagcgucgcuugccgaucaaaa.........................................................  7      1  mv7
....................Agccugucugagcgucgcuugccgaucaaaa.........................................................  2      1  mv7
.....................gccugGcugagcgucgcu.....................................................................  143    1  mv7
.....................gccuAucugagcgucgcu.....................................................................  32     1  mv7
.....................gccAgucugagcgucgcu.....................................................................  234    1  mv7
.....................gccugucugagcguUgcu.....................................................................  81     1  mv7
.....................gccugucugagcgucgcu.....................................................................  35057  0  mv7
.....................gccugucCgagcgucgcu.....................................................................  120    1  mv7
.....................gccugucugagcgGcgcu.....................................................................  47     1  mv7
.....................gccGgucugagcgucgcu.....................................................................  4      1  mv7
.....................gccugucugagcgucUcu.....................................................................  86     1  mv7
.....................gcGugucugagcgucgcu.....................................................................  299    1  mv7
.....................gccugucugaUcgucgcu.....................................................................  31     1  mv7
.....................gccugucugagcgucgAu.....................................................................  159    1  mv7
.....................gccuguGugagcgucgcu.....................................................................  34     1  mv7
.....................gcNugucugagcgucgcu.....................................................................  1      1  mv7
.....................gccCgucugagcgucgcu.....................................................................  238    1  mv7
.....................gccugucUagcgucgcu.....................................................................  24     1  mv7
.....................gccugucugagcCucgcu.....................................................................  168    1  mv7
.....................gccuNucugagcgucgcu.....................................................................  3      1  mv7
.....................gcAugucugagcgucgcu.....................................................................  124    1  mv7
.....................gccuguAugagcgucgcu.....................................................................  96     1  mv7
.....................gccugucugagcgucgcA.....................................................................  1552   1  mv7
.....................gccugucugGgcgucgcu.....................................................................  136    1  mv7
.....................gccugucugaCcgucgcu.....................................................................  18     1  mv7
.....................gccugucugagcUucgcu.....................................................................  41     1  mv7
.....................gccugucugagcgucgUu.....................................................................  133    1  mv7
.....................gccugAcugagcgucgcu.....................................................................  50     1  mv7
.....................gccugucugagcgucCcu.....................................................................  15     1  mv7
.....................gccuCucugagcgucgcu.....................................................................  5      1  mv7
.....................gccuguUugagcgucgcu.....................................................................  89     1  mv7
.....................gccugucugagUgucgcu.....................................................................  118    1  mv7
.....................gccugucugagcgucgcG.....................................................................  861    1  mv7
.....................Cccugucugagcgucgcu.....................................................................  598    1  mv7
.....................gccugucugagGgucgcu.....................................................................  8      1  mv7
.....................gccuUucugagcgucgcu.....................................................................  15     1  mv7
.....................gccugucGgagcgucgcu.....................................................................  11     1  mv7
.....................gccugucugCgcgucgcu.....................................................................  62     1  mv7
.....................gccugucuCagcgucgcu.....................................................................  69     1  mv7
.....................gccugucAgagcgucgcu.....................................................................  50     1  mv7
.....................gccugucugagcAucgcu.....................................................................  54     1  mv7
.....................gccugucugagcgucgcC.....................................................................  4787   1  mv7
.....................Accugucugagcgucgcu.....................................................................  348    1  mv7
.....................gAcugucugagcgucgcu.....................................................................  115    1  mv7
.....................gccugucugagcgucgGu.....................................................................  80     1  mv7
.....................gccugucugUgcgucgcu.....................................................................  56     1  mv7
.....................Nccugucugagcgucgcu.....................................................................  19     1  mv7
.....................gccugucugagAgucgcu.....................................................................  24     1  mv7
.....................gcUugucugagcgucgcu.....................................................................  100    1  mv7
.....................gccugucugaAcgucgcu.....................................................................  52     1  mv7
.....................gGcugucugagcgucgcu.....................................................................  83     1  mv7
.....................gccugucugagcguGgcu.....................................................................  134    1  mv7
```

Mature **Star**

```
cuggguuccucccagggcuaugccugucugagcgucgcuugccgaucaaaauuccccaggguugccucugggcuccuuggggugcccagcuguucuguggcagggccc
.....................gccugCcugagcgucgcu.....................................................................   133    1    mv7
.....................Uccugucugagcgucgcu.....................................................................   486    1    mv7
.....................gccugucugagUgucgcuu....................................................................   9      1    mv7
.....................gAcugucugagcgucgcuu....................................................................   33     1    mv7
.....................gccugGcugagcgucgcuu....................................................................   29     1    mv7
.....................gccugucugagcUucgcuu....................................................................   4      1    mv7
.....................gccugucugagcgucgUuu....................................................................   14     1    mv7
.....................gccugucugagcgucgcuA....................................................................   166    1    mv7
.....................gcAugucugagcgucgcuu....................................................................   23     1    mv7
.....................gccugucugaCcgucgcuu....................................................................   2      1    mv7
.....................gcGugucugagcgucgcuu....................................................................   57     1    mv7
.....................gccugCcugagcgucgcuu....................................................................   20     1    mv7
.....................gccugucugagcgucCcuu....................................................................   5      1    mv7
.....................gccugucugUgcgucgcuu....................................................................   15     1    mv7
.....................gccugucugagcgCcgcuu....................................................................   27     1    mv7
.....................gccNgucugagcgucgcuu....................................................................   2      1    mv7
.....................gccugucugagAgucgcuu....................................................................   6      1    mv7
.....................gccuAucugagcgucgcuu....................................................................   9      1    mv7
.....................gccugucGgagcgucgcuu....................................................................   5      1    mv7
.....................gccugucugagcgucgcuu....................................................................   7432   0    mv7
.....................gccugucugCgcgucgcuu....................................................................   17     1    mv7
.....................gccugucugagcgucgcGu....................................................................   44     1    mv7
.....................gccugucugagcgucgcuG....................................................................   91     1    mv7
.....................gccugucugaAcgucgcuu....................................................................   9      1    mv7
.....................gccugucugGgcgucgcuu....................................................................   26     1    mv7
.....................gccugucugagcgucgAuu....................................................................   36     1    mv7
.....................gccugucugagcgGcgcuu....................................................................   10     1    mv7
.....................gccuguAugagcgucgcuu....................................................................   23     1    mv7
.....................gccugucugagcgucgcAu....................................................................   12     1    mv7
.....................gccugucugagGgucgcuu....................................................................   2      1    mv7
.....................gccugucugaUcgucgcuu....................................................................   2      1    mv7
.....................gccugucCgagcgucgcuu....................................................................   26     1    mv7
.....................gccugucugagcguGgcuu....................................................................   23     1    mv7
.....................gccugucuUagcgucgcuu....................................................................   5      1    mv7
.....................gccAgucugagcgucgcuu....................................................................   55     1    mv7
.....................gccugucugagcgucUcuu....................................................................   17     1    mv7
.....................gccugucugagcguUgcuu....................................................................   16     1    mv7
.....................gccCgucugagcgucgcuu....................................................................   48     1    mv7
.....................gccugucuCagcgucgcuu....................................................................   12     1    mv7
.....................gccugucugagcguAgcuu....................................................................   40     1    mv7
.....................gccGgucugagcgucgcuu....................................................................   2      1    mv7
.....................gccugAcugagcgucgcuu....................................................................   10     1    mv7
.....................gccugucugagcCucgcuu....................................................................   37     1    mv7
.....................gccugucugagcgucgGuu....................................................................   11     1    mv7
.....................gGcugucugagcgucgcuu....................................................................   11     1    mv7
.....................Accugucugagcgucgcuu....................................................................   75     1    mv7
.....................gccugucugagcgucgcuC....................................................................   826    1    mv7
.....................Cccugucugagcgucgcuu....................................................................   108    1    mv7
.....................gccugucAgagcgucgcuu....................................................................   12     1    mv7
.....................gccugucugagcAucgcuu....................................................................   5      1    mv7
.....................Nccugucugagcgucgcuu....................................................................   3      1    mv7
.....................gcUugucugagcgucgcuu....................................................................   31     1    mv7
.....................gccuguGugagcgucgcuu....................................................................   12     1    mv7
.....................gccuguUugagcgucgcuu....................................................................   8      1    mv7
.....................Uccugucugagcgucgcuu....................................................................   101    1    mv7
.....................gccugucugagcgucgcCu....................................................................   49     1    mv7
.....................gccuUucugagcgucgcuu....................................................................   1      1    mv7
.....................gccugucugagcgucgcAug...................................................................   1      1    mv7
.....................gccugucugagcgucgcuug...................................................................   14     0    mv7
.....................gccugucugagcgucgcuuA...................................................................   2      1    mv7
.....................gccugCcugagcgucgcuug...................................................................   1      1    mv7
.....................gAcugucugagcgucgcuug...................................................................   1      1    mv7
.....................gccugucugagcgucgcuuU...................................................................   29     1    mv7
.....................gccugucugagcgucgcuAg...................................................................   2      1    mv7
.....................gccugucugagcgucgcuuC...................................................................   4      1    mv7
.....................gccugucugagcgucgcuGg...................................................................   1      1    mv7
.....................gccCgucugagcgucgcuugc..................................................................   1      1    mv7
.....................gccugucugagcgucgcuugA..................................................................   4      1    mv7
.....................gccugucugagcgucgcuuUc..................................................................   1      1    mv7
.....................gccugucugagcgucgcuugU..................................................................   6      1    mv7
```

```
                    Mature                                                              Star
cuggguuccucccagggcuaugccugucugagcgucgcuugccgaucaaaauuccccaggguugccucugggcuccuuggggugcccagcuguucuguggcagggccc
.....................gccugucugagcgucgcuugc..................................................................   18      0   mv7
.....................gccugucugagcCucgcuugc..................................................................   1       1   mv7
.....................gccugucugagcgucgcuugcc.................................................................   6       0   mv7
.....................gccugucugagcgucgcuugcA.................................................................   2       1   mv7
.....................gccugucugagcgucgAuugcc.................................................................   1       1   mv7
.....................gccugucugagcgucgcuugccA................................................................   1       1   mv7
.....................gccugucugagcgucgcuugccgG...............................................................   1       1   mv7
.....................gccugucugagcgucgcuugccga...............................................................   1       0   mv7
.....................gccugucugCgcgucgcuugccga...............................................................   1       1   mv7
.....................gccugucugagcgucgcuugccgau..............................................................   8       0   mv7
.....................gccuguGugagcgucgcuugccgau..............................................................   1       1   mv7
.....................gccugucugagcgucgcuugccgaA..............................................................   1       1   mv7
.....................gccugucugGgcgucgcuugccgauc.............................................................   1       1   mv7
.....................gccugucugagcgucgcuugccgauc.............................................................   6       0   mv7
.....................gccugucugagcgucgcuugccgauA.............................................................   1       1   mv7
.....................gccugucugagcgucgGuugccgauc.............................................................   1       1   mv7
.....................gccugucugagcgucgcuugccgauAa............................................................   1       1   mv7
.....................gccCgucugagcgucgcuugccgauca............................................................   1       1   mv7
.....................gcGugucugagcgucgcuugccgauca............................................................   1       1   mv7
.....................gccugucugagcgucgcuugccgauca............................................................   3       0   mv7
.....................gccugucugagcgucgcuugccgaucaa...........................................................   1       0   mv7
.....................gccugucugagcgucgcuugccgaucaaU..........................................................   3       1   mv7
.....................gcGugucugagcgucgcuugccgaucaaaa.........................................................   1       1   mv7
......................ccugucugagcgucgcuu....................................................................   113818  0   mv7
......................ccugucuNagcgucgcuu....................................................................   1       1   mv7
......................ccCgucugagcgucgcuu....................................................................   394     1   mv7
......................ccGgucugagcgucgcuu....................................................................   816     1   mv7
......................ccugucugaAcgucgcuu....................................................................   148     1   mv7
......................cAugucugagcgucgcuu....................................................................   260     1   mv7
......................ccugucugagUgucgcuu....................................................................   258     1   mv7
......................Gcugucugagcgucgcuu....................................................................   263     1   mv7
......................ccugucugagcgucgGuu....................................................................   67      1   mv7
......................ccugucugagcgucgcuC....................................................................   12936   1   mv7
......................cNugucugagcgucgcuu....................................................................   1       1   mv7
......................ccugucugagcgCcgcuu....................................................................   727     1   mv7
......................ccugucugagcgucUcuu....................................................................   556     1   mv7
......................ccugAcugagcgucgcuu....................................................................   93      1   mv7
......................Acugucugagcgucgcuu....................................................................   3098    1   mv7
......................ccugNcugagcgucgcuu....................................................................   7       1   mv7
......................ccugucAgagcgucgcuu....................................................................   260     1   mv7
......................cUugucugagcgucgcuu....................................................................   189     1   mv7
......................ccuguGugagcgucgcuu....................................................................   16      1   mv7
......................ccugucugUgcgucgcuu....................................................................   184     1   mv7
......................ccuguNugagcgucgcuu....................................................................   1       1   mv7
......................ccugucuUagcgucgcuu....................................................................   44      1   mv7
......................Ncugucugagcgucgcuu....................................................................   54      1   mv7
......................ccugGcugagcgucgcuu....................................................................   27      1   mv7
......................ccuguAugagcgucgcuu....................................................................   87      1   mv7
......................ccuAucugagcgucgcuu....................................................................   855     1   mv7
......................ccuNucugagcgucgcuu....................................................................   4       1   mv7
......................ccugucugagcgucgUuu....................................................................   187     1   mv7
......................ccugucuCagcgucgcuu....................................................................   41      1   mv7
......................ccuCucugagcgucgcuu....................................................................   374     1   mv7
......................ccugucugagGgucgcuu....................................................................   63      1   mv7
......................ccugCcugagcgucgcuu....................................................................   334     1   mv7
......................ccugucugagcgucgcuG....................................................................   1617    1   mv7
......................ccugucugGgcgucgcuu....................................................................   459     1   mv7
......................ccNgucugagcgucgcuu....................................................................   4       1   mv7
......................ccugucugagcgucgcuA....................................................................   3059    1   mv7
......................ccugucGgagcgucgcuu....................................................................   125     1   mv7
......................ccugucugagcUucgcuu....................................................................   32      1   mv7
......................ccugucugCgcgucgcuu....................................................................   446     1   mv7
......................ccugucugagcAucgcuu....................................................................   111     1   mv7
......................ccugucugaUcgucgcuu....................................................................   81      1   mv7
......................ccugucugagcgucgcGu....................................................................   687     1   mv7
......................ccuUucugagcgucgcuu....................................................................   25      1   mv7
......................ccugucugagAgucgcuu....................................................................   178     1   mv7
......................ccugucugagcgucgAuu....................................................................   248     1   mv7
......................ccugucugagcguUgcuu....................................................................   175     1   mv7
......................cGugucugagcgucgcuu....................................................................   160     1   mv7
```

Mature **Star**

```
cuggguuccucccagggcuaugccugucugagcgucgcuugccgaucaaaauuccccaggguugccucugggcuccuuggggugcccagcuguucuguggcagggccc
......................ccugucugagcgGcgcuu.................................................................... 72 1 mv7
......................ccugucugagcgucgcAu.................................................................... 239 1 mv7
......................ccugucugagcguAgcuu.................................................................... 41 1 mv7
......................ccuguUugagcgucgcuu.................................................................... 190 1 mv7
......................ccugucCgagcgucgcuu.................................................................... 359 1 mv7
......................ccugucugagcguGgcuu.................................................................... 29 1 mv7
......................ccugucugagcCucgcuu.................................................................... 15 1 mv7
......................ccugucugagcgucgcCu.................................................................... 720 1 mv7
......................ccAgucugagcgucgcuu.................................................................... 392 1 mv7
......................ccugucugagcgucCcuu.................................................................... 216 1 mv7
......................ccugucugaCcgucgcuu.................................................................... 56 1 mv7
......................ccugucugGgcgucgcuug................................................................... 2 1 mv7
......................ccAgucugagcgucgcuug................................................................... 6 1 mv7
......................ccugucugagUgucgcuug................................................................... 1 1 mv7
......................ccugucugagcgucgcuGg................................................................... 2 1 mv7
......................Ncugucugagcgucgcuug................................................................... 1 1 mv7
......................ccGgucugagcgucgcuug................................................................... 2 1 mv7
......................ccugucugagcguAgcuug................................................................... 1 1 mv7
......................ccugucugagcgucgcuCg................................................................... 7 1 mv7
......................ccugucugUgcgucgcuug................................................................... 2 1 mv7
......................ccuguUugagcgucgcuug................................................................... 1 1 mv7
......................cGugucugagcgucgcuug................................................................... 1 1 mv7
......................ccuAucugagcgucgcuug................................................................... 1 1 mv7
......................ccugucugagcgucgUuug................................................................... 1 1 mv7
......................ccugucugagcgAcgcuug................................................................... 1 1 mv7
......................cAugucugagcgucgcuug................................................................... 1 1 mv7
......................ccugCcugagcgucgcuug................................................................... 1 1 mv7
......................Gcugucugagcgucgcuug................................................................... 1 1 mv7
......................ccugucugagcgucgcuuA................................................................... 38 1 mv7
......................ccugucugagcgucgcuAg................................................................... 1 1 mv7
......................ccugucugagcgucCcuug................................................................... 1 1 mv7
......................ccuguAugagcgucgcuug................................................................... 1 1 mv7
......................Acugucugagcgucgcuug................................................................... 14 1 mv7
......................ccugucugagcgucgcuuU................................................................... 185 1 mv7
......................ccugucugagcgucgcuuC................................................................... 22 1 mv7
......................ccugucugaUcgucgcuug................................................................... 1 1 mv7
......................ccugucugagcguUgcuug................................................................... 1 1 mv7
......................ccugucugagAgucgcuug................................................................... 1 1 mv7
......................ccugucuUagcgucgcuug................................................................... 1 1 mv7
......................ccugucugagcgucgcuug................................................................... 335 0 mv7
......................ccugucugagcgucUcuug................................................................... 1 1 mv7
......................ccugucCgagcgucgcuug................................................................... 2 1 mv7
......................Gcugucugagcgucgcuugc.................................................................. 2 1 mv7
......................cUugucugagcgucgcuugc.................................................................. 1 1 mv7
......................ccugCcugagcgucgcuugc.................................................................. 1 1 mv7
......................ccugucugagcgucgcuAgc.................................................................. 1 1 mv7
......................ccugucugagcgucgcuCgc.................................................................. 2 1 mv7
......................ccugucugUgcgucgcuugc.................................................................. 1 1 mv7
......................ccugucugagcgucgcCugc.................................................................. 1 1 mv7
......................ccugucugagcgucgcuugU.................................................................. 84 1 mv7
......................ccugucugaUcgucgcuugc.................................................................. 1 1 mv7
......................ccugucugagcAucgcuugc.................................................................. 1 1 mv7
......................ccugucugagcgucUcuugc.................................................................. 1 1 mv7
......................ccugucugagcgucgcuuUc.................................................................. 1 1 mv7
......................ccugucugagcgucgcuGgc.................................................................. 1 1 mv7
......................ccugucugagcgCcgcuugc.................................................................. 2 1 mv7
......................ccuAucugagcgucgcuugc.................................................................. 3 1 mv7
......................ccGgucugagcgucgcuugc.................................................................. 2 1 mv7
......................ccugucugagcgucgcuugc.................................................................. 311 0 mv7
......................ccugucugagcgucgcAugc.................................................................. 1 1 mv7
......................ccugucugagcgucgcuugG.................................................................. 2 1 mv7
......................Acugucugagcgucgcuugc.................................................................. 8 1 mv7
......................ccugucugagcgucgcuugA.................................................................. 34 1 mv7
......................ccugucugagcgucCcuugcc................................................................. 1 1 mv7
......................ccugucugagcgucgcuugcc................................................................. 68 0 mv7
......................Acugucugagcgucgcuugcc................................................................. 4 1 mv7
......................ccugucugagcgucgcuuCcc................................................................. 1 1 mv7
......................ccugucugagcgCcgcuugcc................................................................. 1 1 mv7
......................ccugucugagcgucgcuugcA................................................................. 36 1 mv7
......................ccuguUugagcgucgcuugcc................................................................. 1 1 mv7
```

```
                     Mature                                                Star
cuggguuccucccagggcuaugccugucugagcgucgcuugccgaucaaaauuccccaggguugccucugggcuccuuggggugcccagcuguucuguggcagggccc
......................ccGgucugagcgucgcuugcc.................................................................  1     1     mv7
......................ccugucugagcgucgcuuAcc.................................................................  1     1     mv7
......................ccugucugagAgucgcuugcc.................................................................  1     1     mv7
......................ccugucugagcguUgcuugcc.................................................................  1     1     mv7
......................ccugucugagcgucgcuugcU.................................................................  18    1     mv7
......................ccugucugagcgucgcuAgccg................................................................  2     1     mv7
......................Acugucugagcgucgcuugccg................................................................  1     1     mv7
......................ccugucugagcgucgAuugccg................................................................  1     1     mv7
......................ccugucugagUgucgcuugccg................................................................  1     1     mv7
......................ccugucugagcgucgcuCgccg................................................................  1     1     mv7
......................ccugucugagcgucgcuugccg................................................................  65    0     mv7
......................ccugCcugagcgucgcuugccg................................................................  2     1     mv7
......................ccugucugagcgucgcuugccC................................................................  1     1     mv7
......................ccugucugagcgucgcuugccA................................................................  8     1     mv7
......................ccugucugagcgucgcuugcAg................................................................  1     1     mv7
......................ccugucuUagcgucgcuugccga...............................................................  1     1     mv7
......................ccugucugagcgucgcuugccgC...............................................................  1     1     mv7
......................ccugucugagcgucgcuugccgU...............................................................  2     1     mv7
......................ccuAucugagcgucgcuugccga...............................................................  1     1     mv7
......................ccugucugagcgucgcuugccga...............................................................  30    0     mv7
......................ccugucugagcgucAcuugccga...............................................................  2     1     mv7
......................ccugucugagcgucgcuugccgG...............................................................  1     1     mv7
......................ccugucugagcgucgcuugccgaG..............................................................  1     1     mv7
......................ccugucugagcgucgcuugccgaA..............................................................  10    1     mv7
......................Gcugucugagcgucgcuugccgau..............................................................  1     1     mv7
......................ccugucugagcgucgcuugccgaC..............................................................  2     1     mv7
......................Acugucugagcgucgcuugccgau..............................................................  1     1     mv7
......................ccugucugagcgucCcuugccgau..............................................................  1     1     mv7
......................ccugCcugagcgucgcuugccgau..............................................................  1     1     mv7
......................ccugucugagcgucAcuugccgau..............................................................  1     1     mv7
......................ccugucugagcgucgcuugccgau..............................................................  56    0     mv7
......................ccugucugagcgucgcuCgccgauc.............................................................  1     1     mv7
......................ccugucCgagcgucgcuugccgauc.............................................................  1     1     mv7
......................ccugucugagcgucgcuugccgGuc.............................................................  1     1     mv7
......................ccugucugagcgucgcuugccgauU.............................................................  3     1     mv7
......................ccuAucugagcgucgcuugccgauc.............................................................  1     1     mv7
......................ccugucugagcgucAcuugccgauc.............................................................  2     1     mv7
......................cAugucugagcgucgcuugccgauc.............................................................  1     1     mv7
......................ccugucugagcgucgcuugccgauc.............................................................  57    0     mv7
......................ccugucugagcgucgcuugccgauA.............................................................  16    1     mv7
......................Acugucugagcgucgcuugccgauc.............................................................  3     1     mv7
......................ccugucugagcgucgcuugccgauG.............................................................  1     1     mv7
......................ccugucugaUcgucgcuugccgauc.............................................................  1     1     mv7
......................ccugucugagcgucgcuugccgauca............................................................  9     0     mv7
......................ccugucugagcgucgcuugccgaucU............................................................  1     1     mv7
......................ccugucugagcgucgcuugccgauAa............................................................  3     1     mv7
......................ccugucugagcgucgcuugccgauGaa...........................................................  1     1     mv7
......................ccugucugagcgucgcuugccgaucaa...........................................................  10    0     mv7
......................ccugucugagcgucgcuugccgauAaa...........................................................  7     1     mv7
......................ccugucugagcgucgcuugccgaucaaU..........................................................  6     1     mv7
......................ccugucugagUgucgcuugccgaucaaa..........................................................  1     1     mv7
......................ccugucugagcgucgcuugccgaucaaa..........................................................  21    0     mv7
......................ccugucugagcgucgcuugccgauAaaa..........................................................  7     1     mv7
......................ccugucugagcgucgcuugccgaucaaUa.........................................................  12    1     mv7
......................ccugCcugagcgucgcuugccgaucaaaa.........................................................  1     1     mv7
......................ccugucugagcgucgcuugccgaucaaaa.........................................................  8     0     mv7
......................ccugucugagcgucgcuugccgaucaaaG.........................................................  1     1     mv7
......................ccugucugagcgucgcuugccgauAaaaa.........................................................  5     1     mv7
......................ccugucugagcgucgcuugccgaucaaaaA........................................................  12    1     mv7
......................ccugucugagcgucgcuugccgaucaaaauA.......................................................  2     1     mv7
.......................Augucugagcgucgcuug...................................................................  7     1     mv7
.......................cugucugagcgCcgcuug...................................................................  2     1     mv7
.......................cugucugagcgucgcuAg...................................................................  2     1     mv7
.......................cugucCgagcgucgcuug...................................................................  1     1     mv7
.......................cugucugCgcgucgcuug...................................................................  2     1     mv7
.......................cugucuCagcgucgcuug...................................................................  1     1     mv7
.......................cugucugagcgucgcuuU...................................................................  54    1     mv7
.......................cugAcugagcgucgcuug...................................................................  2     1     mv7
.......................cugucugagcgucgcCug...................................................................  1     1     mv7
.......................cugucugagcgucgcuug...................................................................  168   0     mv7
```

```
                    Mature                                                         Star
cuggguuccucccagggcuaugccugucugagcgucgcuugccgaucaaaauuccccaggguugccucugggcuccuuggggugcccagcuguucuguggcagggccc
.......................cugucugagcguUgcuug...................................................................   1     1     mv7
.......................Gugucugagcgucgcuug...................................................................   1     1     mv7
.......................cugucugagcgucgcuGg...................................................................   3     1     mv7
.......................cGgucugagcgucgcuug...................................................................   1     1     mv7
.......................cuUucugagcgucgcuug...................................................................   6     1     mv7
.......................cugucugagcgucgcuuC...................................................................   11    1     mv7
.......................cugucugagcgucgcuuA...................................................................   21    1     mv7
.......................cugucugagcgucgcGug...................................................................   2     1     mv7
.......................Uugucugagcgucgcuug...................................................................   4     1     mv7
.......................cugucugagcgucgcuCg...................................................................   4     1     mv7
.......................cugucugagcgucgcCugc..................................................................   2     1     mv7
.......................cCgucugagcgucgcuugc..................................................................   2     1     mv7
.......................Uugucugagcgucgcuugc..................................................................   1     1     mv7
.......................cugAcugagcgucgcuugc..................................................................   4     1     mv7
.......................cugCcugagcgucgcuugc..................................................................   1     1     mv7
.......................cuguUugagcgucgcuugc..................................................................   1     1     mv7
.......................cugucugagcgCcgcuugc..................................................................   1     1     mv7
.......................cugucugagcgucgcuGgc..................................................................   1     1     mv7
.......................cugucugagcgucgcuugU..................................................................   43    1     mv7
.......................cuUucugagcgucgcuugc..................................................................   5     1     mv7
.......................cugucAgagcgucgcuugc..................................................................   1     1     mv7
.......................cugucugagcgucgcuugc..................................................................   190   0     mv7
.......................cugucugagcgucgcuuCc..................................................................   1     1     mv7
.......................cuCucugagcgucgcuugc..................................................................   1     1     mv7
.......................cugucugagcCucgcuugc..................................................................   1     1     mv7
.......................cugucugagcgucgAuugc..................................................................   3     1     mv7
.......................cugucugagcgucgcuugA..................................................................   17    1     mv7
.......................Augucugagcgucgcuugc..................................................................   4     1     mv7
.......................cugucCgagcgucgcuugc..................................................................   2     1     mv7
.......................cugucugagcgucgcGugc..................................................................   1     1     mv7
.......................cugucugagcgucgAuugcc.................................................................   1     1     mv7
.......................cCgucugagcgucgcuugcc.................................................................   1     1     mv7
.......................cugucugagcgucgcuugcG.................................................................   1     1     mv7
.......................cugucugagcgucgcuugAc.................................................................   1     1     mv7
.......................cugucugagcgucgcuAgcc.................................................................   1     1     mv7
.......................cGgucugagcgucgcuugcc.................................................................   1     1     mv7
.......................cuUucugagcgucgcuugcc.................................................................   1     1     mv7
.......................cugucuUagcgucgcuugcc.................................................................   1     1     mv7
.......................cugucugagcgucgcuugcU.................................................................   6     1     mv7
.......................cuAucugagcgucgcuugcc.................................................................   1     1     mv7
.......................cugucugagcgucgcuugcA.................................................................   17    1     mv7
.......................cugucugagcgucgcuugcc.................................................................   33    0     mv7
.......................cugucugagcgucgcCugccg................................................................   1     1     mv7
.......................cugucugagcgucgcuugccA................................................................   5     1     mv7
.......................cugucugagcgucgcuugccg................................................................   12    0     mv7
.......................cugucugagcgucgcuugccU................................................................   1     1     mv7
.......................cugucugagcgucAcuugccg................................................................   1     1     mv7
.......................Augucugagcgucgcuugccg................................................................   1     1     mv7
.......................cugucugagcgucgcuAgccga...............................................................   1     1     mv7
.......................cugucugagcguUgcuugccga...............................................................   1     1     mv7
.......................cugucugagcgucgcuugccga...............................................................   24    0     mv7
.......................Uugucugagcgucgcuugccgau..............................................................   1     1     mv7
.......................cugucugagcguAgcuugccgau..............................................................   1     1     mv7
.......................cugucugagcgucgcuugccgaA..............................................................   8     1     mv7
.......................cugucugagcgucgcuugccgau..............................................................   30    0     mv7
.......................cugCcugagcgucgcuugccgau..............................................................   1     1     mv7
.......................cugucugagcAucgcuugccgau..............................................................   1     1     mv7
.......................Augucugagcgucgcuugccgau..............................................................   1     1     mv7
.......................cugucugagcgucgcuuAccgau..............................................................   1     1     mv7
.......................cugAcugagcgucgcuugccgau..............................................................   1     1     mv7
.......................cugucugagcgucgcuugccgaC..............................................................   4     1     mv7
.......................Uugucugagcgucgcuugccgauc.............................................................   1     1     mv7
.......................cugucugagcgucgcuugccgauU.............................................................   1     1     mv7
.......................cugucGgagcgucgcuugccgauc.............................................................   1     1     mv7
.......................cugucugagcgucgcuugccgauA.............................................................   16    1     mv7
.......................cugucugagcgucgcuugccgauc.............................................................   33    0     mv7
.......................cugucugCgcgucgcuugccgauc.............................................................   1     1     mv7
.......................cugucugagcgucgGuugccgauc.............................................................   1     1     mv7
.......................cugucugagcgucgcuugccgauca............................................................   5     0     mv7
.......................Uugucugagcgucgcuugccgauca............................................................   1     1     mv7
```

Mature   Star

```
cuggguuccucccagggcuaugccugucugagcgucgcuugccgaucaaaauuccccagggguugccucugggcuccuuggggugcccagcuguucuguggcagggccc
.......................cugucugagcguUgcuugccgauca.............................................................   1    1    mv7
.......................cugucugGgcgucgcuugccgauca.............................................................   1    1    mv7
.......................cugAcugagcgucgcuugccgauca.............................................................   1    1    mv7
.......................cugucugagcgucgcuGgccgaucaa............................................................   1    1    mv7
.......................cugucugagcgucgcuugccgaucaa............................................................   6    0    mv7
.......................cugucugagcgucgcuugccgauAaa............................................................   2    1    mv7
.......................cugucugagcgucgcuugccgaucaaa...........................................................   3    0    mv7
.......................cugucugagcgucgcuugccgauAaaa...........................................................   3    1    mv7
.......................cugucugagcgucgcuugccgaucaaC...........................................................   2    1    mv7
.......................cugucugagcgucgcuugccgaucaaaG..........................................................   2    1    mv7
.......................cugucugagcgucgcuugccgaucaaaa..........................................................   5    0    mv7
.......................cugucugagcgucgcuugccgaucaaaC..........................................................   1    1    mv7
.......................cugucugagcgucgcuugccgauAaaaa..........................................................   4    1    mv7
.......................cugucugagcgucgcuugccgaucaaaaA.........................................................   1    1    mv7
........................ugAcugagcgucgcuugc...................................................................   2    1    mv7
........................ugucugagcgucgcuuCc...................................................................   1    1    mv7
........................uguAugagcgucgcuugc...................................................................   12   1    mv7
........................ugCcugagcgucgcuugc...................................................................   2    1    mv7
........................ugGcugagcgucgcuugc...................................................................   3    1    mv7
........................ugucugagcgucgcuugU...................................................................   92   1    mv7
........................ugucugGgcgucgcuugc...................................................................   6    1    mv7
........................ugucugagcgucgcuuAc...................................................................   1    1    mv7
........................Ngucugagcgucgcuugc...................................................................   1    1    mv7
........................ugucuUagcgucgcuugc...................................................................   2    1    mv7
........................ugucugagcgucCcuugc...................................................................   1    1    mv7
........................Cgucugagcgucgcuugc...................................................................   2    1    mv7
........................uAucugagcgucgcuugc...................................................................   1    1    mv7
........................ugucugagcgucgcuCgc...................................................................   2    1    mv7
........................ugucugagcgucgcGugc...................................................................   2    1    mv7
........................ugucugagcgucgcAugc...................................................................   2    1    mv7
........................Agucugagcgucgcuugc...................................................................   1    1    mv7
........................ugucugagcgucgcuugc...................................................................   360  0    mv7
........................ugucugagcgucgcuugG...................................................................   4    1    mv7
........................ugucugagUgucgcuugc...................................................................   1    1    mv7
........................ugucugagcgucgcuugA...................................................................   42   1    mv7
........................ugucugagcgCcgcuugcc..................................................................   1    1    mv7
........................ugucugagcgucgcuugcc..................................................................   95   0    mv7
........................ugucugagcgGcgcuugcc..................................................................   1    1    mv7
........................Agucugagcgucgcuugcc..................................................................   2    1    mv7
........................ugucugagcgucCcuugcc..................................................................   1    1    mv7
........................ugGcugagcgucgcuugcc..................................................................   2    1    mv7
........................ugucugagcgucgcuugcA..................................................................   30   1    mv7
........................ugucugagcgucgcuugUc..................................................................   1    1    mv7
........................ugucugagcgucgcuCgcc..................................................................   2    1    mv7
........................ugucugagcgucgcuuAcc..................................................................   1    1    mv7
........................uguAugagcgucgcuugcc..................................................................   3    1    mv7
........................ugucugagcgucgcuugcU..................................................................   22   1    mv7
........................ugucugagcgucgcuugccC.................................................................   2    1    mv7
........................ugGcugagcgucgcuugccg.................................................................   1    1    mv7
........................Cgucugagcgucgcuugccg.................................................................   2    1    mv7
........................uguAugagcgucgcuugccg.................................................................   2    1    mv7
........................ugucugUgcgucgcuugccg.................................................................   1    1    mv7
........................ugucugGgcgucgcuugccg.................................................................   1    1    mv7
........................ugucugagcgucgcCugccg.................................................................   1    1    mv7
........................ugucugagcgucgcuugccA.................................................................   4    1    mv7
........................ugucugagcgucgcuugccg.................................................................   53   0    mv7
........................ugucugagcgucgcuugAcg.................................................................   1    1    mv7
........................ugucugagcgucgcuugccAa................................................................   1    1    mv7
........................ugucugagcgucgcuugcAga................................................................   3    1    mv7
........................uguAugagcgucgcuugccga................................................................   1    1    mv7
........................ugucugagcgucgcAugccga................................................................   1    1    mv7
........................ugucugagcgucgcuugccga................................................................   69   0    mv7
........................ugucugagcgucgcuuUccga................................................................   1    1    mv7
........................ugGcugagcgucgcuugccga................................................................   3    1    mv7
........................ugucugagcgucgcuCgccga................................................................   1    1    mv7
........................ugucugagcgucgcuugccgG................................................................   2    1    mv7
........................ugucugagcgucgcuugccgaA...............................................................   26   1    mv7
........................ugucugCgcgucgcuugccgau...............................................................   1    1    mv7
........................ugucugagcgucgcuugccgaG...............................................................   2    1    mv7
........................ugucugGgcgucgcuugccgau...............................................................   1    1    mv7
```

```
                    Mature                                                      Star
cuggguuccucccagggcuaugccugucugagcgucgcuugccgaucaaaauuccccagggguugccucugggcuccuuggggugcccagcuguucuguggcagggccc
........................Cgucugagcgucgcuugccgau...............................................................   1     1    mv7
........................ugucugagcgucgcuugccgau...............................................................   61    0    mv7
........................ugAcugagcgucgcuugccgau...............................................................   1     1    mv7
........................ugucugagcgucgcuugccgaC...............................................................   11    1    mv7
........................ugGcugagcgucgcuugccgau...............................................................   1     1    mv7
........................ugucuUagcgucgcuugccgauc..............................................................   1     1    mv7
........................ugucugagcgucgcuugccgaCc..............................................................   1     1    mv7
........................ugucugagcgucgcuugccgauG..............................................................   2     1    mv7
........................ugucugagcgucgcGugccgauc..............................................................   1     1    mv7
........................ugucugagcgucgcuugccgauA..............................................................   16    1    mv7
........................Agucugagcgucgcuugccgauc..............................................................   3     1    mv7
........................ugucugagcgucgcuugAcgauc..............................................................   1     1    mv7
........................uguGugagcgucgcuugccgauc..............................................................   1     1    mv7
........................ugucugagcgucgcuugccgauc..............................................................   120   0    mv7
........................uAucugagcgucgcuugccgauc..............................................................   2     1    mv7
........................ugAcugagcgucgcuugccgauc..............................................................   2     1    mv7
........................ugCcugagcgucgcuugccgauc..............................................................   1     1    mv7
........................ugGcugagcgucgcuugccgauc..............................................................   8     1    mv7
........................ugucugUgcgucgcuugccgauc..............................................................   1     1    mv7
........................ugucugagcgucCcuugccgauc..............................................................   3     1    mv7
........................ugucugagcgucgcuugGcgauc..............................................................   1     1    mv7
........................ugucugGgcgucgcuugccgauc..............................................................   1     1    mv7
........................ugucugagcgucgcuugccAauc..............................................................   1     1    mv7
........................ugucCgagcgucgcuugccgauc..............................................................   1     1    mv7
........................ugucugagcgucgcuugccgauU..............................................................   8     1    mv7
........................uguAugagcgucgcuugccgauc..............................................................   6     1    mv7
........................ugucugagcgucgcuGgccgauc..............................................................   2     1    mv7
........................Cgucugagcgucgcuugccgauca.............................................................   1     1    mv7
........................ugucugagcgucgcuuCccgauca.............................................................   1     1    mv7
........................ugucugagcgucgcuugccgauAa.............................................................   1     1    mv7
........................ugucugagcgucgcuugccgauca.............................................................   16    0    mv7
........................ugucugagcgucgcuugccgauGaa............................................................   1     1    mv7
........................ugucugagcgucgcuugccgauAaa............................................................   6     1    mv7
........................ugucugagcgucgcuugccgaucaa............................................................   26    0    mv7
........................ugucugagcgucgcuugccgaucGa............................................................   1     1    mv7
........................Agucugagcgucgcuugccgaucaa............................................................   1     1    mv7
........................ugucugagcgucgcuugccgaucaCa...........................................................   1     1    mv7
........................ugucugagcgucgcCugccgaucaaa...........................................................   1     1    mv7
........................ugucugagcgucgcuugccgaucaaG...........................................................   2     1    mv7
........................ugGcugagcgucgcuugccgaucaaa...........................................................   1     1    mv7
........................ugucugagcgucgcuugccgaucaaC...........................................................   2     1    mv7
........................ugucugagcgucgcuugccgaucaaa...........................................................   31    0    mv7
........................ugucugagcgucgcuugccgauAaaa...........................................................   11    1    mv7
........................ugucugagcgucgcuugccgauGaaa...........................................................   1     1    mv7
........................ugucugagAgucgcuugccgaucaaaa..........................................................   1     1    mv7
........................Agucugagcgucgcuugccgaucaaaa..........................................................   1     1    mv7
........................ugucugagcgucgcuugccgaucaaaa..........................................................   23    0    mv7
........................ugGcugagcgucgcuugccgaucaaaa..........................................................   1     1    mv7
........................ugucugagcgucgcuugccgaucGaaa..........................................................   1     1    mv7
........................ugucugagcgucgcuugccgaucaaaC..........................................................   1     1    mv7
........................ugucugagcgucgcuugccgaucaaaG..........................................................   1     1    mv7
........................ugucugagcgucgcuugccgauAaaaa..........................................................   6     1    mv7
........................ugucugagcgucgcuugccgaucaaaaA.........................................................   10    1    mv7
.........................gucugagcgucgcuugcU..................................................................   12    1    mv7
.........................gucugagcgCcgcuugcc..................................................................   1     1    mv7
.........................gucugagcgucgcuAgcc..................................................................   1     1    mv7
.........................gucugagcgucgcuugcA..................................................................   41    1    mv7
.........................gucugagcgucgcuCgcc..................................................................   2     1    mv7
.........................gucugagcgucgcuugcc..................................................................   37    0    mv7
.........................gucugagcgucgcuugcG..................................................................   1     1    mv7
.........................gucCgagcgucgcuugcc..................................................................   1     1    mv7
.........................gucugagcgucgcAugcc..................................................................   1     1    mv7
.........................gucugagcgucgcuGgcc..................................................................   1     1    mv7
.........................gucugagcgucgcCugcc..................................................................   1     1    mv7
.........................gucugagcgucgAuugccg.................................................................   1     1    mv7
.........................gucugaAcgucgcuugccg.................................................................   1     1    mv7
.........................gucugagcgucUcuugccg.................................................................   1     1    mv7
.........................gucugagcgucgcuugccA.................................................................   11    1    mv7
.........................gucugagcgucgcuugcUg.................................................................   1     1    mv7
.........................gucugagcgucgcuAgccg.................................................................   1     1    mv7
```

Mature Star

```
cuggguuccucccagggcuaugccugucugagcgucgcuugccgaucaaaauuccccagggguugccucugggcuccuuggggugcccagcuguucuguggcagggccc
.........................gucugagcgucgcuugccg.................................................................  36   0  mv7
.........................Cucugagcgucgcuugccg.................................................................  1    1  mv7
.........................gucugagcgucgcuugcAg.................................................................  1    1  mv7
.........................gucugGgcgucgcuugccg.................................................................  1    1  mv7
.........................gucugagcgucgcuugccU.................................................................  1    1  mv7
.........................gucugUgcgucgcuugccga................................................................  1    1  mv7
.........................gucugagcgucgAuugccga................................................................  1    1  mv7
.........................gucugagcgucgcuugGcga................................................................  1    1  mv7
.........................gucugagcgucgcuugccgU................................................................  1    1  mv7
.........................gucugagcgucgcuugccga................................................................  57   0  mv7
.........................Cucugagcgucgcuugccga................................................................  1    1  mv7
.........................gucugagcgucgcuCgccga................................................................  1    1  mv7
.........................gCcugagcgucgcuugccga................................................................  1    1  mv7
.........................gucugagcgucgcuugccgaG...............................................................  1    1  mv7
.........................gucugagcguGgcuugccgau...............................................................  1    1  mv7
.........................gucugagcgucgcuugccgaA...............................................................  24   1  mv7
.........................gucugagcgucgcuugccgau...............................................................  25   0  mv7
.........................gucugagcgucgcuugccgaC...............................................................  2    1  mv7
.........................gucugagcgucgcuugccgGuc..............................................................  1    1  mv7
.........................gucugagcgucgcGugccgauc..............................................................  1    1  mv7
.........................gucugagcgCcgcuugccgauc..............................................................  1    1  mv7
.........................gucugagcgucgcuugccgauA..............................................................  17   1  mv7
.........................Aucugagcgucgcuugccgauc..............................................................  1    1  mv7
.........................gucAgagcgucgcuugccgauc..............................................................  2    1  mv7
.........................gucugagcgucgcuugccgUuc..............................................................  1    1  mv7
.........................gucCgagcgucgcuugccgauc..............................................................  1    1  mv7
.........................gucugagcgucgAuugccgauc..............................................................  1    1  mv7
.........................guGugagcgucgcuugccgauc..............................................................  3    1  mv7
.........................gucugagcgucgcuuCccgauc..............................................................  1    1  mv7
.........................gucugagcgucgcuugccgauG..............................................................  2    1  mv7
.........................Cucugagcgucgcuugccgauc..............................................................  2    1  mv7
.........................gucugagcgucgcuugccgauc..............................................................  100  0  mv7
.........................Uucugagcgucgcuugccgauc..............................................................  1    1  mv7
.........................gucugagcgucgcuugccgaCc..............................................................  1    1  mv7
.........................gucugagcgucgcuCgccgauc..............................................................  1    1  mv7
.........................gucugagcgucgcuugcUgauc..............................................................  1    1  mv7
.........................gucugagcgucgcuugccgauU..............................................................  4    1  mv7
.........................gucugUgcgucgcuugccgauc..............................................................  1    1  mv7
.........................guAugagcgucgcuugccgauc..............................................................  1    1  mv7
.........................gucugagcgucgcuugUcgauca.............................................................  1    1  mv7
.........................gucugagcgucgcuugccgauca.............................................................  13   0  mv7
.........................gucugagcgucgcuugccgauAa.............................................................  2    1  mv7
.........................gucugagcgucgcuugccgaucaa............................................................  11   0  mv7
.........................gucAgagcgucgcuugccgaucaa............................................................  1    1  mv7
.........................gucugagcgucgcuugccgauAaa............................................................  3    1  mv7
.........................gucugagcgucgcuugccgauGaaa...........................................................  1    1  mv7
.........................gucugagcgucgcuugccgauAaaa...........................................................  10   1  mv7
.........................gucugagcgucgcuugccgaucaaa...........................................................  17   0  mv7
.........................gucugagcgucgcuugccgaucaaaa..........................................................  10   0  mv7
.........................gucugagcgucgcuAgccgaucaaaa..........................................................  1    1  mv7
.........................gucugagcgucgcuugccgaucaaaU..........................................................  1    1  mv7
.........................gucugagcgucgcuugccgaucaGaa..........................................................  3    1  mv7
.........................gucugagcgucgcuugccgaucaaaaA.........................................................  13   1  mv7
.........................gucugagcgucgcuugccgaucaaaaAu........................................................  1    1  mv7
..........................ucugagcgucgcuugccg.................................................................  10   0  mv7
..........................ucugagcgucgcuugccA.................................................................  1    1  mv7
..........................ucugagcgucgcuugccga................................................................  6    0  mv7
..........................ucugagcgucgcuugccgau...............................................................  6    0  mv7
..........................ucugagcgucgcuugccgaA...............................................................  8    1  mv7
..........................ucugagcgucgcuuAccgau...............................................................  1    1  mv7
..........................ucGgagcgucgcuugccgauc..............................................................  1    1  mv7
..........................ucugagcgucgcuugccgauA..............................................................  3    1  mv7
..........................ucugagcgucgcuugccgauc..............................................................  29   0  mv7
..........................ucugagcgucgcuuAccgauc..............................................................  1    1  mv7
..........................ucugagcgucgcuugccgauca.............................................................  1    0  mv7
..........................ucugagcgucgcuugccgauAaa............................................................  1    1  mv7
..........................ucugagcgucgcuugccgauAaaa...........................................................  1    1  mv7
..........................ucugagcgucgcuugccgauAaaaa..........................................................  1    1  mv7
..........................ucugagcgucgcuugccgaucaaaa..........................................................  1    0  mv7
...........................cugCgcgucgcuugccga................................................................  1    1  mv7
```

Mature Star

cuggguuccucccagggcuaugccugucugagcgucgcuugccgaucaaaauuccccaggguugccucugggcuccuuggggugcccagcuguucuguggcagggccc			
...........................cugagcgucgcuugccga...	5	0	mv7
...........................cuUagcgucgcuugccga...	1	1	mv7
...........................Uugagcgucgcuugccgau..	1	1	mv7
...........................cugagcgucgcuugccgaA..	3	1	mv7
...........................cugagcgucgcuugccgau..	8	0	mv7
...........................cugagcgucgcuugccUauc...	1	1	mv7
...........................cugagcguAgcuugccgauc...	1	1	mv7
...........................cugagcgucgcuuAccgauc...	1	1	mv7
...........................cugCgcgucgcuugccgauc...	3	1	mv7
...........................cugagcgucgcuugccgauA...	7	1	mv7
...........................cugagcgucgcuugccgauc...	10	0	mv7
...........................Augagcgucgcuugccgauc...	1	1	mv7
...........................cugagcAucgcuugccgauc...	1	1	mv7
...........................Augagcgucgcuugccgauca..	1	1	mv7
...........................cugagcgucgcuugccgaucaaa..	1	0	mv7
...........................cugagcgucgcuugUcgaucaaaa...	1	1	mv7
............................ugagcgucgcuugccgaA..	6	1	mv7
............................ugaCcgucgcuugccgauc...	1	1	mv7
............................ugagcgucgcuugccgauc...	4	0	mv7
............................ugagcgucgcuugccgauA...	1	1	mv7
............................ugagcgucgcuugccgauca..	1	0	mv7
............................ugagcgucgcuugccgaucaa...	2	0	mv7
............................ugagcgucgcuugccgauAaa...	1	1	mv7
............................ugagcgucgcuugccgauAaaa..	2	1	mv7
............................ugagcgucgcuugccgauAaaaa...	1	1	mv7
............................ugagcgucgcuugccgaucaaaa...	1	0	mv7
.............................gagcgucgcuugccgauc...	1	0	mv7
.................................Cucgcuugccgaucaaaa...	1	1	mv7
....ccaggguugccucugggcuG.................................	1	1	mv7
....gcuguucuguggcaggAc..	1	1	mv7
