# Supplementary material for: SEAweb: the small RNA Expression Atlas web application
Source: Nucleic Acids Res. 2019 Oct 10;48(D1):D204–19. doi: 10.1093/nar/gkz869 (PMC6943056; doi:10.1093/nar/gkz869)
Supplement: gkz869_Supplemental_Files [file gkz869_supplemental_files.zip › SEAweb_Supplementary_material.pdf]

Supplementary material

1. Acquisition and analysis of sRNA datasets

SEAweb acquires raw published sRNA-seq datasets and their primary annotation from Gene Expression Omnibus (GEO) and NCBI's Sequence Reads Archive (SRA) repository. GEO makes two databases in SQLite format available for download: GEOmetadb for annotations and SRAdb for SRA sequences. An automated data acquisition pipeline searches for new sRNA data bi-weekly, keeping SEAweb continuously updated. In order to retrieve relevant samples for downloading, we optimized our search queries to look for the datasets that have, (i) Experiment type as non-coding RNA profiling by high throughput sequencing, (ii) Sequencing platform as Illumina, (iii) Tissue, cell type, disease or cell line information and (iv) is one of the 10 organisms that SEAweb supports at the moment (Table 1). This search criterion is based on regular expressions and sometimes returns false positive datasets. We manually delete completely irrelevant samples such as 'Expression profiling by high throughput sequencing' or samples belonging to an organism apart from the 10 organisms that we can support now. Some of the false positives can still be used, such as samples, which are small RNAs, but they do not have any tissue or disease related information. Following the acquisition of sRNA datasets, the SEAweb analysis workflow automatically analyzes new files using the Oasis 2.0 (http://oasis.ims.bio/). The SEAweb analysis workflow determines data quality, detects and quantifies sRNAs, including the prediction of novel, high-quality miRNAs. We used the DNApi-based

adapter prediction functionality in Oasis 2 to automatically detect and remove adapter sequences (1, 2). Oasis 2 reports many quality measures for each sample, including a summary statistics table, containing information on the total number of reads, percentage of trimmed reads (adapter trimming), and percentage of uniquely aligned reads. In general, low percentages of trimmed and/or uniquely mapped reads indicate problematic samples. When samples show low percentages of trimmed or mapped reads, the adapter or reference genome might not be corresponding to the input sample. These low quality files are flagged automatically and subjected to manual curation. In the manual curation step, we re-run the sample(s) of the corresponding dataset with the actual adapter mentioned in the original work. Any files not passing manual curation are removed from SEAweb. Subsequently, sRNA counts of high-quality samples along with their quality information are stored in the sRNA expression database. Further quality control (QC) includes principal component analysis (PCA) for all the samples in an experiment, the number of reads trimmed for being too short (15 nucleotides) or too long (40 nucleotides), the percentage of adapters trimmed, and the number and percentage of uniquely mapped reads. SEAweb provides these QC metrics for all the experiments stored in its databases. Users can check this for each dataset by clicking on the Oasis output link, for example http://sea.ims.bio/analysed/uploaded/DET__GSE46579_5adb777d65246/summary.html. We took special care to exclude low quality samples from SEAweb using automatic quality prediction and manual curation. In addition, users can exclude and re-analyze all SEAweb datasets with at least two groups (conditions) using the

multi lasso functionality of SEAweb. The selected samples can then be analyzed for differential expression or classification using Oasis 2.

Pathogenic signatures from bacteria and viruses, supplying information on potentially infected samples is also incorporated into SEAweb. Additionally, SEAweb stores expression information of high-quality predicted miRNAs including the ID, organism, chromosomal location, precursor and mature sequences, structure, read counts, prediction scores, and detailed information on the software and its versions used to predict the miRNA. SEAweb's primary analysis results including per sample quality and expression information can be examined and downloaded as interactive web reports. Detailed information on the primary analysis of sRNAs and predicted miRNAs can be found in the Oasis 2 manuscript (1).

2. Differential expression and Classification

sRNA data differential expression (DE) and classification are obtained by passing obtained sRNA counts to DE and classification modules of Oasis. As biological conditions for comparisons, group annotations (Section 2.3) are used. This means that several comparisons inside of one dataset are possible. The results of DE analysis are mean value, fold change, p value and p adjusted value for each sRNA. The results of classification analysis are Gini index decrease for each sRNA.

Those results show importance of the corresponding sRNA in distinguishing between two biological conditions. SEAweb stores the above-mentioned results together with group information, including annotations of conditions that were

compared, list of samples in each group for provenance as well as DE and classification module version and initial settings for reproducibility.

3. Semantic data layer

Given diversity of biological data, the users of the corresponding information systems should be given a possibility to interpret data independently using common terminologies. For this purpose we developed a semantic data layer, which provides unified access to data together with metadata and interpretations. Metadata is normalized using ontologies, which makes annotations standardized and hierarchically searchable. Data interpretations are represented by DE and classification result, described in Section 2.

3.1. Ontologies

In biological research, controlled biological vocabularies such as terminologies, ontologies, taxonomies play an important role for annotation, integration, and analysis of biological data. In order to allow for the interoperability of data, one important step is to standardize annotations using ontologies and semantic mapping (3). Ontologies define standard terms, their properties, and the relations between them. Each term in the ontology has a unique identifier, and annotation terms that are connected to ontologies are called ‘normalized’. Another advantage of ontology use is a possibility to search by parent terms. Ontology is a hierarchical structure, where children are more specific as parents. Usually parents are connected to children using one of the two relations: “**is-a**” or “**part-of**”. “is-a” relation

means that a child represents more specific term as a parent, for example Alzheimer’s disease **is a** tauopathy, tauopathy **is a** neurodegenerative disease. “part-of” relation means that a child is part of a parent, for example neocortex is **part of** a cerebral cortex, cerebral cortex is **part of** a brain.

In order to enable the search across ontological hierarchies we integrated the relevant ontologies into the graph database Neo4j. Graph databases are NoSQL databases which support storage of objects and connections between them, as is the case for ontologies. Following the manual curation, sample annotations are uploaded to the SEAweb annotation graph database including all ontological parent terms (having an ‘is-a’ or ‘part of’ relation to it). This allows search by ontology terms, as well as by their parents, which are in fact groups of terms (e.g. ‘cancer’ or ‘neurodegenerative disease’). SEAweb accesses ontologies via the Ontology Lookup Service using a REST interface, supporting complex and compound queries and query auto-completion.

3.2. Annotation Process

SEAweb’s sRNA annotation workflow maps free-text GEO annotations to standardized terms in three consecutive steps. In general, GEO data annotations are free text that can be parsed into key-value pairs. In a first fully automated step the annotation workflow extracts key-value relations and stores them in the annotation database. These key-value relations are extracted from an experiment or sample description text. It is unstructured

and contains variety of information: therefore, we opted for a NoSql annotation database with an optimized indexing for prototypical questions (supplementary section 2.4). The second fully automated step normalizes the extracted keys and values using ontologies as standard dictionaries. SEAweb has a list of predefined keys, five of which (organism, tissue, disease, cell type, and cell line) can be currently queried for in SEAweb. Each extracted key is compared to predefined keys. For values, the ontologies are used as standard terminology dictionaries. For each pre-defined key, one or several corresponding ontologies are used. Each extracted value is searched in the corresponding ontologies and, if the same or a similar term is found, we normalize the value by it.

Automatic annotation is followed by semi-automatic manual curation. For that purpose, we developed an internal curation Web interface (Supplementary Fig 1) using Groovy/Grails, which allows browsing and editing of annotations from the annotation database as well as manual normalization of keys and values in annotations, searching among pre-defined keys and corresponding ontologies. Thus, curators examine all keys and values for consistency and update missing or additional information with standardized terms where necessary (e.g. organism, tissue, cell type, cell line, disease). At the moment, all SEAweb annotations are manually curated, a quality standard that we intend to keep for every future SEAweb entry.

3.3. Annotation criteria

Some basic rules for annotation of sRNA-seq samples are discussed below:

1. Annotations can be defined as experiment-level or sample-level annotations. Experiment level annotations are exactly the same for all samples of the dataset. Sample-level annotations differ among the samples of the dataset.
2. In exceptional situations, when experiment-level and sample-level annotations have the same key, the corresponding sample will have two annotations with that key (local annotation does not override the global one).
3. In cases, where alternatives exist for an annotation term, we tried to be as specific as possible. For example, in case the sample is from breast fibrosarcoma and the term “breast fibrosarcoma” is available we will try to annotate it with the same term, although it can be annotated with breast cancer as well i.e. we choose the term deepest in the ontology tree.
4. In cases where the relevant term cannot be found in any ontology, we tried to normalize the terms with synonyms or slightly less specific. The aim was to annotate as much as possible to have standard terms rather than just textual information

3.4. Annotation of groups

Apart from sRNA expression, SEAweb contains also results of differential expression and classification between different biological conditions. In

order to make these comparisons automatically possible, biologically meaningful comparisons are also annotated manually. This annotation belongs to a dataset and we call it "sample group". Sample group annotation includes annotation fields, which are important to define groups to compare. For example:

1. In the case an experiment has healthy and diseased patients. The desired comparison will be to perform differential expression or classification based on these two groups of samples. In this case, sample group is annotated as disease. The field disease contains two distinct values – disease name (for example, Alzheimer's disease) and "healthy". And correspondingly, one comparison will be made.
2. In the case an experiment contains two diseases and healthy controls. Sample group is annotated as disease. The field disease contains three values – disease 1 name (for example, Alzheimer's disease) disease 2 name (for example, Frontotemporal Dementia) and "healthy". Correspondingly, three comparisons will be made: healthy vs. disease 1, healthy vs. disease 2 and disease 1 vs. disease 2.
3. In the case an experiment contains one disease with two stages and healthy controls. In this case, sample group is annotated as disease + disease details. Correspondingly, three groups will be created: disease+stage1, disease+stage2, healthy. Correspondingly, three

comparisons will be made: healthy vs. disease stage1, healthy vs. disease stage2 and disease stage1 vs. disease stage2.

3.5. Querying and visualization

Application programming interfaces (APIs) were developed to access data in SEAweb databases. We developed **annotation-API** to access annotation-DB, **association-API** to access association-DB, **expression-API** to access expression-DB, **predictedMirna-API** to access Oasis-DB and **SEAweb business logic API** to call all these APIs based on the end user request and then combine their results (responses) back to the end user. Each API answer particular queries as explained below.

1. **Annotation-API** to access Annotation-DB, and answer questions like
    a. Get all the experiments for term (will return experiments and samples along with annotation details for term as well as for the sub-type of term). Term can be disease, tissue, cell line, cell type, organism or their combination
2. **Association-API** to access Association-DB, and respond to questions like
    a. Get all gene targets for a miRNA.
    b. Get all diseases that are associated with a miRNA from literature.

c. Get all miRNAs that are associated with a disease or it's sub-types.
d. Get genomic coordinates for sRNA or target genes.

3. **Expression-API** to access Expression-DB and shows
    a. Get expression of one or more sRNAs in a particular or all experiments.
    b. Get list of experiments where a particular sRNA is differentially expressed.
    c. Get list of experiments where a particular sRNA is identified as potential biomarker via classification.
    d. Get expression of a pathogen in a particular or all experiments.
    e. Get list of all experiments where a particular pathogen is differentially expressed.
4. **PredictedMirna-API** to access Oasis-DB (1) and shows
    a. Get list of all novel predicted miRNAs from Oasis-DB
    b. Get genomic coordinates and sequence for predicted miRNA(s).
5. **User-API** to access User-DB and gets
    a. Get list of all user specific experiments (uploaded by the user).
    b. Get list of experiments where a particular sRNA is differentially expressed in their own uploaded data.

c. Get list of experiments where a particular sRNA is identified as potential biomarker via classification in their own uploaded data.

6. **SEAweb business logic API:** was built in order to put all those APIs together and make necessary data transformations between frontend and other APIs. As a result, the user can make queries to answer biological questions like;

   a. What is expression of one more sRNAs in specific cell types or tissues?
   b. Is a particular sRNA differentially expressed in Alzheimer's disease?
   c. Compare sRNAs across different studies?
   d. What are differentially expressed sRNAs in breast cancer and healthy women?
   e. Common differentially expressed sRNAs or potential sRNAs based biomarker across particular disease or tissue.
   f. Expression of one or more novel miRNAs for known diseased states.
   g. Analysis results with all the quality information for 350 datasets and over 4200 samples.

Supplementary Figures

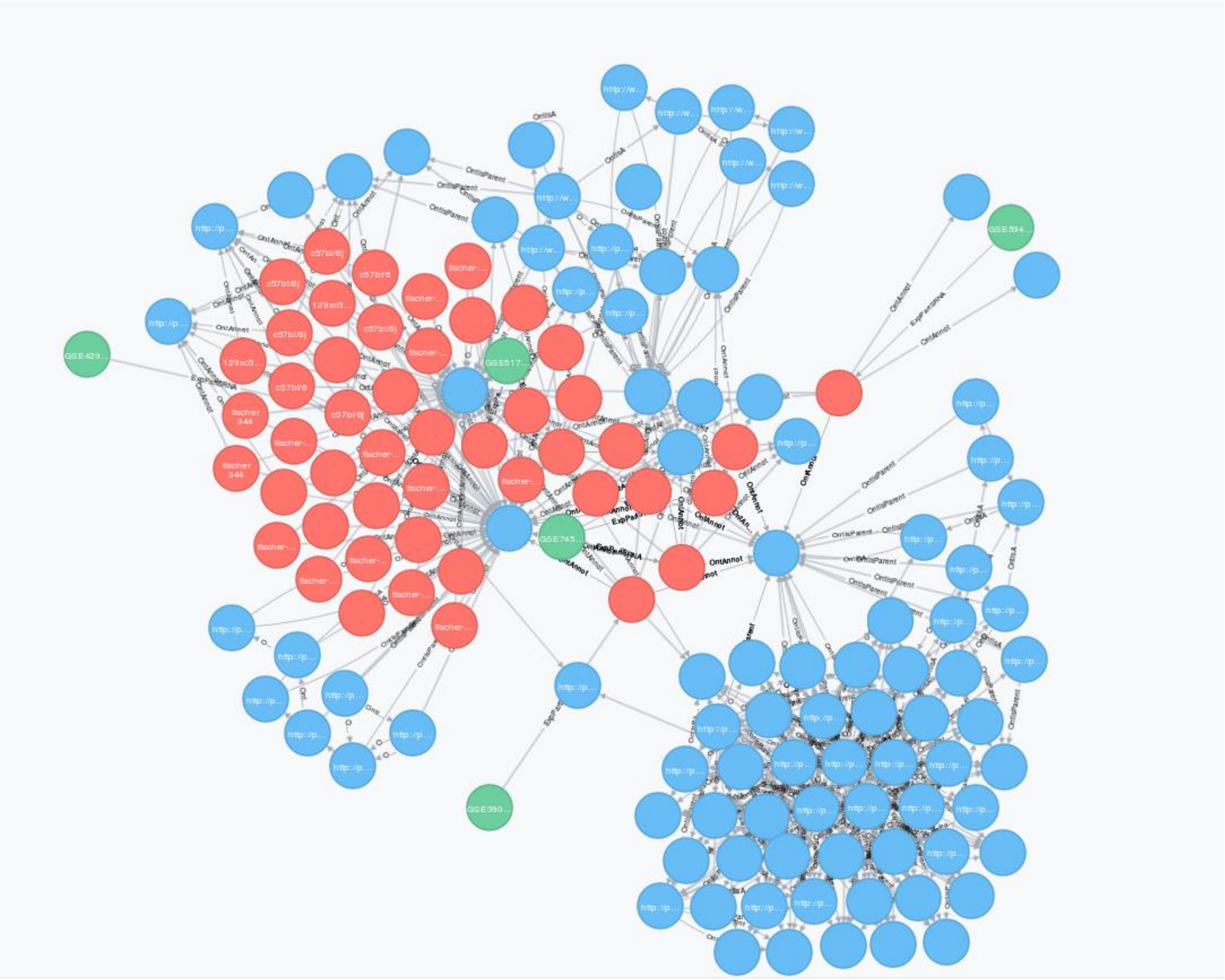

Supplementary Fig. S1: Annotation-DB. Objects in the SEAweb graph database (Neo4j). A fragment of the SEAweb graph database is visualized, where green nodes represent datasets, red nodes represent samples and blue nodes represent ontology terms. Grey edges represent ‘is a’ relations between the different datasets, samples, and ontology terms.

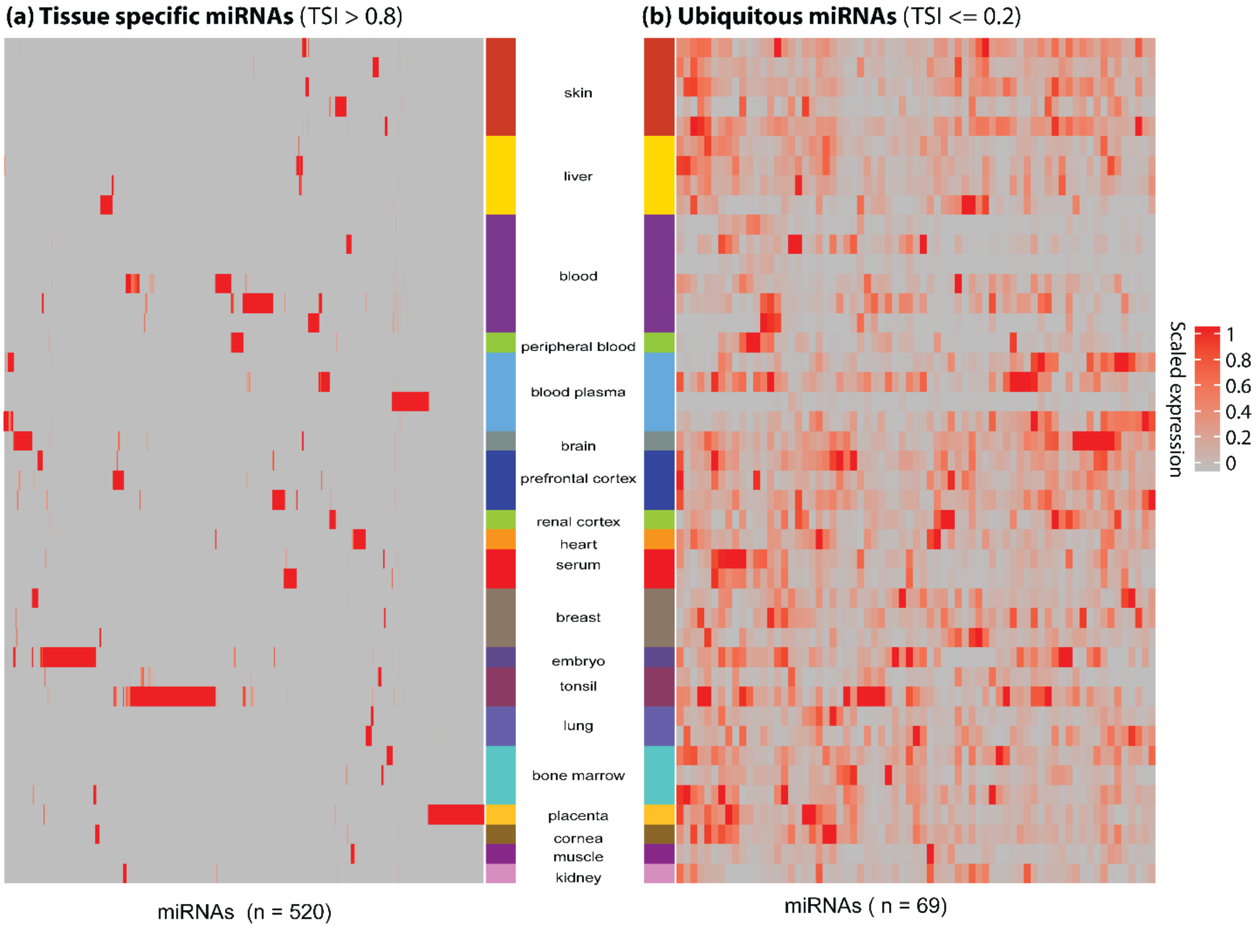

Supplementary Fig. S2: Tissue specific miRNAs. The heatmaps show the scaled expression (0-1) of (a) tissue specific or (b) ubiquitous miRNAs across all the tissues. (**a) Tissue specific miRNAs**. miRNA expression across all the tissues with TSI > 0.8 (n=520). (**b) Ubiquitous miRNAs.** miRNA expression across all the tissues with TSI <= 0.2 (n=69). miRNA names are omitted for simplicity. A complete list of tissue specific and ubiquitous miRNAs with their Shannon entropy score can be found in Supplementary Table 1. These calculations are based on the non-disease samples of an experiment (material and methods).

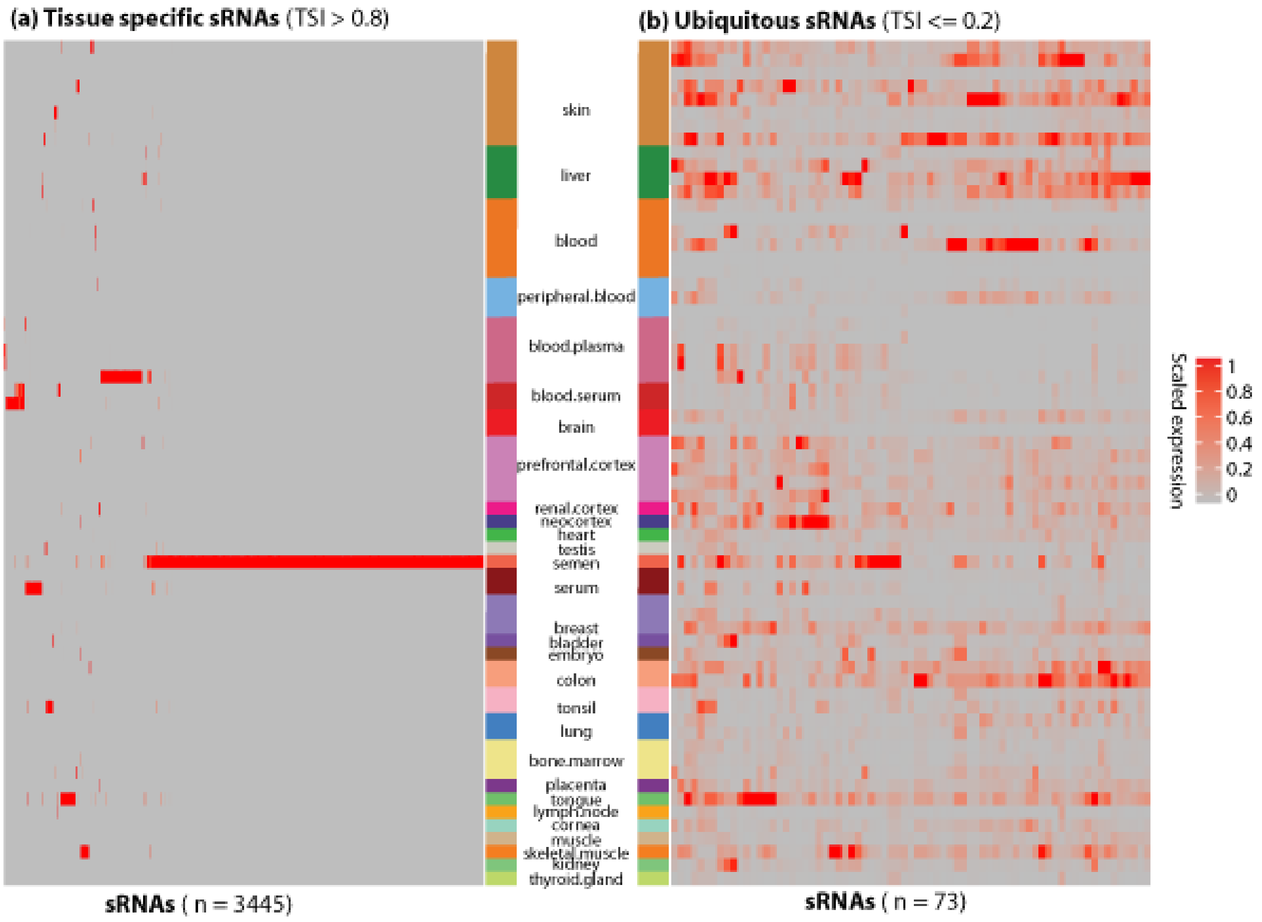

Supplementary Fig. S3: Tissue specific small RNA (sRNAs). The heatmaps show the scaled expression (0-1) of (a) tissue specific or (b) ubiquitous sRNA (piRNA, snoRNA, snRNA and rRNA) across all the tissues. **(a) Tissue specific sRNAs**. sRNA expression across all the tissues with TSI > 0.8 (n=3445). **(b) Ubiquitous sRNAs.** sRNA expression across all the tissues with TSI <= 0.2 (n=73). sRNA names are omitted for simplicity. A complete list of tissue specific and ubiquitous sRNAs with their Shannon entropy score can be found in Supplementary Table 4. These calculations are based on healthy and disease samples within an experiment (material and methods).

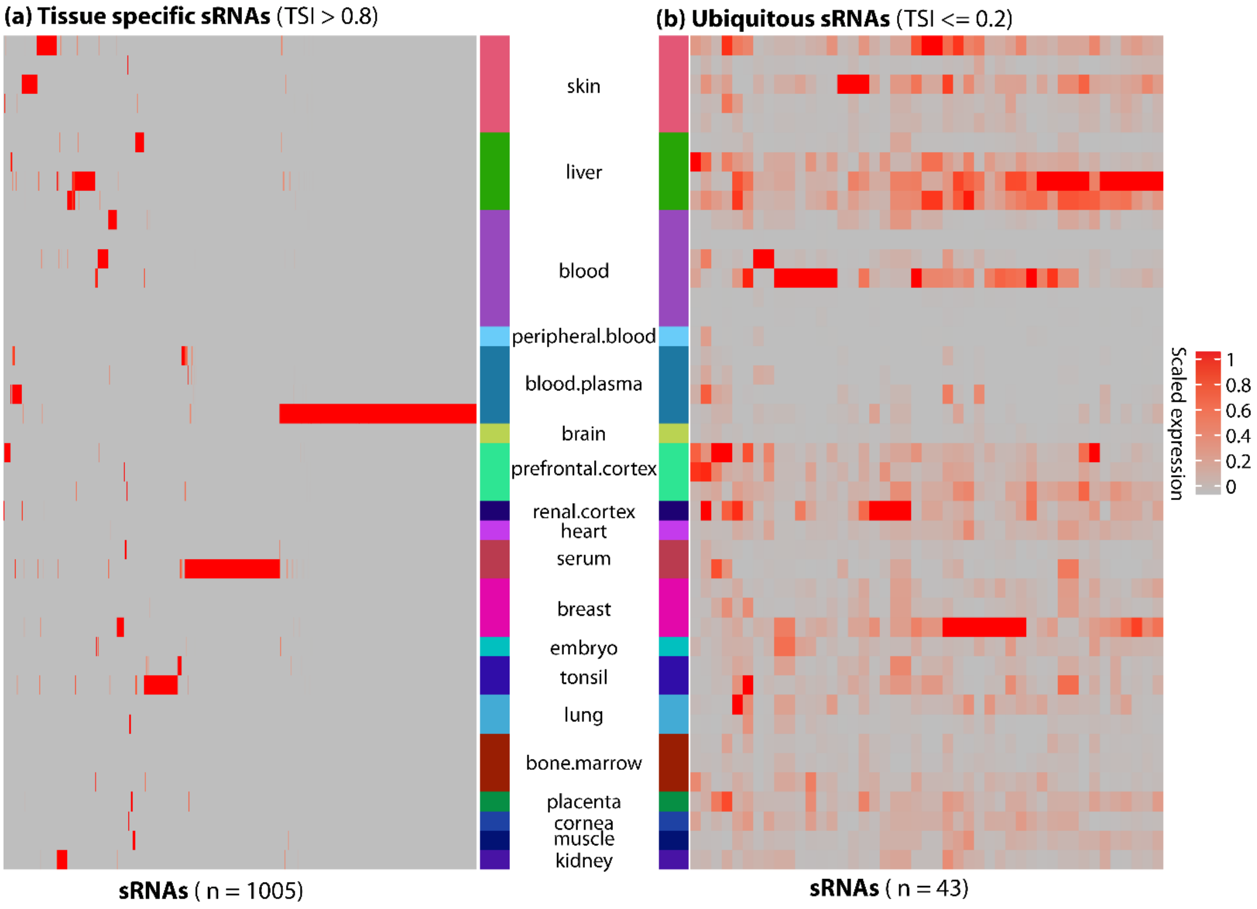

Supplementary Fig. S4: Tissue specific small RNA (sRNAs). The heatmaps show the scaled expression (0-1) of (a) tissue specific or (b) ubiquitous sRNA (piRNA, snoRNA, snRNA and rRNA) across all the tissues. **(a) Tissue specific sRNAs**. sRNA expression across all the tissues with TSI > 0.8 (n=1005). **(b) Ubiquitous sRNAs.** sRNA expression across all the tissues with TSI <= 0.2 (n=43). sRNA names are omitted for simplicity. A complete list of tissue specific and ubiquitous sRNAs with their Shannon entropy score can be found in Supplementary Table 4. These calculations are based on the non-disease samples of an experiment (material and methods).

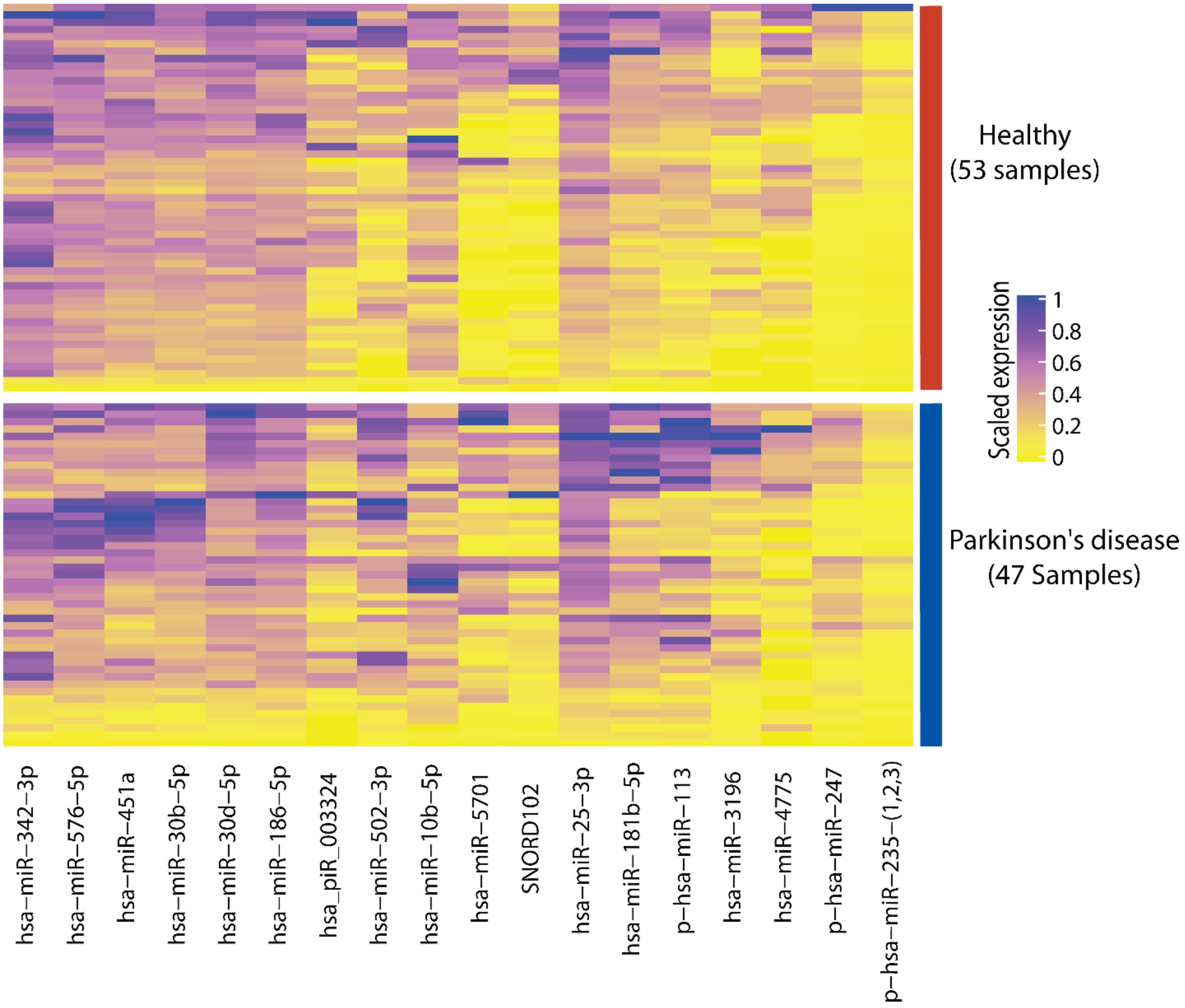

Supplementary Fig. S5: Parkinson's disease biomarkers (important features). The heatmap shows scaled expression values (0 to 1) of the 18 features (sRNS) identified by random forest algorithm in 53 healthy and 47 parkinson's disease samples. Expression levels were scaled between 0 and 1 for each sRNA. Of note, 16 out of these 18 sRNAs are not differentially expressed, but RF still identify these as important features.

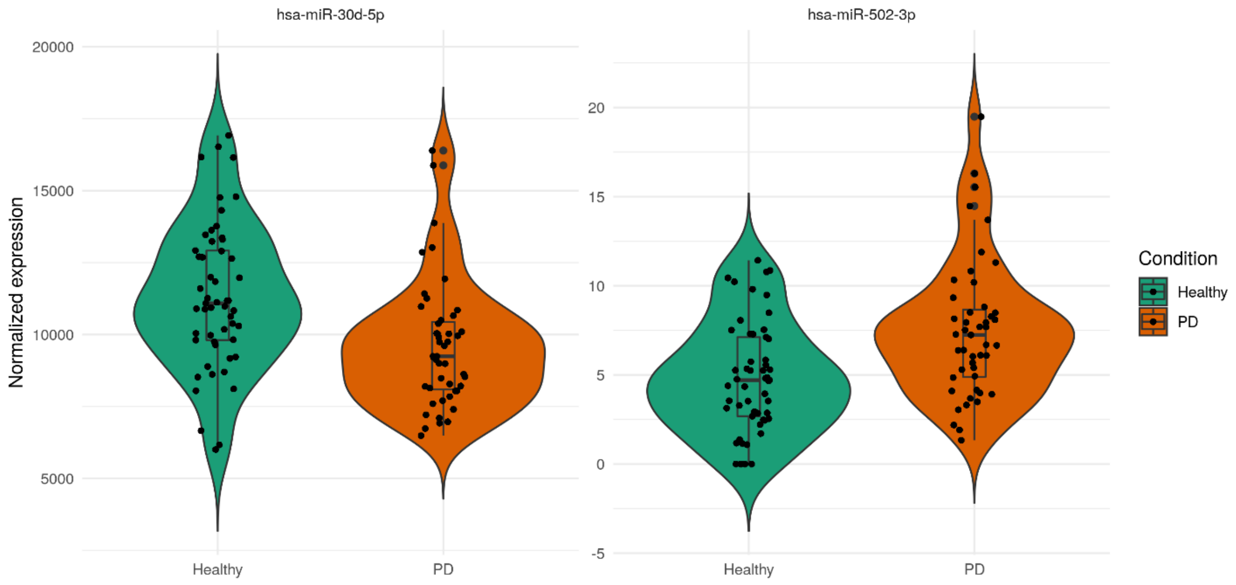

Supplementary Fig. S6: Expression profiles of two differentially expressed sRNAs. Shown are the DESeq2 normalized expression values for the two differentially expressed sRNAs between the 53 healthy and 47 PD samples. hsa-miR-30d-5p is significantly down-regulated in PD while hsa-miR-502-3p is up-regulated. Both sRNAs are also included in the 18 sRNA Random Forest biomarker of PD.

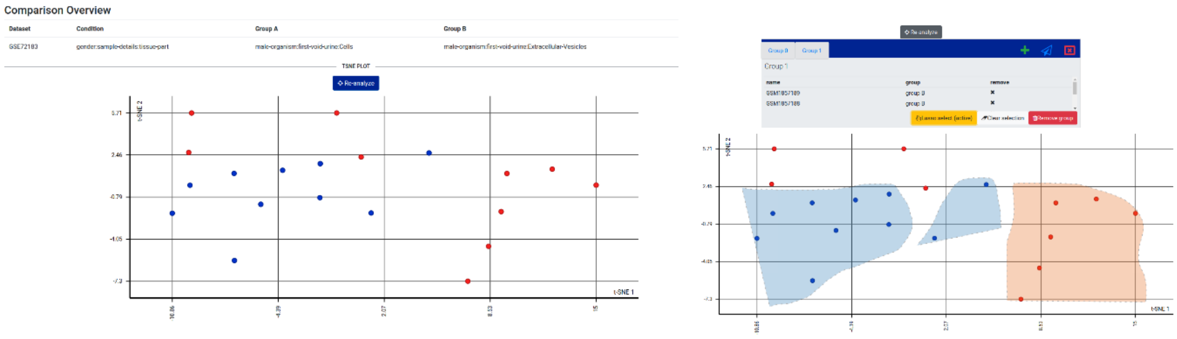

Supplementary Fig. S7: Resubmission of existing data. Left: t-SNE plot of the samples in two groups in a differential expression comparison. **Right**: Selection of specific samples (from the same comparison) to remove outliers or to make novel analysis with different combination of samples.

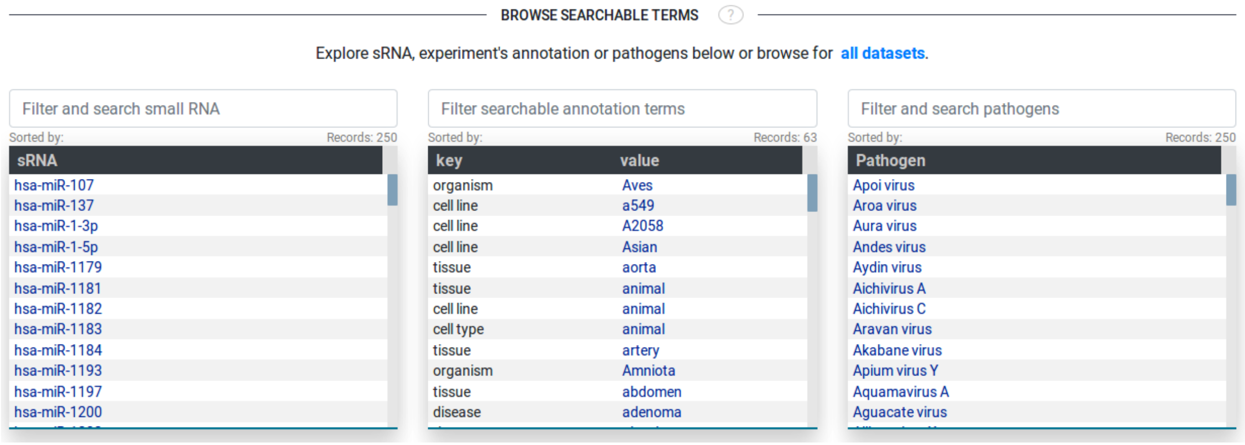

Supplementary Fig. S8: Three panel browsing. Small RNAs (left), annotation terms (middle), and pathogens (right). Users can select a single or multiple terms from the browsing panels, click on any term will add it to the SEAweb search bar and can be used in the next request by the user. For example, the user can click on a small RNA and cancer to see its expression profiles in the cancer datasets. User can browse for all the datasets and samples by clicking on "all datasets" link.

Tables

Supplementary Table S5. Comparison of number of sRNA samples per organism.

Organism	SEAweb	miRmine[1]	DASHR2[2]	miratlas[3]	YM500v3[4]	SPAR[5]
Bos taurus	51					
Caenorhabditis	131					
Danio rerio	19					
Drosophila	174					
Mus musculus	372			118		365[$]
Gallus gallus	31					
Rattus norvegicus	50					
Homo sapiens	3328	304	802	343	>8000	365[$]
Sus scrofa	94					
Anopheles gambiae	8					

This table includes number of samples for each organism across all the tools compared with SEAweb. [$]Number of datasets based on [2](4), for both Homo sapiens and Mus musculus, information about number of samples cannot be obtained for each organism from the original work. [1](5), [2](4), [3](6), [4](7), [5](8).

1. Rahman,R.-U., Gautam,A., Bethune,J., Sattar,A., Fiosins,M., Magruder,D.S., Capece,V., Shomroni,O. and Bonn,S. (2018) Oasis 2: improved online analysis of small RNA-seq data. *BMC Bioinformatics*, **19**, 54.
2. Tsuji,J. and Weng,Z. (2016) DNApi: A De Novo Adapter Prediction Algorithm for Small RNA Sequencing Data. *PLoS One*, **11**, e0164228.
3. Schuurman,N. and Leszczynski,A. (2008) Ontologies for bioinformatics. *Bioinform. Biol. Insights*, **2**, 187–200.
4. Kuksa,P.P., Amlie-Wolf,A., Katanić,Ž., Valladares,O., Wang,L.-S. and Leung,Y.Y. (2019) DASHR 2.0: integrated database of human small non-

coding RNA genes and mature products. *Bioinformatics*, **35**, 1033–1039.

5. Panwar,B., Omenn,G.S. and Guan,Y. (2017) miRmine: a database of human miRNA expression profiles. *Bioinformatics*, **33**, 1554–1560.
6. Vitsios,D.M., Davis,M.P., van Dongen,S. and Enright,A.J. (2017) Large-scale analysis of microRNA expression, epi-transcriptomic features and biogenesis. *Nucleic Acids Res.*, **45**, 1079–1090.
7. Chung,I.-F., Chang,S.-J., Chen,C.-Y., Liu,S.-H., Li,C.-Y., Chan,C.-H., Shih,C.-C. and Cheng,W.-C. (2017) YM500v3: a database for small RNA sequencing in human cancer research. *Nucleic Acids Res.*, **45**, D925–D931.
8. Kuksa,P.P., Amlie-Wolf,A., Katanić,Ž., Valladares,O., Wang,L.-S. and Leung,Y.Y. (2018) SPAR: small RNA-seq portal for analysis of sequencing experiments. *Nucleic Acids Res.*, **46**, W36–W42.
